# Phylogenomic evolutionary surveys of subtilase superfamily genes in fungi

**DOI:** 10.1038/srep45456

**Published:** 2017-03-30

**Authors:** Juan Li, Fei Gu, Runian Wu, JinKui Yang, Ke-Qin Zhang

**Affiliations:** 1 State Key Laboratory for Conservation and Utilization of Bio-Resources in Yunnan, Yunnan University, Kunming, 650091, P.R. China

## Abstract

Subtilases belong to a superfamily of serine proteases which are ubiquitous in fungi and are suspected to have developed distinct functional properties to help fungi adapt to different ecological niches. In this study, we conducted a large-scale phylogenomic survey of subtilase protease genes in 83 whole genome sequenced fungal species in order to identify the evolutionary patterns and subsequent functional divergences of different subtilase families among the main lineages of the fungal kingdom. Our comparative genomic analyses of the subtilase superfamily indicated that extensive gene duplications, losses and functional diversifications have occurred in fungi, and that the four families of subtilase enzymes in fungi, including proteinase K-like, Pyrolisin, kexin and S53, have distinct evolutionary histories which may have facilitated the adaptation of fungi to a broad array of life strategies. Our study provides new insights into the evolution of the subtilase superfamily in fungi and expands our understanding of the evolution of fungi with different lifestyles.

To survive, fungi depend on their ability to harvest nutrients from living or dead organic materials. The ecological diversification of fungi is profoundly affected by the array of enzymes they secrete to help them obtain such nutrients. Thus, the differences in their secreted enzymes can have a significant impact on their abilities to colonize plants and animals^1^. Among these enzymes, a superfamily of serine proteases named subtilases, ubiquitous in fungi, is suspected of having distinct functional properties which allowed fungi adapt to different ecological niches. For many saprophytes, subtilases are the principal broad-spectrum proteases which play a role in nutrition acquisition, such as digesting proteins to release peptides and amino acids for fungal cell absorption and growth^2^,^3^. In many pathogenic fungi, subtilases are believed to play important roles in host invasion^3^,^4^. For instance, subtilases derived from several entomopathogous and nematophagous fungi have been characterized as having the ability to disrupt the physiological integrity of insect and/or nematode cuticles during penetration and colonization. Thus, they are also called cuticle-degrading proteases^5^,^6^,^7^,^8^,^9^.

According to the MEROPS peptidase classification^10^, subtilases are divided into two clans: S8 (subtilisins) and S53 (sedolisins). Clan S8 utilizes a catalytic triad of three amino acids, Aspartate, Histidine, and Serine (DHS catalytic triad), to catalytically hydrolyze peptide bonds, while the catalytic triad of the S53 clan is Serine, Glutamate and Aspartate (SED catalytic triad). The S8 proteases are further classified into S8A and S8B subclans^11^. At present, subclan S8A has been divided into several distinct families such as proteinase K-like proteases, pyrolisin proteases and oxidatively stable proteases (OSP), whereas S8B subclan is composed of those proteases belonging to the kexin subfamily^1^,^3^,^12^.

Proteinase K-like protease, which was first identified in the ascomycete *Tritirachium album*, is a large subfamily of endopeptidases found in fungi^2^. Proteases within this subfamily may play important roles during the evolution of pathogenicity. The cuticle-degrading proteases mentioned above belong to the proteinase K-like subfamily^5^,^6^,^7^,^8^,^9^. Pyrolisin, also named class I subtilisin, was first characterized from the hyperthermophilic archaeon *Pyrococcus furiosus*. It is the most thermostable protease identified to date and retains half of its activity after boiling for several hours. A characteristic feature of Pyrolisin is the fact that these enzymes are substrates of their own proteolytic activity and degrade themselves in a process termed autoproteolysis^13^. However, although Pyrolisin has long been known in fungi, their ecological function is still uncertain^3^. Kexin, which was first identified in the yeast *Saccharomyces cerevisiae*, can process the yeast precursors of alpha-mating factor and killer toxin, as well as play a significant role in post-translational modification in eukaryotes^14^. OSP proteases were identified in the endophytic fungus *Epichloë festucae* and other Hypocreales fungi^1^. S53 family proteases probably contribute to the extracellular digestion of food proteins. Recently, based on the differences in the closest amino acids from the DHS catalytic triad, six new subtilase groups (named new 1 to new 6) were identified by Muszewska *et al*.^15^. Previous studies suggested that most of the subtilases in fungi were extracellular secreted proteases, which were thought to play a nutritive role^16^. However, there are several intracellular proteases localized to the vacuole^4^. The vacuolar proteases appear to play a specialized role in the breakdown of autophagic bodies in the vacuole during autophagy^17^. Moreover, most members of the subtilase superfamily are inhibited by general serine peptidase inhibitors such as diisopropyl fluorophosphate (DFP) and phenylmethane sulfonylfluoride(PMSF), but kexin is resistant to PMSF and requires high concentrations of DFP, which initially led to its misidentification as a cysteine peptidase^18^.

In previous studies, a large variation in the number of subtilase genes was observed among fungal species^1^,^3^,^4^, implying that many changes have likely occurred in subtilases during fungal evolution. It is generally accepted that gene duplication and loss were the main sources of the gene copy number changes among species^19^, and were a significant source of functional innovation^20^,^21^,^22^. In view of this, although the biological functions of most fungal subtilases are not yet described, gene duplication and sequence diversification have likely resulted in functional diversifications of fungal subtilases beyond simple nutrient utilization. Further, these functional differences among subtilases likely enabled fungi to develop new life strategies and adapt to a broad array of habitats as saprophytes and pathogens. Therefore, investigation of the evolution of the subtilase superfamily may help us understand the specialization and adaptation of fungi. In recent years, the increasing availability of completely sequenced fungal genomes provided a good opportunity for us to characterize the subtilases superfamily in different fungal taxa and to infer their evolutionary patterns. In this study, by analyzing the distribution of subtilases genes among 83 fungal species, we investigated the evolutionary patterns of the main subtilase families, and also examined the possible gene duplication and loss which may have contributed to the functional divergence of these subtilase genes.

## Data and Methods

### Identification of subtilase genes

Based on the phylogeny of the fungal kingdom which constructed by James *et al*.^23^ and Spatafora *et al*.^24^, we selected representatives of the whole genome sequenced fungi that are distributed among all the main fungal lineages, including the main phyla of Ascomycota, Basidiomycota, Mucormycotina and the very ancestral Microsporidia for this study. A total of 83 fungal genome sequences and the corresponding annotated protein databases are retrieved from the Fungal Genome Initiative at the BROAD Institute (http://www.broad.mit.edu/annotation/fungi/fgi), Fungal Genome Research (http://fungalgenomes.org/), and the NCBI database.

To find putative homologs of subtilases in the 83 fungal genome sequences employed in this study, the HMM profile Peptidase_S8 (PF00082; http://pfam.xfam.org/family/PF00082#curationBlock), which includes both S8 and S53 domains, was downloaded and used as a query in the search for homologous proteins using the program HMMSEARCH from the HMMER package (http://hmmer.wustl.edu/). Hits were considered significant when they matched the Pfam HMM profile with E values <10^−5^.

CLANS, a Java-based software program which visualizes pair-wise sequence similarities^25^, was used to elucidate the relationships between and within subfamilies of the S8 peptides. Also, the amino acid sequences of each subgroup were aligned using MUSCLE Version 3.7^26^, and those sequences with ambiguously aligned regions around the three active catalytic residues (Asp-His-Ser triad in S8 and Ser-Glu-Asp triad in S53) were eliminated in the subsequent analyses.

The conserved functional domain structures of the identified subtilases were predicted using the MEME/MAST motif discovery tool (http://meme.sdsc.edu)^27^ and InterProScan 5.0 (http://www.ebi.ac.uk/interpro/) with default parameter settings^28^.

### Phylogenetic analysis

Before phylogenetic analyses, MUSCLE v3.7 was used to generate protein alignment with default settings^26^. The ambiguous areas of alignment were located and removed by using the program Gblocks 0.91b^29^,^30^ with default parameters. The gap selection criterion “with half” was used here. Finally, alignments with 184 amino acid sequences were identified for the proteinase K group in Sordariomycetes fungi, and 282, 465, and 363 amino acid sequences were respectively identified for the pyrolisin, kexin and S53 families among the 83 included fungal genomes.

In this study, the aligned amino acid sequences of each gene family/subfamily were performed using two different tree construction methods: Neighbor-joining (NJ) analysis and Maximum likelihood (ML) analysis. For NJ analysis, the data was analyzed using MEGA 6^31^ using bootstrap analysis (BS) with 1,000 replicates. For ML analysis, the best-fit models of protein evolution for each subfamily were first estimated using the program ProtTest^32^, then the recommended models and parameters were used for ML analysis with PHYML 3.0^33^. In the ML analysis, the reliability of the tree topology was evaluated using bootstrap support with 100 repeats^34^.

## Results

### Number of subtilase genes from different fungi

**Figure 1:**
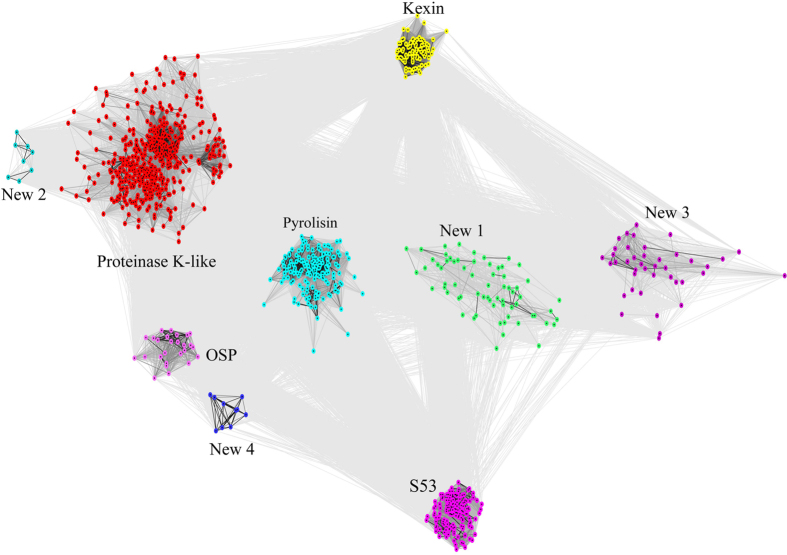
CLANS clustering of 993 subtilase sequences obtained from 83 whole genome sequenced fungal species. The name of each group is marked. Four new groups are identified in our study with new 1, new 3 and new 4 groups being the same as the classification of Muszewska *et al*., and new 2 group in our study corresponding to the new 6 group proposed by Muszewska *et al*.^15^.

We identified the near-complete repertories of subtilase genes from 83 fungal species with different life-styles. After using HMM profile Peptidase_S8 as query model for Hmmsearch, a total of 993 subtilase genes were identified. To elucidate the relationships among the 993 subtilase genes, a clustering analysis which relied on the sequence similarities of proteins was conducted using the CLANS program^25^. As seen in Fig. 1, cluster analysis of the 993 subtilase genes resulted in nine distinct clades. The domain architectures of each clade were analyzed in our study (Supplementary Fig. S1). Although all the S8 family subtilases contain the common DHS catalytic triad, the closest amino acid sequences around the three catalytic amino acids were not the same. For example, the closest amino acid sequences from the DHS catalytic triad of the sequences grouped together in the proteinase K-like clade are Y/I/LDT/SG, HGT/SH/AV/CA/SG/A, and GT/SS, respectively. Also, beside the canonical DHS catalytic triads, the proteinase K-like subtilases contain an N-terminal subtilisin propeptide Inhibitor_I9 (InterPro acc. no.: IPR010259), which is thought to act as an intramolecular chaperone to assist protein folding as well as inhibit enzyme activity^35^. For the Pyrolisin proteases, the sequences around the catalytic triads are I/V/LDT/SGD/N/W, HGT/SH/F/A, and GT/SSM/F/YA/S, respectively, and they usually contain a Fn3-like domain (InterPro acc. no.: IPR010435), which maybe functionally related to adhesin/invasin^36^. For kexins, the catalytic triads are V/I/LDDGL/YD, HGTRCAG/AE/QI/VA/S/GA, and HGGTSAAA/GP, respectively. Moreover, kexins usually have a pro-protein C-terminal convertase domain (P_protein; InterPro acc. no.: IPR002884) and a Galactose binding-like domain (InterPro acc. no.: IPR008979). For OSP proteases, beside the canonical peptidase S8 domain, most of them also contain a Galactose binding-like domain (InterPro acc. no.: IPR008979). Also, few of them contain a Ankyrin repeat domain(InterPro acc. no.: IPR002110) which is one of the most common protein-protein interaction motifs in nature. For S53 proteases, the canonical SED catalytic triads are EA/S/GXLD, S/A/GSGD, and GTSA/CS/AA/S/TP, respectively(Supplementary Fig. S1). Generally, combined with the results based on CLAN and their motif feature of each clade, these nine distinct clades were divided into different subfamilies/families: S53 proteases were clustered into a single compact clade, while the genes of the S8 peptides were divided into eight separate clades: proteinase K proteases, pyrolisin proteases, kexin, OSP, and four newly identified groups (new 1 to new 4).

Because the DHS and SED catalytic triads are the key active centers of S8 and S53 proteases, respectively, any mutation within or around the region encoding the catalytic triads may result in the inactivation of a protein. Thus, those genes with ambiguously aligned regions around the three active catalytic residues were considered to be pseudogenes and removed from subsequent analyses. Finally, a total of 904 putative functional subtilase genes (Supplementary data S2), including 429 proteinase K-like subtilases, 26 OSPs, 136 pyrolisins, 84 kexins, 126 S53 proteases, 53 new 1 group proteases, 9 new 2 group proteases, 33 new 3 group proteases and 8 new 4 group proteases were used in subsequent analyses. The identified number of subtilases for the 83 fungal species are presented in Table 1 (see Supplementary Table S3). These variations imply that gene duplication and loss may have played significant roles during the evolution of the fungal subtilases.

Among the 83 analyzed fungal genomes, remarkable variations in the number of subtilase genes were observed among different fungal species (Table 1 and Supplementary Table S3). The entomopathogous fungus *Metarhizium robertsii* had the largest number of subtilase genes (48), whereas the Saccharomycotina fungus *Saccharomyces kluyveri* contained only 1 subtilase gene. Among the families in the S8 clan, the numbers of proteinase K-like subfamily genes in fungal species were the most variable, ranging from 0 to 19 (Table 1 and Supplementary Table S3). This proteinase K-like subfamily of genes was common to all the species examined in this study, suggesting its functional importance. In our study, Eurotiomycetes and Sordariomycetes fungi showed a large variation in the number of proteinase K-like subfamily genes, and the fungi which contained fewer than 10 subtilase genes were mainly saprophytic fungi, such as *Aspergillus* spp., *Penicillium* spp. and *Neurospora crassa.* The two phytopathogenic fungi (*Phaeosphaeria nodorum* and *Pyrenophora tritici-repentis*) from Dothideomycetes contained 5 proteinase K-like genes each, whereas the other two phytopathogenic fungi (*Botryotinia fuckeliana* and *Sclerotinia sclerotiorum*) from Leotiomycetes contained 1 and 2 genes, respectively. Intriguingly, the only Orbiliomycetes fungi among the 83 species, *Arthrobotrys oligospora,* contained 19 duplicated proteinase K-like subtilases. All the yeast-like fungi shared a highly similar number of proteinase K-like subtilases except *Yarrowia lipolytica*, which contained 16 duplicate proteins. Similarily, all the Basidiomycota fungi contained 1 to 3 proteinase K-like subtilases except *Coprinus cinereus* which contained 6. Moreover, ten proteinase K-like subtilases were identified from the two Mucormycota fungi *Phycomyces blakesleeanus* and *Rhizopus oryzae*, while 1 gene was identified from the Chytridiomycota fungus *Batrachochytrium dendrobatidis*. For the three Microsporidia fungi, only 1 proteinase K-like subtilase was identified from *Encephalitozoon cuniculi* while no proteinase was found from the other two.

For pyrolisin, a large variation in its numbers was found in the Sordariomycetes fungi, ranging from 1 to 16. The pyrolisin genes in *Verticillium* spp., *Metarhizium* spp., and *Magnaporthe* spp. have significantly expanded and the rice blast fungus *Magnaporthe grisea* possessed 16 pyrolisin subfamily genes, to date the highest number of this gene subfamily from a single species. However, we did not find pyrolisin subfamily genes in most Eurotiomycetes fungi except for *Aspergillus terreus* and there was a complete absence of the pyrolisin genes in all yeast-like fungi (Saccharomycotina and Taphrinomycotina). Interestingly, this subfamily of genes also showed a large variation in Basidiomycota fungi: the phytopathogenic fungus *Puccinia graminis* contained 8 pyrolisins while *Postia placenta, Sporobolomyces roseus*, and *Malassezia globosa* contained none. In comparison, the ancestral Mucorales fungi *R. oryzae* and *P. blakesleeanus* contained 8 and 9 pyrolisin genes, respectively. Interestingly, regardless of taxonomic affiliation, none of the human/animal pathogenic fungi possessed pyrolisin genes.

Most fungi investigated in this study contained 1 to 3 kexin protease genes, with the exception of the Basidiomycota fungus *Phanerochaete chrysosporium* and the Saccharomycotina fungi *S. kluyveri, Saccharomyces kudriavzevii* and *Saccharomyces mikatae*, none of which contained any kexin genes. For the three fungi from Microsporidia, although 1 kexin-like subtilase was identified in *Enterocytozoon bieneusi* and *Encephalitozoon intestinalis* each, these two sequences showed ambiguous alignment aroud the three active catalytic residues (Asp-His-Ser). Moreover, no kexin-like sequence was identified in *E. cuniculi*.

In our results, genes from OSP, new 1, new 3 and new 4 groups were present in a few species of Pezizomycotina fungi. However, genes from new 2 were mainly found in Basidiomycota and Mucormycota fungi.The four Taphrinomycotina fungi contained one new 2 group gene each and the Chytridiomycota fungus *B. dendrobatidis* also contained one.

S53 family genes were commonly present in Pezizomycotina fungi with the exceptions of *Verticillium* spp. and *M. poae*. Like Pyrolisin genes, the S53 family genes was completely absent from all yeast-like fungi (Saccharomycotina and Taphrinomycotina). Some Basidiomycota fungi, such as *Laccaria bicolor, P. chrysosporium, P. placenta* and *P. graminis*, also contained S3 family genes and *P. placenta* contained the largest number (23) of S53 family genes.

### Phylogenetic analysis

In our results, genes from the proteinase K, pyrolisin, kexin and S53 families were much more commonly identified in fungal genomes than other subtilase clusters (Fig. 1 and Table 1). Consequently, subsequent phylogenetic analyses were performed based on those four subfamilies. Moreover, the proteinase K subfamily genes from Sordariomycetes fungi showed significant variation compared with those from other species, suggesting that the proteinase K genes may have played important roles during the evolution of Sordariomycetes fungi. Thus, the proteinase K genes from Sordariomycetes fungi were used to construct phylogenetic trees and those genes identified from Saccharomycotina were used as outgroups. The best-fit models for each family, chosen by the program ProtTest, were provided in Supplementary Table S4.

### Proteinase K-like subfamily

**Figure 2:**
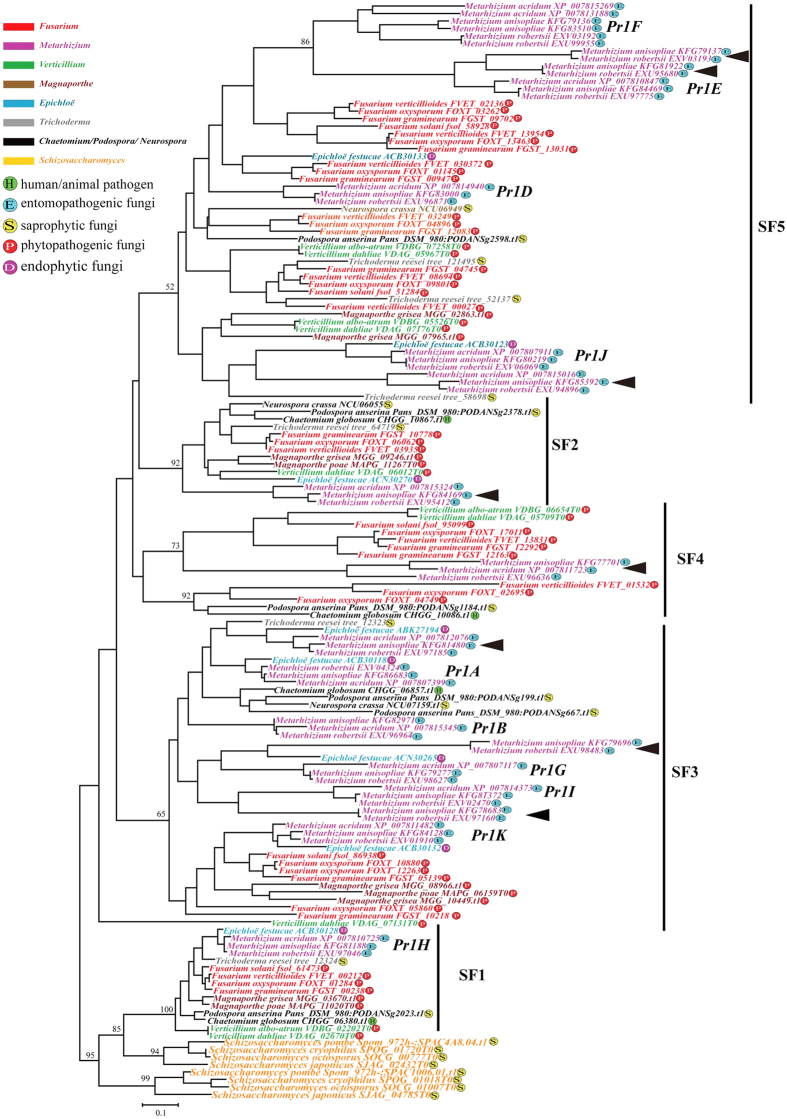
Phylogenetic relationships of the proteinase K-like subfamily in Sordariomycetes fungi. Phylogenetic analyses were performed using maximum likelihood (ML) methods as implemented in PHYML 3.0 with GTR+I+G model based on an alignment of 184 amino acids from 138 proteinase K-like genes from Sordariomycetes fungi and 8 outgroup genes from the four Taphrinomycotina fungi. Five clades were designated (SF1-SF5). The bootstrap support value for each clade is shown. The 10 proteinase K-like subfamily genes *Pr1A, Pr1B, Pr1D* to *Pr1K* and the duplicated genes newly identified in *Metarhizium* spp. are highlighted.

Phylogenetic relationships among the proteinase K-like subfamily genes were analyzed based on an alignment consisting of 184 amino acids from 138 genes (Supplementary data S5) from Sordariomycetes fungi and 8 outgroup genes from the four Taphrinomycotina fungi. Phylogenetic analyses grouped these genes into five distinct clades, which are abbreviated as SF1 to SF5 (Fig. 2 and Supplementary Fig. S6). Among these clades, SF1 (BS = 100% in both NJ and ML) consisted of the specialized vacuolar proteases from the Sordariomycetes fungi except for *N. crassa* and showed close relationships with the outgroup genes from Taphrinomycotina fungi. Clade SF2 (BS = 96% in NJ and BS = 92% in ML) to SF5(BS = 59% in NJ and BS = 52% in ML) were composed of the duplicated proteinase K-like subfamily genes in Sordariomycetes fungi, and inconsistent relationships among the four subfamilies were observed (Fig. 2 and Supplementary Fig. S6). However, given that duplicate genes were commonly represented in the four clades, it was assumed that four duplication events might have occurred at the beginning of the Sordariomycetes lineage. Moreover, within the SF3 and SF5 clades, there were several genus-specific subclades which were composed of the duplicate genes identical to those found inpathogenic fungi such as *Fusarium* spp., *Metarhizium* spp., *Verticillium* spp., and *E. festucae*, implying that the genes were duplicated before the divergence of the species within same genus.

Also, several gene loss events were identified in some species. For example, the duplicate genes formed into SF1 (BS = 100% in both NJ and ML) all came from the Sordariomycetes fungi considered in this study except *Fusarium solani* and *Verticillium albo-atrum*. In contrast, the genes from *N. crassa, E. festucae, M. grisea, M. poae,* and *Trichoderma reesei* were not included in SF4. These results suggest a subsequent loss of proteinase K-like genes in these fungi. The duplicate genes from the endophytic fungus *E. festucae* always showed close relationships with the duplicated genes from *Metarhizium* spp., suggesting the genes duplicated before the *E. festucae* and *Metarhizium* spp. split. However, in some subclades, no *E. festucae* genes were found to cluster with the duplicated genes of *Metarhizium* spp., suggesting subsequent gene losses in *E. festucae.* In the clade SF3 (BS = 78% in NJ and BS = 65% in ML) and SF5 (BS = 59% in NJ and BS = 52% in ML), the genes belonging to genus *Metarhizium* have largely expanded, and some genes have also been lost in *Metarhizium acridum*. Moreover, in clade SF5, at least five duplications occurred before the divergence of *Fusarium* species but *F. solani* retains only two of the duplications.

### Pyrolisin subfamily

**Figure 3:**
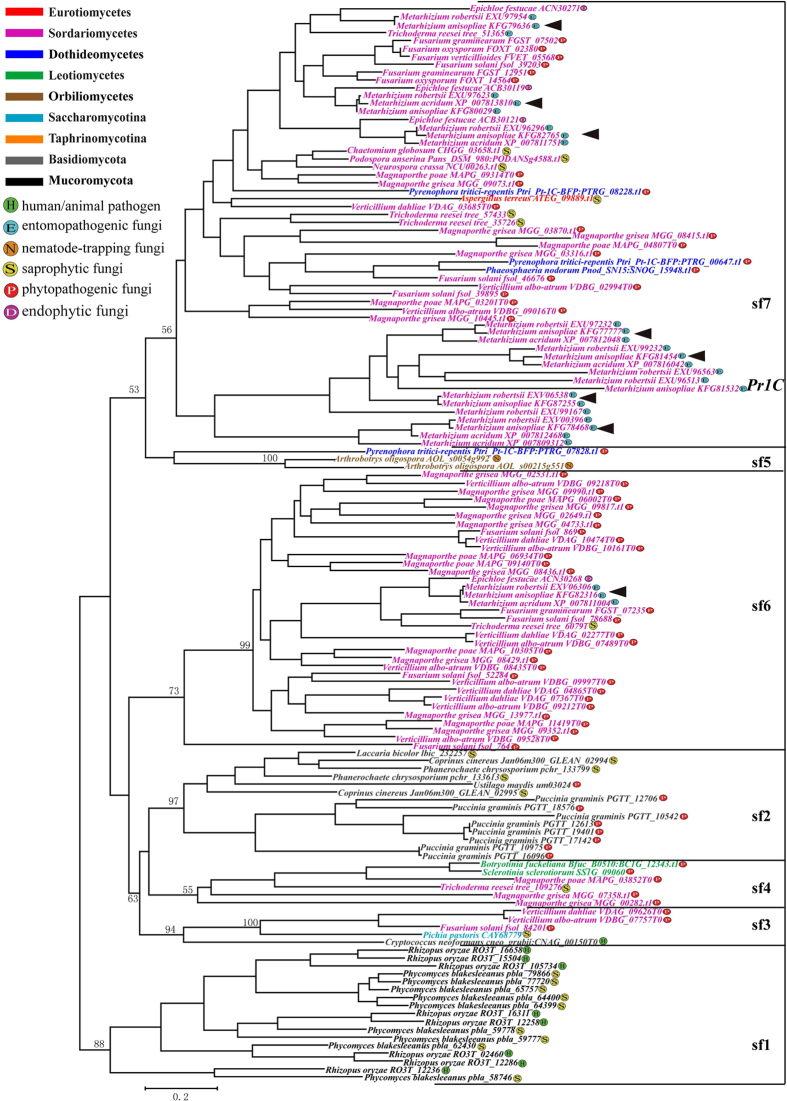
Phylogenetic relationships of the Pyrolisin subfamily. Phylogenetic analyses were performed using maximum likelihood (ML) methods as implemented in PHYML 3.0 with GTR+I+G model based on an alignment of 282 amino acids from 136 Pyrolisin sequences. Seven clades were designated (sf1-sf7) and the bootstrap support value for each clade is shown. The Pyrolisin subfamily genes *Pr1C* and the duplicated genes newly identified in *Metarhizium* spp. are highlighted.

Our phylogenetic analysis based on an alignment of 282 amino acids from 136 Pyrolisin sequences (Supplementary data S7) suggested that the pyrolisin genes are mainly grouped into seven clusters which were designated sf1 to sf7 though inconsistent relationships among the six clades were also observed (Fig. 3 and Supplementary Fig. S8). Among these seven clades, sf1 (BS = 88% in NJ and BS = 90% in ML) is the Mucorales-specific lineage which contains the duplicated genes from *R. oryzae* and *P. blakesleeanus*. The pyrolisin genes from Basidiomycota fungi fall into the sf2 clade (BS = 94% in NJ and BS = 97% in ML). Within this clade, the 8 duplicated genes of *P. graminis* formed one species-specific subcluster, suggesting the duplication occurred after the speciation of *P. graminis.* The gene identified from *Pichia pastoris* (CAY68779) and *Cryptococcus neoformans* (CNAG_00150T0) is clustered into sf3 (BS = 95% in NJ and BS = 94% in ML) with the genes from *Verticillium* spp. (*VDAG_09626T0* in *Verticillium dahliae* and *VDBG_07757T0* in *V. albo-atrum*) and *F. solani* (*fsol_84201*). Clade sf4 (BS = 63% in NJ and BS = 55% in ML) consisted of the six genes from *B. fuckeliana* (*BCIG_12343.t1*), *S. sclerotiorum* (*SSIG_09060*), *T. reesei* (*tree_109276*), *M. grisea* (*MGG_07358.t1* and *MGG_00282.t1*), and *M. poae* (*MAPG_03852T0*). Clade sf5 (BS = 100% in both NJ and ML) was composed of the two duplicated genes from the nematophagous fungus *Arthobotrys oligospora* (*AOL_s0054g992* and *AOL_ s00215g551*). In NJ analyses, the gene from *P. tritici-repentis* (*Ptri_Pt-1C-BFP:PTRG_07828.t1*) clustered together with the genes in clade sf5, while it showed close relationships with the genes in clade sf7 in ML analyses. Clade sf6 (BS = 55% in NJ and BS = 73% in ML) was a lineage-specific clade which consisted of the genes identified from Sordariomycetes fungi. The duplicated genes within this clade are almost all from phytopathogenic fungi except one gene from the saprophytic fungus *T. reesei* (*tree_60791*) and one gene from *E. festucae*(*ACN30268*). Clade sf7 (BS = 52% in NJ and BS = 56% in ML) is composed of the genes from Pezizomycotina fungi. The three duplicated genes from the Dothideomycetes fungus *P. tritici-repentis* within this clade did not clustered together and the two duplicated genes from the nematophagous fungi *Arthrobotrys oligospora* are clustered at the base of this clade.

### Kexin subfamily

**Figure 4:**
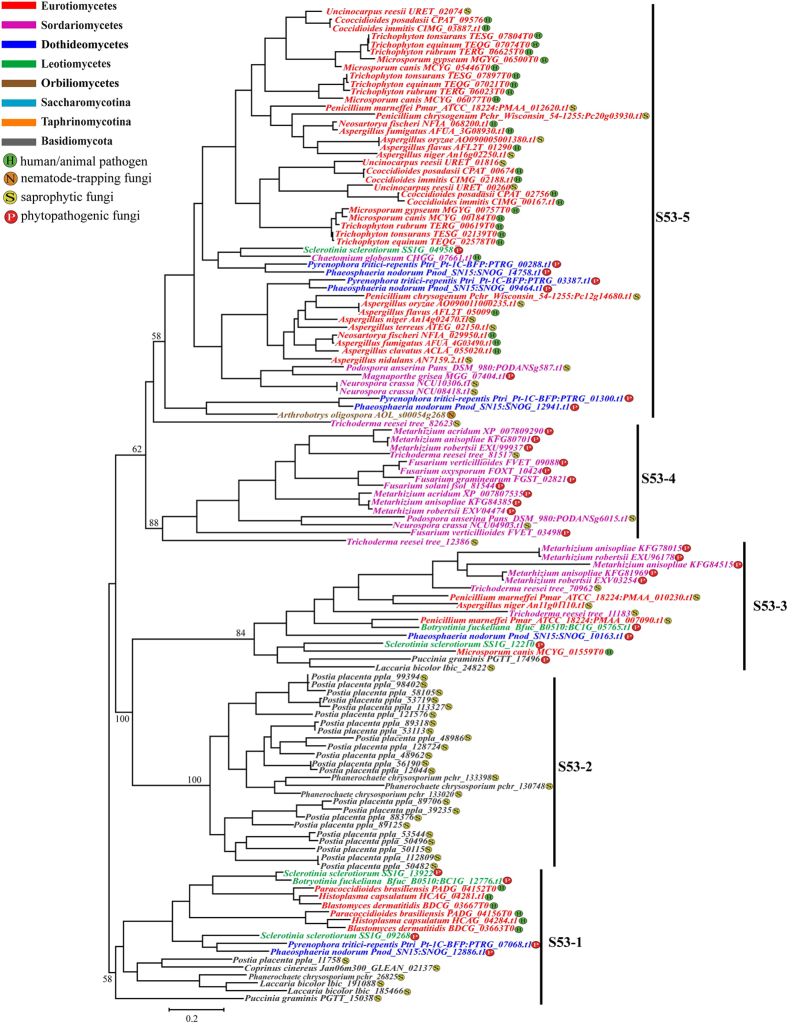
Phylogenetic relationships of the kexin subfamily. Phylogenetic analyses were performed using maximum likelihood (ML) methods as implemented in PHYML 3.0 with TrN+I+G model based on an alignment of 465-bp amino acids from 84 kexin sequences.

Based on an alignment of 465 amino acids from 84 kexin sequences (Supplementary data S9), the observed tree topology of kexin subfamily subtilase genes is largely consistent with the taxonomical relationships of Mucormycota, Basidiomycota and Ascomycota at the main nodes (Fig. 4 and Supplementary Fig. S10). However, several exceptions were noted. In the first, the kexin genes from Taphrinomycotina fungi showed a close relationship with those from Basidiomycota fungi but not with Saccharomycotina fungi. In the second, the genes from the two Leotiomycetes fungi were clustered together with those of Sordariomycetes fungi. Finally, one kexin gene of the Sordariomycetes fungus *E. festucae* was clustered into the clade of Eurotiomycetes fungi.

### S53 family

**Figure 5:**
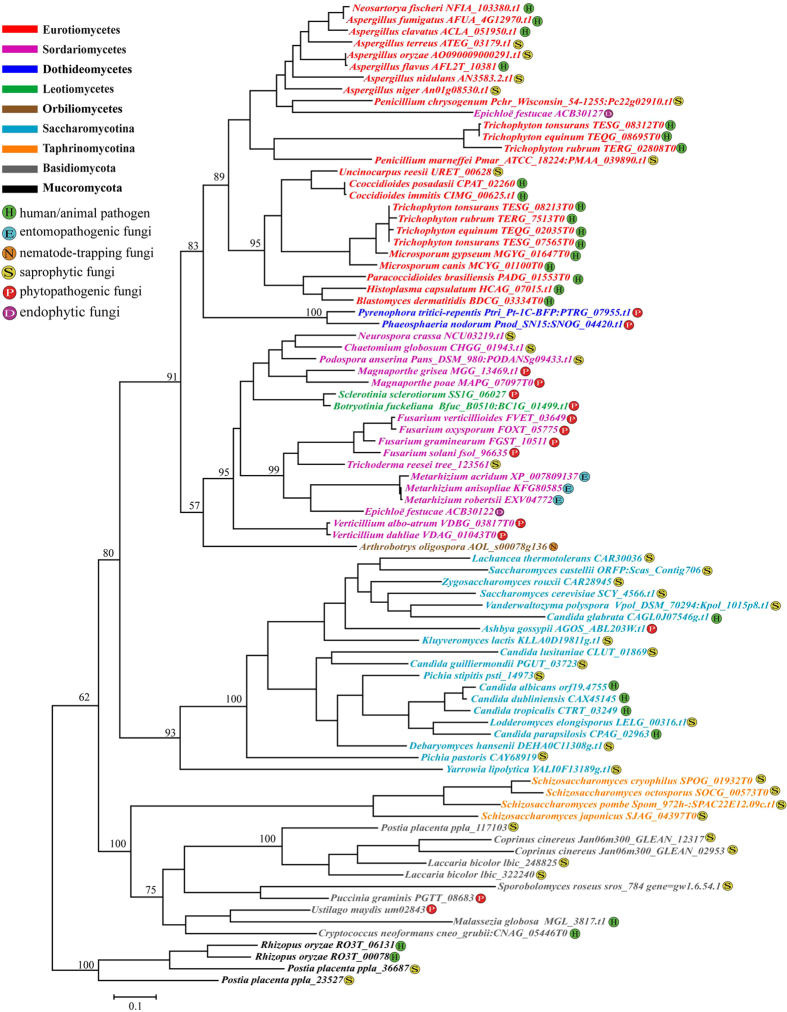
Phylogenetic relationships of the S53 family. Phylogenetic analyses were performed using maximum likelihood (ML) methods as implemented in PHYML 3.0 with TrN+I+G model based on an alignment of 363 amino acids from 126 S53 family sequences. Five clades were designated (S53-1 to S53-5) and the bootstrap support value for each clade is shown.

Phylogenetic analyses based on an alignment of 363 amino acids from 126 S53 family sequences (Supplementary data S11) revealed that the S53 family genes in fungi seemed to have evolved by a series of duplication events after the split of the main lineages. These sequences were mainly grouped into five clusters designated S53-1 to S53-5 (Fig. 5 and Supplementary Fig. S12). As seen in Fig. 5, clade S53-1 (BS = 70% in NJ and BS = 58% in ML) was mainly composed of the genes from the Basidiomycota fungi and showed early divergence. However, it also included the duplicated genes from the three Onygenales fungi (*Blastomyces dermatitidis, Histoplasma capsulatum*, and *Paracoccidioides brasiliensis*). It seemed that the duplications occurred before the divergence of these three fungi. Moreover, duplications must have occurred in the Leotiomycetes lineage, except that *B. fuckeliana* retained only one of the duplications. Clade S53-2 (BS = 100% in both NJ and ML) was a lineage-specific clade which contained 22 duplicated genes from the Basidiomycota fungus *P. blakesleeanu* and 3 duplicated genes from the Basidiomycota fungus *P. chrysosporium.* Clade S53-3 (BS = 100% in NJ and BS = 84% in ML) was comprised of genes from several Ascomycota fungi and two Basidiomycota fungi. S53-4 (BS = 98% in NJ and BS = 88% in ML) was a lineage-specific clade which consisted of the duplicated genes from Sordariomycetes fungi. Moreover, the genes from *Fusarium* spp. and *Metarhizium* spp. were duplicated after the split of *Fusarium* and *Metarhizium* but before the speciation within each genus. S53-4 (BS = 64% in NJ and BS = 58% in ML) was a large clade which was composed of the duplicated genes from the main lineages of Pezizomycotina fungi. Within this clade, the genes from the two Dothideomycetes fungi duplicated at least three times and the genes from the *Aspergillus* spp., *Trichophyton* spp. and Onygenales fungi duplicated before the deep radiation of each genus/family.

## Discussions

Previously, several studies revealed substantial variations in the numbers of subtilase genes in different fungi^1^,^3^,^4^,^15^. In this study, we conducted a large-scale phylogenomic survey of subtilase protease genes from 83 fungal genomes in order to identify the evolutionary patterns and subsequent functional divergences of different subtilase subfamilies/families in the fungal kingdom. Besides the major clusterssuch as proteinase K-like and Pyrolisin subfamilies, and S53 family, Muszewska *et al*. recently found six new groups of subtilase genes (new 1 to new 6). However, only four new groups were identified in our study (new 1, new 3 and new 4 groups are the same as the classification of Muszewska *et al*., and new 2 group in our study corresponds to the new 6 group proposed by Muszewska *et al*.^15^) and the new 2 and new 5 groups proposed by Muszewska *et al*. were not identified in our study. We initial speculated that the inconsistency between our study and that of Muszewska *et al*. may be due to the deletion of the ambiguous alignment around the three active catalytic residues. We tried to find those missing groups from our initial alignment without any deletion, however, no new groups were discovered. Thus we speculated that the missing groups were likely artifacts in previous studies and were removed in our newer assemblies.

Firstly, during the evolution of the main fungal lineages, both gene duplication and loss events have occurred in subtilase genes, leading to the diversification of subtilase genes in different fungi. For example, 19, 19 and 15 duplicated proteinase K-like subfamily genes were characterized in *M. anisopliae, M. robertsii*, and *M. acridum* in our study, respectively, including the 10 subtilases which have been identified previously based on the expressed sequence tag (EST) analyses during growth on insect cuticle (*Pr1A, Pr1B, Pr1D* to *Pr1K*)^37^. The smaller numbers in *M. acridum* suggested that several proteinase K-like genes have been lost in this fungus. In the case of *M. anisopliae*, our phylogenetic analyses revealed that these proteinase K-like subfamily genes clustered into at least five clusters: the vacuolar proteases *Pr1H* clustered with other vacuolar proteases from Sordariomycetes fungi into SF1, while other duplicated genes clustered into SF2 and SF5, respectively. Considering that gene duplication and subsequent functional divergence is an important mechanism for generating the functional diversification necessary for adaptation, the presence of multiple subtilases in one pathogenic fungus may reflect their expanded ecological roles in pathogenesis. Specifically, the duplication and diversification events have likely contributed to increased adaptability, host range, and/or survived in various ecological habitats outside the hosts^38^,^39^. During such events, the duplicated genes within a species/genus that were clustered into one group might have similar functions, whereas novel functions might have formed among the duplicated genes which clustered into separate clades. Previous researches suggested that *Pr1A* was the key virulence factor involved in the degradation of insect cuticles, while other *Pr1’*s are minor components for cuticle degradation^37^. However, from our phylogenetic results, the functionally confirmed insect cuticle-degrading *PR1A* (accession GenBank number of KFG86683) was grouped into SF3 with the other 7 paralogous genes (*Pr1B, Pr1G, Pr1I, Pr1K* and other three genes with accession GenBank numbers: KFG79696, KFG79277, and KFG81372). It is likely that those subtilase proteases clustered within SF3 may serve a similar function during the degradation of insect cuticles. Moreover, SF5 included the genes *Pr1D, Pr1E, Pr1F, Pr1J* and other three genes with GenBank numbers of KFG79137, KFG81922, and KFG85392. Although not all the functions of the genes have been characterized, the duplicated subtilase genes in the entomopathogenic fungi *Metarhizium* spp. likely play different roles in pathogenesis such as increasing adaptability and host range, or have different functions in survival in various ecological habitats outside the host. Our analyses offer a promising basis for further understanding the gene duplication of subtilase genes in fungi. It should be noted that gene duplication and loss events of proteins are common in fungi. Many functional proteinases such as chitinases or Zincin metalloproteinases also experienced complicated gene duplications and losses during fungal evolution. For instance, Karlsson and Stenlid identified the glycoside hydrolase 18 (GH18) family of chitinases in fungi and found that many duplication and loss events have occurred for this group of genes in fungi^40^. Also, the M35 family (deuterolysin) and M36 family (fungalysin) genes of Zincin metalloproteinases showed large variations in gene numbers among Ascomycota fungi. Strikingly different gene duplication and loss events of metalloproteinases have been observed in Onygenales fungi and the duplicated genes have likely diverged functionally to play important roles during the evolution of pathogenicity of dermatophytic and *Coccidioides* fungi^41^. All these studies suggested that gene duplication and loss play an important role in the evolution of novel functions and for shaping an organism’s gene content.

Secondly, subtilase genes belonging to different families have distinct evolutionary histories, with the proteinase K-like subfamily displaying the most complicated evolutionary history while the kexin subfamily shows the most steady evolution. For proteinase K-like subfamily genes, the vacuolar proteases of Sordariomycetes fungi formed SF1 and showed close relationships with the outgroup genes (Fig. 2), suggesting the primitive character of the vacuolar genes in Sordariomycetes fungi. Vacuolar proteases play an essential role in the breakdown of autophagic bodies in the vacuole during autophagy, allowing recycling of macromolecules during nutrient starvation^42^,^43^. Thus, the genes within SF1 are likely the main protease enzymes involved in autophagy in fungi. This result is consistent with our previous studies which hypothesized that the proteinase K-like serine proteases of Pezizomycotina fungi most likely evolved from endocellular to extracellular proteases^4^. Subsequently, extensive duplications and functional divergences of proteinase K-like genes have occurred especially in Sordariomycetes pathogenic fungi (Table 1 and Fig. 2), which may have facilitated the adaptation of these fungi to a broad array of ecological niches.

The pyrolisin subfamily genes also showed variable numbers among the fungal species. In our analyses, 8 and 9 Pyrolisin genes were identified in the two Mucorales fungi *R. oryzae* and *P. blakesleeanus*, while only 1 to 2 Pyrolisins were observed in the Basidiomycota fungi, and no pyrolisin was detected in the Basidiomycota fungi *M. globosa, Sporobolomyces roseus,* and *P. placenta*, suggesting that Pyrolisins have contracted or lost in Basidiomycota fungi. However, the phytopathogenic Basidiomycota fungus *P. graminis* possessed 8 Pyrolisin genes, suggesting that gene duplication of Pyrolisin occurred in this fungus. Interestingly, no pyrolisin gene was identified in any yeast-like fungus (Saccharomycotina and Taphrinomycotina) but pyrolisin genes were extensively found in Basidiomycota fungi, suggesting that the gene loss may have taken place in the common ancestor of the Saccharomycotina and Taphrinomycotina lineages. All of the Eurotiomycetes fungi except for *A. terreus* did not possess pyrolisin genes, hence the pyrolisin gene may also have been lost at an early stage in the common ancestor of Eurotiomycetes fungi. Although gene loss events have frequently occurred in Basidiomycota, Saccharomycotina, Taphrinomycotina and Eurotiomycetes fungi, it seems that duplication of Pyrolisin genes has commonly occurred in Sordariomycetes fungi, especially in the phytopathogenic and entomopathogenic fungi, such as *Magnaporthe* spp. *Metarhizium* spp., and *Verticillium* spp. In *Metarhizium* spp., a Pyrolisin, *Pr1C*, was identified previously based on expressed sequence tag (EST) analyses during growth on insect cuticle^37^. In our study, 9, 11 and 7 duplicated Pyrolisin sequences were identified in *M. anisopliae, M. robertsii*, and *M. acridum*, respectively. Phylogenetic analyses revealed that these duplicated Pyrolisin genes clustered into two clades: one duplicated Pyrolisin gene is clustered into sf6 while *Pr1C* clustered into sf7 with seven other duplicated Pyrolisin genes (Fig. 3). Thus, although pyrolisin has not been extensively studied in fungi, it may play an important role in interactions with the environment, especially in pathogenic fungi. Previously, phylogenetic analysis based on a relatively small dataset of sequenced fungal genomes grouped the Pyrolisin subfamily genes into 3 distinct clusters in fungi: one was Basidiomycota and two were subfamilies of Ascomycota (abbreviated as sf1 and sf2)^3^. However, our phylogenetic analysis of the Pyrolisin subfamily identified three additional clades: the Mucorales fungi *R. oryzae* and *P. blakesleeanus* were clustered together and the Ascomycota fungi formed at least 4 clades.

There is little evidence for gene duplication and loss in the kexin subfamily, which may be related to the functional conservation of kexins in fungi (Fig. 4). The kexins displayed the lowest variations in gene numbers among the analyzed fungal taxa and most fungi contained at least one kexin except for four fungi: *P. chrysosporium, S. kluyveri, S. kudriavzevii* and *S. mikatae*. However, considering that kexins play essential roles in fungi, the absence of kexin proteins in these four genomes may be related to the low quality of their genome sequences. However, we still can not exclude the possibility that these fungi may have lost their kexin during evolution. The identification of kexin-like proteins in Microsporidia *E. bieneusi* and *E. intestinalis* suggested that kexin proteins might have originated from a common eukaryotic ancestor, but was discarded by Microsporidia during evolution. Moreover, the evolutionary history of kexin is similar to the phylogeny of fungi because the observed tree topology of the kexin subfamily shows a general agreement with taxonomic relationships at the main nodes as revealed by other molecular markers. In general, kexins are thought to play an important role in post-translational modification in eukaryotes^14^,^44^. Secreted proteins in eukaryotes are often synthesized as preproproteins, which undergo two proteolytic processing events to become mature proteins. The prepeptide is normally removed by a signal peptidase in the endoplasmic reticulum. The resulting pro-protein is then transferred to the Golgi, where kexin cleaves the propeptide to produce the mature protein^45^. Therefore, the functional conservation of kexins is consistent with the low frequency of duplication and loss of kexin in fungi.

S53 family genes were retained by only a few Basidiomycota fungi. However, the fungus *P. placenta* contained the largest number (23) of S53 family genes, 22 of which formed a lineage-specific clade with the three duplicated genes of its relative *P. chrysosporium*. The unusual number of S53 genes in *P. placenta* implied some particular adaptive significance for this fungus. Intriguingly, S53 genes have been totally discarded in yeast-like fungi but commonly retained in Pezizomycotina fungi. Phylogenetic analyses of S53 family genes revealed that clade S53-2 contained the orthologous S53 genes from most fungal species and showed close relationships to the genes from *P. blakesleeanus*, suggesting that the genes within this clade may be the ancestral orthologous genes of this family (Fig. 5). Subsequently, duplicated S53 family genes formed other clades. The duplicated genes from Eurotiomycetes fungi mainly clustered in S53-8, while the genes from Sordariomycetes fungi clustered as clade S53-4 (Fig. 5). The existence of lineage-specific clades suggested that duplications occurred before the radiation of the fungi within the same class. Although the functions of S53 family genes are still unknown, their common presence and frequent duplications in fungi, especially in filamentous fungi, imply their functional importance in fungi.

Thirdly, although subtilase genes have been considered as key virulence factors for some pathogens to infect hosts, our study revealed no association between gene expansions and pathogenicity in some fungi. For example, several opportunistic fungal pathogens such as *A. fumigatus* and *A. clavatus* share similar and lower subtilase gene copy numbers than those of other saprophytic *Aspergillus* fungi. The yeast-like human/animal pathogenic fungi *Candida* spp. also share similar subtilase gene numbers with other saprophytic Saccharomycotina fungi. The two phytopathogenic fungi (*S. sclerotiorum* and *B. fuckeliana*) belonging to Leotiomycetes also contain low copy numbers of subtilase genes. In comparison, several saprophytic fungi such as *Uncinocarpus reesei, T. reesei* and *Podospora anserina* showed extreme subtilase gene family expansions, suggesting that gene expansion is not associated with pathogenicity in some filamentous fungi. However, it may be difficult to define the exact functions of the expanded subtilase genes in a species because the deletion of one pathogenic factor may not significantly weaken the pathogenicity of the pathogen. Moreover, for pathogenic fungi to infect hosts, the collaboration of several pathogenicity factors are often needed which may also include multiple subtilase genes.

## Conclusions

The present study provides new insights into the evolution of the subtilase superfamily genes in fungi. Our comparative genomic analysis indicated that considerable duplication, loss and functional diversification of these genes have occurred in fungi. The four families of subtilase in fungi have different evolutionary patterns. These evolutionary dynamics may be related to the functional diversity of these subtilases. Our analysis provides useful information to understand the evolution and functional importance of the important subtilase superfamily in fungi.

## Additional Information

**How to cite this article**: Li, J. *et al*. Phylogenomic evolutionary surveys of subtilase superfamily genes in fungi. *Sci. Rep.* **7**, 45456; doi: 10.1038/srep45456 (2017).

**Publisher's note:** Springer Nature remains neutral with regard to jurisdictional claims in published maps and institutional affiliations.

## Supplementary Material

Supplementary Fig. S1

Supplementary Data S2

Supplementary Table S3

Supplementary Table S4

Supplementary Data S5

Supplementary Fig. S6

Supplementary Data S7

Supplementary Fig.S8

Supplementary Data S9

Supplementary Fig. S10

Supplementary Data S11

Supplementary Data S12

## Figures and Tables

**Table 1:** Numbers of subtilase superfamily genes in different fungal species.

Species	Proteinase K-like	Pyrolisin	Kexin	S53	OSP	New 1	New 2	New 3	New 4	Total
*Phaeosphaeria nodorum*^46^	5 (2)*	1 (1)	1	5	/	/	1	/	/	13 (3)
*Pyrenophora tritici-repentis*^47^	5	3	1	4 (1)	/	/	/	/	/	13 (1)
*Aspergillus clavatus*^48^	2	/	1	1	1	/	/	/	/	5
*Aspergillus flavus*^49^	2	/	1 (1)	2	1	1	/	/	/	7 (1)
*Aspergillus fumigatus*^50^	3	/	1	2	/	/	/	1	/	7
*Aspergillus fischeri*^50^	3	/	1	2	/	/	/	2	/	8
*Aspergillus nidulans*^51^	2	/	1	1	/	/	/	/	/	4
*Aspergillus niger*^52^	2	/	1	3	/	3	1	/	/	10
*Aspergillus oryzae*^53^	2	/	1	2	/	/	/	/	/	5
*Aspergillus terreus*^54^	2	1	1	1	/	1	/	/	/	6
*Penicillium chrysogenum*^55^	2	/	1	2	/	/	/	/	/	5
*Penicillium marneffei*^56^	1	/	1	3 (1)	1	1	1	/	/	8 (1)
*Coccidioides immitis*^57^	14	/	1	3	/	/	/	/	/	18
*Coccidioides posadasii*^57^	14	0 (1)	1	3	1	/	/	/	/	19 (1)
*Microsporum canis*^58^	12	/	1	4	1	/	2 (1)	/	/	20 (1)
*Paracoccidioides brasiliensis*^59^	2	/	1	2	1	/	/	2	/	8
*Blastomyces dermatitidis*^60^	2	/	1	2	1	/	/	1	/	7
*Histoplasma capsulatum*^61^	2	/	1	2	1	/	/	1	/	7
*Microsporum gypseum*^62^	11 (1)	/	1	2	1	/	/	/	/	15 (1)
*Uncinocarpus reesii*^57^	15	0 (1)	1	3	2	/	/	/	/	21 (1)
*Trichophyton rubrum*^62^	12	/	2	3	1	/	0 (1)	/	/	18 (1)
*Trichophyton tonsurans*^62^	12 (1)	/	3	3	1	/	0 (1)	/	/	19 (2)
*Trichophyton equinum*^62^	12	/	2	3	1	/	0 (1)	/	/	18 (1)
*Chaetomium globosum*^63^	4 (1)	1	1	1	/	2	1 (2)	/	/	10 (3)
*Epichloë festucae*^1^,^64^	8	4	2	/	1	/	/	/	/	15
*Fusarium graminearum*^65^,^66^	11	3	1	1	1	3 (1)	1 (1)	/	/	21 (2)
*Fusarium oxysporum*^67^	12 (2)	2 (2)	1	1	1	1 (2)	1 (2)	/	/	19 (8)
*Fusarium solani*^67^	6	8	1	1 (1)	/	4	8 (1)	/	/	28 (2)
*Fusarium verticillioides*^65^,^67^	11 (2)	1 (2)	1	2 (1)	2	1 (1)	1	/	/	19 (6)
*Magnaporthe grisea*^68^	6 (1)	16	1	1	/	/	0 (1)	/	/	24 (2)
*Magnaporthe poae*^68^	3 (1)	9 (1)	1	/	/	1 (1)	16 (3)	/	/	30 (6)
*Metarhizium acridum*^69^	15 (2)	7 (1)	1	2	1	0 (1)	2 (1)	/	/	28 (5)
*Metarhizium anisopliae*^69^	19	9 (3)	1	5	2	2 (1)	5	/	/	43 (4)
*Metarhizium robertsii*^69^	19 (1)	11 (1)	1	4	3	3 (1)	7	/	/	48 (3)
*Verticillium dahliae*^70^	6	6 (4)	1	/	/	1	/	/	/	14 (4)
*Verticillium albo-atrum*^70^	4 (2)	10 (2)	1	/	/	1	/	/	/	16 (4)
*Neurospora crassa*^71^	3	1	1	3	/	1	/	/	/	9
*Podospora anserina*^72^	6 (2)	1	1	2	/	2	1	1	/	14 (2)
*Trichoderma reesei*^73^	5	5	1	5	/	3	3	/	/	22
*Botryotinia fuckeliana*^74^	1 (1)	1	1	2	/	/	/	/	/	5 (1)
*Sclerotinia sclerotiorum*^74^	2	1	1	4	/	/	/	/	/	8
*Arthrobotrys oligospora*^75^	19 (1)	2	1	1	1	2	2	/	/	28 (1)
*Pichia pastoris*^76^,^77^	3	1	1	/	/	/	/	/	/	5
*Zygosaccharomyces rouxii*^78^,^79^	3	/	1	/	/	/	/	/	/	4
*Lachancea thermotolerans*^80^	3	/	1	/	/	/	/	/	/	4
*Ashbya gossypii*^81^	2	/	1	/	/	/	/	/	/	3
*Candida albicans*^82^	4	/	1	/	/	/	/	/	/	5
*Candida dubliniensis*^83^	4	/	1	/	/	/	/	/	/	5
*Candida glabrata*^84^	4	/	1	/	/	/	/	/	/	5
*Candida lusitaniae*^82^	3	/	1	/	/	/	/	/	/	4
*Candida parapsilosis*^85^	4	/	1	/	/	/	/	/	/	5
*Candida tropicalis*^82^	4	/	1	/	/	/	/	/	/	5
*Candida guilliermondii*^82^	3	/	1	/	/	/	/	/	/	4
*Debaryomyces hansenii*^84^	3	/	1	/	/	/	/	/	/	4
*Kluyveromyces lactis*^84^	2	/	1	/	/	/	/	/	/	3
*Lodderomyces elongisporus*^82^	3	/	1	/	/	/	/	/	/	4
*Pichia stipitis*^86^	3	/	1	/	/	/	/	/	/	4
*Saccharomyces cerevisiae*^87^	3	/	1	/	/	/	/	/	/	4
*Saccharomyces kluyveri*^79^,^88^	1	/	/	/	/	/	/	/	/	1
*Saccharomyces kudriavzevii*^88^	2	/	/	/	/	/	/	/	/	2
*Saccharomyces mikatae*^88^	3 (1)	/	/	/	/	/	/	/	/	3 (1)
*Saccharomyces castellii*^88^	3 (3)	/	1	/	/	/	/	/	/	4 (3)
*Vanderwaltozyma polyspora*^89^	5	/	1	/	/	/	/	/	/	6
*Yarrowia lipolytica*^90^,^91^	16	/	1	/	/	/	/	/	/	17
*Schizosaccharomyces japonicus*^92^	2	/	1	/	/	/	/	/	1	4
*Schizosaccharomyces pombe*^93^	2	/	1	/	/	/	/	/	1	4
*Schizosaccharomyces cryophilus*^92^	2	/	1	/	/	/	/	/	1	4
*Schizosaccharomyces octosporus*^66^	2	/	1	/	/	/	/	/	1	4
*Cryptococcus neoformans*^94^	1	1	1	/	/	/	/	/	/	3
*Laccaria bicolor*^95^	2	1	2	3	/	/	/	/	/	8
*Phanerochaete chrysosporium*^96^	1	2	/	4 (2)	/	/	/	/	/	7 (2)
*Postia placenta*^97^	2	/	1	23	/	/	/	/	/	26
*Puccinia graminis*^98^	3	8 (2)	1	2	/	/	/	/	/	14 (2)
*Malassezia globosa*^99^	1	/	1	/	/	/	/	/	1	3
*Ustilago maydis*^100^	1	1	1	/	/	/	/	/	1	4
*Coprinus cinereus*^101^	6	2	2 (1)	1	/	/	/	/	/	11 (1)
*Sporobolomyces roseus*	2	/	1	/	/	/	/	/	/	3
*Rhizopus oryzae*^102^	10 (1)	8 (1)	2 (1)	/	/	/	/	/	1	21 (3)
*Phycomyces blakesleeanus*^103^	10 (1)	9 (3)	2	/	/	/	/	/	1	22 (4)
*Batrachochytrium dendrobatidis*^104^	1 (1)	/	0 (2)	/	/	/	/	/	1 (1)	2 (4)
*Encephalitozoon cuniculi*^105^	1	/	/	/	/	/	/	/	/	1
*Enterocytozoon bieneusi*^106^,^107^	/	/	0 (1)	/	/	/	/	/	/	0 (1)
*Encephalitozoon intestinalis*^108^	/	/	0 (1)	/	/	/	/	/	/	0 (1)

^*^Numbers in brackets represent the deleted gene numbers which show ambiguously aligned regions around the three active catalytic residues.
