# Supplementary material for: Phylogenomic evolutionary surveys of subtilase superfamily genes in fungi
Source: Sci Rep. 2017 Mar 30;7:45456. doi: 10.1038/srep45456 (PMC5371821; doi:10.1038/srep45456)
Supplement: Supplementary Fig. S1 [file srep45456-s1.docx]

**Phylogenomic evolutionary surveys of subtilase superfamily genes in fungi**

Juan Li*, Fei Gu, Runian Wu, JinKui Yang and Ke-Qin Zhang*

*State Key Laboratory for Conservation and Utilization of Bio-Resources in Yunnan*, *Yunnan University*, *Kunming*, *650091*, *P.R. China.*

* Corresponding author: Juan Li and Ke-Qin Zhang

Tel: 86-871-65033805; Fax: +86-871-65034838.

E-mail address: [juanli@ynu.edu.cn](mailto:juanli@ynu.edu.cn) (Juan Li); kqzhang@ynu.edu.cn(Ke-Qin Zhang)

**Supplementary Fig. S1:Domain architectures of each clades identified by CLANS.**

The conserved motifs were predicted with the MEME tool. Additional domains were detected with InterProScan. *, no conserved motif was identified from the sequences of new2 and new4 groups because there are only 8 and 9 sequences were classified in these two groups, respectively.

**
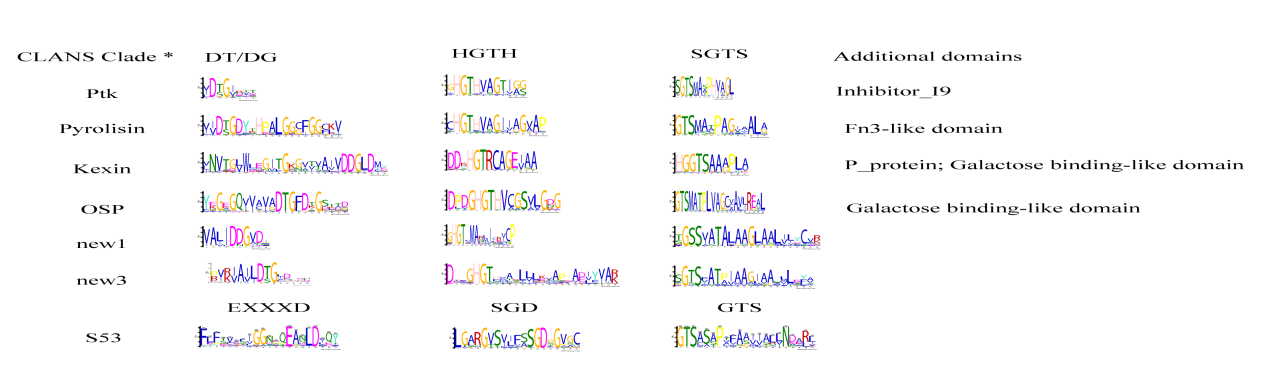
**
