# Supplementary material for: Phylogenomic evolutionary surveys of subtilase superfamily genes in fungi
Source: Sci Rep. 2017 Mar 30;7:45456. doi: 10.1038/srep45456 (PMC5371821; doi:10.1038/srep45456)
Supplement: Supplementary Data S2 [file srep45456-s2.docx]

**Phylogenomic evolutionary surveys of subtilase superfamily genes in fungi**

Juan Li*, Fei Gu, Runian Wu, JinKui Yang and Ke-Qin Zhang*

*State Key Laboratory for Conservation and Utilization of Bio-Resources in Yunnan*, *Yunnan University*, *Kunming*, *650091*, *P.R. China.*

* Corresponding author: Juan Li and Ke-Qin Zhang

Tel: 86-871-65033805; Fax: +86-871-65034838.

E-mail address: [juanli@ynu.edu.cn](mailto:juanli@ynu.edu.cn) (Juan Li); kqzhang@ynu.edu.cn(Ke-Qin Zhang)

**Supplementary data S2: 904 putative functional subtilase genes identified in this study**

　>ACB30119

YIFFKAYPVHVMTVDKLRGITGKRVKIALVDTGDFGHPALGGCFGGCLVSFGTDLVPDPKDCHGTHVAGIIAGTAPDATLGIYRVFGCEILIAFNMAYQGGANIITASVGAEAVSRIVVPCVLSAGNGDQGFYASAAADGHRVAAIAAFDNDGCVLIRGASSTTSWGPTMDLKPQFGAVGGILSTFPGTSMACPAGIMALSNPAPVPQQGAGLIQAYDATVLSNDTLKNDKKRATYRVTHVPTTLRGGLALWSGYIPYQGGRLPPGKYQFVVRALRIFGDW

>ACB30121

FIFFSGWPVHVMTIDKLRGYTGKGVHVAVIDTGDYGHPGLGACYGGCLVTNGYDLVPDPMDCHGTHVAGIVAGGSPGVTLGAYRVFGCDVLIAMNRAYIDGANIITMSIGAVAASRIVVIVTISAGNGKQGFYASSGSSGENVAAIAAYQNDLCVLVRGASNFTSWGPTLDVKPQFGAPGGILSTFPGTSMACPASIYALSNPAPVPQQGGGMVQVYDATLLENETLTNGKGDVTYEISHVPATLKKGLPVWSGYVPYQGGKLPAGTYKIVVKALRLYGDW

>ACB30122.1|_kexinlike_protease

RDYDYTPGSRVGEGPLGSLSDHHVFRKRIKDPLYKRQWHLHNTVQLGHDVNVTGVWLEGITGKKPTVAIIDDGLDMNSLDLKDNYFAEGSYDFNDGDAIPAPELSDDRHGTRCAGEVAAVNDVCGLGVAYESKIAGIRILSKPISDADEAEAMMYKYDKNQIYSCSWGPRDDGRTMEAPGVLIRRAMLKSIQEGRDGLGSIYVFASGNGAASDDNCNFDGYTNSIFSITVGAVDRAGQHPYYSEHCSAQLVVTYSSGSGIHTTDVCASGHGGTSAAAPLAAGIFALVLEVRPDLGWRDMQYLAMDTAKLHAGWQQTAIGFSHVFGYGKIDSYDLVQKAKWKKVKPQAWFFSVSFDVTEDMLSKANERLEHVTVTMNVNHTRRGDISVDLVSPANVSNIATARKDDNKNVGYVDWTFMTVAHWGEKGVGKWTLVVRDDWHLKLWGEARDASKARRRLRYEFLYDAF

>ACB30127.1|_kexinlike_protease

RSFDIAPARELASGPVGELPDHHVFSRRISDPRFESQWHLMNTIQPGNDLNVSGVWLEGVFGEGVTTAIVDDGLDFHNLDLSPNYYAGGSYDFNDDVPEPLPRLQDDHHGTLCAAEIAAANEICGVGVAYRSRVSGIRMLSGTVDDVDQAAAMNFDYQNNDIYSCSWGPKDDGRHMKAPGVLVQRAIVNGVQRGRGGKGSIYVFSAGNGASQDDNCNFDGYTNSIYSITVGAIDRTGRHALYSESCSAQLVVAWSSGSGIYTTDNCTALHSGTSAAAPLAAGVIALALSVRPDLTWRDVQHLLVEAAVPDGSWQTTKTGYSHDWGYGKIDAYALVQAARWKLVKPQAWLHAGSYTVTSEALGGANARLEHVTVTINVRHARRGDLSVELVSPSGVSYLSTPRLPDDAETGYVDWEFMSVAHWGETGEGTWRIIVKDNWRLNLWGEAIDM

>ACLA_011360.t1_gene_ACLA_011360_DS027049:join(770481..770849770918..771856771917..772096)

MLWFLTAILSTALAAQFVVQLQDKTALNAVLNSYALSSDQQGPEITDIFELGSFRAFTGDFSGPILQKLYNDPNVLAISRDGSLKLQEIVLQNNAPSHLVGLSSISPLSHQPFIYRSNGGNGVDVYLLDTGIDIKHPNLSKLNILRMADLTQSPVPQGTDPQGHGTAMAGIIASETFGVLKKCNLVDVRVADSNGDVKLSTILQALTLTQRHIEGTKRPSVVVIPLEVEDGNNPILTEAIASFDQSVPIIIPAGNNAQDALNFSPANVNPKPKNIIVVGSVDVNNNPTKFTNYGSNVDVFTVGEQITTLQSTDLDSQGLSNSLTRQVSGTSASSAITAGVIGYYMSLGLNSTQSINKVLQYSRCANTFAKHSTNTNECARLLQLQP

>ACLA_050840.t1_gene_ACLA_050840_DS027054:complement(join(1752283..17526361752691..17527791752837..17532811753339..1753662))

MRLFMAALFGLRAAGAVRFVAELGDTASVEEVLGEQYAATVAGGGVGAITLGNSFRAIYGEFPQQLLEDFYRSGQIVAMSMDRPLGVAEYMVQQHAPNHLARLSQKSSLRGGNGSDNGAYIYHSNAGNGVDVYLLDTGIDGTHPAFEGRVQKAADFSSDPVATGDPHGHGTAVAGVIGSSVFGVAKKCNLFDVRVANSTGHASLIGVLRALEHASKLAAVTKRPSLITIPLEMPRNTILNSAVEAVVRDLSIPVVVAAGNENRSACSVSPAGAYGALTIGSIDVARNDALAPFTNFAECVDLFTAGVDVATLGLDHSSEHHLSGTSISAGVASGLVAYYMSIGHYGMDAVNKIKLLSLPNVIPNLQQRSPETRNAILQNL

>ACLA_051950.t1

RSYEFSPAQLLGEGQIGELANHHTFSKRIADPIFGDQWHLFNAVQLGHDLNVTGVWMEGITGKGVTTAVVDDGLDMYSNDLKPNYFAEGSYDFNDHTPEPRPRLTDDKHGTRCAGEIAAANDVCGVGVAYDSRIAGVRILSKAIDDADEAKAINFAYQENDIFSCSWGPPDDGATMEAPGVLIKRALVNGVQNGRGGKGSIFVFAAGNGASFDDNCNFDGYTNSIYSITVGAIDREGKHPSYSESCSAQLVVAYSSGSSIHTTDVCYSFHGGTSAAGPLAAGTVALALSARPELTWRDAQYLLVETSVPDGSWQVTKSGFSHDWGYGKVDAYSLVQKAKWELVKPQAWYHSSSHEVTEEMMKTANARLEHVTVTMNVNHTRRGDLSVELRSPDGVSHLSTTRRSDNQKAGYVDWTFMTVAHWGESGIGKWTVIVKDDWRLNLWGEAIDGTNQRKRIRYDFLYNAF

>ACLA_055020.t1

VFEPQGWLQIALQQDVDGFEKALSDPSHASYGKHFMKRMLLPSQESVQSVRSWLDWINFVGVANTLLDADFKWYRTLSYSIPESLASHVHMIQPTTRFTPQCLKSLYSKVAFASFLEEYARYDDLAKFEFSVIQYGGNDQEANLDLQYMLGISAPTEFSTGGRNEPLEFLQNVLKMPQHKLPQVISTSYGEDEQIPYALSVCNLYAQLGSRGVSVIFSSGDSGVGCQTNDGFPPQFPAACPWVTSVGGEEASSGGFSDLWPRPGYQAVRGYLLYNPRGRAFPDVAAQAYAVFDKGYAGTSCSAPAFGGIIGLLNDARLRAHKPPMGFLNPWLYFNDIVVGGCSWNATEGWDPATGLGTPFGKL

>ACLA_060050.t1_gene_ACLA_060050_DS026990:852974..855118

MSSITINGNTLEISPDARHVSARNAESSNYILLRSKDRLKMAQMEELEALGVKAHTIVDTDTYLCEYAPSDLSSLRSKDYLDYVDIYHPSLKVSAHLRQPEAFSSFAARSDDEPVQDELEVVICLHQNGPGSQAVNDELVRRGLIDPQKTKVWESRIQTAVKQAKVDQIAQVESVRAIEPFASQRPCNNIARVLLNHPVCREPNDLGLPGHAYQGAGQVIHVADTGFDTGVAPCQHAHFQDRLLAVYQVNQTEPVPDKPEDTDGHGTHVCGSAVGHGRSTTMGGPEAKGDGQIQGTAPAASFVASRMTDSGSGDFFSGDFKLIVDVPYTQHQARISNHSYGAWKDVPQVGYNESNRDIDQYLFDHPDLLVCFAAGNRGQHPNVGPAQVAGDAISKNVLTVANSYTCRRLDADGKFKGSPNAISENYGINPSSSRGFALDDQRWKPDLCAPGTAILSSVSSRWDPTKPRDKFGDSTDSMYTFNTGTSMATPLVAGCAAVLREALVEQGVAKPSAMLLKALLVNGAVDILRASVSGSESRQKQQGFGRVNMGGSLLSIHASSPTHPAYEPSQGGFVDACWAKTETSPGASPEMKAMTQLKEDESCGFTIQVPAGRTVVPLGDDFPWTLKVTLVWHDQPGPKLQTKLGLAVVHSTGKRRHGNKGDADFAATPKEFDAINNVQRIVWENIPAGECKVTVTCSQYFLKAVPYAVAWTLE*

>ACN30268

YIIFTGWPVHQWTVHELHGFKGKGTKVAIVDTGDYSHQALGGCFGGCKIAGGYDLVPDPMDYHGTHVAGIIAQDVLIAFCDAYSAGADVITASINALVASRIAVFVSIAAGNGEIGFYSGVGSNGRHVVSVAAAYFTSWGPTLILKPDIGAPGYIISTYLGSSMAAPAGIAALGRNAPPFQVGAGLVDARKVTQLSLDTLTNANRTVLYTFQHEPLRIEAGLPLYSGKIPYGGGKLAPGNYSMRIAALRPYGHW

>ACN30271

FNGWTVHVMTVDKLRGLTGDGYKIALVDTGDYTHPDLGGCFGHCIVSFGRDFVPGPKDCHGTHLAGIVAGAAPGVKLGAYRIFGCDLVVALNKAFEDGAHIIATTAEAVVASRIVVPVIASAGNGDSGFYAASPASAKGVIAVASYDSDACVLVRGASSYTSWGPTMDSKPQLGAPGGIISTWAGTSAASPAAIIALAKPAPAAQQGGGIVQAYDASLLYNDTISNGKKSVTYKICHVPATLAPGLPLWSGYIPYQGGKLPAGKYKFVTRALKISGDW

>AFL2T_01290

VVEPDGWLQLAVPEKITEFEQRVSTPGNQNYGRHMVRNFLYSSDVAPRKVLSWLGWITFVSQAEQLLRTRFYTFRTLKYSVPKELRSFVQMIQPTTRFTPSCLRKLYNRLGVSGFLDQYARYSDFHQFLYTVELIGGNLQEASLDIQYAAALADTTFYSTAGRHEPLEQLRYLLDLPDDELPAVLTTSYGELEQVPYARTTCNMFAQLGARGVSVIFSSGDSGVGCITNDGFQPLYPASCPFVTSVGGEMASTGGFSEYFPRPSYQSVNQYLLYNPNGRAIPDVAAQAFIIMDHGTGGTSAAAPVFAGIVSRLNAARLESNKPRLGFLNPWLYFTDIVDGGCSWNATPGWDPVTGLGTPYKAL

>AFL2T_01995_|_AFL2G_01995_|_Aspergillus_flavus_oryzin_precursor_(404_aa)

MMKSTAFTVTGLFCWSILGLATAHEYVVQLTSETSLNSFLESNSLNNLDEPVDKIVIGSDFHGFSGEFEDEVMSELYGDPRVASISIDRMLYLQEYLRQNDAPEHLARLSNGVSNSRVSAKNENAFIFHSHAGIGVDVYIVDSGIDSQHPAFMNRHISKLIDLTDNPIPVGDPHGHGTAIAGLVTSDTFGVMKKANLFDVRVVDAESNGVKLSKLLKSLEFIENHSRATMRPSVAVLPFSMSKNAILNSAIESMPNSIAIVVPAGNQHHMACNFSPASARNSQSILSVGSLDYRSSIEIASFSNYGECVDVFTSGVQMDTLHATTNSDENYISKVSGTSMSCAIAAGTVGYYMSLGLSSVEAIEKIKSAKEILVSGEAIKELKLTL*

>AFL2T_03914_|_AFL2G_03914_|_Aspergillus_flavus_predicted_protein_(699_aa)

MSVITINGNSLDPAVQQAALQAHGIYKPDASSSDYILIQTVQHPSISQKSQLRDLGVEIHEYVSENTYLCGFKAADLTPVRRLDFVKWANIYPQLFVLPPRLKRQVNPSEADAPNLVGAPHTRTLRTVDLILHEGIDVADPDNKSSIAKAAHVDFDIINARGNKIRLQVQEQYLDHLAALDIIKAIHEVHQTKLYNDQARNIMDADINLNRIQYKGLGQVIAVADTGYDQGSTNPGLTLPAFKDPPSGPSGRVKVQHLYALGRQNRTDDPDGHGTHVCGSVVGNDDYNGATIESPASRASLVVQSLLDERNGLGGIPTNLESLFLGPYQEHHARIHTNSWGYVWTGSQLPYDNSSAEIDNFVWNHPSMVICFAAGNDGIDNSPANGIIDLAQIGAHAAAKNCVTVGASESNRNNPRTYHSVWPFDYPSPPIRNDSIANNPNGMAAFSSRGPTKEGRIKPDVVAPGTSILSTRSRRCHLDPANIWGAANGDWVYLGGTSMATPLVASCAAVLRETLVNNGEHEPSAALIKALLINGAVELTGQYNPPEAGQSPNPNSGWGRVNLANSVILRPVNDKEYAEGGPLEQGDENDVFRITIANGNSELKVTLVWTDPPGPCLQNDLDLIVTANGRERHGNMGVSQGYDRVNNVEQVTWTNIPSGQAIVKLRNVCQQSEILYQGLGLRREPKDLDVRDLHLVIN*

>AFL2T_05009

AFEPKGWLKIALQKDAAGFEKTVSDPDHPSYGQHFMKRMLLPRDDTVDAVRQWLDWINFVDTANKLLNAQFKWYRTLQYDVPESVTPHINTIQPTTRFTPTCLKELYSKIAFASYLEEYARYADLENFEFSVTTFGGNDQEANLDLQYILGVSAPTEFSTGGRNEPLEFFQNVLKLDQKDLPQVISTSYGENEQIPYARTVCNLIAQLGSRGVSVLFSSGDSGVGCMTNDGFPPQFPAACPWVTSVGAERGSSGGFSDYWPRPEWQAVSSYLLYNSSGRAFPDVAAQGFAVYDKGFDGTSASAPAFSAVIALLNDARLRAGKPTLGFLNPWLYLQDITLGGCSWNATQGWDPVTGLGTPFAEL

>AFL2T_10381

RSYDFSPAQRLGEGQVGELTQHHTFSKRITDPIFGGQWHLYNTVQVGHDLNVSDVWLEGITGKGVITAVVDDGLDMYSNDLKPNYFAEGSYDFNDHVPEPRPRLGDDRHGTRCAGEIGAANDVCGVGVAYDSQVAGIRILSAPIDDADEAAAINYGFQRNDIYSCSWGPPDDGATMEAPGILIKRAMVNGIQNGRGGKGSIFVFAAGNGAGYDDNCNFDGYTNSIYSITVGAIDREGKHPSYSESCSAQLVVAYSSGSSIHTTDVCYSLHGGTSAAGPLAAGTIALALSARPELTWRDAQYLMIETAVPDGSWQTTKMGFSHDWGFGKVDAYSLVQLAKWELVKPQAWFHSSSYEITKDMMYQANEKLEHVTVTMNVNHTRRGDISVELRSPEGVSHLSTARRSDNAKAGYEDWTFMTVAHWGESGVGKWTVIVKDDWRLNLWGLSIDGFSQRKRLRYDFLYNAF

>AFL2T_10524_|_AFL2G_10524_|_Aspergillus_flavus_hypothetical_protein_(1414_aa)

MDNTLVQVTEELTTDDEDNEQTTSFGIHTEKAGEESGYHTRNDPKAPEQRRTITAYHGSVDVQGESLAIIHGELSPESELYATLLVFEFRFDGVKWKNRIPWVQISLEFRSSTLEAAGPVVHAISPQGTYSLCPVDQDEACQREGQIQAGAEQMGVSMGASYGWSKTTNRTTTNSTKIKGFKTCDVYGNSTGVSWTLEENQATKTGVPTYLRTAVCLERQQESKFEANVKVEYVIDGKSWKERFFGATDPNDPILYNPKRAPTNMLQLVYDIDDLESVQLEEIFDASIHTTLQNRNSALSALSIDNPCRRRTDVDQGESDGEGTFPSITPEISLKTVLDDVLLKLEDKNGWQTVSPETRQYLATQVDAGKSTALHLIVKHQDKKTAKKVRPLIQYLAQEHGQILCQRDNNNDTPLHIAIRDQNDRMVYRVLQAFDNLDEALAHANDSGKNCIHLAIEKKSRDLWALVESASAGTLSAKDNNGNTPLHLAVDYERLDHEDLHLIVETIVNKSDPVMRKEKNGDFNNFFINNKCEQMSPYVYHLWTRNRALKLSGDIAEKDGERARNAMRELRNIQEAKPQMNETQKRITPGVSQIQPAFQKGPGQLKKGQGSVELNEKLLEGRESQVDPEQSTTQLENSISAQGKQRNKDIESAWIQRFLKMHYLRERGHDAALEILQGRNTLANKEIYLDLSNKSNVSVEQICQQTIQTLEFEDILQYVHIPKFKPSQPPQRRSNKRLPKRSLEDIERIFQSEKLEGVKTILKIVVEDLEEPPHTDTAIERFLKPKGVETWDWKKVDICPEVIQQAAPTVRHLNLYWSGRNAVLRGWSEQEGLRRLKQLEKITLHVLPTQSRESKARIEINLSAFERRIRRLFISDRAEEVAHNEYFVDDLKIALKESISESILDEHRQRGVVGIGKAIIEATYQRITKVLIPEMSKYIAQGMVQHFEEIAPSTEESDENLVDCAAIMKMTEPFVVTFIGEMFDKLQRQIEKLQDIDSHPGRTKEAVEKKIEYIIREQILKDSLKIKELSYQLGRGLSNQLRALKVAFDHKLSEDANQETSALSISSPKKKQTQKWTECMKSFNQTLFTALENDSDIEHQIHKRGEPIVVAVIDDGVDIEQMDLKKYGSITGRSFCPQPHDPKFKIPHYNSSRGHGTLMAKQIHRICPKANLLVLKLEDRDDRETNKRCITPISAANAIRYAVQRKVHIISMSWSIRAPAVNNISDEFNELRGALQEAQNANILLFSSASDQGPDTYDTYPAFGTSAIFKIGGADVDGNLHAQVGGEDNVHYIFPGDTFEGDDNNDGLASRSQWFTGSSVATAYASGFAALILYCAQVRIALSNENDRVKCTEAFRALKTHSEMERVFRNIGKTKKYLPVWEVFGRSHKSHSGTSESDFIASVPVNLYPEPYS*

>AFL2T_10813_|_AFL2G_10813_|_Aspergillus_flavus_cerevisin_precursor_(496_aa)

MKLLSILLWISIGLCAAIPQLENDYAKLLSLMEHLEKRTMPHEKSEKSENSTIVLFSGKASISEKLAHVGSLVDKGILITKLYDISNNTAGNGVIGYVGSFTQREVQSIEDSDTIIDSVFADTLVYAQGQLEERQDIYKTVTNQPYHLARISHKQNPKGTTEAGKYSYIQYTSFPTNIYVLDSGIRTSHEAFGGRAQWGANFADDIDRDLGGHGTAVASMAAAVSVESTIWGVKVLQLNTGALSWIIAGLEWSINHATERNQKAVINMSVGSGAVDIYDRFMEIAQERNIVVTVAAGNNDQDACQKSPAKSSKGNIGVYTVGSTGDEDVQSGFSNWGDCVTLLAPGEGIKGASKDSDNGYLYWTGTSMSTPIFAGLVSYWMSISDLDYANLEYLLTRNTGLVTGLKGNTPNILAWNLHT*

>AFUA_3G08930.t1

VVEPDGWFRLAMQERAAEFERRVSTPGHSSYGQHMVREFLRPPEEVSDKVLSWLNWVTFVSQAERMLRTRFYAFRTLAYSVPHDVHRYIQMIQPTTRFTPNCLRELYNRLGVSGFLDQYARYDDFENFMFTVVSIDGNLQEASLDVQYAYSLAYKTYYTTGGRNEPLDQLHYLLDLPDEELPAVLSTSYGEDEQVPYSNATCNLFAQLGARGVSIIFSSGDSGVGCITNDGFLPVFPASCPFVTAVGGEKASSGGFSDHFPRPSYQSVQGYLLYNPSGRGFPDVAAQAFVVIDHGVGGTSASAPVFAAIVSRLNAARLEDGLLKLGFLNPWLYFTDIIDGGCSWNATPGWDPVTGLGTPYNTL

>AFUA_4G03490.t1

VFEPQGWLQIALQHDVEGFETALSDPYHPNYGKHFMKRMLLPTQEAVESVRGWLDWIKFVGVANDLLDADFKWYRTLAYSLPQSVASHVNMVQPTTRFTPQCLKDLYSKVAFASFLEEYARYDDLAKFEFSVIQYGGNDQEANLDLQYIVGVSSPTEFSTGGRNEPLEFLQNVLKMDQDKLPQVISTSYGEDEQIPYARSVCNLYAQLGSRGVSVIFSSGDSGVGCLTNDGFPPQFPAACPWVTSVGGEEASSGGFSDLWERPSWQAVKRYLLYNPKGRAFPDVAAQAYAVFDKGFDGTSCSAPAFSAIVALLNDARLRAHKPVMGFLNPWLYFNDIVKGGCSWNATDGWDPATGLGTPFGKL

>AFUA_4G11800.t1_gene_AFUA_4G11800_Afum_Af293_chr4:complement(join(3113022..31133753113448..31135363113603..31140473114105..3114428))

MKLSLLLLSTLAVALPIAQQPASEYNQLLDLINNQDLEKRDISGQPTIVMFTDNSTISDKLAHVKAIIGNDHIEKLYDVSSTEKGAGVAGYVGTFSNETLALIADAGCISIFQEDIIVKGLGLEEDLEERDSSIYTTESGQPWHLGRISHLQNPRNTPQASEYVYRTGTRDTNIYVVDSGVRTSHVAFGGRAHWGANFNNDVDEDEHGHGTAVASIAASISKNAQIYAVKVLGPQNTGSLSGILAGMEWAINHAAGQKGQSVINMSIGSDSTEVYKTLIQKALEKNIALFFAAGNNNEDACTVSPARFAKDFTGVFTVGASNLADNMEDWSGYGNCVSMVAPGVAISSASRRGDDVWTSWKGTSMASPIVAGIASYWMSIINFDLPSLEWVLTQSKGLVNTHNNTPNILAWNFHP*

>AFUA_4G12970.t1

RSYEFSPAQLLGEGQIGELANHHTFSKRITDPIFNGQWHLFNTVQLGHDLNVTGVWMEGITGKGVTTAVVDDGLDMYSNDLKPNYFPEGSYDFNDHTPEPRPRLSDDKHGTRCAGEIAAANDVCGVGVAYDSRVAGVRILSKAIDDADEATAINFAYQENDIFSCSWGPPDDGATMEGPGILIKRAFVNGVQNGRGGKGSIFVFAAGNGASFEDNCNFDGYTNSIYSITVGAIDREGNHPSYSESCSAQLVVAYSSGSGIHTTDVCYSFHGGTSAAGPLAAGTVALALSARPELTWRDAQYLMVETAVPDGSWQVTKAGFSHDWGYGKVDAYALVQKAKWELVKPQAWFHSSSYEVTEQMMKNANARLEHVTVTMNVNHTRRGDLSVELRSPEGVSHLSTTRKSDNEKAGYVDWTFMTVAHWGESGVGRWTVIVKDDWRLNLWGEAIDGANQRKRILYDFLYNAF

>AFUA_5G09210.t1_gene_AFUA_5G09210_Afum_Af293_chr5:complement(join(2365176..23653552365424..23663622366430..2366798))

MKYSIFTISTFLAVSISAAPSRSRTLKRQTQGATGKIIEQTDASWNLERISSVASIVQGSRNVTDLKYKYRYDERDVATGVDVYIHDTGIDPLNPDFNSRAQIIFTDNPYNIWPSDGHGTHVAGTIGSTHYGVAKNVSIWGIKIMSEPMEDPLGPIVEVKTALEYIQDKVNGIKAAIRQHNIHKKKKDFKGSVMNMSWGVPRDFMEMAPGGSELLMKALKDALAAGIHLTVSTTNSGADACESFPGGYVKEIPSLIVVGNTNISDTRIPTSDWGPCIDLYAPGTEIQSTSLSKKNWVEVMTGVSMAAPLVGGVVATQLAKFPELRGDTVAMKKKILSLALKNVVKDARGGGNLLLNTGISSG

>AFUA_7G04930.t1_gene_AFUA_7G04930_Afum_Af293_chr7:join(1161298..11615461161605..11621381162214..11623101162372..11624611162522..1162709)

MKSLAVISSYLLLLCTIPVTFAAPAASRTQTDLKNNEPQDDFLFILAEHEKRSLGEVIDEMGLDHTKATTFGTHMRGFSISLLESHAHDIASLENVEFMQRNHKRSGPVSNSGLKARDMTNSASFALSKRQADALVEQTTAPWGLERISQKEKIDLGDRRVDDLFFKYHFDRLSGDGVDVYSIDSGINIDHVDFNGRAKMIFTAFGDDGKDDHGHGTHTAGTIGSLTYGVAKNVNILGCKVLDASNFGSDAGIIAGIDAAFASHLKRKEEPGFKGSVMNMSLGGPAFSPAMSDLLRRALNAGMHVVVASGNENTDACSSDPGRLSTQIPIINVGAIDITDNRWVQSNFGKCVTLHAPGVGIVSTWNTGPRAVMAIDGTSMASPHVAGVVADLLAKNPDLKLDPKGMKELLLSKSQKGGVKGIEKVIPGGDFLLNTGMGDTVSDKVSNETMLLPSCVINSSHAVPAGAMDMGITASSVGSTNKGRETTSSVSTIATDASTSVDSYSSSPTATSVVLHTSPQTSDKRPRDPVGPEFFYSSKLRIQCPNPDWTLNYKIRALIADYAGPRHLMPPNAVNHQDEALGVYSPHILYVRQWATKIRDYRKHLVVDQDENVQWNPDQNDSLLQNVRNEQRDCRQCLCDAQGNITFAHRGPKKPKEGRRGQKSGCFSGSWALFCTWFYGCYCAIVLIGNQLSAEHIQQGLTWHAFQDAFNQIPDYIRDEPFNRDFRWWVPKELAERPGQTIGYNPGFRVNVASPNEPPYFLEGLGESVGEDLLRELDALLDGHQEAVPSLPFDDAGFDPAPSPEDPHSNPNYQGRGLPQEDPFPVDIEAYARQQASLWPLPNANQPLPLGQDAPSFFPMDIPADYENYLIDDGTSGGDSGGYSYEYPPGNYDFPAEFEYNSGPRGGGGGSGSGGGFFKKREAKFENSKAGDDETAHGIEGVDQGRRKVAAPT

>AFUA_7G08340.t1_gene_AFUA_7G08340_Afum_Af293_chr7:complement(join(1890381..18914621891535..18919231892025..1892347))

MFFFVPAFTSDQTDAIRGHAQVADAYIPKGQLMVRFWGVARNPTSESGPPAGDDDWFMDTLNSTESELQERSILAKRSEIVERNSPDNMVSLSWPPDIGPVPVQGDYRFDSSAGEGTYVYHVDFGAQPSHPEFSDVSFLHPLLPGPYPVSGWMENDPKRHGSLCLSKEVGKTVGIARKATVVATAWDFQKSINEHWLDALAKVHADISTGARGAKSVVNLSISIPQGDLTAAFLEKMALLIREIIKLGAVFVTGSGNSPGSPNGYPALFGDPANPNHIPELIVVGSVLGQGILGQHANADWVTCYAPGYGLRMADSDPESATEYRTTQGTSFASATVAGLAAYFRGLDSTLTTAASVKERILRLAYRRQPQPNHPEGPYQRYIDNVVWNGQKWGRSIVPECSDFSKAKRQSSGGSCPVAFPPQPSPLTFRTGPPQPTCAGAGCGSSCAGFFCPGTPLKQNPDFLDPRNPDSVQNPDSRYYEDWDGTITRTTTTPTKTIPTTTPTPPKSSSVPIGGPCRLTDECEDNCPKPGAVQCESGACTCLTPPPKTTPPHAAMCYDVQQCLNVYTCASGDTMVCEPTDYSNGNGLCQCIKGNPS*

>AGOS_AAR069W.t1_gene_AGOS_AAR069W_Egos_ATCC_10895_chrI:460781..463087

MKTLILFYTTLIITTLQSTVSTAPTSTKEKRWLHPGAKRPVVAHSTDDDPFHDYIAILSDDETRPWAEIFDEMGFSTKGSKKMTHSLGVSGSGRPSYNMTSYDVNGIKTFGKNLRAFTINMRESEAEGMDGNPSLVSLEKTKMRQWAFVPGSKKVLPSLPLVEQGVRDFSLRKRQDDVMWIQQGNAPWNLQRLSCEHKIDVKGRLATDLKYNYRFDEVAGNGVDVYVVDSGINVDHVEFQGRARMMFSYFGKVNDDVGHGTHCAGTIAALHYGVAKNVNIWGIKVGDSSGVNGEAIVAGIDAAITRHNQRKSQPGFMGSVISMSVGTAAPQQSDFMILQKAVQAGMHVSVAAMNSATDACNFSPGAFSQQIPIINVGAAGIDDTRAPFSNFGKCVDIYAPGVSIVSTFNNDATGIASLDGTSMACPAVTGIIADELVRNPKLRLDPAAMKKHILGKALKGVIKADNVINGSELMANNGFPGDPQV

>AGOS_ABL203W.t1

KDHEYADEELLEEHAVRGLERHYVLSKRIKDPLFDEQWHLLNTRYPKNDMNVTGLWQKNITGHGIVVAVVDDGLDYESEDLKDNFCAEGSWDFNSNTALPKPMLSDDTHGTRCAGEIAAANQFCGLGVAFNSKVSGIRILSEDITPEDEAASLVYGLDINDIYSCSWGPTDNGEELQAPSDLVKKAIIRGVTEGRDRKGALYVFASGNGGALGDNCNYDGYTNSIYSITVSALDHRGLHPTYAESCSAVLVVAHSSGSGIRTTDVCFDHHGGTSAAAPLAAGVYALLLQVNPNLTWRDVQYLTILTSIEQRLQEGSLGYSHKYGYGKLDAYNIVELAKWKNVNPQAWYYHSTTSVSRDALDKANKRVEHVTVTVDIEASIRGFTTVDLIAPNNISHLGVVRKKDKSHAGFRNWTFMSVAHWGYAGEGDWKLQVGWRLKLFGESIDASKARRFEFMLSD

>AGOS_ACR012C.t1_gene_AGOS_ACR012C_Egos_ATCC_10895_chrIII:complement(378750..380375)

MKWQLISTSFILISLPATFAKPTVNRGNFKVKPRWHKSEVTLTASSKDSTPPTYGTLKADDDDPFNTYIVTMKKNEKRPWMEIFDEMGFNATEKKDNIYSAHANSKSGYQNHIRDFETDFGEKIEAFGHNMRAFTMNLRESEADGLSGLEEVAIIEKDSIARPAVIEEDEYEVLNVTVGKFEKRQQQGQSHIYTQRTAPWSLQRISSRNKVMTRGRKVTDMSYYYTYDSMAGFGVDVYVLDTGTNVEHTDFAGRAKREFNAFPGDDGKDARGHGTHTAGTVGSIHYGVAKNANILAMKVINNAGVGPSSAIVQAFDHAIRRHNERRRDPNFKGSVISMSLSGKGTAESLKNIMRTATQAGIHVSIAAGNAKEDACTVWPGRYSREIPIITVGASDINDQRAGFSNFGPCVNIHAPGVAIMSTYNKGPTSTTSMQGTSMACPAVSGIIADEMVKNPRLRFNPIAMKRHLIAMSAGVAVRGANDGRGLANNGFYGAA

>An01g08530.t1

RSYDYSPAQRLGEGPVGELPSHHTFSKRIADPIFGEQWHLYNTVQLGHDLNVTGIWLEGVTGQGVTTAIVDDGLDMYSNDLRPNYFAAGSYDYNDKVPEPRPRLSDDRHGTRCAGEIGAANDVCGVGVAYDSRIAGIRILSAPIDDTDEAAAINYAYQENDIYSCSWGPYDDGATMEAPGTLIKRAMVNGIQNGRGGKGSVFVFAAGNGAIHDDNCNFDGYTNSIYSITVGAIDREGNHPPYSESCSAQLVVAYSSGASIHTTDVCSTTHGGTSAAGPLAAGTVALALSVRPELTWRDVQYLMIEAAVPDGSWQDTKNGFSHDWGYGKVDTYTLVKRAEWDLVKPQAWLHSSSYEVTEDMLKGANERLEHVTVTMNVNHTRRGDLSVELRSPDGVSHLSTPRRPDNQEVGYVDWTFMSVAHWGESGIGKWTVIVKDDWRLNLWGEAIDGAEQRKRIRYDFLYNAF

>AN0238.2.t1_gene_AN0238.2_Enid_FGSC_A4_chrVIII_scaffold_1:complement(join(738071..738843739151..739227739699..739823739874..740797740853..741293741506..741514741775..741876742095..742168742347..74235)

MKSIILSTTLFFATLQSASTFPTETPTKRWYRSGDKIAAIAHNIKTLPPATKPCLTPQSHAKDPKDDDPISEYLVAMTEDEKRPWPVIFDEMGYNATRVKETRDVHKFLAFDEQLGDSSYIRFQTEEGIRIDTFGKSIRAFTMKMSESEAEGMADAENIGSLEKNGLVYASVMPQDEEDLAVEAAYNLTSWDEEHIFEKRQNGRIRTQNTAPWNLQRVSSANRVNAQGRRATDLNYNYRFDQSGGAGVDVYVLDTGINTAHTEFGGRASMGFTGYGNNFNDDGGHGTHCSGTIGGTRFGVSKNVNLIGAKVLPGRGPGTFAAILGGLEFAYRRHLQRMKDPNFKGSVVSMSLGGNGRATGALRAMEKGIQAGMHFSVAAGNDNADACNFWPAGFSAQIPIITVGATDINDNRARFSNFGRCVDIHAPGVAIISSHNRGPQSITSMQGTSMACPAVSGMIAEELVKNPNLKLNPRGMKQLILSKAIRGVVRGTNNAPNMLNNGLR

>An02g02850.t1_gene_An02g02850_Anig_CBS_51388_An02:complement(join(663653..664375664418..665017665068..665594665646..665818665871..666796))

MIFKESLKIFREATPSETAKNPDLDLDYDDDDSTNYAVQMSPKDKVQAAFDEIISKICNKTLDLRNEGSRAAFMADYGLFLEERTRVDRQTFFHIAASRLAHSTAIRFVVRKKRHLLQYKDASDRTPLHVAIVYGNNAFVDAILKEVNGTELDLLLRETCANGQNCIHAAIYHSLNQGCALKLIEGASNTTLSASDHNGLTPLHLAVEYHRSSASQVGLVRALIARGDGALDKFTTNPPSLSVYEYHRYTREKAHMGQQGAKSGSSLELQGGQVEDQSTLVRGRRALDELPIHQNTRNLPQQMDKGDQRQEPRTDVSREIDTDSYADSIQQVMKLYYLRSTFESAPERNIRDQLSATRFLYGANIRNVNLSFDFSQGPLTISQDSFEDSYRHMVFDEVLRYVSFRPVLLQKPPGPAPGSRLAKKLAQTKKEDQGPNDLVFFFNWLYRKNVRHILKVVVHDTEDAPHTDKAMVDCLSKFEIETLSWRKVDLSSQTLFNACRNLKEVYLRWSGNLSILKTWTEQDGLLKLEELECVHLVWNKEEGPETFHKVASAVEYLQSRFNQAVAEINTARRNNNKNSSLVEPRNPIVVRKSEIGVTPNKRPKDTASSANVSIDIRKRRIQPNRWLDCVDRFADELQNVKLLPTDMPMLKENIKVGVIDDGVDIHIESLQGKVIGGESFDRTYLDGNGTSPYYISGGGHGTMMADMVCRVCPTARLYVCKLEMHPDPDGGGRQISAESAAMPNKSQAVMAAVRQKVDIISMSWTIQETEDNQSSITALQDAIRAALDAKILLFGAASDKGAVTEIEYPCFFDRRIFRIGASTADGRVYGPSGNPQNLSFIFPGHKISPRSPYLEKGLPIDSEEKSGSSISTALAAGLAALVLHCIRLGAIQAEMEARQTGRCSSTAVRLSDLKKARDFYGMRSIFRGMGLNEDNQRYIEIWKRLDGPARRLRSPSGESADMTGLEIIAGLARDLVSGIADH*

>An07g03880.t1_gene_An07g03880_Anig_CBS_51388_An07:join(821957..822325822396..823331823388..823576)

MKSLTLLTTSLLLTTTLSAPATPSQSEDEYIVLLASDNKDSWSSIFSSMGYNRTTRHVSAHNTYGVRTLGATFLTSHGINIQTFGNGENFRGFTMKMPRTHSVNVAGMEGIAIVEKNHRRGKQMPKVSLTHSVRNIKHSPPSSSSPSSYSSQGVYRRYLHRRQQQPATNPNSTTGFTTELIQQSTAPWGLQRISSQPPIPADGRNITELTYKHRFDRTAGTGVDIYIVDSGIKIDHSDFNGRAKVLFSAFNDSGDDKDGHGTHVAGTAGSLHYGVAKNSNLWGVKVLDDNGGGSDSGIAAGIDAVLAAHNKRKGEKEFAGSVINMSLGAPETSQIIFTAIKRAVEAGIHFTIAPGNDNKDSCTDFPAGFVTQLPSLITVGATDIDDNRADFSDFGKCVDIHAPGVMIVSTTNDGNIADKDGTSMATPAVAGVVADLLAQNERFKLDPMGMKKFLLERSLKGVIKLKAGDRATDGGTERVLLNTGVGGVPK

>An09g03780.t1_gene_An09g03780_Anig_CBS_51388_An09:join(856630..856953857005..857449857497..857585857641..858033)

MVTVPTTLAAPAAPALVIPSASKLNPEWYPVNKTLPIRPTGSNILNTNSTRGGKDDPSAQYIIIMKDNEQRKWGEVFEDMGFSTREIKRSFRAHGASGNHTFQSFRTFQIDDGSHIKAFGTHVRGFTLEMLESEAESMGTLENVKYIEKDQIYEAGVVRDGGYYSQYSGVDRTVKHESKHVKRQQQQQPQQPQQPQVGQFYQQSGAPWNLQRISEVNRIAPNSGSGTSLGFNYIYDGTAGTGVDVYMIDSGINQHAEFGNRARMIFSAFGENYSDDHGHGTHTAGTVGSLRYGVAKNVNLMGIKVLDSNNRGSNTGIIAGMEAALASHLQRKNAPGFAGSIISMSIGGGGSQAQFDVLRRITEAGMHVSLSAMNDNKDACTAFPGGYSRELPIFNVGATDINDNRASFSNYGACVNIHAPGVEIVSTNNQDAQGTKVMQGTSMACPAVSGVIALELVRNPRFKFDPRGMIAHIQSMAINGVVGGANGGQIMLNNGVKGR

>An11g01110.t1

VHEPSVWMRIGLQSNLDRGHDLLSHPQSSRYGKHLVHDLFAPSNEAVETVRTWIQWLQFASEVEQLLQTEYYIYTCHEYHVPETIQSHIDYITPGVKMTPDCIRAMYNELGIFEDLGDIYSQDDLNLFFPTLDSIGAAPTESDLDFQIAYPIIWPILYQTDNYLDPPDPSPGPKQCGVYTPTNVISISYGSPEALPYQRRQCHEFMKLGLQGISVVVASGDSGVACFGDADFVPDFPATCPYLTAVGGEIAPSGGFSNIYARPSYQSVETYFVYNRIGRGYPDVSAIAIIIYNQGVGGTSAAAPAFAAMLTRINEERLAKGKSTVGFVNPVLYFRDVTVGGCGFPVAGGWDPVTGLGTPFEDL

>An14g01380.t1_gene_An14g01380_Anig_CBS_51388_An14:complement(join(371636..372625372692..372806372987..373189))

MDVIEYAAPSVQHIHLHWSGNQTVLYGWASSENGMPLLCKNPTSLLSKITLHAYQLRLSSLKGIMGNREQMEKPRSDAWIEAMERFRGSLIRMHDKSMLSNTKRVKVALIDDGIDLQDFNTYRFAQCTGVSYCSSDISNGDPWWKSTNGHGTIMANMISRINPWVQLEVIKVQSSPSYVHGDGARSMSPRSAADAINAAVVRGADIISMSWTITDVGFRMSVLSDSASNVDGDKRRADENDLKLLQSAIDDAVKGDKRLLICSAADDVRLNGDNTFPYSQAPELILRMGSTGPQANRDLGSGSGGSITYYLPGNQVAEDRRPHSAKPVVYHNGSSVSTALAAGLASLIMYCCHCLHSCQAGAEYENCAKALRSHANMRKAFNSINRYFEWKDDEKIVPVWGPFGDKSSMLDKATNSEDKIKVLKELVACLCLDIK*

>An14g01530.t1_gene_An14g01530_Anig_CBS_51388_An14:complement(join(401109..401155401221..401662401712..403532403590..403811))

MASEDPIDFAYTAIAQVANIASLLGVESKRPLCGRLGAELFLVANYIDTAGLDQERRLRLRISKLLRDVEAMCNWPKDIPRNGYPLLEAIGRSLPGVTSYEKARKSIRRGLDQLTSCPRDAEVAISHVQKFIERNRKSTRRDESGDLSSRKRFARPERSDLANELAHRVLREQMCCTCQSNGVRRQMERGHLVCLLLQPPSQSVASNNLAQFDMLFSSKPFWDQSQLTHWQDVELLVPNDHTPSKKCVRFLDEDVGNDDQHASKTTAKRRHRTDKGQFCRLLDLATGWRLCLSIEDQELYHRFGPHKQVVDHTPGISLAKILSTYYLTARMKLVLAYIIAYSVWQYYDTDWMRTRWTSETIQFMRESNSSGDGGRGKLFTWKPYVSVHFNDEDPLCYEQYRIAGMVHVYPRIRALGIMLVEIGLGFPLPKREEQGRPLAAEVNTELLTAINYARDEEHWKNFDYPDYKSSICHCLEPGTFDQAPCVESLSASEQKQNLKQRRNILYEKVVFPLENLLQGTKWIDNYEKIGPLHVLSQDAAIQEATVPVNSDKGKAPERRRKRTESEKNASNWLLDVKEFNRELAQVAPAAGLHGLSRRVRIAILDTGYDDNAPFFFSPDVMERLKGWKDWADGSIQPEDCHGHGTHLVSLVLKCAPDADIYVARIAKRPQQLLSASEKVAEAISWASEQWEADLISMSFGFAEEQPCISDAIREALYKRKDSILFFAAASNYGANDREMFPARHESVMSIRATNSNGLFADFNPPKHEDEPVVFGTLGIDVPSAWPNCDGEEYRSGTSIATAIAAGIAGSLLGYISSHPPEKPFYDAKTRLFVPDGTGLDRHS*

>An14g02470.t1

IFEPNGWLQIALQHDVAGFEQAVSTPGHADYGKHFMKRMLLPSETAVDSVRDWLDWVKFVNKANALLDADFKWYRTLQYSIPDALVSHINMIQPTTRFTPHCLKQLYSKIGFASYLEEYARYADLERFEFSVVQFGGNDQEANLDLQYILGVSAPTEYSTGGRNEPLDFLQGILKLNNSDLPQVISTSYGEDEQIPYARTVCNLYAQLGSRGVSVIFSSGDSGVGCLTNDGFPPQFPASCPWVTSVGAEQASSGGFSDLWPRPSYQAVQTYLLFNASGRAFPDVSAQGYAVYDKGFDGTSCSAPTFSGVIALLNDARLRAGLPVMGFLNPFLYLNDIVNGGCSWNATTGWDPVSGLGTPFAKL

>An16g02250.t1

VVDPDGWFWLSMHEYKADFEQKVSTPGHRDYGRHMVMAFMRPSDQVSKIIFSWLDWVAFLAQAQSMMKTDFYNFRTLKYSVPEQVDAHLQMIQPTTRFTPICLRQLYNVLGISGYLDQYARYSDLDEFLFSVVSIGGNPQEASLDIQYALSMAFDTFYTTAGRLEQLQYLVGLPDEDLPAVLSTSYGEDEQLPYTEATCNLFAQLGARGVSVIFSSGDSGVGCVSNDGFQPIFPASCPFVTSVGGEKASSGGFSERFARPSYQSVEAYLLYNPDGRGIPDVSAQAYVIRDHGTAGTSAAAPVFAAVISRLNAARLEQGKPTLGFLNPWLYFTDIVDGGCSWNATKGWDPVTGLGTPYQTL

>An18g02630.t1_gene_An18g02630_Anig_CBS_51388_An18:join(562389..562634562696..563715)

MINTFIKKVEKSTEGRVQVKQTSHQERIIDTRADEETHYSPGHGDSTNEKQYHWVTRMEEFRDAMLKVHEVSKDIVDLPRVRVALIDDGLDYSDFDVYPHTEVRGFSCYPPIGQTEHPWHTSTNGHGTAMANMILRINPWIRLLVIRIHDGVSYANPSSPSRTIHPDSAAHAVEVAIKHNVDVISMSWTLRKRISEFRSSDPGSLGKKSIDEPGIERLEKAIKEAVDNNILMVCSAADDIELLGKDNLPFCAAEHNIFRIGSCNSQAQRDPITENRETISYFLPGIQVPEARRPHSVKPIVYHDGSSIATALAAGSISMILHCARYMAQCRGCQNSSQDGSNESELFKQRAERLRMHKNMQTALNNIVRSHGSEEPEDPKQLPVYGLFAETANNLENAIGWQKIERLGELVRYLCHNVKVD*

>AN5558.2.t1_gene_AN5558.2_Enid_FGSC_A4_chrV_scaffold_6:join(1473206..14735261473586..14740301474088..14741761474231..1474587)

MKSFTLISTTFLLAALPSSFAAPTASNEVSARWFKPDEQPTVKAAQVGDVETQGDDAADDYVVIMAEGETRPWAEIFAEMGYNATEAKTFGTNIRGFTTSMKKSTGVSMFALSNVAIVEKNVIYKAAVMPSNTEPQIMPRNLVKESLRLSSLAKRQNGQQVFIEQSTAPWGLQRISSDKTVVANGRRDTDLTFKYRFDQASGSGVDVYVLDTGLNVNHVDFVGRARSGFSVDGSRGANDAQGHGTHTAGTVGSRTFGVAKNVNLIGVKVLGDEGSGSIAGIVAGIDFVVTEHGKRSNQGDFQGSVISMSLGGDGLPRAMFNALQKASGAGIHISVAAGNENQDACNTSPAGFSRQIPIISVGATDIDDARASFSNFGNCVDIHAPGVDIVSTFSTGNTATRSLQGTSMACPHVTGMIADLLVLNPNLRQDPVGMKRLVISKAQQGVIRAGANVPRGGQVLLNSGFPGVPA

>AO090003001036.t1_gene_AO090003001036_AP007155:join(2791868..27921912792242..27926862792746..27928342792891..2793244)

MKYSLVISTSILLSTFTTTLAAPVNPSTATVPAASSDEGDEYIIVLAPAEKRPWGQVFTDMGYNMTSRAHDISVNKHTFGTNYGDLRTFGKEFRAFTTHMKSTDAASLASLPNVLSIEKDVKWTVKVQRYNDTFGTHNVKDNKWSFPIVKRQAQQDQLNQVVQQSTAPWNLARISTAQPLTSNGREVIDLKFNYQFERSSGLGVDVYMLDSGINTQHVDFGGRAKMVFTAFGNDITDGHGHGTHTAGTVGSTTFGVAKNVNLLGVKVLDSQNAGSLSSFVAGVDFALSSHLQRRTQPGFKGSIINMSLGGPGFAQPLFDALQRATQAGMHVAVAGGNDNTDSCQFSPAQFSQSLPIINVGATNVVDQKWEGSNFGKCIDIHAPGEGIVSTSNKGPQAMEPMSGTSMACPAVAGAMAVELFKNPSLDPASLKKLIVSKALPGVLQGVTGGNVLLNNGLRAVG

>AO090005001380.t1

VVEPDGWLQLAVPEKITEFEQRVSTPGNQNYGRHMVRNFLYSSDVAPRKVLSWLGWITFVSQAEQLLRTRFYTFRTLKYSVPKELRSFVQMIQPTTRFTPSCLRKLYNRLGVSGFLDQYARYSDFHQFLYTVELIGGNLQEASLDIQYAAALADTTFYSTAGRHEPLEQLRYLLDLPDDELPAVLTTSYGELEQVPYARTTCNMFAQLGARGVSVIFSSGDSGVGCITNDGFQPLYPASCPFVTSVGGEMASTGGFSEYFPRPSYQSVNQYLLYNPNGRAIPDVAAQAFIIMDHGTGGTSAAAPVFAGIVSRLNAARLESNKPRLGFLNPWLYFTDIVDGGCSWNATPGWDPVTGLGTPYKAL

>AO090009000291.t1

RSYDFSPAQRLGEGQVGELTQHHTFSKRITDPIFGGQWHLYNTVQVGHDLNVSDVWLEGITGKGVITAVVDDGLDMYSNDLKPNYFAEGSYDFNDHVPEPRPRLGDDRHGTRCAGEIGAANDVCGVGVAYDSQVAGIRILSAPIDDADEAAAINYGFQRNDIYSCSWGPPDDGATMEAPGILIKRAMVNGIQNGRGGKGSIFVFAAGNGAGYDDNCNFDGYTNSIYSITVGAIDREGKHPSYSESCSAQLVVAYSSGSSIHTTDVCYSLHGGTSAAGPLAAGTIALALSARPELTWRDAQYLMIETAVPDGSWQTTKMGFSHDWGFGKVDAYSLVQLAKWELVKPQAWFHSSSYEITKDMMYQANEKLEHVTVTMNVNHTRRGDISVELRSPEGVSHLSTARRSDNAKAGYEDWTFMTVAHWGESGVGKWTVIVKDDWRLNLWGLSIDGSSQRKRLRYDFLYNAF

>AO090011000235.t1

AFEPKGWLKIALQKDAAGFEKTVSDPDHPSYGQHFMKRMLLPRDDTVDAVRQWLDWINFVDTANKLLNAQFKWYRTLQYDVPESVTPHINTIQPTTRFTPTCLKELYSKIAFASYLEEYARYADLENFEFSVTTFGGNDQEANLDLQYILGVSAPTEFSTGGRNEPLEFFQNVLKLDQKDLPQVISTSYGENEQIPYARTVCNLIAQLGSRGVSVLFSSGDSGVGCMTNDGFPPQFPAACPWVTSVGAERGSSGGFSDYWPRPEWQAVSSYLLYNSSGRAFPDVAAQGFAVYDKGFDGTSASAPAFSAVIALLNDARLRAGKPTLGFLNPWLYLQDITLGGCSWNATQGWDPVTGLGTPFAEL

>AO090020000517.t1_gene_AO090020000517_AP007167:complement(join(1324922..13251041325166..13261011326163..1326531))

MKFSKFVLFGLVFFTTANSHPSEAYKSIKSIVHEHSLKLEHVISKRQVSPDTKSFIISFPGASIDDISQHISSIDNLVAGKSEQGITADFDLSESPENPTIIGVAAVLDESVAEAVEHLPFALVEPDGKIYLWDSLERNSDGVVYCDQLPGYGQPGFEPFYDDDLLAELSAPTGDSKVQVATITTEIIGVTSVDGQATTITSTITFTSTLKHAGYTLLAEPTTAGAISPTGSNHGSSGSNGNTAVDNGNNGNSNSNDNGNNINGNDKSGNDVTSFIDDIRVSNSTPTAVESTQLSQSSASSSPILTNSSQSLDSIAFSDSQFISPIVLSTTPNSGSTTPSNSGTITGSEVTSSTSPTSSSGSSSSSTILPSSSSASPSSRPSSSSAPVSSSSPVSSSSPSSSNPGSSSSSSPSSSSPSSSSSSPSSSSSSSSPSSSSSSSSSSPSSSSSSSSPSSSSSFSSSSPSSSSSSSSSSSSSSSPSASSSSSSSTSLSSSSSSSSSSSSSAPLPLVTQQGMPWGLSRISHQSPEMAPYQDPMVGEYIHQQFVDPNPKVVVYVVDSGVNINHDNFATKPIWLANYADSDDSDANGHGTFVAGVVAGTRSGVDPNLQVKSIKVFSGETTDASILMSGITRAINDFKADTTPGKKAVLNLSLGGDVSTALDSLIKQAVAEGMFVAIAAGNNMENACNNSPGRVSTSTPGSVTVGSIDRSDKLSVYAGNNKGTAWGTCITGFAPGSDIMSSMNTPNDGYGIGSGTSFATPMVAGIAGYLMSQEGTKDLTPAELESRIMNSNDGRIQGDLKNSPNKIAYNGV*

>AOL_s00004g122p

MKFSLVLLASVALGLPTTSNNDYRSSVSQVWDIVDKYSTLGHAQLESRDLSDLDSDIKHRYLVTFRQNATQQEISQHFQDVDKWLSSGSNKRDLGFTQLIEGAFSSVSRNSHALNAFAATDSSPFDGYYGKFDNQTVAKVESSSIVDSVESDTIQTSPSFNGHTLSKEAVEEDKTQADKAAALNGFATVTLNKQFTKNWGLDRISHIENDIPEAAGGNYSLIPEMEYIYGPSKYDTVAYVLDTGVNVTNSGFGGRASEPVKFIEGEPPGDYSGHGTHVAGILGSSTFGVAKDAKIISVKVLNMGNAGPTSAVVNGVNWIIQNNNATRAVINYSATGPVSKAMNQAFVKAVEAGITVVVSAGNFMKDACNYSPANLGSVIDGAIVVAASDQHDKFAVGSSWGSNYGRCVNVFAPGTDIPSLHWKNENLVFFDSGTSMSAPFVSGLVLYHQSQSDNPLSPKDVSDKIINSNPGQIQSPMQDTPNGLAYNEALGQAPPPTTTDLGPKIPTGQPLN*

>AOL_s00007g170p

MRQTEGALELTTGMPRSVFVISELETGNKARVAAQNVIKILSNRQLLDDTIPRIKFPVKQGTGPMTNYHDLIDKFIRYFDCEYRALVTRVLGTVATELEKCPPVLGHKIMIQLPSIKETDLFPSDSLHLVKWSLDGFVYCPELSQWHNIKCRFDQESQAKNTEAQRLCEVVKSALQREEGLLLLFDKALAAFIESPELSDLPTMNPNIYPIRNLKEMIMVDGFFNLPSSKLKGSCLFKHTERRELAAKLALFLLMSCDSEKASAMDSWNGNHVTFFSSSDSQKGCNRESPYISCLLGQAPSTSQSFIPNNYDTPRRYTEIARLLMEIEYGPIFDNDEHFSAKNNFGLETIKESLKMHREYDDPTKSNYLDAIGNCLRFGNLFRYEYRSEAIRSTERPADTCRRIIRTEIVLKLLLDLPAFKKPLPKRARPSNNEQFPNSQEKLLDIVDDGDTQIPNQHQASSFSNIGHGLINPVVRGRVDRGTSFGLSSANSCQDSRSKTLSSRPSMIPRRSQGSKTNQGKKKVSFGEASKKNIGSLFDSQESIEAPATAESAKLWFQKLNDLVVDPILSVRRRQADRLVKVAVLDTGIDIAHPMITKAMGENGSKITKFQDWTQSPYSISDRVGHGTAVSEILLRVAKVDLYVGKVSDAAEFDDKTPGIVAQAIEYATSSGGWDVDIVVLSLGFESEDNGIRRAILNAHIRNKIIFAAASNSASLVPELRVSFPARMCGQVISIRSASGQSVRSNASPIASDGDDNFMTLGEGIEAAWPSDLNDGNPTRYVSGTSFATPVAAGIAALILEFSVQQGKVKGDSNLEAADRAILWSHRGVRKIFQVMSTIVNRDNKPDCRMVYPWGLFDNKRKYPGHAMEINRWLQSI

>AOL_s00043g166p

MDIEFKKKSAYRKRGAKNSGENEEPQNEQTKTMQNFQKNFLDLLFNVSGKPADQPLRFLKKKVEEHNEFLETCTGGRNILDKLLGQLVNSYEELPEDILLVIFSSIEYMAVKKPGLLHGKDVRETTALNIIAKCYPDVCMNIVNILIPPNTLDKAKLERNCRGGSDDKYICPLSTINEYLREHSYRKDIAPEHPVSKKYKCFHDLIDLEILEDKNTELKCALESAILPKETTSLGSSTPEHGQGKSILGNLLNEDFIDPINDPDAIEDQLSGFESFLNLCKNEVFSYRNEHGLTPLQQAICFYRDSGKKSINWSFLYEIIKSLVVRFPESIYIESTARATAYTLLGRFESSKDRSSDQTLIKTYTRNPNEQVNPEADNDEMKPQRRGERKLTLLESKKRTEYLLKMNCIRDGSKSHNEKIRYLYSNNGPVRNAPAMEFEEILELVRLPQKWQPKSPTGEMPKVDGLRFSDEDRESRHQDPYTAIFRWLRNDKKVKKIVRVEVDEIVEGVHDHNVSPHSNHAIRQCLQGFDVEELDWRKFDICGNTIFEAVAPKISQSNSSPGREPALRILDLYSSGNTAVLRGWANDDSLSKLKNLKEITIHIRASNRRDRADCEAYKETLKTKLEQFHPSLTVRFDEKMFSSAITDQARGSHGAKTVSLSESKTQWIDKMENFRNCIQRAIDPDRSQPILKNFPTIKVAVLDDGVNFETVPPICIKDAKAFCHKGHLYYRFDNNHGTITASLVREVCPDVELYIARLDDTKELSRDRFHIRSAAEGIQSAIDKKVDIISMSWSFAASPNDEDKNKVREAIQLAASKKILLFASMPDKGPDAKLEDYWPAGLPDVIRIGSATTTGQRSTDNWSSEPEYIFPGEDIELRSEKGENDKKVVSGSSASTALAAGLAALILFYMEAYVQVKSMTAEEAGDIVGKVRTCAGMRASFRALSSKHPTWNDYYVRPYMNKTFNKSVGDRSSSIKGMITDVGTVLADRLQSVIL

>AOL_s00043g49p

MKPSVIIALAVACMAAPMSPQGAYKDALDIINGQASKFNDAGLKKRHGQEPDTFNVIFKDGVSIDQISEHLKLLDGLINKDSLNGIKNAFDLPSQKDGSGLMGYSGHFDKSIVEALGKFSDLVTIEQDRVQKLPTFEGFKSTSNVYESSSYGSDWSMRDDFFSWWRNSNTWGDWWYARKPDTQPGAWPQPKPRPEQPVQPIGDNPTSPAPAPAPAPAPATEEPRPETPAAPSSPATFQEVPTKNWGLFRVSHKQNSRDQTQYAMNKNTLNPTVAYIVDSGIRTSHRMFEGRASWGANFADNSNVDFEGHGTHVAGTVGGAGFGVSPSTKLIAVKVFSGKFGSSSQIMQGVSWAIDDYVKNKAKYPRAVINFSGGGDTSEAEDALFRKAVEMGMVVAVAAGNENSDACYVSPARAGAGTAGFITVGASSADDSLAFWNAQTNKASNWGRCIDVIAPGTEILSADYQTDNGILSNSGTSMATPHVAGLAAQLMATSSELLTPAQVEEQIINQNNGKITGRLNGTPNKLAYNGSGR*

>AOL_s00054g268p

TFEPNGWLRIHLQQNVPQFEKALSTPGHPSYGNHMIDDILRPHEDTAAAVRSWLDWIVVIGKAESLLDAKYKVFRTLSYSVPRNLHSSVTLIQPTTLFTPDCLANLYNKLGVNGFLEQYAQNDDLAKFLFTCTPIDGCTQEANLDIQYTVGASNPIYYSTAGRNEPLEWLEYMLNLSDEQLPQTITTSYGDNEHVPYAIKVCNMIGQLGARGVSVLFSSGDSGPGCTGKDGFVPTFPATCPFVTSVGGEKASSGGFSNYFSRPDYQAVSNYLYYNASGRAFPDISAQGFHVFVRGVSGTSASSPAFAAVISLVNNDRISGGKKPLGFLNPWLYIVDITSGGCGWKATTGWDPVTGLGTPFSVL

>AOL_s00054g992p

YIVFNGSPVHVQGVNRLHGLDGKGIKIAVIDTGDYTHPSLGGKFGGNKVAFGLDLVPKPIDCHGTHVAGIIAGVAPEVTLGAYKVFGCDVLIAFLQAQADGADLITASIGAKVVTRIVTPCSIAAGNGAEGFYASGAADAIGAIGVGSVNNACVVIRGASVFSSWGPTMYQRPHISAPGGILSTYPGTSMATPAGVIGLGRPAPVAQQGAGIVDAYKFTSVDNDTITNGDKQATYKLTEINIKPGIPVYGGWIPYMGGRIPAGTYKLVFRALKMTGDY

>AOL_s00075g8p

MKPSVILSLAVACLAAPMAPQGAYKDALDIINGQSSKFSDSSLRKRHGQEPDSFNIVFKDGVSIDQISEHLKLLDGLINKDSLNGIKTAFDLPSQKDGSGLMGYTGHFDKSIVDTLGNYSDLVTVEKDRIQKLPTFESFKSTQNVYQGTPDAYQGLIDNFFKWWRQGNSPVQDPNNGQGQQPEDDAVQPIGNQPNPIGQFPPPIGGENPFPSNQPSPSKPAPEDPVTAPSTAPSSAPSNGSSPGTGNFQEVPTKNWGLFRVSHKQNSQSQNQYAMNKDTKNPTVAYIVDSGIRTSHKMFEGRASWGANFADNQNVDNAGHGTHVAGTIGGAGYGVSPSTKLISVKVFANMYGSTSQIMQGVSWAIDDYVKNKDKYPRAVINFSGGGDTSEAEDALFRKAVEKGMVVAVAAGNDNTDACGISPGRAGSQTSGFITVAASSSDDSLAIWDPSQNKASNWGSCVDVIAPGTNILSADYQSDDGILSNSGTSMATPHVAGLAAQLMATSSELLTPAQVEDTLINANNGKITGNLNGTPNKLAYNGSGL*

>AOL_s00076g307p

MKLATSLTLFTAVLAAPLAAPAPDVAPAVAEGSASAAYSAILSVVSKQSSKFSALQKRELDEEDRYIVVFDSEASVEQIAAEIAKLDALVDQESSNGITSALDLSAYNDGSGFLGFVGKFNSTIVNQLNQSPILTVEQDTYVPEPKFFAEPVLDKRAIETDENPPNWGLGRISHKQFQGEGNETYVRETTAKHSTVAYVVDSGVRITHEMFQGRAVWGANLLDDIDEDLKGHGSHVAGTLGGKDYGVDVNTKIVAVKVFGANGGGPQSVVNAGFTWALNDFIKNRNTVPRGVLNYSGGGSVLASQDVILARAVKEGLVVVTSAGNDAKDACTDGPANNGAKTHGFITVASLNEHNVNAYDSNWGTCVDVWAPGSKIPSASHKSDTGIVIMRGTSMASPHVAGLATYYMSISDKTLSPGEVEDLITKSNTDVLTFDLKGSPNAVAYNGNGQ*

>AOL_s00076g4p

TPSTGPAAAAYSSILSVVAKQSKKFKHHKRDLDEKDQFIVVFDSSATVDQIASEIQKLDSLVDEDSSNGITSALDLPVYTDGSGFLGFVGKFNSTIVDKLKESSVLTVEPDTIVSLPEIPASSNAKRAIQTTPVTQWGLSRISHKKAQTGNYAYVRETVGKHPTVSYVVDSGIRTTHSEFGGRAVWGANFADTQNADLLGHGTHVAGTVGGKTYGVDANTKLVAVKVFAGRSAALSVINQGFTWALNDYISKRDTLPRGVLNFSGGGPKSASQDALWSRATQEGLLVAIAAGNDAVDACNDSPGNIGGSTSGIITVGSIDSSDKISVWSGGQGSNYGTCVDVFAPGSDIISASYQSDSGTLVYSGTSMACPHVAGLASYYLSINDEVLTPAQVEALITESNTGVLPTTNLKGSPNAVAYNGVGI*

>AOL_s00078g136p

RDYDYSPAKRLGVEPIGRLDDHHLFRKLINDPIFKDQWHLINTREIGHDVNVGRLWLDGIFGENATVAIVDDGLDFKSHDLAENYFKEGSWDFNDPGPDPLPRLSDDRHGTRCAGEVAAANDVCGVGVAYKAKVAGIRILSKSITDADEAVALNYAYEKNNIYSCSWGPPDDGVAMDAPGILIKKAIQQGVQKGRDGKGSIFVFASGNGAANGDNCNFDGYTNSIYSITVGAIDRAGAHPYYSEECSANLVVTYSSGSGIHTTDVCYTMHGGTSAAAPLAAGIFALVVSVRPDLTWRDMQYLCVEAAVPDPDWETTTIGFNHKYGYGKIDAVKLVEAAKWKLVKPQAWFHSTTIEITKEHLENANQRLEHVTVTMDLNHTRRGDLDVDLISPNGVSKIAAQRPKDSSTEGYKEWTFMTVKHWGESGIGKWTIVVKDWWRLNLWGEAINGQKQKKHQAYEFLYDAF

>AOL_s00079g433p

MKVTFFMLAGALALPSDYERAQAVFSRAKSSFVQSSTLNDRYIITFHEQRIDESADQLESLEATVDLTTADLQPLDIPAYQGAGLIGYHGLFDDSTVDLLRSDSRIKAVEKDFETHITASGPIPANISYSGEPETAPTYEWGLSRISHRDNQVNRTCLVNCEGRSYQRLKSKHQTVIYIVDTGVRVSHEQFGGRAIRVKNFNPSEDDEDNNGHGTHVAGTLIGSRYGVSNTHSTLKVVKVFGKNGAAPVSNIAASVAWVVQDHLENPGQRAVLNFSAGGPRIAAYDRVFQHAVKSGLAVAVAAGNGQRDACTFGPANNGETLKGLVVVGATDMWDNIAVFPTWWGSNWGKCVNVFAPGAYIDSTYNTSDNATFSSSGTSMAVPHVAGLMAYFQSVIDTPLSPAELEGLVTAVPNYVRGELNGAANVLANNRFQEAKTNVTLPWWKRQVHGHSE*

>AOL_s00080g47p

MLLINIIVFISVPVFGLVSESVYARVNTSQSLLSSQPTYIVKLKHSYLNSTTFATEPTNFINQHYSIYQKSKLSAAGVNSLESNETDTEDDYIKPIQLNRKLSTVAGSFSPTFVSYLDKLEAVEYVEPNQIYRATRQPSPPTGKRRGRPRFPISPLANWGLTRIHHRKRNDFSEGSADPKGGSDVHVYVFDTGINVNHTEFSGRATMDANFIEGEDSIDLAGHGTHVAGIVGGDTFGVARNVKLHGIKILDRYGDGTTIALLKAIEHVVEIAEPGKSIINLSLSGPRSAMIDDALTSAVLDHNIPIFVSAGNSGDDACQYSPSANEYVFPVGASSEKDSLPFFSSYGPCVKMYAPGTNIISSWLGQDIKIQDGTSMASPHVAGIAALLMSRKYYGTVQELYDTLTEVATRDILSMSPYQAGSSQNLLAYAPI*

>AOL_s00081g154p

MKVIILGLTFTVASAFAQYHATYMPTHIIRFKESVNGTDMANSFVLSQHRSFTIQKHKQQVRRALAKRNSRKTIKMRETNSLSKKEESRMDKAFEEKDTDRSKPESKGEDENENEDEGENNKDNNDKGEEDNDNDSANDDEDEDEDEDEDEDEDDEDKYENEDEDNKDSTSYGNKAKVDDGGFVYAASMGLRLLLEPVSVDPGFTVMTGVFHDKSFLDYLGDQGNVDYIEPNNIYKSNIRLPDEHFGKRSDIRTSPSPDWGLSRISQRLSGTFDSYTYEATAGSGITVYVLDTGVNTEHSDFDNRASLSINLVHNEPETDMGGHGTHVAGKIAGKTYGVAKSARIRSVKILGQSGDGTTASLVKGISHVIETAEPGKSVINLSLSGPKSRMIDEVLSKAARDYNIPIFVSAGNAGTDACYFSPSSNQDVFVVGATNINDEVPLFSNVGQCVHLYAPGSNIKSTWIGSSTAIQSLDGTSMACPHVTGIAASLLSTKNYRNVHELYDEIRSIATKDLLKFKTVVHISQSHNLLAYAPYYL*

>AOL_s00083g321p

MSVRKKKFSLGSVLKNETTSLLAKDLYLNHIDIGNQFSVVSGVFSDHAFLDYLYQQSTIEYVEPNQIYKAAVMPSKRLTEEDEEEERIQRADMDSLKTSKPANWGLARINHRQRGNFDGYIFDSVGGMGIDVYVLDTGVYVDHLDFDNRALHSINLIQHEDQSDMGGHGTHVAAKIAGTEYGVSKAANIRSVKILNKLGDGSTSTLLKGIEHVIQVATPGKSLINLSLSGPHSRLIDDAIDTLVLKYNIPVFAAAGNAGTDACFFTPSSNPNVFSVGAIDIQDRVTRYSDVGECVSIYAPGSAIVSAYVGGNDSSKSMDGTSMASPHVAGTAANMMSKKSFSTPHALYDALRSLATKDVLHFDPQKSSSPNNNLLTYNSVF

>AOL_s00097g257p

MQELSLLVFITTISVALAAPLVSEKRQQPQTVTIVEQPNAPWGLQRISSPDPIPLEIRPKSREMAFRYQYDQSSGQGVDVYVIDGGVNVTHPEFEGRAKMLYNDFKDDNNDVSDHGTMVAGVIGSDPYGVAKNANIWGLRCEMGNGNRTSRCLAQVLLNHQRRRDLPGFAGSVINMSFGSYKMYRSSLEFGNLTALVQAGVHIVASAGNDNKDACDHWPSAANQNPQTQSIISVGASNISDGKAYFSNNGSCVDIYAPGWTLTTIADGGIQQAFGTSLAAPAVAGVLASELVLNPSLRLDPIGLKRHILSKGVPIPSLGVLVNTRINEMQ

>AOL_s00097g43p

MIWKPLVLGFAVLFSLANAAPSHSKAEHYIVKVNDNVVLQDFMPRLLAAALDAVDDLVKKEESRHHRRCLEKHAEIVKTFEMGSFKAFSIEVLDRRVIDALTNKFPEIQMIVPDELIQYDILDVPNHKDSDLQKRYRIRYPRPNYPRQSTDDTDEDHEDNHEEYHRANPRLNRRCHKPAKTVASTNTTSDSLVLSQKNSLWNLARLSVRDRNLNAVYEYEKNAGYFTFIRFFRKSLLTGVTVYVVDDGLLITHKDFGGRAKWGWSPTSTYGKEGGGHGTHVAGIIGGTQYGVAKNVSLTAVQVLNRFGQGTVSTMLSGIEFVIKDAKKHKGRALVNMSLGMPTGSNIATLIDEAVGTLIKNNIPVVAAAGNTPVDACNTVPAGTDNVFTVSSIDNNDTMDPYSAYGKCVDVLAPGISIRSTWIGSSNSETALMSGTSMASPHAAGVAALLMTQLGSNPTPAQVYAALKADATLGKVKDLKPKTPNAIVHHTV*

>AOL_s00110g294p

MGRKLKTTQNIFILFYSSSPLNTYIYIYILIYLYEYTYCSTPEIARHPTMKPQCILISLLVNLAYAEEYLVRFKNPTAFQQFTSNSNRSWRQFIDNKIEKKFSIGSFRGVTMNLSKNLVNKLKKSPLVADIVPNFRFEAFENDGTNDAELNYTFNATAKYLYEDIDEAQNITYQSDAPRHLARISRHYQLPFDIEDKNRYKSWFNYYYEDDYLGQDVNAYIMDTGIFSDHPEFEDRVIQGIDLTKEGFGDQNGHGTHVAGLVGSKTYGAAKKVNLVEVKVLGKDGSGEASNVLSGLEFIVEHCTRVSRPQGKKCVANLSLGSFRSPIINMAVEGAIEEGIVFVAAAGNFNLDAYWASPASAENVITVGAFDDHIDTIAKFSNWGPCVNIFAPGVEIESLSHLNYEDTLILSGTSMSTPIVTGVAAVLLSKGIEPALIAQEIEYLSTRNVFHRRTLFFKPSTANQILYNGVDKLDDPYNDETFPRLNIEAIAKELEEYNATLQNPMADGLHSGSKLWGWNNDVTLPLGEIRLKRRDFIKDL*

>AOL_s00112g42p

MKSQCILISLLVNLVYAEEYLVRFKNPTAFQQFTSNSNRSWRQFIDNKIEKKFSIGSFRGVTMNLSKNLVNKLKKSPLVADIVPNFRFEAFENDSARDVEYNYTFNATAKYSYENLDEEQNITYQSDAPRHLARISRHYQLPFDIEDKERYKSWFNYYYEDDYQGQDVNAYIMDTGIFADHPEFEDRVIQGIDLTKEGFGDQNGHGTHVAGLVGSKTYGAAKKVNLVEVKVLGKDGSGEASNVLSGLEFIVEHCTRVSRPQGKKCVANLSLGSFRSPIINMAVEGAIEEGIVFVAAAGNFNLDAYWASPASAENVITVGAFDDHIDTIAKFSNWGPCVNIFAPGVEIESLSHLNFNDTLVLSGTSMSTPIVTGVAAVLLSKGIEPEMIAQELEYLSTRNVFHRRTLFFKPSTPNQILYNGVDKLDDRYDDETFPRLNIEAIARELEEYNATLQTPMSDNIQSGSKLWGWNNDVTLPLGEIRLKRRDFVRDL*

>AOL_s00140g30p

MKPQCILISLLVNLAYAEEYLVRFKNPTAFQQFTSNSNRSWRQFIDNKIEKKFSIGSFRGVTMNLSKNLVNKLKKSPLVADIVPNFRFEAFEGDSVNSAESSYTFNATAKYSYEDIDEEQNITYQPDAPRHLARISRHYQLPFDVGDKDRYKSWFNYYYEHDYQGQDVNAYIMDTGIFADHPEFEDRVIQGIDLTKEGFGDQNGHGTHVAGLVGSKTYGAAKRVNLVEVKVLGKDGSGEASNVLSGLEFIVEHCTKVSRPQGKKCVANLSLGSFRSPIINMAVEGAIEEGIVFVAAAGNFNLDAYWASPASAENVITVGAFDDHIDTIAKFSNWGPCVNIFAPGVEIESLSHLNYNDTLILSGTSMSTPIVTGVAAILLSKGIEPEMIAQEIEYLSTRNVFHRRTLFFKPSTPNQILYNGVDKLDDPYDDETFPRLNIEAIAKELEEYNATLQTPMSENLQSGSKLWGWNNDVTLPLGEIRLKRRDFMKNL*

>AOL_s00170g103p

MKTTIRFLLLLYICPLIVGEEYLIKLKKSESIQSFLKSTKSQSLQIKDDIKNHIRERFTFGAFRALVIDLPNDMIEKLKKNPTISEIIPNLKVNAFANSSTYSELLTYSDKIIIQREAPRHLSRLSRRGQLPYDINNIDRYDNAFDYYFDAKYQGSSVNAYIIDTGIYKEHPEFEGRVKFAIDLTQEGPGDENGHGTHIAGIVGSKTFGVAKKVNIIDVKALDKKGQGQLSTILKALEFTIRHCKESVDKKCVANLSFGAIRTAIIDEAIKEAHNAGIVLVVAAGNSNIDACWNSPASSKDVITVGAFDDRTETIARFSNWGSCVDVFAPGVKVKSLSHINGSDVLLLSGTSMASPSVSGIAAILLDKGLNPKFVKSKIIESSTKNIFHQGTIMIKPNTPNLIVFNGIEKMDDSFEGARFPRIDFEGLINELKSYRVSVLDGSNMDNYSLGLDILNSSIIDSTTF*

>AOL_s00173g141p

MKVKHFIPQLFLTITFLQEPIYGREYLVSLKQNDSIQSFMDSTITRGRSVKEYLRDKIGKTFSIGSFKGFTVDLTDDLLEAIKKNPLVSDVVPNLRVHAFEDVKDEEGDDDGGQFDNKMQKGKCSVKVQYGAPRHLARLSRRKQLPYDFEDKEAYLDSLKYFYDKRSQGASVNAYIIDSGIYLEHEEFNGRAIPGIDLTGEGSGDYNGHGTHVAGIVGSKSYGVAKKVKLIEIKVLNRLGQGSLAMVLGGIEFAVKHCQETSKKGRCVANLSLGSMRTAILNKAIKAAIDAGLVVVVAAGNNNLNACWYSPASAPEAITVGAFDDRIDAIAKFSNWGQCLDIFAPGVVIKSLSIFKDKEDVTYSGTSMASPTVAGLAALTLDQGVSAEEVKDYILEMGTDNVFHRRSLLFKPGTPNKICFNGVKRNEEEYDDENEEDNEVADTLENIVIPKTGEMPSSIDDSDDKRVAPEEDLVPLGQIHFKRGEENDENRR*MKVKHFIPQLFLTITFLQEPIYGREYLVSLKQNDSIQSFMDSTITRGRSVKEYLRDKIGKTFSIGSFKGFTVDLTDDLLEAIKKNPLVSDVVPNLRVHAFEDVKDEEGDDDGGQFDNKMQKGKCSVKVQYGAPRHLARLSRRKQLPYDFEDKEAYLDSLKYFYDKRSQGASVNAYIIDSGIYLEHEEFNGRAIPGIDLTGEGSGDYNGHGTHVAGIVGSKSYGVAKKVKLIEIKVLNRLGQGSLAMVLGGIEFAVKHCQETSKKGRCVANLSLGSMRTAILNKAIKAAIDAGLVVVVAAGNNNLNACWYSPASAPEAITVGAFDDRIDAIAKFSNWGQCLDIFAPGVVIKSLSIFKDKEDVTYSGTSMASPTVAGLAALTLDQGVSAEEVKDYILEMGTDNVFHRRSLLFKPGTPNKICFNGVKRNEEEYDDENEEDNEVADTLENIVIPKTGEMPSSIDDSDDKRVAPEEDLVPLGQIHFKRGEENDENRR*MKVKHFIPQLFLTITFLQEPIYGREYLVSLKQNDSIQSFMDSTITRGRSVKEYLRDKIGKTFSIGSFKGFTVDLTDDLLEAIKKNPLVSDVVPNLRVHAFEDVKDEEGDDDGGQFDNKMQKGKCSVKVQYGAPRHLARLSRRKQLPYDFEDKEAYLDSLKYFYDKRSQGASVNAYIIDSGIYLEHEEFNGRAIPGIDLTGEGSGDYNGHGTHVAGIVGSKSYGVAKKVKLIEIKVLNRLGQGSLAMVLGGIEFAVKHCQETSKKGRCVANLSLGSMRTAILNKAIKAAIDAGLVVVVAAGNNNLNACWYSPASAPEAITVGAFDDRIDAIAKFSNWGQCLDIFAPGVVIKSLSIFKDKEDVTYSGTSMASPTVAGLAALTLDQGVSAEEVKDYILEMGTDNVFHRRSLLFKPGTPNKICFNGVKRNEEEYDDENEEDNEVADTLENIVIPKTGEMPSSIDDSDDKRVAPEEDLVPLGQIHFKRGEENDENRR*MKVKHFIPQLFLTITFLQEPIYGREYLVSLKQNDSIQSFMDSTITRGRSVKEYLRDKIGKTFSIGSFKGFTVDLTDDLLEAIKKNPLVSDVVPNLRVHAFEDVKDEEGDDDGGQFDNKMQKGKCSVKVQYGAPRHLARLSRRKQLPYDFEDKEAYLDSLKYFYDKRSQGASVNAYIIDSGIYLEHEEFNGRAIPGIDLTGEGSGDYNGHGTHVAGIVGSKSYGVAKKVKLIEIKVLNRLGQGSLAMVLGGIEFAVKHCQETSKKGRCVANLSLGSMRTAILNKAIKAAIDAGLVVVVAAGNNNLNACWYSPASAPEAITVGAFDDRIDAIAKFSNWGQCLDIFAPGVVIKSLSIFKDKEDVTYSGTSMASPTVAGLAALTLDQGVSAEEVKDYILEMGTDNVFHRRSLLFKPGTPNKICFNGVKRNEEEYDDENEEDNEVADTLENIVIPKTGEMPSSIDDSDDKRVAPEEDLVPLGQIHFKRGEENDENRR*

>AOL_s00176g95p

MSTPTETSPLIIKWGLSGFGSRHALYLIHQAMIMAAFQDELKNHKIAFKLHYTVFEKNAGKTGGFGNAFQGNCDASVNTSVTAALPPPLVHDEDVNLRAQDLAEFANLQKAVKNAMESKYDEVLRELKACNPCAADLFKRGFEDRKVIPGVAYVTRGKWGKIQLENIDKALDYIKNNMQDEIEVAFKYPHEVVGVNFDNPQKPKLKVEDLVAKRQFEEEFDFVSFANGTPLVPPFKDSDVGAKHYYSGTPNHSDMKKYLNGRGVLDDDKLKTTAKIACTGLSLSFYDYATLLLAFLPGMDTSEDPVKTVEELGRDKYKGLITVISRGSRGPAPPRTVLDENWHGVGDSFFSARDMHALRLQRNSNWLPIAYEFLEAHIARSENISPDQVRNHGSTEKCMTGYLDDCKKYLAKDNRPTKTGLLRAGYVAFSAGSGIVRDVDQAENKLVLEAKYTREGRFGIPLFGASCFEMSSKNIDKKENAKFFEHWNEQLYYNYASPVSIQRVMAAMFTSGMAVHKQGNFNGFRTEGDGETPKLVYQNSDGKNDEFDALLAPKVFRRDNDCAVSATKDIVRNIIEGVPDYGKGGYFKSSGGDPINAFDSGLGGCGAKHKDGRIVGVRWDGLTNNHHAANTWASSRSYHTLALAIARFLSRDNSESPIETVNKAFERTLPSCKDFKSEIKSFEKDWEDLKERLLFLRLAKKLAGQRSDDYYKITNNIFTPELRDEYISKLKGADKTEYEALENEFRTSKYSFSFVSCDEFERRYPDYTRLQYKAILEKLFASASRDHPAVTSAQGAAPPSSGIENPLIRAVTLGAIAEVLELLDTGTSPNFTDASGLSPLHHSYEANKKRKKDEIFESSEFLGLLGLLFEYGADPDAKDHTGSTLLHYASKEGRDRVTRLLLKYWADPNVKDNSEATPFQLAAKNKHRTIMRLLIAKGADASAGDEPKFALSGPSSEGNTGTDIATPRSEEVALKPLFPIRINGNDLNEAEIKPQSNESNTNYIIVQTAALLSPPQRETLKAAGLDTQEYVSNCTYLCRYTGQDLDKIGQIDPIVYVDIYRQEFKIAPSLKEAIERTLPDDKIEIFVLCHDFESTNDRDFLQLIADKSGLNLWDVKFIDRKTHLTVLSQSIDGIAKIDQVRYIEEVGIAEVSNNRARVTLGLDKLAEDGQALPAHQYQGAGQVIAIADTGLGLGDIAAPPHPAFGDRVVGWYRQNGFTADYDGHGTHVCASAVGNGKTRDNIHIMGTAPRANLVMHSLWRSSEMTIDRPKHFDELFRPPYTEHKARVHSNSWNKPSRGYTTEANEIDSFVWEKQDMVICWSAGNKRAGSTSLAQENEGQIGAEASAKNCITVGACEGEQDHTIVPDYSNCGPLAGDDKCKRRKKPDVVAPGTMILSARSRQMPPSQSGEDPDWYRDTGTSMATGLVAGCAAVLIEAVTANRIGDRQPSAALIKALFINGAEKLVTERVPLPIPNVRSGFGRVNLTNSIKIACQTEGAGFMEGELVWGSKKSFTFRKPPKNECTTVKATLVWSDPPGDLIKNRLFLKVKCGDRQVFSRLYNNVQQVLWDMGSVIYQEVEFTVMAEDDELDQSPQPFDVAWQFS

>AOL_s00188g273p

MKVKHFIPQLFLTITFLQEPIYGREYLVSLKQNDSIQSFMDSTITRGRSVKEYLRDKIGKTFSIGSFKGFTVDLTDDLLEAIKKNPLVSDVVPNLRVHAFEDVKDEEGDDDGGQFDNKMQKGKCSVKVQYGAPRHLARLSRRKQLPYDFEDKEAYLDSLKYFYDKRSQGASVNAYIIDSGIYLEHEEFNGRAIPGIDLTGEGSGDYNGHGTHVAGIVGSKSYGVAKKVKLIEIKVLNRLGQGSLAMVLGGIEFAVKHCQETSKKGRCVANLSLGSMRTAILNKAIKAAIDAGLVVVVAAGNNNLNACWYSPASAPEAITVGAFDDRIDAIAKFSNWGQCLDIFAPGVVIKSLSIFKDKEDVTYSGTSMASPTVAGLAALTLDQGVSAEEVKDYILEMGTDNVFHRRSLLFKPGTPNKICFNGVKRNEEEYDDENEEDNEVADTLENIVIPKTGEMPSSIDDSDDKRVAPEEDLVPLGQIHFKRGEENDENRR*MKVKHFIPQLFLTITFLQEPIYGREYLVSLKQNDSIQSFMDSTITRGRSVKEYLRDKIGKTFSIGSFKGFTVDLTDDLLEAIKKNPLVSDVVPNLRVHAFEDVKDEEGDDDGGQFDNKMQKGKCSVKVQYGAPRHLARLSRRKQLPYDFEDKEAYLDSLKYFYDKRSQGASVNAYIIDSGIYLEHEEFNGRAIPGIDLTGEGSGDYNGHGTHVAGIVGSKSYGVAKKVKLIEIKVLNRLGQGSLAMVLGGIEFAVKHCQETSKKGRCVANLSLGSMRTAILNKAIKAAIDAGLVVVVAAGNNNLNACWYSPASAPEAITVGAFDDRIDAIAKFSNWGQCLDIFAPGVVIKSLSIFKDKEDVTYSGTSMASPTVAGLAALTLDQGVSAEEVKDYILEMGTDNVFHRRSLLFKPGTPNKICFNGVKRNEEEYDDENEEDNEVADTLENIVIPKTGEMPSSIDDSDDKRVAPEEDLVPLGQIHFKRGEENDENRR*MKVKHFIPQLFLTITFLQEPIYGREYLVSLKQNDSIQSFMDSTITRGRSVKEYLRDKIGKTFSIGSFKGFTVDLTDDLLEAIKKNPLVSDVVPNLRVHAFEDVKDEEGDDDGGQFDNKMQKGKCSVKVQYGAPRHLARLSRRKQLPYDFEDKEAYLDSLKYFYDKRSQGASVNAYIIDSGIYLEHEEFNGRAIPGIDLTGEGSGDYNGHGTHVAGIVGSKSYGVAKKVKLIEIKVLNRLGQGSLAMVLGGIEFAVKHCQETSKKGRCVANLSLGSMRTAILNKAIKAAIDAGLVVVVAAGNNNLNACWYSPASAPEAITVGAFDDRIDAIAKFSNWGQCLDIFAPGVVIKSLSIFKDKEDVTYSGTSMASPTVAGLAALTLDQGVSAEEVKDYILEMGTDNVFHRRSLLFKPGTPNKICFNGVKRNEEEYDDENEEDNEVADTLENIVIPKTGEMPSSIDDSDDKRVAPEEDLVPLGQIHFKRGEENDENRR*MKVKHFIPQLFLTITFLQEPIYGREYLVSLKQNDSIQSFMDSTITRGRSVKEYLRDKIGKTFSIGSFKGFTVDLTDDLLEAIKKNPLVSDVVPNLRVHAFEDVKDEEGDDDGGQFDNKMQKGKCSVKVQYGAPRHLARLSRRKQLPYDFEDKEAYLDSLKYFYDKRSQGASVNAYIIDSGIYLEHEEFNGRAIPGIDLTGEGSGDYNGHGTHVAGIVGSKSYGVAKKVKLIEIKVLNRLGQGSLAMVLGGIEFAVKHCQETSKKGRCVANLSLGSMRTAILNKAIKAAIDAGLVVVVAAGNNNLNACWYSPASAPEAITVGAFDDRIDAIAKFSNWGQCLDIFAPGVVIKSLSIFKDKEDVTYSGTSMASPTVAGLAALTLDQGVSAEEVKDYILEMGTDNVFHRRSLLFKPGTPNKICFNGVKRNEEEYDDENEEDNEVADTLENIVIPKTGEMPSSIDDSDDKRVAPEEDLVPLGQIHFKRGEENDENRR*

>AOL_s00188g339p

MKSFGKVGVEDKKGGARPMSKKKIAPENRADITNKHECLLRDLLWGTFQDDDKHTMTKIDLSQYAGVLEKSGYRRSNLLHTLVWELTGDRDPYQDDQDRPDSEVYDEGVPEANVTKRALELVEYLVVLNAGLMCQYDDSRSVPLELFATSYPTILFGIIDMLPSDIDLETMLRKCNTTSTAHATSDNICPLKKLNGCLRDSCLNSDVKNGLEPHKSHCIHPMINTSQLRTKITHIKETIQRFLVSSGNKPNIPQDTFIHVMLGKESFDTAVMRNDQTQLRSFKTILKFFPTAETSVLNQPDSKGYNPLQKAITLFKEGALNYELLYKTIKILVKYQPDSIYFRTKDHREESLGRTAYTLLKEMKPAIGKRAEPFWRKAENLLKKTCIRDRDPRDPNGEKINKIEYLYSDIRSERQIRFDLGHETSTIIDEDYVDFLREKAAMQFESILECVKLPFRTRPVARDSEDPLQYEDRVNRYKTADPYISVFDWLKRDGMVQKIFAIEVDDLGNEPLKSTLTPHSNFAIRDCLRNNFKSQAAVADNKSTQTIYERDRKGMGIEIWNWRKLDICSDTIREAAPGVRILHLYSSANKAVLKGWACKDGLRTLKNNSRDEADCKEYIPAFESKLRKRNAALEVEVFLEPSPKGKGSGENATKNQANSQQSYIYEAETQTDWIQSISRFKTCVRDTIDAISRDLGPNASAPVVKVAIIDDGVILADDGVQGIVRGSSFDFSRQPYFGERSRHGTHMVSSLREVCPMAELYIGRLDDTRSTDGQRFTVKSAIQALEWATKWEVDIISMSWSFKLDNSSCTREEGLAFKNAIDNAVAQGIILFGAMPDKGPIAEDIKRRYPVGLDNVIRITAAAKSGERLKFNQTDVPELLFPGDEVEIGSEESVKITGSSVATALAAGFAALTILCIKLQAAGMLDRSVQAAGTIPRAIETDLSTRLRALRTPGGIRTIFRNVSPKLVNSKDSFVQPHTALPDDIKAGLQGREEAIKFFLNRIV

>AOL_s00210g212p

MRQRLALQLRDELLWEQDSDNKRCVMSKQSCLEAFIWGQKESAEGDNNTTFTHQTRAEIPVLLARSMLHLYNTQWFQHLWDMDRLILCNEVHQIPSGQYSHPYLASTLSMAGGTVNPTNLFVPEEDSCDPLYRHALVLGFGLRLLEIESGTRFPPTDDDIDPEHEEKGALPYLTLQRALSELEGKGQVEDDYLKIAKACLGFHATLKQKRFCDVDPSIRELVAIHNIVFSPLLQLLAQKFRGAAADLLELEAGIRNMRLKSEKSNHKKAESCTLMKPPPRPPAMKMAAPEAPRLPDSDYGCENVIGDDRAFKSLTDGQWNSLQSTSLLEANFCIDKRDNNVQSVVQLSTTGLEVNAELWVRDNDIKKPNAQPQTEGGTMAYEASERSYDNWWQRLDTLNGLLRARSVEKDTSYNHQKVKIAVLDTGISPKHPYADEMAEGMYKDFVDNNCARKQDTNGHGTETLNLIFKAFETPEVYVARVFRNQEADLNTPEHVAKAIEWAQQKKVDIIVMALGFPRSDESIENAINIAAAEHILFFAAAGNWGGQDKVAFPARLDNVICFFSTTPENKNLSSINPPHNHRKAHNFAILGEEIKVPTVSGQPVRTIRGTSISCAIAAGVAGSLLDFSRQPICRDDHRLRNLKRRREMSAVFEELSKDSRDGKYDCVAPWKLLEGTQGNPDRESKRRKICDSLSRAVENCQ

>AOL_s00215g551p

FNGFPLHVQGVHQLHGLDGQGIRVAVVDTGDYRHPSLGGGFGGFKVEFGTDLVPDPIDCHGTHVAGILAGVAPKVTLGAYKVFGCEVLIAFCQAVKDKADIISASISSRAVARIVIPCTIAAGNGGEGFYAGAAGDAAGAISVCSVNNACVVIRLPSPGNSWGPTLNQRPHICAPGGILSTTRGTSMATPAGVIALGQPSPVAQQGGGMIDAYQFTAVDNDTITNGDAEAFYDLTDIRIKKGIPVYGGWIPYMGGQVPAGTYQFIYRALRIGGDY

>AOL_s00215g702p

MKLINLLTIWYLSTIVQIVHGREYVIRLKHKDTLQKFFNSKLLNSVGSKVKDVIGNDKLDKIYSFGDFNCLSAKNLSIDIVERLKRNPFVADVVPNLAFEMFEKGSPWYTSEDNDDDEVDDESEDEDDDEGNDDGDDDDDDDDDDEDDNGINVQYGAPRHLGRISSRGQLPFDIRNENEFKEFIHYYYEDQFKGSNIAAYILDTGIYAEHPDFENRVIVGADMTGEGPGDSNGHGTHVAGILGSKTFGVAKNVTLVEVKVLTGRGSGNLTTVLSGIQFAVDHCRLMRETQGKQCVANMSLGTVRNNIINAAIHEAFKAGLIMVVAAGNSNVSACWTSPASAKNAITVGAFDDRTETIAYFSNWGACVDIFAPGVKIRSLSNSPESHHKHVTHSGTSMASPVVAGLAAILLDKGVEPFDVKDKLLHLSTKDVFNKLTLFFKYGTPNKIAYNGIEDFFENEDDAVDEEEEAKNLLDRFRIFEAVYPIIDNYELAEKLKDYKPSGTVYSSDDEYLDPSWVN*

>AOL_s00215g758p

MKLITIIIYLFAFVSAEEYIVSLKGPKAFQRFMDSQLVQEILESKPIRKRIIKTYSFGNFRGFVIDLPNEWLIKIRRSPFVSQVVPNFSFKAFDEETALSEVYRGRTIVARGENGGSPELQGFKKEECRGDSVYFDKFEASGKDMPQVNGKLLSRYIKTQSEAPRHLARLSRRSQLPYDFNDPTRYESDFNYYYYGWHQGRSVNAYVLDSGVMIDHCDFEGRAYYGADFIRSGNSGDQNGHGTHVAGLIGSKTYGVSKKVNIIDVKVLDSVGSGTLDTVMSGLEFVSNHCNYKGKQGKQGKQGKPVWGSEDSHDDDVESKRCVINLSLGTFRSTIINEAIEELIKQGIVVVVAAGNSNMNACWTSPASVADVITVGAFDDRIDSIAKFSNWGNCVDIFAPGVLVKSLSNKFPYYPIEQTGTSMATPIVTGLVALLLDSGIKPTQIKKRLNELATDNIFPRRTLLFKPGTPNRIAFNGVKRDDDDYHHYSYPNITIEHILKELDSYTTPAITKGVLLV*

>ATEG_01736.t1_gene_ATEG_01736_Ater_NIH2624_scaffold_2:join(2097607..20978922097945..20985192098714..20989252099007..2100096)

MVPNPTYRQSGRADSLGLNQGFMRKDILDELCNRKDHEKILPKVLKAVAETGSLYWDVRNRLGEDPIVQRLKTLVFKHAKSIQDATTALYGDSAEVSELCLDMSDFNRSSHDFEKFVERLTDIVPRQQEMGEESDDEEPISSLPGMVQFEDTLFFVNLPDLNYVKQPCSQETIRKLFNWLGERQGVKTVNRLYIPDNSLNPLSDAFIALYVLDKFTIKALDWRKLDINLDILTSSVSGTSDLTTRGPRRMPTSNTREHLRELNLYSSGNWSVLYHWISEEGLAKLPKGAHDKVTREYYRSLVATYKSSLTKIHDSKKKQNKNNPSLNYRFELAVRTDSRWDYPRPFQEMEEPTVQVPRHKFTSLLTPCRSFMDAQLARATDILLERSVKIKHRHESKTSIPREEFEKLLPPDQLALKLYDHVFATEPDRRIKVAIIDNGADKIRSPIGSMIEKGISYVSSDLLGEAPRPWWMVADAHGTQMASLIGQINPYCRLYIARVGKGRADIDPKKAAKAIHWAVEQKVDIISMSWVTKKNEEELEKAVKEAAQTDKGRRPTLMFCSTADEGAFGGPAYPVDYKEHVVSVSATDSWGGLTSKTDRHTRVDVSIPGEDLEASAPFYLGNVGSTVSGSSVATALAVGIAPLALLLLRTYNDGSEDELSSFYTRKGIMRVFDRMEASKGGVQLQHLFLRDPVEPSNIHGTMASTWKIKKFLEPPELAGT*

>ATEG_02150.t1

VFEPKGWLKIALQHDVEGFEQALSTPGHENYGKHFMKRMLLPSDSAVDAVQTWLDWINLVEHANALLDTQFGWYRTLQYSIPETVAAHINMVQPTTRFTPKCLKDLYSQVAFASYLEEYARYADMVKFQFSVVLYGGNDQEANLDLQTIMGLSAPTEYITGGRNEPLEFLQNILKLDQDELPQVISTSYGEDEQIPYAESVCNMLAQLGSRGVSVVFSSGDSGVGCQTNDGFNPQFPASCPWVTSVGAEQASSGGFSDFWKRPKYQAVAAYLLFNKGGRAFPDVAAQGYAIYDKGLDGTSCSAPAFSAIISLLNDARLREGKPTMGFLNPWLYLNDVVVGGCSWNATQGWDPVTGLGTPFAKM

>ATEG_03179.t1

RSYDFSPAHQLGEGPIGELPHHHTFSKRITDPIFTGQWHLYNTVEVGHDLNVTGVWLDGITGNGVTTAVVDDGLDMYSNDLKPNYFPEGSYDFNEGVPEPRPRLRDDKHGTRCAGEIAAANDVCGLGVAYDSRIAGIRILSEPIDDTDEAAAINFGYQQNDIYSCSWGPMDDGKTMEAPGILIKRAMVNGVQKGRGGRGSIFVFAAGNGASFDDNCNFDGYTNSIYSITVGAIDREGNHPSYSESCSAQLVVAYSSGAKIHTTDVCYSYHGGTSAAGPLAAGTVALALSARPELTWRDAQYLMVETAVPDGSWQILKSGFSHDWGFGKVDAYSLVQKAKWELVKPQAWFHSSSYEVTETMMKDANERLEHVTVTMNVNHTRRGDLSVELRSPDGVSHLSTARRPDEENTGYVDWTFMSVAHWGESGVGKWTVIVKDDWRLNLWGEAIDGAKQRRRKRYDFLYNAF

>ATEG_03900.t1_gene_ATEG_03900_Ater_NIH2624_scaffold_5:complement(join(939035..939388939447..939535939599..940043940107..940430))

MRFSSVLLSLLIGSASAEEYLIRFKSPDGFQFFMNSAKDHTKNFKEFFHNKISHTFSFGSFRGITVDISQDVVEKIRNNPLVADLVPNIQFKAFDDVLGVQELQQNYHGDNLDDEDGFNPFKVKVQGEAPRHLARLSRRAPLPYEFDDELRYEESFNYYYYKWHKGRNINAYIIDTGIYKEHKDFCGRAIFGRDFTGEGPGDRNGHGTHVAGIVGSSNFGVAKKVNLIEVKALNNRGQGNLTTVISAVEFAVNHCKSSGKKGCVANLSLGAVRNSVINQAIKAAHEAGLIIVVAAGNSNINACWNSPASAPEAITVGAFDDRTDTIAKFSNWGPCVDIFASGVKVKSLSAFPPHKPIAFSGTSMASPSVTGLVAILLDKGVEPENIKAKLVELATHDLLQRRTLFFKPGTPNRIAFNGMEREDDEYDDAVYPIVDLESLVQELQNYQPPPLEVNNKDGKFDIINFKDDSPLPMGTIDLTKRKILSPGEEN

>ATEG_06546.t1_gene_ATEG_06546_Ater_NIH2624_scaffold_9:join(799361..799729799795..800730800793..800975)

MRTSLIGALVATSASLAQAEEFLVRLKAPRTLAKAVESRISDGILDVFSEGKILRTFSFGKFEGLTVDFPLSLVSKLRGNPLIAEIVPNVEFNVFDAVEGLEGWGDDEDEDGDYDDDNEEGEDEGDDDVLSIVAQKKAPRHLARISRRTALPYDPENISVPATNFSYYYDDEFSGTCVNAYVIDTGIYKEHPQLEGRAKFGADLVGEGPGDFNGHGTHVAGLIGSRKFGVAKDVSLIEVKALNAKGQGNLTSVISAIEFTVNHCRKSRRGCVANLSLGSFRNTVLNQAVEAALEAGVVIVVAAGNSNANACWSSPASSLSAITVGAFDDRTDTIARFSNWGNCVDIFAPGVKIESLSNVPPFKPVAFSGTSMASPIVSGMVANLLDSGIHPFDIKTELIDLATDSVFHKRTLLFKPGTPNRVAFTGIDKTDDVYEDAVYPSVDIDRLVKDLQSYNSDGHVAEDDATLPLGEIKIKKRSQIVTTLAPPHLSPHSQLA

>ATEG_09889.t1

YIVFNGTPVHQMTVDKLRGYYGSGLRVAVVDSGDYKHPALGGCYGGCLVAFGYDLVPYDNCHGTHVSGLIAGVAPNVTLGHYKVSNCERLMAFKLAFEAKADIITTSVGGVLLQRIVVPCLVAVGNGFYGFLASNGADGKGATGVGAVNNDACVLIRGAADFSQWGPTLVALPTVVSPGGMLSTYPGTSMATPAGCIALANPDSVAHQGAGLINVYEATILNNDTVNNGSESVEYSIGQVDSTIPPGLPIYSGYVPYLGGELPPGTYSLLVRALKIFGDY

>BDCG_01940T0_|_BDCG_01940_|_Blastomyces_dermatitidis_ER3_conserved_hypothetical_protein_(translation)_(881_aa)

MDNKASSPTLATNNEEPTKCEKTSASICATSVFKTTTTKGPITSTITSTSSTCNTIYGCSASDWDTTTTQTQTGNCPLPTALSVQAAAAPPIPPPGCPANAIVYPSDMKDVGQIPQLLEKYEGKYVEIKSEVLRLTTFFWVPFLDQETMNALLQSPDVSEAYYYERWNAQVHPPDPRSSSKGRSHLAVASTLHGQKNGNHFTNGNKTVLDAGNATQFNKRAVKTREHGLIWDLSQISMPRGSIWRSPTSHTVGDDEEFLFHYDDISGDDQYIYIFDEDGVWNDHPEMAYHDDIEILEPYIPYGYVGPIQPDLKHGTRVASKILGANLGICQRCTLIVVNLYGSSQAPLSDLIQERYVEQLLHVLEDVTQKRRNGKAVINMSFQFAEHLLRVPFLNRLYELFTLLDGQNVALLAASSNDGNQYRPINTYPSLFGDPTSPLYLKNLIVVGATNEDGHLADFSHYSNWMTTFAPGEDVYAPTLDEGGYETDDGTSLLSGLVGYYRSLPSPWEAQLQSPANVKKMIKIFHRRFAVQGEPVNFAMMKPTIWNAQVGARSCLSDYHEIPQWDVNRVCPQIELDLENDSNEGETVEPCGPVISNSLQKLAGSYCPNIPGAGSGGHTVSFTSNGGAKPSPTCASGTGCGGHLCTGFFCSPMPTGVPPDRHDPKDPNAGNPVPTTTRPSDPDPTCDDKCKLDRGNRCACNENGCDDQSPGCCANASCPYCECNENSCSPSSPWCCANDTCEWSRTGGGGGYNPRPKPRTGFVLISVSSVENPAPPYIHHYWEVWSKLHPNKVDLCGDEPLVKEKTDDVKGAFPPSLGPFTANGVTCEYIGDKKQAGKLECDTNVAKTSCRALDPIETYECTLENPIKHLTVQCEWEAVD*

>BDCG_03334T0

RNHDYSPAQLLGEGQVGELADHHTFSKRIEDPIFADQWHLFNTVEVGHDLNVTGLWLEGITGEGVISAIVDDGLDMNSNDLMDNYFAEGSYDYNDKSPVPKPRLFDDKHGTRCAGEIAAVNNVCGVGVAYDSKVSGIRILSKPVTDEDEAASINYKYQHNQIYSCSWGPIDDGATMDAPGILIRRALVNGIQKGRAGRGSIYVFAAGNGAGNEDNCNFDGYTNSIYSVTVGAVDRDDNHPYYSEWCSAQLVVTYSSGANIHTTDVCATRHGGTSAAGPLVAGVVALALSVRPELTWRDVQYILLETAIPDSDWQETSIGFSHEFGYGKVDAYSAVHLAKWKLVKPQAWMHSSSFEVTKKMLDMNNERIEHVTLTMNVNHTRRGDLSVELHSPSGVSLLSTTRKNDDHAVGYVDWTFMGESGIGEWTVIVKDDWQLNLWGEAINPKIQRKRLRYEFLYDAF

>BDCG_03663T0

VLKPTGWVSIGLQHGWETLEQHLSDPSHPRYGKYLVNRLVRVAPTSSNLVTAWLDLISFIGVVERLFRANYAEYRSLTWSIPAGLVDHIDVVEPTNSFSIVCLSVLYNSIALVNFLGEVNNRSDIELFLFTTEIIDGDQQEGALDAQTILGLSWPTTYNVGSKNEPLAWLQYMQTKETLPHVISISYADTERVPYARRVCREFAKLGARGVSILVASGDWGVGCVMDNGFAPSFPSSCPYVTSVGAEIVSGGGFSELFERPRYQAVENYLLFNRRGRGYPDIAALGFSVLWNGQDGTSASAPTVSAIIALVNDALLDNGHPPLGFLNPWLYFTDITWGGCGFPALAGWDPATGLGTPFPKL

>BDCG_03667T0

SLKPSKWLQIGLQGRLAELENRLSNPRHKRYGQFLVARLLRPHRDAVDRTQAWLDWLAVIAEAERLLDTKYHIYRAMEWSVPQYLHDAIDTIQPTTSLTSLCLRTLYTKMALVNYLGEFNNRSDVSHFLFEDISIGGNQQEGNLDAEVMLGIAHPTIYSVGKSNEPLVWLNWILDQPDSELPSVVSTSYGDIEHVPYARRVCNGFAQLGARGVSVIMGSGDYGVGCYSNDGFLVSFPDSCPWVTSVGAEVVSSGGFSNYFPRPDYQSIARYLMFNPYGRAIPDVSAQGFVTIWNGIDGTSASAPTFAAIVALVNDALAAEKKPPLGFLNPWLYFQDVTEGGCGFPALQGWDAASGWGTPFPKF

>BDCG_04249T0_|_BDCG_04249_|_Blastomyces_dermatitidis_ER3_autophagic_serine_protease_Alp2_(translation)_(497_aa)

MKFLKIVTSLLLVEIINAEEYLVRLKSPKSLTKFMESKIGDMSLKSFVSTRISKTFSFGKFEGFTADFPKDFVEKLKKNPLVADVVPNSVFNVFDQKEQIAEEYSRRYKVQEDAPRHLARLSRRGPLPYSEEDGWYQEFNYYYDRKHRGDSVNAYIIDTGIYKQHKDLGGRAYFGADMVGEGPGDYNGHGTHVAGIVGSRTFGVAKKVNLVEVKALNAKGQGNLTSVISSIEFSVNHCKKSGMLCVANLSLGSLRNTVLNQAVEAAVESGLVVVVAAGNSNVNACWNSPASAISAITVGAFDDRTDTIAKFSNWGMCVDVFASGVKVMSLSNMPPFKPVAYSGTSMASPSVAGLAAVLLDSGIDPPDIKQKIIDLATENVFQKRTLVFKPGTPNRIAFNGVRKEDDIFEEAVYPVVDINELVKELENYNNGPSDGNVSEDDVTLPLGEIKLTKRNGRRYSVLSPDIWEPTFSRLQDIVM*

>BDCG_04479T0_|_BDCG_04479_|_Blastomyces_dermatitidis_ER3_kp43_peptidase/serine_peptidase_(translation)_(696_aa)

MTLISINGNQLDPLAQSRQLRGLGLQAENASKSDYILIQAGPDRLSENQRRVLKDLGVDIKEYVSKDTYLCSYKGVDLTEIRSLDFVFWANPYLEQFVVQSCLKSNPPRSQPISAFTATPKTSRLQLVDIIMHHDVDATDDAVKAAVARAARADLKSLKASARSIRIHLQQQYLDDVAAIDAVYLIQQVHPFVLWNNKAWEILDCDTTNMSAHVSEYLGEGQVVAAADTGFDIGKTDDTHPAFTGRVKHLYALGRPNATDDPDGHGTHVAGSILGDGDSPSMGGKITAAAPKAQLVLQSVLDSSNGLGGIPSDLSELFIVPYEEHGARIHTNSWGSSSLFGQIPYDVSASQVDKFIWEHPDMLILFAAGNEGLDRNFDGVIDLSQIGSQAAAKNILTVGASENNRPDIGVQYGFRWPSSPFRTDLMADNPAGMAAFSSRGPTVEGRCKPDVVAPGTAVLSSLSRNAQADSQYGESLDPQWWFLAGTSMATPLVAGCAAVVRESLVKNGTPNPSAALVKALLINGAVELVGQYDPSEAGPSPNNNSGFGLVNLRNSIILPNQEDGGFYEGGPLEQGQGLDKPITVNIPPGTSAEGSVLKVTLVWSDPPGAELQNDLDLLVRTANGQERHGNMGEETDFDRVNNVEQVTWTNIPDGEAQIIVSAFRITRDDAPQPYAVVWSINRPIKPTAAGGDKAS*

>BDCG_07545T0_|_BDCG_07545_|_Blastomyces_dermatitidis_ER3_proteinase_T_(translation)_(425_aa)

MQFASLLLLLYIFLGQIYPTEAAKYFVRLKKPHTLDLLFKQDEADASAENRISLHGLRDRIKKKISFGTFEGFVGEFTTELVEKLKKNSLIADITPDIIVSSCDIELQSPAPDHLARLSKEGAVRAQDRLLGPEFFYDGDWTGEGVNVYVIDTGIRVNLDEFEGRASFGADFTGTGKDDSVGHGTHVAGLIGSKTFGVAKNINLISVKALSGNGSGSLSEVLQAIEFAVKHMKASRKPGVANLSLGAPKNSILEKAIEEAFKNGLVIVAAAGNAFVDACNTSPANSPYAITVGAIGDHNDEITRFSNWGACVDLFAGGDTIVSVGLLNGVAVRMSGTSMSAPIVAGLAGILLDQGVAPEDVKGKLIELSDEGKINDNTGILKPGTPNRIANNGIRKSDYEDQKENDNDEDDEDGEDNLEDIEEDEDYWDEERRYREYAVSSLVF

>BDEN_JAM81_01816_gene_BDEN_JAM81_01816_scaffold_21:complement(join(35024..3619936286..3632036423..3705237153..3834438436..3850338605..3880338871..3905639126..3932739412..3949039589..39727))

MTDKTMSRSAFPVEGLLPKNETEAAMFISSNPEMDGRGTVIAILDTGVDPGAPGLQVTSHGLLKIIHLIDCTGAGDVPCSTVVEPTPNADVTTSSLGTIVGLSGRTLTLGNWNCPTNKFRLGLKHSSDLYPGPLVDRLEKSSKEKTLISHHALLTQTEALAVTADNSTATDDNAILTKNDQKARVEVLKDFMKNHEDPGILMDCVVFHDGKTWRAAIDVNETGDLTNVTTLASYSEEHQHLCFGDDSMLNYSVNIYDEGQMLSIVTLAGSHGTHVAAISAANYPEDSRLNGIAPGAQIVSLKIGDTRLGSMETGAGLVRAAIELARLNIDLANISYGEAAAIPDTGRFIELLRDEVINKKGCIVVASGGNAGPALTTVGAPGGTGSAVIGVGAYVSHSMMDAEYALLDKVEERAYTWSSRGPSSDGDIGVDIFAPGAAITSVPQYTIQRSQLMNGTSMSSPNCCGCLALLLSGLKAKQIPYTPYLIKAAIQATGKDIKDPFGIRFVQVQKAWNYLTDTAQGYLPSTLHYAITIPERDDARGIYLRDIVETSELQQLAVKVSPVFPRKDEPSQNLSKLSMEVQVLLKCSDRWISAPNFVLLNNSGRAFNIRVDPTQLRPGFHYGTITGYDANKPEVGSIFTIPITVCKPEVVTSALTETSCYVKYDSLSFKSGDIQRHFVHVPLGANFAELIMRSEGRQTSANFYVHMLQLHPQSRYPMYEKKYTFSLNSVGSGAMNEDSVYRKHFSVLPNVTIELCLAQFWSSLDPSTVSVELKFHGLLASASASTTGGNGLSSGSGGDLMYINPGSNGFARVDITAPVRNESISPSISLDTLRKSIRPTDFTISPLKSRDVLPDTRQLHQLVLTYSTKINESGSITPRFPRMNNMLYDSSLENFGLFVFDSNKRTIAFRDIYPKAIKALEGTYTFRAQVVSHSLDVLEKLQSMPLVLDIALSKSVSLSIYNSLAGAISEDAAMSYKRKTLLRGQRSVFWVGDIASNSVPKDAKHGDLLVGKLDLTSTKIDGDLYPVAYLVSPEIKKDASNDITLAGEKEPIKEDATLITEAVRDLEISWIKKLKSDEERKLLLSKLEKENPTHLPLFKQKLEQLTEKSTQFSSATPMSDELSSQILATTLQIISIIDEGSVAKYFGVQHDVAQGGEKMRTEKSEMEAKKELLIMAYRWKSAAYYSMLDTTKERAEQSTLFVEALAKYAEWLPSSSAADGYYILLSACRNRLRGHHGLALKSLNKFIADRKNVSTEEAGKNLWKAAIELRVSILNELGWNMWADYESKWKSLKFPTEYASF*

>BDEN_JAM81_08408_gene_BDEN_JAM81_08408_scaffold_7:join(878718..878883879062..879288879466..880539)

MREQVKCTLENVLIESHLWYNKNHSHYVKITNTIVGANFVSLYALCNIVYFDIVNQMLISSFYLLTLFNYVVSQGYLVSLKNSKTMEHFRETDLQYPEDQRALPFIERLFKIGNFLAFSGQFPLLVLQRLKKCPVVAEITPDLQVQAFEIMGQKGAPRHLAKLSHVKLEESDFSYYYDDEANGDGVKAYVIDSGVNIEHPELEGRAIRGTNLVSEESGDQNGHGTHVAGIIGSRTYGVAKGATIVEVKALDKFGAGSLSNILASLEFVALDQKNDSLSVVNLSLGAMRNSVLNDAISALVDMEVLVVAAAGNSNINACNMSPASCRDAITVGAINDTSGTMAEFSNWGPCVDIFASGTNVQSLSASDFTVSQTLSGSSMAAPIITGLIANLLSEGVETSEIKDRLLSDSLHNGMPRRSLFFKGRTPNRIAMGIQMQD

>Bfuc_05765.t1

VHEHPRWMRIGLQNNLDKGHEHLSDPASSHFGQHWVIEAFRPSQQTEDEVRKWLGWFAFAEEAENLLYTEYHEYSCEAYHVPKDIRKHIDYISPGIKLTPACVAALYNSMGIFESELQFYTQNDLDLFFPIPANIGGQSTEANLDIQLAYPILYPTLYQVDFNTLDALDGSLMCGTFTPTNVISLSYGGQESLPYQKRQCLEYMKLGLQGVSFLFASGDSGVSCLGFNPTWPGTCPYVTSVGAESASSGGFSNIYPIPDYQFVAKFFIYNRIGRGIPDVAANGIAVYNGNSGGTSASTPIFSAVINRINEERINVGKGPIGFLNPSLYLNDIVNGGCGFSAVPGWDPVTGLGTPYPKM

>Bfuc_12343.t1

FIIFVGWPVLEMGVDKLHGIKGKGIKIGIVDTGDYRHPALGGGFGGFKITGGYSFVGDPLSTCHGTHVSGILGGVAPEANLYMYRTFDCDTIMGMLKAHEDGVDVISMSLAASVVQSITTAVIVAVGNGSLGFTADYPSSEPGAIAVGAIANSTWFEAFSGDYYSNFGPTYDLKPQISAPGGILSTYPGTSMATPAGCFALAAPQQGAGLINAYDATTISGDDIDNSGSTKTYTLSHIGATLAGGPVFGGYIPYIGSAGDGDYRFFASVLRWGGDY

>Bfuc_12776.t1

AVKPAQWLQIGLQGQFDELERHLSDPDHHRYGQHLVNDLVKPSDETHNLVHEWLDWIKVIEDIESLLDTEYSTYRTPEWSLPLHLHDHIDTIQPTNSFTPLCLRTLYNKMALTNYLGESNNRSDTKLFLFDVQVIGGNDQEGNLDSETMLGIGYPIAYTTGGSNEPLTWLQYVLSQKNLPGVVSNSYQDTEQVPYAVSVCKGFAQLGARGVSVLFGSGDNGVGCISNVDFLAMFPSTCPYVTSVGGEVVSGGGFSRYFPRPSWQALKPYLFFNATGRGFPDIAAQGYITVWNGLDGTSAATPAASAILALINDALIAAGKPTLGWLNPWLYFTDVTIGGCGFPAATGWDAATGFGTPFPKI

>Bfuc_B0510:BC1G_06836.t1_gene_BC1G_06836_Bfuc_B0510_scaffold_35:complement(join(283313..284506284758..285129))

MRVQIFFGWIILICSVNAVSQDYLVALKTSETLESFLNYDKRYPVARRLKNFIKKSFNIGTFNGFSGTFSQQDLERLHRCPMVDEITPDINVTAFDLLEQANSPRHLSRLSLFDYGNEDRNSYFYDEGATGVNVNVYIVDSGIEVDHPEFESRAFAGKDFTGEGSGDTNGHGTHVAGIIGSKTYGVAKDVNLIEVKCLDEMGHGSLSSILGAIEFSVNHSRHSRKPGVVNLSLGAMRNSVLNKVIESASNAGLVIVAAAGNLNVDACATLPASARLAITVGAIDDSTNTIAEFSNWGPCVDVFAPGMVVSSVNIYRKRDPQALSGTSMSAPIVSGLVANLLSAGASPASVKSLILSTAHQGRITQASLQRKNQTPNLVVYNGLDPEEIETDSDTDE

>Bfuc_B0510_BC1G_01499.t1

RNYDYDPAGRLGDGQLGELEDHHVFSKRIQDPIFKDQWHLYNPVQVGHDVNVTDVWMQNITGTGSIVAIVDDGLDMYSNDLKANYYAEGSYDFNENTLEPKPRLSDDKHGTRCAGEVSAVNDVCGVGVAYDSKIAGIRILSKMITDADEAVAMNYAYQHNQIYSCSWGPPDDGRSMDAPGILIKRAMVNAVQKGRGGLGSIYVFASGNGAANEDNCNFDGYTNSIYSITVGAIDRKGLHPYYSEKCSAQLVVTYSSGSGIHTTDVCSDAHGGTSAAAPLAAGIFALVLQIRPDLSWRDMQYLVMSTALPTGEWQTTTIGFSHTFGYGKIDTWATIEAAKFKNVKPQAWFYSVSFEVTKEMLQEANERLEHVQVTMNIAHTKRGDLSVDLVSPDKVSHLSASRRYDSEPEGYDDWTFMSVVHWGESGIGTWTITVRDDWHLKLWGESIDAEKARKRLRWEFLYDAF

>CAGL0B03619g.t1_gene_CAGL0B03619g_Cgla_CBS_138_chrB:361572..362978

MHRFILFASFILFTRCETTTFIVSLKPKETLDTFMQYDASYPEELQVKEYITRSFKIGSFSGFTGEFNDQVLDRLSRCPLVDEISSDIVVYSLDTHFQMFAPRHLARLSRRKRLLPIRKYPYVYNNDFAGKKVNAYVIDSGVYIGHPEFQGRARTGRDFSNEGEGDNNGHGTHVAGLIGAKSYGAAKNVELFDVKALNSVGTGTLSTIIAAIEFVVNHRLRSGRPGVANLSFGAHHNAILNSAMEQATRTGLVFVVAAGNSNIDACLMSPASSPYAITVGAIDDHGDSVSGFSNWGPCVDIFASGSNVRSVDFKNIYRSSILSGTSMASPIVAGVVATLLSEGVNPACVKERLIEIATTNRISKTSLYFRPNTPNKIAYVEVEENFMKAGNDSESEDELDSYESPNSP*

>CAGL0E02651g.t1_gene_CAGL0E02651g_Cgla_CBS_138_chrE:251631..253088

MKITQHVVLALLLSTILAHESYLVSLHPKETLSTFMSYDSTYPSHLQVKELIKSSFHIGNFSGFSGNFTDQILQRLSKCPLVEEIVPDITLHAYDLTHQYHAPRHLARISRRKRMIPLRKYPFIYDSNFIGRRINAYVIDSGVAIGHPEFQGRARSGMDFTNEGPGDINGHGTHVAGLIGSQTYGVSKGVTIIDVKALNARGTGALSTILLAIEYAVNHRLRSGRPGVVNLSLGAYKNNLLNRAIEQATLTGMVFVVAAGNNNINACLTSPSSSPYAITVGAIDDFNDSIASFSNWGQCVDVFASGAYVKSVDIRSNVKPLTLLGTSMAAPVVSGIAANLLSEGIDPEMIKDHIIEISTKGMISRSSLFLRKKTPNRIANNGVCTGYKMNQNGDLYFEDFDDCESE

>CAGL0J07546g.t1

KNFDYSHAELSKEHLVRGLDNHYVFSKRINDPLFPKQWHLINPAFPGNDINVKDVWLQNITGKGVVAAIIDDGVDYTSPDLKDNFCKEGSWDFNENQQLPMPLLSDDNHGTRCAGEIAAINNYCGVGVAYDAKVSGIRILSGPLTAEDEAASLVHALDVNDIYSCSWGPTDDGKHLQGPSPLVKKAMKKGVTEGRGNKGAIYVFASGNGGMHGDNCNYDGYTNSIYSITVGAIDHKGLHPPYSESCSAVLVVTYSSGSGIHSTDICYDRHGGTSAAAPIAAGIYALVLEANPNITWRDMQYLSILSSETDGDWQTTKLEYSHKYGYGKLNAHNIVALAKWENVNPQVEFATSTIEITASDLEKAKRSVEHVTINVDISTENRGTTTIDLISPFGVSHLGVVRRKDDSNEGFRDWTFMSVAHWGELGSGEWKLIVSWSITLFGLSEYSEQKRRRYDYLSD

>CAGL0L10802g.t1_gene_CAGL0L10802g_Cgla_CBS_138_chrL:complement(1156934..1158487)

MVNILLSIWILIYFMTGLSFASSSYLVSLHSQETIDTFMAYDATYPQDLQVSELINSKFKIGNFSGFSGSFSEDIIKRLERCPLVDEIVPDITVKAYDATFQNSAPRHLARISRRKRMKPIKKYPYVYESDFIGKKVSAYVIDSGIAIGHPEFQGRARTGKDFTDEGPGDKNGHGTHVAGLIGSHTYGVAKGVQIIDVKALNSKGTGSLSTILVAIEFAVNHRLRSGRMGVANLSLGAYKNKLLNKAIDQATQTGLVFVVAAGNNNINACLTSPSSSPYAITVGAIDDYNDSIASFSNWGECVDLFASGAYVKSVNIRSDFRPSVLSGTSMAAPIVTGLVANLLNEGVDPELIKGQLIEMSTKHRISKSSLFLKKRTPNRIAYNGICYSYYQDEEDCFSD

>CAGL0L13266g.t1_gene_CAGL0L13266g_Cgla_CBS_138_chrL:1416112..1417851

MVKLLLSIWISIYFMAGSSFASSSYLVSLHSQETIDTFMAYDATYPQDLQVGELINSKFKIGNFSGFSGSFSKDIIKRLERCPLVDEIVPDITVKAYDAVFQDLAPRHLARISRRKRMKPIKKYSYIYESDFIGKKVSAYVIDSGIAIGHPEFQGRARTGKDFTDEGPGDNNGHGTHVAGLIGSHTYGVAKGVQIIDVKALNSKGTGSLSTILVAIEFAVNHRLRSGRMGVANLSLGAYKNKLLNKAIDQATQTGLVFVVAAGNNNINACLTSPSSSPYAITVGAIDDYNDSIASFSNWGECVDLFASGAYVKSVNIRSDFRPLVLLGTSMAAPIVTGLVANLLNEGVDPELIKGQLIEMSTKHRISKSSLFLKKRTPN*

>CAR28945.1|_ZYRO0F15598p_Zygos

KDHRYSEAELQEEHAVRGLDSHIVFSKRIHDPLFEKQWHLINTNYPGNDVNATGLWYENITGHGVVAAIVDDGLDYESEDLKDNFCKEGSWDFNDNTKLPKPRLDDDYHGTRCAGEIAAVNDFCAVGVAFNAKLSGIRILSGEITAEQEAASLIYGLDINDIYSCSWGPADDGRHLQGPTDLVRKALVKGVQEGRDKKGALYVFASGNGGAFGDNCNYDGYTNSIYSITVGALDHKGLHPSYSESCSALMVTTYSSGSGIHTTDICSETHGGTSAAAPLAAGIYALILEANPNLTWRDVQYLSVLSAREDGEWQQGALGYSHKYGYGKIDAYAMAKMATWKNVNPQAWYYTSHFTIKEKDLKNANKRVEHIIVTVDVDTDLRGATTIDLISPSGVSNLGVVRKYDNSNEGFKEWSFMSVAHWGENAVGDWQLRVNWRLKLFGESIDASKARRRYEFMLDT

>CAR30036.1|_KLTH0H00418p_Lacha

KDHSYSVPRLLKEHEARGLDKHYVFSKRIEDPLFGIQWHLVNGNYPGHDVNVSGLWYENVTGHNVVVAVVDDGLDYESEDLKDNFSAEGSWDFNDNGPMPKPRLSDDYHGTRCAGEIAAVNKACGIGVAYNAKVAGIRILSAEVTAEDEAASLIHALDVNDIYSCSWGPLDDGRVLQGPDDLVRKALVTGVTKGRNEKGALYVFASGNGGMYDDNCNYDGYTNSIYSITVGAIDHKGLHPPYSESCSAVMVVTYSSGSGIHSTDICSDTHGGTSAAAPLAAGVYSLVLEANPNLSWRDVQYLSILSSEEDGEWQEGALGYSHKYGYGKLDAYRIVTMGRWENVGPQSWYYSSSISVSSEQLKNANKRVEHITVTVSIETTIRGRTTINLVSPKGVSKLGVVRRSDTSPEGFQNWTFMSVAHWGEIGEGDWSLHVDWRLKLFGESLDASKARRLYEFMMGE

>CAX45145.1|_kexin_precursor_pu

RDYNYSQIDFINEHQLSSLDNHYVFSKRIHDPEFTTQWHLINLKYPGHDVNVTGLWLEDILGQGIVTALVDDGVDAESEDIKQNFNSKGSWDFNNNGKSPLPRLFDDYHGTRCAGEIAAVNDVCGIGVAWKSQVSGIRILSGPITSSDEADAMVYGLDTNDIYSCSWGPTDNGKVLSEPELIVKKAMIKGIQQGRDKKGAIYVFASGNGGRFGDSCNFDGYTNSIYSITVGAIDYKGLHPQYSEACSAVMVVTYSSGSGIHTTDICSATHGGTSAAAPLASGIYSLILSANPNLTWRDVQYISVLSATPDGNYQTTALNYSHKYGYGKTDAYKMVHFAKWKNVKPQAWYYSSSVKVTEKDLKIMNERVEHITVKVNIDSTYRGRVGMRIISPTGISDLATFRINDASSRGFQNWTFMSVAHWGETGIGEWKVEVDWQFRIFGESIDGDKARRYEFLFDDF

>CAY68779

YIIFNGYPAHGITVDSLHGYTGSGVVIAVIDTGDYTHPALGGGIGNFPIKAGYDLMDCHGTFVSSIIVGVAPDAQIVMYKVFPCDIVMGMQKAYDDGHKIISLSLGSLMASRIRVVLVAAGNGELGFYASSPASGKQVISVGSVQNCVYILLRGYDYYSSQGPALEFFPTISAPGGSWGAWPGTSFACPAGLTALAVKAPLIQQGAGLVNAVIVSNDTIKNNSETITYQVVHVPGLGPGAPIIQGKIPYMGLSGESS

>CAY68919.1|_Kex2_proprotein_co

EIPTFLEERQINGLDDYHVFSKRIDDPLFAKQWHLFNPRYPGHDVNVSQVWYDGITGKGVVTAIVDDGLDMDSKDLKESFCEEGSWDFNANTRLPKPRLRDDHHGTRCAAEIAAKNKYCGVGVAYDSKVSGIRILSDKITPEDEALSLIYGLDVNDIYSCSWGPADNGITMQGPSSLVKEAMLKGVQDGRKGKGALYVFASGNGASSGDNCNFDGYTNSIYSITVGAIDIKGLHPPYAEACSAVMTVTYSSGSGIHTTDICSDTHGGTSAAAPLAAGLYSLVYQANPDLTWRDIQWLTVLTAVPEPGWQKTAIGYSHKYGYGKIDAYALVNLARFPYLKPQSWIYGSKYELTQEAKDLMNEKIEHVTVTVDIKAAERGKVLVELISPSGVSELAPYRRMDKDKEGFPNWTFMSVAHWGEDGLGEWILKISWQIKFFGESQDPEKAKLERRYEFF

>CHGG_01008.t1_gene_CHGG_01008_Cglo_CBS_14851_scaffold_1:complement(join(3116147..31174183117498..31179913118065..3119367))

MIARQEQPLSNQTDDESDLDLGDDNDFPETAEISGGTEDEVKKAFDRDLRDARDFARQTFANDKEKKKAQMEFVKIRQEGWKRQTEKERRNFLHVLAYYDHTQRPTLQWLMVRAILKLPELMRVLDSSKRTPLTVALSVGNEMFVHATCKNVTDETRKRIGEYLKAECVEHDNDREITCLHVAISANFKPELTAIIISFVPEEMFSVVDFKGRTPLHLAVEFDKCCKTQISIVTQLLHRGPAALDVRTLPDLWKRTHSVFQHHENTRRAAENTSQARNAMKKRQEDAKRADRETDAKIKEAVEMTTAPTKSLHGHGEPRSTAAPGRAIIETGGHGIGGKSFAPGAASPVPHGSGKAPPPLLRVATVNLEVAGGNESGSRGPSSPSTAKGMGPPIDREQERGESADEIKEMLKMVYLRVKKPHEAAHYLHIQDEKEKELWFDFGPPRASSITQEEFRKHFGHLQFDKVLQYVAFPRVELKQQGDALPASEWSQGRRDMTFFFDWLRRKGVQRIIKVIVDDIGLPPHSDEAIEEALQPFGPEILDWRRLDLDPVTLQRVGKNLREVHLQWSGRNTVLRSWSEAEGLAKTPTLESIHILQTEGLSSNERTKKNLSDFKDRLYASWPEDRPKPTVHLPEAANTSRSRASGQPGAFQPSQQQEPSIDPHRWMQCMEEFASCFRQIKGLNDQRSEPALNPVTVALIDDGADITHPELNGKKFRGKSLHEYVDGSGWRVSPYWHSASGHGTLMARLIHRICPSAIIYIIKLQTWIPVGSNKLQIQPQSAIQAIEHAIEQGVQIISMSWTMKPPESAPVRNAFDTAIHRAKGILLFCSASDQGKFQDFTYPHASNPNGSFRIGAAKNTGSMADFVGDAHELNFILPGHNVVVNDRAYADVKDKDFQEFASHTGSSVATALAAGLAALVMECVRLGVFYTEEMKLWEPSLAIHKEDLLAIRDRDAMGYALSSIGVNRQTDNKYIEVWDTFSGVAQRLKYENMRQDQLEIIAGLARMFLRKGVKTTGTTMGY*

>CHGG_01943.t1

RNWDYSPARSLGEGPLGELRDHHIFAKRINDPIFHKQWHLFNTIQVGHDVNVADVWLQGVTGSNTTVAIVDDGLDMYSDDLKDNYYALGSYDFNDKTDEPKPRLSDDRHGTRCAGEVSAGNNACGLGVAYDSKIAGLRILSKLISDADEAVAMNYDFQHNQIYSCSWGPPDDGKSMDAPGILIRRAMLNAVQNGRQGLGSIYVFASGNGAQNEDNCNFDGYTNSIYSITVGAIDRKGMHPPYSEKCSAGLVVTYSSGGGIHTTDVCSNSHGGTSAAAPLAAGIFALALQVRPDLSWRDMQYLAMNTAVPTGEYQDTTIGFSHTFGYGKLDSSAIVEAARWKKVKPQAWFYTVEFKVTEAMLKEANQRVEHITVTMNVEHGRRGDISVDLISPNKVSHLSVTRKNDESTEGYDDWTFMSVAHWGESGVGTWTIIVRDDWHMKLWGETRDASKARRRLRYEFLYDAF

>CHGG_02179.t1_gene_CHGG_02179_Cglo_CBS_14851_scaffold_2:complement(join(131617..131741131806..134179134344..134361))

MSYQPWKRRLEHDGLIQRLLEKLEAALSASSPKIRTGPRNVHLGGHVDFPLLTNIAAAIEDASDGGQKSKHAPVIESSIKSALSRNKIDYNDIFSAFMEVSRIGEEDDGEPEQIPAPELHSGKDDLAGYEKYAKALYKLLVRYGSCNCERQNYDDHHWARLRLKALYQADKHNQISFDILLSVLPNPSRTVQFEWQDVRVFVPTSKNKSPRRVQWAEEHALSLPRQANQLGGTDTKTYERIETLCTLLSSRCGSLLCFQAASDHLKVLREATDLVSHHTVRLDASPGLHLGQVLDRFHMRHGMRPVLAYILAKAAWYYYDSEWTNMGMTKNSVYFMGEALEDEVVYFCKPYLSAQLPPGVSQTVECRQVVGMIHRYPRILALGIMLVEIATGQRFEMEGHPDQWDPRTSNQQLLSFQKLASSGEFHEDCRFPRYKTAVNKCLDPMLFRHAPFNPSEPTENLEKRRSIFYHEIVDPLRQLIEGTGWDAELDDFERTALVPKPRAVKTSKPAAQISISEIATPPVPEKDPWLEEVAVLNTMLKRERKKSSTRSGPFKIAILDTGYDESSPSFDLPGRSRRIKEWRDFACSSPHPVDTDGHGTHLLTLLLQLECPAHIYVARVTENSKTLNSAEASIAEAIRVAGLEWDVDFISLSFGFSRHVQGIRDAIADVVHNKRGAITFFAAANNDGFNSREMFPANLGESVISVRGTNRAGGFESKYNPPTTSDEPVFGTLGVDVLSDWPGLETGKLMSGCSVATPIAVAIAVMLLEYAAARPRDFEPGDLKLMRTRRGVFEMFKEISVHAGDHRHYVAPFNLFRLSEDVRLAKLKTALGRHPEKW*

>CHGG_03658.t1

YYVFKGYPVHLMTVNRFKAVTGKGIKIGIIDTGDYLHPALGGCFGGCLVSYGTDLVPDPLDECHGTHVAGIIAGAAEGVTLGSYRVFGCDILIAYNMAYEDGSDIITASIGAAVVSRIVVPCVVSAGNGAAGFYASTAANGKRVTAIASVDNNGCVLVRGASDFTSWGPTVEVKPQFSTPGGILSTYPGTSMACPAAIYALARPAPVAQQGAGLLQAWDATLLSNDTISNGATAVTYSLSNIGATIPAGLPVYSGYIPYLGGKLPAGRYKFAVKALRIFGDY

>CHGG_05327.t1_gene_CHGG_05327_Cglo_CBS_14851_scaffold_3:complement(join(182194..182754182834..183188183306..184012184232..184341184397..184702184814..186123186194..186201))

MARIEPAAVPGKDQPTLPSSMVSQASPIEPHDSEGPEPFGRSWHHPGQRQRKMKGAKALTRSLEEQRVNLLEKIKEAGRDWPNRPGIKEAARPCLRGDNWTILHWVLWRADHGTKGSDLSWNLEASLALVQLSLQLEPDLLLAKDKKEDTALHKALAMASDAEPDQHFTFEQLANWMCKSAGDATVKKVLDITNSFGETCVHIAVVGQLEIANNLVLLSDARTLLRQRRSEQKGSVQNNTALHDAVAYERNMTTEARCKEQETCPKCSEAEHHMWKKRERVLQLVKNLIQKAPSALTIMNSDNESPYRYYLATKAYATSHGNADAGTQGRTIQSIPGVKEDGMRSFPQPRIDNASLTGDMKLGMKAEKPKPDQLLGGHTKASLNGEDGGSEEPDEPTEQCPNPVPSDSIVAEVERYLFEGAFTLGGFEKACECFFGKVGDKNYKDPNFRPGHALGMEEQKGYSFLKPPKIMSLVDLIVPVTKPEPQEPKTAHRSLEESQQEFRNLSRTLWEQDMDAMRGVFGMLAGMGVERILKLIVTDDAGTLTGGKKTYQPTRSSPRLRGLPSYGCTPVAGMPSSKGFESYEKYIENIQSFDTRLKQNIAHSRFRRVLQCKMETSRHRLVYEETQHERDSRELQSVQEHLAELRAERHEVPKLGVKNEQTADTESDAELAEEIDEYKEEEEYLVDNLEKAQKIMERYRTTLESTSKECRQLDHQMADCLNTRMQSIKVFESNRKSHISTVKDGTTVAFKPSPYPPLLGKSLQGHHHTFFLLSHGSVASNFDLRTIMDDPPRKKTVDDTEIALEEVDEIEATTKHRWLESVDQFVSKLPYDGGRGGETDFRVKVALIDDGVDANQDIFADMFGPEGWPVLEPGSKRYPFYKSAAGHGTEMAKLIRRVCPKIELYVAKLGDWTDQERVERLGEVSTAENAAKAVDWAIRRHVHIISMSWSLVKVDANKEQIALLDSKIQEAARKGIIMYCAAADQGQYGSDLELYPARADTKHVKAVGSARENGAASTSVDPSQVEYLFPGEEIEELGKRKGSSAATALASGLAALVLCCFEKQLGKGGSSRIANPKNMHEVFEKLKAKDSKWVDATRLFKEGNTIETVVKLCTKWIT*

>CHGG_06380.t1_gene_CHGG_06380_Cglo_CBS_14851_scaffold_3:join(3363502..33638733363948..3365180)

MLLSYLFVLLLYSIGGSIAIAIYEKQPYFVALKASESLEKFMAHDKTYPQHLQVRDLIASSVEIGDFRGFAGKFSKEIVERLKRCPLVREVAEDTIFQAFDYAIQEDAPRHLARISRRKKMQPYKKYPYIYDSEFMGQGINAYVVDSGVNIDHPEFEGRAKAGKDFTKEGSGDANGHGTHVAGIIGSVTYGVAKNVTIIEVKALNSKGSGSLHTILHSIEFAVQHSLETGKKGVVNLSLGAFKNRQLNEAIKSATKQGLVFVVAAGNSNVNACLTSPGSSEYAITVGAIDDYNDAMASFSNWGECVDVFASGAYVLSVDAKNNGSSSQVLSGTSMASPVVTGMVANLLSEGVEPGDVKAIVLNMAAKNRISRTSMFFKKHTPNKIVYNGIKEKVLMWENLKQEEF*

>CHGG_06857.t1_gene_CHGG_06857_Cglo_CBS_14851_scaffold_5:complement(join(117760..117954118029..118541118600..118785118845..119132))

MTPTFRKVSPNLRKFQLTIFPSLAFNSYNFSTSIFCYAKIPNLVVYLIRTINTSQIPSSYVYRFWYSVMLIKSNLLLIALVCICAADEPYFVSLKTTESLDNFMEYDQRYPKHLQVRDLISSSVSIGEFKGFAGNFPKEIIDRLKRCPLIQEIIEDSTFENLEYEVQDNAPRHLARVSRRKRLRPNKQYPYIYDNKFMGQGVNAYVIDSGVEVNHPEFEGRASAGIDFTNEGSGDSNGHGTHVAGLIGSATYGVAKNVNIVEVKALNSNGAGSLSTILSAIDFVVKHRIDSGMKGVANLSLGAYKNHILNKAIEHATKTGLIFVVAAGNSNINACTTSPASSKYAITVGAIDDYNDSVAGFSNWGECVDLFASGAYVKSVNARNPYSSSVLSGTSMASPIVTGMVANLLSEGVAPSKIKPTLLKMAAKNRITKSSLFLRKHTPNRIVYNGIKEKALLAEKLSDENENENENENE*

>CHGG_07661.t1

VFEPAGWLRIALQPDEALFERTLSDPAHARYGQHLVASLLAPRAESTAAVLAWLEWVNVVRQAAALLEADFGVWRALRYSVPDEVAPHIRMVAPVVRFTPECLRALYSLFGVAGYLEEWAKYDQLELFSFTAVGVGGNEQEANLDIQYAVALSYKTYYSTAGRNEPLDFFSYLLKLPDQKLPQTLTTSYGEDEQVPYAEKVCQMIGQLGARGVSILFSSGDTGVGCQTNDGFLPIFPAACPYVTSVGGEQASSGGFSDIWPRPSYQAVSKYLLYNPEGRGFPDVAAQGYHVFSQNVSGTSASAPMFAALISLLNNARLAEGRPPLGFLNPWLYLTDIVHGGCSWNATPGWDPVTGLGTPFDKL

>CHGG_10086.t1_gene_CHGG_10086_Cglo_CBS_14851_scaffold_7:complement(join(1576304..15767961577098..15779841578046..1578423))

MLSIIIIGVLLITGCFGDELYFVSLKASESFDKFMEYDKRYPKHLQVRDFISNSIAIGEFKGFSGRFSKDILDRLKRCPFVLEITEDIILSAFDFEIQENAPRHLARISRRKRMKPNKQYPYMYDGEFLGQGVNAYVIDSGVEVNHPEFEGRASAGHDFTNEGSGDSNGHGTHVAGLIGSSTYGVAKNVRIIEVKALNSKGAGSLSTILAAIDFAVNHRLDSGRKGVANLSLGAYKNHILNKAIEQATRTGLVFVVAAGNSNINACLTSPASSKFAITVGAIDDYNDSVTAFSNWGECVDIFASGAYVRSVDAKNHEKTQVLSGTSMASPIVTGMVANLLSQGVEPCDVKASLIRMAAKNKITKSSLFLRKKTPNRILYNGINERDFESFDDED

>CHGG_10867.t1_gene_CHGG_10867_Cglo_CBS_14851_scaffold_8:complement(join(472658..473178473234..473387473444..474043))

MYWVPFERGFSFIKKNGSNPVLFELTEMLINLACVFIALIDKCFSNDSYFVSLKAEESIDHFMEYDKRYPKHLQVRDFISNSIAIGDFKGFSGRFSKDIIDRLKRCPLVQEITEDIMFNALDFEIQEDAPRHLARISRRRRMKPNKPYPYMYETEYSGQGVNAYVIDSGIEVDHPEFQGRASAGYDFTEEGSGDNNGHGTHVAGLIGSATYGVAKNVRIVEVKALNSKGAGSLSTILAAIDFAVNHRIESGRKGVANLSLGAYKNHILNKAIEQATNTGLVFVVAAGNSNINACMTSPASSKYAITVGAIDDYTDSVTAFSNWGECVDIFASGAYVRSVDARNHDKTQVLSGTSMASPIVTGMVANLLSQNVESWKVKSVLIRMAAKNKITKTSLFLRKKTPNRILYNGVQERELENNEDD

>CIMG_00167.t1

LLEPDGWMKINLASQTEDLHQKVGTPGHARYGLHLINSLMTPNEVVLNDVLKWINWIDIIGAASKMLNARFYEYRTTEYSAPKSVAQHIFYIYPLILFTPNCLRGLYNKIAVSGYLDQYAQYKDLAAFLFSVSLVGGNIQEANLDTQYAVALTYNDFVSVKGRNEPMDQLEYLMGLPDKDLPTVLTTSYGETEQVPYARATCNEFAKLTARGVSIIFSSGDTGVGCTSNDGFNPIFPASCPFVTAVGGERASAGGFSNYFKRPGWQAVTKYLYYNPLGRGFPDVAAQAYPIYEKGAAGTSASAPTIAAIIAHLNEVRLSQGKPVLGFFTDITHEGCSWNATKGWDPVTGFASKLNVL

>CIMG_00625.t1

RDYDYSPAQLLGEGRIGELQDHHTFSKRISDPIFVDQWHLFNTEQPGHDLNVTGLWLEGITGNGTVTAIVDDGLDMYSHDLKDNYFAEGSYDFNDKGKEPRPRLVDDKHGTRCAGEVAAVNDICGVGVAYNGKVAGIRILSKPVTDEDEAAAINYGFQKNQIYSCSWGPVDNGATMDAPGLLIRRAMVHGIQQGRGGKGSIFVFAAGNGAASGDNCNFDGYTNSIYSITVGAIDREDKHPYYSESCSAQLVVTYSSGGTISTTDVCSNRHGGTSAAGPLVVGVVALALDVRPDLTWRDIQYLIVETAIPEPGWQTTAIGFSHDFGYGKVDAYSLVQLAKWELVKPQAWLHSSSFEITEELLKKNNERVEHVTVTMNVNHTRRGDLSVELKSPSGISYLSTTRSGDFEKKGYVDWTFMSVAHWGETGKGKWTVIVKDDWQLSLWGEAIDGKIQQKRRRYEFLYDAF

>CIMG_01394.t1_gene_CIMG_01394_Cimm_RS_supercont2.1:complement(join(3653911..36541523654227..36543323654391..36549273654995..3655306))

MYWVPIERGFLIIRKEGANPVLIELTEMLINLACVFIALIDKCFSNDLYFVSLKASESIDHFMEYDKRYPKHLQVRDFISNSIAIGDFKGFSGRFSKDIIDRLKRCPLVQEITEDIMFNALEFEIQEDAPRHLARISRRRRMKPNKPYPYMYETEYLGQGVNAYVIDSGIEVDHPEFEGRASAGYDFTEEGSGDNNGHGTHVAGLIGSVTYGVAKNVRIVEVKALNSKGAGSLSTILAAIDFAVNHRIESGRKGVANLSLGAYKNHILNKAIEQATNTGLVFVVAAGNSNINACMTSPASSKYAITVGAIDDYTDSVTAFSNWGECVDIFASGAYVRSVDARNHDKTQVLSGTSMASPIVTGMVANLLSQNVESGKIKSVLIRMAAKNKITKTSLFLRKKTPNRILYNGIQEREFEYNEDD*

>CIMG_01750.t1_gene_CIMG_01750_Cimm_RS_supercont2.1:complement(join(4607963..46082044608257..46083624608415..46089544609013..4609327))

MLFTKLLFILAFYFNFIHAENYLISLKNNESLEAFFKYDILRPATEQVRALLTNSFSIGNFTGFVGDFSKTNLERLKRCPLVNEITPDVIFKAYGTTTQEQAPRHLARLSSKKKLKSGKSYQYVYNDDYTGSGVYAYVLDSGVAIGHPEFQGRARFGKDFTSQGSGDSNGHGTHVAGIIGSSTYGVSKNVEIIEVKVLDSLGSGSLSTIISALEFSVNHRKRSGKMGVANLSLGSFRNGVLNSAINAAADTGLVVIVAAGNSNINACLSSPASAEGAITVGAIDDYNDSLASFSNWGECVDIFASGAYVKSVNAADYNNPETLSGTSMASPAVCGLAANLLSEGVPPHKIKSKLLSLSLKDQIKRSSLFLRRGTPNRIAYNGIDDEYRDDTDSDSDDD*

>CIMG_02188.t1

LAEPDGWLKVSVAKDPNLLHRSLSTPNHPRYGQHMVRDIVAPHPEASDGIMAWLDWIDFVDTAERLLNTRFYHFRALEYSLPSSLSKHVRTVQPTTFFTPSSLRELYNLLGVSGYLEQYARYSDLNAFIFSVELYGGNDQEASLDIQYTVGLTYNTYYSGGGRNEPMEQLKFFANLSDSRLPTVLSTSYGENEQVPYAKAVCDEFAKLGARGVSVIFSSGDSGVGCLTNDGFNPIFPAACPYVTSVGGESASSGGFSEIFPRPSYQSVDAFLYFNRNGRGFPDVAAQGYAVYDHGVGGTSASAPLIASVISNLNEVRLSQGKPVLGFLNPWLYFTDIVDGGCAWDAVEGWDPVTGFGTPFKKL

>CIMG_02881.t1_gene_CIMG_02881_Cimm_RS_supercont2.1:complement(join(7771237..77714787771544..77716497771703..77722427772303..7772614))

MLLVKYFFFVVLCYNSIFVAAVPYLVSLKSTETLDTFFKYDVNYPLSQRVKALINNSFAIGNFTGFSGDFSKAVLERLKKCPLVADLTPDIIINAFDVGIQTSSPRHLARLSQKDKLTGNQFNYYYDTSCTGNGVNAYVLDSGVMIDHPEFQGRAFKGKDFTKEGFGDTNGHGTHVSGIIGSKSYGVSKNVNIVEIKALDKSGAGSLSTIIAAIEFAVNHRKISGKPGVANLSLGATKNAVLNRAVDAAFDTGLVMVVAAGNSNINACTTSPASASSAITVGAIDDKTDGLATFSNWGECVDVFASGTFVESVNTKDHRYPQVLSGTSMSAPIVTGLVSNLLSKGISPFDVKDHIIEMASKHKINKASMFLRRKTPNRIVYNRVVQESDLDSDSDSDSDE*

>CIMG_03747.t1_gene_CIMG_03747_Cimm_RS_supercont2.2:join(2082253..20825552082614..20831502083222..20833272083430..2083668)

MRVLHLLTLLAVGFALDVPDTSNPGDTLNIRDIIEEPRNFTIDDPLQAQQNLFQPSLPIGKLDLIVKLADTASVSAYLGVYPEAKDYIKSTFAFGKFKGFSGAFDSSFLSTLRRCPWVVDISPDVMVHTTQRFPSSRLRQNDAPRHLARLWSRRRLPDDDVSGTVPADDAPSPPLGTNSPLWFVHNPYNTENVHAYVIDTGINVDHPEFGGRANAGADFTGEGSGDTNGHGTHVAGLVGSRTYGVAKRINIIEVKVLGRNGDGSLSQVIAGIDFAANDRARRDLVAVANLSLGATYNRLLNDAIDAAVDSGLPVVVAAGNSGVPACTSSPASSSKSITVGAIDDRGDTIASFSNYGSCVDIFASGVFVQSLSNDYGTMALSGTSMAAPIVAGVVGTMLAQGVPADEVFPELLRVATYDQIPRMAVQMRPFTKNIIASNRF*

>CIMG_03887.t1

VVEPETWLHLAMNDVLDQFEQRVSTPGNEHYGEHMVQAFLQPPSYTSDAVLAWLDWLHFVKKAEELFNTQFYYYRTLEYSVPKIIAPYVHMIQPTTKFTPDCLRDLYNKLGISGYLEQFARYDDFARFLFDVVSIGGNDQEASLDVDYAIGLSGVYYTTAGRNEPLDQLHYLLSLPDDQLPSVLSTSYGENEQVPYTDMTCNLFARLGARGVSVIFSSGDTGVGCQTNDGFLPVFPAACPFVTSVGAERASSGGFSDRYRRPWYQAVGHYLLYNPAGRGFPDVAAQGFSVVDHDVSGTSASAPVFAAIVANLNSIRQAKGKPVLGFLNPFLYFTDIVHGGCSWNATKGWDPVTGLGTPFEVL

>CIMG_03989.t1_gene_CIMG_03989_Cimm_RS_supercont2.2:complement(join(2680042..26801032680175..26805202680575..26806632680730..26808262680886..26812362681305..2681367))

MLSPLSITCQSLTQIKDWTFDIDVKIYDVQANAPRHLARTSNLEPISAFGPQDFVHDPSDGSGVDIYILDTGVRHQHIEFENRGKPGRDFTGEGMGDQNGHGTHVAGLAASKTYGIAKKANIIDVKVTGEDGMGALSSVLAGLEWAAAEIHRNGRPAVINMSLGAPKNSVFNAAVEAAIDSGIPVVVAAGNTNTPACLDSPASANGVLTVGAFDDRTDTIASFSNWGQCVDIFAPGVEVISLSNQNNRGTVAQSGSSMSAPLAAGLVAYYLGMGDEPHKAMTRIRDWANMNRLSRRGMMFKPFTPNKILFNLAGEPLW*

>CIMG_05557.t1_gene_CIMG_05557_Cimm_RS_supercont2.2:join(7107073..71073817107442..71079787108067..71081727108242..7108507)

MRGILTLFVIWLHFVYPVICTEYLVSLKTPRALRYVFNFKIGGSSVKELIESKIHREFSFGKFNAISVDIPPVLADKLKDVPFISDVVANVKISLFDEFPQIASGLNNDQYDFETEDEEEAEEDEGSDTYEDDDNYDEDTDNGDTDFSDDFISQSRAPRHLARISRRAQLPVIEKDDTSSGLKYHYHEEHQGEGVFVYVLDSGVNHDHPDFEGRAMKGIDTTGEGSGDLVGHGTHVAGLIGSKTYGVAKKVNIVEVKVLDSMGKGTVTNIIEGLEYVVNQCIGKKRKCVINMSLGAPGTHSILDKAVSAVIEEGVVVVVAGGNSGLNACWYSPAKVRKAITVAAMDDRTDLMAPFSNWGACIDIWAPGVNVKSLSNKGRGTFVLSGTSMASPIVAGAAAIYLDQGADPAEVRKILSKTATRGVFKKRAIAVRPRTPNKVLYTGNNQEDDEFAYAVYPMIDEIKFMDQLKQATSRVKPPSGEMINLVTHDRKILKPRLSKFLKKVSNNS*

>CIMG_06672.t1_gene_CIMG_06672_Cimm_RS_supercont2.3:complement(join(2599037..25992222599288..26002262600289..2600657))

MEAAKVPKHQEECYTRDQFIGSYRVLYLSDGISAEEYVITLKAPKTLSKLLAVKLGNRTLKQILDARIKQKFSFGKFEAITVDIPPFLADTLTENKWISHISLNEEYNLLGDEYAGEEDTGDDNGDEFENESDDDDGDDEDEEEEEEEDGDGEDGDDDDDTEEDDSRGPPRPEYPVAPPSPQRPVFPPEPETPIEVPDPEPEPPVEFPEPEEPIEKPGPKRPVNPPEDEEPYNPPEPEAPAEPQPDHPISPPESEQPSDPDTQPPNDPPSFDPQPDQPVERPDLDRPEPEKPGNDEPSEGAPEFPTNPEEPQPPSDSPETPEPSNPPESDPIVPSDPETPSDPEPEQPVDVPVPAPVTPPGPEEPTSTPDPDSPPDKEQRPDDFDQPRENPNDQQPSPNPDRPEPIRGIPAEPVAGAPGEGLHQYAIQVDAPRHLARTCRRTGLPFDPSGDKEYTFNYYYDEEHLGEGVNVYVLDSGIYKSHPEFDGRAHFGIDTTKEGPGDLNGHGTHVAGLVGSRTFGIAKKANIYEIKVMTVDGRGSTSNIIAGVEFAVKHCQQSGKRCVANMSLGGLSFGHSALDSAVEAAIEEGLVFVVAAGNSKERACWYSPAKVKTAITVGAFDDRSDIIASFSNYGKCVDIFAPGVAVASLSNSPSNTMRVLNGTSMSAPITAGVVAVLLDQGIEPHEVKDHLLSISTKGLFHKRRLVSSRTPDRALFTGVKREDDSFSTEKFPFVNEEAYLKFIGDDLKKVQGPSLFRKVLRSSLSKVI*

>CIMG_07023.t1_gene_CIMG_07023_Cimm_RS_supercont2.3:join(3504753..35050643505120..35056593505731..35058363505917..3506158)

MRLSNIITLVIGIISLSNAIVLPDINTLEQTSESQEYDNFFKQSKRPGNVKAPLVAPLGENDEIQNNIVPYRYIVMFKNFAYTNEIKLHIQTVAKLQLRGSISLAPNDPFFHHTRDKEFSTEELGGVLYTFDIGSGFNGYVGYFTDEVVEFIRKQSIVDYVEMDSILTIDDKETDSNSHWHLARISHRPKLGLSNFNEYKFDPNGGQNIDAYILDTGVYQTHAYYIERTIRGIVITPNEDERDMNGHGTEMAGLVGGREVGVARKATLIDVKVLNWQGRGTVSNIIKGLSFVNHHHPQRIEKNGVLGAVVLFSISGAKSRSLNTAFDKMVSSGIHVVVGAGTKGINACNSSPASSSKPITVAGMSITDEPIDRGNWGSCVDIYGPGDRVRTTYPNLERNQFTNEISVTGSSAAAAQVAGLLAYFASLQPGLGTPDVIPLTPEELKERLIDFGTKGKLTNLERMSPNIIAFNGAGGNLDSFFNDGSEIVPTATKNVDPSDVPTETASPTGINTPVPPLPVDPENPEEPSNPEDPENPNDPTNPDDPADPPVDTPPRKLLYREKQQYFVNLKNFVDRVNRF*

>CIMG_09106.t1_gene_CIMG_09106_Cimm_RS_supercont2.5:join(553121..553429553485..554024554099..554204554261..554502)

MFNDTATISDIRVQLESLDKTISQFGRGSSYIESAVDIGNNGNGFLGYYGIFDDKVAALLEQDPRVDVYEDVKVTLPAYGRSKKMLKMASKYSGNLEEDNDFSFNSDSTVSDPLTEPRDFDIDFERLKSLYSDDGHYDYEVGVSYENLKVSQMQQHPPRPKPQASSRPRRQPQTRPQPKAQPKPPPKQQQQQQQQQPRPQQRRPQPGRPLPGRPQPPKPQPKPQQPQRSQQSTTIPAHMLTQEKTTQWGLHRISSQKNTVSPGVAKIIKPYEFMIPQYDTVAYVVDSGIRITHKDFGGRAVWGYNAIDNINEDMNGHGSHVAGTIGANKWGVSKRTLLVAVKAFGEEDCVSVGTYLHAVNWAINDYIAKGHDRAVINMSVGVPRGFEPFDRLVRHAIKVGMPVAIAAGNDGRDACNYSPSRMGNHPGAITAAASSKDDRLVRRFAGWMSRGSNFGECVTVFAPGMNIESVSHQSDTGTDIMSGTSMAAPHVAGLMAYFQSISDQLLTPMQLDYLITHANQGTMRGRLKNSPNRLIYNGL*

>CIMG_09616.t1_gene_CIMG_09616_Cimm_RS_supercont2.5:complement(join(1922662..19229031922966..19230711923122..19236791923731..1924048))

MKFTLLLAGLSLVAAAPADADASAYSNVLNIINKHQGHHAKRDLADSAKDRYLVFFKNGTSIQSINGHIQDLDSKVSPEVGAMAGTNGFAATSSNQGIQGAFDVDAFTDGKGFTGYFGSFNSSVLDSIRSSPDVASVEKDVLISLGKHAAQPVNLTELHKRDFQSTTVAQWGLSRISHLNNNVQGVNPDKPSTVRVPYNRESSKYDTTVYVVDTGTYVQHQDFGGRARWGANFVQGEPMQDLNGHGTHVSGTVAGSTVGVSGEKTSIVAVKVLNQKGQGYASTIIQGMNWVLSDYQKAGQPRSVINFSGGGASSPGLDQVFQQAVQQYGIPTVVAAGNNGQDACNESPARASKGIPGLITVGATDHLDNIAVEQDWSSAKGQCVEMFAPGVTILSLWITNDDAAATMGGTSMAAPHVSGAIAYFQSIADGVVPAAVLESWVKDASTGKVKGDLGGAANNFLWNRASF*

>CIMG_09744.t1_gene_CIMG_09744_Cimm_RS_supercont2.5:complement(join(2283975..22843402284406..22844942284545..22846412284706..22850592285109..2285423))

MKASTILAFFFGLTSAGVTRAPDGLEAHIDQLDSDIKSTNAAPLRRSSQNGPESSSAPKDRRMLAKRALKTQKSAPWGLRAVSHRRAGAFYEKFPPDPASKYYYDDRAGLGTYAYILDGGIRTTHGEFEGRAETVFTLYPGDEIDHRGHGTGVAGVLGSKTYGVAKRAKLLSVKTLDEEGGCTASAALRALSWTAEHILRNGRQHSSVINLSFGVKKVQSLNTFIEALISEADVPVVTAAGNENEDASISTPGSAKGVINVGHMNKHWVLSPKSNWGPSVTMLAPGVDVECPSSGSDTNVILQSGSSFAAPHVAGLVLNAISVHGIKGATKIKQFLLQSATRDQACTYHNTPNIVANNGNTVQKKHTKPRNC

>CIMG_10193.t1_gene_CIMG_10193_Cimm_RS_supercont2.6:complement(join(968993..969234969313..969418969516..970055970119..970433))

MKTSAILAFFFGLTSAGVTRGPEGLESHIAQLDSDIKGTNAAPLRRSLQNGPETSSAPGRHPMLVKRASKTQKGAPWGLRAISHRRAGAFYEKFPPDPASKYYYDDRAGLDTYAYILDSGIRTTHEEFEGRAETVFTVYPGDEIDHRGHGTAVAGVLGSKTYGVAKRAKLLSVKTLDDKGSCAASAALHALSWTAEHILSNGRQHSSVINLSFGIPKLQALDTFIEALVSQVGIPVVTAAGNENEDASLSTPGSAKGVINVGHMDKNWVMSPNSNWGPAVTMLAPGVQVECPSSASDTNVVLESGSSFAAPHVAGLVLNAISVHGIKGAAEIRKFLLQSATKDQACTSRNTPNIVANNGNTAQKKHTKPRNC

>CIMG_10287.t1_gene_CIMG_10287_Cimm_RS_supercont2.6:join(1212155..12124631212517..12130621213116..12132211213289..1213530)

MKTSAILAFFFGLTSAGVTRGPEGLESHIAQLDSDIKGTNAAPLRRSLQNGPETSSAPGRHPMLAKRASKTQKGAPWGLRAISHRCAGGFYEKFPPDPASKYYYDDRAGLDTYAYILDSGIRTTHEEFEGRAETVFTVYPGDEIDHRGHGTAVAGVLGSKTYGVAKRAKLLSVKTLDDNGSCAASAALHALSWTAEHILSNGRQHSSVINLSFGIPKLQALDTFIEALVSQVGIPVVTAAGNENEDASLSTPGSAKGVINVGHMDKNWVMSPNSNWGPAVTMLAPGVQVECPSSASDTNVVLESGSSFAAPHVAGLVLNAISVHGIKGAGEISKFLLQSATRDQACTSRNTPNIVANNGNTAQKKHTKPRNC

>CIMG_10288.t1_gene_CIMG_10288_Cimm_RS_supercont2.6:complement(join(1214218..12145801214636..12147241214786..12148821214939..12153071215373..1215678))

MNISAILTLLFGLASAHLAHTPVVLEPQINQHGSDFNKANGGLPEISLQRNQPEGSLETRNDTTLAKRNRAPKTQTSAPWGLRSISHRLPGAIYEGFPPSRNSEYYYDTNSGSGTFAYILDDGIRETHKEFEGRAKNIYSIFPEKQAGDYVHGTAVAGIIGSKTYGVAKKTTLLSVKTLGTTGADHSEVLKALLWTAEHIVNNTRQKSSVINLSFGVEKSDALNKFIELLVGKYDIPVVTAAGNEGEDASTKTPGSAKGAINVGYINKQWGLAPRSNWGPAVTILAPGVDVETTGSESDTNAVLQSGSSYAAPYISGLVLNAISVHGVKGAANIKKFLLEKATKDRACVSKSTPNLVANNGNAMQDKVKSDDKSALSKLMCCIKGSLNKCK

>CLUT_00086_gene_CLUG_00086_clus_supercont_1.1:complement(165320..166945)

MNISAILTLLFGLASAHLAHTPVVLEPQINQHGSDFNKANGGLPKISSQRNQPEGSLETRNDITLAKRNGAPKTQTSAPWGLRSISHRLPGAIYEGFPPSRNSEYYYDTNSGSGTFAYILDDGIRETHKEFEGRAKNIYSIFPEKQAGDYVHGTAVAGIIGSKTYGVAKKTTLLSVKTLGTTGADHSEVLKALLWTAEHIVNNTRQKSSVINLSFGVKKNDALNKFIELLVSKYDIPVVTAAGNEGEDASTKTPGSAKGAINVGYINKQWGLAPRSNWGPAVTILAPGVDVETTGSESDTNAVLQSGSSYAAPYISGLVLNAISVHGVKGAANIKKFLLEKATKDRACVSKGTPNLVANNGNAMQDKVKPGDKSALSKLMCCIKGSLNKCK

>CLUT_01869

RDYQYTPDNFIREHPARGLDDHYVFSRRINDPIFTEQWHLINTLSPGNDVNVKDVWYRGVRGRNVTVAVIDDGVDCDSEDLAANFNARGLWDFNDNTELPKPRLFDDYHGTRCAGEIAAVNEVCGIGVAWEAKVAGIRILSGTITAEDEAAAMVYGLDANDIYSCLWGPTDNGQTVAAPDVLVRKALIKGVQQGRDKKGAVYVFASGNGGRVGDQCNFDGYTNSIYSITVGAIDYQGQHPPYSEACSAVMVVTYSSGGRIHTTDICASTHGGTSAAAPLAAGLFALVLSANPALTWRDVQYVCAKAAVPDGEYQVTGLGYLHKYGYGKLDADKLVTVAQWKNVKPQAWYYSSTITVSEEELRGMNERVEHVTVKVNIASNVRGRIGARLVSPKGVSTLAQFRAVDTLHNGLSDWVFMSVAHLGEDGVGDWRLEVDWQLRIFGESVDASKARRFEFLFEDL

>CLUT_01871_gene_CLUG_01871_clus_supercont_1.2:complement(1319151..1320500)

MHISITLALFFGLTSARVAHAPVVESQIDQPESVINDNTLAPLELALGLKNHVKLAKRSLQTQTSAPWGLRAISHRSPGDFYEEFPPPESSKYYYDDKAGAGTFAYILDSGIRTTHEEFEGRAKAAHSIYPADQTIHGDHGTGVAGIIGSKTYGVAKKATLISIHLLGPDGCTGSEAINALLWAAEDILKNSRKDSSVINLSFGIPKLKALNTFVERLITDTDIPIVVAAGNEADDASNHSPGSADGVISVGHINQQWAISETSNFGSAVSILAPGVGVETTGAGSDTNIIRETGSSFATPYISGLILNAISIHGIKGAANLKRHILETATKDKACIPPEKEDKKNRTPNLVGNNNNAEQDKEKQKDEPSSSRMFCCNGLLSKLACGRNRPTNV

>CLUT_03659_gene_CLUG_03659_clus_supercont_1.4:complement(904907..906094)

MHISITLALFFGLTSARVAHAPVVESQIDQPESVINDNTLAPLELALGLKNHVKLAKRSLQTQTSAAWGLRAISHRSPGDFYEGFPPPESSKYYYDDKAGAGTFAYILDDGIRTTHEEFEGRAKFAHSIYPADQTIYGDHGTGVAGIIGSKTYGVAKKATLISIHLLGPDGCTGSEAINALLWAAEDILKNSRKDSSVINLSFGIPKLQALNTFVERLITDTDIPIVVAAGNEADDASNHSPGSADGVISVGHINQQWAISETSNFGSAVSILAPGVGVETTGAGSDTNIVRQTGSSYAAPYISGLILNAISTHGIKGAINLKKHILETATKDKACIPPEKEDKKNRTPNLVGNNNNAEQDKEKQKDEPSSSRMFCCNGLLSKLACGRNRPTNE

>cneo_00150T0

YIVFFGRAVHSPSADAVQGNKGKGIKIGIIDGGDYTREPLGGCFGGCKIAGGYDFVPDPYDNCHGTFISGIIGGVAPEASLYVYRVFGCDIVLAMQKAYDDDMDVINLSLGSVFASRVVTVVTVSAGNGQVGFYSSSPAAGKGVINVGSSDSDIYVIVRGTSYFSEIGPTLYFAPSVLAPGTVVGVMPGTSYSSAAGAAALAQQVSVAVQGAGLSNAAVISNDTIKNGNKWVTYKLSHEPALWPGFPIYSGFIPYMGPNGDYQ

>cneo_grubii:CNAG_04625T0_gene_cneo_grubii:CNAG_04625T0_cneoH99_Chr10:join(cneoH99_Chr10:848265..848468cneoH99_Chr10:848589..848694cneoH99_Chr10:848795..849302cneoH99_Chr10:849355..849540cneoH99_Chr10)

MRFFTVFSTFLLTASALPAPHNGEHPPTYTIPGDNKPTVPSGHPTKFTPYLFRFPHPKDGSAVVNAEEILTSLQPHGFNKSHLHHKFSSIMNGFSADMSDHCVKILKAMLPPSGVIIEPIVTYRVQAPFATPQLADIGNFAAEHPVKKPRQAPAPGITNVQTQTNAPWGLQRISQKQMIPVDFNAAADKTWQPSFKYKFDETAGEGVDIYVIDTGININHKSFGGRADRVYTAPSLKSNTLEGEEDRAGHGTHCAGTTGSRGFGVAKKANIHAIKVMGSKGAGSSADIIAGIDEMLTIHRRRKDEPGFKGSVASMSFGIDVDNIDGNEVSASPALERAIMRANAEGIHTVIAAGNQGIDACRVSPGFLSNKLYNGTEFTYLASAITVGAIDIHDNRADFSNYGPCITTYAPGRDILSTYINGDEAVNVMSGTSMAAPHVAGLVAYFLGEDQSLQTDVVGMKKKIMNTAGKGLLHNIKDDKDTKILAYNGV

>cneo_grubii_CNAG_05446T0

RSYTYVASKSLGVERIGELDGHWLVRKRLADPMLDQQWHLINTQMKDIELNVTGLWGRGVTGEGVHVVIIDDGLDVESKDLKDNFFAEGSYDFNDHTALPIPRLRDDQHGTRCAGEIAAVNDVCGVGVAYGSKIAGVRILSAPISDADEAAALNYAYQLNDIYSCSWGPPDDGRSMEAPDGLILKAMVNGVQKGRDGKGSVFVFAAGNGGGSDDQCNFDGYTNSIFSVTVGAVDRKGLHPYYSEMCAAMMVVAPSSGSGIHTTDVCAHNHGGTSAAAPLAVGVFALALSVRPDLTWRDIQHLAVRHAVFDPAWELTAAGFSYKYGYGKLDAGLFVEAAEWELVKPQTWYDSSTYEVTQSMLYDANERLEHVTVRVWIDHQRRGDVEVELISPNGVSVLCRQRRFDDANSGFPGWKFMSLKHWDENPVGTWVIKVKDAWSLQLWGESVDPALAARRYELYDAF

>CPAG_01624.1_|_CPAG_01624_|_Candida_parapsilosis_CPAG_01624_(463_aa)

IQQQPKAPWNIARLSSNASIAKPSTYFFNSTAGRGVDIYVLDTGVFISHLQFAGRAKVGGNIGLGIVDGNGHGTFCASVAAGTTYGSAKKANIISIKTFTDSGGGTASSVIKGVALVFKLAKKSKRPSIISLSASFPANSALDAIVKKATKKGIHFVSAAGNAGADAGSYSPGRSPYSVTVGAVDATDTVAKFSNTGVVVSLFAGGVNVLGAFIGNTAASATLSGTSMSTPAVAGLIAYFISTWGNAPPATMAAALVKAATPNTVKGLRAGTVNLVAGN

>CPAG_01838.1_|_CPAG_01838_|_Candida_parapsilosis_CPAG_01838_(475_aa)

MSGEIIPGEWLVTLKPYANESIDSEHASLLSTRTADPDTHFNCDVQCHFALPELRGYSAKFDDATRAEVEALPEVQAVEPLQVYRHCAAAGATAVQSNAPWGLARISQRGRVAPAGPWEYKYDANAGAGTVAYILDTGIRDTHEEFEGRASKGPTFSQGRPASDEDRDGHGTHVAGTIGGKTYGVAKKAELVGVKVFNDDPQPGATNADIIRALEWVVDQVKSHGKPSVVNMSLGGGASAALDAAVASTVRVGIVVVVAAGNDGRLADRGSPAREPLAITVGASDVKDAGAIFTSSGKVVDIFAPGVDIKSSWNTGDDAVESLDGTSMASPHVAGAVCYLLSQKRVEPLLVMPQLLGWADKNKLTGLKDRTIDALLVVSDN*

>CPAG_01839.1_|_CPAG_01839_|_Candida_parapsilosis_CPAG_01839_(409_aa)

MATQHFMTTGQTTVPGQWVVRVKPYLTPELVQKEHLSLLEEKTEDPATPFNVEILQRFDLYDSKGYSAKFDDATKEELEKIPHVVSIEPEQLYRHCNIQPNSPWGISRVSTRTKLGAPPYSYTYRDDVAGSGTVAYVIDTGINNKHVEFEGRAQKGPKFVSDNVSNDEDVHGHGTHCAGTIASRAYGVAKKANVVGVKVFGDRTGTAQTSDIIKALEWVISDISAKGMGGRAVVNLSLGGPPSDALDAAVASTVHKGVVVCDIQESPAREPLAITVGATDIKDQLANFSSYGKFVDILAPGVDILSCWTGGPTSTKTISGTSMATPHVGGVACCLLSDPTLAGGQATTYDVMSKILILADKNKITGTDARTVNALLHNTTSPMDA*

>CPAG_02963.1

RDYNYSQLDFISEHQLPSLENYYVFSKRIHDPEFASQWHLFNLQYPGHDVNATGLWLEDILGQGIVTAIVDDGLDAESADLKANFNAKGSWDFNDNGPLPLPRLSDDYHGTRCAGEIAAVNDVCGVGVAYKSQVSGVRILSGPITSAEEASALVYGLETNDIYSCSWGPTDNGRTLSEPEIIVKKAMLRGIQEGREGKGAIYVFASGNGGRFSDSCNFDGYTNSIYTITVGAIDHKGMHPLYSEACSAVMVVTYSSGSGIHTTDICSARHGGTSAAAPLASGIFSLILGANPDLTWRDLQYINVLSATPDGNYQTTALNYSHMYGYGKIDAYKMVEFAKWKNVKPQSWHYCSKVTVTEEDLKVMNEKIEHVTVKANIDSSFRGRTGVRLVSPSGVSDLAKFRPLDFSSRGFQDWTFTSIAHWGEDGLGEWTLEVNWQLRFFGTTIDASKARRLYEDF

>CPAG_03446.1_|_CPAG_03446_|_Candida_parapsilosis_CPAG_03446_(446_aa)

MTTGQTTVPGQWVVRVKPYLTPELVQKEHLSLLAEKTEDPATPFNVDILQRFDLNDSKGYSAKFDDATKEELERIPHVISIEPEQLYRHCNIQPNSPWGISRVSTRTKLGAPPYSYTYRDDVAGSGTVAYVIDTGINDKHVEFEGRATKGPKFVSDNVSNDEDVHGHGTHCAGTIASRAYGVAKKANVVGVKVFGDRTGTAQTSDIIKALEWVVSDVSVKGMGGRAVVNMSLGGPPSDALDAAVASTVRKGVVVCVAAGNDPEDVNESPAREPLAITVGATDVKDQLANFSSYGKFVDILAPGVDILSCWRGGPTSTKTISGTSMATPHVGGVACCLLSDPSLARGEVTTYDVMSKILILADKNKIAGADARTVNALLHNTTGPMDA*

>CPAT_00038_|_CPAG_00038_|_Coccidioides_posadasii__RMSCC_3488_oryzin_(400_aa)

MRIPGSHILTFLSFLSVALGTPTIGRRAIERYEGETTDRLIFTLKPGVSKADLFNRLRIRNLDQVVTHQWSHALNGFAAKFSKNDPILDALHSSPEVESISEDGIFSINDLVTQTNATWGLGRLSSVDKVPSFGTGATNYSYIYEDAAGRGTDIYVLDTGIRATHEDFGGRARFAATFGPYRAVDGHGHGTHVAAIAAGSQFGTAKLANVLGVKVLNDTGSGTLADIVSGLNYVLTSARSSRRPSIASLSFGGGATSALDNAVVSLINSGVHVVVAAGNSNTNASNVSPARVPAAITVGAVDITDTKTATSNWGPAVDVFAPGQNIRSAWASNDSATEIASGTSMAAPYVAGLIGYFISFAGNTNPAEMQARVKDWSLVGVLKGLPQGTPNNLVTNSYVCNLVEMMICI*

>CPAT_00674

LAEPDGWLKVSVAKDPNLLHRSLSTPNHPRYGQHMVRDIVAPHPEASDGIMAWLDWIDFVDTAERLLNTRFYHFRALEYSLPSSLSKHVRTVQPTTFFTPSSLRELYNLLGVSGYLEQYARYSDLNAFIFSVELYGGNDQEASLDIQYTVGLTYNTYYSGGGRNEPMEQLKFFANLSDSRLPTVLSTSYGENEQVPYAKAVCDEFAKLGARGVSVIFSSGDSGVGCLTNDGFNPIFPAACPYVTSVGGESASSGGFSEIFPRPSYQSVDAFLYFNRNGRGFPDVAAQGYAVYDHGVGGTSASAPLIASVISNLNEARLSQGKPVLGFLNPWLYFTDIVDGGCAWDAVEGWDPVTGFGTPFKKL

>CPAT_01068_|_CPAG_01066_|_Coccidioides_posadasii__RMSCC_3488_alkaline_proteinase_(401_aa)

MRFFSVIAAGLALFAATPALAGPTTLKSVERYQGETSGRYIVKFKPGVSRRNYINRLKIKAAADWDILNGVAASLDEDTLNTLRASDDVEYITEDGIMTIQATQSNAPWGLGRLNQAGRLANQATGSLAFNFTYDDSAGAGVDIYVLDTGVRVTHSQFGGRARWGTSFVGASTDGHGHGTHCAGTAAGSQFGVAKRANIIAVQVLNSSGSGATSGIVSGLNWVLTQARNSGRPSVVSMSLGGSASTALDNAVAQLTSAGVHVVVAAGNSNTNAANTSPARAPSAITVAASTIADAKASYSNYGAIVDVWAPGSNVISAWIGSDSATNSISGTSMATPHVAGLVAYLIGRDGNVSPAAMATKIINSSVNGALSGVPSGTVNRLVQIR*

>CPAT_01456_|_CPAG_01454_|_Coccidioides_posadasii__RMSCC_3488_alkaline_proteinase_(399_aa)

MKFFSVLAAGLAVIMAVPTLAAPAPAPAPPGRRLKSIERFQGATTGKYIVKFKKGASRRNYLNKLKLKASADWDILNGFAADLDEAALEELRASDDVEYVAEDGIMHAFVTQTNAPWGLQRITQPGRLASQSTSSANYNFTYDASAGAGVDIYVVDTGVQTSHSDFGGRARWGVSYVSESTDGNGHGTHCAGTAAGTRFGVAKRANIIAVQVLNRSGSGATSGIVSGLQWVLNQARASGRPSVVSMSLGGGASTATDNAVAQLTAAGIHVVVAAGNSNVNAANTSPARAPSAITVAASTIADAKASYSNYGAVIDVWAPGSNILSAWIGSNTATNNISGTSMATPHVAGVVAYLIGRDGNISPAAMATKIKNLAVKNVLSGVPSGTTNALLQI*

>CPAT_02260

RDYDYSPAQLLGEGRIGELQDHHTFSKRISDPIFVDQWHLFNTEQPGHDLNVTGLWLEGITGNGTVTAIVDDGLDMYSHDLKDNYFAEGSYDFNDKGKEPRPRLVDDKHGTRCAGEVAAVNDVCGVGVAYNGKVAGIRILSKPVTDEDEAAAINYGFQKNQIYSCSWGPVDNGATMDAPGLLIRRAMVHGIQQGRGGKGSIFVFAAGNGAASGDNCNFDGYTNSIYSITVGAIDREDKHPYYSESCSAQLVVTYSSGGTISTTDVCSNRHGGTSAAGPLVVGVVALALDVRPDLTWRDIQYLIVETAIPEPGWQTTAIGFSHDFGYGKVDAYSLVQLAKWELVKPQAWLHSSSFEITEELLKKNNERVEHVTVTMNVNHTRRGDLSVELKSPSGISYLSTTRSGDFEKKGYVDWTFMSVAHWGETGKGKWTVIVKDDWQLSLWGEAIDGKIQQKRRRYEFLYDAF

>CPAT_02756

LLEPDGWMKINLASQTEDLHQKVGTPGHARYGLHLINSLMTPNEVVLNDVLKWINWIDIVGAASKMLNARFYEYRTTEYFAPKSVAQHIFYIYPLILFTPNCLRGLYNKIAVSGYLDQYAQYKDLAAFLFSVSLVGGNIQEANLDTQYAVALTYNDFVSVKGRNEPMDQLEYLMGLPDKDLPTVLTTSYGETEQVPYARATCNEFAKLTARGVSIIFSSGDTGVGCTSNDGFNPIFPASCPFVTAVGGERASAGGFSNYFKRPGWQAVTKYLYYNPLGRGFPDVAAQAYPIYEKGAAGTSASAPTIAAIIAHLNEVRLSQGKPVLGFLNPWIYFTDITHEGCSWNATKGWDPVTGFGTPFKKL

>CPAT_02986_|_CPAG_02970_|_Coccidioides_posadasii__RMSCC_3488_oryzin_(407_aa)

MRLSTIFTTFAVLLATPVLSAPGASLKSVERYAGPTSGKHIVKLKDGVSRNQWMRKLKLPSDTVELNLINGFSGTLNDETLAALQASEDVEFISEDGIVQMHSPVTQNDASWGLQRISQEQALDDTDFLKLDFKYTYESSAAGSGVDIYLTDTGILTTHTDFGGRARWGGSFGVGGDRDGHGHGTHCAGIAAGARWGVAKNASLIAVRVLSNSGQGPVSGIVAGLDWIRSQAAASGRPSVVSMSLGGSVSQALDDAVTRLTDAGIHVTVSAGNDNKDAFNVSPARVPSAITVGAANIFDAKAAFSNFGPVVTVYAPGQNVTSAWIGADNASSNRVSGTSMAAPHIAGLVAYLISKDGNISPAQMQAKIQNLAVKGAISGLPSGTANNLAQIGPL*

>CPAT_03100_|_CPAG_03084_|_Coccidioides_posadasii__RMSCC_3488_subtilasetype_proteinase_psp3_(408_aa)

MRFFAAFAAATVLLAEPVLSAPGPSLKSVERFPGQTTGKYIVKLRPGVSRKQWIRKLRLAANTVNWGLINGFAGILNDQALNTLRESEDVEYITEDGIMYTMSPVTQSDAPWGLQRVSQAARLSNTDVAALDYQYTYDSSAGSGVDIYIADTGILVSHSQFGGRARWGGTFGVTGTTDGHGHGTHCAGTAAGAQFGVAKNASLIAVKVLTDGGSGSIAGIVSGLDWIRTQVAASGRPSVVSMSLGGSASTALDNAVASLTAAGIHVTVAAGNDNRDAANTSPARTPSAITVGATDIQDGKASFSNFGAAVDVFAPGQNVISAWIGASNSATNSISGTSMATPHVAGLVAYLISKDGNISPAAMEAKIKDLSVKGAVTGLPSTTANNLAQIGPL*

>CPAT_03870_|_CPAG_03850_|_Coccidioides_posadasii__RMSCC_3488_kp43_peptidase._serine_peptidase._merops_family_s08a_(474_aa)

MARYLANTGSQLQIFISVSKAMVCLRRVAANRGQLMPRLARPLFERRYLPYSDIELTPPDEQEDQLETLGAVVHEYVSHNTYLCGYKGSDLAQIRALEFVVWADVYLNTFVIQPTLKSAAPTTQTFQVHKVDVIFHHDVDTHAENLRIALATAAHVDADDLDIGPHKVRLAVQEQYLEGLAAIDDVVAVADIGFDKGSTQDVHEAFTGRVKKLYPLGKSTSGTTGHPDGHGTQVCSSVLGDGNPKSMGGRIRGVAAKSTLVVQSLLDGRGNLGGIPDDLTQLFIQPYNEDNARIHTNYWGSTSGRQLPYDASSSEIDRFVWEHPDIVVLFAAGNDGIDIDRNGVIDDRLSSSCKELHHRRRKREPPPEHSYQVWHNDLMANHPDGVAAFSSRGLTKEGRIKLYAVAPGTGILSTRSHDLLNPGERFGHSDDPNYWFLAGTSMATPLVAGAVAVLRECLTLCAGLEHQQTSSTG*

>CPAT_04063_|_CPAG_04042_|_Coccidioides_posadasii__RMSCC_3488_subtilasetype_proteinase_psp3_(498_aa)

MKVNARKCLVTAALLVFGVRAAEIIPVSNGMEAIPDSYLVVMKDDIQAREFDTHRSWVDGLLHDNAAGANDAGKGWGVKYIEQDAMIRALGLVTQKGAGWYLSRISHKKLPPTPEYVYEERAGQNVHIYLLDTGVDGTNSGLRGRVIQGINTTPDGDSDENGHGTSVAEVIAGTVHGVAKKALVVSVKILDNTGSGSISGIIKGLDWSISDVAKRNIVGKAVMNMSLGGAYSASFNQATEKVIKAGIFVAASVGAGNVDYPPLFFSHSSNFWFQRDASQESPVSANGVCAVAASTPADAPASFGSYGPVVDIYAPGTNITTRIHGDKLVTVSGSSYSTSQVSGMATFIIDMGMSSDKVCETIKKLALPSIQDPKPGTTKLLLYNGSGL*

>CPAT_04414_|_CPAG_04392_|_Coccidioides_posadasii__RMSCC_3488_oryzin_(400_aa)

MKVNDKACLATVALFFLGARAAKIITAANGIEAIPDSYLVVMKAEVQAGEFNTHRQWVESLLDDPVSIANDSGKAWGVHHTFNVAGLQGYSGTFDKQTIQTIAKHPTVKYVEQDAMVKALGLVVQKNSGWHLSRISHKKLPPIPEYVYEERAGQNVHIYLLDTGVDGTNNGLRGRVIQGINTTPDGDGDGNGHGTSVAEIIAGTVHGVAKKALVVSVKILDNNGSGSISGIIKGLDWSISDVTRRSIGGKAVMNMSFGGSYSATMNQATENVIKAGIFVAASVGAGNRDASQESPVSANGVCAVAASSLADAPASFSSYGSIVDVYAPGVNITTRLNGDRPVTVSGSSYSVAQVSGMAAFVIDMGVVLGKTCDTIKDLAHPSIQNPKPGTTRLLLYNGSGL*

>CPAT_07341_|_CPAG_07294_|_Coccidioides_posadasii__RMSCC_3488_oryzin_(398_aa)

MKVNDKACLATVALFFLGARAAKIITAANGIEAIPDSYLVVMKAEVQAGEFNTHRQWVESLLDDPVSIANDSGKAWGVHHTFNVAGLQGYSGTFDKQTIQTIAKHPTVKYVEQDAMVKALGLVVQKNSGWHLSRISHKKLPPIPEYVYEERAGQNVHIYLLDTGVDGTNNGLRGRVIQGINTTPDGDGDGNGHGTSVAEIIAGTVHGVAKKALVVSVKILDNNGSGSISGIIKGLDWSISDVTRRSIGGKAVMNMSFGGSYSATMNQATENVIKAGIFVAASVGAGNRDASQESPVSANGVYAVAASSLADAPASFSSYGSIVDVYAPGVNITTRLNGDRPVTVSGSSYSVVQVSGMAAFVIDMGVVSGKTCDTIKDLALPSIQSPKPGTTRLLLYNGSGL*

>CPAT_07765_|_CPAG_07718_|_Coccidioides_posadasii__RMSCC_3488_oryzin_(401_aa)

MLFLKAVIAILSVLPAADAAAILNFENKQGIIPDSYIVVLKNDISSDDFKSHVAWATGVHNANVAKRDVPLAGMQRTFEMDIFKGYSGAFDRATLDDLLKNEQVDYIEPDRMASAQGWTTQGNAPSWGLGRISHQQRGNTDYVFDSTAGRGITIYGVDSGIDILHAEFGGRATWGANFFNNINTDEFGHGTHTAATFGGTNYGVAKNVNIVAVKVLGDQGQGPWSSIIDGLQWAVNDAREKGILGKAIINFSVGGPSSRAADNALTAAHNAGVFVSAAAGNDGADALNYTPGTARSICVIGNINENDYRFTGNGASNWGTRIDLWAPGTDILSALPQGRYGPMTGTSMAAPHVAGSVAILMASGGVSTAEACGVLKDMSTPSVIEPGQGSTNRLLYNGSGQ*

>CPAT_07875_|_CPAG_07828_|_Coccidioides_posadasii__RMSCC_3488_oryzin_(401_aa)

MLKQFVHSFQPCFSSKALSPSPSFRYFLSSAVYAAPFSKPDSSSDVIPDSYIVVLKKDVSSDSFDSHIKWANNVHKRNVAKRGLSLEGMKHTWAMGEFKAYSGAFSSETIDDIKKHEHVAHVEKDRYATAQGWSTQENPPSWGLSRVSDPNPGNHEYTYDGNGGSGVTVFVIDSGIYIEHEEFEGRATWGANFLDGDNRDLYGHGTHVAGTIGSVSYGVAKKVNLVAVKCLDGRGKGPWSAIIAAIHWTVDEARKRGILGKTVINFSLGGEPSPAVDAALVEAHKAGIFISAAAGNFGSDARSVTPGSASLVCVVGNSDENNYRWTGKDPSNWGPRVDIFAPGTRIMSTLPNGGFGPSTGTSMAAPHVAGQVAIYISHGNFDLSAACEFLKQKATPTVQDPGQDTTNRLLSNGSGKGGPGPNPDQKKPDQNKPDQNKPDQNKPDQNKPDQNKPDQNKPPGQNNPQGSGEPENSPPTPSPPQGPNNWGPSWWDRPSPGWLNRPNLGWWNRPYSGWKP*

>CPAT_07876_|_CPAG_07829_|_Coccidioides_posadasii__RMSCC_3488_oryzin_(405_aa)

MFFFKGVLAVLSFFSAVNAAPLIKPNNVSAKYIEDSYIVLLKRDISHDDFELHKRWASDVHKRDVAKRGVSFSGIGHSWATGSFRGYSGVFSRDTIEEIMKHEHVAHVERDQIGTSQGWVTQSNAPNWGLGRLSNSNPGNADYTYDEGAGGNAVVYVVDSGIDVMHPEFEGRATWGANFIDNNNVDCWNHGTHCAGIVGSVSFGVAKRTAMIAVKVLDCNGQGPYSAFIAGLHWTIDHAQNNGFVGRAIINFSLGGDNSPAVNAALEEAQKAGIFVSAAAGNQGTDAGRITPGGAGLVCVIGNSDGSDYRWTGQGPSNFGPRGESGLPSASLGVMTGTSMSAPHVAGQAAIQVSISGGGFDLKVACAFFKKAASASVKNPGPNTTNRLLVNGASGTKDPKQGENQPNKPPGQDEQPGQNKPPSQNPPPSQNPPPGQNKPPGQNKPPSQNPPPGQNPPPEQPAPSPPVNPGGEPNPGGQPNPGGQPYPGDQTNPGNSGPSWWMPSGGLNPPAWWNRRPSFGGWNNRPMWWNRPLSVWKL*

>CPAT_08077_|_CPAG_08024_|_Coccidioides_posadasii__RMSCC_3488_oryzin_(405_aa)

MFFFKGVVAVLSFFSAVNAAPLMKPNNGTGKYIEDSYIVVLKRDISHDDYELHKRWAHDVHKRDVAKRGVSYSGIGHSWASGSSRGYSGVFSRDTIEEIMKHEHVAHVERDQIGTSQGWVTQENAPNWGLGRLSSHNPGGRDYTYDESAGGNAVVYIIDSGIDVKHPEFEGRATWGANFIDKNDVDCWSHGTHCAGIVGSLTFGVAKRAAMVAVKVLDCGGQGPYSAFVAGLHWSMEDAKNRGLIGRAIINFSLGGSNSPAVNAALEEAQRAGIFVAAAAGNFGSDAGSITPGGAGLICIVGNSDNRDYRWTGQGPSNFGPRVDIFAPGTDILSTIPGGGTGLMTGTSMASPHVAGQAAVQVSMSGFDLKAACYFFKNGATASVHNPGPNTTNKLLVNGANGNKGSEQSPNKPPGQDEQPGQNNPPGQNKPPGQNQPPKQPAPSPPGNPGGEPNPGGQPYPKDQPNPGDSGPSWWLPPSMSPPAWWNRRPSFETWNHRPMWWNKPLSVWKL*

>CPAT_09432_|_CPAG_09367_|_Coccidioides_posadasii__RMSCC_3488_oryzin_(395_aa)

MFFFKGVVAVLSFFSAVNAAPLMKPNNGTGKYIEDSYIVVLKRDISHDDYELHKRWAHDVHKRDVAKRGVSYSGIGHSWASGSSRGYSGVFSRDTIEEIMKHEHVAHVERDQIGTSQGWVTQENAPNWGLGRLSSHNPGGRDYTYDESAGGNAVVYIIDSGIDVKHPEFEGRATWGANFIDKNDVDCWSHGTHCAGIVGSLTFGVAKRAAMVAVKVLDCGGQGPYSAFVAGLHWSMEDAKNRGLIGRAIINFSLGGSNSPAVNAALEEAQRAGIFVAAAAGNFGSDAGSITPGGAGLICIVGNSDNRDYRWTGQGPSNFGPRVDIFAPGTDILSTIPGGGTGLMTGTSMASPHVAGQAAVQVSMSGFDLKAACYFFKNGATASVHNPGPNTTNKLLVNGANGNKGSEQSPNKPPGQDEQPGQNKPPGQNQPPKQPAPSPPGNPGGEPNPGGQPYPKDQPNPGDSGPSWWLPPSMSPPAWWNRRPSFGTWNHRPMWWNKPLSVWKL*

>CPAT_09576

VVEPESWLHLAMNDVLDQFEQRVSTPGNEHYGEHMVQAFLQPPSYTSDAVLAWLDWLHFVKKAEELFNTQFYYYRTLEYSVPKIIAPYVHMIQPTTKFTPDCLRDLYNKLGISGYLEQFARYDDFARFLFDVVSIGGNDQEASLDVDYAIGLSGVYYTTAGRNEPLDQLHYLLSLPDDQLPSVLSTSYGENEQVPYTDMTCNLFARLGARGVSVIFSSGDTGVGCQTNDGFLPVFPAACPFVTSVGAERASSGGFSDRYRRPWYQAVGHYLLYNPAGRGFPDVAAQGFSVVDHDVSGTSASAPVFAAIVANLNSIRQEKGKPVLGFLNPFLYFTDIVHGGCSWNATKGWDPVTGLGTPFEVL

>CPAT_09674_|_CPAG_09609_|_Coccidioides_posadasii__RMSCC_3488_oryzin_(411_aa)

MGLIKRLLLVSLSLLSAVNAAEILSLGNSEDVIADSYIVVMKDGLSQTAFDTHKTEVSSISKRKRDATAVLKHSFDFTGFRGYSGTFDEATIREIATNPAVKYIEHDKVAKAHGLIEQKGAGWNLARISHRQPGATSYVYDESAGQGISVCLVDTGVNVRIPELGGRAIWGVNLIDREDTDGNGHGTFLASLIAGTRHGVAKKAEIIAVKVLNASGSGSTSTIIAGIYWCIQNANDRGALNSTLINLSLGGSYSRGLNQAAEAAVRAGLFVSAAVGGSNIDSGNESPASAQGVCAIAASTMDDRPALFSNYGKNVALYAPGQNIMAISNNGGTVTLSGTSFAAGHAAGVAAYLQRLEGIPGNTICNRLKQLGNPVIRNPHSGSTRLLLYNGSGR*

>CTRT_01009_gene_CTRG_01009_ctro_supercont_3.1:2304234..2305451

MRAIISVALFLSLSLLSAVNAAEILSAGDTDDVIPDSYIVVMRDGLSTDAFNSHTTQISGFRNGDRNVKASLKKTFDLNGLKGYSGTFDEATIRQIANDPAVKYIEHDRIANARGLVEQQDAGWNLARISHKKPGARTYVYDESAGAGISVCLVDTGVDVDNPDLGGRATWGANFVDNDDSDGNGHGTFLASLIAGQKHGVAKKAKIIAVKVLDANGSGSYSNVISGIDWCVKYAKEHGISERMVVNLSLGGGYSQAVNQAAENAVLAGMFVSAAVGGSNRDARNDSPASARGVCAIAASTMDDKAALFSNYGSIVAVYAPGQNIMAAGRMGSVTLSGTSFAAGHASGVGAYLLALEKITGDRVCTRIKELAIPVIRNSPSNTTRLLLYNGSGR*

>CTRT_01010_gene_CTRG_01010_ctro_supercont_3.1:2305773..2306954

MRAIISVALFLSLSLLSAVNAAEILSAGDTDDVIPDSYIVVMRDGLSTDAFNSHTTQISGFRNEDRNVKASLKKTFDLNGLKGYSGTFDEATIRQIANDPAVKYIEHDRIANARGLVEQQDAGWNLARISHKKTGARTYVYDESAGAGISVCLVDTGVDVDNPDLGGRATWGANFVDDDDSDGNGHGTFLASLIAGQKHGVAKKAKIIAVKVLDANGSGSYSNVISGIDWCVKYAKEHGISERMVVNLSLGGGYSQAVNQAAENAVLAGMFVSAAVGGSNRDARNDSPASARGVCAIAASTMDDKAALFSNYGSIVAVYAPGQNIMAAGRMGSVTLSGTSFAAGHASGVGAYLLALEKITGDRVCTRIKELAIPVIRNSPSNTTRLLLYNGSGR*

>CTRT_01885_gene_CTRG_01885_ctro_supercont_3.2:1729783..1731192

MSIFKLMVIYFTLFWVVNAAQLLDLDSHGVIPGAYIVVMKNGVSSHQFSSHVRWLKRAHRRNLAKRVAPFTEGLSSTWDIAGWQAYSGSFDKDTIQEILNHENVEFVEPNREMKAASTIKQENITWGLARISHMENFSHDYVSTYGEGENLTFYGIDSGIDIHQSDFTGRARWGINVADHIDIDCIGHGTHTAGTVAGQSFGILKKASIVSVKVLDCYGHGDTTKYINGLNWAINDAKKRGLLGKSVMNISLGTGRSRAVNEATVRAQEAGIFISVAAGNNAINAEFLSPGSAPELCTVAASTRNDTRAYFSNYGALIDLFAPGEYIRSTLPHNRTGIMSGTSMAAPHVCGIGGLIMAAEGLAPEQVCRRLKELANPAIKYAGFNTTDKLLYNGSGA*

>CTRT_03249

RDYNYSQIDFINEHQLPSLDNYYVFSKRIHDPEFAAQWHLINLKYPGHDVNATGLWLEDILGQGIVTALVDDGVDAESEDIKDNFNADGSWDFNNNGKSPLPRLFDDYHGTRCAGEIAAVNDVCGIGVAWKSQVSGIRILSGPITSADEASAMIYGLDHNDIYSCSWGPTDNGRVLSEPEVIVKKAMIKGIQEGRDKKGALYVFASGNGGRFGDSCNFDGYTNSIYSITVGAIDHKGLHPEYSEACSAVMVVTYSSGSNIHTTDICSATHGGTSAAAPLASGIYSLVLSANPDLTWRDVQYISVLSATPDGNYQVTALNYSHKYGYGKTDAYQMVHFAKWKNVKPQAWYYSSSITVTEKDLKVMNERVEHITVKVNIEANYRGRVGMRIISPTGISDLAAFRRSDASGKGFQNWTFMSVAHWGESGLGEWKVEVDWQFRIFGESIDADKARRYEFLFDEF

>CTRT_05182_gene_CTRG_05182_ctro_supercont_3.7:814568..816025

MSIFKIILIYFAIFWAVNAAQLLDIDSRGVIPGAYIVVLKDRVSSLEFSSHVRWLKRTHRRNLAKRGTPFTEGLSATWDIAGWQAYSGSFDEDTVQEILNHENVEFVEPNKEMQVASTIKQGNVTWGLARISHKENFSHDYVSTYGEGENITFYGIDSGIDINQADFTGHARWGINLADNVDTDCYGHGTHTAGTVAGQKFGILKKASIVSIKILDCHGYGDITRYINGLNWAINDAKERGLGKSVMNISLKTRRSRAVNEATVRAQEAGIFIAVAAGNQATNAEFYSPGSAPEVCTVGASTRNDTKAIFSNYGELVDLFAPGEYIRSTLPHNLTGLMSGTSMATPHVCGVGGLIMATEGLAPEKVCGRLKELANPTIQNPGFNTTNKLLYNGSGA*

>DEHA0B05654g.t1_gene_DEHA0B05654g_Dhan_CBS767_chrB:466411..467613

MSIFKMMLIYFTIFWAVHAAQLLDIDSQGVIPGAYIVVMKNRVSSLEFSSHVRWLKRTHRRNLAKRGTQFTEGLGTTWEIAGWQAYSGSFDEDTVQEILNHENVEFVEPNKEMQVASTIKQGNVTWGLARISHKENFSHDYVSTYGEGENITFYGIDSGIDINQADFTGRARWGINLADHIDTDCNGHGTHTAGTVAGQKFGILKKASIVSIKILDCYGHGDITRYINGLNWAINDAKERGLLGKSVMNISLKTGRSRAVNEATVRAQEAGIFIAVAAGNQATNTEFYSPGSAPEVCTVGASTRNDTRAIFSNYGELVDLFAPGEYIRSTLPHNFTGLMSGTSMATPHVCGVGGLIMATEGLAPEKVCDRLKELANPIIQHPGFNTTNKLLYNGSGA*

>DEHA0C11308g.t1

RDYNYSSYKFIDEHQVRGLDDHFVFSKRISDPIFQKQWHLVNTFYPGHDVNVTGLWYEGNTGKGIVTAVVDDGLDYESEDLHDNFNSLGSWDFNDNTNLPKPRLFDDYHGTRCAGEIGAVNDVCGVGVAYDSQISGIRILSGTISAEEEASAMMYGLDVNDIYSCSWGPTDDGKTLSQPDAIVKKAMIKGIQTGRKDKGAVYVFASGNGGRYADSCNFDGYTNSIYSITVGAIDYKGLHPMYAEACSAVMVVTYSSGSGIHTTDICSALHGGTSAAAPLAAGIYSLVLHANPNLTWRDVQYVSALSSVPDGNYQITALGYSHKYGYGKIDAYAMAHFAEWKNVKPQAWYYSKKIKITKEDLKIVNERVEHITVTVNIQATERGKVGVRLISPHKTSDLATFRPQDRSGAGFKDWTFMSVAHWGESGIGEWAIEVDWQLRLFGESIDPEKARRYEFLFDEF

>DEHA0C14916g.t1_gene_DEHA0C14916g_Dhan_CBS767_chrC:1221169..1222812

MSIFKMMLIYFTIFWAVHAAQLLDIDSQGVIPGAYIVVMKNRVSSLEFSSHVRWLKRTHRRNLAKRGTQFTEGLGTTWEIAGWQAYSGSFDEDTVQEILNHENVEFVEPNKEMQVASTIKQGNVTWGLARISHKENFSHDYVSTYGEGENITFYGIDSGIDINQADFTGRARWGINLADHIDTDCNGHGTHTAGTVAGQKFGILKKASIVSVKILDCYGHGDITRYINGLNWAINDAKERGLLGKSVMNISLKTGRSRAVNEATVRAQEAGIFIAVAAGNQATNTEFYSPGSAPEVCTVGASTRNDTRAIFSNYGELVDLFAPGEYIRSTLPHNFTGLMSGTSMATPHVCGVGGLIMATEGLAPEKVCDRLKELANPIIQHPGFNTTNKLLYNGSGT*

>DEHA0D03520g.t1_gene_DEHA0D03520g_Dhan_CBS767_chrD:complement(236357..237772)

MGFLKLLSTSLATLAVVNAGKLLTANDGDEVVPSSYIVVMNDGVSTAQFETHRNWAANVHARTRSLKGGESGPGKHFDINGMKGYSASFDDRTVKDIASDPTVKYVEPDMVVNATANVVQRNAPSWGLSRISSKKSGATDYVYDSTAGEGIVIYGVDTGIDIGHADFGGRAEWGTNTADNDDTDGNGHGTHTASTAAGSKFGVAKKASVVAVKVLGADGSGTNSQVIAGMDWAVKDSKSRGATGKSVMNMSLGGAYSRAMNDAAANVVRSGVFLSVAAGNEAQDASNSSPASAPNVCTIAASTNSDGSASFTNFGSVVDLYAPGKDITAAYPGGGSKTLSGTSMAAPHVAGAAAYLMALEGVTSDKACARIVELAISSISSAPSGTTSKLLYNGINAQ*

>ECU01_1130.t1_gene_ECU01_1130_Ecun_GBM1_chrI:complement(132275..133672)

MGFLFKLFASTLAVASAVNSAELLNFENERDVIPGAYIVVMKDGLSSSNFRTHLSSVPQGNEGKRGIQGSDGLQFSFDIEGWRGYSGRFDNETLSNIANHPDVKFVEPDRMAKASILKFQKAAPSWGLPRISHRFRGFRNYIYHRSSGKGVVAYVVDTGIDIHHPEFEGRAEYGINVVDEVEGDENGHGTHVSGTIAGKTFGVAKGVKLIAVKALGKNSRGPDSGIIAAMDWAVKHAKEKRTIGKAIMNLSLTGDTSTALNEAAERAVEAGFFLGVAAGNNNRDAINESPASVKSVCTVGASTINDEKASFSNFGARLDIYAPGANITSALPNGRHGAMSGTSMATPHVCGVAALLISSEGIRAKHACDRIKQLATRRAIKNPGEKTTSRLLYNGSF*

>AN3583.2.t1_gene_AN3583.2_Enid_FGSC_A4_chrII_scaffold_4:join(373934..375690375741..376443)

RSYDYSPAARLGEGQVGELDGHHTFSKRIEDPIFTKQWHLFNTVQVGHDLNVTGVWLEGITGKGATAAIVDDGLDMYSNDLSPNYFPEGSWDFNDHTAEPRPRLRDDRHGTRCAGEVAAANDVCGVGVAYDSRIAGIRILSGPIDDTDEASAINYAYQENDIYSCSWGPPDDGATMDAPGILVSRAIVNGVQKGRDGKGSIFVFAAGNGAASGDNCNFDGYTNSIYSITVGAIDREGQHPQYSESCSAQLVVAYSSGISISTTDVCYSVHGGTSAAGPLVVGAISLALSVRPELTWRDAQYIVLETAVPDGSWQVTKSGFSHDWGYGKIDVYSLVQKAKWELVKPQAWYHSASWEVTEQMMKDANEKLEHVTVTMNVNHTRRGDLSVELRSPEGVSHLSTPRKNDNAEVGYIDWTFMTVAHWGESGVGTWTVIVKDDWRLNLWGMAVDGAKQRRRLRYDFLYNAF

>AN7159.2.t1_gene_AN7159.2_Enid_FGSC_A4_chrIV_scaffold_9:complement(join(609698..611409611477..611734611773..611779))

AFEPDGWLRIALQHNVAGFEQALSTPGHSSYGQHFMKQLLLPTEEASSSVRDWLDWINFVDQANALLDADFLWYRTLSYSVPSELAGYVNMIQPTTRFTPSCLADLYSKVAFASFLEEYARYDDLAEFEFSVISIGGNDQEANLDLQYIIGVSSPTEFTTGGRNEPLDFLEAVLKLDQKDLPQVISTSYGEDEQIPYARSVCNLYAQLGSRGVSVLFSSGDSGVGCQTNDGFPPQFPASCPWVTAVGGESGSSGGFSDYWARPAYQAVESYLYFNRSGRAFPDVAAQAFAVVDKGFDGTSCSSPVFAGIVALLNDVRLKAGLPVLGFLNPWLYLNDIVDGGCGWNATEGWDPVTGLGTPFAKL

>EXU96178

VIHTSRVVRIALQRNLDKGMEYLSDPSSKNYGSHYVVDLFSPAPESIETVKRWLGWLDFVGQLEDILKTNYHLYGADSYSLPSEVSQHVDFITPGVVPTPACIKALYNQLGMFESDNEMHKQSDLDQFYPKIDLIWGTKPEAALDFDVSIPVIYPELYQTKSNDDPVDGVTANEACGTFTPANVISFSYGLTENWPTQRQCDEFMKLGLQGSSIVFASGDGGVACLGSNGFNPASPSSCPYVTSVGAESAGSGGFSNIWPSPDYQAVASFFIYNRAGRGFPDIAAIGGVIVLNGTGGTSMSAPIVAAIFTRVNEVRLKAGKKPIGFANPALYFKDVTLGACGFSAVKGWDPVTGLGTPFPAV

>EXU96296

FIFFRGWPVHFMTIDKLHGYTGKGVHVAVIDTGDYKHPSLGGCFGGCLVTKGFDLVPDPMDCHGSHVAGIIAGGAPGVTLGAYRVFGCDVIIAINRAYLDNADIITMSIGAVTASRIVVIVTISAGNGSQGFYASSGSSGEGVAAIASYDNDLCVLIRGASAFSSWGPTMDVKPQFGAPGGILSTYPGTSMACPAAIYALSKPAPVAQQGAGLIQAHDATLLENETLTNGDSKVTYEISYVPTTLAKGLPVWSGYVPYQGGRLREGTYRIIVRMLRLYGAW

>EXU96513

LILFRGWRVHIMTVDLLHGFIGSNITIGLVDSGGCKVAFGDNLGPMDCHGTAVAGVLAGVAPNATLGAYRVLDCDDFMGLIRAFDDGVQIIVSSAVAIIASRIVVPCIIGVGNQDLGFSTSIPSTGRGVIAVTSFGQGSNSASGPTLDIKPNVGAPGRVRLVGTSFAAPGGIVALAAPVARQGGGLVQAWEATLVHNDTITNAESEVTYELSNLPARLRPGLPVWSGWIPYQGGSLPPGSYKLVARALAIFGHW

>EXU96563

YIIFYGWPVHAMTVDMLHGFYGTNITIAVVDTGNYTHPALGGCFGGCRVARGANFVHPMDHHGTAVAGVLAGVAPGATLAAYRVVDSDDLIGWLKAVEDGAQIIASSAGAAVVARIAIPCIVGNGNSKKGFFSLDPSTGRNVMAANSFAHSGLSASGPTLDLKPNVGVSGDIPCPGTSFAGPAGMVALAAVVVQQGGGLAQAWQATLIENDTVTNALFEVTYQLSILPAKLAANLPVWSGWVPYMGGQIPPGDYELEVRALRIFGDW

>EXU97232

LILFHGWPVHLMTVDKLHGYLGSGIKIAVIDTGDYNHPALGGCFGGCRVVTGENFRPIDCHGTIVAGILAGAAPNATIMAYRVLNCDDMIGWLQAEKDGAQIIVSSTGAVVAARIVVPCVVGLGNKEQGFYAMNPSTGRGVTSVNSFGRALIRGAAPMSAYGPTMDIKPTIGAPGHVPVTGTSFAGPAGVFALGAPVAQQGGGLLRAWEATLVENDTITNAKTEVTYRLSHLAATLGPGLPLWSGWVPYLGGAFPPGRYKIVARALSIMGHW

>EXU97623

YIFFKGYPVHVMTVDKLRGITGKGVKIAVVDTGDYKHPALGGCFGGCLVAFGTDLVPDPMDCHGSHVAGIVAGAAPGATLGAYRVFGCDVLIAFNQAYQDGANIITASIGAEAVSRIVVPCTVSAGNGAEGFYASTAANGRRVSAIASYDNDGCVLIRGASTFTSWGPTMDTKPQFGAVGGVLSTYPGTSMSCPAGIIALANPAPVPQQGGGLVQAYDATLLSNDTLQNDSKGITYKITHTPATLNGGLALWSGYIPYQGGRLPPGKYKFVVRALRIFGDW

>EXU97954

YIVFKGWPIHVMTVDKLRGVTGKGIKVAIIDTGDYTHPALGNCFGGCLVSFGTDLVPDPMDCHGSHVAGIVAGAAPNVTLGAYRVFGCDILIAFNKAFEDGAQIISASIVAVAVSRIVVPCAISAGNGDHGFYISTAANGKGVTAVASYDNDACVLVRGASLFSSWGPTMDFKPQIGAPGGILSTYPGTSMSCPAAIITLANPAPAAQQGGGMVQAYDASLLSNDTITNGTNPATYRLGQVSSTVAPGLALWSGYIPYQGGKLPAGKYQFVTRALRIYGDW

>EXU99167

HVVFEGWLTHLMTVDKLHGYTGKGVTIAVVDTGDYTHPALGGCFGGCRVAKGANLIRPMDTHGTQVAGIIAGALPNATIHAYASESSDTMIAWLEAYKDGAQVIVSSLGALVVSRITIPCIVALGNRLGFDAAAPGTGRGAVAVNSFSRALIRGAADLSSYGPTLDIKPNVLAPGEVRSTGTSHAAPAGIYALAEPVAQQGAGLVKAWEATLVSNDTIKNANIAVQYQLTHISATLSSGLPLWSGWIPYLGGSLPPGRYKLMARALSILGDW

>EXU99232

FLLFYGWQVHVMTIDKLHGFTGSGIRIAVVDSGDYTHPALGGCFGGCRVALGGNFDPMDCHGTAVAGIVAGVAPNATLAGYRVLDCDDLIGWVKAYQDGAQIIVSSAGAAVVSRIVVPCIVGLGNNNSGFNTLNPSSGRGVTSVNAFARAQSSTFGPNLEIKPTVGAPGDVPGIGTSFAGPAGILALAVPVAQQGGGLARAWDATLIENDTITNARVKVTYHLDTLAAVLGPGLPVWSGWIPYLGGRLPAGKYNLVVRALRLFGDW

>EXU99937

QHEPQGWLSIALQPEIHRLASKFGSGHLVRTLRAPDPKDAAAVVDWLDWIHVVSTAESLLNTQLQRYRAREYSVPSHLSDAISFINPISNFTPKCLRQLYVRLGVSGYLEEHSNHADVRDFLFKVELVGGDPQEAQLDLEYVMGLGFPTYYATGGRNEPLEFIQALLDKPDNEVPHVLSVSYGDDELVPYAERVCGMLGLLTKRGTSIIHSTGDGGSACRTKDGTMSTFPASCPWVTAVGAPSGSSGGFSQYFERPAWQAVDKYVYYNASMRAVPDISAVGFRVIVGALEGTSASAPVFAAMISLVNDARLRKGKPSLGWLNEILYLQDITKGSCGWPAKQGWDAITGLGVPFAKF

>EXV00396

YFVYKAHQVHVMMVDKLRGITGKGIKIGMIDTGDYNHPALGGCFGGCLFSFGADLVPMDCHGTNAAGIIGGAAPGAQLGMYRITCDVMVAIYRALADGVDIISSSAGSSAATRAVVVFVQGAGNGTLGFSHLDPAVGNGVISVGSVNSHACVLLNGASDFSSWGPSLGLEPSLTAVGGIISTDFGTSFSGPSAIVALSNPAPVAQQGGGLARAYDATLVQNDTIKNGQGAITYRLSHVPATVDPGMPLWSGWIPYQGGQLPEGYYKLVVRALRIFGDW

>EXV03254

IHESGRWVRIALQKNLDKGMDYLSDPSSAKYGQHYVVELFAPDESSINAVRSWLGWVDFVGQLESILKTKYHMYGTDEYSLPNEISDLVDFITPAVVMTPACIKSQYNRMGIFEISEDVYSQEDLDSFYPKIDLIGSAPVESDLDFEIAIPIIYPELYEAANDDDPVDGNTPNEMCGTFKAANVISFSYGTAEAYPYLQRQCDEFMKLGLQGTSIVLSSGDDGVACLGPKGFTPGQQASCPYVTSVGSEIASSGGFSNIWSTPDYQAVSSYFIYNRVGRGYPDVAALGAVVVVNGSGGTSMSAPLVGAILTRINEERIKAGKKSVGFANPALYFTDVVRGACGFSAVEGWDPVTGLGTPYPAM

>EXV04474

LEPDGWFSIALQPEMHGLASKIDGLTRSLRTPAQDDVDHVMEWLDWIRVVGEANNLLDMQLRRYRAPEYNIPDSLDTAIDFIHPIANFTPDCINKLYIRFGIAGFLEQWANYDDTRRSFFTVELIGGNQQEANLDIQFGMAVGYPIYYSTGGRNEPLDFFHHLSSKKNEELPHVLSISYADDELVPYAIRVCNEIGMLASRGVSVLSGSGDGGAKCRSNDGTISTFPASCPWVTSVGANGSSSGGFSAYFKRPDWQAVSEYIYYNSSMRAVPDISAIGFQTVINGLDGTSASTPVLAGMIALVNDARVRQGKPVLGWLNKRLYLQDIKAGSCGWPATEGYDAITGLGVPFNRF

>EXV04772.1|_peptidase_S8_famil

RDYDYPPASRLHEGTVGALSDHHVFRKRIKDPIFTAQWHLFNSVEVGNDVNVTGVWMEGITGKNATVAIVDDGLDMHSEDLRENYFAEGSYDFNDHDPEPAPVLSDDHHGTRCAGEVAAVNDVCGIGVAYESKVAGIRILSAVISDEDEAEALMYKNDKNQIYSCSWGPSDDGRTMEAPSVLIRRAMLKSIQEGRNKLGSIFVFASGNGAKSGDNCNFDGYTNSIFSITVGAVSRDNQQTYYSEPCSAQLAVTYSSGGSIHTTDVCTDRHGGTSAAAPLAAGIFALVLEVDPELSWRDMQYLVMDTAKPGVVWNQTGIGFSHAFGYGKIDTYDLVQKAKWNKVKPQAWFFSANFTVTKDMLKEANERLEHVTVFMNVNHTRRGDISVDLISPSSVSQIATTRSGDEHYAGYVNWTFMSVAHWGESGVGTWTLVVRDDWRLKLWGESIDAKKAKRRYEFLYDAF

>EXV06306

YIVFTGWPVHHWTVDKLHGMRGKGVKVAVVDTGDYSHLALGGCFGGCKVAGGYDLVPDPMDYHGTHVAGIIAGVAPGAELLIFKDVLIAFCDAYTAGADVITASVNALVASRIVVFVSIAAGNGTRGFYSGVGSNGRHVVSVAAAYFTSWGPTLIMKPDIGAPGYILSTYLGSSMAAPAGIAALGRNAPPFQVGTGLVDAWKITQVSLDTITNANQTVKYTFEHESLTLHSGLPLYSGKIPYGGGKLSPGNYT

>EXV06538

YIVFHGWPVHLMTVDKLHGFSGKGIKIAVVDTGDYTHPALGGCFGGCRVAFGDNFKPMDCHGTQVAGVLAGAAPNATLMAYRVLDCDDMMGWLKAYEDRAQIIVSSTGAMVASRIAVTCIGGLGNQEQGFYAMAPATGDGVISVNSVASPYLSAYGPTLRIKPNVIAPGQIWVTGTSYATPGGIVALAKPVAQQGGGLIDAWEATLVENDTITNAKSEVSYELSNLAATLGPGLPLWSGWVPYLGGAYPPGRYKIIARALAVFGDW

>FGST_00151_gene_FGSG_00121_fgra_supercontig_3.1:complement(join(370176..370883370941..371597371654..372037))

MEFFAEWLKRKGVERILTLQVQEDSQDPHSDESIQIVLRHFTVEHLDWQKPDLDPLIMCERADIGNVDQDTWKLDSANTLIKPRKDLRQLTLKWSGSNAALRAWSEPAGLPQMLELRKIDIHIPAVLELPDHRDWIKRNLDLFQVRLNQSYRTNHPLKKPDGQSKELNQPDHRGITVIESWDDRKFENATARKVVPKSTGMPDAADEHEWINQMEKFSRPMRELWEETLKESINPLSSSPEEARLLSNADSKILSTLRKDVVVALIDDGVDTLDPAFSNQFVEGKTFDYQGDDGVGQYYVSANGHGTDMARMILKVCPMAKIYSIKLKTQPSKGGQHLTIDESSISPAIEAALEKNADVISMSWTIPIPVTGSDNQKRIDNVLKLACSKDVLMFCSSPDQRTQTKHYPSHYNREKIFLIGAADDSGTAFNHSGLDNDFIFPGVNVNTGNNLGRYHSNDSTSPVQMATGSSIATALAAGLAAMVTYCFKTSALAAVTTRIAQGRPPAASGTELIKPQDVGRIAHHDGLKKVFDRIGTMDGGKFIAVWRIFRPATECLTDGKMTYEQKVTHIMELCRDLMDRMDR

>FGST_00238_gene_FGSG_00192_fgra_supercontig_3.1:join(586031..586396586453..587685)

MSILFKLFASTLAVVSVVNAGELLNFENERDIIRGSYIVVMKDGTSSSDLKSHMNWAANVHHGNLAKQGPSRSTGFQFSFDIGGWRGYSGKFDNDTLDAIVNNAHVKYVEPDRMASATVLKIQKNAPSWGLGRISHTFRGFKNYLYHSSAGEGVLAYVVDTGIDINHPEFEGRAEWGINVVDEVDTDEHGHGTHVAGTIGSKTFGVAKKVKLVAVKALGKDSRGPDSGIIAAMDWAVKHAKEKGILGKAIMNLSLTGDTATALNEAAERAVEAGLFLGVAAGNNNRDAINESPASVETVCTAGASAENDEKASFSNFGSLLDIYAPGNKIISTLPGGGNGTMSGTSMAAPHVCGVAALLMSSEGIKAQEACDRIKKLARPAIRNPGNSTTNKLLYNKSGF*

>FGST_00947_gene_FGSG_00806_fgra_supercontig_3.1:complement(join(2574981..25759132575964..2576269))

MSILFKIFASTLAVVSVVNAGELLNFENERDIIRGSYIVVMKDGTSSSDLKSHMNWAANVHHGNLAKQGPSRSTGFQFSFDIGGWRGYSGKFDNDTLNAIVNNAHVKYVEPDRMASATVLKIQKNAPSWGLGRISHTFRGFKNYLYHSSAGEGVLAYVVDTGIDINHPEFEGRAEWGINVVDEVDTDEHGHGTHVAGTIGSKTFGVAKKVKLVAVKALGKDSRGPDSGIIAAMDWAVKHAKERGILGKAIMNLSLTGDTATALNEAAERAVEEGLFLGVAAGNNNRDAINESPASVETVCTAGASAENDEKASFSNFGSLLDIYAPGNKIISTLPGGGNGTMSGTSMAAPHVCGVAALLMSSEGIKAQEACDRIKKLARPAIRNPGNSTTNKLLYNKSGF*

>FGST_02821

KVEPNGWLSIAMQPDIENLKTGLDATSGQYIQRHLALALRTPDKKDVDKVLAWLDWIHVVKDAQDLLDAKIGFYRTRDYSLPESLVNSISFIHPIANFKPDCLREQFIRFGIAGFLEEYANYEDAQDFLFSVQLIGANSQEAALDVQYAMALGYPTYYLADGRNEPLEFLDYLLDLSDDEVPHVLSVSYGDNEVVPYAERVCSMFGLLTARGTTILAASGDGGAKCRTNDGTMAVFPATCPWVTSVGGFEGSGGGFSQYFPREKWQSIKSYVNYNASNRGVPDISMPAYITRLKGLRGTSASTPVMAAMIALINDARVRKGKNVLGWINEVLYLSDVTAGPCGWPAAKGWDAITGLGVPFQKL

>FGST_03404_gene_FGSG_12242_fgra_supercontig_3.2:complement(join(526211..526813526864..527233527287..528276528347..528738))

MTTINREIIDTLRTQCSEYQTEDLMDDWVDVDATDDHKIMHSTYQNMESKDTQTSLPKSMEDAQDALYSFYDLLAERWPLDCKDAPHSAMLKLVADRQNVGKNGGTTQLFEFDTVSSTHRKSWVRVRFHAEVPEHISEMPQRVTFQDSSQSGNRHQCEITRLELCRMIEASESNWKCPTINLVSGKTAKTRGDTPVKCSKQVITLESLVRPKMAAVSLRENSDLPPLTLHQKAKLAVVFASSVLQLSKDSWGVKPPDEDPWSHEGPWLQRNWKDTGICFLENYLGKLSLGDPYLPIPIEDPARKSAQTSAQKIDLPYQSATLVALALTLLHLQSHEDRVLEEFDTICKNIRAEGYRNPDTTDYVALLHLLQDSQAFLNQVDDQYKTAIQACLRGRLVDAIGEEDERTVQQYFFQDIIIPLNSYLKTLESSENLSIQKKYSEQKWSLWDDHDGEEHQGDSTLSGYADTWFEKFESKVQGLVDLSTSRHHKDPIKVAIIDTGLDFPREARSLYKKQIKDCRTWLYCDTSKHIKMEDLCTDSDGHGTHCASTVLKVAQNAHIYVAKVLEIRSQEKARSETTTQAIVKALNYAIDEWKVDIINMSFGCRNRVAEIDEVMERAERNRIIMVAAACNLGALEPISWPAKSNRVICVHAADGFGNPAPFTPDPEPNNHNFAAPGMAILGYCPKGSDKLQRRMTGTSCAAPVVAGIVAVLLEFVRKHEAEYTDKHRLLELLRERRGITAVLQKMVSKGGRNKYDFLSPWSLLDVERPYQHHMNTILETLGAVG

>FGST_04745_gene_FGSG_03315_fgra_supercontig_3.2:join(3978896..39795313979588..3980184)

MARINVVVSFLAALAVVQAAQLLNLDGQKDAVPGSYVVVMNDGLSGLDFESHVKSMAKVQKANALKRDFDNTADGVKFKYNINGWQAYSGKFDNKTIQSILDDPRVNYIEPQRTFRAFGWVTQDNAPSWGLGRISHTSRGRMDYVYDSSAGENVTVYSVDSGVDISHPEFEGRAIWGVNAADNSDVDQIGHGTHTSGTIAGKTYGVAKMAKIVAVKVLDAGGQGTNGGIIQGINWAVNHARQNNVTGKAVMNMSFGGGLSRAINEAASSAVRAGIFMVAAAGNSNEDARYTTPASARGVCAVGASTQNDLKARFSNWGPTLAVYAPGDRIWSAMPDGGRDVMRGTSMAAPHVAGVAAVLISSEQIGTERLCDRIKELSVSSIQSPGADTTDKLLYNGSGQ*

>FGST_05139_gene_FGSG_02976_fgra_supercontig_3.2:join(4887470..48877544887804..4888685)

MARINVVVSFLAALAVVQAAQLLNLDGQKDAVPGSYVVVMNDGLSGLDFESHVKSMAKVQKANALKRDFDNTADGVKFKYNINGWQAYSGKFDNKTIQSILDDPRVNYIEPQRTFRAFGWVTQDNAPSWGLGRISHTSRGRMDYVYDSSAGENVTVYSVDSGVDISHPEFEGRAIWGVNAADNSDVDQIGHGTHTSGTIAGKTYGVAKMAKIVAVKVLDAGGQGTNGGIIQGINWAVNHARQNNVTGKAVMNMSFGGGLSRAINEAASSAVRAGIFMVAAAGNNNEDARYTTPASARGVCAVGASTQNDLKARFSNWGPTLAVYAPGDRIWSAMPDGGRDVMRGTSMAAPHVAGVAAVLISSEKIGTDRLCERIKELSVSSIQSPGADTTDKLLYNGSGQ*

>FGST_07235

WPVHHWTVDKLHGIRGKGATVAIVDTGDYTHKALGGCFGGCKVKGGYDLVPDPMDYHGTHVAGIIAGVAPDAELLIYKVFSVTIMALCDAYNAGADVITSSIGAVLASRLVVVMIASAGNGEFGFYSSSGAIGHGVLAVAAVYFTTWGPTLLLKPDIAAPGFITSTVLGTSMSAPAGIAALGKSSPPFQVGTGLVDAIKVTQLERDTITNGGRVHQYKFKLEPQFIEPGLPLYGGKVPYGGGKLVPGNYT

>FGST_07502

YIFFKGWPVHVMTIDKLRGVTGKGLKVALVDSGDYKHPALGGCFGKCLVSFGTDLVPDPMDCHGTHVAGILAGAAPGVKLGSYRAFGCDILIAFNQAYEDGADIISASIGAVAVSRIVVPCVLAAGNGSAGFYASTAANGKKVSAVGSFDNDGCVLLRGASAFSSWGPTMDVKPQFGAPGGILSTYPGTSMASPAGIVALANPAPVPQQGGGLVQAYDAVLLSNDTLKNGKKQIDLQISHVPATIDAGLPVWSGYVPYQGGELPAGQYVVRYKALRIFGDW

>FGST_09702_gene_FGSG_08464_fgra_supercontig_3.5:complement(join(1775181..17762001776253..1776576))

MAHLKFLVGILAAVSASQAAQLLNFQNQKDVIPNSYVVVMNDGVSALDFESHVKSTRSVHDANTRKRGIAFPTGGVKFTYDIAGWRGYSGKFDNETLQEIIKDPKVKYVEPDRMAKALGWVTQSNAPTWGLGRISHRSKGVRDYVYDSSAGEGVTMYAVDTGVDISHPEFEGRATWGINVADNVNTDQHGHGTHTAGTLAGKTYGVAKKAKLVVVKVLNRRATGSDSGIIRGMNWAVDHARANGAIGKAVMNLSIGSPDSSALNEVATKVAESGIFVAAAAGNRNEDARNTTPAAAAKVCAVGASAANDVKARFSNHGPILAIFAPGVSILSSVPGGRARTMSGTSMAAPHVAGVAATLISSQGVRTDKLCDRIKQMSAATVLYPGVSTTNKLLYNGSGQ*

>FGST_10116_gene_FGSG_08102_fgra_supercontig_3.5:complement(join(2887059..28870602887299..28876772887728..28884532888568..28885972888653..28888482889012..28890382889095..28891552889205..2890135))

MPEEMLDSGSDSDPEKPVLRQIAKDHQQAPSKEQDRESLLRELGQFSFTTLEEYKAANAERLKRCYMADERDQNRNILHWLSVHLPSNPTDDENTSLHWLVTTVVELEPKIVTMVATDSQKANCLQTAIEHARFDLIESLFKTSDDEALRVAISQGNHCNETCLHLAVRLGPPGVGLTLQLLENAHPKAILKQRKYRFEDDKPNHGNTVLHDFVHINVCFVKGYMKTLRRFIQLCPEALMVSNAAKESPFQFHIATRNKVYPDWQGLEFSPRTERHDKKKEAAAKVGRLLLDEAFSQSTWEDACGCVYGEKTFNQATTFRPAAPINKRIDSPAQPPRQTRDAVIEECLRDFDVRYLQWTKDDLCIEVLHNAGLSNVKELWLQWSGRNSVLYSWSCKDTGLPKLPQLEMVHIHTRAESIDSVQIDEYQSDLTTKVTKTLQEELSPLGSKLQASMSTWSGGPDYVQIAKVGPGEKPPMIEATIAGFKARAMFDRQDTKGVRQIKVDDESHETLSEANASKADDRQEQKCVENPIVNEKQASDHSFHRGHRWLDAIKRLKTAINIYKQNNQITIRPIRVALLDDGVNPGELVVPGVLKDGWPLPSTSRLHSSKPYYSSDQGHGTKMARLLYFMCPFISIYVAKIDMYREHDTSAAMSAAKAINWAVSKNVDIISMSWTVKQVRYGPNSNQTAITALERAIQAAANSDILLFCAVQDSGHYENDEISFPQKSDTKKLIIVGSANENGDKSTFVNENSFNYLFPGEIVIPDILTEHDKGSSVATAVAAV

>FGST_10218_gene_FGSG_08012_fgra_supercontig_3.5:complement(join(3115444..31159813116032..31162043116275..31164543116515..3116793))

MHFFDTILPLALVVLSTADAAAVLKVPRNSHAVPNSYIVVMKPGTSDDRFESHRSWVSNKLSASDSGVDNGGVRHDYNLDSEFKAYSGIFGEDVIKQISNDEDVAYIEPDIVVKLDKLRIQKNASSWGLGRISSTKPGSPHYIYDEKAGEGITAYVIDTGIDVNHPDFDGRATWGTNTVDSRDEDCGNHGTHVAGTIGGNRHGVAKKIKLIGVKVFGCDEDGGGSNVIRGMAWAYAHATHNGDPKKSIMNMSLGAPYSRSFNEAAAAIVRAGIFLAVSAGNDGFDASKKSPASEPTVCTIGATDSEDTIAKFSNYGSGVDLFAPGVDIVSTVPGGKTDSYSGTSMAAPHAAGVAAYLMAIENISGGAVCDRLKELARDSVKGAPEGTTRKLLFNGVRRHYKSKPRKKVPKRPEECPPCERKYSY*

>FGST_10483_gene_FGSG_13519_fgra_supercontig_3.6:join(579173..580619580675..581201581257..581847581904..582641)

MEETSDSFQDLEGQPNQPRSLTNNEVSDENHGQDELGDSSEYSDTDDDEDEEEVHGDSNQNLARTYEQQAKEFKDQATHIKAQLDRQMDNFASQCPDNDNADARNRFYRQFRHHMAPPKAQIVKHLSVWQWNILYYIADEKQSDRPCDWLVGRLAKEFPSLLNERMPNGGLTVLLKAVKEHNFAFIKAVLDSGISTTNLVKIVVERDVDKKNCIHYAVSDGFDPEITIRLLQHTTGETLGHQDNKGLTPLHYAVEYERCLPEREEMVKALIKRSDVAFDLRSRKPEELSVYQHHVRSREKYGKKKELRRMERERKEKEKQSKDYKEKTSDATPGRHETGSMSRKDEAKNSKRLSEQPNRMGFKMREGADLKVPGGSESNNMDTLQLLAGSKRDARDQNQFGRPLTRTRTGSQKPPNPKKVSSRKRPKLPKPSEDVADRILKELKLHYLRTALCQESHWQYGQRGSRRNPNTAIQFLYGDNEQETQIGFEFPPKIPKTPNEINFRDFTKSYESLKFDKVLQHVEFRQMTVKSPPVKRPDWTDSEGRAGTGRKDVYYFFRWLSKTKSVENIIHLIVEDGYGISHSDEAIEESLKYFNIEILDWRKTDLDPMTLQIACRNSDLREIHLWWDGNNAVLRAWSEPDGLVKITTLQVIHLHETTKNIESSNRTKARIESFKTRLREHRAKVLQPLECYHYEAYEKNPSRTEGQMPTEKLEQRAKIDSYQWLSVMDRFADGIASLEPLECFQGEDDYLKHPSLPDELRKDVKVALIDDGVNFLHKSLAENIDGGKSFDREYDDGYRPGLREPFHGSATGHGTCMAYMIRRVCPRVKIFGCKLNVLRGNVDGKASFTAKSAADAVEYAVARKFDIISISWTVQLQKDEKHDNSADINRLENAISLATTNGILVFCSAPDIGRASKETLELYYPFGCKRSSTDLFKIGAAKADGLSFAWMGHEETVNYILPGHKVAPRETDNLPEEDDTPKTGSSVATALASGLAALIIHCVRLAAIYNIYTNNRDSSSVSEASVRAIKRFPAMKQALARFYSNDGERQTSDRNLRVENFFDRPGHIMGSNEVTEEDKWKEVVNIARDLVSYKTITQTTT

>FGST_10511

RDYDYTPASRLGEGQLGALDDHHVFRKRIQDPIFKEQWHLLNPLQPGHDVNVTGLWLEGITGKNVTVAVVDDGLDMNSDDLKPNYFAEGSWDFNDNDPEPAPVLDDDRHGTRCAGEVAAANDVCGVGVAYDSKVAGIRILSKLISDADEAEALMYKYHDNHIYSCSWGPSDDGQTMEAPDVVIRRAMLKAIQEGRSGLGSVYVFASGNGAGQGDNCNFDGYTNSIYSITVGAVDRTGLHPYYSEECSAQLVVTYSSGSGICYKAHGGTSAAAPLAAGIFALVLQVRPDLTWRDLQYLAMDTALPEANQQNTTIGFSHTFGYGKIDSWALVEKAKWKLVKPQSWYFSVTLDVTEDMLKDSNARVEHVTVTMNVEHTRRGDLSVDLISPDNVSHLAVSRRSDAKDAGYVDWTFMSVAHWGESGVGKWTIIVRDDWRMKLWGEAIDADKARRRLRYEFLYDAF

>FGST_10778_gene_FGSG_09382_fgra_supercontig_3.6:complement(join(1344432..13445981344655..13448501344905..13451951345256..1345927))

MKPNTTDEKFETHRSWVSSKLVAADSGVSNGGLRDHFNFDDQFRAYSGVFGDDTIKQISDDEDVAYIEQDKVVQMHQFVVEKNAPSWGLGRISATEPGVKDYIYDSSAGEGITAYIIDSGIDAKHPEFGDRATWGTNTLDSQDEDCSGHGTHVAGTIGSTTYGVSKRIKLVGVKVLNCDGVGSSTAVLKGVKWAASHVQEHGDPKKSVMNLSLGQGFSRADNEAVAAVVRAGIFVSVSAGNEHRDASNYSPASESTVCTIGATDDKDKMAKFSNFGSGIDLFAPGEDIISTVPGGKIASYSGTSMASPHAAGVAAYLMSLENISGGSVCDRMKSIARDSAKGTPKGTTTKLLYNGRKSIKKDPKPKDPKPKDPKPKNPKPKKPKTKNPKLCHSRRSRRMLAY*

>FGST_12083_gene_FGSG_10525_fgra_supercontig_3.7:complement(join(2152845..21534232153473..21538172153865..2154140))

MLFFDYGLAFVLAALSVVDAAPVNKAPVGIQVVPNGYIVVMKARTSPERFERHRKWVGNMRKNRKGIKAPKALRSFQFNSGWKGYSGVFDSETLIQIANDEDVDYIEEDGIVTANALITQTGAPWGLARVSSRTPGAKNYVYDSTAGQGVTAYIIDTGIETSHRDFGGRAKWGTNTVDSQNTDCHGHGTHVAGTVAGTTYGVAKKANLIAVKVLNCQGSGSNSVVIQGMQWALNHATQNGITKKAVVNMSLGGGYSQATNQAAAAIVKAGIFLAVAAGNDAADASKYSPASEVTACTIGASNSSDLIASFSNRGKLVDVFAPGVDIQSTYVGGGTKSMSGTSMASPHAAGLGAYLIGLEGISGNAVCERMKALSSAVVRGVPSGTTNKLIYNGVK*

>FGST_12163_gene_FGSG_10595_fgra_supercontig_3.7:complement(join(2398982..23998692399922..2400191))

MGFITKALPLALAAFSTVNGAKILEASPGADTIPDSYIVVMKAGVTSETFSAHTDWVGRTYQRRPMRRGANSQPMAGLQHSLSFGGNFMYAGQFDQAMIDDIAKNDDVDFIEPDYKVTLSSITEQKDVPSWGLSRLSTKEPGGTTYFYDESAGEGTTAYIVDTGIDVNNGDFGGRAKWGNNFVDHQDTDCNGHGTHVAGTVGGKNFGVAKKTNLIAVKVLDCNGSGSNSGVIKGMEWVMRDASGGGNSTSKASKSVMNMSLGGPRSEATNRAAKAISDAGIFLAVAAGNDNADSQHDSPASEPSVCTVAASAEDDTKASFSNWGQAVDVFAPGMHITSDKPGNGTAVLSGTSMASPHVCGIGAYLIGLGKKGGPGLCDTIKDMARPVIKNPGQGTTNKLIYNGSGK*

>FGST_12292_gene_FGSG_10712_fgra_supercontig_3.7:join(2709040..27093362709386..2710273)

MGFITKAIPIVLAALSTVDGAKILEAGPHAETIPNKYIVVMKQDVSHEAFNAHATWVGNNFSRRPMRRGGSFKPMAGMQHKFSLGGTFKAYTGEFDEAMIKDISNHDDVDFIERDTVVKATAITQQDNVPSWGLARVGSKEAGGSTYYYDDTAGKGVTAYIIDTGIDIHHGDFGGRAKWGKNFVDKMDEDCNGHGSHVAGTVGGTKFGVAKGVNLVAVKVLDCEGSGSNSGVIMGMEWAMKEASGGGNSTAKAAGKSVMNMSLGGPRSEASNKAAKAIADAGIFMAVAAGNDNMDAQHSSPASEPSICTVAASSEDDSKADFSNYGAVVDIYAPGNEITSVKPGNGTDTLSGTSMASPHVCGLGAYLIGLGKEGGPGLCDTIKEMATDAIKNPGEGTTGKLIYNGSGK*

>FGST_12319_gene_FGSG_13826_fgra_supercontig_3.8:complement(join(49380..4951649564..5123051277..51533))

MVNITINGNTVNTNELPAELPQTAAKTNFILIQTYNRDLNSTEKLELADLDVDIQEYVAQFTYLCRYEKEDLEPIRAKRYVKSVTIYLRELKSTISLKDMVDRELDRTFYRVDCILHETPNVTAEKLAPEIAEKAGVDITKLAVSPVRIRLTVHQDKLEALAKLDSISRIEEVRPDEVLNDLARETLNANILALSTSYEGNGQKVCVADTGFDQGKMADEMGILVHPAFNGRVEHLEALWLGDSKDTAGHGTHVCASICGNGLYKNGDIRVRGVAPGATLMVQSIAQVSRDPNKGAIEVPMDLGLQLFSNPYKLGYRIHSNSWGKVWDAKTGQLGYEGQAWDIDKFVIDHQDFVVLVAAGNNAEKAKSKSNHIGAAGSAFNCITVGATGTTRPNNDYGFDNEVGAKPMTRINDTAKFSSRGPTKPGRDINGNEYAGRIKPDVVAPGVAILSAASRAMAKDSRNRVMYGRTGDDDWTFMSGTSMSTPLVAGCVALLREALKEHGKEKPSAALIKALLVNGAVNFSEQLGLGLGYDYDQGFGRVDIDSSISMVKLSSFVDGGKLFEDTQFDVAPLRQVPEEERRWTSSLIPVPAGRNRLTVTLAYPDKPAQSGLMQNDINLIVLSGGAERHGNMGKGPGYDHTNNVEKIIWENVPGETFKIVASIWNNIDVKAPTSFAVAWDIRPLARL

>FGST_12951

YIFFKGWPVHVMTVDKLRGITGHGIKVAVIDTGDYKHPALGGCFGDCLVSFGTDLVPDPMDCHGTHVAGIVAGTAPGVTLGAYRVFGCDVLIAYNQAYQDGADIITASIGAVAVSRIVVPCPVSAGNGDVGFYASTAANGNKVMAIASYDNGGCVLVRGASTYSSWGPTMDVKPQFGSPGGILSTYPGTSMACPAATIALSNPAPVPQQGGGMIQAYDAVLMDNDTIHNGKKEIDLSVSHIPTSVGAGLALWSGYIPYQGGKLPAGKYKVKYQALRIFGDW

>FGST_13031_gene_FGSG_11405_fgra_supercontig_3.9:complement(join(283675..283711283851..284638284690..285004))

MGFITKAIPIVLAALSTVNGAKILEAGPHAETIPNKYIVVMKREVSDEAFSAHTTWLSQNLNRRVMRRSGSSKAMAGMQDKYSLGGIFRAYSGEFDDAMIKDISSHDDVDYIEPDFVVRTSTNGTNLTRQDNVPSWGLARVSSKKAGGTTYYYDSSAGKGVTAYVIDTGIDINHEDFRGRAKWGKNFVDDMDEDCNGHGTHVAGTVGGTKYGLAKGVSLVAVKVLDCEGSGSNSGVIKGMEWAMREASGGGNGTAKAAGKAVMNMSLGGPRSQASNQAAKAISDAGIFMAVAAGNENMDAQHSSPASEPSVCTVAASTEDDGKADFSNYGQLVDVYAPGKDITSLKPGGSTDTLSGTSMASPHVCGLGAYLIGLGKQGGPGLCDTIKEMAHDAIQRPGEGTTSKLIYNGSGK*

>FOXT_01145_gene_FOXG_01145_foxy_supercontig_2.1:complement(join(3613218..36141563614207..3614515))

MGFITKAIPIVLAALSTVNGAKILEAGPHAETIPNKYIVVMKREVSDEAFSAHTTWLSQNLNRRVMRRSGSSKAMAGMQDKYSLGGIFRAYSGEFDDAMIKDISSHDDVDYIEPDFVVRTSTNGTNLTRQDNVPSWGLARVSSKKAGGTTYYYDSSAGKGVTAYVIDTGIDINHEDFRGRAKWGKNFVDDMDEDCNGHGTHVAGTVGGTKYGLAKGVSLVAVKVLDCEGSGSNSGVIKGMEWAMREASGGGNGTAKAAGKAVMNMSLGGPRSQASNQAAKAISDAGIFMAVAAGNENMDAQHSSPASEPSVCTVAASTEDDGKADFSNYGQLVDVYAPGKDITSLKPGGSTDTLSGTSMASPHVCGLGAYLIGLGKQGGPGLCDTIKEMAHDAIQRPGEGTTSKLIYNGSGK*

>FOXT_01284_gene_FOXG_01284_foxy_supercontig_2.1:complement(join(3988376..39896053989657..3990022))

MGFITKAIPIVLAALSTVNGARILEAGPHAEAIPNKYIVVMKREVSDEAFNAHTTWLSQSLNSRIMRRAGSSKPMAGMQDKYSLGGIFRAYSGEFDDAMIKDISSHDDVDFIEPDFVVRTTTNGTNLTHQDNVPSWGLARVGSKKPGGTTYYYDPSAGKGVTAYIIDTGIDIDHEDFQGRAKWGENFVDQQNTDCNGHGTHVAGTVGGTKYGLAKGVSLVAVKVLDCDGSGSNSGVIKGMEWAMRQASGGGNGTAKAAGKSVMNMSLGGPRSEASNQAAKAISDAGIFMAVAAGNENMDAQHSSPASEPSVCTVAASTKDDGKADFSNYGAVVDVYAPGKDITSLKPGGSTDTLSGTSMASPHVCGLGAYLIGLGKQGGPGLCDTIKKMANDVIQSPGEGTTGKLIYNGSGK*

>FOXT_02380

YIFFKGWPVHVMTIDKLRGVTGKGLKVALVDSGDYKHPALGGCFGNCLVSFGTDLVPDPMDCHGTHVAGILAGAAPGVQIGAYRAFGCDVLIAFNQAFEDGADIISASIGAVAVSRIVVPCVLAAGNGATGFYASTAANGKKVTAVGSFDNDGCVLLRGASTFSSWGPTMDVKPQFGAPGGILSTYPGTSMASPAGIVALANPAPVPQQGGGIVQAHDAVLLSNDTVKNGKKQIDLQISHVPATIDAGLPVWSGYVPYQGGELPAGQYVVRYRALRIFGDW

>FOXT_02695_gene_FOXG_02695_foxy_supercontig_2.3:complement(join(162933..163884163934..163974))

MGFITKAIPLALAAASVINGAEILETRAGVQTLADKYIVVMNDGMTDKDFDSHRSWVNRTHRRRLVRRGAKAMTGMKHTYRFPTGMKGYSGHFDEQMINEIAKRADVKYIERDARVQINAIEMQDNVPSWGLARVGSKEPGGTTYYYDSSAGQGVTAYVIDTGTDIKHEEFSGRATWGGNFVDDIDMDCNGHGTHVSGTVAGTKFGVAKKANVVGVKVLDCDGSGSNSGVIMGMEFATNDAKKKGAGKAVANMSLGGAFSQASNDAAAAIAQGGVFLAVAAGNDNVDAAMASPASEPSICTVAASTEQDGKASFSNYGQVVDVYAPGDGITSAKPGGGSQVLSGTSMASPHVAGLAAYLIGTGKSGGPQLCDTIKNMAIDVITNPGAGTTGKLINNGSGK*

>FOXT_03262_gene_FOXG_03262_foxy_supercontig_2.3:join(1931099..19314071931459..1932478)

MGFITKAIPLALAAMSVVNGAEILETRAGVQTLADKYIVIMNDGVTEKAFDSHRSWVNRTHRRRLVRRGAKAMGGMKHTYKFPTGMKGYSGHFDEDMINQIAKHSDVKYIERDARVQINAITEQDNVPSWGLARVGSREAGGSTYYYDSTAGEGSTAYIIDTGTDIEHEEFEGRATWGSNFVDDMDMDCNGHGTHVSGTVGGATFGVAKKSNIVAVKVLDCNGSGSNSGVIMGMEWATNDAKKKGADKAVANMSLGGAFSQASNDAAAAIANGGVFLAVAAGNDNVDAANSSPASEPSICTVAASTEQDGKADFSNFGQVVDVYAPGDSITSAKPGGGSQVLSGTSMATPHVAGLAAYFIGLGMPGGPGLCDTIKQKAIDAIANPGAGTTGKLINNGSGK*

>FOXT_04749_gene_FOXG_04749_foxy_supercontig_2.5:join(185821..186117186172..187113)

MGFITKAIPLALAAASVINGAEILETRAGVQTLADKYIVVMNDGMSDKDFDSHRSWVNRTHRRRLIRRGAKAMGGMKYTYNFPTGLKGYSGHFDEQMIKEISKRADVKYIERDARVQINAIEQQDNVPSWGLARVGSREPGGTTYYYDSTAGEGTTAYIIDTGTDIQHEEFDGGRATWGENFVDDMDMDCNGHGTHVSGTVGGRTFGVAKKSNIVAVKVLDCNGSGSNSGVIMGMQWATEDAQSKGADKAVVNMSLGGAFSQTSNDAAKAIAEGGVFLAVAAGNDNVDAAEASPASEPSICTVAASTEQDGKADFSNFGQVVDVYAPGDGITSAKPGGGSQVLSGTSMASPHVAGLAAYLIGLGKGGGPQLCDTIKQMAIDVIQNPGSSTTSKLINNGSGM*

>FOXT_04896_gene_FOXG_04896_foxy_supercontig_2.5:join(599559..599846599894..600244600290..600838)

MGFITKAIPLALAAASVINGAEILETRAGVQTLADKYIVVMNDGMSDKDFDSHRSWVNRTHRRRLIRRGAKAMGGMKYTYNFPTGLKGYSGHFDEQMIKEISKRADVKYIERDARVQINAIEQQDNVPSWGLARVGSREPGGTTYYYDSTAGEGTTAYIIDTGTDIQHEEFDGGRATWGENFVDDMDMDCNGHGTHVSGTVGGRTFGVAKKSNIVAVKVLDCNGSGSNSGVIMGMQWATEDAQSKGADKAVVNMSLGGAFSQTSNDAAKAIAEGGVFLAVAAGNDNVDAAEASPASEPSICTVAASTEQDGKADFSNFGQVVDVYAPGDGITSAKPGGGSQVLSGTSMASPHVAGLAAYLIGLGKGGGPQLCDTIKQMAIDVIQNPGSSTTSKLINNGSGM*

>FOXT_05775

RDYDYTPASRLGEGQLGSLDDHHVFRKRIQDPIFKEQWHLLNPTQVGHDVNVTGLWLDGITGKNVTVAVIDDGLDMHSDDLKPNYFAAGSWDFNDNDPEPAPVLDEDRHGTRCAGEVAAANDVCGIGVAYDSKVAGLRILSKLISDADEAEAMMYKYDDNHIYSCSWGPSDDGQTMEAPDVVIRRAMLKAIQKGRRGLGSIYVFASGNGAGQGDNCNFDGYTNSIYSITIGAVDRTGLHPYYAEECSAQLVVTYSSGSGIHTTDVCYKAHGGTSAAAPLAAGIFALALQVRPELTWRDLQYIAMDTAIPESNQQNTTIGFSHVFGYGKIDSWALVERAKWPLVKPQSWYFSVTLEVTEDMLKGSNARVEHITVTMNVEHTRRGDLSVDLISPDNVSHLAVARRGDAKEEGYIDWTFMSVAHWGESGVGKWTIIVRDDWRLKLWGEAIDADKARRRLRYEFLYDAF

>FOXT_05860_gene_FOXG_05860_foxy_supercontig_2.6:complement(join(814584..815109815167..815339815404..815562))

MGFITKAIPLALAAASVINGAEILETRAGVQTLADKYIVVMNDGISDKDFDSHRSWVNRNHRRRLIRRGAKAMGGMKHTYNFPTGLKGYSGHFDEQMINEISKRADVKYIERDARVQINAIEQQDNVPSWGLARVGSKEPGGTTYYYDSTAGEGSTAYVIDTGTDIQHEEFEGRATWGANFVDDMDMDCNGHGTHVSGTIGGKTFGVAKKSNVVAVKVLDCNGSGSNSGVIMGMEWATKDAQQKGADKAVANMSLGGAFSQASNDAAAAIAKGGVFLAVAAGNDNVDAADSSPASEPSICTIAASTEQDSKADFSNFGQVVDVYAPGDSITSAKPGGGSQVLSGTSMATPHVAGLGAYLIGLGKGGGPGLCDTIKQMAIDVIQNPGASTTSKLINNGSGM*

>FOXT_06062_gene_FOXG_06062_foxy_supercontig_2.6:complement(join(1396289..13964551396513..13967081396762..13970641397123..1397782))

MVLLSTLLPLALAIFSVDAAKILSAAPGVKTVPNGYIVVMKDGVSSQDFDSHRNWVTQLHHERLARRGSTNVGGMRHTYKTTLKGYSGTFDEETIQEIANRDDVAFIERDQIMTINEIETQPNVPSWGLARVGSMRPGGTEYHYDSTAGEGVTAYVIDTGIDIDHQDFGGRAKWGVNTVDNMDEDCNGHGTHVSGTTAGTTFGVAKKANLIAVKVLDCNGSGSNSGVIMGMEWATEHAQQTGADKSVMNMSLGGGFSQATNQAAAAIVQAGVFLAVAAGNDNRDARSFSPASEPSVCTVAASTRQDGKASFSNFGQVVDVYGPGAEIISARVGGGSMTLSGTSMASPHVAGLGAYLIGLGRGSGGSLCNTIKEMAQPVIRSPGSNTTNRLIYNGSGQ*

>FOXT_09594_gene_FOXG_09594_foxy_supercontig_2.12:complement(join(153392..153403153568..153951154006..154329154663..155877155971..156030))

MLSLWWNETCRRTDDAKLDFLPEISVQKKSNDFPTLHDGEPPFAVWKVAKSIFDALLECKSCSCPSDHEFKAKLELGTYRSREKKPVLKPSRRPNRKHDGDSDMGSFEMDMFLCMEHDWHEFRIQAAKETIVRFSGHEEASPCSERKTRSSKRIERLCKPIIEIRTRPLQRLLVRLKSGHLFEVRPEKSNFQIDKTTEPISLSGCFDERKDFFTEKTKRILSLIIGYTVLHLYGTSWLQPGWGSSNIKFFQTTACKTPLRPFVEAQLRKTDDPGDYEDEWTEEFDSGHSCPEMVALAVVLMEIYFVKPFRQLAEMHNIPLIDTRSGRITLMDVDQVFWGDEEGEEGWRTQIPEDSPLLEAIDNCLDGEIWEDNEGDRLDVEELRTRIYEKVVRPLELHLRHGFSQIQLDGVDQYAKSLDFGKWGQYRTWRSEYEDVYKTFVGDYINQKPTQPVKIAILDTGIDRDHPFLELYEDNMKGKKNFHNASQKNVPDTNGHGTFAANLILDYARDAHLYIIKIADKKNATPDAAIVANAIYHAVDKWAVDIISMSFGWPSSDMVGHDALEDAIDHAYSKKVLMFAAASNSGARLGRAYPASNPHVICVHSTDTNGEASGFSPTAEPNSINLATVGESVESAWPTLLSQDSRCLQSRSGTSYATPIMFAQA

>FOXT_09801_gene_FOXG_09801_foxy_supercontig_2.12:complement(join(696540..697136697189..697530697580..697882))

MVFLGKILPLALAALSVNGAEILSAPGAENIPNGYIVVMKEGTSTQDFDAHREWVASVHHERLARRGSTNVGGMRHTYNFNQGFMGYAGTFDEETIQEIANRDDVAYIERDQIMKASAIQTQRNVPSWGLARVSSRQPGGRDYSYDSTAGQGVTAYIIDTGIDIRHTDFGGRAVWGTNTVDRRNEDCNGHGTHVAGTTGGTSFGVAKRARLVAVKVLDCNGSGSNSAVIAGMQWAMQHASQNDPRRAVANMSLGGGYSQATNQAAAAIVRAGIFLAVAAGNDNRDARSFSPASEPTVCTAAASHVRDGKASFSNWGQLVDVYAPGQDIISARPGGGSRSLSGTSMASPHVCGLGAYLIGLGRGSGGGLCDTIKRMALPVISNPGSGTTNRLINNGVSQ*

>FOXT_10424

KVEPVGWLSIALQPDIDNLKTNLDCTSGKYTQNHLALALRDPDQKDVGQVLGWLDWIHVVKAAEDLLDTKIGFYRTKQYSVPESVADAVNFIHPIANFTPDCLREQYIRFGIAGFLEENANYQDSNDFLFSVQLIGANSQEAALDVQYGMALGYPTYYLADGRNEPLEFLDYLLDLSDDEIPHVLSISYGDNEVVPYAERVCSLFGLLTARGTTIVAASGDGGAKCRTNDGTMSVFPATCPWVTSVGGFEGSGGGFSQYFPREKWQSIKSYVHYNASNRGVPDISISAFITRLKGLRGTSASAPVVAAMLALVNDARVRKGKEVLGWLNEVLYLQDVTNGSCGWPAAKGWDAITGLGVPFQKL

>FOXT_12083_gene_FOXG_12083_foxy_supercontig_2.16:complement(join(554659..555281555332..555425555480..556118556345..558231))

MASDMEEEYANSLDEFESALIQDPLDDEDGVYRYQHLEEAYDTRLEKARGWIREEQDFTSEAQADDFFHTFSDITAKSSTKAAGNLLHVLVEVVKHNGLQPEKIERLARRLVEEAPDLLQYKNKDGQTPILMAIRTRQDQLLDYMISACVSHKKPRDSAQNLNTALCSKHDGKPCLHAAFGEKLKPETMKMLIENASDEALSMTDHTGKTPMHHAVQFRDCTDSRSALIDLMIQRDFMARLNKPKSAKTFLDISDQNGCSVFQEHANTRKTYMEQYRTAQAAQRRKKESKEAQKEAERAPPREPRLHANTRESKTIPTVKAPGDRDAERYGRSSGPAASTDDRERIRQQKKEEERARLEQAGAPAINDRLRDRDATERDGSRFRSPRTNGLEANDTDALYPSRHTEPTSNTGIKRSNTARPEREAESEKRVVKPSSSSKKNPDFQSALKNRVKNSDMVLQKLKLYYMRTRNAEMVMFFLYGKNMDDIQIGFDYRGLPCPIPWKEFTKRFGENAVDGYKFDPVLQYATFPRVEVLVKGRSTDRQDKLSHQAKATLPGANSRKDVKYFLDWLYKKGVRHIIKLSVEDSGDSGQKVHSDEVIQSALEKFVIEHLDWQKTDLDPETILHISSKRLTVEKTYDSPHWINQRIMGFERRLNKNRESPHEAQLRISDSLSFSGNKSGHIRVKLVDPATGSTLGVASNIVSPTTISTPDQGVNEDRWLRSIENFASAMAPYWEDTVTNFLETRQNSGTTERVESDVVIALIDDGVDKFEIGRPDQVLEGKSFDFHDERVNPPYLSAQGHGTTMASMILRVCPMAKVYPIRLKTYNSADGKSTIDPRYAARAIQAALDKNATIISMSWTLPIESGKDHMKEELHAVLKRAVENKVLMFCSAPDKGKFTELDYPSGPWPNSFFRIGAAYSNGTVFQWTPEVGITYILPGVDVVQDQIKGGSSKSGEGSKSGKNLTGSSVATALGAGLAAMIIYCVKASILSVKTANQNKAAIHPIPDDRAIQVAKPDAMKRAFASLGSTTSNKFIQVWEELDKARVILERWEREKSIPEANLKCTQEFMQFGIKLASSVKQ

>FOXT_12263_gene_FOXG_12263_foxy_supercontig_2.16:complement(join(1011536..10122551012309..10124941012555..1012839))

MVFLGKILPLALAALSVNGAEILSAPGAENIPNGYIVVMKEGTSTQDFDAHREWVASVHHERLARRGSTNVGGMRHTYNFNQGFMGYAGTFDEETIQEIANRDDVAYIERDQIMKASAIQTQRNVPSWGLARVSSRQPGGRDYSYDSTAGQGVTAYIIDTGIDIRHTDFGGRAVWGTNTVDRRNEDCNGHGTHVAGTTGGTSFGVAKRARLVAVKVLDCNGSGSNSAVIAGMQWAMQHASQNDPRRAVANMSLGGGYSQASNQAAAAIVRAGIFLAVAAGNDNRDARSFSPASEPTVCTAAASHVRDGKASFSNWGQLVDVYAPGQDIISARPGGGSRSLSGTSMASPHVCGLGAYLIGLGRGSGGGLCDTIKRMALPVISNPGSGTTNRLINNGVSQ*

>FOXT_13463_gene_FOXG_13463_foxy_supercontig_2.19:join(1015679..10159961016064..1016990)

MPAILFRASAVAFAALIACTDASKLLGFENKRNIIPNSYIVALKEDVPEHDFDAHMAWVSNVHSASVAAAGSASTSGVKFTFKINGWKGYSGSFDDNTLNELLANENVDYIEPDRLSRIASVESQALVTQRNAPTWGLGRISHKQRGSRDYVYDDSAGEGVVVYSIDTGIDITHPDFEGRAFWGINTVDNIDRDGHGHGTHTSSTIAGKTYGVAKKAKIFAAKVYDSRGIGPDSATLKAIEWAIDHAQKNNHTGKAAMNLSLVTDSPRAVNAVCTRAVEAGIFVAVAAGNDNRAVTNESPASADKVCTAGATAESDSKASFSNYGRLVALYAPGQSIRAAVPGNRSGIKSGTSMAAPHVCGVGAALMSLENIRPQDVCNRLKALAHPSVRNPGPNTTNKLLYNNSGR*

>FOXT_14511_gene_FOXG_14511_foxy_supercontig_2.23:87800..89773

MSAPTINGNPFSVSSLPDDPQSQQRSGENRLNHLPENTNYIVLETQDPLKRPEKHTLKDLHVDLQEYLGGNTWLCRYEPTDIKSLRDLAFVKDAKPLHPELKLEPALKSSDGAARTVDIVLQSRADDSAEDISQRIQDSLGISPEDITIRDGNSIIRLTGNNDKLQEIAKIDSVGSIQQVHKLTFFNNVAREILHADVTGSSPNTTIFKGANQIVTVADTGFDTGDKNKTHEAFTGRVQKLIPIGRSSKTNDPDGHGTHVCGSVLGDGHSEKMGGPIQGTAPEATLIVQSLLDSRGGLFGRSEKTLGDLLREALDHQSFIHTNSWGPVWTEQLTYNNASTDLDNFVSTHQEMTVCFAAGNDGGEKTDLGHIGAQAAAKNCIAVGSSDNRRKAKNNRFDAFKADGVVEGDPNNISWFSSRGPTLERRIKPDVVAPGTMILSAKSRDAKPSDQFGRSTDPAWTFSSGTSMATPLVAGCVAVLRETLQKDGVASPSAALLKALLVNGAVSTGKKVEAQGFGRVDLANSVILKGITTNKDYLEGELLDEDGQDEFVKTLTLGDLLMPDGPDSEAVTLKVTMVYSDLPGAALQNNINLQVAVGDAPSRFGNLGDRPDDVNNVEQVVWSGIPADDRETKITLNVSAPRTMCGDKQAFALVWSVN

>FOXT_14564

YIFFKGWPVHVQVVDKLRGITGHGIKVAVVDTGHPALGGCFGNCLVSFGTDLVPDPMDCHGSHVAGIVAGAAPGVTLGAYRVFGCDVLIAFNQAYQDGADIITASIGAVAVSRIVVPCTVSAGNGDVGFYASTAANGNKVMAIASYDNGGCVLVRGASTYSSWGPTLDVKPQFGSPGGILSTYPGTSMACPAGVIALSNPAPVPQQGGGLIQAYDALLLSNDTLHNGKTDLDLSISHIPTSVGAGLALWSGYIPYQGGKLPAGKYKIAYRALRIFGDW

>FOXT_17011_gene_FOXG_17011_foxy_supercontig_2.40:join(187839..188135188187..189074)

MAAIFKASLVAFATLASCTDASKLLGFENKRHIIPNSYIVALKEGLQERDFETHMAWVSDVHSANVALAGGASTSGVKHTFKINGWKGYSGSFDENTLHELITNENVDYIEPDRMSHIASLDRKFALETQRNAPSWGLGRVSHRQGNSRDYVYDSTAGEGVTVYSIDTGIDIKHPDFEGRASWGINTVDDIDEDGHGHGTHTSSTIVGKTYGVAKKAKIIAAKVYDARGRGPDSATLKAIEWAVDHAQKNNHTGKAAMNLSLVTDSPRAVNAVCTKAVEAGIFLAVAAGNDNRAVTNESPASADKVCTVSATRLGDQKAGFSNYGRLVALYAPGQSITAAFPNGRTGTISGTSMAAPHVCGVGATIMALEGVTPQKLCDRLKQLAHPSVRNPGPNTTNKLLYNGSGQ*

>fsol_36288_gene_e_gw1.2.723.1_fsol_sca_2_chr3_3_0:join(3197010..31971863197233..31977243197962..31983183198376..3199029)

MESEKKDGADSNHKSSSKVDEATVKAVERFLKLHYLRSRSYGVAMEILYGRNTTSGQILQELYFDLTGDVNSGTITQAGLENLLSKLKFEDILQYVAIPKLSVQVNRAGERSGSRRSAKPDGAGRRDLCYVFDRLRKKGVKTILKVFIDDSSMPAHSDEAIEDALKSMDVEVWDWNKTDLCTEVIYKVAPKAREVHLYWSGNNAVLRGWSEEGGLKKLRSLKTHEWIQCMKEFRRLLFDAERYYDRGKIEESIEEPIKIALIDDGVDIKDLEYSFIGGRTFCKRDPEHNLNDPYYVSSTGHGTIMAKQIHLLCPRAQFYVLRLEDHPSDESARQITAKSAAQAILAAVRKKVHIISMSWTIDPPEDEEERRALESAITKAASADILMFCSASDQGAKHVATYPSKATPKIFTIGAATASGTVDSWVGNINNISFTFPGTKVELDGGPIDTAVKEVTGSSVATALAAGLAALILYCVQVRILLATDPVEKQKARRDFQSLQKHESMMRAFKDIGTTEESNHKFIAVWEVFGKRVEEKERVDQEDWINLIAKVGTTLCMKV*

>fsol_39203

YIFFKGWPVHIMTVDKLRGATGKGIKVALVDSGDYTHPALGGCFGGCLVSFGTDLVPDPQDCHGTHTAGTLAGAAPGVTIGAYRAFGCDILIAFNRAFEDGADIISASIGAVAVSRIVVPCILSAGNGEAGFYANTAGNGKHVTAVASFDNDACVLIRGASAFTSWGPTMDVKPQFGAPGGILSTYPGTSMATPAGIVALSKPAPVPQQGSGIVQAHDAIILSNDTLKNGSEEVEFQISHVPASISPGLPVWSGYVPYQGGELPAGMYVVRYRALRIFGDW

>fsol_39895

FIVFHGWPVHMITVDKLRGYTGNGIRIAVVDSGDYTHPALGGCFGGCLIAYGRDLVPDPFDDCHGTHVAGTIAGAAPDVTLGMYRAWGCDIMLAFNAAFEDGSDIISYSAGAIAASRIAVSIMVAPGNGSSGFLTASPATGVNVNAIGSVQNDACVLVRGGSISSSWGPTGESKPQFTAPGGILSTYPGTSMATPAAIFALSKAPVAQQGSGLIQAYDATVLSNDTIKNGDKDVTYKLGHKKATVPAGLPVYSGLIPYMGGQLPEGRYKVVFSALRVFGDW

>fsol_40446_gene_e_gw1.12.475.1_fsol_sca_12_chr5_2_0:complement(join(41039..4173741787..4228442342..4248842539..4309643270..44631))

MSDAEDRDPQRDSDDDEEETDEEDDLVKLAISDLKKTFDSEADEEHVDKFFRHHGETVKHFSEDGVTFLHRIVQLVKDKAVNAQHVRPSVERIVAQYPDHLKTRNDDGQTPLYQAIHLRRYTWKLVGFMLNSCSDPKCIEDALESPCGEGDSAKTCLTLAFEKDLRLKALQILVQFASERALEAKDGSGRTPFHWAVQYSQCTDERVDVIKLLLEKDREAVTKLRETASLRPVDTFLDIKYVRREDSIEYSVYGEYERTAEVYLAEELARKEREARVREAKVQEAEDERAESKTSTVNVTVRERESPKAGLRGKDPKAQIGPERDSGWKRPERDHDRRKPDADMLDERERLRQQLKEEEKEEQDRRAREERNWRDQAPEREVSSTRHHAKNERRFDHATGVPRVETGFTGGDNAPNTPLKRVATGRLGASDDKRRREKKAVSSSKTSSKKPNPKICFDYEGLPSEIQAHVFNERFGKDRNSGIQFDEVLMYVRFPDVTVIHSGRRAPKPRALGRQDMEFFFDWLYAKGVRRILKLEVEDSGKIPHSDEAIQISLDKIMIEHLDWQKSDLDPRVICNISSKADRSSVSSDDSDDGAKNILREVTLKWSGNNAVLRAWSEPEGLPQLQKLEVINLCIPPHSDLYDTRSWVQKNLEEFRARLNKNVNLIRNVTTADAVSAGPFEETHSSTPAVIAREGKKGTEIQVSSGSSKTSARTNPVTEHQWLTCMERFSGCMGRFWKDTIETSRERLGQGASAVDGQAAADLERLSKDVVVALIDDGVDSCDPAFSGRAIEGKTFDYQDGGVGQYYISAKGHGTEMARMILKVCPMASIYSIRLKTHISPEKGHSTIDAASAAFAIEAALEKKASIISMSWTIPVPEDGSKEKQLLDAVLERACRQKVLMFCSSSDQINATEHYPSAYKRQRFFLIGAAHDDGSAYGHAGKDNDFIFPGVSVNTNGGNSLPLYLADKTSSTKESTGSSIATALAAGLAAMITYCFKASALGIVSARTQQGKDYISGSELVKPGDVDRIAEHEVLKAAFGRFGNMENGQFIPVWNRFGPASDVLEGEIKYESKLTCVMNLYSNLIER*

>fsol_46676

FIVFNGWPVHVMTVDKLRGVTGKGIRVAIIDSGDYTHPALGGCFGGCLVEAGWDFTPDPMDDCHGTHVAGTVAGAAPGVKLAAYRAWGCEILLAFIRAFEEGADIISCSDGSVLATRIVVPVVISAGNGGLGFYASAPATGRGVTGVGAVTNNACVLLEGGGDLSSWGPTLAMNPQIAAPGAILSTFPGTSMSAPAGIYALAKAPAPQQGAGIAQAFDAVELSNDTVKNGASDVALQLSHRKANVPAGLPVYSGHIPYLGGLLDEGVYSLRVSALRVFGDW

>fsol_51284_gene_e_gw1.52.158.1_fsol_sca_52_chr10_4_0:complement(join(68959..6922169278..6947869535..6967069723..7006170112..70420))

MAAIFKASLVAFATLASCTDASKLLGFENKRHIIPNSYIVAMKEGLPERDFETHMAWVSDVHSANVALAGGASTSGVKHTFKINGWKGYSGSFDENTLHELITNENVDYIEPDRMSHIASLDRKFALETQRNAPSWGLGRVSHRQGNSRDYVYDSTAGEGVTVYSIDTGIDIKHPDFEGRASWGINTVDDIDEDGHGHGTHTSSTIVGKTYGVAKKAKIIAAKVYDARGRGPDSATLKAIEWAVDHAQRNNHTGKAAMNLSLVTDSPRAVNAVCTKAVEAGIFLAVAAGNDNRAVTNESPASADKVCTVGATRLGDQKAGFSNYGRLVALYAPGQSITAAFPNGRTGTISGTSMAAPHVCGVGATIMALEGVTPQKLCDRLKQLAHPSVRNPGPNTTNKLLYNGSGQ*

>fsol_52137_gene_e_gw1.32.182.1_fsol_sca_32_chr11_3_0:complement(join(44167..4442944480..4468044739..4487444942..4531045358..45666))

MGFFRTLFSFSIFALSLADTSKFIGLDDVDNIIPNSYIVVMKGAVTEAEFKDHQVWASRIHRRSKRDGAADGLDGLKTTFDFQGFKAYCGTFDKESIERITRSSDVDYVEADRVVKMAALNTQRNAPSWGLGRISHKKAGSFDYVYDSDAGSGITIYGVDTGIDIHHPDFGGRATWGVNTVDSENSDQNGHGTHTAGTFAGATYGVAKKARIIAVKVLNAEGTGSTSGVIQGIEWSTNHASSNGLSGKAAMNLSLGVRSSSVFNSAAEAAQRSGIFLAVAAGNDGFSPASARGVCTVAATDAQDQATSWSNYGSTVALYAPGDKILSIYPNGGTATLSGTSMASPHVCGVGAYLMALEGIGPGRVCDRIKQLALESVKNPGPDTTRRLLYNGSGA*

>fsol_52284

FIIFKGWQVHHMTVDKLHGVLGKGAVVAVVDTGAYNHPAVSRRFGGFKIEGGYDFVPDPMDNHGTHVSGIIAGVAPDATLRVYKVFAKDVLIAFLKAYDDGADIITASIGAVVASRIVVVVTISAGNGQAGFAASTGSSGAHVLAISSIDGEACVLVRGASYFTSLGATLFIKPDVAAPGGILSSYLGTSMACPAGIAALGEAASVAQVGTGIVNATKVTSLSNDTITNGDKATTYDLSVENYTIQPGLPLYSGKIPYMGGRLAEGRYKMRFAALKPFAGW

>fsol_54608_gene_e_gw1.28.155.1_fsol_sca_28_chr13_5_0:complement(join(565574..566275566322..566879566944..567129567176..567244567301..567357567408..567577567636..569010))

MTVDPDPNQVPGGLRRSQTVDYESDIDNDDEFLPPALVTAGFDDEDHVKVRFDQDIEDALSFVQSLASRKPTKEEKSQQLLEFVDSRKMEWHKTTREGQNFLHVLAYCNSIRKPATSLQWLMSRAMLRLPHLIGSMDKTKRTPLTVALSNGNEVFSYAACLNLKPGTRQRFKEPLESECENQGSDREVTCLHTALTCAFNKEDLRQDIVKIMCSFVPDKMFTVTDHKGRTPLHLAVEYERCCKAQVGIVEELLRWGPQALDVQIQAALYSNQTYSVYQYHVHTQRQAIEESKKKTSQSAPRKEDDNEARTSSNTKKPMSRPEKTSMGPPPVPRDRDRDRDRTEPKVGIARAHTGAPSQEKPGSYVVAANQERSAASYTTAYTQEKLTPLPSPSLHPVGSSKASAYSSAPKQDATDPYSPEKERERQDAAADISHQLKHLYLRTQKPENASRCLSVGGLGKELWFDFGPPKKLTRADFRRHFGHLRFDTILQYVAFPQIELDDHKQIPDRRYAGKEDMTFFFDWLKQQGVSRIIKVIVDDLKSPSHSDAAIEKALKPFNVEILDWRRLDLDPVSLSEVGQSLREVTLHWSGRNSVLRAWSEKEGLALIPTLEVINLVQTEGLESSERTRRNLDAFEQRLHESWPKDKPKPRVDRPKTGGGRALVRGLSVETLSDRQERSVDPHRWMQCMENFAEHFRQIPALRDKVTDPSLEPVEVALIDDGADITRPDLSDLKGKKFPGKSFCYYQEGTTWRVSPYWDSSSGHGTLMARLIHKICPSAVIHVIKLQTFEVENSNKLQINPDSAIKAIEYAAERGSQIICMSWTIKPPEGDKKKEFDDAIHNALNSKGVLMFCAASDQGKSADLTYPHGSNRASFRIGAAKATGSMSDTVGDAHDLDFIFPGHQVVVNSSDDVYDKDLQKFEAHSGSSVANALAAGLAALVIECVRLGVFYTNENKQIDPTSAIRKDDLVKIRDRNQMKYALSWIGTNRNTDNKYIEVWDTFNAVAEKLRQNEGSRIDRLENIATLARFFLKKGVNYGE*

>fsol_58928_gene_estExfsol_Genewise1.C_sca_2_chr3_3_00462_fsol_sca_2_chr3_3_0:complement(join(991312..992340992392..992709))

MGFFRILFSLSLCALSLAIPSKLIGLENTKDVIPNSYIVVMKSAVSEAEFQSHQAWASKIHSRSLGKRDGVLDDFGGLKATFQFEGLKGYSGAFDKKTIELITRNPAVDYVEVDRVVKLDAISTQRDAPSWGLGRISHRRVGSADYVFDDSAGSGITIYGVDTGIDIRHPEFGGRAAWGTNTVDDEDTDQNGHGTHTAGTFGGATYGIAKKANIVAVKVLNAQGTGTTSGVIQGIQWCTDHAGRNGLRGKAAMNLSLGIRGSTIFNRVAEAAQASGIFLAVAAGNDGTDAGQFSPASARGVCTAAATNSQDAATSWSNYGSVVAVYGPGADIVSAYPNDDTATLSGTSMASPHVCGVGAYLMALEGIGPDTVCDRIKQLASESVTNQKPNTTKKLLYNGSGA*

>fsol_61473_gene_estExfsol_Genewise1.C_sca_5_chr5_3_01309_fsol_sca_5_chr5_3_0:complement(join(2289735..22909612291018..2291383))

MGFFRHLFSLSLCALSLAIPSKLIGLENAQDVIPNSYIVVMKSTVSEAEFQTHQAWASRIHRRSLGERDETLGGLDGLKATFEFEGLKGYSGAFDKKTIELITRNPAVDYVEVDRRVKLDAITTQRNAPSWGLGRISHKRAGSSDFVFDDSAGDGITIYGVDTGIDINHPEFSGRATWGTNTVDSEDTDQNGHGTHTAGTFAGATYGIAKKAKVIAVKVLNAQGTGSTSGVIQGIQWCTDHAGRNGLRGKAAMNLSLGIRGSTVFNRAAEAAQQSGIFLAVAAGNDGFSPASARGVCTAAATNSQDAATSWSNYGSVVAVYGPGADIVSAYPNEDTATLSGTSMASPHVCGVGAYLMALEGIGPDKVCDRIKELALESVTNQKPNTTRKLLYNGSGA*

>fsol_76110_gene_fgenesh1_pg.sca_18_chr2_1_0000301_fsol_sca_18_chr2_1_0:join(855893..856138856189..856652856745..857860857914..858208858317..858946)

MDDDDTDAPSLLQSVLALFSGNRPQRSAKPASLQSRKRQAWLRADSFMQRDDVREADPASVKRTLRAFEHLVQDDPHREMDFSPEQNFEQDGNERFPNLRRMVENPSRDPHADLGAAIRVGKRGRECDDAIQELSQLEQDRKRRRVLPREEPTRESSLDDPDANFAVLMRRHLVSLDNALRRHWVCVCQKCSGLSVRLSLPQHKQGANLESEASFQVFFGVRSVPASILQEARITVKRSSDPILSNQPAFAHHILCQSITESIGQRNCLHFALEDGVFQRLRPQQKTFGSDRMSSTVSLSALFQRQQELPGGKSVLPFKGRAVLAATLASALLPFLETPWLQFSFNHSKIQFFEPRQNGELPDITKPFLALEHVPVMPARSTTDTGDASKHIFHPNASVLALGILLCELHFCTPVELMAEETPAAGVPGARNINDDCYTCLDKLQILEDDAGVDYYLATKACLTGEYYPIGEQVEFDDVIVQRLFYQNVVKRLENVIFKAWGIRLGDLGSSDARSNESCWGPIGREVVRLHAGQVDSQMANDASARGTWHRSMSTDSAPVSYLHSDMVLRLSGQPSPGPQAQGHLAKPSGKSLHFFDASLHMGPGPESENPLSERWMDNLLSSISRYVNPVLDPVEPVRIAILDSGLDTTNPFLIEDQQRANPQIKEALSFVHGTQPHEIRDEIGHGTHALGLLLKVAPCAEIYVARAIDHAVKQWKVDIISMSFGIREYNEPMSNAIANALNERTLLFAAASNDGANLDRAFPAQYPGVFCIHSTDGNGNPSDFNPTASEKDVNYSLLGQQVSSHWPAGMNGHNQPVRSLSGTSVATPIAAGLAASVLSFVRRQDRQVAVESERLGQWLKRDNSMDMVFNSMVRRRRGPGYDYITPEILFDSGSTEEDVYRKIRDIKRTMYRYQK*

>fsol_764

FQGWPAHNDTVAAMHGHFGEDVVIAIVDSGDYTHPAFGGGFGGYRVEAGYDLVPDPMDCHGTHVAGIAAGVAPKARLRAYKVFGCDTIVAFIKAYEDGADVINASLGALIASTIAVLVVFAAGNGATGFYTSSGGNGVGSLAVGSVQAMVIPGKNDFSSWGPTARMKPEISAPGGILSTWPGTSMASPAGVGALGRLASIVRQGAGIVDAASVTSVSNDTLTNNDETVTYKVTHETLGAGLPVYGGRITYMGFPDGEYRLLGRAVRTFGDW

>fsol_76658_gene_fgenesh1_pg.sca_18_chr2_1_0000849_fsol_sca_18_chr2_1_0:join(2789529..27897532789811..27902892790336..27912142791265..27913362791386..27917192791775..27921902792469..27924722792698..27)

MASSNTIINFAVDVTERLATVARGVGSDDKLFGPALAAALWLVNQHLKEQKQSDSVLGTQARLLKLLSNLERICKPPTGPSPSRANHDLANRYPGLSLLRQNNRKNAVDGIRKLASRQSPEGRKKQQDLLRILEAFIKPLTVADETPNPKTKAEVWVGDDFTGSMRTLYKVLSSYIFCNAGSEQTQIAGRLRLALESGTEGDSPAFDLMFLAHPHHELHEEAFRWRETRIAVDRRKAKFAVAGDEDVKLHIDGPTHIADFCERISTREQYQLSLKVSGKQLHFVEWCEGARSWVPNTPSVSLSTILRNHKLSQKMKYLLSYLLAKSVWQFYSTDWMGKEWTNESIHFMFERRQGAKNAGIYLNEPFISARFDPDSSSNDAEFRPHKFPKIKALGIVLLEIELGTVIEDHYDEECYASDGELNSDAELYAALKLFDDPDRLEDTFPLLKTVIGDCLRPSKFMQHRQSAEELRKVLQDDVVDHLHTLIKLYGRPEQIALKPTVQMQTSQIRQITQPPHSAPLLQQPQVSRNVPILEQQAVNAKSVAFNGAVASSITQASSKAWFEELDQLNEVLSSLPNEIDQTYKPVRVAVIDTGINGSDIYAKHIRDYRDFVTNKDDIKQDNTGHGTNSVKLVYKVCADAEVYVARVFEYDEADDDTQDLMLKAIEHAKNVWNVDIISIASGFERDHALMRRAIKRAASDGTLVFAAASNYGNIRQVTFPARMQDVICVYCTDGRAKVSQSINPAAQTTKSKNFAILGEGVSVPPSIREQVTGTSVATSIAAGLAGRLLDFSRQKDCQQRIRWVTSVESQVIFMLLS*

>fsol_77026_gene_fgenesh1_pg.sca_26_chr2_2_0000102_fsol_sca_26_chr2_2_0:join(322595..323286323343..324404324456..324792324875..325504)

MPREPGSPNQDDFHAALTISLQESLKTAKEVIPVFISGLNRTPLLRSWLSLLQLKLNELPSPYDLLALNTQALAKLSTRLKELLCQLDGLTYQDSSENLKPLLAVTSRPDINLDRAAQDESQILETCELLQLAIDRAWPRSAAGVGDLIMDQNAAMDQVEDVLREAKNANQVCSRLEKLYELLSKKLRGCKSSEHVAKFHLSGLDDGIIGFLFSRCGGTQGWHPMPCKIHHGKTNHHVHNNGPSGRPTMIPFRGCKDFRHAETLNKTLNKSLTLSLSKGQEILTGSVPKSQLAREYGTVPEKFLTLKEVLGDNKPDQTARATTKAGWWYTSLMARLSQTPEQELNDGELDYKSSFTKNVATVQYLLSESVLHLYNSPWLLDIWQPDHIEFPKDGTLLNFRRPYYPSSLQVDSSQESIPEKVDVPDPYDHAEMFMAKFGLLILQLQLQQAFPLEMEDQSDDIWPLIALGRYYDDFKESIEPIKEVVDACLDFRRHLFEELDAQDESDAFRFRMVFYKLILMPLRVILQLNFPDVAKDIVCNTIPQEEDQETLNTQPREGATAIELNGVSSRAAFDPQVLGETMLKRPSPASSTEWFSNLDVLNEYLTAQTCEGSENYDMSRVRVAVIDSGLREERQGDAYITYQNFIETWNDAHKDNPQHGTNSVDLVRKVYGRADIYVAKVFQGDEADGNTSKYMAEAIQWAINKKVDIISISAGFKEECHEELEAQIKMATAGGEAPEILVFAAASNWQNINGVAYPASMTDRVIGIFCCNGGLKSSRQWNPNPRNHAANFAMLGEDVALDSSARLLGGTSVSTALAAGLAAKLLDFSRQPDIQTWMSKANREKMKTKAGMSAILKEMSRNNVNEGYECIAPWEILPADAASGIASREKVREAVCTIIKKAMKKV*

>fsol_77386_gene_fgenesh1_pg.sca_2_chr3_3_0000108_fsol_sca_2_chr3_3_0:complement(join(282983..283582283641..284013284081..284649284707..285203285264..286416))

MATGAQPTDEGPSAPLPTSDFVNSHFFDEYFAPPSFNHDVSPKAVPEITTAQRREDLFDHVMNKISSAGTSTTIERLKQDLLKTFFDQITACKYGVEDEERTILHWIAHKIKFERIRESDPKFKAAGLLAEIATSMDPKMLSLRDTEKKSTPIHMLISCAKSSETGIGEIILAMCRAAEKDSPEGKKYASEAISMQNKDNENCLHMAIKKELSITKDLITLASAQAVVAVCSIDGNTPLHDAVGPNRFAYRKQVCGANPRPCEKCQAIQAQMDRSKGRVMKDIVALIVKNPKALRSKNKEDLSPYLLHKQSREPVRPAAPTNKSSLTLMDKDDSIRGLGDTGKQKCKTVPSNDLDEEIERYLVESAFDLGGFEEACDCFFGDKTHLKGKDSVFRPERPLSGLDSDNMYPFFNCGTTLAQVDLSIDPTDSKGSAASNDTIQVEDARIALSRIFEMLKKKNVKRILKLKVRDNTKTPCSDDFIENCLRPFDIRYLDWNKPDLCADVVIRSTPRVTGLWLYATGNNAVLRSWAGSNGLSNLLQLRSVHVQSKVGLESYSNHLAAVGAFKRDLRASIEVVRKLKIMAMGQMQHQTIMGQYKSYSDNARQHLQEVQSPLEPSQDKQLWQARLEWAEAAMAKGEAIGTKLTAGVTMLLQRKEANLQDIINRLGLQQQPQDQKKAPTDEIDISELQQWGIQSLGVLESTTNFVALRFRAESKIDQPVTGSGHKLTKKSSDKYDDSSCKPHQWVESLERFVSDHVQRSDRGARVKVGLIDDGVDVHLGDLKDKVKAGWPTEKPTSSRVPFYQSAEGHGTTMARLIATACPHVDLYVAKLESLNRVILRDEPGKLIDNANRKDWISTADEAASAIKWAREQKVDIISMSWSFIATTQNQAHLERLTQELQSAANEHIILYCAAADQGMYGSRKSLYPANAGANIKVVGSATETGHSSSFVNQGLVDYLFPGEEIKGIGNRKGSSAATALAAGFAALVIWCHEVHFKSNPEKRAWIKKAERMDSLFQGMQNLATKPDAPQSGWVDASLVLDEGDIKQVIDFVDREVKRGVSSK*

>fsol_77513_gene_fgenesh1_pg.sca_2_chr3_3_0000235_fsol_sca_2_chr3_3_0:complement(join(656925..656971657103..657607657657..658643))

MIYNEDENPIDTAMKLIPPLIESIATSNKLKSLALFNSRADLDFQLRLALLELGNSRSLPQNNEPLSKFAFYLLKVLWDFDGKDNDGRLKKEVPHRRSRSALQQDFCNEARSPKQSPIRLPETAEELRVLAQQISSLRKPISSRPDVPLDPEVSHDRTNTHHRTTIREGHDVNKVYEREKESEFHSFETPKAELLSNDRGPNYSNVKVNIVPQPETDEQRWGVNFMDQAYAFYEKKIKPLKKKRPIKVAVLDTGVKKKTNHFLAVRLEGGTPIKDQKSFIGDDQNDTDDDDWHGTQVAALVVDIAPHVDLYIAKVSKGLEESDEGQFAEAIEWAIKEKVDIINISAALPDNVETRKAIDEAEAQGIIVLAAASNRGANESRVFPARMDTVLAIHATDGNGNPCGFNPSPLEKAMNFSTLGTAIRSPLGDGTFVPSGTSFSTPIAAGMAANILTLVEEYFNESHNDGRTWYLAHRRTGMGAIFKELSVNRHNYDYLCPILVSNAHRVFHWGAL*

>fsol_78688

FSGWAAHYSTVDKLHGIRGKGATVAIIDTGDYTHKALGGCFGGCKIRGGYDLVPDPMDYHGTHVAGIIAGVAPDAELLIYKETIMALCDAYSAGADIITSSIGAVLASRLVIVVVASAGNGEIGFYASSGATGHGVLAVAAVYFTTWGPTLLIKPDITAPGFIVSTVLGTSMAAPAGLAALGSSASPFQVGTGLVDAWKVTQLDRDTITNDKKGHRYTFKLEPQFVQPGLPLYSGKVPYGGGKLAPGNYTMRFAALRPYGHW

>fsol_80887_gene_fgenesh1_pg.sca_5_chr5_3_0000686_fsol_sca_5_chr5_3_0:complement(join(2358455..23591742359249..23596032359669..23607902360849..2361945))

MPFNTQPFARGIEHTVEWSLISDLRQMSDRFGPPGAVGNTRSSTLTLKLRNAINDLELQIRRTFTILQFDLLLQLQKETQNYAPKPRMHKDSRQNSWGDDSDDVSSESSTEGHLEDWSSDGSQQGFGPAQPSVEEARSRLERLCEFLEARVSPNLRDNKANEYPRLTSLIESVKSQPDSSPLDLIPTVRPTPTPFLFNITEDEATARAFLAFVDPAQGKPSKEIRRQMITDAKEMAQFFLSFNSFVDALQMSTGTLDVLTWEPTTRRLASDESVASFSLLRKFQHQTEAACRAVLSHIASCSHPKHEALLQLPGWEEVSNWDSTKATESLPVPLFFTMCLLDKKTQKKQEHGPLDSWQYARMFFLQPDHPGNYTERTLCEALRLSHRQRIDLEFYVSEHHRPAIPVTIPIHIPSKLQRTRKYGKDFPRHSLWDLIEQGRLKERNQQNQQHQRMSFNEWVTVSRPGNIVGMHERKALAVKLVLGLMLSLDSDYVFETWDPKQVHLLESVNTYTPFVSLPSKSDLSGQEKTLSLSHFPSHGSIDDLDIPEPSPQFVFLAKALVQIAQGERLTAYEQDPCTDPWEACNQIRDDIREYSQMATCGAEVDREVFPFFNAALGCLEFHEKFPCRLMANQSSHNKMEVAWKLVFDTILVKIDNNLTLESMIAPSSPPLAQDPSLSRSSFPRHEISQQSLSIGGTLQNFSHTTATEMALSGQPSLPSVELGQPNVQLFDAKQVARPSTANKFWERLDTFHKSYARFVTDRKTQSGDETPRRIRIAVIDTGVDFGHPGITVAKDKGRMKKEWCHSWVGDDAKDEDNELHGSNCAHLLHKSAPEADIYVAKVFNQNNVRDYEAENIAKAIDHAVTKWDVDIISMSFGLNRPAARDDGDVEKELLALKKYNDIVEEIEKSIRKASPRLIFAAASNNGKNDRRAFPASDNPYVTCVHASEGNGADGGINPEIGSGFNFMTLGMGLDLMEKEKILDLVKKDKMEKQGRDPVRYKQVVKSGTSFATPIAAGIAATVLDLADRVDAINKRAKEKLRSPEGMEKMLKLMSTPKDVRDCMCYMAPWNHWTGGWELEDSEIKLAWASINVQFSQY*

>fsol_81544

VVEPDGWLSIAMQPDIDNLKTRLRDTSGKFTQNHLALALRDPDQADIDEVLTWLDWIHVIEAAEDLLEMKIGFYRTREYSVPESVADAISFIYPIANFVPECLHKLYIRLGIAGFLEEYANFQDSDEFLYSVELIGGNSQEAALDVQYAMALGYPIYYLAGGRNEPLEFLDYLLDLSDDEIPHVLSISYADNEVVPYAERVCSLFGLLTARGTSVLAASGDGGAKCHTNDGAMSVFPATCPWVTSIGGPVGSGGGFSQYFLREKWQDIESYVFYNASYRAVPDISAVSFITKVAGVRGTSASTPVVAAMIALINDARVRKGKDVLGWLNEVLYLQDITGGSCGWPAAKGYDAITGLGVPFEKL

>fsol_84201

FIVFQAWPVHEVTVDELHGIKGKGQRVCVVDSGDASHPVLSGRIAGGKNMQDCHGTFVSSVIVGVAPEAEVYMYKVFGCDIVLGMLAADADDCDIVSLSLGSRVASEIRLVIIAAGNGEQGFYASSPASGRGVVSVASVNSDSCVLIKGFSAFSSWGLTNDFSPSIAAPGGVYGAFPGTSFSTPSGLAALAMGASLAQQGAGLVDAVLVSNDTLRNGSEAVTYQVSHVAATIAAGGALWSGKIPYMG

>fsol_85630_gene_fgenesh1_pg.sca_82_chr10_2_0000233_fsol_sca_82_chr10_2_0:join(618554..619040619099..619273619342..620632620699..621126621473..622158622212..622600622645..622792622839..623614)

MPSKHRVLAELKRALGAFTGRRQAQQLKEVETCDDASQPLEPGEEDSFPAQVLSQTLRSFAEVDVAQLAISHGGAVPPNVEQQMLQIIENHDSGLEAQNNTSPLPSLQALGEYNKVFDMLDDLEPANVSPALTPEQDHSESQLRSEELLDPRVQNCLQFLFTGLTKQLGPCDLSAESKHRHTVSLHLTGFRDQIEDQSHRVFGFYLSSRLDWIPCRWLESREILEAAKPGCGLIRDCEEAKKRLCILLEERQGGRLLPQPDEADCYASISTPTMSLGQLLDTPGLNRQFPSADNPNRFLPLDRATLCLNLALSLLQLSQDEWRQIVWCPENIFFLKNPLSGVVEEKTKPYLSWVVMSTPSVQDNARDMGELACDSHLLHFGRLLMEIHAFERIEEPVHGLELQTTLLMAIQDERSYLPQDSAFVAAVKACLSLEGKIAAAGGGGSSQIRDFIYRNIVVNLGCSVAGKERPQNLSRCSALSKHLAVNSVCQETMLDYDSKLGKTPITIKLVSPLLMIMYGQTSGLTSFSSSTTARNFLKQMDDWLELNIKPLRPMKPPENNTHSQAKIKIAIIDSGASMIDDRIRAAVRGKKIKGGRNFLPSADPHDWDDDCGHGTIVTRLLLKYAPNADIYVAKVTEGSRIDSKGLFCVSQAINWAVQNWQVDIISMSFVVEHEHPGIEEELTQAIDPSYEGPGISRKIVFAAAGNRGGNSLMGWPARRNGVIAIHATDGLGSPVNINANPDHMGDNFATLGQDIEIEWTPEGKQHKEKIYVTGTSFATPIAVAIAANVLEFSSLGRKDIWRYNQVTGSDGFGLPAKAPALLGETTDGDSSSLAYHGDEACMAAGQVRRSCVSFSTPWSSISHISLVDLLCLPRRHHREDQARNMDRREQARISEISCEFEELFTRYVHGSRSHISPGDLLPLPRRHPLEDQARNIDRQEHTRISDISRECDELFMRYVHGSRPQQFAFQSSQQRFWAWWHAFNSFSRPRLPLNFLANEGENAATQQRVELLLGVLRLNLKLALQPTQDSEKVGVSDEIDPLGTLDIALFGIDGSLKRLEKIVSAIHRGAAGSLQERIFAYAGEKRDLIFEEYVNHQVRCSSPGLRKLLFASILYRHYRILHERRTGQRQQRRSDSEEKRFQSKKDSYMRDKRRYIKIHPSRGLEMSITPKPLPSLRNRKTPFARFVKRHIPPPCLKENSGDNTRSHVDQDLQPYFCISQHCAKNLLFFANSTSWVLHMRQVHTTMWVRHLHNPVLWKCALPHDGKVLIEHGTEEGLLEHMKTEHKGLFKSEDHLRSLASSSGVPLPQPLNICPICDYNHLDSRTWVGGDPTQQSEVGTKEHGENSRNAAIQFTLPQVSSSATEALRDHQQTERCISKHLRSLAYYFCHYLIDSRDDRDPDIDNKDKRYPDKGTMLEVWLNEARSDGPFNPWVFEPRKGPSFASTYSVDQVSRAFNNSK*

>fsol_86785_gene_fgenesh1_pg.sca_48_chr11_4_0000006_fsol_sca_48_chr11_4_0:complement(join(19184..1979219843..2126221320..2219822243..22457))

MPKNEAQQPLCMSATRPECEFSRRRNSEKLIFRSDLKAYRCMQEDAKEKYWLSGASLICVSLSFPSKTPTKLGPSLVSVTESPGWNGLKDLGACVSGRLGQYSRPGMPDHRPGKRDNDASIIEIYLGPIIRANYSVTDLTQEAFKRMDDTALNLLRSLNSIVNLQLITTERPPIDIHDVPGTTHAEDNHLAELRSSVGRMARLGAGFEQLHHLTSFLVSPSTLGDQRELSLGTGKYPLFTLPPGDTGDLAIGCLRRWHEILKTLPGGSPTFEDLSFYPSEATEESTENYEWNERRGEHVGSIVKVITDEFRRLSCVKERRHEILLQVSNDLPATRPDNPPKLDMFISRCPTSNLIWQESQCGDFDAKISGKEKCMCEGIKQAMKDRKKLHLLVDHRGVIDVTDSVKPAPLRRDSFDGISLSELLRQDVFGPIDTQAWFNNTAARKVNSATKAQVALGLSRCLMDFFDKGLELASHSWTAENVHFRESSDSNREGMQRLLYVSLRPKLDQARSADINKMLDNDDINKMFDSDNPVVLSFAKLLLEIFDGKAISIQRIPTGEEKLWNWLDLGDVVENLLQDQSRGSFAFKYLEVVESCLRLRATLRDCKSRSDLVATSRFVRKTIYENVVRKLEVLASWERKIHGQRKRKNQDPVLDEPPTKKLAVLPQSPDAPRAPGQAGGHDPGLSASNSGECTAVEEEFTEHTEEGEGGPSLYDDQGEADQSDRVRAKEYLRKLSQGFNRRIKPLKDSAANEIDPIKVAIIDSGVDLDDPVIRARSCQIGDKRNWTSDQPDACADDCGHGTHVTRLILEAAPAVKVYVAKVTERKKLDSKVSGRIRQAIEWATGVWEVDIISLSFAMDGEDETIRATLDKVLNPPRHITKNVIVIAAASNWGGNRHIGFPACYEDIICVHSTDGYGNPSKTNPTARKGKDFSTLGMSIKSSFKGKGKDKRRSEVYISGTSYATAIGAGITANILDFARRDPKLRDEEKWWLFSSCGMSCVLRSMSEERGGYRYVMPWALFDGRDEKDIWRDIRGALKSR*

>fsol_86790_gene_fgenesh1_pg.sca_48_chr11_4_0000011_fsol_sca_48_chr11_4_0:join(35696..3773137805..3819838257..38886)

MRVRDTLGSTQVLEPDPAWSSELLKGVKLSVDAMAISPEPKDGAGNEALLLDTVGTVLQMAKIARMNETNEEVKVFYSHLSLHCFRLQEHLTRARQASSRLNGELVSQALSHLGSLVPESGLHELPTYPVTPDFRLKRLYLHWEEARRRHDRTELDFIYRWLKPGVSEAQRHEVDKALEACADELDKQQPEQPSLKSLHKYASTEITEPPYAVCKAAKSIYDALINCKGCSCPDPHDFKAKLELGTYRSPAKKQVVKPARRRARRGRGEDDASGGIELDMFLSMERDWHEFRVQTVKERAERAVRFGPSVDDAPCRGGKIVEQYAKVERLCRPIIKTKTKALQRLVLRLNSGELFEIGFEKSNFQIDHNAEPISLSQCFEDRQDFFTEKTKRILSLIIGYTVLHLHSTSWLQPGWGSSNIKFFQTTSCKTPLRPFIEAQLLKAGPESFGDDLEDEDDELDSGHCCPEVVALAVVLMEVYFAKPFKRLAAMHDIQLIETKSGRITLMDVDQVFEGDEENEEGWRSQIPEDSPLLEAIDNCLDAELWEDEEGQPLDSATLRSLIYQKVVRPLELDLTNGFRNIQLDEVDQYARNLDFGKWGQPMFTQESDGRTGLLCPREVLPVRSPSPGPFALLSPGQIAIGPDGWKYMHHASRLHSFASISTPDTDSMSSSPFDYKAFQRLRLRMFDIYDTAADVTAHRTDKYIRWKSEYEQVYKKFVGNYLQNPPSQPVKIAVLDTGIDRDHFVFEAREENLKGKKNCYNESQKNVPDTHGHGTFTANLILDYAQDASLYVIKIAGKENTRPDAKIVANAINHAIDKWDVDIISMSFGWPSSDFDGYDALQTAIDKAMSRKVLMFAAAANSGGRLGRAYPASSSQVICVHSTNTNGSASDFSPTAEPNAINIATVGESVQSAWPMLLCQNNNTGYMMSRSGTSYATPIVAGMAAFLLQYARLHLSEGEALALKRREKMEALLRRCAVRGPSYQPRDGYFFVHLSLDKHNLFGGELDWVSYEISRALKT*

>fsol_869

FYGWPNHNATVWALHGIKGKGVKIAVVDTGDYTHPDLGGGFGGFKVAGGHDFAPDPMGLHGTHVAGIVAGVAPEATIYAYKTYAAINIAFLAAYEAGVDIITASIGEEVATRLAVVITVSVGNGEGGYRALSPSGAKGVLAVSNIDTSWGPLLQMKPDVGAPGAIYSTVFGTSMATPAGVAALVRKASVAQVGSGLINATSLTDLMNDTVINSKKPVEYTFTKEDSTLQPGMPLYSGNIPFVGGKLARGEYVLRFAVLVPFGDW

>fsol_86938_gene_fgenesh1_pg.sca_39_chr11_5_0000069_fsol_sca_39_chr11_5_0:join(212658..212930212977..213162213209..213381213428..213956)

MGFFRHLFSLSLCALSLAIPSKLIGLENAQDVIPNSYIVVMKSTVSEAEFQTHQAWASRIHRRSLGERDETLGGLDGLKATFEFEGLKGYSGAFDKKTIELITRNPAVDYVEVDRRVKLDAITTQRNAPSWGLGRISHKRAGSSDFVFDDSAGDGITIYGVDTGIDINHPEFSGRATWGTNTVDSEDTDQNGHGTHTAGTFAGATYGIAKKAKVIAVKVLNAQGTGSTSGVIQGIQWCTDHAGRNGLRGKAAMNLSLGIRGSTVFNRAAEAAQQSGIFLAVAAGNDGFSPASARGVCTAAATNSQDAATSWSNYGSVVAVYGPGADIVSAYPNEDTATLSGTSMASPHVCGVGAYLMALEGIGPDKVCDRIKELALESVTNQKPNTTRKLLYNGSGA*

>fsol_95099_gene_fgenesh1_pm.sca_9_chr7_10_0000199_fsol_sca_9_chr7_10_0:complement(join(1246050..12469431247002..12472351247281..1247307))

MGFFRHLFSLSLCALSLAIPSKLIGLENTQDVIPNSYIVVMKSTISEAEFQTHQAWASKIHRRNLGERDETLGGLDGLKTTFEFEGLKGYSGAFDKRTIELISRNPAVDYVEVDRVVKLDAITTQRNAPSWGLGRISHKSAGSSDFVFDDSAGSGITIYGVDTGIDIKHPEFGGRATWGTNTVDNEDTDQNGHGTHTAGTFAGATYGIAKKANVIAVKVLNAQGTGSTSGVIQGIQWCTDHAGRNGLRGKAAMNLSLGIRGSTVFNRVAEAAQQSGIFLAVAAGNDGFSPASARGVCTAAATNSQDAATSWSNYGAVVAVYGPGADIVSAYPNEDTATLSGTSMASPHVCGVGAYLMALEGIGPDKVCDRIKELAVESVTNQKPNTTRKLLYNGSGA*

>fsol_96635

RDYDYAPANRLGEGQLGALDDHHVFRKRIADPIFTEQWHLYNPIQLGHDVNVTGLWLDGITGQNVTVAVVDDGLDMNSDDLKPNYFAAGSWDFNDNDPVPAPELDDDRHGTRCAGEVAAANDVCGVGVAYDSKVAGLRILSKLISDADEAEALMYKYDDNHIYSCSWGPSDDGQTMEAPDVVIRRAMLKAIQEGRGGLGSIYVFASGNGAGAGDNCNFDGYTNSIYSITVGAVDRTGQHPYYSEECSAQLVVTYSSGSGIHTTDVCYKAHGGTSAAAPLAAGIFALVLQVRPDLTWRDLQYLAMDTALPEANQQDTAIGFSHTFGYGKIDSWALVEKAKWELVKPQTWYFSVTFEVTQDMLKDANARLEHVTVTMNVEHQRRGDLSVDLISPKNVSHLAVSRERDAKAEGYVDWTFMSVVHWGESGAGKWTIIVRDDWRLKLWGEAIDADKARRRLRYEFLYDAF

>FVET_00027_gene_FVEG_00027_fver_supercontig_3.1:join(61549..6185161899..6223762284..6241962472..6267262728..62987)

MGYFASVFLLLITALPAARAGEMIQAAAGTPGIIPDAYIVVMNDGISDSDFESHRGWAASMHNKAVQKRGRIFSGMTRMWNATELKGYSGSFDRQTIQQIANHSSATGIIFLTICWSGITIYGVDTGIDIQHPEFQGRAIWGTNQVDNVDRDQNGHGTHTAGTFAGTTFGVAKRATIVAVKVLDAQGGGRASSIISGINWCVSHARQNNLLGRAVMNLSLGGSGTRSFNQVATNAANAGIFLSVAAGNDAEDAANTSPASARGVCTVAASTEQDTRADFSNFGQIVDIYAPGDRIISAFPNNSRQVLSGTSMAAPHVAGVGAYLMALEGISATQVCDRLKRLSQPSIRNPGPRTTNRLLYNNSGV*

>FVET_00212_gene_FVEG_00212_fver_supercontig_3.1:join(551500..551865551917..553143)

MGFLSSAILLLITAFPAAQAGEMINAAAGATDVIPDSYIVVMNEGISESDFESHRTWATGMNSKSRKRAGAFSGVSRTWSATGMKGYSGSFARETIEQIANNSAVAYVEPDRMVNITAFVTQRNAPSYGLGRISNKRPGNRDYIFDESAGRGITIYGVDTGIDIRHPEFEGRATWGTNEINDVNQDENGHGTHTAGTFAGRNFGVAKRANIVAVKVLNAEGSGSTSGIISGINWCVDHARRNNILGRAVMNLSLGGTGARAFNQVATNAANAGIFLAVAAGNDGEDAANTSPASARGVCTVSASTERDTRADFSNFGSVVDIYAPGDQIPSVFPNNARRVLSGTSMAAPHVAGVGAYLMALEGISSGQVCNRIKRLSQPRIRNPGRDTTNRLLYNNSGV*

>FVET_00372_gene_FVEG_00370_fver_supercontig_3.1:join(1013096..10134041013456..1014394)

MGFLSSAILLLITAFPAAQAGEMINAAAGATDVIPDSYIVVMNEGISESDFESHRTWATSMNSKSRKRAGAFSGVSRTWSATGMKGYSGSFARETIEQIANNSAVAYVEPDRMVNITAFVTQRNAPSYGLGRISNKRPGNRDYIFDESAGRGITIYGVDTGIDIRHPEFEGRATWGTNEINDVNQDENGHGTHTAGTFAGRNFGVAKRANIVAVKVLNAEGSGSTSGIISGINWCVDHARRNNILGRAVMNLSLGGTGARAFNQVATNAANAGIFLAVAAGNDGEDAANTSPASARGVCTVSASTERDTRADFSNFGSVVDIYAPGDQIPSVFPNNARRVLSGTSMAAPHVAGVGAYLMALEGISSGQVCNRIKRLSQPRIRNPGRDTTNRLLYNNSGV*

>FVET_01532_gene_FVEG_01530_fver_supercontig_3.2:complement(join(35707..3665836877..36902))

MALFKVILTAFAALSAVDAARLLSSPNDKDVVPNSYLVVMKDSVTSAEFDSHVTWATDLHGESVSRRGADGLNGFKYSYKINGWHAYSGSFHQETLDEILNNDKVDFVEHDRYVYISGFVTQKDAPSWGLGRVSHRHNGTRDYVYDERAGKGITFYGVDTGIDIHHPDFGGRAVWGTNVVNGTKDNDRQGHGTHTAATATGTKYGLAKNANVVAVKALNDYGAGLWSNIMKALEWCVEDATKKNIIGKAVLNLSISGGKVVAANQAVTKTAKAGIFVSVAAGNDNQDATNKSPASAEGVCCAAATTIRDDKAKFSNYGSVVKIYAPGQGITSATPNNSTGVMSGTSMAAPHVGGVGATLMASKGIAPADLCAELIKVASGPVQNPGSSTTNKLLYNGSGQ*

>FVET_02136_gene_FVEG_02133_fver_supercontig_3.2:join(1841180..18414881841541..1842560)

MGLFTVVFTAIAALSAVDAAELLRSPNSKDIVPNSYLVVMKDSVSSADLDSHVSWVTDLHSESITKPGVKNLDGFKHSYKINGWHAYSGSFDSETLASILDNDQVDFVEHDRYVYIDGLVTQKDAPSWGLGRVSHRMNGTRDYVYDETAGSGITFYGVDTGIDIRHPDFGGRAVWGTNVVSGTGDNDRHGHGTHTAATATGTKYGLAKKANVVAVKALNDHGAGLWSNIMKALEWCVDDARKKNALGKAVLNLSISGGKVVAANQAITNAANAGIFVSVAAGNDNQDATNKSPASAENVCCAAASTIRDEKASISNYGSVVKLYAPGQGITSATPNNSTGVMTGTSMAAPHVGGVGATLMASKHIAPSAVCAELIKMATGAVRNPGANTTNKLLYNGSGQ*

>FVET_03249_gene_FVEG_03245_fver_supercontig_3.3:complement(join(2133827..21343752134421..21347712134819..2135106))

MGLFKVIFTAVAALSAVDAAELLSPANSKDIIPNFYLVVMKDSVSSAELDSHVSWVTDLHREGIAKRGTENLGGFRHSYKINGWHAYSGSFDSETLASILGDDKVDFVEHDRHVYISGFVTQKDAPSWGLGRVSHRMDSTRDYVYDENAGSGITFYGVDTGIDIHHPDFGGRAVWGINVVNGTKDNDRHGHGTHTAATAAGIEYGLAKKANVVAVKALNDYGAGLWSNIMKALEWCVNDAREKEILGKAVLNLSISGGKVVAANQAITNAAKAGIFVSVAAGNDNQDATNKSPASAENVCCVAATTIKDDKAKFSNYGSVVKLYAPGQGITSATPNNQTGVMSGTSMAAPHVGGVGATLMASKGIAPSAVCAELIKMASGPVLNPGANTTNKLLYNGSGK*

>FVET_03382_gene_FVEG_03378_fver_supercontig_3.3:join(2501577..25036162503691..2503705)

MASSISINGVSVELNHDCDGSDTIYIILRTKGMPLNKKQKTKLLELGVHVNEFVGDEKQQIYLCGFHQDSLDKVQDLGFVEYAGEYVEEFALTKKVQQDAKGQTCNVAIMLHQDVEEITEELTEKIAEAANVDPSAIVVEDRGLQVKVATDKLDGIAALDEVRVLHTANEAALFDTKARQILRVDEALAPKSHTETQNIVYRGEGQIVCVADTGLDRGSKTNVHEAFSGRVKELFAWGRPETNLADDLDGHGTHVCGSVLGSGQHQSHGLVEGVAPGAELLVQSLFSKFNPLNNAPRLDGLPKTNLGPLFQQAYDAGARIHTNSWGSPLPMSRIQRPYDGRSESIDQFVHENQDMTILFAAGNDGQDADLDGKLDGAINERSLGAEAAAKNCITVGATENDRPDLASSDSKRPYTYGGFWTQRFAVNPLRDDHMANNPDGLAAFSSRGPTAENRLKPDIVAPGTAILSARSQNKKYLGGVHLTGESGDSKYMYLAGTSMATPLVAGCCAVLRQALIANGYQDEQDGVKNPTGSLIKALLINGAAPVGGQYMPDGVNEGYNAHSGFGRVDLSASIPGINDAYSGYGIGVVDEDDDDPFEYTVNIPKPLEGDNGSLTLKVTMVYADRPGGKLQHDLNLVVASGELERHGNQVNKSFPLGAAEGFDRRNNVEQVVWHHVPVGSVILTL

>FVET_03498

IHEPGDYLRIYLADLEKPYLKVSDPNSKQYGQYLLRSMLPDKSLASTKVAGWLERLDVIADWNRVLNTTFHRFRTTKYSIPSTQQDTISYIFPTIHFCPSYLRTKYSTFAIAGFLNNFPNITDVRAFLINTVSVKGPPVEAELDLDYSMAFTGPTFYSVGGHNEPAEFFDYVLGLKSPPKVISISYNDDEKVPYAQHVCDLFAKAAARGISIIGSSGDGGASCLGVSGFVPTFPSSCPWMTSVGAASSGGMSNIFKRPSWQAVSGYILYNVSGRAQPDVSLLGYLTLTGGHDGTSASAPVFAAMVALTNDIRLREKKPALGFLNPLLYFRDIKDGGCGWEALAGWDAATGLGEPFTKL

>FVET_03649

RDYDYTPASRLGEGQLGALDDHHVFRKRIQDPIFKEQWHLLNPTQVGHDVNVTGLWLDGITGKNVTVAVIDDGLDMHSDDLKPNYFAAGSWDFNDNDPEPAPVLDEDRHGTRCAGEVAAANDVCGIGVAYDSKVAGLRILSKLISDADEAEAMMYKYDDNHIYSCSWGPSDDGQTMEAPDVVIRRAMLKAIQKGRRGLGSIYVFASGNGAGQGDNCNFDGYTNSIYSITIGAVDRTGQHPYYAEECSAQLVVTYSSGSGIHTTDVCYKAHGGTSAAAPLAAGIFALALQVRPELTWRDLQYIAMDTAIPESNQQNTTIGFSHVFGYGKIDSWALVERAKWSLVKPQSWYFSVTLEVTEDMLKDSNARVEHITVTMNVEHTRRGDLSVDLISPDNVSHLAVARRGDAKEEGYIDWTFMSVAHWGESGVGKWTIVVRDDWRLKLWGEAIEADKARRRLRYEFLYDAF

>FVET_03935_gene_FVEG_03930_fver_supercontig_3.4:complement(join(1364693..13648591364918..13651131365166..13654681365526..1366185))

MSLFKVIFTAVAALSAVDAAELQSSANSKDIIPNSYLVVMKDSVSSAELDSHVSWVTDLHHEGMAKRGAKNLGGFKHSYKISGWHAYSGSFDSETLARILDDDKVDFVEHDRHVYISGFVTQKDAPSWGLGRVSHRMNGTRDYVYDESAGSGITFYGIDTGIDIHHPDFGGRAVWGINVVNGTKDNDRHGHGTHTAATAAGTKYGLAKKANVVAVKALNDYGAGLWSNIMKALEWCVNDAREKKILGKAVLNLSISGGKVVAANQAITNAAKAGIFVSVASGNDNQDATNKSPASAENVCCVAATTIRDEKAKFSNYGSVVKLYAPGQGITSATPNNQTGVMSGTSMAAPHVGGVGATLMASKGIALSAVCAELIKMASEPVLNPGANTTNKLLYNGSGR*

>FVET_05568

YIFFKGWPVHVMTIDKLRGITGKGLKVALVDSGDYKHPALGGCFGDCLVSFGTDLVPDPMDCHGTHVAGILAGAAPGVQIGAYRAFGCDVLIAFNQAFEDGADIISASIGAVAVSRIVVPCVLAAGNGAMGFYASTAANGKKVTAVGSYDNDGCVLLRGASTFSSWGPTMDVKPQFGAPGGILSTYPGTSMASPAGIFALANPAPVPQQGGGIVQAHDAVLLSNDTVKNGKKQIDLQISHVPATIDAGLPVWSGYVPYQGGELPAGQYVVRYRALRIFGDW

>FVET_08694_gene_FVEG_08679_fver_supercontig_3.11:join(1039736..10400381040090..10404311040484..1041080)

MSLFKVIFTAVAALSAVDAAELQSSANSKDIIPNSYLVVMKDSVSSAELDSHVSWVTDLHHEGMAKRGAKNLGGFKHSYKISGWHAYSGSFDSETLARILDDDKVDFVEHDRHVYISGFVTQKDAPSWGLGRVSHRMNGTRDYVYDESAGSGITFYGIDTGIDIHHPDFGGRAVWGINVVNGTKDNDRHGHGTHTAATAAGTKYGLAKKANVVAVKALNDYGAGLWSNIMKALEWCVNDAREKKILGKAVLNLSISGGKVVAANQAITNAAKAGIFVSVASGNDNQDATNKSPASAENVCCVAATTIRDEKAKFSNYGSVVKLYAPGQGITSATPNNQTGVMSGTSMAAPHVGGVGATLMASKGIAPSAVCAELIKMASEPVLNPGANTTNKLLYNGSGR*

>FVET_09088

KVEPVGWLSIALQPDIDNLKTSLDCTSGKYTQNHLAFALRDPDQKDVGQVLGWLDWIHVVKAAEDLLNTKIGFYRTNQYSVPESVANAVNFIHPIANFTPDCLREQYIRLGIAGFLEENANYEDSNDFLFSVQLIGANSQEAALDVQYGMALGYPTYYLADGRNEPLEFLDYLLDLSDDEIPHVLSVSYGDNEVVPYAERVCSLFGLLTARGTTIVAASGDGGAKCRTNDGTMSVFPATCPWVTSVGGFEGSGGGFSQYFPREKWQSIKSYVHYNASNRGVPDISISAFITRLKGLRGTSASAPVVAAMLALVNDARVRKGKEVLGWLNEVLYLQDVTKGSCGWPAAKGWDAITGLGVPFQKL

>FVET_10880_gene_FVEG_10863_fver_supercontig_3.15:join(1050938..10512311051292..10514771051531..1052253)

MGFIKSVAAAFAALSVVDAAQLLGASSKDIIPNSYIVVMKDSVSSTEFDSHVSGVTNLHHEHLSKRGSTNFGGLKHMYSINGWQGYSGSFSRDTINEILKDDNVDYVEHDRRAKILGWASQPNAPSWGLGRVSHRERGNSTLVYDEMAGEGITFYGVDTGIDITHPDFGGRGVLGTNVVGGAHLDGHGHGTHTAGTVAGNAYGIAKKASIVSVKVLNNRGSGSWSGIIAGLNWCVTHARENNVLGKAVMNLSIGGGRMTSVNQAATNAANAGIFLAVAAGNCNTDARNISPASAENVCTVAASTEFDRKASFSNYGATIEIYAPGNNIISTVPGNRSRAMSGTSMAAPHVAGVAAAIMASQNIAPSEVCAHLAGMAEGKITNPGRSTTNKLLYNGSGE*

>FVET_11234_gene_FVEG_11217_fver_supercontig_3.16:complement(join(737437..738117738171..738767738825..739351739401..740302740447..740655))

MTKVAFTTDVKGVVSDDDDEDYENDDVEEEPAKIPNHDEVLFAFESITNEARNKKLNLGKRESRESFLQNKTPLHIAIAKKNHAFIEVVLDEKKAIDDLDSLLRITCEHRRNCIHTAIYHNLQPQYTTRLVKRSSEETLKAQDQSGLTPLHLAVDYKYASEGQLRIVEALLAHGGSALDEYTKEPQNLSVYEYQDYTQRLWRKQFDVPLSARVGERKQDESRDEPQGQESEHAGTRTKGGLEHVGGRNRISQQGKDGHGGLHRTPDNSHAPRSEQTEPRTSSESVRHAAANRNSPIEAPEGFKPLQRRQTNIDDQLVKQAQEEKRKSEYAEKIRQTVKLHYLRTTLTINSKPASRDQLKAVKFLHGANLNNINLCFDYSEAPPEIYEDSFKQSYDHINFDQVLRYVSFRRIELQKPPVSVVKARLTRNRGQTSNQGRGRDDLTVFFNWLKGKGVKHILKVMVDDLRDPSHSDKSLEDCLRPFEVEILDWTKVDLCPETILTACRNVRQLYLRWSGNRAVLRAWSEPEGLAKLEKLEAVHLVYSEDQALESADRITMYVNDFEERLNESAAANPKESVSTDGDDAEQARRILVFRCKADVPGSERIVREGASKAQSGINEMSLQSNRWLNCMDKFADELQNTCSEFVKPQNSIKVALIDDGADPYVESLRGKIWGGETFSRGFPHENGPSPYYRSTKGHGTVMADMICRVCPMARLYVYKLETQTSLNLATQTQGKEYIAAESAALAVRAAIDQKVDIISMSWTVKETVENRDGVNTFRQAIKDALDAGILLFCAAADTGAITEVEYPWSFDCQRIFRIGAATADGRVWGPTGNPQNLSFILPGHKVVSRNPHREGALPDDFEERTGSSVATALAAGLAALILHCVNLAVVHGKEHPSTTAVSAEDLERLANHDDMYNVLRGIGLDEGQQRFIEVWRRFDRPAKELKEPMSDKMDALGIVARLARDLVPSGSS

>FVET_11645_gene_FVEG_11628_fver_supercontig_3.17:complement(join(800319..800936801031..801403801457..801518))

MEASQSLQFFDASHQMASLQEKALSNRWIDTMAMKIYEYVDRHNQETQSAAPGVDEPVRIAILDSGFDPNLQSVRTNEGFLDPRIKGFKNFVAGQNELECRDEIGHGTHALGLLLKVATTCTEIYIARIANQGTLGRDSYSAISKAINHAVSEWKVDVISMSFGIREFDEGMMKEIQNARMNRTLMFAAASNDGANHGRAFPAKDSGVFCIHSSDANGRPSGFNPIAEEDDVNFCFLGENVESHWPVGIGGHNEDMRVMSGTSVATPIAAGAAASLLSLVRQHEKDVPFDSDRLGWWLKDLDFMKAVLKSMRKGNKYDSYDYIPPLFLSNMGSSREDVYNRIKQIRGEMLQ

>FVET_13379_gene_FVEG_13360_fver_supercontig_3.23:110500..112461

MSGPTINGNTFSVSSLPDGPEQSGEGEMRVNRLPENTNYIVLETQDPLKRPEKRTLQDLHVELHEYLGGNTWLCRYEPTDIKSLRDLAFVKDAKPLQPELKLQPALKDDTAESRTVDIILQNRADDSAEDISQRIQDSLGISPEDITIRDGASVIRLTGNNDKLQEIAKIDSVGSIQEVHKLTFFNNVAREILHADVTGPNTTIFKGAGQTVTVADTGFDTGDRNSTHEAFTGRVKKLIPVGRSTMTNDLDGHGTHVCGSILGDGHSEKMGGPIQGTAPEATLIVQSLLDNRGGLFGRSGKTLTDLLNEAIEHQSFIHTNSWGPVWTRQLEYNNASTGLDTFVSNHPQMTVCFAAGNDGDEPTRLGHIGAQAAAKNCIAVGSCDNQRKAKDKTFEAFDADGVVEGNPNNISHFSSRGPTLERRIKPDVVAPGAMILSAMSRDAKPDDRFGRSSDPAWMFLSGTSMATPLVAGCVAVLRETLQKDGVASPSAALLKALLVNGAVNTGKKAEAQGFGRVDLANSIILKGITTNKGYLEGELVDNDDEDEFIKTLTLGDLLMPDGPDSETVTLKVTMVYSDLPGAALQNNINLQVAVGDAPSRFGNLGDRPDDVNNVEQVIWSGIPAERDTKITLSVTEPRTLLGHKQAFALVWSVI

>FVET_13831_gene_FVEG_13811_fver_supercontig_3.24:join(549428..549721549771..550658)

MRFMKAVAFAFELLSAVNAARLLDVANKNDIVPDSYIVVLKKSVSSLDFDSHLAWAANIHHENLSKRGSMTAGGLRHVYRINGWYGYSGSFDRETLGAILENDDVDYVEPDRHVSLNALVTQPNAPSWGLGRISHRQRGSPDFVYDDTAGQGITFYGVDTGIDIRHPDFGGRAVWGTNTAGGSNTDGHGHGTHTAGTVAGATYGIAKKARIVAVKVLNDRGAGQWSGIIGGMNWAVNHARQNRVLGKAVMNMSLGGGLSSAVNQAATNTQNAGIFLAVAAGNDNRDAANTSPASAQGVCTVAASTEQDSKASFSNWGRTVEIYAPGTNIISTMPGGRAGRMSGTSMAAPHVAGAGAALMAMEGTRASDVCSRLIQLAQGRISNPGTGTTNKLLYNGSGR*

>FVET_13954_gene_FVEG_13934_fver_supercontig_3.25:complement(join(263158..264084264136..264453))

MSIMKIATLCFAALSAVEAAKLLTPSDKRDIVPDSYIVVMKDNVSPLKFDSHMSWATNVHHANLARQGSTATGGLKHVYRIDGWQGYSGSFARETIDRILENDDVDYVEPDRRVHLTGLITQPNAPSWGLGRISHRNNGNSSFIYDDRAGEGITFYGVDTGIDINHPDFGGRAVWGTNTAGGSDSDGHGHGTHTAGTVAGASYGIAKKAKIVAVKVLSDGGTGQWSGIIEGINWSVNHARANNALGKAVMNMSLGGGLSTSVNQATTRAQRAGIFIAVAAGNEDSDAANTSPASAEDVCTVAASTEQDGRASFSNWGSMVEIYAPGTNIVSTTPGGNTGKMSGTSMAAPHVAGVGAAIMATEGISPSEVCSRLVEIGLEQISNPGSGTTNKLLYNNSGR*

>gi|116766025|gb|ABK27194.1|_subtilisinlike_protease_Epichloe_festucae

MKGVLSLSLLPLLAAPSPILVDTIHRDAAPILSSHNSKEVPDSYIVVFKKNVSPASAAAHQVWVQDLHTTVMAKRSLRKRNQFPFKNDAFDGLKHTYDIAGSIMGYSGHFDEEVIEQVRRHPDVQYIEKDSEVHAWDEPVTENNAPWGLARVSHRDSLTMGTFNKYLYAANGGEGVDVYVIDTGTNIEHVDFEGRAHWGKTIPTGDDDVDGNGHGTHCSGTVAGKKYGVAKKANVYAVKVLRSNGSGSMSDVVKGVEWAAGAHLSKMVEARKKGNKAFKGSAANMSLGGGKSFTLDLAVNAAVDAGIHFAVAAGNDNADACNYSPAAAEKAVTVGASTLADERAYFSNYGKCTDIFAPGLNILSTWIGSKYAVNTISGTSMASPHVAGLLAYFLSLQPEQDSAFAVSPISPAKLKKDMIAIATKNALTDIPADTPNILAWNGGGSSNYTAIIQQGGYEATRPGNKAAQLTEKIEKLGQNTASQLGAIYSEIKDAFTI*

>gi|170674474|gb|ACB30118.1|_PrtI_Epichloe_festucae

MKSVLSLSLLPLLAVASPISVDTIHKDAAPILSSTNSKEVPDSYIIVFNNDVSPASVAAHHTWVQDLHTTVMAKRSLTKRNQFPFKNDVFDGLKHTFDIAGSLLGYSGQFDEEVIEQVRRHPDVKYIEKDSEVHALETPQTQTNAPWGLARISHRDSLSFGTFNKYLYAATGGEGVDVYVIDTGTNTEHVDFEGRAHWGKTIPAGDEDVDGNGHGTHCSGTVAGKKYGVAKKANVYAVKVLRSNGSGTMSDVVKGVEWAAASHAQKMAAAKKDGKKLKGSAANMSLGGAVNAAVDAGIHFAVAAGNDNADSCNYSPAAAEKAVTVGASTLADERAYFSNYGKCNDIFAPGLNILSTWIGSKYAVNTISGTSMASPHVAGLLAYFLSLQPDQDSAFAVEPLSPAKMKKNMISIGTQNALTDVPSDTANILAWNGGGSSNYTDIIKKGGYQAATLNGKAAHLAEKIEKFEKYATKELGAIYSEIKDAFTL*

>gi|170674478|gb|ACB30120.1|_subtilisinlike_protease_Epichloe_festucae

MPEDIVINGKAIATAHKHQHKDASSSDHILLRTTGEPLNKAQKHELKALGVHIHEFVGNESQQLYLCGYEKDSLVKIRGLDFIEYANVYSNDLCVPDVVQTATNQTDSTMEFDILMHQDVEDISDELIAKISQAADVNQDAISVDSSMVRVKVDAEALEKLAALDEVRVIHPVNERALFTNVARRLLGSCDMMSENGTIYKGQGQTICVADTGFDNGTITDVHDAFTGRVQQLYSWGRAQENNSSDPDGHGTHVCGSVLGRGQHNSEGAIEGTAPSASLIVQSMFVRFNWKGESILGGYPSDLGQLFDQGYQAGARVHTNSWGTPPPPTNVQRPYDTSSEGIDRYVWNHQDMTVLFAAGNDGQDTNLDGQVNERSLGAEASAKNCITVGASENLRPGLLSGKTGGPYTYGAFWPRKFSQNPLKDDHQADNPDGLAAFSSRGPSAENRLKPDVVAPGTAILSAKSRKMRSGSGVDRTGVSSDNRYLYLSGTSMATPLVAGCCAAIRESLLNNGYTDETDGITNPTAALVKALLINGAVPVSGHYMPSHISQEPNPHSGFGRVNLAHSIAIVEPSITTSGYGIGVIDEETEEPFEIEIPIPSSGGKDLTFKVTMAYADLPGASLANDLNLVVVADGKQRHGNQGTQEFDVDVKQTFDRSNNVEQVVWHQIGGDKVKVIVKHYRLLSPRVPFAYAWAFI

>gi|170674484|gb|ACB30123.1|_subtilisinlike_protease_Epichloe_festucae

MKGLLALSIFPLLAASSPLVIDSIHQDAAPVLSSVNSKHVEDSYIIVFKKHVTSASAAAHQSWVQNLHTTALAKRSQLNKRNQFPFKNDVFSGLKHTYDIAGSLLGYSGSFDEEVIEQVRKHPDVDFIEKDSEVHTMEKHKIEHNAPWGLARISHRDSLSFGTFNKYLYAAAGGEGVDVYVIDTGTNVDHVDFEGRAFWGKTIPIGDEDVDGNGHGTHCSGTVAGKKFGVAKKAHVYAVKVLKSNGSGTMSDVVKGVEWTTTRHLRQVEAAKSGKGRKGFKGSAANMSLGGGKSMTLDLAVNAAVDAGIHFAVAAGNDNADSCSYSPAAAEKAVTVGASTLADERAYFSNYGPCNDIFAPGLNILSTWIGSKYAVNTISGTSMASPHVAGLLAYFLSLQPAEESAFAVAPLTPEKLKATLIDIGTWGVLTQVPADTENVLAWNGGGSSNYSAIVAKGGYKVASFQEQAQDTLDRLETAANHELHAIYSELKNAFVL*

>gi|170674491|gb|ACB30128.1|_vacuolar_subtilisinlike_protease_Epichloe_festucae

MKGLLALSIVPVLAAASPVVIDSIHKDAAPILSSVDAKVIEDSYIVVFKKHVTSASAVAHQNWVQNLHTTAIAKRSKLDKRNQFPFKNDVFSGLKHTFDIAGSLLGYSGSFDEEVIDQVRMHPDVDFIEKDSEVHTTALETQNNSPWGLARICHRDTLDFSTFNTYLYSDMAGEGVDIYVIDTGVNTEHVDFQGRAFWGATIPVGEENVDGNGHGTHCAGTSAAATFGVAKKASIYAVKVLRSNGSGTMSDVVKGVEYAAQSHQKQMKAAKEGARRKGFKGSVANMSLGGGPSRVLDLAVNAAVGTGMHFAVAAGNDNADACNYSPAAAESAITVGASTLSDERAFFSNYGPCVDIFAPGLNILSTWTGSKYAVNTISGTSMASPHIAGLAAYFVSLYPSTQSAFAVAPLTPKDLKTALLRISSKDMLTMMPEDTNNLLAWNGGGSSNYSEIVGKGGYNAGSLKDQTKDALDRLEKLATDELHAIYSEIKNAFIL*

>gi|170674497|gb|ACB30132.1|_subtilisinlike_protease_Epichloe_festucae

MKGFLGLTLLPLLTAANPIGVGSIHNEAAPILSAANAKEVPDSYIVVFKKHVKDDAATAHHMWVQDIHDSQFARTELKKRSLLGLGDEMYLGLKNTFNIAGSLMGYSGHFHEDVIEQVRRHPDVEYIEKDSEVHTMEEVTEKNAPWGLARISHRDSLSFGTFNKYLYASEGGEGVDAYTIDTGINIDHVDFEGRATWGKTIPSNDEDADGNGHGTHCSGTIAGKKYGVAKKANLYAVKVLRSSGSGTMSDVVQGVEWAVQSHLKKAKDAKDGKVKGFKGSVANMSLGGGKSKTLEDAVNAGVEAGLHFAVAAGNDNADACDYSPAAAENAVTVGASTLADERAYFSNYGKCTDIFAPGLNIQSTWIGSKHAVNTISGTSMASPHIAGLLAYFVSLQPSQDSAFAVSELTPAKLKKNIISIATQGALSDIPADTPNLLAWNGGGSGNYSEIIANGGYKAGSDSIKNRFDGLVDKAEKLLSEELGAIYSEIQDAVVA*

>gi|170674498|gb|ACB30133.1|_subtilisinlike_protease_Epichloe_festucae

MRGILGLSLLPLLAAASPVAVDSIHNGAAPILSASNAKEVPDSYIVVFKKHVSAETAAAHHTWVQDIHDSMTGRIDLKKRSLFGFSDDLYLGLKNTFDIAGSLAGYSGHFHEDVIEQVRRHPDVEYIEKDTEVHTMEETTEKNAPWGLARISHRDSLSFGTFNKYLYASEGGEGVDAYTIDTGINIEHVDFEDRAHWGKTIPSNDEDADGNGHGTHCSGTIAGKKYGVAKKANIYAVKVLRSSGSGTMSDVVLGVEWAVQSHLKKAKDAKDGKVKGFKGSVANMSLGGGKSRTLEAAVNAGVEAGLHFAVAAGNDNADACNYSPAAAENAITVGASTLQDERAYFSNYGKCTDIFAPGLNILSTWTGSKHAVNTISGTSMASPHIAGLLAYFVSLQPAQDSAFAVDELTPAKLKKDIISIATQGALTDIPSDTPNLLAWNGGGADNYTQIVAKGGYKAGSDNLKDRFDGLVNKAEKLLAEELGAIYSEIQGAVVA*

>gi|223641935|emb|CAX43899.1|_cerevisin_precursor_putative_vacuolar_serine_protease_yscb/subtilisin_family_putative_Candida_dubliniensis_CD36

MSIMKIATLFFAALSAVEAAKLLTPSDKRDIVPDSYIVVMKDNVSPLKFDSHMSWATNVHHANLARQGSTATGGLKHVYRIDGWQGYSGSFARETIDRILENDDVDYVEPDRRVHLTALTTQPNAPSWGLGRISHRNNGNSNFVYDDRAGEGITFYGVDTGIDINHPDFGGRAVWGTNTAGGSNSDGHGHGTHTAGTVAGASYGIAKKAKLVAVKVLSDGGTGQWSGIIEGINWSVNHARANNALGKAVMNMSLGGRLSTSVNQATTRAQRAGIFIAVAAGNEDSDAANTSPASAEDVCTVAASTEQDGRASFSNWGSMVEIYAPGTNIVSTTPGGNTGKMSGTSMAAPHVAGVGAAIMASEGISPSEVCSRLVEIGLEQISNPGSGTTNKLLYNNSGR*

>gi|223644906|emb|CAX40904.1|_cerevisin_precursor_putative_proteinase_YSCB_putative_vacuolar_serine_protease_putative_Candida_dubliniensis_CD36

MVSMKVLSILFAALSAAEAAQLLSVQSKQDVIDDSYIVVMKDEVPMSEVNSHVSWVRATHNSGNARRNATITGVKATFDIGKFKGYTGAFDHETLDKILADDKVKYVEPNQRMTIQGVVTQRNAPSWGLGRISSQRPGSRDYHYDDSAGQGIVIYGVDTGIDIRHPDFGGRAIWGTNTIDRDNRDGNGHGTHTAGTFAGNTFGVAKKATIVAVKVLDNRGSGSNSAIIEGMNWAVQHARQNNVLGRAVMNLSLGGGYSQATNNAAENCVRAGIFLSVAAGNDNQNAGNYSPASAPNVCTVGSSDIRDNRSSFSNWGRVVDIFAPGSDILSTRTGGGTTTMSGTSMAAPHVAGLGAYIMAIERTHPSRVCDRIKQVSIRAVRNAGPGTTSQLAYNGSGR*

>gi|223645017|emb|CAX39610.1|_cerevisin_precursor_putative_putative_protease_subtilasetype_YCR045C_precursor_putative_Candida_dubliniensis_CD36

MVSMKFLSAVFAAITAANAAEILSVPNTQDVIPDSYIVVMKDEVATSDLEAHVTWVRNHHHSGHVRRNGTLTGLKTTFDISGFRGYLGAFDHDTLDEILADDKVKFVEPNRIMRIQGTQTQRGAPWGLARLSSSRPGGSDYVYDDRAGEGVIIYGVDTGIDVNHPDFEGRATWGINTIDQDNTDGNGHGTHTAGTFAGARFGVAKKATIVGVKVLDAQGSGSNSAIMEGISWSVDHARKNNALGRAVMNLSLGGSFSQAVNDAAERAVRAGVFLAVAAGNDNQDASNYSPASAPNVCTVGATDRMDVRATFSNFGSVLDIFAPGVDVESTMPGGGTQMMSGTSMAAPHIAGLGAYLMSTENLQPSQVCDRIKQLASNSVRNPGNGSTSKLANNGIGQQIIGSITP*

>gi|223645018|emb|CAX39611.1|_cerevisin_precursor_putative_putative_protease_subtilasetype_YCR045C_precursor_putative_Candida_dubliniensis_CD36

MVSMKFLSTVFAAITAANAAEILSVPNTQDVIPDSYIVVMKDEVATSDLEAHVTWVRNHHHSGHVRRNGTLTGLKTTFDISGFRGYLGAFDHDTLDEILADDKVKFVEPNRIMRIQGTQTQRGAPWGLARLSSSRPGGSDYVYDDRAGEGVIIYGVDTGIDVNHPDFEGRATWGINTIDQDNTDGNGHGTHTAGTFAGARFGVAKKATIVGVKVLDAQGSGSNSAIMEGISWSVDHARKNNALGRAVMNLSLGGSFSQAVNDAAERAVRAGVFLAVAAGNDNQDASNYSPASAPNVCTVGATDRMDVRATFSNFGSVLDIFAPGVDVESTMPGGGTQMMSGTSMAAPHIAGLGAYLMSTENLQPSQVCDRIKQLASNSVRNPGNGSTSKLANNGIGQ*

>gi|223954270|gb|ACN30265.1|_putative_subtilisinlike_protease_Epichloe_festucae

MKGVLSLSLLPLLAAPSPILVDTIHRDAAPILSSHNSKEVPDSYIVVFKKNVSPASAAAHQVWVQDLHTTVMAKRSLRKRNQFPFKNDAFDGLKHTYDIAGSIMGYSGHFDEEVIEQVRRHPDVQYIEKDSEVHAWDEPVTENNAPWGLARVSHRDSLTMGTFNKYLYAANGGEGVDVYVIDTGTNIEHVDFEGRAHWGKTIPTGDDDVDGNGHGTHCSGTVAGKKYGVAKKANVYAVKVLRSNGSGTMSDVVKGVEWAAGAHLSKMVEARKKGNKAFKGSAANMSLGGGKSFTLDLAVNAAVDAGIHFAVAAGNDNADACNYSPAAAEKAVTVGASTLADERAYFSNYGKCTDIFAPGLNILSTWIGSKYAVNTISGTSMASPHVAGLLAYFLSLQPEQDSAFAVSPISPAKLKKDMIAIATKNALTDIPADTPNILAWNGGGSSNYTAIIQQGGYEATRPGNKAAQLTEKIEKLGQNTASQLGAIYSEIKDAFTI*

>gi|223954277|gb|ACN30270.1|_putative_subtilisinlike_protease_Epichloe_festucae

MKGVISLSLLPLLTVASPVMPSTIHNDAAPILSSSNAVEVPDSYIIVFKDHVDSASAAAHHNWVQDIHSQHNELRKRSQFPFAYDPFAGLKHTFNIAGSFLGYSGHFEENVIEAIRRHPDVDYIEKDSLVHTMEDPALEKNAPWGLARISHRESLSFGSFNKYLYAADGGEGVDVYVIDTGTNVDHVDFEGRASWGKTIPTGDEDVDGNGHGTHCSGTIAGKKYGVAKKANVYAVKVLKSNGSGTMSDVVQGVEWAATQHIKKVKDAKAGKAKGFKGSAANMSLGGGKSVTLDKAVNAAVDAGIHFAVAAGNDNADSCNYSPAAAEKAVTVGASTLADERAYFSNYGKCNDIFAPGLNILSTWIGSKYAVNTISGTSMASPHVAGLLAYFLSLQPASDSAFAVAEITPKKMKENLIAIGTQGALTDVPSDTTNILAWNGGGSANYTEIVAQGGYKIKTVSDQVDELIIKAQEVVNGELGAIYSHIKDAMVA*

>gi|238029672|emb|CAY67595.1|_Vacuolar_proteinase_B_(yscB)_a_serine_protease_of_the_subtilisin_family_Pichia_pastoris_GS115

MGCIKVISVFLAAVAAVDARAFFHNRGGNDVIPNSYIVVMKDGVTAEDFESHISSVSATHNLNKAKRGSETAGHKDSFDINGWRGYNGHFDEATIESILNDDKVKYVEHDRVVKLAALVTQPNAPTWGLGRVSHKAKGNKDFVYDSSAGQGVTIYGVDTGIDINHPEFRGRIRWGTNTVDNDNTDGNGHGTHTAGTFAGTTYGVAKKANIVAVKVLSAGGSGSTAGVIKGIDWCVTDARSKGALGKAALNLSLGGSFSQANNDAVTRAQEAGIFVAVAAGNDNRDAKNSSPASAPAVCTAASSTIDDVKSSFSNWGTIIDIYAPGSSILSAAPGGGTRTLSGTSMASPHVCGVGAAMIAQGVSVAQVCNRMKQIANAVIRNPGTGTTNRLLYNGSGQ*

>gi|238030162|emb|CAY68085.1|_Putative_protein_of_unknown_function_Pichia_pastoris_GS115

MGCIKVISVFLAAIAAVDARAFFHNRGGSDVIPNSYIVVMKDGVTTEDFDSHISTVAATHNLNKAKRGSETVGHKDSFNINGWRAYNGHFDEATIESILNDDKVNYVEHDRVVKLAALVTQPNAPTWGLGRVSHRAPGNRDFVYDSSAGQGITIYGVDTGIDIRHPEFAGRIRWGTNTVDNDNTDGNGHGTHTAGTFAGTTYGVAKKANIVAVKVLSAGGSGSTAGVIKGIDWCVTDARSRNALGKAALNLSLGGSFSQANNDAVTRAQEAGIFVAVAAGNDNRDARNYSPASAPAVCTAASSTIDDQKSSFSNWGSIVDIYAPGSSILSAAPGGGTRTLSGTSMASPHVCGVGAAMLAQGVSVAQVCNRLKQIGNAVIRNPGTSTTNRLLYNGSGQ*

>gi|238030480|emb|CAY68404.1|_Vacuolar_proteinase_B_(yscB)_a_serine_protease_of_the_subtilisin_family_Pichia_pastoris_GS115

MGCIKVISVFLAAIAAVDARAFFHNRGGSDVIPNSYIVVMKDGVTTEDFDSHISTVAATHNLNKAKRGSEAVGHKDSFNINGWRAYNGHFDEATIESILNDDKVNYVEHDRVVKLAALVTQPNAPTWGLGRVSHRAPGNRDFVYDSSAGQGITIYGVDTGIDIRHPEFAGRIRWGTNTVDNDNTDGNGHGTHTAGTFAGTTYGVAKKANIVAVKVLSAGGSGSTAGVIKGIDWCVTDARSRNALGKAALNLSLGGSFSQANNDAVTRAQEAGIFVAVAAGNDNRDARNYSPASAPAVCTAASSTIDDQKSSFSNWGSIVDIYAPGSSILSAAPGGGTRTLSGTSMASPHVCGVGAAMLAQGVSVAQVCNRLKQIGNAVIRNPGTSTTNRLLYNGSGQ*

>gi|238933101|emb|CAR21282.1|_KLTH0A06094p_Lachancea_thermotolerans

MGCIKVISVFLAAVAAVDARAFFHNRGGNDVIPNSYIVVMKDGVTAEDFDSHISSVATTHSINKAKRGSETVGHKDSFNINGWRAYNGHFDEATIESILNDDKVDYVEHDRVVKLAALTTQPNAPTWGLGRVSHKAPGNKDFVYDSSAGQGVTIYGVDTGIDINHPEFRGRIRWGTNTVDNDNTDGNGHGTHTAGTFAGTTYGVAKKANIVAVKVLSAGGSGSTAGVIKGIDWCVTDAKAKGALGKAALNLSLGGAFSQANNDAVTRAQNAGIFVAVAAGNDNKDAKNSSPASAPAVCTAASSTIDDQKSSFSNWGTIVDIYAPGSNILSAAPGGGTRTLSGTSMASPHVCGVGAAMLAQGVSVAQACDRIKQIANAVIKNPGTGTTNKLLYNGSGR*

>gi|238934090|emb|CAR22273.1|_KLTH0C11396p_Lachancea_thermotolerans

MGCIKVISVFLAAVAAVDARAFFHNRGGNDVIPNSYIVVMKDGVTAEDFDSHISSVAATHSLNKAKRGSETVGHKDSFNINGWRAYNGHFDEATIESILKDDKVNYVEHDRVVKLAALTTQPNAPTWGLGRVSHKAPGNKDFVYDSSAGQGITIYGVDTGIDIHHSEFAGRIRWGTNTVDNDNTDGNGHGTHTAGTFAGTTYGVAKKANIVAVKVLSAGGSGSTSGVIKGIDWCVTDARSKNTLGKAALNLSLGGSFSQASNDAVTRAQEAGIFVAVAAGNDNRDAKNSSPASAPAVCTAASSTIDDQKSSFSNWGTIVDIYAPGSNILSAAPGGGTRTLSGTSMASPHVCGVGAAMLAQGVSVAQACNRLKQIGNAVIRNPGTGTTNRLLYNGSGR*

>gi|238935693|emb|CAR23873.1|_KLTH0F02288p_Lachancea_thermotolerans

MVCLKTLSVFLAAFAAADARAVFKTQSSNKADIIPDSYIVVMKNGVSHDDFKAHVSSVSQIHSNNKAKRGTNTNGMQREFDIMNWRGYHGHFDPDTLDEILNDSKVDYVEHDQIVRISGLVTQRSAPSWGLGRVSHRQAGIRDYVFDDSAGRGVTIYGVDTGIDINHQDFRGRARWGTNTADRDNADRHGHGTHTASTFAGTTYGIAKNANIVAVKVLGSDGSGSTSGIIAGINFCVQDAQSRGILGKAAMNLSLGGGFSQANNDAVTRAQNAGIFVAVAAGNDNKDARNYSPASAPAVCTVASSTVNDSKSSFSNYGSIVDIYAPGSDIIAARPGGGSTTMSGTSMASPHVAGMGAYMIGMGIDPRQVCDRLKQISLPAIRSPGPSTTNRLLYNGSGQ*

>gi|238938882|emb|CAR27058.1|_ZYRO0C07942p_Zygosaccharomyces_rouxii

MVCLKTLSVFLAAFAVADARAVFKTQSNKNGEMIADNYIVVMKDGVSHDDFKAHVSSVASIHTTNKAKRGTNTAGMKREFDIMNWRGYHGHFDRDTLEEILNDSKVSYVEQDQVVRISGLTTQRSAPSWGLGRVSHRRAGSRDYVFDDSAGRGVTIYGVDTGIDIRHQDFGGRARWGTNTADRDNADRHGHGTHTASTFAGTAFGIAKNANIVAVKVLGSDGSGSTSGIIAGINYCVQDAQQRGILGKAAMNLSLGGGFSQANNDAVTRAQNAGIFVAVAAGNDNRDARAYSPASAPAVCTVASSTIQDSKSSFSNWGSIVDIYAPGSDIIAARPGGGSQSMSGTSMASPHVAGMGAYLIGMGADPRRVCDQLKQLSTPAISNPGSGTTNRLLYNGSGQ*

>gi|238939709|emb|CAR27884.1|_ZYRO0D08822p_Zygosaccharomyces_rouxii

MVCLKTLSVFLAAFAAADARAVFKTQGHKNSEMIPDNYIVVMKDGVSQDDFKAHISSVSSIHSTNKAKRGTNTEGMKREFDIMNWRGYHGHFDRDTLEEILNDSKVDYVEQDQVVRISGLVTQRSAPSWGLGRVSHRQAGSRDYVFDDSAGRGVTIYGVDTGIDINHQDFRGRARWGTNTADRDNADRHGHGTHTASTFAGTAYGIAKNANIVAVKVLGSDGSGSTSGIIAGINYCVQDAQQRGILGKAAMNLSLGGGFSQANNDAVTRAQNAGIFVAVAAGNDNKDARNYSPASAPAVCTVASSTINDSKSSFSNWGPVVDIYAPGSDIIAARPGGGSTTMSGTSMASPHVAGMGAYMIGMGANPRQVCDRLKQLATAAIRNPGSSTTNRLLYNGSGQ*

>gi|238940823|emb|CAR28997.1|_ZYRO0F16764p_Zygosaccharomyces_rouxii

MVCLKTLSVFLAAFAAADARAVFKTQGHKNSEMIPDNYIVVMKDGVSQDDFKAHVSSVASIHSTNKAKRGTNTEGMKREFDIMNWRGYHGHFDRDTLEEILNDSKVDYVEQDQVVRISGLVTQRGAPSWGLGRVSHRQAGSRDYVFDDSAGRGVTIYGVDTGIDINHQDFRGRARWGTNTADRDNADRHGHGTHTASTFAGTAYGIAKNANIVAVKVLSSDGSGSTSGIIAGINYCVQDAQQRGILGKAAMNLSLGGGFSQANNDAVTRAQNAGIFVAVAAGNDNRDARNYSPASAPAVCTVASSTINDSKSSFSNWGPVVDIYAPGSDIIAARPGGGSTTMSGTSMASPHVAGMGAYMIGLGADPRSLCDRLKQLATPAIRNPGSSTTNRLLYNGSGQ*

>gi|594711826|gb|EXU94848.1|_ankyrin_repeat_protein_Metarhizium_robertsii

MSTGKKLLLAAKLGRFAEVQSLLDEGVDPNAKDSSGSTPLHIAAKGESPEVVELLLEHGANSNTKEESGRTPLHYAAQNTRDEIAQILLDYWADPKITDKVGSTPLHYAATHGNPEIIRLLLESGANPNAQDESGLTPIHYAAKHGEPDSVGLLLKKGADPKVKDRSGSTPLFYAAAKNVLELLLGRRNISGMETDAKGKQMSLTPMYHISINGNHLDESIKPATDASETNYILVQTRMQLNEPERQYLVNAGLIFHDYVSKNTYLCGYRDEDLDKIRQLDRVVFVDAYRKDFKIASGLKSSKANIDRDVKVNVIFHDGINSSRARSLPSEILELEDVKLDYTNIPYKAQITIPREKLEPLAGIDEVRLIEEVGEIKPRNNQARLIVGLDVEKESDVMTDQKRYEGEGQVIAISDTGLGTGHRESNHYAFEDRILALYAMNGTTLDPIGHGTHVCGSAVGNAAMNNGVHIRGTAPKSQLVMQSLWHSGSERLNAPRDLYQLFNMPYTHPDQKVRVHSNSWNQVMVDGQLPYTSRAEDIDTFVWKHKDMVICWAAGNDATFHHAMGRANEGQIGAEAAAKNCITIGACENRRPDMQITYDDIDKAQFPDSIKYQRVARDPSLVAAFSSRGPTSQDGLYKTRIKPDIVAPGTLIISAHACIRSTTGSGLEKCHLHDEDNRWCYDSGTSMSTPLVAGCAAVLRQVLIPKPEFNQASGCEYPSAALIKAILINGADIVSDPNIPPVTPNVHSGFGRVNMAKSMTIACGKDGTGFKEEELSNDTEKIKQLINLRANHACNTIKATLTWSDFPGDALKNRLRLELRHSSFLNPKSSDHQYNNVQQVLWKNLPPGNIAITVGIIRKLFRSPQPFAVVWHLYSEKGKDSAKTCSWL

>gi|594711868|gb|EXU94884.1|_peptidase_S8_family_protein_Metarhizium_robertsii

MHHIQINGNSRPDEGAASTASLSTFAVPPPSVAISNYILIQTKGPLSAQQKQELRDKDVEILEYKGEDVYLCGYKPTTLEPLISNLRDFVTDAEVYHPDYVVEPDLKTGNDDDEAEVEVALHDNIGDNDMSRVVNDIAAQADIPVANVELHDRKACVKVPKSKLAQLARIDEVKAINEVHTRRIFNNVACGILEAHTPVGTANTTYKGKGQVVCVADTGFDTGSLAGYHEAFGDRVISLHARGRPAARGEPGNSDDPDGHGTHVCGSVLGKGDHAEEGEIEAPASEAKLVMQSLFDKFDKSRGPDPKSWNAGLGGIGNSYPDLFGGVFREGATIHTNSWGGPPQPYVERESARIDSYLWGNKDMTVLFAAGNSGVDIDRHPGHVDPHSLSPQAVSKNVITVGASENCRPNVKSPVAGNKPLVYGAWRSKFPFGPISVDNVADNPEGMAAFSSRGPTRPDGRIKPDVVAPGTTILSARSSQIEPGHHGESWGHSNDARWMYLGGTSMATPLVAGCCAVIRGALVDNHVPRPSAALIKALLINGAVPMKGQYKQALPNGEFGPSVDAPNPNSGFGRVNLANSLRNIVANQESTVYGYQDVTGDQSLGMDDQHRGEHAVTVDIPEDSPAALTLKVTLVWTDFPGERLQNDLDLIVAGAGVEKHGNQGDGSGFDRVNNVEQVVWKNVKPGSYTVTVRACRTTKDPQPFALAWRAFA

>gi|594711882|gb|EXU94896.1|_peptidase_S8_family_protein_Metarhizium_robertsii

MVCLKTLSVFLAAFAAADARAVFKTQGHKNSEMIPDNYIVVMKDGVSQDDFKAHVSSVASIHSTNKAKRGTNTEGMKREFDIMNWRGYHGHFDRDTLEEILNDSKVDYVEQDQVVRISGLVTQRGAPSWGLGRVSHRQAGSRDYVFDDSAGRGVTIYGVDTGIDINHQDFRGRARWGTNTADRDNADRHGHGTHTASTFAGTAYGIAKNANIVAVKVLGSDGSGSTSGIIAGINYCVQDAQQRGILGKAAMNLSLGGGFSQANNDAVTRAQNAGIFVAVAAGNDNRDARNYSPASAPAVCTVASSTINDSKSSFSNWGPVVDIYAPGSDIIAARPGGGSTTMSGTSMASPHVAGMGAYMIGLGADPRSLCDRLKQLATPAIRNPGSSTTNRLLYNGSGQ*

>gi|594712446|gb|EXU95412.1|_peptidase_S8_family_protein_Metarhizium_robertsii

MAFVKILSVSLAATAVANAATILSPRYPNDVIPNEYIVVMKDGVSSASFASHSAWVADMHYYNHTKRALPGHGIQEVYDIYEMKAYSGKFDEDTIQRIAKEPDVAFVEPNQIVTISEISVQKAAPSWGLSRISVKENHVSSNTDYFYDSSGAAGIWVYVVDTGVDIKHPDFEGRAVWGTSTVDRSKTDRLGHGTHVAGTIASKTYGVAKAVKIIAVKVFKERTTSYKDIIGGIDWAVKHAKKNNMLSKSVVNMSLGGGRSSAMNMAAANAHKAGMFVAVSAGNTPIDAVNFSPASEPLACTVAASDKDDMQARFSAFGPSVDIFAPGTDIVSLVPRKKFGTKSGTSMAAAHVSGAGAYIMAIEKIPGNEVCNRLKELAQSSIVRASDKTTTKLLYNNSGK*

>gi|594712727|gb|EXU95680.1|_peptidase_S8_family_protein_Metarhizium_robertsii

MGFVKILSLSLAATAVADAATILSPRYPNDVIPNEYIVVMKDGVSSASFASHSAWVADMHYYNHTKRALPGHGIQEVYDIYEMKAYSGKFDEDTIQRIAKEPDVAFIEPNQIVTISEISVQKAAPSWGLPRISVKENQLSSNTDYFYDSSAGAGIWVYVVDTGVDIKHPDFEGRAVWGTSTVDRSKTDRLGHGTHVAGTIASKTYGVAKAVKIIAVKVFKDRTTSYKNIIGGIDWAVKHAKKNNMLSKSVVNMSLGGGRSSALNMAAANAHKAGMFVAVSAGNTPVDAMNFSPASEPLACTVAASDKDDMQAQFSAFGPAVDIFAPGTDIVSLVPRKKFGTKSGTSMAAAHVSGAGAYIMALEKIPGNEVCNRLKELAQSSIVRSSDKTTRKLLYNNSGK*

>gi|594712891|gb|EXU95838.1|_peptidase_S8_family_protein_Metarhizium_robertsii

MLEARLEQRLEQLSTTLEQAEEGHLTGARPGASSDEGIRESVQHRVIQPVKENEYTAFLATHVQTREPNSNWTAVTAKSFFRSISSFNRDFILEDASPRLEPWARRRTRVAIIDSGIISCENKQHLRKLDPMICAAGERIINRRSWVGVESDCNDECGHGTHVVRQLLEVAPRAELVIAKVASNTENIPLSYVAKAIEWAVDDNDVDIVALSLGVSQDDKHINTVLDRILNLDGKQTGQVSNGVKGHRRRIVFAAAGNHGGNRMRASPARRRGVICVHAADGEGDTKSFNPDPDHEWEDNFSTLGVDIVSLRQGESVYISGTSYATPIAAGIAANMLHFARNQPGMREEDKNLLHSYFGMRQIFKRISFPRGNYDYICPWRRKDRMANQPDEATDMSLSELLWSSIGVAREEARKAEAL

>gi|594713583|gb|EXU96516.1|_peptidase_S8_family_protein_Metarhizium_robertsii

MYDPTTNEVVDITNPYIQFSFDSMSSGDFPANGQHSYIHVFSFGLMLLELELGHQIAITAEDENDADEEYPPVYMALLRIFHSRKDDLDDPYIREVINSCLDFENRVEGIKHRAFSDHLRPRAALLRYIVQPLVNRLRAAHPDVSFDLINGPQQSIRAGFPHPASNHTGLSRAKVQHRTSTLQQFPGNSASLRMSSAQPHFIRSQSESGRQNALNKHATLVSNTVSVAGHALSASKKPKNDHPRSTEMAISLFDERTELISETDKSVRLASTFFDLFEKFRRHMQPAHGRCRVKIAVLDTGIDTAQCGLQLRREAIITDRETAEPSVNGDPVKATRSFIGPPDDTNDVCSHGTHIAEILLRLAPEADLYIAKISNYLHVDKVDQIAEAVNWALEHGCDIISMSFGMNPSLVPEASLFNVQNAIHRAATARKIMFAAASNCGGNGPRAYPASDPSVICTHAADGNGYDGGINPPKGDHADYFSTLGMAIGCIWDERCVYKSGTSFATPVAAAIAANVLDYAAYSVAAGKLTDQRYRELRQSQGMKKAFTKFLSVQMQHYRYVAPWHFWKTGVTDDYIWQRLKVDLTP

>gi|594713705|gb|EXU96636.1|_peptidase_S8_family_protein_Metarhizium_robertsii

MGFFKTFAVLAAASLANAAEIFSAERDAIPNQYIVVMKDEVSSQSFGSHRAWVADMHHSNLEKRALVGHGIKKTFEFENMKGYSGVFDEETIKEISENPDVAFIEEDQVVKIADIIEQPNAPTWGLGRVSNRQVGINDYFYDRSAGAGIWAYDVDTGVDIRHPDFEGRAVWGSNHVDRSNTDGHGHGTHVGGTIGSRTYGVAKRARIIAVKVLDSRGSGSNSGVIAGIDWSVNHARQNNMQTRSVMNLSLGGGRSAASNMAVANAQRAGLHVAVAAGNDNRDASNSSPASEPTVCTVASSDIRDNKSSFSNWGSLIDIYAPGSSITSLAPGGRTRVLSGTSMAAPHVAGVGAYIMALENIPGNRVCDRLKQLSHAAIRNPGSRTTNRLLYNGSGH*

>gi|594713944|gb|EXU96871.1|_peptidase_S8_family_protein_Pr1D_Metarhizium_robertsii

MGFIKTLSISLAAASAANAAKILSPSRPDDVIPNQYIVVMKDGVSGEAFGSHRAWVSDMHHTNLTRRALLNHGIKKTYDFMRMKGYSGVFDRDTIKDISQSPDVAFIEHDHVVRLTELVEQPDAPTWGLGRVSHQEPGNMDYVYDDTAGDGVWAYDIDTGVDIEHPDFEGRAVWGSNHVDDDDTDGNGHGTHVGGTIGSLTYGVAKKVRIIAVKVLDARGSGSNSGVIAGIDWSVNHAMENNVAERAVINLSLGGARSDTTNMAVANAVQAGLHVAVAAGNDNQDAENSSPASEPTVCTVAASNINDQKASFSNFGAVVDIYAPGEEILSLAPGGGTQTLSGTSMAAPHIAGMGAYLIALENITASAACDRIKELGLEVINNPGAGTTNKLTYNGNGQ*

>gi|594714038|gb|EXU96964.1|_peptidase_S8_family_protein_Pr1B_Metarhizium_robertsii

MGFIKTLSLSLAAASAANAAKILSPSRPDDVIPNQYIVVMKDGVSGEAFGSHRAWVSDMHHTNLTRRALLNHGIKKTYDFMRMKGYSGVFDRDTIKDISQSPDVAFIEHDHVVRLTELVEQPDAPTWGLGRVSHQEPGNMDYVYDDTAGDGVWAYDIDTGVDIEHPDFEGRAVWGSNHVDDDDTDGNGHGTHVGGTIGSLTYGVAKKVRIIAVKVLDARGSGSNSGVIAGIDWSVNHAMENNVAERAVINLSLGGARSDTTNMAVANAVQAGLHVAVAAGNDNEDAENSSPASEPTVCTVAASNINDQKASFSNFGSVALIRNAVDIYAPGEEILSLAPGGGTQTLSGTSMAAPHIAGMGAYLIALENITASAACDRIKELGLEVINNPGAGTTNKLTYNGNGQ*

>gi|594714121|gb|EXU97046.1|_peptidase_S8_family_protein_Pr1H_Metarhizium_robertsii

MGFLTVLYLSLAALSVTNAAQIMSAPNGAEVVPNGYIVVMKDDTSKQDFASHRVWVSGIHHNITRRGLDGEGVKQTYDFDNLRGYSGIFDKDTIKDISNDPKVAFVEPDAIISQHVVVQQRKAPWGLSRLSNRKGGRNYVFDSTAGAGVWAYVVDSGVDIRHAEFRGRAVWGSNQVDNQNSDGTGHGTHVAGTIAGKTYGIAKKAKVIAVKVLNSEGKGPTSGIIAGINWSINNARQNGMLHKSVINMSLGGSYSAGLNHATAQAIKAGIFVSVSAGNDNINSNNNSPASEKSVCTIAASTEDDGKASFSNWGPAVDLYAPGHNILSARPGGGSQVMSGTSMAAPHACGVAAYLIAKEGIPGSRACLRLKQLSQPTIHNPGPDTTRRLLYNGSGR*

>gi|594714236|gb|EXU97160.1|_peptidase_S8_family_protein_Metarhizium_robertsii

MTGFLTILSLSLAALSVTNAAQILSVPQGAEVVPNGYIVVMKDDTSEQDFSSHRAWVSNIHHNVTRRGLNGEGVKETYDFDNVRGYSGIFDKDTIKDISNDPKVAFVEPDAIIKQHVFVQQRKAPWGLSRLSNRRGGRNYVFDSTAGNGVWAYVVDSGVDIHHSEFQGRAIWGSNLVDNKNSDGTGHGTHVAGTIAGKTYGIAKKAKIIAVKVLDSEGKGPTSGIIAGINWSIKHARQHGKLQKSVLNMSLGGSYSAGLNHVTARAIKAGMFVSVSAGNDNINSNNNSPASERSVCTIAASTENDGKASFSNWGPAVDLYAPGHNILSARPGGGSQTMSGTSMAAPHAAGVAAYLIAKEGIPGDRVCLRLKQLSRPTIRNPGPDTTSRLLYNGSGR*

>gi|594714262|gb|EXU97185.1|_peptidase_S8_family_protein_Pr1I_Metarhizium_robertsii

MTGFFTILSFSLAALSVTNAAQILSVPKGAEVVPNGYIVVMKDDTSQQDFSSHRVWISSIHHNMTRRGLDGAGVKQTYDFDHLRGYSGIFDEDTIKDISNDPKVAFVEPDAIISQHVVVQQRKAPWGLSRLSNRRGGRNYVFDSSAGSGVWAYVVDSGVDIRHSEFQGRAVWGSNLVDNKNSDGTGHGTHVAGTIAGKTYGIAKKAKVVAVKVLNSEGKGPTSGIIAGINWSIRHARKHGMLQKSVLNMSLGGTYSAGLNHATAQAIKAGMFVSVSAGNDNINSNGNSPASERSVCTIAASTENDGKASFSNWGPAVDLYAPGHNILSARPGGGSQTMSGTSMAAPHAAGVAAYLIAKEGIPGNRACLRLKQLSQPTIRNPGPDTTSRLLYNGSGR*

>gi|594714576|gb|EXU97496.1|_peptidase_S8_family_protein_Metarhizium_robertsii

MLVNIKLYQSINDLTYPTLRRSENNPFESSTGDDQEARKSKAAAKFQVFEAGAPLVSDSRGNLEAIFRVDELCKQGWNDSALNQIETLLGDEVRDFGSKIQVNPEAGMTAHENVDEPEAKAKEYIDALSQTGLPDPRFHLTVFPASLDEGLAGLKQKTELNARIEKRTQAVCSPLLLKPQPDYSAAVEFADPTKLYELLATRKHCSEWRHSAFLLLSDPDDSDILFSMYLSSYMRAWNLRELQIPKLISGLHHRSIPLDEKNLTPVSLGALMTGEELKTVTLDDEVRKAWLAHRLSLSLRLLYMGPWIQQDWDFDTLHIMPKNNSELLPPIDELYIGCTLAPGYEMANSTPTARCSPYSSQMCPKFFLSFAQLLVDILKGERGYRSYSESQLNDWFYMLQYEVGHNLKEMSNEYYWRAIEGCILFTMHDDSHECADALVRAKDVISKHIITYLRMHVESCQAKHSRCLQPDGTSEETGIRLWSEIESKYQQIDSPKARGEDSFVTHMNRFTATFIKNLPALEKKVRVAIIDSGFYVHECDPWLNNPAVEARIGEKRNFFSPDDNDPDKSKWQDMTGHGTQVARLVLQFAPLAEVVIAKITNSPTLKYTKTERLVEALKWAGERADIINLSFSLGTLPIIDVQKEINNLVDNKKLVFAAASTNAAKWASRAWPARERGVFAIHASDETGETCAGINPDPLSEDDNFLAFGLEVDSFWDGRYQRISGTSFATPVAAAIAADMIELARRIPESNFGEDITRYRVMRNLFLKYMSENKVPGNFHRLVPWTEKLWDWNGPVETQEKFKYALRDVITFC

>gi|594714858|gb|EXU97775.1|_peptidase_S8_family_protein_Metarhizium_robertsii

MTGFFTFLSFSLAALSVTNAAHILSVPKGAEVVPNGYIVVMKDDTSQQDFSSHRVWISSIHHNKTRRGLDGAGVKQTYDFDHLRGYSGIFDEDTIKDISNDPKVAFVEPDAIISQHVVVQQRKAPWGLSRLSNRRGGRNYVFDSSAGSGVWAYVVDSGVDIRHSEFQGRAVWGSNLVDNKNSDGTGHGTHVAGTIAGKTYGIAKKAKVVAVKVLNSEGKGPTSGIIAGINWSIRHARKHGMLHKSVLNMSLGGTYSAGLNHATAQAIKAGMFVSVSAGNDNINSNGNSPASERSVCTIAASTENDGKASFSNWGPAVDLYAPGHNILSARPGGGSQTMSGTSMAAPHAAGVAAYLIAKEGIPGNRACLRLKQLSQPTIRNPGPDTTSRLLYNGSGR*

>gi|594715051|gb|EXU97966.1|_peptidase_S8_family_protein_Metarhizium_robertsii

MLGNIDVFMSGSMEAQITKTESIDGPGHVISPQTQLVVQAFKDLEQKAEAAHGLEMLTTSKRLVTIFLLKLRGHNLEFLQSLADLTDNNCRYVETVGEALLESVVKRRCGSEYGRYCDLLCRTLKELKTMEKFLGFTRYGPEDISLIFSRHKQLKKLLQSLCTLGGVAGAALQISFARIQENLDELVEIRRLSQKDPSAQFFTALGTLRKCAASAADILGKQNGITCRCSRSHQVYFRIEDCPSLNQSEDDSNPWCLACSLLVHTPSSLIKQKERVGMTFTNIAVSLVTDPHALKQGKTENSILPTVMEKVEEGGRSSCINSQEDESNNSSADMPATASDSLCATLNEALRPQEQHAADIVIYRQDLGLHLLGQPEIGWANPLPIRRWAENFPRRELLTEQKLHIAYKVAFSLLYLSSTPWIRESWTWQDIYLTWHENFDDVIYPVFSGDMSTSKPLAADNPQTHGPATHRIPSVTILGRFLVELWCGTSWKQLEKAFLAGVESSDTADPDTFIFSQLINWVRNSRIADRDKPFYQEGTSYFMAVENCFQCDFNPGPLNGLPLLESNGFACWIYKHVLRPLEYALEDFRRRQDRLLGARLNLNPEINSPDDGEPDRRLRLFVNERIPADRSMKEDLADDWICRYSKVKELARKLGRNRPDDPSDRVRVAILDTGVDVTHDNLYGPWTKGQIFYQNFVGMPSNIPEDSDGHGTHITSILLQMAENVDIYVARVSPDGQHWKSREVEDAIRWAADEKQVHIISLSFGFPNVDQSLEGIRKAILDAHAADVLVFAATGNKSHSDYIAFPACLDEVISVASTDGDGALSNFVPELRVGKRLCAIGEAIEAAWINKDNPTLHVHCKTERQYGTSYATPVVAGVAAMVMDLLWSIKDKRKYHINYLKPWKFFDCTSKSLESESRWCNVNTNGAGFLLLETLSHVYGKGFSRRPSI

>gi|594715103|gb|EXU98017.1|_peptidase_S8_family_protein_Metarhizium_robertsii

MLPFRPHQLPRTTNPSQSRPVEEWINDIYLGSYEQGNDPAVDCMCLPIRLAPESRIRRFRKDESGTAFLMRRNVDGFVLTSVMIVYAFPSISHKDMGREHEYINALADHHGQATMYEIGFLWVAPGMAVLLAQDYDMQNIVAAIISPLICWRIMPHQTFSVIIFWDEEENLRAYIPDPQSMNGDDPGYIEATNLSEVFPTCKQTLSSASQQERNLGTTLFGYQNASAGADDRANIGADDFFTDDHHMKRVQSFFSTIRRPHQRRVKIAVLDTGEELGRDFTMHPLGEPDLKDNTGHGTACTHLLLKNCPTAVVYTAKISNQSTFDEKTAERISEAIQTAITEWEVDIISMSLSYEFEVEIIDEALKNRQGDKKRPVLFFAASGSFGKDKGEHSAGFPARHENVICASSSTYEGNKSDFNQGPDDMNRWKNFSTIGENLCVAFPAELNRGNYEKRVSGTSMATSIMAGIAALVLEFCNTWKERGGRPTLEKAAKMQGMLQIFRECMLSNSNSSSQQGHLNLVPWYLFDGSSYTRDYASVGNHIAEAPRRL

>gi|594715573|gb|EXU98483.1|_peptidase_S8_family_protein_Metarhizium_robertsii

MTGFFTFLSFSLAALSVTNAAHILSVPKGAEVVPNGYIVVMKDDTSQQDFSSHRVWISSIHHNKTRRGLDGACVKQTYDFDHLRGYSGIFDEDTIKDISNDPKVAFVEPDAIISQHVVVQQRKAPWGLSRLSNRRGGRNYVFDSSAGSGVWAYVVDSGVDIRHSEFQGRAVWGSNLVDNKNSDGTGHGTHVAGTIAGKTYGIAKKAKVVAVKVLNSEGKGPTSGIIAGINWSIRHARKHGMLHKSVLNMSLGGTYSAGLNHATAQAIKAGMFVSVSAGNDNINSNGNSPASERSVCTIAASTENDGKASFSNWGPAVDLYAPGHNILSARPGGGSQTMSGTSMAAPHAAGVAAYLIAKEGIPGNRACLRLKQLSQPTIRNPGPDTTSRLLYNGSGR*

>gi|594715661|gb|EXU98570.1|_peptidase_S8_and_C14_domain_protein_Metarhizium_robertsii

MRRYEDVQALLICWKDAIKTFQDQRTELQRVLRSPYNFGAKAIDIPSTDPEKYLEDKIRNFREAHDKEQNLLLVYYGGHGDVLRLDGQLIIKCFDGIQHPYVEWNTRQKSFLKGSKADTLIILDCCHSASAIESIYCGQDNVVELLTACSIEGKAPLRGNHSLTSKLTDLLQSKELFTSGFDTSYLYNRLVHYQKLKGQVYLGDDEDEKGVTPLRIVLLSRREQFRNLHICRRITPDSEDEENDAEINGATGRESEPGKQDGQESGEAAHLSATHRSKSYSDVATNTEWDLVIRRESAVSIQNQSVRSVYVDAWTNTTTDTAPLTKRQPAIQALGPVQIEPLTFYREPATRRPWRRKMAIEQDEQERDHRAWFHRYAIFLGQWGMAREEENRTSPYKIALLGTGVDCALILSHDCHQVIRGRNFTSTGENGSWDQEHAGHGASCLTMLLETAPFAEFFIAKITDRQRISNADTIAQSIEYSVKEWKVDMIVLPLGLHIHHDGVANAISQAIRQNIICLAATGNDGANTRAAFPARMHGVIPIFSTDSYGNPSPYNASPMRGRKNFSTFGENVMVWRDDRTDGPSYKSGTSFAVCIAAGMLAAMLVFARDYLQLDERDWNTLHTPAGAEKYLELMSSSRGGYEYVAPWLLISNEILMENSSGSEVDFKNAIKSQIVTALRHLR

>gi|594715718|gb|EXU98627.1|_peptidase_S8_family_protein_Pr1G_Metarhizium_robertsii

MGIFRFISISLAAVSAANAGHILSMGHAKTIPNSYIVVMKDGTTEEDFTHHQSWVQSIHTHNVTRRGLLDNAGVRHKYGFGSMMGYAGLFDEDTIKDISDDPKVMFVEPDTTITIHGELTQNDVPSWGLARISSQRPGTEDYTYDSSAGEGITVYSVDTGVDIHHEDFEGRASWGTNMIEDGYDKDGNGHGTHTAGTMVGKTFGIAKKAKVVAVKVLDNNGSGPTSGIIAGINWCAQHASQNGGTDKAVINMSLGGGSSSALNRAAAQAVQKGMFLAVAAGNDNQDARTSSPASEDTVCTVGASAENDERSSFSNWGPAVDLFAPGSNIVSTRPGGGSQSMSGTSMASPHVAGLGAYIMALEGISGSAVCDRLKQLGTSSVTNPGPGTRTNILINNGDAKNGGKKPSQPSQPPKPSQPSKPQQPSEPQEPSEPQEPAPGQPAPAPAPVPQHPHTPFPNDDFNFDDFWKKYFGTDHWRKTFGRFWN*

>gi|594715872|gb|EXU98779.1|_peptidase_S8_family_protein_Metarhizium_robertsii

MDIQRSAAGRFAAPQTTRYSPNIPEYRFGHVSNAIFENDIEELHRVLSSIRIMPPASVYHEIVPIWETKRNFVHAVLEWGDRAHKFQVLQAAASHIYRHDENENLVHNSVAEEISICILTQSPDVIYHRHHDGQPTVLHIAAKSRSEALSQTIFSSIKNNKTFDQLLHVKNKGSKTPLRIAVENDSLFFVRQILRYNTRPINLDDSKLLTWVLNEGTSEALQTLIELRPEEMDENVLQHALEMKSERLINALKNARECRDLFFGKRAFLHQLVKDGETLIVNTLLDKFPELALELDEEEKPVLSYNSDESIRDRVAGIILRTLPSMCPEKRQRYYRLCADNSSIPSTSEIVRALIDDVPDKRKEISLSLGGFGSWVSHAESFLGLIQKSNSYTSPDDKPSNALLQLMFEKSLIFVDIPITDLPAPRTDGKPTLIRSEVWSILNWLQKTKHVEGIYELSIRDSCYLPHAENVIRACLKDFNISVLDWRCPDLSLEVLHDGDESKTPICPDLETLRLYARGWPALAYWTSEESLYFLGRFTRLKQVEVFVLKEFVGMTLCETYATEATERFDKFKTAHSNAIPFQLEITPKRWSDLSPTDQRPVLRRETTAVEVTKLGDFLLAYESIHRDFANQAWRSDRLAKGILCNELDESRKHTPYIRVAIIDNGVDPESIHCHKITGASFVPSHTGESNWWYIRHPHGTKMARIVTDLNPHCHLLVAKVGDSRSDFTAARIIKALGWAVAAGADIISLSLTLDKEDVNLELAVNKAAASGAVILASVRGEGVNTEIKPIPAAYGNVLAIGSADGTGAASSGTLEGQARHLFPGERIVAHTEYLGGLDDAPNVSGPSVATAVAAGVASLVLSCNRFALFKKRDFKRPDYHHTLQVNVVKQIFEQMSDGKYVRPWVFFKDEKAKPSWGEGDSVLDWIQGKYRGIKDDGRA

>gi|594716119|gb|EXU99024.1|_peptidase_S8_family_protein_Metarhizium_robertsii

MGHPKSRVFASEMLASVASEASDILFPIAIANMSASAGEIATRLRYIGGDLLEQQLYQLPPDIQSLCLQALQDLLKICKWPVLSTAASHFHSSESPQELRDVLIQLCEKEQFNNGVQRSITKFCKERAETIKPYQLRFVQLSDKLLKNAQEIGCSVNDSGPYLHLRPLGHDVYPDDVYKLLLQGTKSLATCAHQYHNAPALSGFTGDDWHLTMLCLNSGIRSENKRAIFNIITATPEMVYWQEMGVTVPIKDIEPNDVQRHQQAKPFDPSKPSLMEYGDICKRLENPSYAKLYLDLDQDCQLHERPDPVGLQHIITGCGINLSELLSLNELTVEHKIKLSYTVARAFWQFYNSELMNARWTSEDIVFIPLNKEFSPTEGIPLRAFVPFPFGPRYKKSPQEFCQEDQFTHRYPRILYLGIILLEIGLGQALRLEHNPKLSLLAHINTAHAKAKMKLKELDDAEWDGFRWKEYFVEAVRNCFDSTNFKESPRLRNPRQRGSSECADNDAKDSAFLERRDALYQKVVAPLLWLATTGFEDSEEVPLVPIRKKIRRQPTLAKNGDEELQTFWNEIHARPSLMSGSSISTEGFLEDLQIIAGHIARCRRLAKATKPIRVAILDTGCHRGLRFFQNPQRFNRLKGWKDFTSAGSESEIDTFGHGTFMARLLMHVAPIIDVYVVRVAENTEDLETQENSIAKAIEFAGLDPDWNVDIISMSFGFPNKPGVKHAVISDAIDKIKKDRNDSVLFLASAGNSWERRVDFPASHQEVIPIYAADATGAFLRSNPARAGKGSEKLGTYGTDIPTSILEEIQEAFPKAALSAGTSLATAIAAGIVAMMLSYIEAVPALLQLRGFQEVCAKLYTKRGMENMLHVMSLTTGYRQQFINPVHFWGEKKTDVDVFISICSAVEKMNNET

>gi|594717055|gb|EXU99955.1|_peptidase_S8_family_protein_Metarhizium_robertsii

MGVFRFISISLAAVSAANAAQILSMPHAQTVPNSYIVMMKDDTSDDDFNHHQSWLQSTHTHNITRRATVQNAGMRHKYNFHKMKGYSGVFDDETIKDIAKDPKVMFVEPDTIISVHGKVDQNNVPSWGLARISSSKPGTQDYTYDSSAGEGITVYSVDTGVDINHEDFEGRAIWGSNQVNDGDDNDRSGHGTHTSGTMVGKEFGIAKKAKLVAVKVLGNDGSGPTSGIVAGINWCVEHARQNGGTNKAVMNMSLGGGSSSALNRAAAQAVEQGMFLSVAAGNDNTDARSSSPASEPSVCTVGASAEDDSRSSFSNWGPSLDLFAPGSNIISARPGGGSQSMSGTSMAAPHVAGLAAYLMALEGISGGAVCDRLKQLGSASISDVGPGTPTNVLINNGGAKGDGKSPKPSPKPSHPSEPQQPTEPQQPAPGEPSTPAPAPMPPTPQHPHTPFPGGDDFDFDGFWKKYFGGEHWRKMFSSFFN*

>gi|594718993|gb|EXV01884.1|_peptidase_S8_family_protein_Metarhizium_robertsii

MSQSSSDKIKMEKHERGRTHDKSKKASSTKVAKGKKPGNTQRPQHTANEHDNQKEETLSEILSDALQKFPDTCTEACNPVKCERMSCKQRLHDFEVLFKTKLKDMTQRAQEKQDNTLHLLVKDKGWKSEHTTPPKRLFDWMLQQREHDVLLQQRDPRGFTPVHNALYDHVHDFIDVLLKADRLSTISILRQDFAGSNCIHLATQHASPHLSTMIHKCKGDNEIFMDGDKTEKGTPLHIAVQDVFIPVVDEVEEDDSDPEDQGLEDDDQDGLLDCHDNADHDSHHLDEDVDEFALDSDYESSSLSEYETDLELEEVKYRFENKEEREKRLVKILDDLDKEENTRPRPPPRTHDANPDNPQRMGAEDAVDDLAKENNSPSIPKVVMSQGLIDLPPDHSVRLLVEAYPKALIAKNSCKRTPYQEREHVLLSDDVVKRLVQQYAHKKGPRGDTMEIREARAKRTIIVQDPVARYIMSYCIRESKSRELTMQQLYKPGQERHIEFDLEGFPSPSVTHNYFDNLGKHLQFESILRYVALPVLSVEPPPSRNPRIKQVSPTETQYELHRNGRSDLVTVFDWLWSKGVREIVKIRVVDGDLPHADASIVEALYGFNVKMWDWKRVDLCSDVIYESSPVIKEVSVYSSGNNAVLMGWASEDGLGNREKFPCLEKINLFVQDGLEDDKRWQSNNRLCQKKITEHGLKGRDGHGGKEIEFKIIIDNDQGRSTTENIEPAPAWIKSTRQFATFLMNASREQGKDKQVAPVKIAIIDDGIDATLHDLQSKIAGGATFCPYPHSSELVNSYFVPRGKHGTLMAQLICDLCPGTELYIARLEELPLLSGSGRRVTARSAAKAVEWAVNCGVDIISMSWTIQTAAHDSEDMSSLETALNNAKAAKIHMFCSASDQGANTKEEYFPGDWKQCIRIGGATFTGEKLTWVDDKVDFWFPGRNVPFLSRDGKSVVYDSGSSIATAAASGLAGLLIYSARLIYSGTNEARNYPFHTQTAMAAAFRIMAKGMDGKFPRTDEVLNKLFKNKIQQATQKPPKSLDMETLEWSDSSKKALTDLLLHIQVGSPIEEVVLW

>gi|594719019|gb|EXV01910.1|_peptidase_S8_family_protein_Pr1K_Metarhizium_robertsii

MGVFRFISISLAAVSAANAAQILSMPHAQTVPNSYIVMMKDDTSDDDFKHHQSWLQSTHTHNITRRATIQNAGMRHKYNFNKMKGYSGIFDDETIKDIAKDPKVMFVEPDTIVSVHGKVEQSNVPSWGLARISNPQPGADSYTYDSSAGEGITVYSVDTGVDVNHEDFEGRAIWGSNQVNDGDDRDGSGHGTHTSGTMVGKMYGIAKKAKLVAVKVLGNDGSGPTSGIVAGINWSVEHARQNGGTKKAVMNMSLGGSSSSALNRAAAQAVEQGMFLSVAAGNDNQDAQSSSPASEPSVCTVGSSAEDDSRSSFSNWGPAIDIFAPGSNIVSARPGGGSQSMSGTSMAAPHVAGLAAYLMALEGISGGAVCDRLKELGTSSITDAGPGTPTNVLINNGGAKGGQPNPNPAPAPSPSQPSEPQQPTPSQPGQPGEPFPGEPFPGEPFPGQPFPGESAPAPAPAPMPPTPQHPHTPYPGGDNFDFDSFWKKYFGGEHWRKMFSSFWN*

>gi|594719580|gb|EXV02470.1|_peptidase_S8_family_protein_Metarhizium_robertsii

MGVFRFISISLAAVSAANAAQILSMPHAQTVPHSYIVMMKDDTSDDDFNHHQSWLQSTHTHNITRRATIQNAGMRHKYNFRKMKGYSGIFDEETIKDIAKDPKVMFVEPDTIISVNGKVEQSNVPSWGLARISNSQPGANSYVYDSSAGEGITVYSVDTGVDINHEDFEGRAIWGSNQVNDGDDRDGSGHGTHTSGTMVGKEFGIAKKAKLVAVKVLGNDGSGPTSGIVAGINWCVEHARQNGGNDKAVMNMSLGGSSSSALNRAAAQAVEQGMFLSVAAGNENQDARSSSPASEPSVCTVGSSAEDDSRSSFSNWGPALDLFAPGSNIISARPGGGSQSMSGTSMAAPHVAGLAAYLMALEGISGGAVCDRLKELGSSSITDVGPGTPTNVLISNGGAKGGNPKPAPGPSPNPSQPSEPQQPAPSQPGEPGESFPGEPFPGEPFPGEPFPGESSPGESAPAPAPMPPSPQHPHTPYPGGDNFDFDGYWKKYFGGEHWRKMFGSFWN*

>gi|594719582|gb|EXV02472.1|_peptidase_S8_family_protein_Metarhizium_robertsii

MSTQVIVNGNELSTAVPSTSDTSSTNFIVVRGTRRIIADDKRELAKKGAAVTEYLGNDIYLCHYEPSNLQPIRDLEFVAQANIFPHDVKKSRSIVDAIKSSESEGHPKKTFDVEIFLHKGEKVSDSFINDLREKTGIVPKDIVAEENVIKLTADASTIDKIVEVDAIKAIEQSIKPKLFNDISREDIGIVYPRVSPLGVNYEGQGQVIAIADTGFDTGSKDSPHPAFRGSIVALLSNKQRAGRTDDPVGHGTHVAGSALGDGWSDTMGGSIQGAAPKASLVLQSILDSDDVNIRIPRDLTGLFDEAYSTDVIVFAAGNDGEEMSYYSKMSQIGSTSAAKNSICVGATESRRPSIGQKYDPGGFIGHPDKIAYTSSRGPTFHKIIKPDVVAPGVAILSTCSRHEAMADRRQYFGQTDDNNWMFYSGTSMAAPLVSGCCAVLREALQSNNKTASPSAALVKALLINGADDLKLPKPDQGFGRVNLKESLRCVVESGPGEQVDWEQFGFEDVGGENALKEGKTWPREPKKIDFTGDHTGTLKVTLAYSDPPGENLQNNLILKVVIEQQSGVSEEQRGDEGFVAENNVEQVKWDITSKDKAAWITVIADRIAQLDGLQPFAVVWGIY

>gi|594720243|gb|EXV03132.1|_peptidase_S8_family_protein_Metarhizium_robertsii

MTLDDEDLTLDNEDEFHLLSEGATDEDQVRIQFENDVEDARRLSQTKFQDEGEEREQRTRFVLDRTPQWSRTTHDGRNFLHHLASYDYNRKPFVSLQWLMSRAMNKLPHLMGAMDRSRRTPLTTALAAGNVWFSYAACKNQKDETRQQFGAALASECEDLDNDRGSTCLHTALVCPMSGEALRGEIVKIMCGFVPRSMFSAVDVKGRTPLHVAVEYERCCRVQVGIVDELLRRGPGALDVEVAAYSGRAMSVYQYHEYTRRRAESRKNPARGRKEVREGGRSAAANPRPDLRAAAAPERPDKMVMGPPLPRDRAEPLPGLRRRVSMPVAAAPPDGKPPGLSLNTQHPCPPAAGVDESPLDAPLNGIDAALQRDEERDQAAGQIAQLLKLFYLRTQMPERASRCLHVQDEQDKELWFDFGPPKKLTKTDFRKHFGHLQFDSALQYVAFPQSTLDKGEDSRHVLRQGRTDMVFFFEWLAQKGVERIIKVIVEDLKAPSHSDQAIEKSLKPFNVEMLDWRRADLDPVSLARIGQCLREVHLYWSGRNTVLRAWSEKEGLALIPTLETIYIVQVEGLEPETRVRENLDAFERRLGESWPTEAKPKVYIQLLGAGGPLPSLTQPSDLQPRRQRPVDPHKWMQCMEEFASHFRQIRALNDKSADPALAPVKVALIDDGADITHPDLKGMKFPGKSFHHYREGSSWRVSPFWDSSSGHGTLMARLIHRICPSAVIHVIKLSTFAGEASAKLQINTDSAVQAIEYAVEQGAQIISASWTVKPPTEAGRKKAFDDAVHNALNTKGALMFCAASDQGKSADLTYPHGSNPNSFRIGAARATGSALDNVGDGHELSFLFPGHEVVVDSAYEDVPDKQFGRFAPHSGSSVATALAAGLAALIVECVRLGVLYTGETGPLDETVTIGRDDLVRICERRQMEYALASIGTSRNTDNKYIEVWNMFGAAAEKLKHSEGDRMSQLEIIAGLARLFLRKGA

>gi|594720304|gb|EXV03192.1|_peptidase_S8_family_protein_Pr1F_Metarhizium_robertsii

MGVFRFISISLAAVSAANAAQILSMPHAQTVPHSYIVMMKDDTSDDDFNHHQSWLQSTHTHNITRRATIQNAGMRHKYNFRKMKGYSGIFDEETIKDIAKDPKVMFVEPDTIISVNGKVEQSNVPSWGLARISNSQPGANSYVYDSSAGEGITVYSVDTGVDINHEDFEGRAIWGSNQVNDGDDRDGSGHGTHTSGTMVGKEFGIAKKAKLVAVKVLGNDGSGPTSGIVAGINWCVEHARQNGGTDKAVMNMSLGGSSSSALNRAAAQAVEQGMFLSVAAGNENQDARSSSPASEPSVCTVGSSAEDDSRSSFSNWGPALDLFAPGSNIISARPGGGSQSMSGTSMAAPHVAGLAAYLMALEGISGGAVCDRLKELGSSSITDVGPGTPTNVLISNGGAKGGNPKPAPGPSPNPSQPSEPQQPAPSQPGEPGESFPGEPFPGEPFPGEPFPGESSPGESAPAPAPMPPSPQHPHTPYPGGDNFDFDGYWKKYFGGEHWRKMFGSFWN*

>gi|594720305|gb|EXV03193.1|_peptidase_S8_family_protein_Pr1E_Metarhizium_robertsii

MRLFMFLSGLAVSIAAPGLVPRAVHAPLIIPRGDGASLVADQYVVYLRSDASEADHKDALKSLNIKPRHEYRSLVRGFSANLDGNTLAGLRRHPAVDFIEQVTMSSISNLKPVNRAPRNSTGAIIEQKGVTWNLDRISHQKSSSESETKYVYDSRAGEGTCAYVIDTGVDDTRPEFKGRALQIKSFVANETGDNSRDGHGTHVAGIIGSGVAKKTRIFGIKVLNSEGSRETDALIAGYASIGLEYVPIDAANRTCPNGVVVSFSINSGLYEKAISVAAGALVKKGYFMAASAGNIVAHSSPASEATVCTVGSVDINNKPASDYGYGPGLDLLAPGVDILSLQPDNRTALLSGTSQATPHVTGLAAYFASIFGKSAVPKLCQYMKDVAVEGAVKEQNLYTANLLVTNSVVGA

>gi|594721437|gb|EXV04324.1|_peptidase_S8_family_protein_Pr1A_Metarhizium_robertsii

MRLLIFLSGLAVSIAAPGSAPRAVHAPLLIPRGDDASLVADRYVVYLRSGASEADHKDAIESFNIKPRHEYKHLRRGFSANLDGNTLAGLRRHPAVDFIEQVTTSSISSSEPVNRALQNSTGAPIEQKSATWNLGRISNQYSTSEGESKYVYDSRAGEGTCTYVIDTGVDDTHPEFEGRALQIKSFVANSTVDDSTDGHGTHVAGIIGSASYGVAKKTKIFGIKVLDSNGNAEGDRLIAGYAPTGLEYVPVDAANRTCPNGVVVNYSINSNGYAKSINVAAAELAKKGYFVAVAAGNKPRDVAQSSPASEATVCTVGSVDINNKPAVDTGYGPGVDLMAPGVDIMSLQPDNRTSLLSGTSMATPHVTGLAAYFASIYGKSAIPNMCQYLKDVAVKGAVKEQKLYTANLVATNAVVDV

>gi|594723183|gb|EXV06069.1|_peptidase_S8_family_protein_Pr1J_Metarhizium_robertsii

MRLLIFLSGLAVSIAAPGSAPRAVHAPLLIPRGGDASLVADRYVIYLRSGASEADHKDAIKSFNIKPRHEYKHLRRGFSANLDGNTLAGLRRHPAVDFIEQVTTSSISRSEPVKRALQNSTGTPIEQKSATWNLGRISNQNSTPEGESKYVYDSRAGEGTCTYVIDTGVDDTHPEFEGRALQIKSFVANSTVDDSADGHGTHVAGIIGSASYGVAKKTKIFGIKVLDSNGDAEGDRLIAGNTSTGLEYVPVDAANRTCPNGVVVNYSINSKGYAKSINVAAAELAKKGYFVAVAAGNKPRDVAQSSPASEATVCTVGSVDINNKPAVDTGYGPGVDLMAPGVDIMSLQPDNRTSLLSGTSMATPHVTGLAAYFASIHGKSAIANMCQYLKDVAVKGAVKEQRLYTANLVATNAVVDA

>gi|629683643|ref|XP_007807117.1|_subtilisinlike_protease_PR1G_Metarhizium_acridum_CQMa_102

MHALAYFAILPLALGAVLPADSYTTTPRPGKLVPGRYTVKFKDGTPVSVRDNILSQLDNANEHVDYNDIFVGFTKSMSETEVDLVRNDPNVEYVEQDRDVYGFGIIEQPKASWNLGRIANRKRGIDKYVYDETAGEGTCAYVIDTGVDDTHPDFGGRAKQIKSFVPGETTDGHGHGTHVAGILGSTTYGVAKQTRIFGVKVLDNNNQGYESRIIQGIDFVVNDQKKRRCPKGIVVNLSTGAAKSKIFNAAAAALVKTGVFFGAAAGNFNDDASNYSPGSDPSVCVVGGTDKDDKPFFIKGRGGQPDFATNFGARVDIFAPGQDIVSTRTGGGELTMSGTSQACPHVVGIAAYLASLEGITGTKPLCDRIRKLSTKNAIINQHPNTPNRLAFNGATLSSNRKPLCTEIKQLEFGIALSDDMFAGTNDEIGAILEGPAGKAEFSIVTDASRGFNTRVPVDMKASFGSDTIKIDGINSISLTAKGPWISLLTNDKWKVKDVTLHAKCAEPGLEVADEKYISLNAWYQHPDASWLPFTGHSKQIVAKLGVSRADWTMKPPCVEVKDITYWFQLGDKWLGGADGILSFKLGDGKRITIGENLDAGFFKSGTMDLKDIYGRDTMDLRDIKKLQIFDNVGYKGKTDEWFLQGIGFGATCVDGGQKVKLSKFGNEDEWLGDDHHYEDLVYARDIIPSDWVKAV

>gi|629684207|ref|XP_007807399.1|_Subtilisinlike_serine_protease_PR1A_Metarhizium_acridum_CQMa_102

MHALAYFAILPLALGAVLPADSYTTTPRPGKLVPGRYTVKFKDGTPVSVRDNILSQLDNANEHVDYNDIFVGFTKTMSETEVDLVRNDPNVEYVEQDRDVYGFGIVEQPKASWNLGRIAHRKRGIDKYVYDETAGEGTCAYVIDTGVDDTHPDFGGRAKQIKSFVPGETTDGHGHGTHVAGILGSTTYGVAKQTRIFGVKVLDNNNQGYESRIIQGIDFVVNDQKKRRCPKGIVVNLSTGAAKSKIFNAAAAALVKTGVFFGAAAGNFNDDASNYSPGSDPSVCVVGGTDKDDKPFFIKGRGGQPDFATNFGARVDIFAPGQDIVSTRTGGGELTMSGTSQACPHVVGIAAYLASLEGITGTKPLCDRIRKLSTKNAIINQHPNTPNRLAFNGATLSSNRKPLCTEIKQLEFGIALSDDMFAGTNDEIGAILEGPAGKAEFSIVTDASRGFNTRVPVDMKASFGSDTIKIDGINSISLTAKGPWISLLTNDKWKVKDVTLHAKCAEPGLEVADEKYISLNAWYQHPDASWLPFTGHSKQIVAKLGVSRADWTMKPPCVEVKDITYWFQLGDKWLGGADGILSFKLGDGKRITIGENLDAGFFKSGTMDIKDIYGRDTMDLRDIKKLQLFDNVGYKGKTDEWFLQGIGFGATCVDGGLKVKLSKFGNEDEWLGDDHHYDDLVYARDIIPSDWVKAV

>gi|629685231|ref|XP_007807911.1|_Subtilisinlike_serine_protease_PR1J_Metarhizium_acridum_CQMa_102

MLSLTNLLLVVPAALAAPVLLNTRQTDSISGSWIVRVNQQSVLSDVISQVTAAAGAGATKKHTYDFGGFKGFSIDGVSDLTSIVANIAAIQSIERNTVVRTSALVTQDNVPSYGLARISSRKNGATSYIYDSSAGAGTYAYIIDTGIRDTHEDFGGRATFGASFVDGEQGGDGNGHGTHVAGTTGGTSYGVAKKTNLIAVKVLGAEGSGSNAGVLAGINYAVKDAQSKGRIGKAVANLSLGGLFSPMTNAAVTEAVKQGLFLAVAAGNSGLPTITSSPASAPDVCTVGASDKNDAKASYSNFGLLVDIFAPGTNITSAWKNSDTDTNTISGTSMATPHITGLGAYLLGLEGSRNPKALCERIQELSTKNTLSSALLSKNYLAYNGNGR*

>gi|629690867|ref|XP_007810725.1|_subtilisinlike_serine_protease_PR1H_Metarhizium_acridum_CQMa_102

MIPSALLLLLPMTAAAPASKRAEPAPLIVPRDDSAVFHDQYTVILKDDSDSQALTNVMELIPGNATQVYGNLFKGFTAELDEASLGALRDHPAVDFVEMDQKVSIPDEPQGKVDAVEANPNATAPVVGPQGPVPSHPDQVFRRYNGLTQYLNNPTGGEGVCAYVVDSGVDVTHPEFGGRAHMVESTVDPHGLDLVGHGTHVAGILGSNSYGVAKRVTIYGIKALSERPDASGISNMIAGLDYVARDAPHRHCPNGIVVNLSAGIAERNDALNMAARGLVERGYFVAVAAGNERHDARLNSPASEPSICTVGDYRYRDSNFGPAVDIQAPAVNVLSTVPGGRIYRLTGTSMASPYIAGLAASIASAHHQRAGPDLCAWMVQRATPQW

>gi|629691111|ref|XP_007810847.1|_subtilisinlike_protease_PR1E_Metarhizium_acridum_CQMa_102

MELIPGNATQVYGNLFKGFTAELDEASLGALRDHLAVDFVEMDQKVSLPDEPQGNVDAVEANPNATAPVVGLQGPVPSHPDQGFRRYNGLKPYLNNPTGGEGVCAYVVDSGVDVTHPEFGGRAHMVKSTVDWHGLDLVGHGTHVAGILGSNSYGVAKRATIYGIKALSEQPDASGIWTLIAGLDYVARDAPHRHCPNGIVVNLSAGIAERNSALNRAARGLVERGYFVAVAAGNEGHDARFNSPASEPSICTVGDYSYRDSTFGPAVDIQAPAVNVLSTVPGGRIYRLTGTSMASPYIAGLAASIASAHHQRAGPDLCAWMVQRATPQW

>gi|629692381|ref|XP_007811482.1|_subtilisinlike_protease_PR1K_Metarhizium_acridum_CQMa_102

MHPTLSLLLSILPLTLASPTRKRSEPAPLIIPRGEAFTLVPDEYIVKLKQDSAKAALDDAIKIIPGDADQVFDSIFKGFTGKLDSPTLDAMRAHPDNAYFEAYGGATQQQAPWNPARLSHRRPVASDYIFDESAGEGTCAYVVDSGLYAAHPFEGRAQFLGTFIGDHNDNCLHGTHVAGTIGGRQVGVAKKTAIYGIKVLDLNREEKCGADTSVIVAGMEHVARDAAQRHCPNASSSTSAWGAAGRSPSTRPPRRWATRTTTPWTRPPSPRPARDSNYGGVVDIQAPGVDVVSARAGGGYISMSGTSMATPHVAGLAAYLLGLRKTSASNLCSYLQENA

>gi|629692863|ref|XP_007811723.1|_putative_serine_endopeptidase_Metarhizium_acridum_CQMa_102

MHPTLSLLFSILPLTLASPTRKRSEPAPLIIPRGEAFTLVPDEYIVKLKKGCAKAALDDAMKIMPGDADQVFDSIFKGFTGRLDSSSLDALRDNPDVDYVEQNAYYEAYGVTTQQQAPWGLARLSHRRPGASDYIYDESAGEGTCAYVVDSGNPSNSAGWQEFEGRAHFLGTFVGDQNDNCLHGTHVAGTIGGRQVGVAKKTTIYGIKVLDMNREQKCGADTSVIIAGIEHVARDAAERHCPNGVVVNLSLGGGWSQAMNEAAAALVRRGFFVAVAAGNGDQNHNPMDAASVSPASEPSVCTVGSVDSRDRPARDSNYGDVVDVQAPGVEVVSARAGGGYITMSGTSMAAPHVAGLGAYLLGLRKASASNLCSYLQESALQNSISGLHWGTRNLLVQNGVGA

>gi|629693569|ref|XP_007812076.1|_subtilisinlike_protease_PR1I_Metarhizium_acridum_CQMa_102

MHPTLSLLFSILPLTLASPTRKRSEPAPLIIPRGEAFTLVPDEYIVKLKKGCAKAALDDAMKIMPGDADQVFDSIFKGFTGRLDSSSLDALRDNPDVDYVEQNAYYEAYGVTTQQQAPWGLARLSHRRPGASDYIYDESAGEGTCAYVVDSGLYAAHPEFEGRAHFLGTFVGDQNDNCLHGTHVAGTIGGRQVGVAKKTTIYGIKVLDMNREQKCGADTSVIIAGIEHVARDAAERHCPNGVVVNLSLGGGWSQAMNEAAAALVRRGFFVAVAAGNGDQNHNPMDAASVSPASEPSVCTVGSVDSRDRPARDSNYGDVVDVQAPGVEVVSARAGGGYITMSGTSMAAPHVAGLGAYLLGLRKASASNLCSYLQESALQNSISGLHWGTRNLLVQNGVGA

>gi|629695793|ref|XP_007813188.1|_subtilisinlike_protease_PR1F_Metarhizium_acridum_CQMa_102

MMHLARLLPLLALAAAAPALRDAPAELLTPSDNSTVIPGKYIVKMKDSVGASGFSGVVKSLAAEPHLTYDSIFRGFATELDEAGLKALREHPDVDYIEPDQEAAASGLVVQQTAPWGLTRISHRRRGSTQYVYDNSGGKGVCAYVIDTGVDARHPEFEGRAHQLKSYIPGSNIDDNGHGTHVAGTIGSRTYGVAKRVTIFGVKVLAANNKGSNSVIIKGMDFVHSDARRRRCPNGVVVNMSIGGGYSKAENQAAARLVRDGFFVAVAAGNDNRDARYFSPASEPSVCTVGGTDKFDNRYTMSNWGPALDINGPGVDVLSTLPNGRAGRKTGTSMATPHIAGLGAYLAALGRKRAGPWLCKKIQNLATKNAINNQVAGTVNLLAFNGAT

>gi|629698163|ref|XP_007814373.1|_subtilisinlike_protease_PR1I_Metarhizium_acridum_CQMa_102

MQLLRATTCLLLPTLALAAPPVREAPLLTPRGAAASQLIDGSYIVKLRDGSSSRASPPSCPLRSWLPSAASRGVSILPYPADRLIHDAKQKSQVEFVEQDAVVQAYDFLTQEDVPWGLARISHRSPGQTSYVYDESAGEGTCSYIIDTGIYVNHTQFTNRAHWLANFIDTDNTDGNGHGTHVAGTIGGITYGVSKKTSLYAVKVLRASGSGTLAAVIAGIDFVAADFPTRGCPNGATANLSLGASRSTAVNAAAAAAVRAGIFLSVAAGNSADDAFFYSPASEETVCTVGATDEGDVRAWFSNYGEGVDVFAPGVGVESAWIGGPSATNTISGTSMAAPHVAGLASYLLALLGPKTPAELCEYIRETSTNGTITDLPTGTFNGIAFNGNPGAL*

>gi|629699035|ref|XP_007814809.1|_subtilisin_putative_Metarhizium_acridum_CQMa_102

MAANQPSYLLAPNWDFPPDRDVRLGNIIKDPKLPGCSLNSESRIDPNQDSESPHAVTTTKKQSWSLTRHNLLSLNAGLQTSFLAPLIGFGGDVAGRAFRDDGARFKCQTLETQYFNPSDRYIAESLSAKTVKAYTKKYWWKSIYMITGVKIAHGASIETFRNTGSGADVAVTTDLTPSTAVSVEEKPHFSSETMHEHHIVFDVCEEPFIIGYQLLKIKPKSDGKYADKPFNKFALLSDEQIEDATSKEAVELLHQVYDIGNVEGQDDIDGETTEIWSQMRVGVLNCFWLLSPRKIRHPSFVLTGTCSLQALGIRGAETNPPVRFLCSQLFMDDKSCYFCYYKVRIYFEVVILSRLLTISTSSSAYVEKPSLPPGANMKRFRSIFSKATKLNFLRCESDLSKYEKLMERSASGQPERVDWEITEKRRNFSNINWEETEALQDGPEVSPTDDEYCLTMKDVHPACDTSDAQEAIRRFYDTLCDHWPFHCGDTHRSMLRLVGHRQYISEDGRAAHTFEFDTLSSSHNRHWGRIKFLAEVPQRRRKVAFEGIADQENPNRPTPVDLCQTIERSAREKMCYFVNFGCGQSSKLPGHLPIILREPLIGLGNLLQAGDADQVTCGYQLSLREKALVAVIFASTLLQLSKGAWGKGALDEGPWLQRNWTENGIQFLLNIDNKPILDEAYLPIIFNGPPDGSTFNCSTSSFLALAITLLQLSNSEVVQKFNAIRKEILQLEEIKETENDQIDRWALHHLMTVDTFTALVDDQYQKAIKACISGKLATDIGQVEEGIVQECFEQEVILPLKNYLEKMMGPEQAMASMRRNLVSQTKHSRQFQEGNWCCSSDKSDDEGRHSIDEENAKYAKQWFHMFEAKVYELVKLQPEDDVRGTSSPARIKIAILDTGIQFPREAFDLFGDQIIEYKSWIQCNEDRSELDGGHGDLDGHGTHCASVLLNVAKNAHIYVARVFRARTERNMDDNATNKAIAEALSYAIDKWQVDIITMSFGRPDRVECINSQIQRARDRGILMVSAASNAGGLGTISWPAKAQDVICVHALDGYGNGTNFTPSPRPHNHNFAAPGVSIEGYWPTHLSPSRKPFQYMSGTSCATPIVAGILAVLLEYVRKHETKYGYYADLFKRLREKEGVVTILGKMTSTEKRNKYQFLEPWKLLDAQDEFQNDDIRMKEIIRLLKSVG

>gi|629699297|ref|XP_007814940.1|_subtilisinlike_protease_PR1D_Metarhizium_acridum_CQMa_102

MRISTISFGLLVAGSATATIEKRAPLITRDESGIEGKYIVMMKPASGNEISTARVKSAAKAVDVKPDMVFDNLGGFSASLTARDVEDLRNNPNVAYIEQDSVARISATQKQPPWGLARVSSKTTGATRYTYDDSAGKGTCAYVLDTGIDTTHPDFEGRAEFIATYVDDIWVDDHGHGTHCAGTIGSKTYGVAKKTKLYGIKLFNSTGEGVASSIIAGMDLVLRDAPKRDCSKGVVVSMSFGEIPSKGINDAAKALVNAGFFAAAAAGNGDDNGRPVDASGFSPASEPSICTIGATTKDDTVATFSNYGKVVDLYAPGVAVLSTWPGGITRSISGTSMATPHVAGVAAYFLGLGKSAAGLCEYLQSIALKDVIKGVPSGTKNLLLQNGQAK

>gi|629699449|ref|XP_007815016.1|_subtilisinlike_protease_Metarhizium_acridum_CQMa_102

MKGEVATLAEDDLTASISSQPDYRYTMSGFRGFSGTLTSKELSRLQESEHVDYIEQDAKVHASTMVLQYNSSWGLARISHKKPYETTYLFDESAGEGTCVYVIDTGIETENPEFQGRAFFLADFSGEDSWKDLMGHGTHVAGTIGSATWGVAKKTTLFAVRVLDRYGSGTNAGVLAGMQFVLKDAPQRAAACPKGLVVNMSLGGYKTKAMNAAAAAIASAGLFITVAAGNDGEDAVGYSPASEPSVCTVGATASNDTFASWSNHGPLIDILAPGVDITSTWLGGAIQTFSGTSMATPHITGIGAYLLGLGAKADNLCGIIAGMAIKDAIDPSTMYANTVNLLAWNGAQGSSGYGGS*

>gi|629699955|ref|XP_007815269.1|_subtilisinlike_protease_PR1F_Metarhizium_acridum_CQMa_102

MRVTATLVCILPLALGARLLKARGGTPLNSYIVVLKETYNGGEVTISDVEDGLGDVNKSQTYTSAVGFRGFAAKLNATQLDALKSSPKVDYIEEDAMVHISGSVTSQTGADWGLARLSSRAPNDTTYRYDATAGTGTCAYVIDTGIMIDHSEFEGRATWAGNFVDKNDTDGNGHGTHVAGTIGGVTYGVAKQTRLFAVKVLDSSGSGTNSQIIAGMNFVVQDAPKRNCSKGVVVNMSLGGEQSTAVNSAARAVVQAGYFLAVAAGNESNDTKLYSPSSEESVCAVGATTRNDSMASYSNFGAGVALFAPGSDIKSAWNDGKTKSISGTSMATPHVAGLGAYLMGIRGPMSGSAVCDLVKKSALRGKVSGLPSGTANRLAYNGGA*

>gi|629700033|ref|XP_007815308.1|_intracellular_serine_protease_Metarhizium_acridum_CQMa_102

MDMSFWQELRLMLTSTSSESSVPQREVLDEGDICALLSEPTFARLVYSIDREKACFLRLRQPQLPELLPAPGNGLSLSDILSRYRLTPSHRILLAYVVAHTYWQFYDSEVYCLDSKNFSQYNTVQELGEPEIGKEVKKEGSPQVKGVEARRKSLYYHVVRPLAWLANNGFRPRLGDRGYISKNQSFSSLRSSVRTDGTYPHDSGLFHSSVSLGPLRWLQQLKTISASVDDMRQATSSTRPVRIAILDTGLRLSIPYFQDDFDGPERKKQVISYKDFVGQSDSDMKDLFGHGTFMMRLVAESAPAAEIMVARVAENTEGLSSCQTNIVKAIQWAGSEGADIISMSFGFPRPIKDISDTIESVSRRGEGVVFVASAGNSPYEEEGFPACHPSVISVYAANSHGTFLESNSQTPSKRADVLCVVGEVPGEILEELRDELPGICQAGSSVSTAIAAGISATMLAYGDFLPHILPARQGSSVYKRMHTSRGITHASRDDTVFKSLWEARRRCQAKTKTGFNY

>gi|629700065|ref|XP_007815324.1|_putative_subtilisinlike_protease_Metarhizium_acridum_CQMa_102

MKLQILLALLPLTFAAPAPLAPRDGTPIPGQYIVKFKSNALLEHSILNLINDLFQDATQKAYVKHIYNIGSFAGFAVTTTDSAAQQIRLNPNVELVEQDRVIQIELEHVEEIVQRSPVTQYTTSSTWGLGRLSSKATNSGRYVYDSTAGAGICAYVIDTGIETTHPEFQGRATFLANFAEDGSNTDGHGHGTHVAGIIGSKTYGVSKRVNLFAVKVLNADGTGALSGVLAGVDFATNDAKSRGCKSSVANFSFATGKSDALNSAAANAASSGLFIAVAAGNSAADAIDTSPASEPSVFTVGSTDSNDRLAASSNFGASVDILAPGVAILSTWKSGGTAVLSGTSMATPHVTGLAAYLLAYEGIRTPAALSSRMTSLANINRITGVPAGTVNLLAFNGNSRA*

>gi|629700107|ref|XP_007815345.1|_subtilisinlike_protease_Pr1B_Metarhizium_acridum_CQMa_102

MNDQTSDTISKPRRGRIPDAEGAELVNMPKLEGTIPSITPQKRQGVFAMRGFILFSLLSVVTTGSSVGRDNLAPLFKSKTSVPDSYIIKLKDGNSSTSFESTLAPFTDNSHHVYEAAFKGFSATLDTAAIRHLRRHPDVEFIEQDAIFTINGFVEQKTAPWNLARISRRKRGSTSYVYDDSAGEGTCSYIIDTGIDASHPQFGGRAQNIKTFVNQTTDGNGHGTHLAGIVGSVIYGVAKKTKLYGVKCLDDQGSGTISNVIAAMDFVAKDAKTRKCPKGAMANMSLGGGYSAAVNKAAASLVASGVFVSVAGGGSNTDAKNTSPASEPTVCTVGASTEKDERATYSNYGAVVDIFAPGGSILSTWLNGTTNTISGSSMSAAHITGLGAYIAALEGSPGGEKLCKRLQELATKGVLTNVPLGTPNLLAFNGNPSG

>gi|629701627|ref|XP_007816105.1|_subtilisinlike_protease_Metarhizium_acridum_CQMa_102

MHRIQINGNSRPDAGAASTASHNAFAVPPPDVANSNYILIQTRGPLNAQQKQALRDKEVEILEYKGEDVYLCGYKPTTLEPLTSNLSDFVTDAEVYHPDYVVEPDLKAGNDDDEAEVEVGLHDDIGDEDMSRVANDIAAQADIPVTNVEVHDRKACLRVPKSKLAQLARIDEVKAINEVHTRRIFNNVACGILEAHTPVGTANTTYKGKGQVVCVADTGFDTGSLAGYHEAFGDRVISLHARGRPAARGEPEKSDDPDGHGTHVCGSVLGKGNHAEEGEIEAPASEARLIMQSLFDKFDKSRGPNPQSWNAGLGGIGNNYPDLFDGVFREGATIHTNSWGGPPQPYVERESARIDSYLWGNKDMTVLFAAGNSGVDIDRNRGHVDPHSLSAQAVSKNVITVGASENNRPNVKSPVAGNKPLVYGAWRNSFPFGPISVDNVADNPEGMAAFSSRGPTRPDGRIKPDVVAPGTTILSARSSQIVPGHHGESWGHSNDVRWMYEGGTSMATPLVAGCCAVIRGALVDNHVPRPSAALIKALLINGAVPMKGQYSQALPNGEFGPSVDAPNPSSGFGRVNLANSLRNIVANQESAVYGFQDVTGDQSLGMDGRHRGEHAVTVDIPQGSPAALTLKVTLVWTDFPGERLQNDLDLVVAGSGIEKHGNQGDGSGFDRVNNVEQVVWKNVKPGSYTVTVRAFRTTKDPQPFALAWRAFA

>gi|672375368|gb|KFG77701.1|_putative_serine_endopeptidase_Metarhizium_anisopliae

MRGFILFSLLSMAAVDANIGQDNLAPLFKSKDSVPGSYIVKFKDGISSTSFDSTLASFTDDSHHVYDAVFKGFSATLDSVAIRNLRRHPDVEFIEQDATFTINGFVEQKNAPWNLARISHRQRGSTSYIYDDSAGEGTCSYIIDTGIDATHPQFGGRAQNIKSFVNTATDGNGHGTHLAGVIGSVIYGVAKKTKLYGVKCLDDQGSGTTSNVIAAMDFVAKDAKTRGCPKGAMANMSLGGGYSAAVNKAAASLVASGVFVSVAAGGSGTDAKNTSPASEPTVCTVGASTEKDERASYSNYGPVVDIFAPGVSILSTWLNGASNTLSGSSMSAAHITGLGAYIAALEGFPGGEKLCKRLQELATKGVLTNVPSGTLNLLAFNGNPSG

>gi|672375511|gb|KFG77835.1|_putative_subtilisin_Metarhizium_anisopliae

MRRYEDVQALLICWKDAIKTFQDQRTELERVLRSPYNFGAEAIDIPSTDPEKYLEDKIRNFREAHDKEQNLLLVYYGGHGDVLRLDGQLIIKCFDGIQHPYVEWNTRQKSFLKGSKADTLIILDCCHSASARNPFTVSKELFTSGFDTSYLYNRLVHYQKLKGQVYLGDDEDEKGVTPLRIVLLSRREQFRNLDICRRITPDSEDEEDDAEINGATGRESEPGKQDSQESGEAAHLSATHRSKSYSDVATNTEWDLVIRRESAVSIQNQSVRSVYVDAWTNTTTDTAPLTKRQPVIQALGPVQIETLTFYREPATRRPWRRKMAIEQDEQERDHRAWFHRYAIFLGQWGMAREEENRTSPYKIALLGTGVDCALILSHDCHQVIHGRNFTSTGENGSWDQEHAGHGASCLTMLLETAPFAEFFIAKITDRQRISNADTIAQSIEYSVKEWKVDMIVLPLGLHIHHDGVANAISQAIRQNIICLAATGNDGANTRAAFPARMHGVIPIFSTDSYGNPSPYNASPMRGRKNFSTFGENVMVWRDDRTDGPSYKSGTTFAVCIAAGMLAAMLVFARDYLQLDERDWNTLHTPAGAEKYLELMSSSRGGHEYVAPWLLISNEILMENSSGSEVDFKNAIKSQIVTALRHLR

>gi|672375948|gb|KFG78255.1|_hypothetical_protein_MANI_020298_Metarhizium_anisopliae

MEMTWRHFVDPNGEFDELDVAKDALSRISSVAADVGARQKLPVAGCLAAKLCIAKKTLDSADAGQLLRVQRRLPKLIEDLERWADPPEAKRSVLPSVVRPKSYRSFQDAEDRARIGMAAFLRGPSFIRDQYFKKLDLFSRAVMVVGFEETDETKENVARDAEEYPAHINLSLYSVLKAHSTMKLVLAYIISKSFWQFYDSPWMDAKWTSDCIHFLPESAPNDEAATQDGMLYASKPYLAVDFDAKSREIVEYCDSYAVIYRYPRLLALCIILLEIGRGATLMLQDYGSMEANLNATWTLANRLTDRKQSWGDFDYPDYRRAVANCLDGKLLKQNYRTDAVQPEMDTLTRRTVIYNTIVQPLKKLLERVGFLDTLNTLDPIDSKGPEPAFSIPAAPAFPARSQGDGPSSSRQWLEHFPSINRYIRDLTRSNPAGRPVRITILDTGFDNEAVFFRNPARRQRIKGWKDWVDLSDSPLDDNGHGTHTVALVMKVSPQADIYVARIARDRNSLRDCVESIVEAIHWAATTCQTDIITMSFGFSDEILAITKAICEAELHRDNKLLFFAAASNSGGNGYEMFPASHDSVIAIRETNSKGAFSDTNPPVNPLGPAVLGTLGKDVPSAWLSNLDGEAPKSGSSVATAIAAGIAGMCLTLAGAGLHSPNVNLPRRAGKLWTRRGMKALLMKMSQDMGNRCYFISPMRFFSGLDEASIWNALADACVR

>gi|672376391|gb|KFG78683.1|_subtilisinlike_serine_protease_precursor_Metarhizium_anisopliae

MRGLILFSLLSVAAADANIGQDNLAPLFKSKDSVPDSYIVKFKDGISSTSFDGTLASFTDNSHHVYDAVFKGFSATLNSVAIRNLRRHPDVEFIEQDATFTINGFVEQKNAPWNLARISHRQRGSTSYVYDDSAGEGTCSYIIDTGIDATHPQFGGRAQNIKSFVNTATDGNGHGTHLAGVIGSAIYGVAKKTKLYGVKCLDDQGSGTTSNVIAAMDFVAKDAKTRGCPKGAMANMSLGGGYSAAVNKAAASLVASGVFVSVAAGGSGTDAKNASPASEPTVCTVGASTEKDERASYSNYGPVVDIFAPGVSILSTWLNGASNTLSGSSMSAAHITGLGAYIAALEGFPGGEKLCKRLQELATKGVLTNVPSGTLNLLAFNGNPSG

>gi|672376853|gb|KFG79136.1|_subtilisinlike_protease_PR1F_Metarhizium_anisopliae

MKLSVLLALLPLALAAPAPVIVPRAGSPIPGRFIVKMKNENLQQLVDTALKLLRKDPAHVYKFAGFGGFAADMADDIVELIRNLPGVEYVEQDAVVKANLGEIDSIEKRAFTTQSSSTWGLSRVSHINRQTSGTSYTYDSSAGQGTCVYVVDTGIETSHPEFEGRATFLANFAGDGQNSDGNGHGTHCAGTIGSKTYGVAKKASLYAVKVLDASGSGSNSGVIAGINYVANDAKTRSCPNGAVGSMSLGGSKSTAVNSAVANAVTAGVFFAVAAGNDGADASRYSPASEVSAFTVGATDSSDRVASFSNYGTLVDMHAPGVSILSTWLNGGTNTISGTSMATPHVAGVAAYILALEGKISPAALSTRLTTLATKDKITGLKGSTKNYLAFNGNPSG*

>gi|672376854|gb|KFG79137.1|_subtilisinlike_protease_PR1E_Metarhizium_anisopliae

MQLSLVIALLPLAMALPAPVIVPREGAPIPGKYIVKMRNDLKTDNLDNLITAALKVIKKDPAHVYKFGAFGGFSAEITDDVVETLRNMQGVDYIEQDAVVKANLGTSEPLQKKAYVTQSSATWGLGRISHVARGTSSYTYDSTAGAGTCSYVIDTGIATSHPEFEGRATFLANFAGDGSNTDGNGHGTHCAGTIGSKSYGVAKKTTLYAIKVLDASGSGTNSGVIAGINFVANDVRTRSCPNGAVANMSLGGSRSTAVNSAAANAVSAGVFMAVAAGNSAVDAANSSPASEPTVFTVGATDSSDRLATFSNYGSVVDILAPGVSILSTWLNGGTNTISGTSMASPHVAGLAAYLLAFEGKKTPAALSTRIQSLSTKNKITGLPSSTKNYLAFNGNPSG*

>gi|672377000|gb|KFG79277.1|_subtilisinlike_protease_PR1G_Metarhizium_anisopliae

MRPGLLFLQLLPLALAAPGARRSEPAPILAPRGAVIENKYIVKYKKTFSIASADHTLKACSAGADRVYSNIFHGFSGTLNESAIEQLRHHPDVDYIEKDAIFKMNTFVEQRDAPRGLRRVSHRKGDIGGYVYHESAGEGTCSYIIDTGVDDSHPEFEGRAQLVTSFVDGEDADGHGHGTHVAGTIGSRSYGIAKKTQLLGIKVLSDQGSGNNSAIIAGMDFAVQDARQRSCAKGVLANMSLGGRYSQSLNDAAAQMIQSGVFLAVAAGNNRQDASGYSPASEPSVCTVGSTDSSDSLSSFSNYGSVVDILAPGSDILSTWPGGSIKILSGTSMATPHIVGLAAYLAGLEGFPGAQALCKRIQSLATPGAISNVPGGTLNLLGFNGNPSG

>gi|672377426|gb|KFG79696.1|_putative_subtilisinlike_protease_Metarhizium_anisopliae

MFPSFLLLNLLPLAIAAPAKRAEPAPLLVPRGDTIPDKYIVKYRETFSISSADSIIKAHHAEAEKVYSHVFNGFAGALNATAIETLRHHPGVEFIENDATVKISAFIEEPGAPWGLSRISHRRGPGGSYGSYAYDDSAGEGTCAYVIDTGVDGSHPDFEGRAQLIRSFINGENYDGNGHGTHVSGTIGSRSYGVAKKTTIYGIKVLSNQGSGDYSGILAGMDFAIQDSRQRRCPKGVVANMSLGGGYSAAINQAAAQMIRSGVFLAVAAGNDANDASNTSPASEPSVCTVGATDSLDRLSSFSNYGAPLDILAPGSDILSTWPGGGTNSISGTSMATPHVVGLAAYLASLEGFPGAQALCERIRSLATPSAIKGVPPGTVNLLAFNGNPSG

>gi|672377960|gb|KFG80219.1|_Subtilisinlike_serine_protease_PR1J_Metarhizium_anisopliae

MFPSLLLLNLLPLAIAAPAKRAEPAPLLVPRGDTIPDKYIVKYKETFDISAADSTIKEYHAKAEKTYSHVFNGFAGALNATSIETLRNHPAVDFIENDATVRISAFVEQPGAPWGLSRISHRQRGGSSYAYDDSAGEGTCAYVIDTGVEASHPEFEGRAEFIRSFVAGENSDRNGHGTHVAGTIGSKKYGVAKKTKILGIKVLSDQGSGDYSGILAGMDFAIQDSRTRGCPKGVVANMSLGGGYSAAINQAAAKMIQSNVFLAVAAGNDAKDASQTSPASEPSVCTVGATDSSDRLSSFSNYGAAVDILAPGSDILSTWIGGITKSISGTSMATPHIVGLGAYLSSLEGFPGAQALCERIRSLAIRNTISGVPGGTVNLLAFNGNPSG

>gi|672377992|gb|KFG80250.1|_hypothetical_protein_MANI_011173_Metarhizium_anisopliae

MDIQRSAAARSAAPRTTRYSPNIPEYRFGHVSDAIFENDIEELHRVLSSIRKMPPASVYQEIVPIWETKRNFVHAVLEWGDRAHKFQVLQAAASHIYRHDEDENLVHNSVAEEISICILTQSPDVIDHRHHDGQPTVLHIAAKSRSEALAQTIFSNMQNNERFDQLLHVRNNQGSKTPLRIAVENDSLFFVRQILRYNTRPINDSKLLKWVLNEGTSEALQTLIELRPEEMDESVLQHALKMKSERLINALKNARECRDLFFRKRGFLHQLVKDGETLLVNTLLDKFPELALELDEEDKPVLSYNSDESIRDRVAGIILRTLPSMCPEKRQRYYRLCADNSSIPSTSEIVRALIDDVPGKEISLSLGGFGSWVSHAESFLGLIQKSNSYTSPDDKPSNALLQLMFEKSLIFVDIPITDLPAPRTDGKTTLIRSEVWSILNWLKRTKHVEGIYELSIRDSCYLPHAENVIRACLKDFNISVLDWRCPDLSLQVLHDGDESKTPICPDLETLRLYARGWPALAYWTSDESLYLLGRFTRLKQVEIFVLKEFVGMTLCETYAAEATERFDKFKTAHGNAVTFQLEITPKRWSDLSPTDQRPVLRRETTAVEVTKLGDFLLAYESIHRDFANQAWRNDRLEKGILCNELDESRKHTPYIRVAIIDNGVDPESIHCHKITGASFVPSHTGESNWWYIRHPHGTKMARIVTDLNPHCHLLVAKVGDSRSDFTAARIIKALGWAVAAGADIISLSLTLDKEDVNLELAVNKAAASGAVILASVRGEGVNTEMKPIPAAYGNVLAIGSADGTGAASSGTLEGQARHLFPGERIVARTEYLGGLDDAPDVSGPSVATAVAAGVASLVLSCNRFALFKKRDFKRPDYHHTLQVNVVKQVFEQMSDGKYVRPWVFFKDEKAKPSWGEGDSVLDWIQGKYRGIKDDGRA

>gi|672378693|gb|KFG80924.1|_intracellular_serine_protease_Metarhizium_anisopliae

MTLDDEDLTLDNEDEFHLLSEGATDEDQVRIQFENDVEDARRLSQTKFQDGDEEREQRTRFVLDRTPQWSRTTHDGRNFLHHLASYDYNRKPFVSLQWLMSRAMNKLPHLMGAMDRSRRTPLTTALAAGNVWFSYAACKNQKDETRQQFGAALASECEDLDNDRGSTCLHTALVCPMSGEALRGEIVKIMCGFVPRSMFSAVDVKGRTPLHLAVEYERCCRVQVGIVDELLRRGPGALDVEVAAYSGRAMSVYQYHEYTRRRAESRKNPARGRKEVREGGRSAAANPRPDLRAAAAPERPDKMVMGPPLPRDRTEPLPGLRRRVSMPVAAATPDGKPPSLSLNTQHPCPPAAGVDESPLDAPVNGIDAALQRDEERDQAAGQIAQLLKLFYLRTQMPERASRCLHVQDEQDKELWFDFGPPKKLTKTDFKKHFGHLQFDSALQYVAFPQSTLDKGEDNRHVLRQGRTDMVFFFEWLAQKGVERIIKVIVEDLKAPSHSDQAIEKSLKPFNVEMLDWRRADLDPVSLARIGQCLREVHLYWSGRNTVLRAWSEKEGLALIPTLETIYIVQVEGLEPETRVRENLDAFERRLGESWPTEAKPKVYIQLLGAGGPLPSLTQPSDLQPRRQRPVDPHKWMQCMEEFASHFRQIRALNDKSADPALAPVKVALIDDGADITHPDLKGMKFPGKSFHHYREGSSWRVSPFWDSSSGHGTLMARLIHRICPSAVIHVIKLSTFAGEASAKLQINTDSAVQAIEYAVEQGAQIISASWTVKPPTEAGRKKAFDDAVHNALNTKGALMFCAASDQGKSADLTYPHGSNPNSFRIGAARATGSALDNVGDGHELSFLFPGHEVVVDSAYEDVPDKQFGRFAPHSGSSVATALAAGLAALIVECVRLGVLYTGETGPLDETVTIGRDDLVRICERRQMEYALASIGTSRNTDNKYIEVWNTFGAAADKLKHSEGDRMSQLEIIAGLARLFLRKGA

>gi|672378963|gb|KFG81188.1|_Subtilisinlike_protease_PR1H_Metarhizium_anisopliae

MFSSLLLLNLLPLAIAAPAKRAEPAPLLVPRGDTIPDKYIVKYKETFDMSAADITIKEYHAKAEKTYSHVFNGFAGALNATSIETLRNHPAVDFIENDATVKISAFVEQPGAPWGLSRISHRQRGASSYAYDDSAGAGTCAYVIDTGVEASHPQFEGRAEFVRSFVAGENADGNGHGTHVAGTIGSKEFGVAKKTKILGIKVLSDQGSGDYSGILAGMDFAIQDSRERSCPKGVVANMSLGGGYSAAINQAAAKMIQSGVFLAVAAGNDATDASQTSPASEPSVCTVGATDSSDRLSSFSNYGAVVDILAPGSNILSTWIGGATKSISGTSMATPHIVGLGAYLASLEGFPGAQALCERIRSLAIRNAISNVPASTVNLLAFNGNPSG

>gi|672379151|gb|KFG81371.1|_KP43_peptidase_Metarhizium_anisopliae

MSTQVIVNGNELSTAVASTSDTSSTNFIVVRGTRRIIADDKRKLENKGATVTEYLGNDIYLCHYEPSNLQPIRDLEFVAQANIFPHYVKKSRSIVDAIKSSESEGHPQKTFDVEIFLHKGEKVSDSFINDLREKTGIVPKDIVAEKNVIKLTADASMIDKIVEVDAIKTIERSIKPKLFNDISREDIGIVYPRVSPLGVNYEGQGQIIAIADTGFDIGSKDNPHPAFTGRIVALISNDRRNGKTDDPVGHGTHVAGSALGDGSSDTMGGRIQGAAPKASLVLQSILDSDDVNIRIPRDLTGLFEEAYQYGARISCNSWGASWPSYQIPYDVGAWTVDNVICHKQDHVIAFAAGNDGEEMSLYSQMSQIGSTSAAKNSICIIKPDVVAPGVAILSTCSRHEAVATRRQDFGQTHDNNWMFCSGTSMAAPLVSGCFAVLREALQSNKMADPSAALIKALLVNGADDLRLPKPDQGFGRVNLKESLRCVVESGPGEQVGWEQFGFEDVGGENVLKEDETWPGEPKKIDFTGDRTRTLKITLAYSDPPGENLQNNLMLKVVIEQQSGVSEEQRGDEGFVAENNVEQVKWDITSKDRAAIITVTADRIAQLEDSQPFAVAWGIY

>gi|672379152|gb|KFG81372.1|_serine_protease_Metarhizium_anisopliae

MRLSVLLSVLPLVLAAPAIEKRAEPAPLLVPTTKHGLVADKYIVKFKDGSSLQAVDEAISGLVSNADHVYQHVFRGFAATLDKETLEALRNHPEVDYIEQDAVVKINAYVSQTGAPWGLGRISHKARGSTTYVYDDSAGAGTCSYVIDTGVDATHPDFEGRATLLRSFVSGQNTDGNGHGTHVSGTIGSRTYGVAKKTQIYGVKVLDNSGSGSFSTVIAGMDYVASDSQTRNCPNGSVANMSLGGGYTASVNQAAARLIQAGVFLAVAAGNDGVDARNTSPASEPTVCTVGASTSSDARASFSNYGSVVDIFAPGQDILSTWPNRQTNTISGTSMATPHIVGLGAYLAGLEGFSDPQALCARIQSLANRNLLSGIPSGTINAIAFNGNPSG*

>gi|672379263|gb|KFG81480.1|_subtilisinlike_protease_PR1I_Metarhizium_anisopliae

MQLSVLLALLPAILAAPAVEKRAEPAPLLTPRGVDVVADKYIVKFKDGIARIAVDESMNILESKADFVYETAFRGFAGHLTKAELQTLRNHPDVDYIEKDALMHINAFVEQPGSPWGLGRISHRSKGISSYRYDGSAGAGTCAYIIDTGIEASHPEFEGRATFLKSFVSGQNSDGNGHGTHCSGTIGSKSYGVAKKTKLYGVKVLNNSGSGAYSAIIAGMEYVANDYPSRGCPAGAIASMSLGGGYAASVNRAAAALVRSGVFLAVAAGNENRDAQNTSPASEATACTVGATDINDNRSSFSNYGRVVDIFAPGTNILSTWIANFIPPWDQRSISGTSMATPHVAGLAAYLSALEGSSNPAALCGRIQSLSTKNAISNIPSGTVNYLAYNGNGQ

>gi|672379712|gb|KFG81922.1|_subtilisinlike_protease_PR1E_Metarhizium_anisopliae

MHLSALLTLLPAVLADPATIGRRAEPAPLFTPQAESIIAGKYIVKFKDGIARIATDDASRETCKRVDFIEKDAVMRISSITEQNGAPWGLGRISHRQQGSTTYRYDDSAGEGTCVYIIDTGVEVSHPEFGGRATWLRSFINGQNRDGHGHGTHCAGTIGSRSYGVAKNAKLFAVKVLDDQGSGSYSGIISGMDFVAQDSKSRNCPNGHIASMSLGGGYSASVNQGAAALVRSGVFLAVAAGNDNRDAQNTSPASEPTACTVGATASDDSRSTFSNYGRVVDIFAPGTGILSTWINGRTNTISGTSMATPHIAGLAAYFSALSGKTSPAALCQKIQDTSTKNVIRNVPAGTVNFLAYNGNGA

>gi|672380775|gb|KFG82971.1|_subtilisinlike_protease_Pr1B_Metarhizium_anisopliae

MHLSALLTLLPAVLAAPATIGRRAEPAPLFTPQAESIIADKYIVKFKDDIARIATDDTVSALTSKADFVYEHAFHGFAGSLTKEELKMLREHPGVDFIEKDAVMRISGITEQSGAPWGLGRISHRSKGSTTYRYDDSAGQGTCVYIIDTGIEASHPEFEGRATFLKSFISGQNTDGHGHGTHCAGTIGSKTYGVAKKAKLYGVKVLDNQGSGSYSGIISGMDYVAQDSKTRGCPNGAIASMSLGGGYSASVNQGAAALVNSGVFLAVAAGNDNRDAQNTSPASEPSACTVGASAENDSRSSFSNYGRVVDIFAPGSNVLSTWIGGRTNTISGTSMATPHIAGLAAYLSALQGKTTPAALCKKIQDTATKNVLTGVPSGTVNYLAYNGA

>gi|672380805|gb|KFG83000.1|_subtilisinlike_protease_PR1D_Metarhizium_anisopliae

MHLSALLTLLPAVLAAPATIGRRAEPAPLFTPQAESIIADKYIVKFKDDIARIATDDTVSALTSKADFVYEHAFHGFAGSLTKEELKMLREHPGVDFIEKDAVMRISGLTEQSGAPWGLGRISHRNRGSTTYRYDDSAGEGTCVYIIDTGIEASHPEFEGRATFLRSFISGQETDGHGHGTHCAGTIGSKSYGVAKKAKLYGVKVLDNQGSGSYSGIISGMDYVASDSKTRGCPKGAIASMSLGGGYSASVNQGAAALVNSGVFLAVAAGNDNRDAQNTSPASEPSACTVGATDSSDRRSSFSNFGRVVDIFAPGTGVLSTWIGGSTNTISGTSMATPHIAGLAAYLSALQGKTTPAALCKKIQDTATKNALTGVPSGTVNYLAYNGNGA

>gi|672380985|gb|KFG83178.1|_hypothetical_protein_MANI_002863_Metarhizium_anisopliae

MESHIRRPSSTLNVCDAIYDGLLVIKRNSEELLRAAETSVTHQDALRFVKTATSLLIKIFPDMKRSLDLNVITIQIHHIACDLELHLTLPEEINSTDMVVALCQAEKDVRMLHYPRLIAHTARFGDILGSVERCKLLQETLTTAYDALDPVLPEDNIDDNDAESVACSTPYDDLFHSSADRLFHAIFPCESRGSKEVRLRLGAFRSVHPVDDYRSLDILVQRKDETGQFWLTISIHFPRSDMPKFQNSPKCSISPNGAPERRVAFANTEKQSCPWRTNKPFKVGCIWSCAEALNSEFKKWLQHLEFDKSDAWLLHKPVELPEAYAEEECFSDLLSQMTWTDVDKIALAMQLSYALSYHYDDSWLSGRWQQANICFFRWGRRIPCKPWLRVKSLPNQPPQAAPESHFHRFPQLLELGTILLELQIGQSLEASLRKNRARNLDEQWAYATSVFYGKLNNGRRLLSRHYQRAIEFCLRPDESLTQADSIRRSIYENVVRPLEQSIAEADLDDKLFEELDLGSLERTAVRSSIFAEQTEQALPPAADEMINVVSIPQPEEEFAADEIDEGFDLFGDEDGLQPGQEARKASNDWLAAFEKIIQEETRNSKPVSQPLAGQPVGGQNVGDGQQVRRVKVALLDTGVDLKHSYFDNQYPDGQIKSIRSWIGGKDGVEDKDGGDFSGHGTFIASILLKYGPNIDLYVARVAGTRRFRRGTSENVANALYCARVEWGVDIITMSFGYPLGHRGIRDQIIKATQNNILVFAAASNDGRNRPRMFPARQLSVFAIHSTNGHGHKSHFNPPPQRDENFSILGEYIESAWLTGPAEGSGATRCLSGTSFATPIAVCLSSFLLIYVPFILPEHKNFFYKMNTYEGLRNVLQAIVTVDESDAGSRYQYLGVERFFNENNRTAIKEIIRKALSI

>gi|672381324|gb|KFG83510.1|_subtilisinlike_protease_PR1F_Metarhizium_anisopliae

MRPVVFLSFLSLAAAAPVLQARDGKTIPGSYIVMLKDGGVSAFSEGFVQLMSGIEKTHDFDALNGFAAQLSDDELKALESSPEVDYIEQNAEVSINSYVTQSGATWGLARLSSSKTGSTSYTYDDSAGKGVCAYILDTGIRVTHNEFGGRAIWATNTIDSATTDGNGHGTHVAGTVAGTTYGVAKAATLYAVKVLNNSGGGTTASVIAGLNYVVTDSPKRSCPKGVVVNMSLGGGNSPSLNAAARAVVNAGHFLAVAAGNSNTNAANTSPANEPLVCTVGSTTKTDARSSFSNYGPVVDVFAPGTDVTSAWYSSNSATNTISGTSMATPHITGLGAYLLGLLGSRSPAALCSYIASTAQSGIISGIPSGTVNLLAFNGNPTA*

>gi|672381875|gb|KFG84041.1|_hypothetical_protein_MANI_029750_Metarhizium_anisopliae

MLPFRPHQLPRTTNPSQSRPVEEWINDIYLGSYEQGNDPAVDCMCLPIRLAPESRIRRFRNDESGTAFLMRRNVDGFVLASVMIVYAFPSISHKDMGREHEYINALADHHGQATMYEIGFLWVAPEMAVLLAQDYDMQNIVAAIISPLTCWRIMPHQTFSVIIFWDEEENLRAYIPDPQSMNGDDPAYIEATNLSEVFPTCKQTLSSASQQERNLGTTLFGYQNASAGADDRANIGADDFFTDAHHMKGVQSFFSTIRRPHQRRVKIAVLDTGLDINHPLLQKFVRSKQIGEELGRDFTRHPLGEPDLKDNTGHGTACTHLLLKTCPTAVVYTAKISNQSTFDEKTAERISEAIQTAITEWEVDIISMSLSYEYEVEIIDEALKNRQGDKKRPVLFFAASGNFGKDKDEPSAGFPARHENVICASSSTHQGNKSDFNQGPDYMNRWKNFSIIGENLCVAFPAELNRGNYEKRVSGTSMATPIMAGIAALVLEFCNIWKERGGRPTLEKAATMQGMLQIFHGCMLSNSNSSSQQGHLNLVPWYLFDGSSYTRDYASVGNHIAEALRRL

>gi|672381981|gb|KFG84128.1|_subtilisinlike_protease_PR1K_Metarhizium_anisopliae

MRSFAVLSLFTLASAAPLLKARSGTPIPGAYIVVLKNESADEFRIQSAIMSTVEKTAEWKQEEFNGFAAKLSSEELQALQDAPEVEFIEEDAEVSINAIVTQTGAPWGLARISSTARGGTTYRYDDSAGVGTCSYIIDTGLYAAHSDFGGRASQVANFVDSSNTDGNGHGTHVAGTIGGTKYGVAKRTTLLGVKVLNASGSGTNSGVISGMNFVVTDARTRSCPNGVVVNMSLGGSTSTAVNNAARAITSAGHFLAVAAGNSNANAASFSPASEPSACTVGATTSTDAKASYSNYGALVDVFAPGSGILSAWIGGTTASRTIDGTSMASPHIAGLGAYLLALRGPQTPAALCSYIATNANRGVITGLPSGTVNALAYNGA*

>gi|672382026|gb|KFG84169.1|_putative_subtilisinlike_protease_Metarhizium_anisopliae

MLFSAAALLALLPAALAAPATSGPLDKRAPIISARAGKVVPGKYIVKLKDGASDAVVNKVLGKHKADQIYKGGKFKGFAGALDDASLEAIRYLPEVEYVEEEAEFTINAVVSQTGAPWGLARISSRTPGGTTYRYDDSAGAGTCSYIIDTGIYTAHSDFQGRAIWGSNHVDSSNTDGNGHGTHVAGTVGGRLYGVAKKTTLIAVKVLNASGSGSTSGVVAGINYVQTNFPSRNCPNGTVANMSLGGGYSASINTAARNLVSAGVFLAVAAGNDNANAANYSPASEASVCTVGATASNDARSTFSNYGAVVDIFAPGTNILSTWIGGTSATRSISGTSMASPHIAGLGAYLLTLQGRRTPAALCSYIASIATNNVITSVPSGTINKLAYNGVA*

>gi|672382339|gb|KFG84469.1|_subtilisinlike_protease_PR1E_Metarhizium_anisopliae

MKLSAVLALLPLAMAAPSAPIDKRAPILEARAGTQAVPGKYIVKLRETASDDDLDKAVKKLGNSKADHVYKHAFRGFAGRIDDKTLDDIRSLPEVEYVEQEAVFTINTYTSQSSVPSWGLARLSSKTTGKTTYVYDSSAGAGTCAYIIDTGINTAHSDFGGRATWLANYAGDGINSDGNGHGTHVAGTVGGTTYGVAKKTQLYAVKVLDSNGSGSNSGVIAGMNFVAQDAQSRNCPNGTVANMSLGGGYSASTNSAAAAMVRAGVFLAVAAGNDGANAANYSPASEPTVCTVGATTSADAIAYYSNYGTIVDIFAPGTSITSAWIGSTTAKNTISGTSMATPHITGLGAYLLTLLGKKSPAALCSYIASTANSGVISGIPRGTVNKLAFNGNPSAY*

>gi|672383277|gb|KFG85392.1|_Subtilisinlike_serine_protease_PR1J_Metarhizium_anisopliae

MHFSVALLALLPAAIAAPTAEPIQKRAPIIAARVGQVVPNKYLIKLREGTSDDALEAAIGKLGKSKADHIYRGKKFRGFAGKLEADLLDQIRLLPEVEYVEEDAIFSINAYVSQSGAPWGISRLSHKSAGSTTYVYDSSAGEGTCSYVIDTGIYTAHSDFGGRATFAANFVDSSNTDGNGHGTHVAGTIGSTTYGVAKKTKLYAVKVLGSDGSGSTSGVVAGINFVATDAPKRSCPKGVVANMSLGGGYSASINQAAAALVDAGIFLAVAAGNDNANSANYSPASEASVCTVGATDSSDRKASYSNYGSVVDIQAPGTNILSTWIGGRTNTISGTSMASPHIAGLAAYLLALEGSKTPAALCNYIKSTALSGAVSGLPSGTTNRLAFNGNPSA*

>gi|672384588|gb|KFG86683.1|_subtilisinlike_protease_Pr1A_Metarhizium_anisopliae

MHGASTQKNAPWGLARISKKLPGKDHTTYTYDESAGEGTCTYVLDTGIEVDHPEFEGRARFVQNFVDNADLDANGHGTHIAGTIGSKTYGVAKKTQLFAVKVLNEYTAGQTSGILAGIDFIVEDATTRNCPKGIVVNMSVSVASSPAINAAARYIVKSGYFLAVAAGNDDTDASRVSPSNEPMACTVGATAQNDTRASFSNYGVSVDVFAPGVDIKSTWIKGGVKLESGTSMATPHVTGLAAYLLGLKDIKAAELCNLIASMSLKDVMKGIPENTVNLLIQKGEAM

>gi|672384945|gb|KFG87036.1|_subtilisinlike_protease_Metarhizium_anisopliae

MHHIQINGNSRPDAGAASTASLSTFAVPPPSVAISNYILIQTKGPLSAQQKQALRDKDVEILEYKGEDVYLCGYKPTTLEPLISNLRDFVTDAEVYHPDYVVEPDLKTGNDDDEAEVEVALHDNIGDNDMSRVANDIAAQADIPVANVELHDRKACVKVPKSKLAQLARIDEVKAINEVHTRRIFNNVACGILEARTPVGTANTTYKGKGQVVCVADTGFDTGSLAGYHEAFGDRVISLHARGRPAAQGEPGNSDDPDGHGTHVCGSVLGKGDHAEEGEIEAPASEAKLVMQSLFDKFDKSRGPDPKSWNAGLGGIGSSYPDLFGGVFREGATIHTNSWGGPPQPYVERESARIDSYLWGNKDMTVLFAAGNSGVDIDRNPGHVDPYSLSAQAVSKNVITVGASENCRPNVKSPVAGNKPLVYGAWRSKFPFGPISVDNVADNPEGMAAFSSRGPTRPDGRIKPDVVAPGTTILSARSSQIEPGHHGESWGHSSDARWMYLGGTSMATPLVAGCCAVIRGALVDNHVPRPSAALIKALLINGTVPMKGQYNQALPNGEFGPSVDAPNPNSGFGRVNLANSLRNIVANQESTVYGYQDVTGDQSLGMDGQHRGEHAVSVDIPEGSPAALTLKVTLVWTDFPGERLQNDLDLIVAGAGVEKHGNQGDGSGFDRVNNVEQVVWKNVKPGSYTVTVRACRTTKDPQPFALAWRAFA

>gi|672385959|gb|KFG88044.1|_S8_family_Peptidase_Metarhizium_anisopliae

MGHPKSRVFASEMLASVASEASDILFPIAIANMSASAGEIATRLRYIGGDLLEQQLYQLPPDIQSLCLQALQDLLKICKWPVLSTAVSHFHSSESPQELRDVLIRLCEKEQFNNGVQRSITKFCQERADTIKPYQLRFVQLSDKLLKNAQEIGRSANDAGPYLHLRPLGHDVYPDDVYKLLLQGIKSLATCAHQYHNAPALSGFTGDDWHTTMLCLNSGIRSENKRALFNIITATSEMVYWQEMGVTVPIKDIEPNDVQRHQQAKRVPFDPSKPSLMEYGDICKRLENPSYAKLYLDLDEDCQLCERPDPVGLQHIITGCGINLSELLSLTELTVEHKIKLSYTVARAFWQFYNSELMNARWTSEDIVFIPLNKEFSPTEGIPLRAFVPFPFGPRYKKSPQEFCQENQFTHRYPRILYLGIILLEIGLGQALRLEHNPKLSLLAHINTAHAKAKMKLKELDDAEWDGFRWKEYFVEAVRNCFDSTNFKKSPRLRSPRQRGGSEGADNDAKDSAFLERRDSLYQKVVAPLMWLATTGFEDSEEVPLVPIRKKIRRQPTLAKNGDEELQTFWNEIHARPSLMSGSSISTEGFLEDLQIIAGHIARCRRLAKATKPIRVAILDTGCHRGLRFFQNPQRFNRLKGWKDFTSAGSESEIDTFGHGTFMARLLMHVAPIIDVYVVRVAENTEDLETQENSIAKVCAAPLASDVLTIIQAIEFAALDPDWNVDIISMSFGFPNKPGVKHAVISDAIDKIKKDRNDSVLFLASAGNSWERRVDFPASHQEVIPIYAADATGAFLRSNPARAGKGSEKLGTYGTDIPTSILEEIQEAFPKAALSAGTSLATAIAAGIVAMMLSYIEALPALLQLRGFQEVCAKLYTKRGMENMLHAMSLTTGYRQQFINPVHFWGEKKTDVDVFISICSAVEKMNNET

>HCAG_00443.t1_gene_HCAG_00443_Acap_NAm1_scaffold_1:complement(1525027..1527075)

MPSICINGNTLDPSTESRQLMDFGMIADDASKSDYVLIQASSDCLSTEEHGALQTLGVNIMEYVSEGTFLCSYKGTDLTQIRSLEFISWANTYCDQFVVQGSLKSRTPQVNQLSAFTATPKTSHLQLVDIIVHHDVDPNDDSVREAVAKAARADLKCLEATSQGFRLQVQQEYLDDIAAIDQVYLIQQVHPFVLWNNKAREIVGCSGTSTYTEPGDCQGEGQVVVACDTGFDIGDKEDTHPAFKGRVDKLYDMGRKGRSDDPDGHGTHVAGSVLGNGESKSMGGSIMGTAPKAHLVLQSVLDGSNGLGGIPRDLTQLFRVPYEECGARIHTNSWGSASPFGQLPYDASARQIDDFVWKHPDMVILFAGGNDGVDANRDGKIDQGQIGSQASAKNIITVGASENNRPDIPVTYGSRWPVDPLCNDRMADNPKGMAAFSSRGPTVEGRFKPDVVCPGTAVLSTLSRRAQMTSRFGQSSDPLWMFLAGTSMATPLAAGCVACVRECLAKNGVKSPTAALLKALILHGAMELVGQYTPSEAGPSPNNSSGWGLLNLTNSICIASGRGGGYLEGKALGHGEKAPTLTLTVPPNGTLKVTLVWTDPAGPHLQNDLDLSAIALDGQERHGNMGTGSGYDRVNNVEQIMWNNPPEGKVDITVKAFRITRPSSPQPYALVWSTTWPPDPKA*

>HCAG_00635.t1_gene_HCAG_00635_Acap_NAm1_scaffold_1:complement(join(2165746..21659222165988..21669262166994..2167365))

MRSATLLALLPFALAAPSASVSRRTEPAPVIRPRGVKLVDGKYIVKMKAGVRAASVDSAVSTIQADADYTYTKSFSGFAASLKDEEIETLKHDPNVEYIEQDAVITIKATVDQDNAPWGIARLSSSKPGSKTYTYDESAGEGTCSYVIDTGIDVEHPDFDGRAKFLKNFAGGRDGDGQGHGTHVAGTIGSTTYGVAKKTTLYAVKVLGDDGSGTNSAVIAGMDFVAGHADDENCPNGAVVNMSLGGEASDAVNSAAKSIVDAGLFLAVAAGNEAVDASGSSPASEASACTVGATTRNDTLSYFSNFGDLVDVLAPGTDILSTWPGGKTNTISGTSMASPHVAGLGAYFLGLGKKAEGLCEYIASQALEGVVAQVPRDTVNKLINNGVSK

>HCAG_04060.t1_gene_HCAG_04060_Acap_NAm1_scaffold_4:join(127681..127761128137..128197128267..128451128505..128571128605..128678128742..129180129254..129433129610..130179130251..130425130506..130671130)

MSPPSKCFKTILISEYSQSRDHSRQATPNPPYMVDNQHNYYKTAPDKGEQNGPVWVESGSFEAYMSQHLKQLNRASVDVSEDLNKAAGSAPNTTAASSYWLPKLARLGVQPLAGADYKFYRDVVEYGADNTAIEDGDRCGLECGNTFAKGAIIYFPPGTYKICRPIIQLYYTQFIGDALDPPTIKGCDTFQGIALFDTDPYVPNGNGQNWYINQNQFFRQIRNFIFDLTEMPLSTADHDQPLVPTGIHWQVSQACSLQNLVFNMPKATDSNKVTHVGIFMENGSGGFVSDLVFNGGNIGWRAGSQQYTAMNLKFNGCLTAVQMIWDWGFNWQRIEVDGGAIAFNISGRGGDTGQGVGSVSIIVKSDDGDVILDGTNHVGLWAMGRRYDGYNGTYISGEVDAPKKGKRLLDDDGKLFYRPRPQYEDLKVDEFLIATEHDCKNDGTGDNTGDINAFLEKAKKERKIAYFPAGVYRVGGTVFIPTGSRVQGASWSQIQGAGFYFNDIHNPRVVVQVGEKGDVGDMEIVDMMFTAQGATAGAILVEWNVHEDSQGSAAMWDSHIRVGGAAGTDLDIETCPKFEFSDACICASLLFHITPQASGYFENIWIWLADHDNDKSVYDSPDKIANQISLYAARGTLIESDGPSWLYGTGSEHTVMYQYQVYGAKDIYLGHIQTETPYYQPVPIAPLPFSSAKEFPGDPSFEKCKTTGCSTAWGLRIINSEGITLHSSGLYSFFQEYYQDCVPTHNCQERILEVKGSKDVALFNTFTVGIVEIGTGINHGAIFQNDSNQSGFTTEVSVWIPLEGDDEYDIVYVGPEVYDKPSLTCPADCILVFPTSSLSSKTTIDPGKYTTSVEYGHRSTTTIGGREVPTFYTTVTTITLTIDPITTDGMPYSNINITEGQTSTPLTVLPSVDIPPIPVPIPDGEGKTTTRNITVPPWPAITRGPPESWNNPNASPTDGTKEGVYHTPFVTTVVATKPTVSTLSFPSTVSPIVVKCPPHSKVPFNTPKTTPTINCRVPTTVSIAFTCPATKVVTFIGSSTGVFTVDCTVSTTFTKPDQTITSGPTNKPPPPVWPTWPPRVVTPIEEEVKKPEPGKTPCKLWFFSFCPNGDEGETKGLRWNLPPGIYPPGPPPPRAIDLPPSWTIKSPLPPWPPITVGQDRILTYPKEEPTKCEKKSAEICMTTVFKTTTTRGTITSTASSTSSTCDTIRGCSASDWDTTTTQTKPDHCPSPTANPVKGAASIIPPPGCPANAIVYPSNMKDVGQIPQILAKYKGKYVEIKSIELQQVAFIWVPLLDQETMDVLLESPDVADAYYYETWYANTNRRGSDSDRRAKRDNTPPEPIFPYKQGNINHSTSNRAVLDEHNTNQSAQSTSKSRSNQGIWDLSQVSSPKKEVWNLPGGSTVDGNGDPKFNYDETSGRGQHIYILEDGYYKDHPEFRGVDIELLFPDDVNQQPWAPEVILSPRVDHGAEVASKVVGANLGFCDNCKLHVAPFFLLGDLWYERLIEQLMTIQNRVRRNGFQYKAVINMSFDLDDDVREACLRRIHEMLATLDSWGVSIVVAVGNVDPDFPDGVFYPALFGEPGSPFYMENLIIVGETDEDGNQGPSGLYRTWITTFAPGYGVWVPQTPTGGYTVKAGTSLSAPLVSGLIAYYRSIQSPWQDQLKDPANVKKMIKIFHRRIEVYGKQIDMTRAKPIIWNGQVDTRSCLADYDTIDEWDQGRRCPRIREKLEDETNEGETVEPCQPGTGNPLKELDGSYCPRMPGAGAGGHTVSFTSKAGVKPTPTCASGTGCGGHLCTGFFCSPMPTGVPPDRHDPKDPNASSRVPTTTGPGDPEPTCDDKCKLDKGNRCSCGENGCDSESPSCCANASCPMCECGGNTCSPSSPACCKTETCQWSWTGGGGGDGSGKPPGPRLGTLLFALDETYTSHPGGGVLTRFWKVFLSELHKEPNPCTSTPLIVVDASSDPENRPNYPPSIEFAVGPGHRCAYTGNSNGAGILECDGSKHASCKARGLDEKKLCFGSPPSTFYMAVDCYREAAELAVERSPVDWRQRF*

>HCAG_04281.t1

LKEPSKWLQIGLQVRLKELEKRLSNPLHKRYGKFLAAKLLHPGQDAINRTQAWLDWLRVIAEAERLLDTKYFIYRAMEWSLPQSLHDVIDTIQPTTSFTPLCLRTLYTRMALVNYLGEFNNRSDISQFLFEDISIGGNQQEGNLDAEVMIGIAHPTTYTVGESNEPLTWLNWILDQPDSELPSVVSTSYGDIEHVPYARRVCNGFAQLGARGVSVIMGSGDHGVGCYSNDGFLVSFPDSCPWVTSVGAEVVSSGGFSNYFPRPAYQSVARYLMFNPYGRAIPDVSAQGYVTIWNGVDGTSASTPTFAAVVALVNDALAAEDKPPLGFLNPWLYFRDINEGGCGFPALHGWDAASGWGTPFPKF

>HCAG_04284.t1

LIASSSWVCIGLQHGWETFEQHLSDPSHPRYGKHLVSLLVKAAPESLNAVTTWLDLVSFIEVVERLFMAKYAEYRSLSWSVPADLVNHIDVVEPTNSFSIVCLSVIYNSISLVNFLGEVNNRSDIDLFLFTTEIIGGTQSEGALDAQTILGLSWPTTYNVGSKNEPLAWLQYMQSKETLPHVISISYADTEQVPYARRVCKEFAKLGARGVSIIVASGDWGVGCVLDNGFAPSFPASCPYVTSVGAEIVSGGGFSELFSRPRYQAVENYLLFNRKGRGYPDVAALGFSVLWNGQDGTSASAPTVAAIIALVNDALLDKGHPPLGFLNPWLYFTDITWGGCGFPALTGWDPATGLGTPFPKL

>HCAG_06003.t1_gene_HCAG_06003_Acap_NAm1_scaffold_7:complement(join(1233383..12337561233832..12339371234008..12345471234625..1234813))

MRTATLLALLPLAFAAPSKRAEPAPVLRPRGVQLVDGKYIVKMKSGVRATSVSSAVSSIEADADYTYSKSFNGFAASLKEDEIEKLKHDPNVEYIEQDAVITIKATTEQDNAPWGLARISSDKAGGKTYTYDDTAGEGTCAYVVDTGIDVDHPDFDGRAKFLKNFAGGSDNDGQGHGTHVAGTIGSTTYGVAKKTSLFAVKVLDDNGEGTNSGVIAGMDFVAGHAADASCPKGVVVNMSLGGQFSSAVNNAAKSIVDAGLFLAVAAGNDGADASDSSPASEPSACTVGATTRNDTLAYYSNTGKIVDVLAPGTDILSTWPGGKTNTISGTSMASPHVAGLGAYLLGLGQKAEGLCDYIVSTALDGVVSSVGSGTPNKLINNGVGKN*

>HCAG_07015.t1

RNHDYSPAQLLGEGQIGELADHHTFSKRIADPMFHDQWHLFNTVQLGNDLNVTGLWLEGITGKESISAIVDDGIDMHSKDLKDNYFADGSYDYNDKSPIPKPRLFDDKHGTRCAGEIAAVNDICGVGVAYDSRVSGIRILSKPVSDEDEAAAINYKYQDNQIYSCSWGPVDDGMTMEGPGTLIQRAFVNGIQKGRAGRGSIYVFAAGNGALHEDNCNFDGYTNSIYSVTVGAVDRDDNHPYYSESCSAMLVVTYSSGVNIHTTDVCSTRHGGTSAAGPLVAGVVALALSVRPELTWRDIQYIFLETAIPDSDWQDTSIGFSHEFGYGKVDAYSAVHLAKWKLVKPQAWLHSSSFEVTKKMLKLHNQRLEHVTVTMNVNHTRRGDLSVELRSPAGVSHLSTTRKRDNNPVGYTDWTFMSVAHWGESGVGKWTVIVKDDWQLDLWGEAIDPNIQRKRIRYEFLYDAF

>Jan_02953

RNHDYVPTRHLGVDRVGELDDIWLVRHRVEDPLFPRQWHLVNERYPENMMNVTPVWDMGYTGKGILTALIDDGLDYTAADLAEKFDAENSYDFNDHVPLPYPKLEWQHHGTRCAGQIAASNNVCGVGIAYDSRVSGLRILGGRITTVDQATALNYGFQNVHIYSCSWGPRDDGTKMQAPRYIVRKAFLNGVNKGRGGKGSIYVFASGNGGRSGDQCNFDGYTNSIYSVTVGSVDYKGLHPTYSETCTANMIVAYSSGSGIVTTDRCAFSHGGTSAAAPNAAGVIALALQARPELTWRDVQHLCVETARRDRDWDRTAAGYSNKYGFGVIDGSLYVQRALWKLVDPQAWLQSSTISITKEMLEEANKGLEHVTIKVWIDHTRRGDVEVELVSPNGKSVLAQKRGRDEATTGYPGWTFMSVKHWGEKPIGDWTIRVSDGWNMILWGSTIDPSKARRG

>Jan_12317

RNHDYAPSNQLGVERVGELDDVWLVRKRIEDPFFIMQWHLANGDYPEHMMNVTPVWDMGYTGKGVITSILDDGLDYTSEDLKDNFDPDNSYDFNDHEALPYPKRVRDHHGTRCAGQIAAGNKACGVGIAYESKVAGAVLNGINKGRGGKGSIFVFASGNGAHKGDQCNFDGYTNSIYSVTVGAVDFRGQHPKYSEACAANMVVAYSSGSGIVTTDRCALVHGGTSAAAPNVAGVFALALQARPDLTWRDIQYLCVETARQDRDWERTATGYSYKYGYGVLDASLYVQRALWKLVKPQAWLETSKMTITKKMMQDANESLEHVTIKVWIDHTRRGDVEVAIVSPNGRSVLAGARERDDSMSGFPGWTFMSVKHWGEDPVGDWTIHVTDGWNMILWGTTVDPSKAGCGYDQD

>Jan02137

VKEPPGWLRIALQPHFNILEKHLSDPDHPRYGAYLVEKIIAPHPASLEAVNTWLDWVNVVALAERILQTEYHVWRTTSYSLPEDLHDHIELIQPTTIFTISCLKQLYTSIGVTGYLEEFANEQDLQSFFFKFVSVGGNNQEANLDVQFAFGLAYPTFWSTAGRNEPLDWVNFVLSQKDVPHAISTSYGEPEQTGRQLAQLSARGVSLMFSSGDGGVGCFTNDGFLPNFPATCPYVTAVGGETASGGGFSDYFRRPNYQVVPAFLLYNREGRGLPDVAAQGFRVWYRGIGGTSASAPAFTAIIALLNDARIAKGMPPLGFLNPLLYFNDITVGGCGFNATKGWDPRLGTPFIRL

>Jan02994

FIIFVGHPAHLLTVDKLHGILGQGIKIAVLDTGDYTHPFLGGGIGGKKIIGGFDFVPDPLDQCHGTHVAGIIGGVAPEAEQFAYRIFGCDVIIALFRGVDDGADILSLSLGAVVASRIAKIITIAAGNGSRGWYAASPSSAINAISVGSLENDACTIVRGGSTFSTYGPTFYFKPAISAPGGILSAYPGTSMATPAGAAALAMTETVTQTGSGLINVYNATTVTNDTIANGDAPKTYKLSHTAAVAPGYPVYSGFITYIGPNGSYRLLVRALRVTGDY

>Jan02995

FIIFVGRPVRGLDVDKLHGITGKGVKVAVIDSGDYNHPNLGEGFGGFKVAGGHDFVPDPMDECHGSHVAGIIAGVAPDATLYAYRVFGCTLIIAMLRAVKDGVDVINISIGSVVASRIAKVVVISAGNGMSGFYTSGPANGIDVISVGSVENDACVLVRNASNFSSYGPTFYFKPALAAPGGILSTVPGTSMSAPAGSAALASYQTAIQQGAGLVNVHKATLVSNDTLNNGDTERTYSVKHVPALAPGFPIYSGFVSYIGPPGDYRLLLRALKVTGDY

>Jan06m300_GLEAN_01910_gene_Jan06m300_GLEAN_01910.gene_ccin_Chrom12:join(1845541..18457601845807..18459061845966..18460311846090..18461571846211..18462331846291..18463401846400..18464351846504..1846...

MRSATLLALLPFALAAPSRRAEPAPILRPRGVKLVDGKYIVKMKNGFQASSIESWVDKMIESIEADADYTYSKGFGGFAASLKDDELNKLKHDPNVEYIEQDAYISITATTQQSNAPWGIARVSSQSPGGSTYTYDNSAGEGTCAYVIDTGIDVDHPDFDGRAKFLKNFAGGSDSDGQGHGTHVAGTIGSTTYGVAKKTSLFAVKVLGDDGSGTNSAVIAGMDFVSGHAKDENCPKGVVVNMSLGGETSDAVNQAAKAIVDAGLFLAVAAGNDGKDASGSSPASEESACTVGATTRDDTLADYSNFGSVVDVLAPGTDILSTWPNGKTNTISGTSMASPHVAGLAAYFLGLGQKAEGLCDYIASKALDGVISNVPSGTVNKLINNGVGGSNSSSIHH

>Jan06m300_GLEAN_06118_gene_Jan06m300_GLEAN_06118.gene_ccin_Chrom8:join(2338645..23388792338939..23390442339096..23391612339218..23392822339337..23393592339419..23394682339520..23395552339620..2339656)

MRSATLLALLPFALAAPSAPSRRAEPAPILRPRGVKLVDGKYIVKMKKGFQASSIESWVDKMIESIEADADYTYSTGFGGFAASLKEDELNKLKHDPNVEYIEQDAYISIAATTQQSNAPWGIARISTQSPGGSTYTYDDSAGEGTCAYVIDTGIDVDHPDFDGRAKFLKNFAGGSDSDGQGHGTHVAGTIGSTTYGVAKKTSLFAVKVLGDDGSGTNSAVIAGMDFVSGHAKDENCPNGVVVNMSLGGETSDAVNQAAKAIVDAGLFLAVAAGNDGKDASGSSPASEESACTVGATTSDDTLADYSNFGSVVDVLAPGTDILSTWPNGKTNTISGTSMASPHVAGLAAYFLGLGQKAEGLCDYIASKALDGVISSVPSGTVNKLINNGVGGGSNSSSIRH

>Jan06m300_GLEAN_06483_gene_Jan06m300_GLEAN_06483.gene_ccin_Chrom11:join(90112..9032890381..9047490528..9059390644..9070890760..9076590822..9083890897..9097991036..9107291145..9118591245..9126591327..)

MKVSALLALLPLLPVAVAAPTKRASPAPVLVPRGVQLVEGKYIIKMKGDSNIQSVNAAISSIRASADHTYSHSFNGFAASLTPEELEQLRQDPSVDFIEQDAIMTISATQSGADWGLARLSSQKAGSTTYIYDDSAGEGTCAFIIDTGVEADHPEFEGRAKLLKNFAGDGEDSDGNGHGTHVSGTIGSKTYGVAKKTQIYGVKVLDAQGSGSNSAVIAGMDYVAKEAQNQSCPKGSVANMSLGGSKSSAVNEAAAGITGAGIFLAVAAGNDGQDASDYSPASAESACTVGATTRDDELATYSNIGKLVDVLAPGSNISSTWIGGKTNTISGTSMASPHVAGIGAYFLGKGQKIDGLCEYIVQNGVKDAIKGVPSETVNVIINNGEGGGNSTRRHW

>Jan06m300_GLEAN_06495_gene_Jan06m300_GLEAN_06495.gene_ccin_Chrom11:join(142078..142318142375..142468142523..142588142644..142913142970..143294143360..143510143585..143619)

MKASTLLAILPLAMAAPSKRSFPAPVLVPRDAQLVEGKYIVKMKEKAAFSAVSSAISSIAADADYTYEAFNGFAAKLTDEELEKLRGDPNVDYIEQDAIVTIYATQQNADWGLARLSSQKAGTTTYTYDDSAGEGTCAFIIDTGVEASHPEFEGRATFLKNFAGDGQETDGNGHGTHVAGTIGSKTYGVAKKTKLFGVKVLDAQGSGSNSAVIAGMEFVAQEAKGQQSKCPKGIVVNMSLGGQRSSAVNQAAQAISSAGLFLAVAAGNDGKDASGYSPASEQSARTVGSGSNSAVIAGMEFVAQEAKGQQSKCPKGIVVNMSLGGQRSSAVNQAAQAISSAGLFLAVAAGNDGKDASGYSPASEQSACTVGATTKTDALAQYSNHGSIVDVLAPGSDIASTWINGGVNTISGTSMASPHVAGIGAYFLGKGASVQGLCDSIKQKGIRGAIQGVPGTTPNVLINNGEGSNSTSPIGF

>Jan06m300_GLEAN_11204_gene_Jan06m300_GLEAN_11204.gene_ccin_Chrom3:complement(join(1156204..11562471156296..11564461156501..11566861156741..11567671156822..11569361156989..11570091157069..115714611572)

MKGSTLLAILPLAMAAPAKRSFPAPVLVPRGAELIEGKYIIKMKAKAEVSAVSSAISSIAAEADYTYDTWNGFVATLTPEELQKLTDDPNVDFIEQDAIMTMYATQQNADWGLARLSSQKPGTTTYTYDDSAGEGTCAFIIDTGIEAGHADFEGRAEFLENFTQDGQNTDGNGHGTHVAGTIGSKTYGVAKKTKLFGVKVLDAQGSGSNSFVIAGMEYVAKNAKSKPCPKGVVVNMSLGGQKSEAVNQAAQAITKAGLFLAVAAGNDGQDASGYSPASESSACTVGATTKTDGLATYSNTGSGVDVLAPGSDIESTWIQGGIKTISGTSMASPHVAGIGAYFLGKGESIQGLCESIKQKGVKNAIQGVSGGTANVLINNGEGSNSTTPIRF

>Jan06m300_GLEAN_11245_gene_Jan06m300_GLEAN_11245.gene_ccin_Chrom3:complement(join(909874..910000910160..910298910358..910633910673..911003911080..911508))

MKGSTLLAILPLAMAAPAKRSFPAPVLVPRGAELIEGKYIIKMKAKAEVSAVSSAISSIAAEADYTYDDWNGFVATLTEDELQRLTDDPNVDYIEQDAIMTIYATQQNADWGLARLSSQKPGTTTYTYDDSAGEGTCAFIIDTGIEAGHADFEGRAEFLENFTQDGQNTDGNGHGTHVAGTIGSKTYGVAKKTKLYGVKVLDAQGSGSNSAVIAGMEYVAKNANSKPCPKGVVVNMSLGGQKSEAVNDAARKITGAGLFLAVAAGNDGKDASGYSPASEASACTVGATTKTDGLATYSNTGAGVDVLAPGSDIASTWIQGGIKTISGTSMASPHVAGIGAYFLGKGESIQGLCDSIKGKGVKNAIQGVAGGTVNVLINNGEGSNSTTPIRF

>KFG77777

LILFHGWPVHLMTVDKLHGYLGSGIKIAVIDTGDYNHPALGGCFGGCRVVTGENFRPIDCHGTIVAGILAGAAPNATIMAYRVVNCDDMIGWLKAKQDGAQIMVSSVGAVVAARIVVPCIVALGNKDHGFYALNPSTGRGVTSVNSFGRALIRGAAPMSAYGPTMDIKPTIGAPGHVPVTGTSFAGPAGVFALGAPVAQQGGGLLRAWEATLVENDTITNAKTEVTYRLSHLAATLGPGLPLWSGWVPYLGGAFPPGRYKIVARALSIMGHW

>KFG78015

VIHTSRVVRIALQRNLDKGMEYLSDPSSKNYGSHYVVDLFSPAPESIETVKRWLGWLDFVGQLEDILKTNYHLYGADSYSLPSEVSQHVDFITPGVVPTPACIKALYNQLGMFESDNEMHKQTDLDQFYPKIDLIWGTKPEAALDFDVSIPVIYPELYQTKSNDDPVDGVTANEACGTFTPANVISFSYGLTENWPTQRQCDEFMKLGLQGSSIVFASGDGGVACLGSNGFNPASPSSCPYVTSVGAESAGSGGFSNIWPSPDYQAVASFFIYNRAGRGFPDIAAIGGVIVLNGTGGTSMSAPIVAAIFTRVNEVRLKAGKKPIGFANPALYFKDVTLGACGFSAVEGWDPVTGLGTPFPAV

>KFG78468

YFVYKAHQVHVMMVDKLRGITGKGIKIGMIDTGDYNHPALGGCFGGCLFSFGADLVPMDCHGTNAAGIIGGAAPGAQLGMYRITCDVMVAIYRALADGVDIISSSAGSSAATRAVVVFVQGAGNGTLGFSHLDPAVGNGVISVGSVNSHACVLMNGASDFSSWGPSLGLKPSLTAVGGIISTDWGTSFSGPAALVALSNPAPVAQQGGGLARAYDATLVQNDTIKNGQGAITYRLSHVPATVDPGMPLWSGWIPYQGGQLPEGYYKLVVRALRIFGDW

>KFG79636

YIVFKGWPIHVMTVDKLRGVTGKGIKVAIIDTGDYTHPALGNCFGGCLVSFGTDLVPDPMDCHGSHVAGIVAGAAPNVTLGAYRVFGCDVLIAFNKAFEDGAQIISASIVAVAVSRIVVPCAISAGNGDHGFYISTAANGKGVTAVASYDNDACVLVRGASLFSSWGPTMDFKPQIGAPGGILSTYPGTSMSCPAAIIALANPAPAAQQGGGMVQAYDASLLSNDTITNGTNPATYRLGQVSSTVAPGLALWSGYIPYQGGKLPAGKYQFVTRALRIYGDW

>KFG80029

YIFFKGYPVHVMTVDKLRGITGKGVKIAVVDTGDYKHPALGGCFGGCLVAFGTDLVPDPMDCHGSHVAGIVAGAAPGATLGAYRVFGCDVLIAFNQAYQDGANIITASIGAEAVSRIVVPCTVSAGNGAEGFYASTAANGRRVSAIASYDNDGCVLIRGASTFTSWGPTMDTKPQFGAVGGVLSTYPGTSMSCPAGIIALANPAPVPQQGGGLVQAYDATLLSNDTLQNDRKEITYKITHTPATLNGGLALWSGYIPYQGGRLPPGKYKFVVRALRIFGDW

>KFG80585.1|_kexinlike_protease

RDYDYPPASRLHEGTVGALSDHHVFRKRIKDPIFTAQWHLFNSVEVGNDVNVTGVWMEGITGKNATVAIVDDGLDMHSEDLRENYFAEGSYDFNDHDPEPAPVLSDDHHGTRCAGEVAAVNDVCGIGVAYESKVAGIRILSAVISDEDEAEALMYKNDKNQIYSCSWGPSDDGRTMEAPSVLIRRAMLKSIQEGRNKLGSIFVFASGNGAKSGDNCNFDGYTNSIFSITVGAVSRDNQQTYYSEPCSAQLAVTYSSGGSIHTTDVCTDRHGGTSAAAPLAAGIFALVLEVDPELSWRDMQYLVMDTAKPGVVWNQTGIGFSHAFGYGKIDTYDLVQKAKWNKVKPQAWFFSANFTVTKDMLKEANERLEHVTVFMNVNHTRRGDISVDLISPSNVSQIATTRSGDEHYAGYVNWTFMSVAHWGESGVGTWTLVVRDDWRLKLWGESIDAKKAKRRYEFLYDAF

>KFG80701

QHEPQGWLSIALQPEIHRLASKFGSGHLVRTLRAPDPKDAAAVVDWLDWIHVVSTAESLLNTQLQRYRAREYSVPSHLSDAISFINPISNFTPKCLRQLYVRLGVSGYLEEHSNHADVRDFLFKVELVGGDPQEAQLDLEYVMGLGFPTYYATGGRNEPLEFIQALLDKPDNEVPHVLSVSYGDDELVPYAERVCGMLGLLTKRGTSIIHSTGDGGSACLTKDGTMSTFPASCPWVTAVGAPSGSSGGFSQYFERPAWQAVDKYVYYNASMRAVPDISAVGFRVIVGALEGTSASAPVFAAMISLVNDARLRKGKPSLGWLNEILYLQDITQGSCGWPAKQGWDAITGLGVPFAKF

>KFG81454

FLLFYGWPVHIMTIDKLHGFKGSGIQIAVVDTGDYTNPALGGCFGGCRVALGDNFDPMDCHGTAVAGIVAGVAPNATLAGYRVLNCDDLVGWVKAYEDGAQIILSSAGAVVVSRIVVPCIVGLGNRDSGFSTLNPSSGRGVTSVNSFARARFSTFGPNLEIKPTVGAPGDVPGIGTSYAGPAGMLALAVPVAQQGGGLARAWDATLVENDTITNAKVNVTYHLDTLAAVLGPNLPVWSGWIPYLGGRLPVGEYKLAVRALRLFGDW

>KFG81532

LIIFRGWPVLAMTIDKLHGFTGNGIRAAIVDTGNYTHEAFGACVGNCRIVTGDNFKPMDCHGTALAGILGGVAPNATLMAYRILDCDSAMGWEKAALDGAQIIVSAFGAMMVSRIAIICVGPAGNPENGFNSVSPSAGRGVISVGSFSRQDQSSYGPTLDIKPSLGAPGAVPTPGTSIASPAGVAAVAKPVAQQGGGLISAWDATLVQNDTITNAQSEVTYRLSPLHATLQPGLPIWSGWIPFLGGEIPPSRYRVALSTLAPFGDW

>KFG81969

IHESGRWVRIALQKNLDKGMDYLSDPSSAKYGQHYVVELFAPDESSINAVRSWLGWVDFVGQLESILKTKYHMYGTDEYSLPNEISYLVDFITPAVVMTPACIKSQYNRMGIFEIFEDAFSQEDLDSFYPKVDLIGAAPVESDLDFEIAIPIIYPELYEAANDDDPVDGNTPNEMCGTFKAANVISFSYGTAEAYPYLQRQCDEFMKLGLQGTSIVLSSGDDGVACLGPKGFTPGQQASCPYVTSVGSEIASSGGFSNIWSTPDYQAVSSYFIYNRVGRGYPDVAALGAVVVVNGSGGTSMSAPLVGAILTRINEERIKAGKKSVGFANPALYFTDVVRGACGFSAVEGWDPVTGLGTPYPAM

>KFG82316

YIVFTGWPVHHWTVDKLHGMRGKGVKVAVVDTGDYSHLALGGCFGGCKVAGGYDLVPDPMDYHGTHVAGIIAGVAPGAELLIFKVFSDVLIAFCDAYTAGADVITASVNALVASRIVVFVSIAAGNGTRGFYSGVGSNGRHVVSVAAAYFTSWGPTLIMKPDIGAPGYILSTYLGSSMAAPAGIAALGRNAPPFQVGTGLVDAWKVTQVSLDTITNANQTVKYTFEHESLTLHSGLPLYSGKIPYGGGKLSPGNYT

>KFG82765

FIFFRGWPVHFMTIDKLHRYTGKGVHVAVIDTGDYKHPSLGGCFGGCLVTKGFDLVPDPMDCHGSHVAGIIAGGAPGVTLGAYRVFGCDVIIAINRAYLDDADIITMSIGAVTASRIVVIVTISAGNGSQGFFASSGSSGEGVAAIASYDNDLCVLIRGASAFSSWGPTMDVKPQFGAPGGILSTYPGTSMACPAAIYALSKPAPVAQQGAGLIQAHDATLLENETLTNGDSIVTYEISYVPTTLAKGLPVWSGYVPYQGGRLREGTYKIIVRMLRLYGAW

>KFG84385

LEPDGWFSIALQPEMHGLASKIDGLTRSLRTPAQDDVDHVMEWLDWIRVVGEANKLLDMQLRRYRAPEYNIPDSLDTAIDFIHPIANFTPDCINKLYIRFGIAGFLEQWANYDDTRRSFFTVELIGGNQQEANLDIQFGMAVGYPVYYSTGGRNEPLDFFHHLSSKKNEELPHVLSISYADDELVPYAIRVCNEIGMLASRGVSVLSGSGDGGAKCRSNDGTISTFPASCPWVTSVGANGSSSGGFSAYFKRPDWQAVSEYVYYNSSMRAVPDISAIGFQTVINGLDGTSASTPVLAGMIALVNDARARQGKPVLGWLNKRLYLQDIKAGSCGWPATEGYDAITGLGVPFNKF

>KFG84515

VHEHQQWVRIALQSNLEKAEDYLSDPASPKYGQHFIVDLFAPSEQTINQVKTWLGWINFASELESILKTKFYLYGTESYSLPHDVSSLVDFVMPGISFTPRCISALYNTLGIFETEGDVYSQEDLNQFYPIIHLIGANPPESDLDFEIAIPIIYPSLYQIQGNEGSGKECNDLTPPNVLSVSWGDSEDQPFAQRQCTEWMKYGLQGTSVFVASGDYGVACLGPKQFVPDGLCSCPYITAVGSEVASSGGFSNIFATPEWQAVSEYLIYNRGGRGYPDISAIGGVVVVGGIGGTSMSAPLVAAIFNRINEERLNIGKSPVGFINPALYFNDITKGGCGFSAASGWDPVTGLGTPYTQL

>KFG87255

YIVFHGWPVHLMTVDKLHGFSGKGIKIAVVDTGDYTHPALGGCFGGCRVAFGDNFKPMDCHGTQVAGVLAGAAPNATLMAYRVLDCDDMMGWLKAYEDRAQIIVSSAGAMVASRIAVTCIGGLGNQEQGFYAMAPATGDGVISVNSVASPYLSAYGPTLRIKPNVMAPGQIWVTGTSYATPGGIAALAKPVAQQGGGLIDAWEATLVENDTITNAKSEVSYELSNLAATLGPGLPLWSGWVPYLGGAYPPGRYKIIARALAVFGDW

>KLLA0D00979g.t1_gene_KLLA0D00979g_Klac_NRRL_Y1140_chrD:86703..88388

MHPKSFIFFAISCIGVVTSIGLDDNILEDFSTVKGNGNYIVQLHPNTTIKSFVPRFLDGAITVFSQSMSRDKLEVKKNKIVRRDTQEDQVHVFDAYDIGNAFKGITVKFEDLNIVKDLALLFSHEILKIIPDQDVQFELPTPKNKKNSINKRFNARARKASSQKRIVRRGNIDTFEDDQIQAASVKSKKKTTTKKKTLKKTSKKHTTTKKRATSTKKKTSTKKKHTTSAKKKHTTTTKKKHTTTTKKSTKKSTTKKTTKKVSHTTTKKGSKTKTTTSKKTPVPTKATVSSSDYVKQDKAEWNLVRISEHKRNLSQPYIYDSDAGSDAYVYVIDDGMNIDHEEFEGRAKWGWSAFNGTSRLGEGHGTHVGGIIGGKTYGVAKKTNLIAVQVLNEEGRGSISSLLSGLQWVTTNAKKYKGRSIINMSLGMKTGGVSSSALDALNQAVDAVVASGVPMFAAAGNWGDVDACDVSPASNKNVYTVGATNRNDEMTDYSSYGKCVQILAPGDDILSSYIDSKTSTVRMTGTSMASPHVAGVAALLISEIDNPTPQNVYKKMTSLSTAGLVKTISHNTVNKLLFNGQKLSNA

>KLLA0D19811g.t1

KDHQYSYGNLLAEHDVRGLANHYVFSKRISDPLFDQQWHLINPNYPGNDVNVTGLWKENITGYGVVAALVDDGLDYENEDLKDNFCVEGSWDFNDNNPLPKPRLKDDYHGTRCAGEIAAFNDICGVGVAYNSKVSGIRILSGQITAEDEAASLIYGLDVNDIYSCSWGPSDDGKTMQAPDTLVKKAIIKGVTEGRDAKGALYVFASGNGGMFGDSCNFDGYTNSIFSITVGAIDWKGLHPPYSESCSAVMVVTYSSGSGIKTTDLCSNTHGGTSAAAPLAAGIYTLVLEANPNLTWRDVQYLSILSSEEDGKWQDTAMGYSHTYGFGKLDAYNIVHMAKWINVNPQGWLYLSTVSVSAEEFKQNNKRLEHVTVTVDIDAPYRGHVLVDLISPDGTSTLATARRLDKNRYGFQNWTFMSVAHWGSSGVGSWKLKVSWRLKMFGETIDAKKARRIYEFEVND

>KLLA0F06028g.t1_gene_KLLA0F06028g_Klac_NRRL_Y1140_chrF:576687..578213

MLPKTFILFAVSCVTIVTAAGLDDNILEDFSAVKGNGNYIFHLSPETDIKTFVPDFINGATHAVIQSMGGDKVQVQKNKIVKLGTDEEQVQVIDVYSIADSFKGLTVKFENASVIEDLTKEFSKNILRVFPDQDIKFDLPKPKNKKRFLARSRSQKRAERKGNYDPDTFDNFGKPDDLELENSTLSKRAATSYSRQSGAQWNLVRISQHRRDLSQPYIYDSNAGSGAYVYVVDDGMRTDHSDFGGRAQWGWSSYSGISQYGTGHGTHVGGIIAGSTYGVAKKANLVAVQVLDDTGSGSVSSIISGLQWVVNNAKKGRSVVNMSLGMKTNGVPSSTLSAFNSAIDAVVSSGIPVFVAAGNSGSDACYVSPANNKNVFTVGATDKSDAMTDYSCYGSCVQILAPGDNILSTYVNSRTSTTTMSGTSMASPHAAGVGALLLSQAAGTSPSSIYSQMSSLATSNAVTSITGRTVNKLLHNGQQSTSA

>lbic_185466

MDDFIAHSSDPDHVRYAQHLVADFMTPHSSSVEAVESWLEWVTLVAEAERMLGAKYHIFRTLAYSLPRELHRHIDVVAPTTYFTPACLRALYNKIGIAGYLNEFASYSDLGTFFFVTVQVNGDDQEANLDIQYTTAMTFPTYYSTGGSNEPLDWLDFILTQDTIPQVISTSYGDNEQVPYAETVCKRLAILGARGTTVLFSSGDYGVGCKTNDGFQPSFPASCPFVTTVGGEVASGGGFSRYFPQPSYQAVDGYLLFNRSGRAYPDVAAQAFQVVVGGIGGTSASCPTVASVFTLLNDYRLSLGKRSLGFINPLLYFNDIVAGGCGFSAGKGWDPVTGLGTPFLKL

>lbic_191088

DLAPKGWLRIGVQAGMEDLIANLSDPGHGRYAQHLVEAFAKPHPDSTEAVSSWLDWVTVVAQAERMLGTKYNVYRTMGYSLPRELHSHIDVVAPTTYFTPTCLRDLYNKLGVAGYLGEFASTSDLQTFFFSIEQVGGNDQEANLDIQYTIGMSFPIYYSTGGSNEPLDWLNFILAQKTIPQVITTSYGDDEQVPYAVKVCNMFAQLGSLGTTVFFSSGDFGVGCKTNDGFQPAFPASCPFVTAVGGEVASGGGFSQYFTQPSYQAVSAFLMFNQTGRAYPDLAAQGFQVVVSGVGGTSASSPTVAGVFSLLNDFRLSKGKTSLGFINPLIYFNDITSGGCGFTAVKGWDPVTGLGTPFGKL

>lbic_232257

FIVFVGRPVHILTVDKLHGITGKGIKIGIIDTGDYTHPLLGGNFGGNKVIGGYDFVPDPLDQCHGTHVAGIIGGVAYDSSLSAYRVFGCDIIVALLRGVKDGQDILTLSLGSVVASRIAKVVTIAAGNGASGWYSSSPGNGVDVISVSSVDNDACVIVRGASSFTSYGPSFYFKPALAAPGGILSTLPGTSMATPAGSAALAKRQTVSQQGAGLINVFDATVVENDTVKNGNASKKYKLTHFPALRPGYPLYSGFITYIGPSGSYRVLIRALRVTGDY

>lbic_248220

LHEPSGWLRFGLQSSINKLEDMLSHPDSSNYGNHWVTQTFAPSQETVEIVRGWLNWLEVVEEAEHLLKTEYHVYACSEYHLPIYVSPHVDLVNPSIHFTPICLRALYNTFGIVEYTPQAYLQSDLDLFFPEFVSIGGTMQESNLDLQYAMNLVNPVLYQVGPQDDPQDEIFPAANCGTTKPANVISTSYGYNEALSYTLRQCAEYAKLGLMGVTVLFSSGDNGVACLNVDGFNPGFPSTCPFVTSVGAEVASGGGFSNYFGIPDYQAVGKYLIWNSTGRGYPDISANGYVVAVDGVFGTSASTPVVGSILAMVNDARLTIGKKPIGFINPAIYFHDITSGGCGFSAVPGWDPVTGLGTPFAKL

>lbic_248825

RTYDYASARALGVEQAGELRDHWLVRTRIRDPLFSQQWHIVNEDDPEHMMNVTGVWEMGLTGKGVLSSLIDDGLDYTHDDLAANFDAANSYDFNDHEALPTPKTDRDHHGTRCAGQIAAINDVCGVGIAYDSKVAGLRILSAPISDVDEAAALNYGYQDVSIYSCSWGPRDNGEKMQGPGYLVKKAVVNGINNGRQGKGSIFVFASGNGGGYGDQCNFDGYTNSIYSVTVSSVDHKGLHPYYSEACAANMIVAYSSGDGIVTTDRCATNHGGTSAAAPNAVGVFALALEARPDLTWRDIQYLCVETAQMDPDWERMASGYSYKYGFGVLDAYRYVTVAKWKLVKPQAWLATSAIKITKDMMVEHNETLEHITVKVWIDHTRRGDVEVEIVSPRGRSILAGSRERDDDKTGFPGWKFMSVKHWGENPVGEWTIKVSDGWNMVLWGTTIDPSKAKRALYDED

>lbic_291381_gene_estExlbic_fgenesh2_pm.C_580005_lbic_scaffold_58:complement(join(181734..182164182216..182350182402..182912182970..183389))

MSVSCISVVTAAGLTDNILEDFSAVKGNGNYIIHLRPETNIKKFVPDFLNGATDIVGQSMGRKNLAVKKNKILRRDTEEDQVHIYDTYSIGNSFKGLTVKFENISIIQDIAKEFSKNILRIIPDQDIHFDLPTLKNRKRFYARSRSEKRAERNGNFDPNDFDLDNLDSEHVESSNKTLFERAATSYTKQTNAQWNLVRISGRKRNLAQPYIYDSNAGSGAYVYVVDDGMRVDHTDFNGRAQWGWSAYTGISRYGTGHGTHVGGIIGGTTYGVAKKANLVAVQVLDDTGYGAISSILSGLQWVVSNSKKGRSVVNMSLGMKTAGIPSSTLSALDEAIDAVVASGIPVFVAAGNWGNVDACNVSPANNKNVFTVAATDKNDRMASFSSYGSCVQILAPGDNILSSYIDSKTSTTSMSGTSMASPHAAGVAALLVAQSAGSSPSAIYKQMSSLATSGSISYINGNTVNKLLFNGQQSTNA

>lbic_299797_gene_eu2.Lbscf0017g02510_lbic_scaffold_17:join(741526..741688741756..741858741916..742052742112..742117742180..742196742250..742289742357..742367742419..742544742603..742643742705..742725)

MLPKTFIFFAVSCISVVTAAGLTDNILEDFSTVKGNGNYIIHLRPETNIKKFVPDFLNGATDIVGQSMGHKNLAVKKNKIVRRDTEEDQVHIYDTYSIGNSFKGLTVKFENISIVQDIAKEFSENILRIIPDQDIHFDLPTPKNRKRFYARSRSAKRAERNGNFDPDNFDLDKADSEHVESSNKTLFERAATSYTKQKNAQWNLVRISEHKRNLAQPYIYDSNAGSGAYVYVVDDGMRVDHTDFNGRAQWGWSAYTGVPRYGTGHGTHVGGIIGGTTYGVAKKANLVAVQVLDDTGYGATSSILSGLQWVVSNAKKGRSIVNMSLSMKTAGIPSSTLSALDEAIDAVVASGIPVFVSAGNWGNVDACDVSPANNKNVFTVAATDKNDRMTSFSSYGSCVQILAPGDNVLSAYIDSKTSTTSMSGTSMASPHAAGVAALLVAQSAVSSPSTIYKQISSLATSRSITSITGNTVNKLLFNGQQSTSTQV

>lbic_322240

RTYNYVSAQALGVEQAGELQHHWIVRKRIQDPMFSRQWHLVNDDFPEHMMNVTPVWDMGFTGKGVIASLVDDGLDYESEDLAANFDADDSYDFNDHEALPTPKNFDDHHGTRCAGQVAAGNNVCGIGIAYESKVAGVRILSGPITDIDEAAALNYGFQNVSIYSCSWGPPDNGRSMEGPGYLINKAVVNGINNGRGGKGSIFVFASGNGAAHGDQCNFDGYTNSIYSVTVSAVDYKGLHPYYSEPCAANMIVAYSSGGGIVTTDKCATTHGGTSAAAPNAVGVFALALQARPDLTWRDVQHLCVETARMDPDWERTAAGYSYKYGFGVLDASRYVRAAQWKLVKPQSWFLSSTLTVSTQMLIDNNESLEHINIRVWISHSKRGDVEVEVVSPHGKSVLASTRQGDQADTGYPGWTFMSVKHWGEDSVGDWIIKVSDGWNMIFWGSTIDPSKARMVKLYDAF

>LELG_00316.t1

RDYNYSQIDFISEHQVNSMDNFYVFSKRIHDPEFSKQWHLLNLQYPGHDINVTGLWLDGVFGEGITTAIIDDGLDAESEDLKDNFNAKGSWDFNDNGNIPLPRLYDDYHGTRCAGEIAAVNDVCGVGVAYKSKVAGIRILSGGITAAEEAAAMVFGLDTNDIYSCSWGPTDNGKTLSEPENIVKQAMIRGIQEGRDNKGAIYVFASGNGGRYSDSCNFDGYTNSIYSITIGAIDYKGEHPIYSEACSAVMVVTYSSGSGIHTTDICSALHGGTSAAAPIASGIYSLILGANPNLTWRDLQYINVLSATPDGNYQKTALGYSHRYGYGKTDAYKMVEFAKWKNVKPQSWYYSSTVTVTKDDLEVMNEKVEHITVKVNINSSFRGKVGVRLVSPLGVSDLATFRPGDMSSRGFQDWTFMSVAHWGEDGLGEWQIEVNWQFRIFGVSIDADKARRFEFLFEQF

>LELG_02417.t1_gene_LELG_02417_Lelo_NRRL_YB4239_supercont1.31.3:293455..294885

MKISLTLFVVAIFAVVPLGCCAPLERGNKSETGPKGRTTEPATAQKDVPEPEPSEKYLPYLVLFKDSENRPWETILANMGFGAAVKESKHPKRGPLASRALDQGDSQYFTTPNGRQLHAFGGGEGTMRVLKMNMTLPEVERVKLLEYVEFVEQNKRIPVFEPPPEDNPGVKPSLGLIRPATVPYINAPGFECRRRGFDGRDLPIIEQCGAPWGLYRISSYEPPVFTSSASEQASLSYTYRFREGAGIGVDIYILDTSLNENHPDFEGRARLLWIPTDVDDNPDMALHGTHVAGIAASRTFGVAKGAMIYGVAVIVGDDILDEDIYGGGNAILRHHRGRRDSPGFAGSIVNISAGVDEGWPAIEIMVGRFLREGIHVTVSAGNRNKDACGASPAGMSRIFPVITTGAIAFDDQRHPRTSWGRCVDIYAPGTNIASLSSTSNTVPAVLSGTSMAVPHVAGVMAAELTFRPEFKFDPSGLKRFILDRAIKGGVKLKRSKEDEKVVGGRLLLNNASPGPPVQISPARSFLPDGGF

>LELG_04877.t1_gene_LELG_04877_Lelo_NRRL_YB4239_supercont1.71.7:698769..699986

MKSFFTVAASLLALASTTLAVAPRQDGFISKYALPANHSTELKEYIITFQRNETIPPKVDTYLDMLELKRNAPDGGDIILYESNNTSPDATKVIILRMCDEHADIMREFSEINVVEPKAEIQSYETRNGAPWGLQRISSEAGASGSPQGQDFTYTFDDPSLGKGVDIYVVDTGVRDTHAVFTGRASQGFSATGSPVDGDGHGTHCAGTAGGAKFGVAQGANIIAVKVLSDDGSGSSSDTIKGMDWVISRHEQRKTEPGFVGSIMSMSWGLQGTANSVDEVIAGASAAGIHVSVAAGNDGIDACGSTPAHLGGANSNVVTVGSVNIRNTVSSFSNTGKCVDIYGPGEQILSAWATGDTIINFLSGTSMACPHTTGVMAYLMAQDAAALGQNPAALKKKLLETARQSKITGNLGGSANLLLSNGVNGTPATKRLMKNYVVPDRSGSPAARAAASMSGRDIVIDERFQLHSRDSKLRF*

>LELG_05185.t1_gene_LELG_05185_Lelo_NRRL_YB4239_supercont1.81.8:complement(461250..462587)

MKSTFSVAAGLLGLCSSVLAATNEGFINKFALPANHSSELKEYIVTFWRNETIPRPIDDYLDALDLKKTNDIKIFESSPGVDPHVLIVRMCDEHAQIFKDLTEVNIVEEKAIVKSFATQQGAPWGLQRLSSATGATGSPKTEDFTYSFDDSSLGAGVDIYVVDTGVRTTHAVFTGRAKQGFTATGSFTDGDGHGTHTASTAAGAKFGVAQGANVIEVKVLGDDGSGASSDTIRGMDWVITQHNTRKTQPGFVGSIMSMSWGLQGTAASVDEVIAGASEAGIHISVAAGNDGADACGSTPAHLGGANSNVVTVGSVNIDNKVSSFSNIGKCVDIYAPGEQILSAWNTGDAVINFLSGTSMACPHTTGVMAYLMTQDVAGLGQNPKALKAKLLETARQGVVSGNMGGSANLLLSNGVNGAVAQRLVKNYVVPDNSDLSNSGLSGSGAARAAAVMASKVTLDKRFTFHSRETTLRF*

>MAPG_00124T0_|_MAPG_00124_|_Magnaporthe_poae_ATCC_64411_hypothetical_protein_(963_aa)

MPLLSLTASFFAQTAPAPDSEELSLLLKAVLPTFIAAVDRRRDVLLEVDSPEAPLQSTQSSDKLLGDFYLRLYVGLVNLQAVLVQWSPEDMIQSSSAEEDQMKRLLGAVLSCLEERFLSRKEKNAGPAFKRLGIEQTYGTTLWKLRALRKSKRMPARDQEMPESYLRDIDLVVRLESKKAAKRDELLGAVEAAFEFFAGIQLFAGEPTSSCPLQYLSYPMKHVSKRTKALFVVVQSKWSCQCDGSPSHVSRKARLNLTQHQRFDTAPVRGARLPDDVHWYRIFFPTTAHEIEWQDTDIAVDSRERLDTAHQKIGGDLCGVISNVKPKIRPCMAVWAKELWRLQPGIEERRSVLTQIKEADFVSLAELLLQQSSHGGGRSSKSSPIGLAPWRPKDRLILSFILATTLLHLSKINVPGLKLDLTSDSICFLRPPRRALPDITRPYLTVSCAPRSSTPQQQSSSTWNQPHRFPNILALGILLLEIERGVPIEPDASQDLCVEAMTELDHWTQSSSLLEKRTVPEGLRRAIAACIQPKQFKTHNLDKNNIRDDDVRRYIFDRILYPLEEALFTAYEIRPHMLLVDPEPQNKAVSGPWGSFDGEEDGEVDEYHRAAAKEWMMNLEGVHDLVYACQDRCEELAQRGGSDPRAERVKIAVLDTGLQLPTHLHTAYEGEGRILIDESKSFVGNTDSAASQQNRGWDRDCDGHGSRVGEIILRCASCADLNVAKVFQTRRDLTSPDLADQAHKRIIEAIALATNVWKVDMIVMCFGFDEPIRPIRNAIDRATKASKPPLFFAATRNNGAHREVAWPASEPSVIGISSTTAEGEASPFNSSRRDDALPILYAFGQGVPVNVAAPHDPEHFESRHFSGTSYATPIAASLAANLLGSVRMLLKASSPEDRARFAHVPGDLQEWRGMLRVLLNRMQREHVSGQKSLLPWDFLRLDMMKGNKLLREVDAAMNGKLS*

>MAPG_00951T0_|_MAPG_00951_|_Magnaporthe_poae_ATCC_64411_hypothetical_protein_(727_aa)

MLALLVSVDAHECRPGPALQRAPSLKIGVSPTELARPFDLRRIVRELIKARPRVLVDFEDDSGQTPFQARLASLQRNPDIEDVDARQDRKREIQRNKLIDKDEILSYMREYIGTFSRADAVKALYKVGGERVHEFDLSGLPRPIIDPDYLKGLQKVLWFEGLLKYVALPRLVFEADEGQGVMESNADGSAGQGQGNKILTVYASHLQLAEGSRCQLGLEGDRHRRRGAISFDQVIEECLNGLNVRIWNWFKADLCSDVILKSAKNARDLTLYSPGNNAVLKGWSSPEGLAKLEGLERVHIRVQEGLESPKRLEANVTAFKDALGKLRPDVKCTWEPHRPDSNYNAVFTNRNGENQPQVRPIPSVSKWAKAMGDFAEFLGSARPDPSVRPIKIAVIDDGIDMTLAHFANKIQVGESFYSLTGEMSGRRGAYFVPSGPHGTLMAKAICSVCPKVMFYIAQLEILPGPHGQRSFTAESAIEAIKWAVVQGVDIISMGWSINTSVKIPALHEALQAVEHARIIMFCASIDEGAKASDNAYPARASTSCMKIGACTVDGDKLSWVSEENSKFLLPGDAPISAAGDKDQWSPHRHLLAGSSVATARAAGLAAALLYCDRLIGAPRAKIFLPQSTNFTFQMGPGRGIRNHKEVDLLRSNDKITETFSGLSSGSNHKFPQLWQHLPDIQNLEWDSRAHPTDTNETKPTLDAFMRHLRTLRVGGGEELVQSSSTA*

>MAPG_01098T0_|_MAPG_01098_|_Magnaporthe_poae_ATCC_64411_hypothetical_protein_(907_aa)

MRYIVECREWRLLQALGALVGQRKSRFVPDSIKANSLSLPDGPSLAGGLLEFELRRIFLLNDVSRSLSDVEFEECDMILRTLCNLLDKLVDPRLARGSGSDYPCLRAVVGLLEDSDETYDLASSSNPSLFKPPDDEAELERALAVVTEQNDLLSRILSPSTREPAIRPARRDLKRRRKTWKDSELRDRATATLRALFDHLRCGGNHEVILKLSDSDAGTISPELNLMLSACPDRCGWLEVQSGSTELSWSASIAALQNICADLSLNMGRGKGLVVHIERYGLFGAWTTSQTGSPAPPKETLDQLISNGAFKPLSLGALASGSFSMRYKPQEKRALAVRLGYCLMDFFDSDLSSKRIYFLGTSSQSSRNGTLYLSFTSGSPAPEEPHIFRVGHPALLSFAKLLLEIDFGETIPLEISQHYDKTNQATWAELCDMVDQLEEERHDSYIQAIRGCLIVHSQISKALRLTCADRKAAELTIRKELYREIVCKLEDGLEESMPRSQRKRQRSESPEPTADRKSNARTITGERDSAFSGRPFVLQDPSPGLPARPPSIAWDRPSSACLVNPGPIPQNQRSTTPLPGSSGLFDDCTPDAYPADVCAYADNFISTHRGIYDELIQTTATIPRVKVAVLDTGLDVDHPSILANKERIRDVKSWLPASRVTNGDDICGHGTHVTGLLLDMAPDCDVYVAQIADNEPLSPHLIAKAIDHAVTAWQVDIVSMSFGFLDEKEQGCGELREAVLRAHASGVLMFAAASNVGAYGMAPAFPARLSNVFCIYSGDGMGNCSRTSPTARRHGFNFLTLGEGVESAWPRLLSQNPWTKRKSGTSFATPIAAGLAAALLLYAHQNLPPEDAKKFKEYDKMRDWLFHASNERNGYDALSLSNFFNRAPEERRLLLNSILEGRPWRT*

>MAPG_02154T0_|_MAPG_02154_|_Magnaporthe_poae_ATCC_64411_hypothetical_protein_(323_aa)

MLDRVYHNPRVTWDRAKHKKVAVAILDSGLELSDDQQAKYFNECKITYKNWVDGVDVKTSWKNGRDNTGHGTHLSTLLEKVAPEADIYVARVFGGRQPEMDKDLPNIAQAIRHAVNSWKVDIIVMSLGWDVTPRQHENEDLCAIRAIRHAADKGVAIFAAASNDGKNKLRGVAWPARMEEVICVHSFDGHGNPSKFTPSPDENNTLAVLGEGIVAAWPKRLRAQGERSRSGTSFAALIAAAMAAVVLDYSRRFLDNEEWEELRRVGGLRRMFSQLKNHKTSGYTYVLPWETFNADRSEEWIQEEIRTAVGLRRVPKEPEKSR*

>MAPG_02596T0_|_MAPG_02596_|_Magnaporthe_poae_ATCC_64411_hypothetical_protein_(894_aa)

MSPATASHSSTNTEFPEWIKLVTLRSFRGRLINNTKYYPLEPLASSDEHPDEDETQIPLELLEVVLRGLFGKNEACFLDRADVVLAAESRDHLRSLCGLLNGLVNVALLEEQDQEHSWQGAEVTTREYHKLDALLSVLLASEWPFNLATGGGGRTLFYAPSSKEANEAKKIVEEVDNWFRNQFRPRLTDISPRPITSPLESDPEMHATEGTCAQSSTGRALDALLRLVHEKKSCGKEDHQVLVQLIDSGAFPDHPPEPGVNLFLSTCHSPGTAIETRHLWQEAQLLVREATEVGNPSDWKKTTAICDELKLPHGGKALCLKIHDGAVSVFDPPDTSTRRRARFRSRWPTTTLCDKIGKDLAFAAEIYGDTRPKEKLFGLEEKRSLAARLVLSMGQSLDSGLAVMAWDSTQVFFLSEDNHQTWHLGMRDYLAAVEDCYDYGLELRRMAARSRGHAGHLYTQIVRRIRQAPVLRRAASGPGLAESQAKYQAFDQPWSLPTSGNYETMFDHPPSTPTHVEAHRVPPQQYPRRVLYDSQDSNEDPTASRKWIDDFINFRKSIIPTFDPDTTKRVRITVIDTGIDDTQPYIRRGGWRSHRQEPSKTPLPLFKDFTATNSTDECNPVDDDGHGTFIAGLILRLAPDVELSVARVIRDRQTMKEDTEIAWKIAKAIDHAVNTWEAEIISISFGLDGKSKPVHTAINLALYKNVIVLGAAGNFGNRRDVGYPASAERVFKIFATSHLDWGYDTNPPASSDSGYCFGILGLNVESTWPLKLRDKAKQVRNRSAEDPELWAVMSGTSFATPIAASLVAIAYQFYNENKRKIALRAHSEGFKSIAVVKAVLKAMSRPAGKGDKFNLLSPELGRDNVFRYDHPRGQDKISFYAKKLEDVIWASGQ*

>MAPG_02988T0_|_MAPG_02988_|_Magnaporthe_poae_ATCC_64411_hypothetical_protein_(861_aa)

MADGVWFGQLKKLATEISDTIQIASGEKRIRPYHSFYALAHDIMVGLRLVNQYLDTMPVYWIQPDIEDLCHALLRELRGMTIPPENHWSLLRIGRPEPSRLEASLLAFFKGQKAESQEIKRICDDLRVRLTREDDDDAARLPTSALQRLDVIDMSTYFNEGAFDTLQVVCQCLPQLDGKPGDPAELKVWHRARLCLNEADGDIRVLVSGPKDGSWQEFCVKTQPETSVDDDHQLLVHSWLCDLLNRELTNRLFLKFVRDRGFELLPRSDPVQQILEAGLDVSLAEVLRNYELNPKDKVLLAYAVARAYWQFYDSALMRTRWTSDALLFMPESGSEAFRGQLPLCAYLSFPFGNPGNFSEDISPDYFLTHRCPRIFDIAVLLIEIGLGKPFRIPRKKDDIVGQRNTHHKIAKDKLAELKMETWDGFSNKKFFDKAVEFCLDHKNFIKLPKNESRKLGAARAAQPSTREAQGEGTLTRRKIFYNNVVLPLGWLATKGYKAKSGDITYVSKKRELEQGVAGVGRQPEAMFHSEIVPKKWLEDIKRISESVELMRRDKKVKTPIRVAILDTGLNLNSKLPGFKSAEQLTRGTHWMDFVDPEATELTDVNGHGTLMAHVVMQCSPGAEILVARVAENTKKLKDSLENIRRAILWAGEPGRADIIFMSFGISRKDGGAIEQAIEAVVRGRKEDIVFLASAGNSDVDDENFPACHPSVIAVYATDCHGERLPASPMIPEQKAWVLGTYGDPPASLRSSFANLYPGVCEAGSSVATAVMAGISATMLAYAAVLPSLVDMPCSHVLNRLRTTKGMEALLCRLAPEPNQTLRAVKPPVFWKNKPDNGMRMCVLIDSQSEVERLYPRRSGV*

>MAPG_03201T0

YIAFKGWSVHVQTVDRLLGFTGKGLRIGIVDTGDYTHTALGGCFGGCLVEYGADLVPDPMDSCHGTHVAGIIAGAAPGAKLGIYRVFGCDVILATLRAFEDGSDIITGSVGSLVVSRIVVPCTFAMGNGAEGFGVSAPASGPGVMGITSFENDSSTFVLWGSTLTMKPQFGAPGGIISTLPGTSMATPASIVALAKPSPVPKQGAGIMQAYDAAVLSMDKVTNGSSAVTYNLSHVAATVEPNLPFYSGWVPYFGGRLPAGRYSLVTLALRINGDY

>MAPG_03852T0

YILYLGTPVLRMAIDKVHGIKGKGIKVGIIDTGDHRHPALGAGFGGKKIAGGYAWVVQPLTTCHGTHVSGIVGGVAPEAEIFMYRIFDCDYIIAMLRGLQDKVDILSLSLAADIIKKVTVAVIVAIANAGPSYTQEFPSTEPTAIAVGSVANASWFEVSSDYYSSFGPVYDLKPQISAPGGILSTFPGTSMATPAGCYALAKPQQGAGLVDVHAATRLSADNLGNGKSPKTYKLSHRGASVAPGLPVFGGYVPYSGGLEPGDYRWLLSVLRWGGDW

>MAPG_04316T0_|_MAPG_04316_|_Magnaporthe_poae_ATCC_64411_hypothetical_protein_(1001_aa)

MSTKRLSELREWKFLQEMHPMLLWAIPNLISHLEDSVNDDVDSQVTNMKLRSRREFWIYEVKKTALSAIHTQELVEKDLQRCRSQLQHVLSAIEEMLKESICCEDALNDAADYQVASSERQYSAPMISKYGSIYLLAAVHESCIPDEEKTRILTTAVQLQISLVIPGFKPLEALTSCNMCLESIFRPCRDLKDPWTQTTPTEVESMGLDISIAPFAEAALQNLHYHILTCKNPPGHRILLQLSGFQTEAAFQNAPMLALLLSTCQLPQSWQETYIKKSPYGARKPHSLSAPSYVKQLCERIATARTRGEAMQLLVEGGFRILEESVQGDKALLLPSNEPNISLVDALRRGLFKRLSQQGLTNAVPTRDKRRLALSIARSFMYLYPSGWTSKGWSSDEILFLSSIGDVTDKDGLDCIPFVPCAFPTTRPPSPIGLHEAVDAASSSGGSLAVETDILLLGKILLEMECGNEILPERLPDASEASLFLALDRVIEELEGNTQAHYISAVEGCLELHRRLSSMSLAERTQDGQRMIYDAVVCHLEKEFLTWRRPNKKRRLPDVTDEGPAKQKPRHELSEKPKKGLSWQKHRLIDSLNAKLTRWQKLPGLSKADNPGTVNEVYLSKHCLPGGNSSCAPESAENEPRKRQMDSALVGTLYLSSGAGLGSPKLVEEEVNQITLFDDLASTENSKSAKTFLKSCAKFFEKYLDPLEDDESPLARPHDRPQGNVRVCIIDTGVQKGNPDLQGALLDGRFKEGYSWLDEEDPTNVTDTFGHGTHVAALLARVAPRADLYIAKVADSRTTQPGQVAFISNAIWKATDEWKVDIIVMSLGLSAWDNQIHKAIKHAANNSVIMLAAASNHGSNEPRTFPATHRDVICVHASDGNGGGVSFNPLPQDDDHNFSTLGASVPLIWNGFPVSKSGTSFATPVAAGFAVNALEVVTRQAIIPPERIFCGEGMRELFYLMSIKPEHSGYRYLAPWHLWRPSRQIHLTAGLIKEHFGVVR*

>MAPG_04515T0_|_MAPG_04515_|_Magnaporthe_poae_ATCC_64411_hypothetical_protein_(940_aa)

MSHLELISNVIPAFVALARDYCNLLESEEHDQDLTRREHEGDLAFDANICYQDLALIAQTIAFSLGEWRQGLDDKAIINLMKALESRLPKLPAQSLSLWLGKFHDTLKAGSPKPEPNETSVDGHLSAQFPRLTALASKLSAEGLGGYGECERLVGLSSEDIQLYRSFLSRKIRLFRESEERSQNERNKFLHRIKDARAFLVDVYCRCPSRLADEAGSHISPSSSFIRTAAETAYCLLGRNWKCSCSQRVRQAGLSLSREVRLGLSHNSQAQAESALFEVIFPLCQDDQEWKPAKVEVRTSSPRYKSGMEAECVAHDICLRLKESGKGQVHFVAGGDQLWQRSPKGSDSSQILYQHMKTLDHLLGDGTASPDIARYYPKERLLLCYALCKTCMDFYQGAWLRRLWRSDHIYLRQMAGHIFRWDLEMPFLPADLTELEPNSTPKATFYHYHKYPIILGLGIVLLEIATSTRFPTSQNPDQRKRYNEDFDKAKDLFEELKFSKRRGGAKLLMSGLIDAIRACLEIEPPSDSPTNQLAEDEQVRQYVWTRIVRPLEETLANVWQPKAIKPQAHQKEVSGRRMVTIQTTHMVQPPPPTKVGPSPSSNVTPGQNETVVSDEGERRRVDEQKVKLAETWFKYHGNALNYIKELGNGSAAENKRIKIAILDSGLKLSEDHQIRYNYTPEMKYCSWVDEPGSTEPKDGVGHGTHLAVLLRKIAQEAEVHVARVYRKRPGEKSIPLITKAIEHAADVWQVDIVVMAFGFDREFQTLKDAIRSRTDRVLFFAAASNDGKNRTDQIAWPARHPDVICVHSADGHGNPSTFSPDHKDNMRIMTLGEGIRSAWPPGLLANSNADKKGEVAMSGTSCATAVAVGIAALILGHAREFATEDEWKTLHTPDCMRRILGHKTWSKDVSGYLWVMHWNLFEEKWSREWIRDSIRSCFR*

>MAPG_04807T0

YMVFHGWPVHVMAVDRLHGFTGRGMRIAVVDTGDYTNAILGGCLGDCIVTHGWDSVPDPMDCHGTHVAGIISGVAPDARLGAYRVVDCETIAGMLRAFDDGNDILTLSISSLTASRIVVPVFVAIGNGELGFDPVAPADARLVSSVSSYDATGCVLGGSSFSSWGPTLRSASTFGGPGGIMSTYGTSMAAPAASAALAKHPPSQAGAGLIQLWDAGVLSNDSLRNGGSDAIYKLAHTATSVAAGWPIYSGYIPYIGGFLQEGRYAVLVSALRLFADW

>MAPG_05594T0_|_MAPG_05594_|_Magnaporthe_poae_ATCC_64411_hypothetical_protein_(877_aa)

MDWRPSLLSVKFALSSLEKAIPVFGGLSGEINQNVTRERRSVASRIIPALDGWLDSLPNPDDTRCSPAAVEDGLEERYGCDDDIRDSLNEFNDCLHKLWPSRCEAPHKLMLKLVPHRQYESGGQLAVDTLSSGHDQHWGRIVFTLRKRPPIVTVQESSSRRVKFADESTDDGDDFRGRAHDYTARPHDEANGVFHGSQADCPTLCSLIEKSGKERVCLRVDVESLTSSEPRGEKGIRSSKPTVALRDLLTPLKMVGCDDQTSREACLTPAQKAEVGLVLACSLLQLCRGQWGKGSREGAWLRRDWAEQGICFLPGVRNELDIGSPYLPWLLDSSAEEGGFDYHASSVVALATTLVQLQDDEVIQELDSIFQGLLDDGGMTPTTKYCALLELLNTEIFISYVDEKCQMAIKACLNGEFASSIGLEEEAATETLFRTLVVRPLSEYHHRMQQFTGRRGLAQRIQGTTRGKDKRHQSSRLPETVEAFPPSSQTATSWAYGEADDATGVAPRREIFQIQGAAFQAGQYAKPDDWLHGFKKISEDIAKIPKRCVSQPRGVRVAVLDTGVNLARQFFQSGARCKKIKQTANFVPDSRPVGAAFGAVGHDTFGHGSLMAQLLMEAAPMADVYIARVAENTHTLWESKEAIVKAIEWADDQDVDIISMSFGFADDDQAISTAIESVHTRRRGSVIFVASAGNSPYEREKFPARHRCVMAIYATDGYGTFAKSNPPLSGDPSLVFGTYGDQIPPRITAQYESDICQPGSSVATAIAAAIAAIMLAYVESLPWVGLAAADEARLEWELERRKERLQNLQKLRTREGMSSLFNRMAPQSIDQRRWICPIWFWMSRRGNAELETALLEVANGLHTPVPVRRFDGTAPL*

>MAPG_06002T0

YIVFTGWPNHKATVDRLHGILGKGVKVAIVDSGWYKHPALGGGFGGFKVAGGWDFAPDPLDTHGTHVMSAATIIAWLRAFDDGADVITTSIGAVVASRIVVVMTMSAGNGAEGFFGGEGGSSEDALAVASVQADACALVRGCSSFSSWAATLLLKPDVAAPGGIFSTWPGTSMAAPAGIAALGRSAPALQMGSGLVNASRVATLDNDTVTNGASTVAYNFTLEPASLAPGIPTYSGKVPYLGGRLAPGNYTMRVAAARPFGGW

>MAPG_06159T0_|_MAPG_06159_|_Magnaporthe_poae_ATCC_64411_cuticledegrading_protease_(383_aa)

MKFSAIVSLFAVAVAAQKSIEQIVSEHQERFRLAKRDVVPSNDSDETVSAFAASVQDRYIILFEQGASLQTREEHYNQVDTMTSAQGAISNVFGSLADIGHKITQGIEGTYAFGTAKEGSQSLYGYYGKFSADTVEKIKNTKGVKVVEKDSIETIAAPAVQFDEVPEEQTLAKRQSSPTYYLKQNATWGISRISQRNNPLGTNTEGFYYFRGATTGAAPLVYVIDTGIRTTHKQFYGRASFGANFIDDVDTDENGHGTHVAGTIGAYDYGVSMWTRLISVKIFDKNRNGSLSGFLSGLSWAIDHHLKNPSQRAIVNYSGSGAVSQARDAAVARAIAAGLPVVAAAGNAAGNACDVGPANNANVTGFISVAASDNEDAIAGFSNFGKCVNVFAPGVDILSLSFQSDTGLATMSGTSMATPHVSGIMSYWLTLNLGMTPAQMADLLTENATPIKIKGNHPDTVNLMANNLVFDSNSP*

>MAPG_06721T0_|_MAPG_06721_|_Magnaporthe_poae_ATCC_64411_hypothetical_protein_(928_aa)

MWYLVYLSLTAPAFAALAKESRDGIRDGKCETLRRDAEDCYSDIVNIGLAISRSKGEWSPGLRDDVLVRFLEALETRFPKSPAPWRQRSTRSDQDAVAFASINNFPRLRALAKDIRLCKLGGFLQCSELFTMPTEQHDALLERLRSLSRSIELFRGSEIKLSSKRQKFLRRVKEARKHLHDIYCSYGAPTGLGTAYLSLSQHPSHSVRSAANEVYGLIKNHWQCTCPQQASAASQSKEVRLGLKSRGETAPGSPLFELLLPICENRKEWKEIVVEVEDVRPGELPDGERVAVDKDICRWIKDSDCLTVKFLVERGILWHLKPDIDLERRYDEHILPLCHLLGGAEPSLNILAYTPEEQHLLCYSLADSVLWYYPGSWLRESWSSSRIFLTKAQDSDRLTLCSPYLSVRLTEYQGETGPPPHMQSHRHPVILALGIMLLEIVTGSQFPRSDSPILSRRCNDDHIAALQLLNSLERRDGAGGTRFLTSGLRHAIRSCLRLEPLPNLRQERMSEEGPIRHHILTNIVRPLALQLGFGGAEQGGKPRDDGLIQTPSTNAPATTFSTSRRPQLEPSGRRLVGTVDSQQDRIPWAWNRDMSLYGQDYGPMRTRERASADRWFEWHHGVLQRIRGLREGAPSDDKRVKIAILDSGLELSEAHKVCYDFEPRIKYRSWVSDPDEKRDEAGHGTHLSILLRKIAPEAVIHVARVFRKNPGVKDTPKRIVRAIHHAATEWKVDIVVMAFGFGEKIPDLSCEIKKASDNGILFFAAASNDGRNLPGGVAWPARDPDVVCVHSADGHGFPSRFTPRPLDNMRLMTLGEGVVSAWPPALMPKGSIPSVDESGGVAMSGTSCATVVAAGIAAVVMDYARGSLTDEQWRNIHRADCMRNLLGRMNEGPSGGRSSDYNWVMHWAWFQSRYTKAWIDNSIGAYL*

>MAPG_06934T0

YILFTGWPVHSHTVDKLHGILGKGARVAVVDTGDYDHPALGGGFGGFKIAGGYDLVPDPFDTHGTHVAGIIAGVAPEASLYIYKVFAHDTLIAFLMAFQEGADIISCSIGAVVASRLVVVVTIAAGNGYDGVFSSSGAAGKNVIAVASTDPDPCALVRGASEYTSWGTLLESKPDIAAPGAIYAPYVGTSMACPAGVAALGGAAPVPQVGGGQIDALKVTQLTNDTITNGSGPVTYKFALQPATVQPGLPIYSGKIPYTGGKLAPGNYTFRFAALLPLGNW

>MAPG_07097T0

RNYDYSPARELGEGPLGELADHHIYSKRIFDPIFLEQWHLINPIQVGHDVNVSDVWLAGITGKNSTVAIVDDGLDMDSRDLKDNYYAKGSYDFNDKHPDPKPRLSDDRHGTRCAGEVAAVNDVCGVGVAYDGKVSGIRILSKLISDADEAVALNFDYHNNHIYSCSWGPPDDGVSMDRPGILIRRAMLNAIQNGRNGKGSIYVFASGNGAMSGDNCNFDGYTNSIYSITVGAIDRTGQHPYYSEACSANLVVTYSSGSGIHTTDVCYKQHGGTSAAAPLAAGIFALVLQVRNDLTWRDMQYLALMSAIPDGEWQDTPIGFSHTYGYGKVDTWGVVELAKWKLVKPQAWYFSVKFEVTADMLKEANERLEHVTVTMNVEHTRRGDLNVDLISPSKVSHLSVTRERDNTRAGYKDWTFMSVAHWGESGVGTWTIVIKDDWHLKLWGEAKDASKVRRRLRYEFLYDAF

>MAPG_07109T0_|_MAPG_07109_|_Magnaporthe_poae_ATCC_64411_hypothetical_protein_(1109_aa)

MPFIGDTVEWDLISQLRECSQALVGHTLQARHRAVARSTRVHKLWTALRELNIHIRTTFMTLQLELLLQPDSEEEDGWYSDSESDGDDLSSEPSTASFHTAWEGQADRHGGGKAKMGYDSDDPDESFETMGRSRSLSQLQLLCSLLEARVNPLLVPLEGATAGLQPSLYPKLAHLVEYLKSSIAAAPHSRDNDLEFIPLPGSGTAPFLFNVSTPDQADTAEAFVSICESSSRWRPQKEIRKQMEADVDYMKEQILNFNIFVSTLKVSPGLLETLASRPIGSAPASTVDSFPGSSHTSDPNTADSFATSFTALQSFRQQIAAAIRAISSHFRLCEPGKHSFLLQLPSWEDISGRRHTTTMDAAQVVRFFFTGCDRGDWQYASIRMLRSGNSPRCRPGKQRLCKALTSAYNEDRDLEFYVSGAQERSDGRTEMPPIHVPLLPGRGRRYGKSAPGNNNLSTLIERDYFAKELSMDLIDHAGCSAAVFTVEQRRALAVKLVLGLMLSMNSDHSINTWEPKRIRFLEPLDAERTPFIVIDGDEPEAAERRWDCVSLADLDAVIDSGAEDDDAGDPKPLPPFTLLAKSLLHIAGQQMGSFRILRTKSGKDFHENWRRFRRTLAEYTKRVTCGRKVDLEALPFLHAAQNCLEFHTLYTAQSILAQDRPRMEIAWQLVFDDILARIDSSLRWASGYAQTVAVQGAHPPVPASQGPMGEFSLPTPLNNYYNPNSSLRFASTLPEVALFDGLDVAENSREAKNADIFFANLGDFHRSYSRFVAPRIVSTPSGEEHPRRIRIAVLDTGVDFRHPGIRAAKADGRIREEWCHSWVGPEADVADEDDEMHGSNCADLLHQVAPEADIYIGKIFQCNKLKTYQAENITKAIRHAVEIWKADIISMSFGLQRPAPSEDDDRQAELMALEHYNSLVEGIEAAIKRAPSNVTMFAAGSNGGKNASRAFPATLKPWVIAVHASDGLGGAGGLNPPLEADDNNFMTLGMGVGLMRREWDGSSGRLQPKYKAVRKCGTSFATPIAAGLAATAMDLSLRVDAITKRTREQLTQPVQMAKMLQLMSTGGTADYGGYRYVAPWHLWRPGWQADGERCRHIWNTINLKFRAH*

>MAPG_08397T0_|_MAPG_08397_|_Magnaporthe_poae_ATCC_64411_hypothetical_protein_(978_aa)

MTSMEENWLGESIEFRLVNRLCRLCYGRLAPPTYGRAKVTSRRRNGAKSFEIELRKALRRPSDLDLLLGLDPEDDDEDIKEAQELLCDLCVVFDSLSSHHPPVWDSFLNSRQGKGMKYPKFRALLVHLEGSDTGQQIEAVSGHDANPAWRLFALLSEAHMEKTSRVLKRFNVFLDQLSLSKAEVDDLAGAAVTEPPPQPPHQNPVGGGIRNRFRHAEAAIKAIFSRLSKCEHNATKAHDILIQLPAVRDIISQDNRSPDSDLELFLSACADSSQWQEAQVVHKSNRCDSTESSICDAIKEALFLSGGLEFHIQDQLTDISENDFLIPSYDYVKPRHGRLKPCKTLWDLLDSGTLSRVRLATHFLSTTEVRLISADERRELAIQLLYGLVMCLRYGCTIATWDSKRVFVLADADGRQHAVATFPHVSCVEGDPKAKWFDLPDLEVSLERAPTPKAFIKMAKALLEIGYGQCLDRLEVNASGDNQELSKNLRKMVQNMRLEVDESGSGGGDPRLKNWTNSLDVFPYIAAAESCLRFSFLYRKEVNRIGMGRERIDPWPIVENIIYNQIMCKLEKTDLMDSPIGSMESVQEARSRSQERPVAGLKTKSRAYVIRGAVVDAHPGRQNVVRLFDGSRISGDHALASQAERFFADLEAFRAAFESYVDDDVNSITPPRPVRIAILDTGIDKDNVEIDFDGEHMKDDWCVNFVGGPLNPSGAPDPFAFHDHDGHGTHCASLLRKVAPDAEIYVAKVFGKNEFDLTQARNISKAIKHSVQTWDVDIISMSFGLDSPTSEASMCEWRSIRKEIEEQITLARPRLLFAAASNDGKNRPRAFPSTHRDVFCVHASDGNGNGCGLNPHHDGADNNLMTLGTGIRLLEDDSYVYGEGTSLATAIAAGMAASIMELTSRMSRLNERTRTALRTSEGMRKLLLRRMSGPGDSGKPRPYVAPWNYWRPEYWTSNPDGLEMVWSQLNCDFIDYY*

>MAPG_09140T0

IIVFTGWPMHNYTVDKVHGVYGKGVVVAVVDTGDYTHPALGAGFGGHKVVGGYDLVPDPKDQHGTHVAGIIAGVAPEATLRAYKVFGGDVLIAFLMAYKDGVDIITASIGAVVASRIVIIVTISASNGVFGFVASSGSSGKNVIAVASSLADACALVRGASDFTSWGPLLQIKPDVTAPGGILSTYPGTSMACPAGVAALGGAAPVAQVGTGMINAAKVTRLTNDTITNGKEPVEYSFQLQPTTVAPGMPLYSGKVPYLGGALPSGKYRMRFAALRPFVSW

>MAPG_09314T0

YIVFTGWPVHVMTVNQLRGIRGKGIKIAIVDSGDWKHEALGGCFGGCLVSFGYDLVPDPMDCHGTHVAGIIAGAATDVTLGMFRVFGCDVLIAFGMAYESGADIISASLGAAVVSRIVVPCTFSAGNGPKGFFSSNPADGHGVTSIASFDNNGCVLIRGASDYTSWGPTGQMKPQFGAPGGILSTYPGTSMACPAAVYALAKPAPVAQQGGGLLQARDATLLSNDTISNADKAITYTLSNLGATIPAGLAVYSGYVPYQGGLLPPGVYKFVVRSLRIFGEY

>MAPG_10305T0

FVVFAGWPVHHSTVDKLHGILGKGVKVAVIDSGNYRHEALGGGFGGFKVSGGFDLVPDPMDVHGTHVAGIIAGVAPEAEILSFKVFGADTLVATLMAYEAGADIITASVGATVASRIVVVVTIAAGNGEEGMYASSGSSGRDVLAVAAVDTDGCVLARGASSYSSWGGTLGIKPDVAAPGSIYSTFLGTSMATPAGVAALAESSPVPQSGSGLINATRVTSLANDTITNGTEPVSYTFGLLPAVVQPGLPVYSGKVPYYGGRLAPGRYRMRFAALAPFASW

>MAPG_10648T0_|_MAPG_10648_|_Magnaporthe_poae_ATCC_64411_hypothetical_protein_(880_aa)

MDEDPCSLLAAQAASTSGVIFRAGLKPREASYHLAYDVATKLHLISGYIGSLPPYWIGPDVEQLCRALLRDLTTERLDKAPANPFLFAFPFGRNSAADRVARVATFFRTRTDEAGRLQATCDELLARLTNEDDAEGQRLPDSVLQRRDGTDDSAHIHDGIYEALEMITKCDLESHGDGIPANDRSARDLRHPAKLCLHESCQTDSYASFNVRIFVSGMNMNIWQEFCLRAHQQVQSATEDYQKPLSRGGFCRTLERDITARLFLSFKDDCGLVLLNDAEVPRQILESGNGESLASVLHNYELTPRDKVVLAYAVARAYWQYYDSNLMRTKWTSESIRFMHEKGGRGHEGQLPLCAYLSFPFGVPSNPSDDVLYEDMLSHRCPRIFDIAVLLLEIGLGKPFRSANKQDPVAQANFNHKIATDQLLELHKASWDGFTNKPYFDGAVKFCFNGENFIPTSKQPRTSRQGVVVPTPPMTASERQEGIRFRRRAFYKNVVRPLEWLAKTGFMSQTGDMTYVSKKQKPLDGLALADAARLPEPEALFHSAIVPKMWLDDLKKISRQVEQKRRLAKDPVTRRIRVAILDTGFNRGLPVFEQKTSLLGCIAEEKDFVDGAPTVKDEFGHGTFMARLVMECAPGAEILVARVARNTKELGESKDRIRQAILWAGQPGKADIISMSFGFPTEDEGIREAIEMVQRERKEKIIFLASAGNSSSDDESFPARHPSVISVYATNCHGTFLESNSESTSRGASIIGTFGDNIPDSMCDEFSTVHPGICKPGSSVATAVMAGIGATMLAYATALPSLLGLQGRPANACSRVLGRLWTAQGMEALLYRLGPENKDFRRRRAVKPMWFWKNRPDDMKRCWGICEAVSDVESRSPTS*

>MAPG_10656T0_|_MAPG_10656_|_Magnaporthe_poae_ATCC_64411_hypothetical_protein_(899_aa)

MCRPCISAGAQPCCSRLLQGLLYGRAFLPLYAEYGNPGSFSHAAFRPATRLRGTYAMANSQEWFEIPHRSCGATAAIYQVGQVLFDPFNLDSALFRNGSFNGKSSQEITETIRLGKALTVAYPRTPSSFEVNTGHATMIEGARIDGKGIPVLWQVLEEERIYRNDALLKAVIQEDEITRHLARPDSSKHLYLVTDVIKVQEIIPKTHEPGSLQPPPPLRLATPVPLPRFAAPSISLSEDLHLPVGFDGYKNGTPFIWKYKLERINNPEDVASVYAERYSPGRAISDRNEETWVSLILVFLFGWMWSPSLRNAIPNAGIGGSSALPARPLALEWRVEHEDLASSPSGDANGVFPRAYRNSIYPPQPPEPWESYRFPRPSLEHVNPVDEISASPDEGDDEIMRPPAGGTGVPRSPSPRRGRRKRVSSEGDLWGWRHSGKISRESTPGSPRGARAQSTGPETHTGLEAAETASLPDGLEKIEAAEKASPDIVITQDGTKQGQAAVNGVGSNETTAETKVGRIGERNHAELFAVPALSNPPAPNSPAAGTRWTEYDVFDSEPLASVPSQDEKAINAGKKWFNDAEYMKKVEKFIGRFRSGRPSERPKIAVLDTGLDFEHPLLKKFLDNKTLSRGRSLDFTLEPNPQVKGDDVGHGTHCTHLILKTCAMAEVYIAKVFNKKKADQYTASYIAKAIDHAVNNWKVDIVSMSFAFDNPHGEIEEAISKAQGAEKKVLFLAAASNYRDLDRLPVGFPANNSNVIAVFSHTLNRNRSEFSPMRSGRDNFSVLGEQLTAAVPPGTNQGDYERRDSGTSMATALMAGIAGLVIELSRLPLQEKVTAPDRLLTRAGMEAVFQLMVPKEELVNRDPNGYLCLRPWILFRDGTRHDPWLLFYEINSALRRVA*

>MAPG_10661T0_|_MAPG_10661_|_Magnaporthe_poae_ATCC_64411_hypothetical_protein_(524_aa)

MVPYLEAVEACYDYERLLRDMGGGRSRNAGHLYAQIVDQIRHAPVPGPTAPEAGLTRRQEKYQALNQQWSISTSDNQETMFDHPPSRPTATKTRPVPQQQRSDTRRQTKRVKFTRDDRDINDPTASGNPLSRPNRTITPHPGPKNHEKVRSPCQAGGDNNHRHSHRTIDSIQEDPSHQPSSSGSRQADDGKFLGSTVVHDPTSWLEKLDEIARVMDKLRRENSTRVTRPVRVAVLDTGLDMEADCINGLGLTRPPAWKDYVGDSSEPVDDDGEKHGTAVAALLLRTATHADVYVIRIAENQKKLSEAEAAIEKAIRHAVDVWDVDIISISFGFSRQKRRIKTAINDAVAIERNSGGGSVLFFAAANNEGLNERELFPASDPNVIAVRGTNEWGEFVRRYNPDPHDSKADLVRFGTLGENIPYDLLDRHAVKSGCSFATPIFAGLVADVLQCFDYVCEREQLRRHLRTKEGLVALLKLDCLTQIVNRTERWHYLAPWDFFLGMDDNVRLSCVHAALHGIIKHSA*

>MAPG_10820T0_|_MAPG_10820_|_Magnaporthe_poae_ATCC_64411_hypothetical_protein_(878_aa)

MDEDTYTRLRERSAEIAKAIYAAAGSQRMTPSEACYSVAQNVAARLYLINVYLGSLPPYWIEPDVGDLCHSLLKDLLGITTPPKGLSGSLLFRHLGRFSQPSSGANPPDLETSLLKFFRAQHTTAQKLMSTCDELLAKLARDDAENDAQEVPQSALKKLNCGADMSEYFNGGVFDALQLISKCNPKFHKNVVGDPWHPARLCLDETEVGIRVLVSPTTETGDWQEFCVRTQPESLGDEYLPLPEQGGFCDILEKQITARVSLSFIKGRGFTIVGEAQQPQQILPAGREESLATVLRKYDLKPKDKVLLASAVARAYWQYYDSDMMRTRWTSDTIWFIPEEKGGAQRDQLPLCAYLSFPFGSSDGSSKDIFLDLFLTHRYPRILDVGVLLLEIGLGEKCPTGNKRDLVAQLNTNHMIATKTLERLEKATWDGFLNKKVFDRAVKFCLDGKNFIKAPKKPKTSRLGPANPPKPMTVEEQKKGVIERRRIFFQHVVQPLAWLAREGFKAQPGLITYVSEKRPEPQLGPTDPAQQPDGECLFHAEIVPKMWLEDIKKISASVERKRRAQRIKDPVRVAILDTGLNTDLPIFEKKPSLKHAVKDCIDFVDGASAMTDTFGHGTLMARIVMECAPGAEILVARVARNSKELKTSRENIRKAILWAGQPGKADIIFTSFGVSREDEGGIGEAIEAVERERGEDIIFFASAGNSDTDDESFPACHASVVAVYATDKHGVPLSSNAAAPGQKACVLGTYGEPPDSLRAEFATTPYPGICEAGSSIATAAMAGIGAAMLAYAAVLPSLEERPCNHVLKRLRTSRGMEELLFRLAPEAKAHPWLRAVKPPVFWKNKPNDSIRHCVFIDALSDVERLYPRRPRGPAKKI*

>MAPG_11020T0_|_MAPG_11020_|_Magnaporthe_poae_ATCC_64411_subtilisinlike_proteinase_Spm1_(537_aa)

MSSLSPLSDAPLQHIVKLKDDIDMDAHIVTFNNARGNGHIVHRKWDPRFLNAYLGTFSEAVLEEIKGHPNAEFVTEDSEQSDEFEVTTQNDAGWGVSRINHVKSLKGKSDQLTNYDYEYDSTSEVGSGVDIYILDSGICTEHVDFGGRATWGITINGTDGVDVKGHGTHVASVNFITRSSILLTKCYQSGIAGGSRWGIAKGANLIAVKVLDDEGHGRQSYNLIGLEYVFMRRLFDGRSSIINMSYGGQQGQSEIIDFALMRITSVGIHVTVAAGNKNVDAKDTSPANCPCVNTVGATDINDDRYIKEHDDDTITGSNFGELLCGLGVELQLTGYLLYAGPCVDFFAPGFQITSCSIKKDKNGKYTKSTKKDGTSMAAPLVAGVIAAIISLVGDIPPPGMTNILKALCLRNILGRIPDDGTPNALIRGALHVPIAFSLTSDWASMAINNRNTTLSRIKANKYDENTSTTSKSVQYGIEGSRVYHDCTIPLMFVSVDLSVDISLCHGSDGKEDEDQAGMATATFASEEYVSAGISDNVRNDDDYNDCPLESS*

>MAPG_11267T0_|_MAPG_11267_|_Magnaporthe_poae_ATCC_64411_protease_(463_aa)

MSSRSSSIATKKPRRALTGLLFSTCLAARLPTIPSPDNNQVASIEVDVKGSRNTSAAQQNAPGNLVRLSHYQTENVQTYEYDSSAGQGITVYVLDGGIRLTHEEFEGRATFGAGFASHQGEGDSNGHGTHVAAIIGGAKYGVAKQVQIVSVKLQPREPQLEKALDFVLKDVQHRNITGKAIISMSMSFPASDDIDKMFRRLVNSGIVCVVSAGNNNWDASKNSPSRDPGVITVAAMNHRSDSRWEESSYGPAVDLYAPGADITSASRDSDSASVTLSGTSQAVPHVAGLAAYIMSLESITKPSQVAARLKDIAKQSGAQVQWNAPHTTGLIASNGLDKGGPSSLFPPKRIPWTPEPKYSDECGPEYSERKCGSQKYCNALDEAPRAPKTGFFKNAEECFDAHEPAPKLPWIKAPSLKTGPDSCGYSFTGNPAWAIYDDASCGTQVYCEAFDKIKPRPDFLFGFKNTKACLEAHDPPPSG

>MAPG_11370T0_|_MAPG_11370_|_Magnaporthe_poae_ATCC_64411_hypothetical_protein_(980_aa)

MGPDNVDKAVLSEMTLAASLEWSLLQRVGAHLRPRRSQARLRQSHHDGNLTFIEPDLRELLFYEKLVTEILPRATFAELQLYLEPLCSQLDQCVNPSLLSQNGAGDDTASTSAATYTEGVRGISATHYSRLRSLSSFSRTAEIWNWDLISTPGTGGTNSAIFLISPGAAAEDMLNAVKSCILLLRKLLENGLNGHSSPVTPTHHTAATATGSLYRARAHEILDVLFEQYTKMEPAHEHSVLLSVPVTSIVDMLLPKCFGPNKWQRTQCLSYEGDFSQHYGVDRWIEDICTEVQAEQTGSTLTVFLKDGRLFGARPHHGVSSTSRFDEKSLRSFHHLINTHAFRVPDLSSLQIPVVFSREQKAALALNLAHCLTEFFDSKSAWPLCDSKRIFVIYPPNERKYRGGPLYFTFSAGEISPDDMPLPESGMHPVIMAFAKLLLEIDNGKPIDLDQDSELHDQWAELCVQTKLAQDNGKGIYCKAVRNTLFFKSDKRPGEDPRTAIRRAMREDIITALEEVAKPPNLAGGKRRRSESEPSVNEHGGGNYPTVTRNQALSSTRLVKDNCSTPGREDGLPRWSKRQRMVTPPRAEVPRASGLDTEYPNLSSMLPNRPIEAVVGTQLEPAFHGGKQPTTAQWVNNLKKINIQVEQDRREHFPMTDQRILVAILDTGYDPDLIDRENVLRERDFVTESNIVGDSYGHGTYMARLVIACAPFAKLAIARVAENTSTLESSQDRIAKAILWAGCECQADIISMSFGFPSDSEAVSKAIGAVKDQRGGGVVFLASAGNSSMEEEAFPARHPSVISIQATDRNGCFLPSNARSSNGNHGPAALGTFGDVPSTLEFEGICNKYPKICQPGSSVATAVAAAISATLLAYANILPSLRSPNAVAELPFCRLRESQGMEALFRDSAMAQETSHRRWFVNPIRFWTENPGHEARYCSLYSLLDKFNRKVPRTTTHEKQGGICHGASQTAVRRDAQKE*

>MAPG_11419T0

FIVFKGWHAHDMTVDRLHGILGKGAKIAVVDTGDYSHRNLGGCFGGCKVAGGYDFVPDPMDQHGTHVAGIIAGVAPDATLYAYKIFAQETIIAFLRAYEDGVDIISASVGAEVSRRLVLVVVIAASNGQLGWQMSSGASAPDVLAVASVDADTCPLIRGASIFTSWGPNLSLKPDIAAPGGILSTYPGTSMATPAGVAALGRTAPPIQVGTGLVDAVAVTSMSNDTITNGRVDVEYRFSVQDAVVGPGLPVYSGKVPYFGGRLKPGRYAMRVAVLLPFGNW

>MCYG_00184T0

VVEPEGWFWLAIRENPEQLYDTISTPGRARYGKHLLDDLLRPRVETSEGIISWLDWIRFVKTAEQLMKTQFHVFRTLEYSVPASISSHVQMIQPTTLFTTACLRELYNRIGVSGFLEEYAQYRDLDLFLFSEGLIGGNTQEANLDMQYVVGLSHKTYYSTAGRNEPLEQLRYLVKLPKNQLPSVLSTSYGDTEQLPYTKATCDLFAQLGTMGVSVIFSSGDTGPGCQTNDGFNPIYPASCPFVTSIGGERASSGGFSDRFPRPQYQAVKGYLLFNPNGRAFPDIAAQGYAVYDKGVSGTSASAPAMAAIIAQLNDFRLAKGSPVLGFLNPWIYFTDIVDGGCSWNATKGWDPVTGFGTPFQAL

>MCYG_01100T0

RDYDYSPEQLLGEGQIGELEGHHTFSKRIWDPIFKQQWHLFNTLYPGNDLNVTGLWLEGITGNGSISAIVDDGLDMYSNDLKDNYFAAGSYDFNEMHSEPRPLLDDDKHGTRCAGEVAAVNDICGVGVAYDSKVSGIRILSKAINDADEAVAVNYGFQENQIYSCSWGPIDDGRTMDTPGILVRRAIANGIQKGRGGKGSVFVFAAGNGAGHGDNCNFDGYTNSIFSITVGSVDWNNEHPYYSESCSAQLVVTYSSGGGIYTTDVCSSQHGGTSAAGPLVVGVMALVLQVRPELTWRDLQYLLVETAVPAPGWQTTSIGFSHDFGYGKVDAYTTVHLAKWKLVKPQAWFHSTSFDITTEMLKKNNERVEHVTVTMNINHTRRGDLSVELHSPSGISYLSTARPQDDERAGYVDWTFMSVAHWGEKGVGNWTVIVKDDWRITLWGESIDPSIQRKRIRYEFLYDAF

>MCYG_01476T0_|_MCYG_01476_|_Microsporum_canis_CBS_113480_alkaline_proteinase_(translation)_(396_aa)

MKSLHGLVGLLFLSEDVINAAQDEIGNTIRKRNVGKRSTDNRPLSQEVISFRIDDESRGIIIDADDATIQTLLMSNEIATIEANTRGSLDAKVSQSDAPGNLVRLSKNLIDCVSNYDYDESGGEGITVYVLDGGIRLTHQEFKGRATFGARFSKSTSEEDQDGHGTHVSGIIGGVKFGVAKKVKLVAVKLDPEASQMIQALEFVLADVKKKGIQGKAVISMSMHVDGSEIVDKKFKHLVDSGVVVVMDAGQFSPGRDPSVITVAAMDHRNDFHWSKSNYGPSVTIYAPGVGIESSYFRSDTATRYLNGTSQATPHVAGLAAYIMALEGITQPAKVMSRLRELADDTGARVQWTAPNTTSLIATNGLAGKLDPAIANKVKKFPWISSVIQSWNCGIPDYSDEDCGTLVYCNSYDEWPETPKRGFFKSAQECFDAHEPAPILPWIEKPTTVRPESCSGEIISEGVLGYGERVCGTKLFCEAFDLDPKPEGYKNAKACFDAHEPQPSANKTTPAVAA

>MCYG_01559T0

VHEPVWWLTIALQQNLEHAEDYLSDPSSPQYAQYWVAAKFAPSEPTARKVMSWLGELYMAQEAEKLLHTTFYIYICEKYSVATFVEKYVDFITTTEQFTPDCLRALYNSLGIIEFTWVGYLESDLDKFFPKFESIGGIQTEADLDIEYAMALTHPTNYQVGDIYDPPDPTPASSDCGTHKPTKVISISYAYEEGFSYERRQCLEYLKLGLQGVTVVFASGDHGTACGNTVIYVPTFPSTCPYVTSVGGDAESAGGFSNVFAVPGYQATRDYLLNTGFGRGFPDVAANAYATAVNGVYGTSASAPVFASVIAWINDARLNMGKQPVGFVNPVLYLNDVAKGDCAYHASSGWDPVTGLGTPFDRM

>MCYG_01699T0_|_MCYG_01699_|_Microsporum_canis_CBS_113480_peptidase_S8_and_S53_(translation)_(696_aa)

MPIISINGNDLNPENQRPVLQALGLESEDSSKSNYILVQTKEPLEDDQEDELERLGVDIQEYVSPNTYLCNYKPADLAAVRSLPFVGWANVYLDMFVVQSTMKSASSTNTVADFTNTVRKSAHRRMVDIVLHRDVDSTSQELHEALSRATRADTSSLNVEPTKIRMMVQDQHLDEIAAIDGVRSILEVHPAVLFNNKATPIIRGEVDTNCDHPISSAEADKVDALPYEGEGQIVAVCDTGFDKGSTTDTHPAFKCRVKHLYALGRTNPATADDPDGHGTHVCGSVLGDGISEKMGGKIQGTAPKATLVLQSVLDSNNGLGGIPDDLTKLFIQPYKDHGARIHTNSWGSDAPGRQLPYDVSCEEIDRFVWEHQDMVILFAAGNSGIDANKDGINDSNQIGSQAAAKNCITVGASENDRPDITTTYAPWFPNKPYNTDRVADHPNGMAAFSSRGPTKEGRIKPDVVAPGTSILSTRSAKLLKPGTTFGTSTDPDWFFNGGTSMATPLVAGGVALIREALVKNGNKNPSAALIKAMLINGAVELPGQYIPTEAGPSPNISSGFGRVHLKNSISSPCDATAGYYEGGPLRRGQSDRKLVIRVPCKPNGCSTLKVTLVWSDPPGAMLQNDLDLIVTAGCTKRHGNMGEKKGFDRTNNVEQVVWTNVPSGEVRVTVRAHRIFKRRFAQCFAVAWSLDRPLK*

>MCYG_02070T0_|_MCYG_02070_|_Microsporum_canis_CBS_113480_serine_proteinase_(translation)_(491_aa)

MKSLDGLVGLLLSASLASAGLVPRDSSIPIVNQDSKNAVPNRYLVAFDPRLSKDVTQAERAKMIGTVKKRNVGKRSTDNRPLSQNPYTFDIDGETQGMIIEADDATIQTFAKSGALASIECDTRGSYDAQVSQSNAPDNLVRLSNNVIDGVSNYTYDESGGEGITVYVLDGGIHLTHEEFGGRATFGASFIPESPNEDVADDHGTHIAGIIGGAKFGVAKKVKLVAVKLDNEASQMIQAVDFVLEDVKKKGIQGKAVISMSMHNDPSDILDQKFKHAVDSGVVVVVSAGNNNADAGNYSPGRHPSIITVAAMYHKTDFHWPNSNWGSAVTIYAPGVGIESAATWSDSATRYHDGTSQAAPHVAGLAAYIMALEGITEPPKVMARLISLAEETGSRVHWTAPNTTTLIATNGLAGRLDPAIANKVKKLPWITDEEASDWGSCGRFYDSDLSCGTLVYCDSYDTRPTLPKKGFFESAQECFDAHEPAPILPWIEKPTTVRPDTCDKSTESAQCPRVCGSGGYDEYVCGTKFVCEGFDLNPKPNWAKGYKNAKACFDAHAPQPVSSH

>MCYG_02345T0_|_MCYG_02345_|_Microsporum_canis_CBS_113480_proteinase_R_(translation)_(401_aa)

MTGKHGIPELPLRCLRVSRKILGPRRRPDTSEAIAHRPEVTRRSKLQLDAPWNLARISQVHARNDGLRRYRYDATAGSGAYVYVVDSGINFSHAEFSGRAFRGANFVLGSPDDDESGHGTHIAGIIGGKTYGVAKSCTMISVKVVSKSGRSNMLWIRQGISWATNDAIAKGIADRSVINVSVGGLYNASVNFAVKNATDAGITVVVAAGNHATFAGAFSPASADTAITVAASGPDDCRAPFSNYGRCVDMFAPGAHIASAWNYVDNGMKYESGTSAAAAHVTGLAAYFISSENLRGFKAVRERILGASLNGVITDTRYSSNRLAYNDNRVS

>MCYG_02748T0_|_MCYG_02748_|_Microsporum_canis_CBS_113480_alkaline_serine_protease_(translation)_(389_aa)

MLNLLAITSLRASRKILCPIQRPATSDTIARRPEVTRESESQPNNPWNLERISPGYATNHGSPRCHDDETEKPGAYVYVIDSGINFLHAEFSGRALHGANFVPESPDYDEVGHGTHMAGIIGGTTYGLAQNCTMISVKVVDKRGKGKQEWLRQAIEWATNDAIAKGVAEKSVINISLGWKYSATTNHAVKKATNAGITVVVSAGNQSKPASTYSPASASTAITVAASGPDNRRAPFSNYSLCVNIFAPGTRVPTVPSSVDNGPQYSSGTSPAAAQVSGLAAHFISSENLRGFKAVRKRIMRASLNGVIKDRRWSRNRLAFTGVSRD

>MCYG_03645T0_|_MCYG_03645_|_Microsporum_canis_CBS_113480_conserved_hypothetical_protein_(translation)_(926_aa)

MDDDTDSRSLLQSVLGLFALGRPQRSAKPASPKSQKREAWLRTDAFPQRNDLGEAEPESVARTLRAFEHLVQDDSDRKIDSFAEQSFQQPRNEQYPELRRMVQNPNRDPHAGPGTVIRVGKRRRECDDAVQELSHLEQESKKRRVLPREEPTLGPNLEDPDAEFAVLMRRHLVSLDNALRRHWVCVCQKCSGLSVRLSLPQHQKDLEAEASFEVFFGVQSVPRTVLQEAKITVKRQHNSTLRRSSEPVISNPPDFTLCQSITESLGQRNCLHFALENGIFQRLRPQPKEFGSDQMSRAVSLSALFNRQQELLRGESVLPLKGKRVLAVTLASALLPFLETPWLQFSFNHSKIQFFEPWQDGELPNITKPFLALEHVPIISARGTDTGDSSDASKHMIHPNASVLALGILLCELHYCTPVELMAKDPHVARNVNDDFYTCLDKLQTLEDDAGVDYYLATKACLTGEYYPLGQHADFEDISVQRLFYQNVVKRLEAVIFKAWGIRLENLGSFDSRQNESCWGSIGREVVRLHTGKVDSSNANNTARASPHRSMPDTAPASYASRHSDIVLRMPAQPSGSQTHGRLTETSQKSLYFFDASHQTGPKQENPLSEGWMDNLLSSIYPYVDPVSELAEPVRIAILDSGLDPENPFLIEDQQLANPRVKEARSFVHGTRPHDIRDEIGHGTHALGLLLKVSPCAEIYVARIARGETLDPNTYDDIAKARLFEQWKVDIISMPFGIREYHERMSTAISNALNKRTLLFAAASNYGANLGRAFPAQYPSVFCIHSTDGNGNPSNFNPTASETDVNFSLLGEHVSSHWPAGVNGHDQIVNVMSGTSVATSIAAGLAASVLSFVRLQDQHIAVESERLGPWLKRDNSMDAVFKSMVRRRRGVGYDYIMPHVLFDSGSTREDVYGRIKDIKRNMYKY*

>MCYG_04040T0_|_MCYG_04040_|_Microsporum_canis_CBS_113480_alkaline_proteinase_(translation)_(398_aa)

MHFFAIVGLAAAIASPLASGAVTPPAPNLARPPLPPIPVRPRSASAVVLAKNNEVPEDLVIPDTYIIRYKPSLDVLRRLQHEEDIDRMAKKDEKRGIFDRFDIPGLQGYVAEISPSELKNLTECDLKDTIIKTTAVAASPALAKRTMVKQYNAPWGLARISHYSRHRRDYVYSDTAGTGTVVYVVDTGIRTSHADFNGRAIWGANFLAETSDSDEDGHGTHVAAIIAGSTYGVAKNATVIAVKVLDKTGSGSMSGLLQGLNWAVDDARRRGVVSRTVINLSVAGTYTQSVNDAIKIATDAGITVVAAAGNKNDDVANWSPGSAPTAITVGAIDEDDRRGEFSNWGPGVDIFAPGVSINSAYNTGDYETAWLSGTSMATPHVAGLAAYFMAREGLSGSEVTERILGAANKGVADRHEGADRIATPFEIFISSLSLLFLSISATSVTTTSPNCPSVNPSLSTTNRPSFRLVPPPASAPSLATNLATYPPTPPSSTSTNAPSSTTHFNLPCAPAPSPVQLTNFLRNRYRPPPTSPPTPPTARADTPSTLVRRQAVEPAPRPGSGPLPSPPRVDGQLRGEGEGRTGRGW*

>MCYG_04495T0_|_MCYG_04495_|_Microsporum_canis_CBS_113480_alkaline_proteinase_(translation)_(486_aa)

MKLVIFLFLLAELIGLTLAQAKVQNANIEPQLVVPNSYIIKYKSKASSSSKNKHEKAIHKKAKSKGKEGIVDNIDLDGFKGYVAEIPSSELQEVIDSDLVDYIEQDTIVNVSAVAAPSLDPLTKRGYTSQTSAPWGLARISQIYSEKGVDARYSYDATAGAGSTVYVLDSGIRTTHKEFGGRALWGANFISGSPDTDEYGHGTHVAGTVGGKTYGVAKGCRMYAVKVIDKNGGGTMSNILQGLQWAVNHAKSRGTTKTSIINASLGGPYTKIGNAAVKAATDLGITVVVAAGNDGADAADYSPASAPSAITVAAIDSASYRTLWSNYGTVVDIFAPGSDILSAGHLSDSSSVYKSGTSMAAPHVAGLAAYFMAKEGLRGSVAVTKRIISAADTTAVEYAVDSPQRVAYNGGGK

>MCYG_04669T0_|_MCYG_04669_|_Microsporum_canis_CBS_113480_intracellular_serine_protease_(translation)_(932_aa)

MGWEVKLLLDYLSAAICFLGVPSQPSSHPPSHRRRHHLPDHSAAWSRADLAALYNRLEESPDEQTAHDILGELNSGITAEGAEECTPSNYPYVEQRTRIYRENREQPDNLRAKKRKVDFVPAELQVVEQWAERDNPKKRRKAYNAAESNVEAAPDQAEYSYHSEVKGKHEEYEHEILTLKNKSICLYRELQSHWSCACEQCQGICLQLPWPYPDSDDVQLDGYFALGSNKIENWQEGKFFMTYQKHGLVRIADESDNTAGEYWSALDHTDICPSIKDCHKKSASLHIMVEDNKLQELRMGDNMCEHTPAAISLKSIFDFKQKIGGSSVFSRRGKLTLALYLTEMLRPVLETPWLLPNFSCHDVYFFRRPGALPDLTYPFIFTSANTFIQDTASQGASKETVHPNQTVLALGILLLEIFEFKPFADENHGPSRKRSLDQEKIQARRTLRAMTNEEGADFCDAVSACLDAKYFPRGLESEFDTPAVQDSFSAKVVSRLYNSTNNWINDKKDLLSLRENETEKRALWTVEAQNKCTEIGSHISSSPSAKAAVASTYQPPADTLMKPTARSAAVPLRTTNYSQNKEAVIVHRNYNCSPIAQAPVSEGGLFDFLEHPASDSVSWLKELDRTLSTYVGNPPQSSSGALIRIAILDTGFTRDNDNPEPRLKATRNFLLDTDDPQFMHDKVGHGTHTLGLLLRTAIGADIFVAKVSDTRTIGRDGYEPIIKAIRYATERWEVDIITMSFGIREFHDSLSKALSFAHNRNVLLFAAASNGGPLTNRAFPASEHLTVFGIHSMDGQGSLSKFNPKPVKTAFNLVALGEAVPSDWPKGIDGHREDFRALSGTSIATPIAAGFVASLLEYVRQQEAGLPQDSSIMGWLKRSQAIQATLVRFATDKVDGYHRLHPKKLFNDPDTKESIYDQIKKFKKYDYLHEI*

>MCYG_04955T0_|_MCYG_04955_|_Microsporum_canis_CBS_113480_proteinase_K_(translation)_(407_aa)

MKLNATLFGLTGLLGLALAAVPIKNDGISADIVVPEKYIVKYKADADAGRKKKHESDITNKAKKKNKKGVVESINIDGLSGYVAEIPDSELKELRDSDLIEYIEKDTVIQINAVAAPRVAADPVEEKHQLAKRAYVTQLHAAWGLARISRRSTWNSGYYYDNTAGQGIRVYVLDSGIRTTHVEFEGRAVWGANFIAGSPNTDEYGHGTHVAGTIASKTYGVAKKATVVAVKVLDKNGSGTMSGLISGLNWVVNNAKARGIAKKAVINISLGGGYTASVNAAVKGATDAGLTVVVSAGNSNANSANYSPASAPSAITVGAIDGTGYRAWFSNWGNLVDIFAPGVSVLSAYHTSNTATWYMDGTSMAAPHVAGLAAYFIAKENLSGSPAVTNRILGAAVTGSIGDPKGSWNRRAYNAGGA*

>MCYG_05446T0

VLEPQGWFKLALQDKAAAFEQHVSNPKHENYGKHMVDAFLQPPAHMTDSVFNWLDWLTFVQKAEKLLNTRFYNFRTLQYSVAETVAPYVHMIQPTTKFTPDCIRDLYNRLGISGYLEQYARLDDFSTFIFDFKSIGGNEQEASLDVDYAIGLSGTYYGTAGRNEPLEQLHYLLGLPDSELPAVLSTSYGENEQVPYTDSACHLFARLGARGVSVIFSSGDTGVGCQSNDGFNPIFPAACPFVTSVGGEVASSGGFSERFSRPWYQDVNHYLMYNPSGRGFPDVSAQSFATRDHGVSGTSASAPLFAGVVSILNSIRLAHHKPRLGFLNPWLYFTDIVHGGCSWNATRGWDPVTGLGTPFETL

>MCYG_06077T0

TVEPEGWMNLALQEKAHAFEQMVSTPGHSNYGKHLLKDFLRPRKEVSDSILSWLDWIHFVSQAERMLKTRFHYYRTLQYSVPSHLAPDIHMIQPTTKFTPQCLRDIYNVIGVSGYLDQYARYNDFYKFIFSVKYIKGNLQEASLDIDYALGLSNVFYTTSGRNEPLDQLHYLLSLPSDELPAILSTSYGENEQVPFSNATCSLFAQLAARGVSVIFSSGDTGVGCLTNGRFNPTFPASCPFVTSVGAEMASSGGFSDRHIRPRFQAVLTYLLYDPRGRGIPDVAAQGFAVYDHGVSGTSASAPAFAAIIANLNSIRLNANKPVLGYLNPFIYFTDIVHGGCSWNATEGWDPVTGVGTPFEIL

>MCYG_07213T0_|_MCYG_07213_|_Microsporum_canis_CBS_113480_oryzin_(translation)_(400_aa)

MKFLLISLAAIAAALPTDKGIIPGKFVVALKPTDDLNLHARWVSDVNRESLSRRGVESVGIDKTFSFPGFKGYSGSFDEETIKIIKANSTVLRVEPEQEYTVAAQVTQQSPPWGLSAISNAKPPSSKASYTYDSTAGEGVFAYVLDSGIYLEHEEFQGRAVFGADTSGVTSQKQHGTLVAAIVNGATYGVAKKATVVDVQVLGDSTGTTSGVIDGVAWTVNDIVAKKRAGKAVINMSLSGASSEIMNDAVQKAIDAGVPVVAAAGNMNVNAADWSPGNNPNVITVAASNKNYQRWQHSNWGPACDIFAPGEEILSAWPTSSTGSRTADGTSEAAPHVAGVIAYLLALEGPRTPAKIWERVKELAIKDKITDSKGVPNLFLYNGIGK

>MCYG_07696T0_|_MCYG_07696_|_Microsporum_canis_CBS_113480_pen_c_1_(translation)_(402_aa)

MLYQTVVSLGLATIALALPKDKDSKALAASKFIIALKPTTNIDLDLHARWVSDVHGQALTHHGVESSGIDKTFSFPGFRGYSGSFDEDTIEIIKANSSVLHVEPDQTFTIASPVTQQSPPWSLSAISNANPPSSKASYTYDSTAGAGTFVYVLDSGIFLEHEEFQGRAVFGADTSGISSKKQHGTLVAAIVNGATYGVAKKATVVDVQVLGDDSGSSSGILAGLSWAVNDVVSKNRAGKAVINMSLSGGNSVIFNEAVQKAIDAGIPVVAAAGNANDDASKSSPGNQPNVITVAASNKNYQRWAWSNWGSACDIFAPGEEITSAWPTSASATYIASGTSEASPHVAGIVAYLLALEGSRTPAQIWSRLKELAIKDKITDVKGVPNLLVYNGIGK

>MCYG_07961T0_|_MCYG_07961_|_Microsporum_canis_CBS_113480_pen_c_1_(translation)_(401_aa)

MVYKTSLLLGLAAIASALPTDKDSKQGLVAGKFIVALKPTANVDLDLHSRWVSDVHGQALARRGVESGGIDKTFSFPGFQGYSGSFDEDTIEIIKANSSVLLVEPEQVFTIASPVTQQSPPWGLSAISNANPPSSKASYTYDSTAGAGTFVYVLDSGIYLEHDEFQGRAVFGADTSAVSSKKQHGTLVAAIVNGATYGVAKKATVVDVQVLGDDGGSSSGVLAGLSWTVNDVVSKKRAGKAVINMSLSGGNSVVFNEAVQKAIDAGIPVVAAAGNSNEDASQSSPGNQPNVITVAASSKDYKRWAQSNWGSACDIFAPGEQIISAWPTSTSATYIASGTSEASPHVAGVVAYLLALEGSRTPAQIWSRLQELAIKDKITDTKGVPNLLVYNGIGK

>MCYG_08690T0_|_MCYG_08690_|_Microsporum_canis_CBS_113480_preproalkaline_protease_(translation)_(424_aa)

MSYKTLFLLGLATITLALPSDRVNKEGLVAGKFIVALKPTANVDLDLHARWVSGVHGQALARRGVESGGIDKTFSFPGFQGYSGVFDDNTIEIIKANSSVLHVEREQSFTIASPVTQQSPPWGLSAISNANPPSSKASYTYDSTAGAGTFVYVLDSGIYLEHEEFQGRAVFGADTSGVSSKKQHGTLVAAIVNGATYGVAKKATVVDVQVLGDDGGSSSSVLAGLSWTVNDVVSKNRAGKAVINMSLSGANSVIFNEAVQKAIDAGIPVVAAAGNSNEDASKSSPGNQPNVITVAASNKDYQRWAWSNWGPACDIFAPGEQITSAWPTSASATYIASGTSEASPHVAGVIAYLFALEGSRTPAQIWSRLQALAIKDKITDVKGVPNLLVYNGIGK

>MCYG_08753T0_|_MCYG_08753_|_Microsporum_canis_CBS_113480_secreted_serine_protease_(translation)_(396_aa)

MARLAFLALGSTAPAVEQWIVALKPEVSSDADLKLHARWVSDLHAKNVQKRDDAPAGLEKTFQFPGFSAYVGSFDEDTLDAIKHNPNVTAVEKNPEVFLASPITQENPPWGLSAISHANPPSSSSNYRYDSSAGAGTFSYVLDSGLLASHREFEGRAALAYDATNGAATDHGHGTHVAGIIGGVTYGVAKKTSIIGVQVTGSSSGSGAWILDGLDWTVRDVISKNRVGKAVINMSLLTGSSTVVNNAVQAAIDNGIPVIAAAGNSNVDSADWSPANLPAAITVAASNSNYQRWRASNWGSVVDLFAPGESITSAWYTSSTSTYTTSGTSQAAPHVAGVVAYLLALEGPRTPAQIKARILALATKDLISDRREVPNLLLYNGNGA*

>MGG_00282.t1

FIVFTGWPVHKMGVDRLHGITGAGIRIAVVDSGDTSVHGLSETKITYSVPDNCSIHGTHVLGIVGMYDDLIGFTEAAKRGVDIITCSFGAATRIAIYVSLPAGNAGPGFTGVNPATAPVVAATGSVDNLITSGTYYSSWGPSRTMPTYMAPGYILSTFPGTSMATPAGVAALAQPAPILQQGGGLLDAYAATYVNNDTISNGTKELTYNLQHVGATLGPGVSYFGGYVPYTGPAGDYFWRIKMLRLNGAW

>MGG_02531.t1

FIIFTGWLNHIVTVSKLHGLFGKGVKVGVVDTGWYDHPALGGGFGGFKVAGGWDFVPDPIDSHGTHVAGIVAGVAPEAELYAYKVFSQATLISFLRAYEDGMDIITASIGAEVASRLVVVVTISAGNGAIGFYGSSGSSGRNVIAVASVSTDGCPLVRGANTFTSWGALLQLKPDIAAPGGIFSTWVGTSMACPAGVAALGVAAPPAQVSTGLIDAFKVTQLENDTVTNGSSPVSYKFSSQPATLRPGLPLYGGKVPYMGGKLAEGRYMMRVAVLKPFGNW

>MGG_02649.t1

YIVFQGWRNYSATVDRLHGILGQGVKVGFIDTGAYRHPALGGGFGGFKVAGGWDFVPDPDDQHGTHVAGIVAGVAPNATIFAYKVMGTETLVALLRAYDDGMDVITISISAEVASRIVTVVTMSADNGDRGFWIGDGASGRNVIAVASVDADGCPLIRGASIFTSWGPLLTPKPEIAAPGGIFSTYLGTSMATPAATAALGVAASVAQVGNGLVNAYKVTVVDKDTVRNGSSTVTYTVSMSAAVLQAGLPTYNGKVPYLGGKLAPGNYTMRFAALRPFASW

>MGG_02863.t1_gene_MGG_02863_Mgri_7015_supercont5.1935.193:complement(join(1179214..11801131180180..1180485))

MFTSPKLLLILLASASSGVLSAPLDTEAVAAAGRFVVTLKPNAAVDLDLHARWVADVHARNLRRRGDITPGLSTTFSFNDFAGYAGAFDDDTLEAIRANVNVSSVEPDSFFALTSSTPLEKQKNPPWGLSAISRASPPSSNSSYVFHRSAGQGTFSYFLDSGIYTDHVDFEGRASLGYIAAGAENAVTDKAHGTAIAAVIGGKVHGVAKRTSLIGVKVSGSIEGSLSWLIDGVTWTANDIVSKGRVGKSVINISMVGLNSPAINSAVQAAIDAGIPVAVSAGNFNSDSADWSPANLPDAITVAASNSNYHRWAASNWGPTADIFAPGEDILTADVKSPTSTIRHSGTSEAAPYVAGVIAYLLALEGPRTPAQIKARLLELATKDVISDPQGVPNFLLYNGSGA*

>MGG_03316.t1

FVVFNGWPVHIDTIDMLHGIKGNGKRVAIIDSGDWKHPALGGCFGGCVIEGGWDFVEDPYDNCHGTHVTGIIVGAAPGAKIRMYRAWNCEIYIAFLRAFDEGADIISLSAGAMVASRIAVPVVVAVGNGGAGFYTLNPAAGRSVLGVGSVRNDACVLLRGGSTFTTWGPTLSLKPSVAAPGGIFGTFPGTSMACPAGAFALAKAPVAQQGAGIIRAFDATTLSNDTIENGSDEATYALGHTRATVPAGLPIYGGYVPYLGGKLPAGSYKIVLTALRVFGDW

>MGG_03670.t1_gene_MGG_03670_Mgri_7015_supercont5.1345.134:complement(join(231030..232268232367..232738))

MFTSRRFLLTLLASASSGVLSAPLDTDVVAAAGRYVVTLKPHAAVDVDLHARWVADIHARNLRRRGDITSGLSSTFSFNDFAGYAGSFDDDTLEAIRANVNVSSVEPDSFFALTSSTPLEEQENPPWGLSAISRANPPSSNSSYVFYRSAGQGTFSYFLDSGIYTDHVDFEGRASLGYVAAGAENAVTDEAHGTAIAAVIGGNVHGVAKRTSLIGVKASGSHGGSLSWLIDGVAWTANDIVSKGRVGKSVINISMLGTNSPAINSAVQAVIDAGIPVAVSAGNYNSDSADWSPANLPDAITVAASNINYRRWAASNWGPTADIFAPGEDILTADVKSPTSTIRHSGTSEAAPYVAGVIAYLLALEGPRTPAQIKTRLLELAAKDLISDPRGVPNFFLYNGSGA*

>MGG_03870.t1

YIVFNGWPVHMQTVAKLHGFTGKGMKIALIDSGDYTHPLLGGCFGGCKVSFGYDLVPDPMDCHGTHVAGIVGGVAPDAELGAYKALNCEMLIAFNMAYEAGADIISSSTGSSAVSRIVVPVVIAAGNGEQGWRPSSPSAGRGVTSVASVDNDACYLGASYWSSWGPDLFATPNVAAPGGIVSTYPGTSMACPAGALALSKHSVLQGGPGLLQVFDAGLLSNDTLKNGKREATYELGHRPARVPAGQPFYSGFIQYQGGLMPEGKYKIAVAALRLFGDW

>MGG_04733.t1

YIIFTGWYNHWATVDQLHGVFGDGVKVGVVDTGQYTHAALGGGIGGFKVAGGWDFVPDPMDYHGTHVAGILLGVAPRATMHSYKVFGRETIMAFLRAYEDGMDVITASIGAVVANRIAVVVTIGAGNGEGGYYASSGSSGEYVLAVASSYFTSWGGLLSVKPDITAPGTIYSTYIGTSMATPAGVAALGSPAPVHQVGGGLVNATAAMQLENDTLTNGATAASYTFDHEDWELQPGLPVYSGRVVYQGGKLVPGNYTSW

>MGG_07358.t1

YIVFPGWQLHGSVKELHDITGSEITVAVVDTGDYLHPALGGGVGGFKVRFGIDLVPRPYAECHGTHVSGIVAGVAPAANLEHYRVVGCDMIIAVLMAQAREVDVLSLSLTSEVLTRISILVVVASGNGWRGFSARAPASAREVLTVGSVNDACILVGGANGQGSWGPTYDLTSILAPGQIWSTIPGTSMAAPAGCAALARPAPVAMQGNGVVDAMGATFISNDTIQNGGEPAEYNLSHKPATITPCPLYSGFISYSGGLAEGLYKFKVCALRAW

>MGG_07404.t1

VFDPRGWLRIALQQNAAALEQVVSNPRHANYGQHLLRSYTAPTPRAVRSVTSWLDWVTLVGAADRLLGADFAWYRTLSYGVDDSVAPHVDLVQPTTRFTPLCLRTLYNLVAFASFLEQYARYSDQQAFTFSVETVGGNDQEANLDLQNEPLEFLTYLLAQPDSAIPQTLSVSYGEEEQVPYAIKVCNMFMQLGARGVSVMFSSGDSGPGCVRASDFGSTFPAGCPYVTSVGSERASSGGFSIYHARPDYQVVPKYIFFDGNGRGIPDVAAQGFVVIDKGISGTSASSPAFAGMVALVNAARKSKDMPALGFLNPMLYMTDIVNGGCRFNATAGWDPVTGLGTPFDKL

>MGG_07965.t1_gene_MGG_07965_Mgri_7015_supercont5.1835.183:join(877096..877398877453..878292)

MILAIISLSVVICREVSDYIVMFDQDPSMDRASMKVLYNMNIHEAQNILRPDESIGIKMTNGFVARMSESTAERMKRHPSVKMVVKDSPVGISGLKFDVPGSDDRSGIVMQRHAPWGLARVGGSVSLVHGNYCYPINSGKGVDVYVLDTGVEIEHPEFGGRARWGANFVPKSPDRDEHGHGTHCAGVIGGKNFGVTKESSIIAVKVLDKYGSGMTSRLLQGVDFVIKEHEKKKDELYNAAADEYLSSGGSSDIEIEMDGSESFSFVQPETPSIQRLVDAISRKALQPKTVVNLSVGGFRNAALNFAIEYASRLGIHFSTAAGNEHEDACDFSPGSSRAAITTGASTYRDTVAFFSNFGKCVNVFAPGVDILSSWIGGTQKIVSGTSMAAPHTSGAIAAYLTYYDYDPHMLKSRIIGDARLIEDVSEDDYDGTTIWPLPSLFNANKKKLPILSMERLLRRVRNKMR*

>MGG_08415.t1

YMVFKGWPVHVMAVDRLHGITGAGVRLAVVDTGDYTNAILGGCLGGCVVTHGWDAVPDPMDCHGTHVAGIVAGAAPGVSLGAYRVMDCETIAGMLRAFDDGNHILTLSVSSMTAARIVVPVFVAIANGETGFDPVAPADARHVGSVSSFDPGGCVLGASSFSSWGPTLRPAPVFGGPGRILSTYGTSMAAPAASAALAIPHPLQAGAGLVQLWDAGVLSNDSLRNDAAPAVYRLSHTAASVPAGWPIYSGRVPYVGGLMQEGRYSILVSALRLFGDW

>MGG_08429.t1

VMVFVGWPVHYSTVDALHGVFGKGVRIAVIDSGEYTHPALGGGLGGFKVSGGYDLVPDPMDHHGTHVAGIIAGVAPEAEILAFKVFGADTLVATIMAYEAGVDIITASIGATIASRIVVVVTIAAGNGEDGVYASSGSSGKDVLAVASVDTTGCVLVRNASYFSSWGGTLEIKPDVAAPGDIYSTYMGTSMATPAGVVALAETAPVAQVGGGLVNATKVTAVSNDTLTNGAAPLTYTFALQPATVQPGLPIYSGKVPYYGGKLVPGTYKMRFAALTPFGGW

>MGG_08436.t1

FIVFTGWPMHKYTVEHLHGLSGKGVTVAIVDTGDYSHPALGGCFGGCKVAGGYDLAPDPRDRHGTHVAGILAGVAPGATLLAYKVFGSDILIAFLMAYKDGADVITASIGAEVASRLVVVVTISASNGQTGFFASTGASGKNVISIASAEGDACALIRGAKPDVAAPGGIFSTSLGTSMATPAGVAALGAAAPPAQVGSGMVNATKVTTIDNDTLTNGDAAVTYRFSLQPAVVAPGMPLYGGKVPYVGGAMNSGQYTMRFAALRPLMSW

>MGG_08966.t1_gene_MGG_08966_Mgri_7015_supercont5.1965.196:join(3015769..30160203016108..30162933016399..30169173017031..3017216)

MKDIVVDAVEFVEQNQIYKAPFLANNKQKIPYSSNLIKSDYLAINQKRNFVTQWNVPSWGIARINHRDLQDLTLYTVEEAAGAGIHVYVLDSGIQEDHPDFEGRAHTEANFITYEDSGDHSGHGGNIFGVAKKTFIHAIKILDRNGDGTTSALLQAGNTGDDACDYSPAANPDVFAVGASDSGDNIPSFSSFGSCVSMYAPGTNITSTWIKDSALTMDGTSMANPHVSGIAAMLMSKRIFQSPHELYGILRAMATPNTLKPQDKASGALLAYNGPNAL

>MGG_09073.t1

YIVFKGWPQHLMTVNQLRGVVGKGIKVAVIDSGDWKHPALGGCFGECLVSFGYDLVPDPMDCHGTHVSGIIAGAATGVTMGHYRVFGCDVLIAYNMAYQDGADLITASIAAVAVTRIVVPCVISAGNGATGFFTSSAADARGATAIASFDNNACVLIRGASTYTSWGPTGQVKPQVAAPGGILSTYPGTSMACPAGVYALAKPAPVPQQGAGLIQAYDATLLSNDTISNGSSSVTYTLSQRGAEVGPGLPVYSGYVPYMGGILPSGHYKIVFRALRVFGEY

>MGG_09246.t1_gene_MGG_09246_Mgri_7015_supercont5.1865.186:complement(join(751806..752669752742..753413))

MRFLQSTSVLVGTILPFLSAFPTYRPNENAIIPDKYVVTFKEGTTDAEMHAHTAWVSSVQRRNLAAGFTTAEAPGVEGMYNINTFNAYSGSFDRETIKEIKSHPNVKSVEPDRIVYVAGLVEQTDAPFGMKRISHRSLPITDGYWYDDKAGEGSFVYVMDTGINKAHNDFEGRAIPGVNLHPDSAFDDSTGHGTHCAGIAVSKTYGVAKKATVVDVKVFNRASGAWSLLISGLDWSVKNITGEGRLARSSVSISISGATYGPMNEAVKAAVDAGVVVVAAAGNDGRDASRNSPGNAPEAICVGSINSRRNTDTRSRFSNYGSTVDIFAIGEGVLSTARNTRTGTANMSGTSMATPHIAGLVGYLQSIHNLPNPAAARRMLLQLASTGTIADGRGSPNRLGYNGSGK*

>MGG_09352.t1

FIVFRGWHAHDMTVDKVQGILGKGVKVGVVDTGDYTHPNLGRCFGGCKVEGGYDFVPDPQDAHGTHVAGIIAGVAPEATLYSYKIFGFETIIAFIKAYEDGMDIISASVGAEVSRRIVIVVVIAAGNGELGWAMSSGASSPDALAVASVDADACPLIRGASTFTSWGPTLSLKPDIAAPGGVLSTYLGTSQATPSGVAALGKTAPPIQVGTGMVDAVAVTDMSNDTITNGQESVEYTFTVQDAVLAPGLPIYSGKVPYFGGKLAPGRYQMRVAALAPFGNW

>MGG_09817.t1

YIVFTGWPNHAATVDRLHGILGQGVKVAVVDSGWYKHPALGGGFGGFKVAGGWDFVPDPLDTHGTHVAGIVAGVAPNATILAYKIMATATIIAWLRAYSDGADVITMSISAVLAARLVVVMTVSAGNGADGYYAGDANTSPHLLSVASVGADACALVRGASVFSSWASTLQLKPDVAAPGGIFSTWPGTSMATPAGIAALGSSAPPLQMGSGLVDATRVTALDNDTVTNGAAAVSYSFALEPASLGPGIPAYSGKVPYMGGKMAPGNYTMRVAASRPFAGW

>MGG_09990.t1

FIVFRGWYNHRATVEALHGILGQGALVGIIDTGDYTHDALGGGIGGFKVAGGYDFVPSPRDRHGTHVAGIVAGVAPEATIYAYKVLAATLVAFMRAYEDGMDVISASIGAVIASRLVVVVTIAQGNGFAGFHGGTGSEGKNVIAVGSVNADACPLVRGASDFTSWGGSLGFKPDISAPGGIFSTWPGTSMATPAGVAALGEGAPTAQLGAGLVNAVKVTTLDNDTVRNADGPVTYNFSLVAHTLQPGLPLYGGKIPYMGGVLERGSYKMRFAALKPFGDW

>MGG_10445.t1

YIAFNGWPVHVQTVDKLHGLTGKGVRISIIDSGDYLHPALGGCFGGCIVSYGYDLVPDPLDQCHGTHVAGIIGGAAPGATLGMYRVFGCDVLIAVTKAFEDGSDIITGSLGSVTVSRMVVHVTFAAGNGTEGFGISAPSTGDGVLAISSYEADASRFTSWGPNLQLKPQFGAPGGILSLWPGTSMATPAGVIALAKPAPVVQQGAGLIQALDAITFDNDTVTNGQEVATYNVSHSPATLAPGLPMYSGWVPYFGGSLPAGRYRLGISALKIMASW

>MGG_10449.t1_gene_MGG_10449_Mgri_7015_supercont5.1805.180:complement(166441..167595)

MRLFQSTCVLVGTVLPLFTAFPISSPREIEIIPDKYIITFKKGIDQAAIEAHTAWVSSVQARNIARGFTTAETPGLERMFSIHNFNAYSGSFDRETIEEIRSHPNVESVEPDSMAYVTELIEQRNATYGPRRISHREIPTGDNSYWYDSKAGEGSFVYIMDTGINKAHVDFEGRAIPGVNLHDVAFDDTHGHGSHCAGIAGSKTYGVAKKATIVDVKVFTRGGGAWSLLMDGLDWSVKNITGEDRQAKSAVSISISGPTNQAMNNAVKAAVEAGVTVVVASGNDGRDAGRNSPGSAPESITVGSINSRREMDTRSSFSNYGSSVAIHAPGEGIISTYKGSRDATANMSGTSMATPHVAGLIAYLQSIHDLPDPAAARRKLLELATSDKIQDVRGSANKLAYNGSGK*

>MGG_13469.t1

RDFDYEPARELGVGQLGELDSHHVFSKRIEDPIFHEQWHLFNSVQLGHDVNVTDVWLSGVTGKNATVSIVDDGLDMYSDDLKGNYYAKGSYDFNDKTEEPKPRLSDDRHGTRCAGEVAAVNDVCGVGVAFDAKVSGLRILSKLISDADEAVALNYDFHNNHIYSCSWGPPDDGKSMDAPGLLIRRAMLNAVQKGRDGKGSIYVFASGNGAANEDNCNFDGYTNSIYSITVGAIDRKGLHPYYSEACSANLVVTYSSGSGIHTTDVCYNGHGGTSAAAPLAAGIFALVLEVRPDLTWRDMQYLAFMTAVPSGDWQDTTIGYSHTYGYGKVDTFGIVEAAKWKLVKPQAYFFSTKFEVTEDM

>MGG_13977.t1

FIVFRGWHSHHMTVDELHGILGKGVKVAVVDSGDYTHPALGGAFGGNKVVGGYDLIPDPMDHHGTHVAGIIAGVAPEATLLAYKVFGEDTLIAFLRAYQDGADVITASIGALVASRLVIVVTISAGNGQAGFGASSGATGINVVSVASVQGEACVLARGASAFTSIGSTLAIKPDVAAPGGIFSTFLGTSMSCPAGIAALGQPAPINQVGTGLINATKVSALSNDTVTNGSTPVTYTFQATPFTLQPGLPLYGGAIPYMG

>MGL_0154.t1_gene_MGL_0154_AAYY01000001:complement(join(318543..322277322282..322359))

MCTKSATYGVADTFPKHGLLPKQATNSRSRQRFPDYDGRNVRVAVLDTGVDPAALGLDGPNKVVDIIDCSGAGDVPLQQVEAQARTDDSAILELESPTTKRKLLVDAAWPNPSGVWKVGTKRAYDLWPTGLVERRTKERKKAFDVSHAALLQRALDELASERASTPSSTSDAKDRDAAAQRCEELQARVSVLKDMHKAWKDPGPVLEAVVFHDGMHWRAVVGGAEGDVIDSSKGEPESQHAMVLDLREKPRLTDYRLEREWAYFGEMDLLTYSVNIMNDGQLLSIVTLSGTHGTHVAGIIGAQTQDPATDGVAPGTEIVSLRIGDARLGSMEQGQALLRAAQALIDTRCDVANMSYGEDGAFGVEDKGAFAHALHQVIREHGVCFVSSAGNNGPALTTVGQPGGTTSGVLSVGAYVTAGDMQQAEYALVERGVPSNVTTWCSRGPTADGAAGVSIYAPGAAITSICRYALQSTQLMNGTSMSSPNAAGAVALLVGACKPEGITPTPFRIFRAIQESGADVRDPQGIKFLDVEKAWDYILAHRDDPYADADMRVRVTRAGKPLNVVDQRGVYLREVEETHRTTQFLVTVQPTFRSGETQRAYKLDLKTSLSATQPWVHVPEFLALGGNGRTFEIRIAADALPPGLHTAQVIAHDTERNGAVVFDVPITVAKPVVLPTATYAYPRVRLASGDIHREFVQVPMGATWADVRVRSVKHEAPGTSVRFWLHMLQLVPQRRLSKVEQHFVLALNENEPISKRVPVYGGMTLEVCAAQFWSSKAGFELELDVEFHGLDTVPKLVAHSGDAHTKVDVTSLVRCEDLKPSASLDTRRTYVRPSKYVLRPLREPRDRQPSGHQLHELVLEYPVIVKDACALTWRLPLSGYLYDASVTLLTQLLDVNQAQVAFGDVYAKPVDVAKGEYTLRVQALHESAAVLDHLHAMPLSLEQKLKKDISLDVYRDHVDLQSHVRPAKEALKLHKGERAVLCIDTALHGERWPSDGVSVHTGDVLCGTLTLGGSSDAKAAKVPLDVVVGLPPPSKSAATAAPNDPQREPASLPTLLAGLVCKVPESDKSAFVEQLVQKYPTDLSVRVAALDACDAEDADASLRAAQGVRECIDETALRLWLGAKQPPTAEQTHEQKATAKTMQTHKAALVNALVREAKAHAQRDPSSQVCTDAIMHARQYVDDSDARLRAMHTNLLTAWHQRNERYGYALQGVRKQLDDLGRGTSDTRDDLRRAHDLQCELLEHLRWDVWRHYETRWAWLRRPSTGAAPF*

>MGL_3247.t1_gene_MGL_3247_AAYY01000011:350270..351937

MRLFQSTCVLVGTVLPLFTAFPISSPREIEIIPDKYIITFKKGIDQAAIEAHTAWVSSVQARNTARGFTTAETPGLERMFSIHNFNAYSGSFDRETIEEIRSHPNVESVEPDSMAYVTELIEQRNATYGPRRISHREIPTGDNSYWYDSKAGEGSFVYIMDTGINKAHVDFEGRAIPGVNLHDVAFDDTHGHGSHCAGIAGSKTYGVAKKATIVDVKVFTRGGGAWSLLMGGLDWSVKNITGEDRQAKSAVSISISGPTNQAMNNAVKAAVEAGVTVVVASGNDGRDAGRNSPGSAPESITVGSINSRRGMDTRSSFSNYGSSVAIHAPGEGIISTYKGSRDATANMSGTSMAAPHIAGLIAYLQSIHDLPDPAAARRKLLELATSDKIQDVRGSANKLAYNGSGK*

>MGL_3817.t1

RTYHYTPARALDVERVGELEGHWLLRKRIRDPLFYKQWHILNEQMPGHDLNIEGAWKLASGKGVTVSLIDDGVEYTHPDIAHAFEPAASYDFNDHTELPWPRLFDDTHGTRCAGEIAAANDVCGVGVAPDAHIAAVRILSAPISDADEAAALNYGYQISDIYSCSWGPSDSGRSMDGPHGLVAKAMLNGIYNGRKGRGSLFVFAGGNGGSLDDQCNFDGYTNSIYTITIAAVDSSGHRPYYSEMCSAIIASAWSSGKNITTSNVCTSVHGGTSAAAPLVAGVLALALEVRPELTWRDAQHLIIQSSVPDPDWQRTTAGYSHKSGFGVVDATRLVENARHKLVPPQSWLEMNTMNVTQAMMNEANASVEHVTVKVWIEHPRRGDVQVSLYGPHGKSVLASPRRYDNDVHGFPGWTFMTLKHWNESPIGTWTIEVSDAWSLTFWGAAKDPKLALLPWLYNAF

>MGYG_00757T0

VVEPEGWFWLAIRENPEKLYDTISTPGRARYGKHLLDDLVRPRAETSESIVSWLDWIKFVKVAEQLMKTQFHVFRTLEYSVPAAISAHVQMIQPTTLFTTACLRELYNRIGVSGFLEEYAQYRDLDLFLFSVGLIGGNTQEANLDMQYVVGLSHKTYYSTAGRNEPLEQLRYLVKLPKDQLPSVLTTSYGDTEQLPYTKATCDLFAQLGTMGVSVIFSSGDTGPGCQTNDGFNPIYPASCPFVTSIGGERASSGGFSDRFPRPQYQAVKGYLLFNPNGRAFPDIAAQGFAVYDKGVSGTSASAPAMAAIIAQLNDFRLAKGSPVLGFLNPWIYFTDIVDGGCSWNATKGWDPVTGFGTPFQAL

>MGYG_01647T0

RDYDYSPEQLLGEGQIGELDGHHTFSKRIRDPLFKEQWHLFNPFTPGNDLNVTGLWLEGITGKGSISAIVDDGLDMYSNDLKDNYFAKGSYDFNEMQEEPRPLLDDDKHGTRCAGEVAAVNNVCGVGVAYDSKVAGIRILSKYINDADEAEAVNYGFQENHIYSCSWGPIDDGMTMDAPGLLVRRAIANGVQKGRGGKGSVFVFAAGNGAGHEDNCNFDGYTNSIFSITVGSVDWNNEHPYYSESCSAQLVVTYSSGSSIHTTDVCSSSHGGTSAAGPLVVGVMALALQVRPELTWRDLQYILVETAVPSEGWQTTSIGFSHDFGYGKVDAYSTVHLAKWKLVKPQAWFHSTSFDISPEMLKEHNERVEHVTVTMNVNHTRRGDLSVELRSPSGVSHLSTTRSKDSERVGYVDWTFMSVAHWGEKGTGVWTVIVKDDWRLTLWGESIDPSIQRKRLRYEFLYDAF

>MGYG_02140T0_|_MGYG_02140_|_Microsporum_gypseum_CBS_118893_alkaline_proteinase_(482_aa)

MQLLNLGLLLLLPFVAGEIAPQPEPLRAGPSDIVPGQYIVTLKEGLASAQIREHKKWVSSVHQANLDSFAAGASGVETVGIMKNFHIHNLNMYSGGFDDKTAEDLRRSPDVKSVHPDQHVYLAKTVTQPQARWGLGYMSSKGKPVPLHSTLVDYLYDDKAGEGVWAYVLDTGINVDHIEFEGRGILGHNAIPNKPHTDEFGHGTYVAGIIAGKTYGVAKKANVVSAKAFDTGSSTYNYILETYDWIVKNITDSNRKNKAVINLSISGAKYQPFDDAVENAFKAGITTVVAAGNDGKDAKNNTPASSPNAITVGAVRWENTRPSFSNYGKIVDIWAPGELIKSCWKGGNNATSTQSGTSAASPHVAGLVAYLMSLENLPSPSAVTARVLNLTIPNLVKDAKDSPNRVVYNGIQERKFTLPKNTK*

>MGYG_02570T0_|_MGYG_02570_|_Microsporum_gypseum_CBS_118893_alkaline_proteinase_(398_aa)

MQFLSLNLLLLLPFVAGDLAPQPEPLRAGPSDVVPGQYIVTLKEGLSSAQIRDHKKWVNSVHRANLDSFAAGASGVETEGIMKHFHIHNLNMYSGGFDEKTAEELSRNPYVKSVHPDQHVYLAKTVTQPQARWGLGYMSSKGKPVPLHSTLVDYLYDDKAGEGVWAYVLDTGINVNHVEFEGRGILGHNAIPNKPHTDEFGHGTYVAGIIAGKTYGVAKKANVVSAKAFDTGSSTYNYILETYDWIVRNITDSNRKNKAVINLSISGAKYQPFDDAVENAFKAGITTVVAAGNDGKDAKNNTPASSPNAITVGAVRWENTRPSFSNYGKIVDIWAPGELIKSCWKGSNTATSTQSGTSAASPHVAGLVAYLMSFENLPSPSAVTARVLNLTIPNLVKDAKDSPNRVVYNGIQERKFTLPKDY*

>MGYG_04371T0_|_MGYG_04371_|_Microsporum_gypseum_CBS_118893_kp43_peptidase._serine_peptidase._merops_family_s08a_(691_aa)

MPVISINGNDLNPENQAPVLRAFGLEAEDASKSNYILIQTKELLEDEQEDELERLGVDILEYVSPKTYLCCYKPTDLAAIRSLPFVAWANVYMDMFVVESSMKTAPTSNTVAGFANAVPKSARRRMVDVILHHDVDATSKELQEELSMATRADISSMNVGAKKIRMMVQNQHLDEVAAIDGVRSIQEVHPAVLFNNKAVPIIKGDADTSGDHPIGTTETKEKASALPYEGEGQIVAIGDTGFDKGSTTDTHPAFKGRVKHLYALGRTEPARSDDPDGHGTHVCGSVLGDGFSEKMGGKIQGTAPKATLVLQSLLDSQNGLGGIPDDLTQLFIQPYKEQGARIHTNSWGSNAPGRQLPYNINSEEIDRFVWEHQDMVILFAAGNAGVDANQDGINDKNQIGAQAAAKNCITVGASENDRPDIVTTYGRWFPNKPYNTDKVADHPNGMAAFSSRGPTKEGRIKPDVVAPGTSILSTRSSKLLKPSTTFGTSNDPDWFFNGGTSMATPLVAGGVALIREALVKNGNKSPSAALIKAMLINGAVELPGQYVPSEAGPSPNSSSGYGRVNLKNSISKPDEPTAGYREAGPLTQGQTDSKLVIKVPKSGSTLKITLVWTDPAGASLQNDLDLIVTASDGSERHGNMGETKEFDRTNNVEQVVWANVPSGEVKVQVRAFHIFKRQFAQRFAVAWSLK*

>MGYG_04593T0_|_MGYG_04593_|_Microsporum_gypseum_CBS_118893_alkaline_proteinase_(403_aa)

MQLLNFGLLLLPFVAGDLAPQPEPLLVGPSDIVPGQYLVTLKEGLTSAQIRDHKKWVSSVHRANLDSFAAGARGVETEGIMKHFHIHDLNMYSGGFDEKRVEDLSRSPYVKSVHPDQHFYLAKTVTQRQARWGLGYMSSKGKPVPLHSTLVDYSYDDKAGEGVWAYVLDTGINVNHVEFEGRAILGHNAIPNKSHTDEFGHGTCVAGIIAGKTYGVAKKANVVSAKAFDTGSMNQSTYNYILETYDWIIRNITDSNRKNKAVINLSISGAKYQPFDDAAERAFKAGITTVVAAGNDGKDAKNNTPASSPNAITVGAVRWENTRPSFSNYGKIVDIWAPGELIKSCWKGGNNATSTQSGTSAASPHVAGLVAYLMSIKNLPSPSAVTARVLNLTIPNLVKDAKDSPNRVVYNGIQERKCKLPKYY*

>MGYG_04715T0_|_MGYG_04715_|_Microsporum_gypseum_CBS_118893_oryzin_(400_aa)

MQLLNFGLLLLPFVAGDLAPQPEPLLAGPSDVVPGQYIVTLKEGLTSAQIRTTTGASGVETEGIMKHFHIHDLNMYSGGFDEKTVEDLSRNPYVKSVHPDQHVYLAKTVTQRQARWGLGYMSSKGKPVPLHSTLVDYSYDDKAGEGVWAYVLDTGINVNHVEFEGRAILGHNAIPNKPHTDEFGHGTYVAGIIAGKTYGVAKKANVVSAKAFDTGSSTYNYILETYDWIVRNITDSNRKNKAVINLSISGAKYQPFDDAVEKAFKAGIATVVAAGNDGKDAKNNTPASSPNAITVGAVRWENTRPSFSNYGKIVDIWAPGELIKSCWKGGNNATSTQSGTSAASPHVAGLVAYLLSTENLPSPSAVT

>MGYG_05000T0_|_MGYG_05000_|_Microsporum_gypseum_CBS_118893_oryzin_(398_aa)

MHFLSLSLLLLPFAAASPLPQENKDIIPGQYIVTLKDGLTAAEIESHRSWVTTMHRSNLAATGLSGIESEGIHGHFQINKLNLYSGGFDKKTVEELKRSPYVKSVLPDQKVYLAETVTQSNAIWNLGHMSNKGKESESWDSLTEYKYDSAAGEGVWAYVLDTGINVNHVEFEGRAILGRNAITNKPHLDTFGHGTYVGGIIAAKTFGVAKKANVVSAKAFDGGSSSYRYIFDAYDWIVKNITDGNRQSKSVINLSICKSSFGIEASSLDVNKHVPLAGSKYQPFDDAIENAFKAGITTVVASGNDGRDASQNTPASAPNAITVGAVRWDNTRPSFSNFGRVVDIFAPGEIIKSCWIGSNSAIRYASGTSAASPHVAGLVAYLMSMETFSSPSAVTARVLGLTIPDVVKDARGSANKLAYNGIQERR*

>MGYG_05064T0_|_MGYG_05064_|_Microsporum_gypseum_CBS_118893_proteinase_T_(517_aa)

MQLLNLSLFFLLPFATANPIPQDSQDIIPGQYIVTLKDGLTTAEIDAHKTWLASTHRSNIAAKGHSGIESEGVFKHFQIHKLNMYAAGLDKKTVEELRRSPHVKSVLPDQKIYLAEAVTQSNAGWNLGYMSSKGQPSPSWSTLTNYTYDSTAGEGVWAYVLDTGVNVNHVEFEGRAILGRNSIPNRPHEDTFGHGTYVGGIIAGKTYGVAKKATVVSAKAFDGGSSSYRYILDSYEWIVKNITDSDRKSKSVINLSISGAKYQPFDDAIENVFQAGITTVVASGNDGRDASQNTPASSPNAITVGALRWENTRPGFSNYGKVVDLFAPGELIRSGWTGGNNATRVASGTSAASPHVAGLVAYLMSIETLSSPSEVTARVLNLTIPGLVKDARDSPNKVAYNGIQEML*

>MGYG_06500T0

VVEPQGWFKLALQSKTAEFEQRVSNPRHADYGKHMVDAFLQPSSLAKESVLNWLDWLTFIENAEKLFDTHFYTFRTLKYSVPASAAPYIQMIQPTTKFTPDCIRDLYNRLGISGYLEQYARLEDFSTFIFDFKSIGGNEQEASLDVDYAIGLSGTYYGTAGRNEPIEQLFYLLDLPDSELPAVLSTSYGENEQIPYTSVVCSLFGRLGARGVSVIFSSGDTGVGCQSNDGFNPIFPAACPFVTSVGGEVASSGGFSERFARPWYQDVRHYLLYNPSGRGFPDVAAQSFATRDHGVSGTSASAPLFAAVVSILNSIRLAHNKPKMGFLNPWLYFTDIVHGGCSWNATKGWDPVTGLGTPFEKL

>MGYG_06576T0_|_MGYG_06576_|_Microsporum_gypseum_CBS_118893_oryzin_(423_aa)

MQLLNLSLFFLLPFATAHPIPQDSQDIIPGQYIVTLKDGLTTAEIDAHKTWLASTHRSNIAARGHSGIESEGVFKHFQIHKLNMYAAGLDKKTVEELRRSPHVKSVLPDQKIYLAEAVTQSNAGWNLGYMSSKGQPSPSWSTLTNYTYDSTAGEGVWAYVLDTGVNVNHVEFEGRAILGRNSIPNRPHEDTFGHGTYVGGIIAGKTYGVAKKATVVSAKAFDGGSSSYRYILDSYDPPWNTTFQSLYAKKNVAQAGAKYQPFDDAIENAFQAGITTVVASGNDGRDASQNTPASSPNAITVGALRWENTRPGFSNYGKVVDLFAPGELIRSGWTGGNNATRVASGTSAASPHVAGLVAYLMSIETLSSPSEVTARVLNLTIPGLVKDARGSPNKVAYNGIQEML*

>MGYG_07290T0_|_MGYG_07290_|_Microsporum_gypseum_CBS_118893_alkaline_proteinase_(397_aa)

MQLLRSSLLLLLPFVVANPIPQEDSDIIPGQYIITLKDGLSQADVESHKTWVSSVHRSNLAATGRQGIQSGGIAKAFQIHDLNVYSGNFDEQTAEDIRRSPYVKSVTPDRKVYLAETVTQEDAIWNLGHMSSKGQPSTTYKYDSAAGEGVWAYVLDTGIHISHEEFEGRAILGYNAVKNTPHEDRNGHVAKKATVVSAKAFDTGSSSYTYIFDAYNWIVKNITESRRQTKSVVNMSISSAKYQPFDDAVERAYRAGVVTVAAAGNDGRDASRNTPASARNAITVGAFRADNTRSTFSNYGRVVDIFAPGELIKSSWPSPTNNLTNIASGTSAAAPHVVGLVAYLMSLETFTSPAAVARRVIQLAIPNLVKNPGTGSPNRLAYNGIQERR*

>MGYG_07342T0_|_MGYG_07342_|_Microsporum_gypseum_CBS_118893_proteinase_R_(401_aa)

MQLLRTSLLLLLPFVAASPVPSDDKDIIPGKYIVTLKDGITQADMDSHKAWVANVHQSNLAAAASAGRTGGESGGIRKIFQINSMNAYSGAFDEQTAEDIKRHPNVKSVYPDRKAYLAETVTQNNAQWNLGHMSSKGRRSFTYRYDSKAGEGVWAYVLDTGIMTDHVEFEGRAILGYNAVPDVPHVDNVGHGTYVGGIIGAKTWGVAKKATVVSAKAFHGTSGSYEHIFDAFNWIVKNITESGRQKQSVINMSITSAKYQPFDDAVEEAFKAGVSVVVASGNNGRDGTNNTPASAPNAITVGSVRFDNTRSYFSNYGRVVDIYAPGERITSCWMGGRNATRKSDGTSVASPHVAGLVAYLMSIEDLPTPGAVTKRVLELSIPDLVKDPGANTANRLAYNGIRERS*

>MGYG_07924T0_|_MGYG_07924_|_Microsporum_gypseum_CBS_118893_proteinase_K_(409_aa)

MNAYSGAFDDQTAEDIRRNPYVKSVTPDRKVYLADTVVQENAGYNLGHMSSKGRHSFTYRYDSTAGEGIWAYVLDTGINVDHIEFEGRADSGYNAIKNVSNTDNFGHGSFTAGIIAAKTYGVAKKATVISAKAFDTGSSTYDYIFDAYNWVVKNITDSGRQKKSVVNMSISSAKYQPFDDAVDNAFEAGITTVVAAGNDGRDASNNTPASAANAITVASIRFDNGRSLFSNYGSVVDIFAPGERIVSCWIGGNDATRKADGTSVSSPHVAGLVAYLMAIEDLPDPAAVTKRVLDLSIPDLVRDPGEGSPNRIAYNECNSNMYVCALGNTPRAART*

>MGYG_08213T0_|_MGYG_08213_|_Microsporum_gypseum_CBS_118893_alkaline_proteinase_(401_aa)

MKLLKSSLLLLLPFVTANPIPSEDKDIIPGRYIVTLKDGITQEDIEYHKSWVASVHRSNLAAATAAGRPRLETEGIRKFFQIHKMNAYSGAFDDQTAEDIRRNPYVKSVTPDRKVYLADTVVQENAGYNLGHMSSKGRHSFTYRYDSTAGEGIWAYVLDTGINVDHIEFEGRADSGYNAIKNVSNTDNFGHGSFTAGIIAAKTYGVAKKATVISAKAFDTGSSTYDYIFDAYNWVVKNITDSGRQKKSVVNMSISSAKYQPFDDAVDNAFEAGITTVVAAGNDQRDASNNTPASAANAITVASIRFDNGRSLFSNYGSVVDIFAPGERIVSCWIGGNNATRKADGTSVSSPHVAGLVAYLMAIEDLPDPAAVTKRVLDLSIPDLVRDPGEGSPNRIAYNGIQEMNETVIA*

>NCU00263.t1

YIVFKGYPVHLMTVNKFRGITGKGIKIAVIDTGDYLHPALGGCFGGCLVSYGTDLVPDPMDTCHGSHVLGLLSGAAPDVTLGAYRVFGCDILIAYLKAYDDGSDIITASIGAAVVSRIVVPCLVSAGNGATGFYASTAANGKRVTAVASVDNNGCVLIRGASTYTSWGPTVDVKPQISSPGGILSTYPGTSMACPAATWALAHPAPVAQQGAGLIQAWDAALLSNDTVTNGKKAVTYQLGHTSATLNPGLPVYSGYIPYQGGALPADTYKITLKALKIYGDW

>NCU03219.t1

RDFDYSPARSLGEGPLGELQDHHLFVKRIRDPIFKEQWHLFNTVQTGHDVNVTGLWLEGVTGKNATVAIVDDGLDMETDDLKDNYYAQGSWDFNDKGPDPKPRLSDDKHGTRCAGEVSAGNKACGVGVAYDSRIAGLRILSKLISDADEAVAMNYDFQHNQIYSCSWGPPDDGQSMDAPGILIKRAMLNAVQKGRGGLGSIYVFASGNGAGNGDNCNFDGYTNSIYSITVGAVDRNGDHPYYSESCSANLVVTYSSGGGIHTTDVCSDTHGGTSAAAPLAAGIFALVLQVRPDLSWRDMQYLTVNTAVPSGEWQTTAIGFSHMYGYGKLDSYAIVQAAKWKKVKPQAWFYSVSYEVTQAMLDEANERLEHITVTMNIMHTRRGDLSVDLISPNNVSHLSVSRKNDEARAGYDDWTFMSVVHWGETGVGNWTIIVKDDWHLKLWGESKDASKARKRLRYEFLYDAF

>NCU04903.t1

VLEPAGWLSIALEPGIEELKRRLSTSDDHPNSRQFVEKHRQPDQRSVTAVGRWLSWITFAATVQMLFEADLAYYRSRSYTIPRWLSDDIDFVHPLTNFFPGCIRKLYVRFGIASFLEQYITHRDVTSFLITITLLNADPHEANLDVQYALSLGHPIYYATGGRNEPLEFLQALLALPDNQIPHVLSISYADDEQVPYAHRVCDLFAAVAARGTSVLVATGDGGAACIKNDGFVPTFPASCPWVTSVGALTGSSGGFSEYFDRPLWQAVDPYVVYSHNGRGMPDMAAIGFQIIHRGVRGTSASTPVVAAMVALVNDQRLRQGKRSLGWLNGHLYLTDVKWGGCGWDARKGWDPVTGLGVPFQEM

>NCU06055.t1_gene_NCU06055_Ncra_OR74A_chrVII_contig7.21:complement(120173..121474)

MHIFRNLYFLGLLVPLVSSIPIDQSQTQASNKVERDYIIVLKNNISKRELLSHTVWAREINAKFLRKRQDSGLVQGIEPAGVKKVFDIHKFKAYSGTFDSETLNEINKHKQVDYIEKASPIFLNEFKTDLTDEWGLSGISHKVPQFPTELNGTYRYDDSAGEGMFAYVIDSGVNIAHKDFEGRAEIGYDALHPDSKTHNDTHGHGTHTAGTIASKTYGVAKKARVISVKVIDRVEYKRNTSADLLDGINWAANDIVKKNRQNFAVINLSMAISYSRAINDAIDNAYKLGVLSIVAAGNNNMNVKEENSSPTMASKAFVVGAIGANNRRWIDNDRIGSNYGDMVDIMAPGENIMSLGIGNNIDATSLKSGTSMAAPHVAGLACYLRRLEGLKSPEEVTSRLLQLAQKDVLDGASLKGSPNLLAYNGNGL

>NCU06949.t1_gene_NCU06949_Ncra_OR74A_chrVII_contig7.32:join(21904..2220922317..2271222809..23372)

MHLLSLATLLPLVGFFLQASADLQLLNANSKQRSPNSYIVVLKPSTNRTQAEEHTHRITTYHRARSLDERAGTTGIGGSFNIDGTGPGFKGYNVHCDGRTLREILRSPEVQYVEADTKTKSDLIQSGSTWGLARISWRRLPTTYSYRYASAWNGIGITAYVVDSGVRITHQQFEGRARWGYNAVPRSSNTDKFGHGTHVAGTIAGKTYGVAKKAKIVAVKALDDKGDGFHSYTIACLNWIGRNAKAGKSVVNLSLGGEKSKAVNDAVEALYKKGIVVVVSAGNKNNFASLYSPASAPNAITVAASTSGDERAKFSNYGTSVDIFAPGQNVLSAWYTSNTALKYRDGTSMAAPHVAGIAAYLLSSKASYQSPLTIRNRIVTLSRKGYISKAGTGTVNRLAWNGYLSN

>NCU07159.t1_gene_NCU07159_Ncra_OR74A_chrV_contig7.37:join(305078..305359305426..306334)

MHFIPLASLLPLAGLILQVAADLTVLGAGSKDKIPNSYIVVLKPSTNQAQVQEHTQRISNYHTRRSLQERGVTTGIREQFDIQSVKGYTVECDHGTLSQILTSPEVQYVEQEGKTKVQVTQKPSTWGLSRISWKTLPKAPYSYRYQNTWGGRGTTIYIVDSGVRISHKEFEARATWGYNAVPDSPNTDNHGHGTHVAGTAAGKTYGVAKYARIVAVKVIGDDGTGQDSYTLAGLNYISKVAKPGKSVVNMSIGGPKSEAVNAAVEALYKKGIVVVAAAGNENENAGLSSPASARSAITVGATDETDTRARFSNFGSIVDIFAPGVNILSADITSDTASRLDNGTSMASPHVAGLAAYFISSRTTSQSPLNIHSLFITYSQKGLVKSPGPSPNRLAYNGWDEYQPYPY

>NCU08418.t1

VKSPSGWLKIALAHQPDALETAISDPNHHEYGMHLVRSLVAPADETTDAVTSWLDWVSFVAKANNLLNTTFDWYRTLQYSVPDELDAHVDMIQPTTRFYPDEIRSLYNTIAFASYLEQYSNYDDFTSFAYTVKLVGGHDQEANLDLQYILAISNPREYSIGGRNEPLDFFQYLLSLKNSELPATLSTSYGEEEQVPYALKVCSMIGQLGARGVSVIFSSGDSGPGCIRNDGFEPTFPGACPWVTSVGGEKASSGGFSMYHKRPVYQVVKKYLFFDEQGRGFPDVSAQAYAVYVDGVSGTSASAPMFAGLVALLNAARKSHGLPSLGFINPLLYFTDIVNGGCKWNATEGWDPVTGLGTPFDKL

>NCU08734.t1_gene_NCU08734_Ncra_OR74A_chrII_contig7.57:complement(join(118346..118984119071..119427119709..120214120307..120598))

MSATSSRPKTKILQSQPPSDAAGLAELENRPPRLPDGPQENEPVAKSLTTKERKSPETSKARAIAAKRVEQLLKLHYLRTRDEQTCIDILFHDEITPDFGDQVLNCDLSGMTHMTEEEFQRLMSTLKFHDVLQYVELPGLRIGTPSTTDLDGVNENEQKGRKNLAWVFEKLRSNGVQKVLRVSVDDTLLPPHSDEVIEDSLKPMEVEIWDWKKIDLCSEVIFNAAPMVREVYLYCSGNNAILRSWSDEGGLKRLSHLKRVYVDVKMEFRKLLIDAENNYDTGTTKKVWEMIETPIKVALIDDGVDIMKLDLTPAQCLGGRTFCPRSGPHDMCKNHPHYISSAGHGTIMAEAILEVCPRASLLVLKLEDLPSKNGNREITTQSAVKAIKKAVQKRVDIISMSWTIDVDESDPAKNDLEEAITSASKLGILMFCSAKDNGADNRQTYPAKAASEKIFKIGAALESGVADEWVGDLRLLDFTFPGSKKEYNGMTIFGSSIATAYAAGLVALILYCVQVRLFLATSEADKMQARKDFEALKKHENMSNVLGDTIGTTPMSQHKFVMVWNMFLPAVQNRDGKPDKLLQLIAEVGRKLCARVE*

>NCU10306.t1

VKSPSGWLKIALAHQPDALETAISDPNHHEYGMHLVRSLVAPADETTDAVTSWLDWVSFVAKANNLLNTTFDWYRTLQYSVPDELDAHVDMIQPTTRFYPDEIRSLYNTIAFASYLEQYSNYDDFTSFAYTVKLVGGHDQEANLDLQYILAISNPREYSIGGRNEPLDFFQYLLSLKNSELPATLSTSYGEEEQVPYALKVCSMIGQLGARGVSVIFSSGDSGPGCIRNDGFEPTFPGACPWVTSVGGEKASSGGFSMYHKRPVYQVVKKYLFFDEQGRGFPDVSAQAYAVYVDGVSGTSASAPMFAGLVALLNAARKSHGLPSLGFINPLLYFTDIVNGGCKWNATEGWDPVTGLGTPFDKL

>NFIA_001580.t1_gene_NFIA_001580_DS027697:complement(join(297897..298972299047..299435299542..300050300090..300285300390..300461300550..300570300621..300664))

MVAILFLSFPAGPGSNPGIYPEGGPPPIGPNPPPGLTLKIPWPQITIGPDRKPTYPDKPDPEEDICETATASVCTTTLSYGIVAKRAAEGAAPTVAPRIPSEGFQKLNERAVTGCGASDITSTTSIQGTATTNPRVVIPRDPGSVNGIRTTLQQQLGGSALDLFESRTDQLGTMFFFVPAFTDDQTAAIWGHAQVADAYIPWGPLTAAYWGLASDPTSGGGPPAGDDDWFMETQNSTKSELQERSILAKRSEIVQSNMPNVMALLSWPPGMSPVPYEGDYRFDSSAGEGTYVYDLNYGAQPSHPEFSDVLFHLPLLPGPYPVSGWMENDPKRHRTMCLSKVVGKTVGIARKATVVATVWDFKKSIFDHWLDGLTKVHADISNGARGAKSVINISVSMPQNRVSAAFVDKMALLIREIIKLGAAVITGSGNSEGNPNGYPALFGDPANRNYIPELVVVGAVLGSGILSDYSNAPWVTCYAPGISVEMATSEPSLYRTAHGTSYASAAVAGLAAYFRGLDPTLTTAARVKQRIVDLAYRRPAIPNYPNERYPDKVVWNGQMNGRSVAGDCSGSSKTKRQSRGGSCPVAFPPQASPLTFRTGPAQPTCAGTGCGSSCAGFFCPGALLKQNPDFLDPRNPDSVQNPDSPYYKDWEGTITRTTTTTSTTKTTPTPTPTPTPTPTEIPGVPIGGTCTSNAVCEGDCPKGRLLGCLNGVCNCYIDQNIPPYGTLCFEVQGCLNVYYCALGDTMVCEERDYSNGEKVCLCIKGSSS*

>NFIA_025920.t1_gene_NFIA_025920_DS027695:join(444078..444308444367..444924444976..445072445133..445222445283..445470)

MMRHFLLLLSFVLSIVNGDPYLMPNQDLLSQSSLISVHLHNNHSVKPHSIERFSTILNAYIVVFKPETDQETALTHYNEIEEIQQSDISHTNQNLDYGIIHKYEIGSFKGYSGKFSSSVISTIQTYEEVDYIEQDKHVQAACIQNQAPWGLARISHKEALTDNTFNKYLYNKHAGKHVTVYIMDSGIYEEHEDFGGRASFGVNFLKGSPDNDTNGHGTHSAGIIGGKRYGVAKKAKLVSVKILDENATGSVSNAIAGIDWIVKCHEFDIQKSLLKGNTYKGAVATLNFITTSTKALNKAVIEGIYGGVTIIVPAGNDGQSACDYSPPSVTAAIKVGSSTYYDKKSLFSNNGNCVNIYAPGQSIISAGNSHKNSKRVLSGTSEAASHVAGLAAYFISMREGQNTPEYIKSKIMDIATPDVILELDPYGRSTLKPIAFNGFKL*

>NFIA_029950.t1

VFEPQGWLQIALQHDVESFETALSDPYHPNYGKHFMKRMLLPTQEAVESVRGWLDWVKFVGVANDLLDADFKWYRTLAYSLPQSVASHVNMVQPTTRFTPQCLKDLYSKVAFASFLEEYARYDDLAKFEFSVIQYGGNDQEANLDLQYIVGVSSPTEFSTGGRNEPLEFLQNVLKMDQDELPQVISTSYGEDEQIPYARSVCNLYAQLGSRGVSVIFSSGDSGVGCLTNDGFPPQFPAACPWVTSVGGEEASSGGFSDLWERPSWQAVKRYLLYNPKGRAFPDVAAQAYAVFDKGFDGTSCSAPTFSAIVALLNDARLRAHKPVMGFLNPWLYFNDIVNGGCSWNATDGWDPVTGLGTPFGKL

>NFIA_031000.t1_gene_NFIA_031000_DS027693:join(754988..755071755135..756471756572..757747757821..757892757997..758737758843..759231759304..760382)

MKLLAALASALAWGVSLAEAKAVFAHYMVGNTKSLGLIDWRHEMQAAQAAGIDAFVLNMASKDPTNNIALPMAFTAADDMGFQLLFSFDYAGNGPWDKSIVIDMIKEYGAKDTYFKTAGKPFVSTFEGPNNADDWKDIKKETNCFFMPDWSSVGAQPAVHLGDGIADGLFSWDAWPKGPANMTTYPDASYYDFLGSKPYMMPISPWFYTNLPGYGKNWLWRGDDMWFQRWQQAISLDRQPDFIEIISWNDYGESHYIGPLDDRQYEAFDIGRAPFNYVKDMPHDGWRETLPYYISMYKSGTATVTEERLVAWYRVNKNGACSDGGTTGNTANQLQFEYSPNVMMEDRVFYDVLLTSNAQVQVSIGGVVQAGGWDQEPYGGVGVYHGSVPIGMASGQVVVTVKRGGTTIATITGASITSSCNGGLNNYNPWVGSARGAPIIPVTTTGDLSKLDCVKGFGVFDFIGVCDFACANGYCPSAACTCLKKGVANAPNETGLAGYPLPGKSGSFAGLCSFDCNHGYCPDSVCGQTPDDGVVLGYSPFLPPACTGGTGAGAFQGLCDFGCHLGFCPIHACTCTSTGILVQTPPKTNVTGYYLDSSTDDYGLCKFACEHGYCPDVCGSRPIGDDGNQGKYPTITLDPAVWTAPTAQCAPPCVLVLPPSSLASPTTISFDPWQTSLEFGWMTTDTVDGTVTTHYTAVTVSTEISIPPVITDLISFSEVILTTTVDGGVPSIIIPTASVSPPPFVITPTPVADITAAPVARTIRPPPWPWSGASAMPDPSGTSTTTPTSGPVVVVPIVTGPFPTTVFPTETASWVRDWLPEPTATQVDDGDPVPVVPCWAWFIWSCPPNVGGIVLPGFKNPGIYPEGGPPPVGPNPPPGLTLKIPWPQITIGPDRKPTYPDKPDPEEVSCETATASVCTTTLSYGIVAKRAAEGAAPTRAPRIPFEEYRKLTKRAVTTTTSTISFCTQVTGCGATDITTTTAIATTATPIPRVVIPHDPWSVDGIRTALQQQLGGSALDLFESRTDQLGTMFFFVPAFTNDQTDAIKGHAQVADAYIPQGQLISYLGMARDPTSGGGQPASDDDWFMDTLNSTESELQERSILAKRSEIVQSNLPDVMVSLSWPYGIGPVPDQGDYRFDSSAGEGTYVYHVDYGAQPSHPEFSGVSFLHPLLPGPYPVSGWMENDRKRHGTKCLSKAVGKTVGIARKATVVATVWDFTKMIFEHYLDGLAKVHADISTGARGAKSVVNFSISFPQGLVSDAFVDKLALLIREIIKLGAVFVTGSGNEAGSPNGYPALFGDPDNRNYIPELIVVGSVTGWGFLGGHADALWVTCYAPGFFLRLATSGPTDSRDPGYQSTLAGTSYASATVAGLAAYFRGLDDTLTTAAMVKERIVRLAYRRQPTLAYPDEDYPDNVVWNGQKWGRSIVRDCSGGSKAKRQSNGGSCPVAFPPQPSPLTFRTGPPQPTCAGAGCGSSCAGFFCPGTPLKQNPDFLDPRNPDSVQNPDSPYYEDWDGTITRTTTPTKTIPTTTPTPPKISSVPIGGPCRLTDECEDNCPKPGAVQCQSGACTCWPPPPKTTPPHAAMCYDVQQCLDVYDCGSGAFMVCEPTDYSNGNGLCQCIKGNSS*

>NFIA_068200.t1

VVEPDGWFRLAMQERAAEFERRVSTPGHSSYGQHMIREFLRPSEEVSDRVLSWLNWVTFVSQAERTLAYSVPDDVHRYIQMIQPTTRFTPNCLRELYNRLGVSGFLDQYARYDDFENFMFTVVSIDGNLQEASLDVQYAYSLAYKTYYTTGGRNEPLDQLHYLLDLSDEKLPAVLSTSYGEDEQVPYSNATCNLFAQLGARGVSIIFSSGDSGVGCITNDGFLPVFPASCPFVTAVGGEKASSGGFSDRFPRPSYQSVQGYLLYNPSGRGFPDVAAQAFVVIDHGVGGTSASAPVFAAIVSRLNAARLEDGLPKLGFLNPWLYFTDIVDGGCSWNATPGWDPVTGLGTPYNTL

>NFIA_078120.t1_gene_NFIA_078120_DS027696:join(1587553..15879211587989..15889271588992..1589171)

MANRETHSITDQDFSSQTPLLSKHPSEAASVTLDPIRQFLSIPDSYLIIFKQGTKQETVLSHHDEIEEIQRIEKFLTSRDLDFGIIHKYEIGNFSGYSGKFSSSVIRIIQTYEEVDYVEQDKYIEAASIQNRAPWGLARISHREVLNSVTFDKYMYEKNGGKYVTVYVLDTGIYDKHNDFDGRASFGVNLLNCSSEEDTNGHGTHSAGIIGGKVYGVAKRAKLVSVKILDEDGIGTLSNAIAGINWVFKNHAFGVRRSLSRKWIYKGAVATLNFITTKSTALNQAVDEGISIVVPAGNNMGNACNYSPSSAKNCITVSASTYYDHDTRPSNFGECVDIYAPGQNILSAWNTDMNSKKVLSGTSTAASHVAGLAAYYTSMVEGQSTPEYIKRKIIYTSTPDIIRVDLVSRSYFEPLAFYETNT*

>NFIA_103380.t1

RSYEFSPAQLLGEGQIGELANHHTFSKRIADPIFNGQWHLFNTVQLGHDLNVTGVWMEGITGKGVTTAVVDDGLDMYSNDLKPNYFPEGSYDFNDNTPEPRPRLSDDKHGTRCAGEIAAANDVCGVGVAYDSRVAGVRILSKAINDADEATAINFAYQENDIFSCSWGPPDDGATMEGPGILIKRAFVNGVQNGRGGKGSIFVFAAGNGASFEDNCNFDGYTNSIYSITVGAIDREGKHPSYSESCSAQLVVAYSSGSGIHTTDVCYSFHGGTSAAGPLAAGTVALALSARPELTWRDAQYLMVETAVPDGSWQVTKAGFSHDWGYGKVDAYALVQKAKWELVKPQAWFHSSSYEVTEQMMKNANARLEHVTVTMNVNHTRRGDLSVELRSPEGVSHLSTTRKSDNENAGYVDWTFMTVAHWGESGVGRWTVIVKDDWRLNLWGEAIDGANQRKRILYDFLYNAF

>NFIA_104430.t1_gene_NFIA_104430_DS027685:join(846125..846448846506..846950847017..847105847177..847530)

MEQFSIVPDAYIIVFKPETKQETALSHYNEIEDIQQSEISHTNQNLDYGVIHKYEIGGFKGYSGKFSSSVIRIIQTYEEVDYIEQDKYVQAASIQNKAPWGLARISHRKRLNNMTFDKFIYEDDGGEYVTVYILDTGIYEEHEDFEDRASWGVNFLRNSPDIDIDGHGTHSAGIIGGKIHGVAKMAKLVAVKVLDDHGTGTVSTVIAGINWVSKSHQYNIQQSCLHPLDSIYKGAVATLNFFTTKSVALNQAVNIGTYHGITIVVPAGNTKVNACEYSPASATEAITVGASTYYDAGTSFSNFGDCVDIYAPGQNILSAWNSGRHAKKVHSGTSEAASHVAGLAAYYASMRKGQSTPEYIKETISLTSTNKTITVAYPSGLHSAEPLAFYDIN*

>orf19.2242_gene_orf19.2242.gene_Ca21chr2:complement(1413957..1415354)

MKTSTVLAGLLGSALPALAALELRIQDKAPWNLRAISHRSPHRAPKVTRLRNFKYYYSWNDETPYYAYVIDSGIRVSHNEFENRAENLWTAFKTSDKKDNFQNGNGHGTHVAGIIASKTYGVAKKARVVSVKVLNNEGRGDLSQAIAGFDAAIRDIHKNRRYHHAVINISAGWTTCSKALATAIDRAYNTRGILTIVAGNEGPGTVQCTPTDSTHSITVGAMAPDWSAAPFTNLRYKVDIFAPGVDILSLAKDSDSATTTKSGTSMAAPHVAALALNAMSVFSKQGQEVTSFLQKTATKNKVKGDLGGSPNLLVNNNNDKQNSCKAQPESDDSC

>orf19.4755

RDYNYSQIDFISEHQLSSLDNHYVFSKRIHDPEFTTQWHLINLKYPGHDVNVTGLWLENILGQGIVTALVDDGVDAESDDIKQNFNSEGSWDFNNKGKSPLPRLFDDYHGTRCAGEIAAVNDVCGIGVAWKSQVSGIRILSGPITSSDEAEAMVYGLDTNDIYSCSWGPTDNGKVLSEPDVIVKKAMIKGIQEGRDKKGAIYVFASGNGGRFGDSCNFDGYTNSIYSITVGAIDYKGLHPQYSEACSAVMVVTYSSGSGIHTTDICSATHGGTSAAAPLASGIYSLILSANPNLTWRDVQYISVLSATPDGNYQTTALNYSHKYGYGKTDAYKMVHFAKWVNVKPQAWYYSSSVNVSEKDLKIMNERVEHITVKVNIDSTYRGRVGMRIISPTGISDLATFRVNDASTRGFQNWTFMSVAHWGETGIGEWKVEVDWQFRIFGESIDGDKARRYEFLFDDF

>orf19.7196_gene_orf19.7196.gene_Ca21chr7:853163..854644

MKGSTALAVLLGLASASPTPTAEVQTQHKAPWNLRTISHRSSPRFPMVNFFRNFKYYYQSWPSDKTYYAYVVDSGVRISHKEFEGRAENLWTALKTRDGKDDFEDKSGHGTHVAGTIAAKTYGVAKTARVVSVRVLDKEDRAPTSTIIKGLEQAISDIAKKNRHNNAVINMSVGAECSTAMNTIIQRAYKRRDASGKGLASILVVAASGNEGADASTCSPASSNDALTVGAIDSSWNVVKWSNYGRKVDILAPGDGVTSLSSKSDYGTETMSGTSMAAPHVAALALNAMAVFSKLSNEVKFYLGQTATKDMIKGDLRGAPNLLVNNNNNEQESCERRSQPKNENEDDAC

>orf19.7463_gene_orf19.7463.gene_Ca21chrR:164523..165788

MKISILLAAFSCLVSAAPPAKPKTAAQSKAPWNLQAISHRSAPTRLNMFRNSDYLYTPWPKDKTLYAYVLDTGIRTTHQEFEGRAENFWTAFKTADNQDDFDDESGHGTHVAGIIAAKTYGVAKQARVLSVKVFGPNGQVLTSQAILGFTFAMNDIIKKGRQNSAVINYSGGRKFSMAWNTIVERAFNRPNGPILTITSAGNDAKDAAGASPACADEAITVGSIRSDWSVAPSSNFGCKVNILAPGGKILSLSNTSDVATKTLSGTSMAAPHVAALALNAMAVFGKSSKDVLEFLTQTATKDKVKGDLKGSPNLLANNNNPRQRA

>orf19.7464_gene_orf19.7464.gene_Ca21chrR:163022..164161

MKASILLAAFSCLVSAAPPAKTKTAAQSKAPWNLQAISHRSAPTRLNMFRNSDYLYTPWPKDKTLYAYVLDTGIRTTHQEFEGRAENFWTAFKTADNQDDFEDQSGHGTHVAGIIAAKTYGVAKQARVLSVKVFGPNGQVLTSQAILGFTFAMNDIIKKGRQNSAVINYSGGRKYSMAWNTIVERAFNRPNGPILTITSAGNDAKDAAGASPACADEAITVGSIRSDWSVAPSSNFGCKVNILAPGGKIVSLSNTSDVATKTLSGTSMAAPHVAALALNAMAVFGKSSKDVLEFLTQTATKDKVKGDLKGSPNLLANNNNPRQRA

>ORFP:Scas_Contig657.11_YEL060C_Contig_c657_1983722068_reverse_complement

MKTSTALAVLFGLALAAPEGPKATVQNKAPWNLRAISHRFPHKLPSVTVFRNFQYYYDSWTNGKTYYAYVVDTGIRTTHQEFEGRAENLWTAVKTATGEDDFSDGTGHGTHVAGIIASKTYGAAKQARVLSVKVFDDKNDATTSQILAGFNHAANDIADKGRKNTAVINCSLGAASSPALKLVYERAHHRGILTVTSAGNNAQSVGAASGGSASGSITVGSINQDWSIASHSNYGSHVTIFAPGADILSLSHKSDSATAIMSGTSMAAPHVAAVVLNAMAAYSQESSLVDFFLETTATRDKITGDLRGSPNVLVNNNNDRQESSCGQQDDRC

>ORFP:Scas_Contig659.19__Contig_c659_2807929434_reverse_complement

MLFKPVSSMAKLSLLLLAVVSPFTVMAAPSLHKAAGTSVPNQYIILFKDHMFEESVEKHFEWIKNLIKPYLPAAPIFSFQGDEDRLFDRPEPKVSTLGMMDELFGVMHRYHMDGFQGYSARLPQFVASLLKEHGDIAHIEEDKVMTIFDTQPKAPSWGLTRISFRDHPDASQKVYSYPSSAGTGVNAYIIDTGVFIKHPEFEGRAHLGKSFTKDGNNDGNGHGTHVAGTIGSKSYGVAKNVTLIAVKVLDNQGSGTTSDVIAGIDWASKDAAAKNGKDKPLKSVANMSLGGGASAALDKAVKAATKNGLVFAVAAGNSGRDACSLSPARVPEAITVAASDKNDNLASFSERGKCVDVIAPGVDITSTWNNGKINTISGTSMATPHVVGVVALALAEGSFTQVKAVHDYIKLVASKDRISGDLRGAPNSLLYNNVIDGGFPDDEPKEPEPQPEPEPIPPNEGECPIPQCLFDPECTSCCVDCLWAAFRV*

>ORFP:Scas_Contig720.75_YCR045C_Contig_c720_172010173488_reverse_complement

MRINFLAKAFFIAQAFALGSAVNPLRQFFPDDRSGAGIHPVDSLQNSGSVNAEFAAQNYIIVFKSHVADAEIEKHENWLQSMHQKRSIDWHDVTNFVIKHTFNIGDLFRGYAGKLAPWLVRELQKHPDVLLVEPDQVMHVKEERTLAPWGLARISHKEKLNLVTFTRYQYDESAGEGVTAYVVDTGINTEHRDFGGRAVWGITIPTGEPDEDQNGHGTHVAGTIAGNTFGVSNKAKVVAVKVLNAEGSGLVSDIIKGIEWVYKASKNDTESISSVVNMSLGGDASVALDTAVSAAIQQGLFFALAAGNEAQDACQVSPARVSTAMTVGAMTWKDEVASFSNIGSCVDVFAPGHYVLSDWIGSDSAFMLLSGTSMASPHIAGLAAYFTALNHSLAYNPAALKAHILSAATEGLLDNVPSDTPNLLAYNQYEA*

>ORFP:Sklu_Contig2020.3_YCR045C_Contig_c2020_53886827_reverse_complement

MKVSFAAGLVFAAQLCSVSTAFNPLRFFLDDNSFSPNVHGPVPIGDDIAAPPLNHPSQKPYETDVEGSSSITNDHYIVMFKPSVDKAKINSHHEWIQHQHHKRSLDWQDVSTFLLKHTFEIGDSFMGYAARFSPWLVNELKKHPDIAIVEPDRVMHVTAEQRFAPWGLARVSHRDRLGLSTFTRYNYNETAGEGVTAYVIDTGVNVNHQDFEGRASWGATIPNGEKDIDNHGHGTHVAGTIAGKTFGVAKNSKIVAVKVMRADGTGTTSDIIKGIEFAFKESKKDEDSVASVANMSLGGDASLALDLAVSAAIKGGLFFAVAAGNDAEDACNTSPSRVSSAMTVGAMTWTDGISSFSNHGSCVDIFAPGSLILSDWIGSDKASMLLSGTSMASPHVAGLAAYYISLEPSLANKPADLKKYMLKYALTDKLEGIPEGTPNVLAFNNFSE*

>ORFP:Skud_Contig1526.4_YCR045C_Contig_c1526_39385560

MKLSFAAGLVFAAQLCSISTAFNPLRLFMDDDSFSPNVHAPVSSGNGIAPPSFNHPAQNPHDVDVEGSSLVTNDHYIVMFKPSVDKAKINSHHEWIQHQHHKRSLDWQDVSTFFLKHTFEIGDSFMGYAARFSPWLVNELKKHPDIALVEPDHVMHITTEQRFAPWGLARISHRDRLGLTTFTRYNYNETAGEGVTAYVIDTGINVDHQDFGGRATWGATIPNGEKDVDNHGHGTHVAGTIAGKNFGISKNAKLVAVKVMRADGTGSTSDIIKGIEFAFKQSKNDNDSVASVANMSLGGDASQALDSAVSAAINGGLFFAVAAGNDAEDACNTSPSRVSSAMTVGSMTWTDGISSFSNHGSCVDIFAPGSLILSDWIGSNKASMLLSGTSMASPHVAGLAAYFISLEPSLAHKPADLKKYMLKYALNDKIEGIPEGTPNVLAFNNFSE*

>ORFP:Skud_Contig1923.6_YEL060C_Contig_c1923_880710633

MAWLKKLALVLLAIVPYATASPALSPRSREILSLEDLESEDKYVIGLKQGLSPTDLKKHLLRVSAVQYRNKNSTFEGGTGVKRTYAIGDYRAYTAVLDRDTVREIWNDTLEKPPWGLATLSNKKPHGFLYRYDKSAGEGTFAYVLDTGINSKHVDFEGRAYMGFSPPKTEPTDINGHGTHVAGIIGGKTFGVAKKTQLIGVKVFLDDEATTSTLMEGLEWAVNDITTKGRQGRSVINMSLGGPYSQALNDAIDHIADMGILPVAAAGNKGIPATFISPASADKAMTVGAINSDWQETNFSNFGPQVNILAPGEDVLSAYVSTNTATRVLSGTSMAAPHVAGLALYLMALEEFDSTQKLTDRILQLGMKNKVVNLMTDSPNLIIHNNVK*

>ORFP:Smik_Contig1661.1_YOR003W_Contig_c1661_1581594_reverse_complement

MASMRNIVLGAVFLWVQLIAASPAPIGGDSGLFGLLGLGSSQNGPNENGDKNASPTPAKSNVNANSKIVPNSYIAVYKNTTSEKAVKAMTASVSAQLKKRNLNKRGPGGEPLSTDVRAIKMNTWHAMTFQAEESMALEIGDADEVDYVEPDQWMSTSELVVQQNAPPGLQRLSEAAPVGQQQQKGAYVFDSSAGNTTTAYVVDSGCLTTHQDFEGRATTIANLVRGEKATDANGHGTHVACTIGGKNFGVAKKATVKCVKVMNANGQGQNADIIAGLQSVVNDVQKNNLQGKAVVNLSLGGGKSQALDAAMNNVFKAGIVPVVAAGNENACANRVLQQDAANTSPASARNAITVGAVDANTDQKASFSNFGRDVDINAPGVKVQSCGINSNTDVSVKSGTSMASPHVAGLAAYLMTLENIDSPANVTARLKQLSGNTQAQVGNGKSGTTPLIANNGNQKDKNEFLTGDGPAQGTPDTAAALN

>ORFP:Smik_Contig2758.2_YCR045C_Contig_c2758_10242499

MASIKNMVLGAVLLWAQLIAASPTPIGGDGGLLSLLGLGSSRNGDKNASPTPAKSNVKAKSRIVPNSYIVVYKNTTSAADVKAMTASVSSQLKKRNLNKRGSEGQPLSTNVRSFQINNWHAMNFEAEASMALEVSQYDGVDYVENNTWFSIQELVEQRNAPVGLQRLSEAAPVGQQTQKVDSGCRTTHQDFEGRATTIANFVKGERATDANGHGTHVACTIAGRKFGVAKKATVKCVKVMNAKGQGTNADIIAGLQTVAEDVKKTKPRAATMNMSLGGGRSQALDTAINNVFKAGVLPVQNAQNVSPAAAPNAVTVGAVDANTDQKAGFSNFGPSVDINAPGVDVQSCGIKSDTDVSTKSGTSMASPHVCGLANYLMRLENVSDPAKVTALLKGLSKETDATVEGGRRDTTPLIANNGNQKDKNTFLDENGPVKGGAQGNGAASN

>ORFP:Smik_Contig2875.5_YEL060C_Contig_c2875_907410900_reverse_complement

MAPITSILAPLAAAVAVLAPLATARPQLFPDDKFMGLPLANAEALNLIPSSFIVVYNSSKDQADIDASQASIMATIAKRNVFKRSLDGRQLSTRVETCQINKWRALMLDADDSTIMDIMQDPTVAYVEGDAKVQLNIPVNADIPEEDVDLHTLPADAAGPLTKRATSTQDGAPNGLARLSSSQPNPETQGTYAFDSSAGQNITVYVVDTGIRETHAEFEGRATFGANFVNRVNTDENGHGSHCAGTIGGKTFGVAKKANIIGVKVLDANGGGSNAGVIRGMEFVARDAKTKGLSRKAVMNMSLGGAASRAVNDAINNIRAAGVVPVAAAGNENADARGSSPASAPGAITVGAIDQRNDRKASFSNFGTVVDIFAPGVNVLSVDAKSDTGSKTLSGTSMAAPHIAGLAAYLMALKPELTAGGELSKVADAVDAEMKSLATAQNARVAGNPAGTTTLIANNGNV*

>ORFP_Scas_Contig706.10_YNL238W

KDYRYSTAELLEEHPVRALPDHYVFSKRISDPSFPKQWHLINAAFPGNDVNVKQLWYENVTGTGIVAAIVDDGVDYDNDNIKDNFSREGSWDFNDNGPLPKPKLKDDYHGTRCAGEIAASNGICGVGVAYDAKVAGIRILSGELTAEDEAASLVHALDVNDIYSCSWGPRDDGTHLQGPTDLVKKAMIRGVTEGRDQKGALYVFASGNGGAYGDNCNYDGYTNSIYSITVGAIDHKGLHPPYSESCSAVMVVTCSSGSGIHTTDICSNTHGGTSAAAPLAAGVYTLVLQANPELTWRDIQYVSILSSKQDGDWQMGALGYSHKYGYGKMDAYDMVTMARWENVKPQSWFYSSTIKIDEDQLKKANQRVEHVTVTVNIDTQIRGPTIIDLISPEGISNLGVVRKRDVSSDGFKDWTFMSVAHWGETGIGEWKLQVNWKLKFFGESIDPEKTRRRYEFTNDT

>PADG_01553T0

RNHDYSPAQMLGAGQVGELADHHTFAKRIDDPIFTQQWHLFNTEQPGHDINVTGLWLEGITGKGAISAIVDDGLDMYSNDLKDNYFAAGSYDYNDKVDEPRPRLYDDKHGTRCAGEVAGVNDVCGVGVAYDSSVAGIRILSKPVSDEDEAASINYRFQDNMIYSCSWGPVDDGTTMDAPGILVQRAIVNGIQKGRGGRGSVYVFAAGNGALHEDNCNFDGYTNSIYSVTVGAIDHNDDHPYYSEPCSAQLVVTYSSGGRIHTTDVCTTKHGGTSAAGPLVVGVVALALSVRPELTWRDVQYILLETAIPESDWQDTATGFSHEYGYGKVDAYSAVHLAMWKLVKPQAWLHSSSFEVTKELLMRNNERLEHVTLTMNINHTRRGDLSVELRSPTGVSYLSTTRKLDDLRAGYVDWTFMSLVHWGESGIGKWTVIVKDDWQLNLWGEAINADIQRKRIRYEFLYDAF

>PADG_04152T0

RPYWSNPLHRRYGQFLAARLLRPQQHVIDRIQAWLDWLTIIAEAELLLGTKYSIYRTMEWSLPQDLHDAIDTIQPTTSFTPLCLRTLYTRMGLVNYLGEFNNRSDISQFLFEDISIGGNQQEGNLDAEVMIGIAHPTTYSVGGPNEPLAWLNWILDQPDSNLPSVVSTSYGDIEHVPYARRVCNGFAQLGARGVSVIMGSGDHGVGCYSNDGFLVSFPDSCPWVTSVGAEVVSSGGFSNYFPRPDYQSVSQYLMFNPQGRAIPDVSAQGYVTIWNGVDGTSASTPTFAAIVALVNDVLAAENKPSMGFLNPWLYFSDVTEGGCGFPALPGWDAASGWGTPFPKF

>PADG_04156T0

VLRPAGWVSIGIQQGWDMFEQHLSDPSHSRYGQHLVDQLVQASPASFDAVTAWLDWISFIWILERLFDAKYAQYRSLSWSLPANLADHIDIVEPTNSFSVVCLSVLYNSIALVNFLGEVNNRSDVDLFLFTTEIVNGDRQEGALDAQTILGLSWPTAYNVGSKNEPLAWLQHMQSKETLPHVISISYADTEQVPYARRVCNEFAKLGARGVSILVASGDWGVGCIIDSIFAPSFPASCPYVTSVGAEIVSGGGFSDYFERPRYQAVEEYLLYNQNGRGYPDIAAMGFSVLWNGQDGTSASAPTVAAIIALVNDALLENSRPPLGFLNPWIYFTDVTWGGCGFPASRGWDPATGLGTPFPKL

>PADG_05742T0_|_PADG_05742_|_Paracoccidioides_brasiliensis_Pb18_subtilisin_(translation)_(691_aa)

MLTISINGNLFDPVNQLQELIDLGLHDEDASKSDYILIHTSQPLTNDQYDKLENRSVKILEYVSANTYLCSYKNTDLEERIRSLPFVSWANPYLNQFVVQSSLKSMPPIFNPISVFTPVPKTSRLHLVNIVVHHDVDPNNDAVKLAVAMAARADPDSLAVSARQIRLQVQQQYLDDVAAVDAVYLIQQVHPFILFNNKAWQVLGCDTKIVGEETEYTGEGQVVGIGDTGFDIGKTDDTHHAFTGRVKKLYSLGRPNKTDDPDGHGTHVSGSILGDGSSKTMGGRIAGAAPRASLALQSVLDTTNGLGGIPDDLGDLFIVPYREVGARVHTNSWGASSIFGQIPYDASATQVDKFIWDNLDMLVLFAAGNDGADRNFDGVIDLQQIGSQAAAKNCLTVGASENNRPEIGVKYGYRWPSTPFRTDKMADNPAGMAAFSSRGPTREGRYKPDVVAPGTAVLSSLSRKAQADNQFGESSDPQWFFLAGTSMATPLVAGCAAVVRESLVKNGTEHPSAALVKALLINGAMELVGQYKPSEAGPSPNNNSGFGLVNLVNSIILPKQDNGGFKEGGPLSQGEGEDNPITITIPKLTSELAAEGSTLKVTLVWSDPPGAELQNDLDLIVKSSDGQERHGNMGEEVDFDRVNNVEQVTWTGIPEGETQIIVRAFRITRREAPQPYAVVWSINRPLKPKK*

>PADG_07422T0_|_PADG_07422_|_Paracoccidioides_brasiliensis_Pb18_subtilasetype_proteinase_psp3_(translation)_(496_aa)

MAPISSILAPFAALAGLVAALPSNFPGSEKFAGVPVSNINALNVIPQSYIVVYNSTFDSKIIDDSQAMAMAKVAERNLGKRSEDGRDMSTTATTCQIGTWRGLMLDADDQTIMEIMADPAVRYVEADAAMNILIDEEASEMPPPAEITEMQAIARRAEQTETGAPNGLARLSQSEPNPGTSGTYRFEDSAGEGITVYIVDTGIRATHTEFEDRATFGANFIDQVDTDQNGHGSHCAGTIGGKTFGVAKKATLVGVKVLGASGGGSNRGVIQGMQFVANDVTQKNLARKAVMNMSLGGSKSTAVNEAINSMIKAGVVAVVAAGNENDDASKSSPASAPGAITVGAIDQKNDRKASFSNFGTDVDIFAPGVNVLSVDAKSDTGSKTLSGTSMASPHVAGLAAYLMALDPTLTAGGGIETVAGKVDARIKELAKAMGAQVGQAPAGTTTLIANNGKTGNGAGNGTAAAAPADNADAAPATGATRRLRKAQRLTFQRMQQFQRQQIQRQQSQRQQ*

>PADG_07705T0_|_PADG_07705_|_Paracoccidioides_brasiliensis_Pb18_glucan_13betaglucosidase_(translation)_(1863_aa)

MAREQNGPVWLKSGSLKNYMAHLADPAGYSHTLTGDSGKFMEVSGDNFNVTAAVSNYWLPQLAPLGKPGTYKICRPVVQLYYTQFIGDALDPPTIKGCDTFQGIALFDTDPYIPGGNGQNWYINQNQFFRQIRNFIFDLTEMPLQTNDHDQPLVPTGIHWQVSQACSLQNLVFNMPKATDKNKATHVGIFMENGSGGFVSDLVFNGGNIGWRAYSKISNCAIAILTNSRKDTKSSPPNIVIDNLEMSNVGTTVKSETGEVILAGTNKVDLWAIGRRYNGYKGTYTSGKVDAPRKGSRLLDKDGKLFYRRRPQYEEFGIGQFLIATEHGCKNDGTGDNTQAINSFLQAAKDAGQIAYFPAGIYRVGSTVLIPTGSRVVGGSWSQIQGAGFYFNDLHNPRVVVQVGKKGDVGTMEIVDMMFTVQGATAGAIVLEWNVYESSQGSAAMWDSHVRIGGATGSDLDFKTCPKFGYEDSCICASLLFHVTPQASGYFENVWIWLADHDNDMNVYNTPDKISNQISLYAARGTLIESKGPSWFYGTGSEHTVLYQYQLYGAKDVYLGHIQTETPYFQPVPLAPLPFHTGKEFPGDPSFEKCKTIGCQEAWGLRIINSEGITLHSSGLYSFFEEYYQDCVPKHNCQEGLLEVRGSKDVVLFNIFTVGVQKIGTGINGFTTEVSVWLPLPGDDNFDVVYVGTEVYNKPSVTCPANCILVFPTSSLASKTTIDPGKYTTSLEYGRSGTTTIGGQPIPTFFTTTTTVTLDINPITTDGIPYSNINITEGQAKSTLTVLPSVDIPPIPVPLPDGEGKTTTRNVTVPPWPDITRGPPGHTATRTDDDTRPGVYHTPYVTTISAARAIVTTISFPSTVSPIVVTCPPNTEVPFNTPKTIATVFCPTPTTISVAFTCPATKVVTFLGSSAGVFTVDCILSTTFSKEPPITTPVPTGDPICLNKGTFKGLEWILPPGVYPPVGAHRLPALLICLQHGRSNLLSRLGPKSLSGGAATTTTTKTPDNCPLPTTRPKALQTGNADSGGSKPESGLVSAAGPPPPGCPADAVVYPSNPKNVGKIPDILAAYKGKYVEVKSESFQFTSFFWVPFLDQKTMDILKESPDVEFAYYYQQWNANVGPEEPRPRTPTFANSQTQGVGSRSNVSNVVSDHDHGNATLNRFNKRLPIKKSSPFTWDLSQISIPKGWYWNTLSSGSVDPQTKNWAYRYDSVNGEGQTVYLLKEDGFWPNHLEFSNMAGSIETLPITIPIGNIPVGNSDNIAHGSAVAAKAAGSQLGACTKCRIVLVPISGVERIPRGIWYERLIERILQRLIDVLEDIQRNNLQGKAVINMSVSFIGPLLNATFLRSLFTHLQLLEDQVQAVLVVTSGNKSPEFPMINDYPALFGNPTNRLGYLPNLIVVGATNHQACQAKFSQFSDWMTTFAPGQNMWVPNDPAIPSPIFFAISSGTSLAAPLVSGMIAYWRSLQSPWRDQLALPQNVKKMVKIFHRRIPIHNQQIDPLLRKPIIWNGQVIDHSCLGDYDTISQWDITHACPKINRDLSQETNEGETIQPCGPGTFGTLADGTYCPHLPDDGTGGHKVTFTTGATPAPTCPSRTGCGGHLCTGFYCSPNPTGIPPDHRDPKDPNASKPVPTRTIGAPPGPTNKPTITKTSGPEPTCDDRCKLDAGNRCKCSENGCDSESPSCCANASCPMCECGENGCSPKSPSCCASGTCEWSWTGGGGGDGSGKPPNRNPKAGTVLMVFMESLSQGPGGEVWTRQWNIFGAAGDASVDMCWDTPLLVADSNKDATGNKPGLPPKLPMIRTQGEKCSYTGTETTLGEFRCERGLSESKCQVLDPFPLERCPPLANPVMRAVVHCRWKT*

>PADG_07910T0_|_PADG_07910_|_Paracoccidioides_brasiliensis_Pb18_alkaline_proteinase_(translation)_(396_aa)

MAPISKEVPAGAITGAADFFAKGFSAMLNSESTERVSNSYIVVYNNTFDDDTINFRQTEVMNHIKRRNIGKRGLDGRQLSTEVHALAMNGWRAMTLESDDDMILQIMNEPEVAWVEANARVKLSASVAQMNAPPGLNRLSNAQAGTGNYVFDTTGGEGVTVYVVDTGIRTDHSEFQGRATFGVNTVDNVDNDQNGHGSHVAGTICGQTFGVAKSANVVAVKVLDGTGAGSNAGVLDGLQFIINDVQQKNLRGKAVMNMSLGGPQSAAVNRAVQALFDAGVVPVVAAGNENQDAANTSPASAPKAITVGAIDASNDQKASFSNFGADVDIFAPGVDVLSVGIRSNTATDTLSGTSMASPHVAGLAAYLIALENINTPEAVASRLTELATASGAQVLRNTPGTTNLIANNGQL*

>PADG_08053T0_|_PADG_08053_|_Paracoccidioides_brasiliensis_Pb18_glucan_13betaglucosidase_(translation)_(2038_aa)

MIYPKNMSSLGLFLQFLSLVAAQASLLYDPARFPNIPSYGPQPNPPYMVDNQYNYFKVAPDRGEQNGPVWLESGSFETYMLNSGHSKQHNDTSVDAPGNLKKIVGSASNTTAASSYWLPKLAPLGQPLAGAGYKFYRDVVEYGADNTGETDATEAINAAIGDGKRCGLECGNTFVQGAIIYFPPGTYKICRPVIQLYYTQFIGDALNPPTIKGCDTFQGIALFDTDPYIPGGNGQNWYINQNQFFRQIRNFIFDLTEMPLTTDDHDQPLVPTGIFMENGSGGFVSDLEFNGGNIGWRAGSQQYTAMNLKFNGCLTAVQMIWSWGFNWQRIEVDGGAIAFNISGRGGSTGQGIGSVSIIDSRISNCPIAILTNTRKDGVNGSPNIVIDNLAMYNVETTVQSDNGDVILKGTDFVKLWAIGRRYNGYNGTYTAGEVDAPRKGSGLLNINGKLFYQPRPQYEDLRLDQFLIATEHGCMNDGTGDNTADINSFLEKANRGGKVAYFPAGIYRVGGTVLIPTGSRVQGASWSQIQGAGVYFNDLHNPRVVVQVGERGDVGSMEIVDMMFTTQGATAGAIVLEWNVHQDRQGSAAMWDSHVRVGGSLGTDLDVKTCPKFEFRDACICASLLFHVTPQASGYFENVWIWLADHDNDMSVYDSPDKLSNQISLYAARGTLIESEGPSWFYGTGSEHTVMYQYQLYGAKNPVPIAPLPFVPGKEFPGDPSFEKCKTIGCQAAWGLRIINSKKRSLEVRGSKGVALFNIFTVGMVEIGTGINHGAVLQNDSNQSGFTTEVSVWLPLSGDDEYDIVYVGPEVYEKPSVTCPADCILVFPTSSLSSRTTIDPGNYTTSLEYGHRGTTIISGREVPTFYTTVTTITLAIDPIITDGMPYSNINITKGQTSAVLTVLPSVDIPPVPVPMPDGEGKTTTRNVTVPPWPDITRGPPESWSDPNASPTEGTREGVYHTPFVTTVVATRPTVSTISFSSTVSPIVVECPPNSKVPFNTPKTTPTINCRKPTTVSIGFTCPATKVVTFIGSSTGVFTVDCTVSTTFTKSEETITPSPTNKPTTTQPLPVWSTWPPRVITPIEEEVKKPEPGKTPCKLWFFSQFCLNREKGETRGLRWILPPGIYPPGPPPPRAINLPSWTIKSPLPPWPPITVGRDNIITYPQKEPTKCEKKSASICATTVFKATTTRGTITSTSSSTSSTCDTIYGCSASDWDTTTTRTKPDNCPSPTAKPGQAAARPPIPPWGCPANALVYPSDMDDVRHIRELLAKYKDKYVEVKSEALHFTVFFWVPYLDQETMNTLVESPDVADAFYYEHRPDNDGILTDLRGRVRDISYNVDAIKSHGQGGVNHSNDSVVAQGNGNVDQLMPSALIARESNYIWDLSQISMPRGMIWKDPYSATLSANGQFMFHYDRISGDDQYIYIMDEDEISETHPEMTHHNDIEYLRPDDRYSDPGPARPDVEHGTGVASKVIGRNLGSCQRCTLIVSSLFEGVDEQDESVTLARYLDQVRGVIDDVLLKNRMGKAVINISANFDPTVGVVFIRELHKLLVELDGLNVAIVVSASNRADESPEIDRYPALFAKPGSDLYIPNLIVVGATDAHGHITDFSQYADWMTTFAPGDNIWIPLGDMDYMTSSGTSYSAPFVSGLIGYFRSLPSPWVNQLKDPGSVKKMIKLFHRRIVVSNEPINVHKMKPIIWNGQFGEYSCLSDYRTVEQWDPRGYCPRIKLDLHEETNDGETVAPCRYDSVVLDNSLKRFDGSYCPRLPKTGPSGHTVSFTSNGRVKPSPTCASGTGCGGRLCTGFFCSTMPTGVPPDRHDPKDPNASSRVPTTTKPSDPEPTCGDKCKLDKGNRCSCGENGCDDQSPSCCANASCPYCECGENGCTPSSPWCCGNDSCEWSRTGGGGGNNPRPKPRTGFVLISVSNVENPAPPHIHHYWELWSATPSKNVDMCKDDPLVKEKTDDIAGAFPPSLGPFTANGVTCKYIGNKKNAGKLECDTNVSKTSCKAVDPIKKYECTLENPIKYLTVSCEWEAQELP*

>Pans_587.t1

VMDPRGWLRIALQQRAEALEQAVSTPGHPKYGRHLLRSYTTPSQSATLAVTRWLDWVTFVENASHLLKTDFAWYRTLAYSVPDEVAPHIDLVQPTTRFTPDCLKWLYNTIAFASFLEQYARYDDFQTFQFTVELVGGDDQEANLDVQYIHAVSHPLQYSTGGRNEPLEWLTYLLNQTDDKIPKVISVSYGEEEQIPYAVKVCNMFMQLGGRGVSVIFASGDSGPGCIRSTDFEPTFPAGCPYVTSVGAEKASSGGFSMYHPRPQWQAVEPYLYFDPRGRAIPDISAQGFNVIDKGLSGTSASAPVVAGIVGLLNAARYTLGLPSLGFLNPWLYFTDVVAGGCSWNATVGWDPVTGLGTPFGRL

>Pans_6015.t1

VILPPGWLTIALEPGFAEVKARLRQFGGNNHHLLHTYLQPKNKNIETVKSWLSLLSFARQVKDLFSADLKYYRALSYSIPSWLRAYIDFVHPITNFSPRCIKQLYINFGVAGFLEQWILHADVAYFLFTVELIGGNPQEASLDVEYAMALGYPIYYVTGGRGNPLPRRNRQRALPRIVPYALRVCDLFAALAARGVSTFVASGDGGAACVMNDGFIPTFPASCPYSAGGFSNYFDRPSWQAVKPYVLFNSTGRAVPDISAIGFQIIMGGVLGTSASAPVVAAMVALINDARMRAGKQSLGWLNPLLYLRDVMVGGCGWPAVQGYDCVTGLGAVFDEL

>Pans_DSM_980:PODANSg1184.t1_gene_PODANSg1184_Pans_DSM_980_chrm5_SC1:join(489676..489972490026..490985)

MALIRTVSLGLVLLWSQLVAATPAPSRPVPTNPVLPIANANAPNIIPNRYIVVYNSSFSGEAIDAKMLSVSTAIKKRNLNKRGLEGRQLSTDTMSFKMNKWRAMALDADDSMIADINRANEVAYVEADQWIHASATIKQINAPLGLQRLSEPRPAGESSYLFDESAGEGIMVYVVDTGVRITHSEFQGRARFGASFVGQDSRNADDDNGHGSHVAGTIAGGTFGVAKKARITAVKVLDAKGSGANSGILGGLQFVMDDVKRRNLKGKAIMNMSLGGEFSDAMNHAIENVVNSSVICVVAAGNENQNAARVSPASAPSAITVGAIDARNDAKAAFSNFGPDVDIFAPGVDVLSVGIRSDTDTKTLSGTSMASPHIAGLTAYLMHIRQDVREPADVKKMLKTLGSKTGARVKGNPPRIETTRVIANNGHAETNSGPINQPGNQQPGTGRPGNAQPGTGNERPRTGRPGNVQPGTGNERPRTGRPGNAQPGTGNERPRTGRPGNVQPGTGNGGNG

>Pans_DSM_980:PODANSg1911.t1_gene_PODANSg1911_Pans_DSM_980_chrm5_SC4:join(118126..119104119218..120130120279..121272)

MMDRQYFNTIPEERQRFDSSIQERRILDVVQPLFATNLPWKLEADSDHGNGLAEEINAQTAKEMFLHELVTAGLYLRELNSALSVRSFRLCLETSSDLLAFLETLVCNDILGQPRPGDTSGVDNTPGIHKDKPPENEPVQVVGVPLVLDLVAKWLFQIPEPKPTALGRTEQPTVRPHDDIGALQDLCDVEKYPKLEMLGRILQESGDRACHKLINFHQRMANFQEGVRKAWHLNKCLANLRKMAPTKRFRPPAAQRIPRTKWENTIPRDLSSRLFEKLYSSVCQNQDHQVKLQLNGFDMDSFLAPPIHFNLFLPSCPTNDLWQECQYVEMIVDLCEDIRALNENHHLPTVLAIHFEVVEPGHRYYLWHDPKYQLDTPQHFQDAKPTISLDYLICSGFLTDIPEGGVFGMNDKAVLALSLSRCFLHLWGTNWLKDPWTAGNIHFLARSDEIWNPHHPFIHCSLDNSSPTSSIQATISLFSFAKLLLEIETGTRIDVDPSSVTQDEFEDTIVSILDSRGDRFGRREYNAAVDGCFHIFALVNKQSNEAGGVMDELDMMRKAIYDAIVEPLEQNFNLIPNPSKALYVKKLHLERKRGTYGVATFQPEYHQPTRKRGSTDSTMFFDGEIVRTVDERVDRAAKFFDSMDRFHAQFIQPRITKSNGLRPVKIAILDTGIEVDNSSLQGVLDNIKDSRKASGFRDWRRGSIRAVQSFVTSPGDERDVVGHGTHVAWLALKTALNVEVYIAKVSAGKQFDDNTAVVKAIDWAMDQEVDIMVMSFGSAYIDKRVSDAIDRAVTTRPHPTLVFAAASNSGLNSRPSFPATHDKVIGVYALDGYGNDNGGLNPSRQPGGTYFGTLGVGIEMLWNNKKEARSGSSYAAPIAAGIAANCLVWLEHMLKKGYFSQDQYTWLRTIEGMRYMLRKQAMGSNGDRVGILSLAPWVLWRRDLTDVEVCTILKEGMPLFR*

>Pans_DSM_980:PODANSg199.t1_gene_PODANSg199_Pans_DSM_980_chrm2_SC3:join(37625..3790937967..3815238227..3839938455..38989)

MVRFSVAAAFLLSALGVTAAPSGGRHNHQNTQNTGATAGNAAGVPVANSDISNIIPGRYIVVYNNTFGEEAINAHQIKVTSLVAKRNLGKRDAKTGRIMSPSVKAFKMGTWRAMALDADDDMINDINSAQEVEYIEADQYVKLNALTSQNSTTTGLARLSHAGPSKKAAPYIFDSSAGEGITAFVVDTGIRVTHSEYEGRATFAANFVNNVDTDENGHGSHVAGTIAGATFGVAKKAKLVAVKVLDGSGSGSNSGVLQGMQFVADTATSQKLGGKAVLNMSLGGGKSRAINSAINQIAAAGVVPVVAAGNENQDTANTSPGSAPAAITVGAIDQRTDARASFSNFGAGVDIFAPGVNVLSVGIKSDTDTDTLSGTSMASPHVAGLAAYLMALEGLTDVTAVGNRIKELAQKTGAKVTNNVRGTTSLIANNGNL*

>Pans_DSM_980:PODANSg2023.t1_gene_PODANSg2023_Pans_DSM_980_chrm1_SC3:join(54626..5499755058..56281)

MGLVTNPFAKNIIPNRYIVVYNNSFGEEAISAKQAQFAAKIAKRNLGKRGLFGNELSTAIHSFSMHTWRAMALDADDIMIKDIFDAEEVAYIEADTKVQHAALVAQTNAAPGLIRLSNKAVGGQNYIFDNSAGSNITAYVVDTGIRITHSEFEGRATFGANFVNDDTDENGHGSHVAGTIGGATFGVAKNVELVAVKVLDADGSGSNSGVLNGMQFVVNDVQAKKRSGKAVMNMSLGGSFSTAVNNAITALTNAGIVPVVAAGNENQDTANTSPGSAPQAITVGAIDATTDIRAGFSNFGTGVDIYAPGVDVLSVGIKSDIDTAVLSGTSMASPHVAGLAAYLMALEGVSNVDDVSNLIKNLAAKTGAAVKQNIAGTTSLIANNGNF*

>Pans_DSM_980:PODANSg2378.t1_gene_PODANSg2378_Pans_DSM_980_chrm5_SC5:complement(176474..177754)

MAFLKTFLASLLIAAPLATAAPVDVAQTIGNMAEAVGSDIGGGLGEELDKKMGISAFLRPMLSNPDSLNIIPNRYIVVYNDTFDDDAIAAKEFSIASAIKKRNLNKRSSIGQAMSTSIKSFRMNTWRAMSLDADDMMVQDLFKSDEVAYIEADTRVQLNAAIAQVNAPPGLNRLSHAQADQENYIFDDSAGEGITAYVVDTGIKVDHSEFEGRATFGGNFIDNVNGHGSHVAGTIGGATFGVAKKVDLVAVKVLDASGGGSNSGVLQGMQFVVDDVKKNNRAGKAVMNMSLGGDKSEAINRAIEALFKAGVVPVVAAGNENRETALTSPGSAPNAITVGAIDATSDQRADFSNFGPEVDIYAPGVDVLSVGIKSNTDTATLSGTSMASPHVAGLAAYLMGFQKLDGPAQVASLIKSLAAESGAKVRNNVRGTTDGIANNGNQ

>Pans_DSM_980:PODANSg2598.t1_gene_PODANSg2598_Pans_DSM_980_chrm5_SC10:complement(join(26837..2738827508..2794228036..28326))

MALLRTFLASLLVAAPFAAAAPIDVAETLGDMADSIGGGLGGEADKKMGISAFLRPMLKNPDALNVIPNRYIVVYNDTFDDDTISAKEASFAAAIKKRNLNKRSSIGKAMSTSIQSFRMNKWRAMSLDADDLMVQDLWNSDEVAYIEADTKVQLNAAIAQVNAPPGLDRLSHAKVNQDTYVFDDSAGEGITAYVVDTGIKIDHSEFEGRATFGANFINNVDDDENGHGSHVAGTIGGATFGVAKKVDLVAVKVLDASGGGSNSGVLQGMQFVIDDAKKKNRVGKAVMNMSLGGDFSQAINRAIEALFKAGIVPVVAAGNENRETALTSPGSAPNAITVGAIDATTDERADFSNFGPEVDVYAPGVNVLSVGIKSNTDTATLSGTSMASPHVAGLAAYLMGFQQLDGPAQVASLIKSLAGQTGAKVQNNVQGTTDSIANNGNQ

>Pans_DSM_980:PODANSg4255.t1_gene_PODANSg4255_Pans_DSM_980_chrm1_SC4:join(1215435..12159291215986..12161311216187..12164451216500..12166251216690..12174611217525..12176971217762..12182451218310..12194)

MVDNQKNYYGVAPDKGKQNGPVWRYSESFAKYMSNLRANKVIRGGYGKGLTPGAGDRPQNHLNGTLGSNSTLYLNNTRTLLSRGSAPLHKRASDYWLTTLGPLGIQPHAGGSNYKFYRDVVADYGADNTGESDASEAINAAVEDGNRCGLECGNTFTQGAIIYFPPGTYKICSPVVQLYYSQFIGDPHDPPTIKGCRSFKGIALFDADPYIPGGGGQNWYINQNQFFRQIRNFIFDLTEMPISTADNDQPLVPTGIHWQVSQATSLENLVFNMPKATDDETTTAVGIFTENGSGGFVADLTFNGGAIGWRAGSQQYTARNLKFNGCLTAVQMIWDWGFNWQGIEVKDSAIAFNISGRGGATGQGIGSISVIDSSITNTPIGILTNSHEQSPNIVLDNVKISNVAQVVQVDNGPSLLSGTSDTATIDLWAHGRRYNGDKGSSETGPVKAPSKAAGLLGDDKKLFTKSRPQYADFSPENFLVATKEGIKNDGTGDQTLAINAFLLKAKANSQIAYFPAGIYQVGGTVFIPTGSRVVGSSWSQIQGSGFYFADMNAPRVMVRVGNRGDLGTMEITDMLFTVKGATAGAILVEWNVAGDEQGAAGMWDSHVRVGGGIGTDLDIDNCPKGGFNDQCICASMLLHVTAQASGYFENVWVWVADMVVYDSPDKLINQISLYAARCTLIESQEPTWFYGTGSEHCVMYQYQLNKAKNVYLGHIQSETPYYQPNPVAPYPFDGARPMAADPSFLECTTDSCKAGWGLRIIDSENITIHGSGLYSFFQDYYQDCLETFDCQDKILEVKGSKNVAIFNLFTVGTVNIASATRISLGMNLTNGKSNSSATQQEGNISRLTRDSGFTTEVSVWLPLDGSDNISVVYVGPEIWDRPTAACSPPCVLVLPTSTLGQDTTISPSEYTTSLEYGRQGSSTGPGGQVITTFYTTTTTITITVPPITIPKNSGLPYSNVNVTRGQGGGGFIATPRVEIPPIGVPLPDGNGGTTTRQVFLPPWPDVNRGPPEDWEYDGPWGNPSPIPSGGVGQAFHTPWSTIVTASAATVTTLTFPAIVHPQTYQCPPSSEISFNTPRMTLTVDCPTPTEFKFGFSCPTTKVVTFLGPSAGVFTVDCTVSSVWDFPRFPEPTPGPSESTTSDEPLPVWTTWPPGEITPVEREVEESEPEDDGTFTSCKLWFFFFCIRWDNIKIGGWKWTLPPGIYPPGPPPPFIKFPFKVEGTLPPWPEITIGRDRKLTYSNEPTSCTTKSASLCTTTTIFSVTKIAEDSTRTTATATSRQCETVRGCDVTDQNTRTVSTAIESCTPKPRVKARDGDDPSLLNPRQNGNGNSCGKNAIVYPKDPKSAGDIPSLLSAYAGKYETIGVPELNIVGYWWVPLLDEETMKRLKSSPFVNDAYYYQDWNNAGNGPDRSGNINGEMALPAHSEGITHDDDGWTSLASPLFKRARTTTATNFWASSIVSLPKGWNWREAGTDSYDYSNLANPYLYQWDDQGGSTEHTIYVTGEARVWTDHPEFKASEGFQGELDIVLPGGKYDVENDKADAFHGTCVAAYAIGAKLGICKKCRGVWFETKLWSTDDPYFNPNFVRERGIAHLMAAFRDIHLRGNAKKAVINMSWSYPPGRAIPATLQSTHWVLTELDKMGVVLVASSGNHAHDEGREISRFPARFASSDKKRNPYGEIKNLIVVGATQNIGIEYTRGQTSNYMTTFAPGENVPCTSDPNAAGDKYSRNMASGTSAAAPQVAGLAAYWRSLPSKWQTQLEEPANVKKLIRLFHRRYGFWNAQRAKNFPILAQSKPVIWNGQVKDKNCLVDYDTRQTWDTTKACPDIPDRLSTLPENPGESVNCNNPAPPPNSKRQAGSGGSCPFTPGGSGAEKSIDYQPKPTPSPTCQSNNCGGKLCTGFFCRPKPSGIPPDYMDPKDPNAGNPVPVTHIPGPNKPTTTRGGGGTPTPSPECDDKCKLDRGNPCRCGDTGCDEQSPACCHNASCPKCDCPKNGDGCSKNSPACCASGTCQWQYTGGGGGLEPNPVDEPDSGANRVASPPSSTETVTEDPEHVDAVYEIYSERDAKGGFVVSGFSGDVNGTVVGMGGVEPDWVMSGNGTSGLETSYKGVVAYGRRCDFLAGSVNGYEGMERPGMVVGALTCEGVAPAVCVRGEGGGEVREELVCRWE*

>Pans_DSM_980:PODANSg667.t1_gene_PODANSg667_Pans_DSM_980_chrm4_SC1:join(193606..193759193824..194134194218..194736194830..195027)

MALLRTFLASLLVAAPFAAAAPIDVAETMGDMADSIGGGLGNEADKKMGISAFLRPMLKNPDAQNIIANRYIVVYNDTFDDDAISAKEASFAAAIKKRNLNKRSSIGKAMSTSIQSFRMNKWRAMSLDADDLMVQDLYNSDEVAYIEADQKVQLNAAIAQVNAPPGLDRLSHAQVNQDTYVFDDSAGEGITAYVVDTGIKVDHSEFEGRATFGANFINNVDDDENGHGSHVAGTIGGATFGVAKKVDLVAVKVLDASGGGSNSGVLQGMQFVIDDAKKKNRVGKAVMNMSLGGDFSQAINRAIEALFKAGVVPVVAAGNENRETALTSPGSAPNAITVGAIDATTDERADFSNFGPEVDVYAPGVNVLSVGIKSNTDTATLSGTSMASPHVAGLAAYLMGFQQLDGPAQVASLIKSLAGSTGAKVKNNVQGTTSNIANNGNQ

>Pans_DSM_980:PODANSg717.t1_gene_PODANSg717_Pans_DSM_980_chrm4_SC1:complement(join(357616..359716359805..360827361158..362014))

MEDEAADSLSWNIALAPTGDSGSQFHTKNDPNKPLQRQNYVERKGAVDVRCSCADVVHGFLSPDSDILCTLIVLDFRFDSRKRARRIASVHIDLRFSSLDPNSPYQPEVRAISPDGNFTVAPTTQTESSTLSGNLGISATPGPAPTLSAGISIDKSITRDMTYAATVVGAKSLRGRNFGQPNSASWTLLENPATETGVPVAMRAAVLLEREDEELFQCSVTIKARADWRTTLESVFGSTPPDDPVLFDPTIESKRTHYDEMNLAEAFKQLSTVPNVTFRKGLPEQSVEVDDSDEEYDDGPAHNPFHSFAIIQKDPTKEAFDQILADAESNKLRLGDKAYREAFKQKYQQYFEGRVAPENQTLLHLVANRVGHRGLTLLLIKNSPQLLSVADDNGKTPLWIAITRKNENVLRAIVEKFNGDLDSLLARTCDHGRNSIHAAIIHNLPEQHTLNLIQRASKGTLCAGDYDGLTPLHLAIQYDRCSASQQRIVEALLQRADECLDQFTTNPSQLSVYEYHYYTRADAERKTGLLAGQAGEPNGTSQSSNQNRGKSPSGRVGHLPDNVPHLYNVESSRGRTTNHAPGKKGGDVEPQRGREAIREQSNAPSTRNMDPPPPGPINGVKRDVNREARHCDFSTGPTSRVRISVSEPSRQYGVVTDAPPDWNLYFDYSKAVSPVTRKNFEQSFAHMRFDSVLRYVAFNKIELDSPGNNTSKLHAKRLAQQPRPGRGRVDLVFFFNWLREKSVKHILKVVVDDWPERPHSDKAIEDSLKHFEIESLEWRKSDICPETLFVACQHVRHLHLWWSGNRAVLRAWSEPDGLVRLEKLKTIHLLWNSAEVLEPADRIQSYVEDFKRRLRKAVRNYELEKQGITAAARPQNDKAVPVPGIRRTVTGDRPSMDGPVRTITVETIEENFSRGDSGSSSGTRAKPMRVANQRNLSAHKWLNCMDAFADGIQGVEVPPTQHKLLLKDITVALIDDGVNIDTSSIGGKVIGGATFDRGEPDENGPSPYFISASGHGTVMADMICRVCPTAKLYVFKLETHLSPDSLVDGQTHNQIVARSATQVCHPPYALPLLSAQILTPVQSVDAAVARGVDIISISWTVKKPKERERDADLKDLGDAIKRALDAGILVFCAAGDAGNFSDEEYPYEFDRSRIIRIGAATDDGRPWERSGDTHNLNFIFPGCSVVSRHETINSSIPSHFQENTGSSVATALAAGLAALVLHVVRLAAIFGENERERVKSGGGGTAVNGVGSGAAGPVVEAGRLVSLKDHRNMKLVFQKMGVDREIGKFIEVWEYFEGPTEGLRMGGKAPEVVTGLAVRFVSSMKL*

>Pans_DSM_980:PODANSg872.t1_gene_PODANSg872_Pans_DSM_980_chrm4_SC2:join(26..253345..557623..770842..1869)

DVELSFDLSGYDNWTVSEEQFKIFLGKLKFSDVLQYVGIPKVPLIHRKAAVSREPLGSAIGRNDLLFASDCLRDKGQLKDHLKALGSKSGIGKKTDTCSEVIWNVAPNVREVQFYWNGNNAVLRGWAESGGLRKLSKLKQVFLSIEEIRIRSPNWHLCYGLCRAHEGTLRWSQGRTTRSSVPKPHRIISLPAYPTESRKYFDVETDAGGSKTLRQTVGASIKIALIDDGVDYKDLPKRTFIGGHSFSTRDKERNLIHPHYVSIMGHGTAMARHIYYMCPSAEIYVLRLEDYHHPEDPNLRLIAARSAVKAIRAAVGKEVNINSVSWTIEPTGTEGDQNLELAIADAAQRGILMFCSASDRGAKQNATYPSKAAPGRIFTIGAASVWRNAVASVGSLDRITFILPGEKVSVPGSERASLSFREPSGSSVATALGAGLAALMLYCVKVHLVLAKPGPEKERIQEDYSLLLKHEIMVKVIYGIGMTPDTKFIKVCGVFGKSTSAERVVRRERVSPFNSRGWEDTVRETAVDSVVQLAPKMT*

>Pans_DSM_980_PODANSg09433.t1

RNYDYSPARSLGEGPLGNLKDHHIFVKRIQDPIFNEQWHLFNTVEVGHDVNVTGLWLEGITGKNATVAIVDDGLDMYSDDLKDNYYAAGSYDFNDKTEEPKPRLSDDRHGTRCAGEVAAGNTVCGVGVAYDARISGQRILSKLISDADEAVAMNYDFDHNQIYSCSWGPPDDGKSMDAPGILIKRAMLNAVQNGRQGLGSIYVFASGNGAMAEDNCNFDGYTNSIYSITVGAIDRKGLHPYYSEKCSAGLVVTYSSGSGIHTTDVCSNTHGGTSAAAPLAAGIFALVLSVRPDLTWRDMQYLAMDTAIPDGDWQPTTIGFSHTYGYGKLDSYAIVHAAKWKNVKPQAWFYSVPFEVTEDMLKEANERLEHVTVTMNLKHARRGDVSVDLISPNKVSHLSTTRKFDDSTEGYDDWTFMSVAHWGESGVGTWTIIVKDDWHLKLWGESRDASKARKRLRYEFLYDAF

>Pans_DSM_980_PODANSg4588.t1

YIVFKGFPVHLMTVNKFKGITGKGIKIAVIDTGCLVSYGADLVPDPVDNCHGTHVAGIIAGAAEGVQLGAYRVFGCDLLIAYNMAYEAGSDIITASIGAAVVSRIVVPCVVSAGNGAAGFYASTAANGKQVTAIASVDNHGCVLIRGASSFTSWGPTVESKPQFSTPGGILSTYPGTSMACPAAIYALAKPAPVPQQGAGLVQAWDATLLSNDTVSNGSSSVTYSLSNVGATLGAGLPVYSGYIPYLGGRLPAGRYKLTVRALRIFGEY

>pbla_17468_gene_e_gw1.4.689.1_pbla_scaffold_4:complement(join(1716833..17172491717334..17178611717931..1718167))

MAGRFLVSLSVALSALGVSAAPATTFTKSEGFYIGVPIANPDAQNLIPHKFIVVYNNTFSDDEVFAHESSIASVIAKRNIGKRSPLTNNILSTTVETFQIDQWRAMALEADDALINEIYAAKEVSYIEQDAVIKLNVRQLQSRAPTGLARLSHDGARQNGYFFDSSAGEGITAYVVDTGIRTTHEDLQGRARFGASFIRGEDETDLNGHGTHVAGTIGGWKFGVAKKTQLVAVKVLGADGSGSNSGVIAGMDFVARDATRRGLRGKAVMNMSLGGSFSQAVNTAINRIEAAGVVPVVAAGNENQDTALTSPGSAEAAITVGAIDQTNDRRASFSNFGPLVDIFAPGVRVESCGIRSDSDTATLSGTSMASPHVAGLAAYIMALEGITGVQQVADRLKQLAGQTNSRVVAASNVAGTTDLIANNGFR*

>pbla_17780_gene_e_gw1.4.721.1_pbla_scaffold_4:complement(join(1714758..17151621715257..17157781715915..1716256))

MAGRVLLCFTLALSALGVSGVPTSLPNKTEGEFLGIPISNPGVLNAIPNRYIVVYNNTFNDDDIDVHEKSVIQTIAKRNVAKRSLTGKLLSTTVDTYKIGKWRAMALDADDLMINEIFSAKEVSYIEQDAVININARAVQGRTTTGLARISHAQAGSRTYVFDDSAGEGITAFVVDTGIRVTHSEFEGRATFAANFIDDVDTDEQGHGSHVAGTIGGKTFGVAKKVNLVAVKVLGADGSGSNSGVLAGMQFVVNNATAQGLGGKSVMNMSLGGGASAAINAAINNIEAAGVVPVVAAGNEAQDTANTSPGSAEAAITVGAIDQTTDRIAEFSNFGRLVDVFAPGVQVLSVGIETDNSTKALSGTSMASPHVAGLAAYLMALEGITGVQEVGDRIKELAQGTGARVRGSPRGTTTLIANNGADQL*

>pbla_2206_gene_gw1.2.201.1_pbla_scaffold_2:complement(join(2746288..27465842746667..27471882747323..2747583))

MTIIFGSKISMSLLLAVQLSFWFIPETGSLVPPLTQKHPGNNATLENPPNLPRNKTSTPTKNRSTLRPVSKNAPATESHKSYIVLLNDQVEVSEFVSALKKSWASGSGSPLNENQVGYVYQEVGFKGFSAQLDDQAFKSLSRSAGVQEIIPDTLVSVHPIDEVISDALAVTGLGVEPSHNATVNAQGVPDHYSQKGTAPWGLQRIDQRKVITTHGANSAAVNYDYYYSQPAGEGVIVYILDTGARETHNDFGGRVEMAAQFGGYDLNDGNGHGTHCAGIVAGNRWGVAKKAQVKAIKVLADDGSGSTSDVIAGVQYTLQQYKASGYQPSVVSLSLGGDKNSALDRAVQAAINQGLHFVVAAGNSNTDACLSSPASVSGANVVTASDIKDNRASYGSWGTCVDFFAPGTDISSAWFSADDAVKRMSGTSMATPHVAGLIAAHLSRQNYTTQQMSDKLKRDATQNVIYLGPADSDPSSTPNLLLYNSLQ

>pbla_23437_gene_e_gw1.23.208.1_pbla_scaffold_23:join(573875..574182574243..574328574400..574526574594..574723)

MTIIFGSKISMSLLLAVQLSFWFIPETGSLVPPLTQKHPGNNATLENPPNLPRNKTSTPTKNRSTLRPVSKNAPATESHKSYIVLLNDQVEVSEFVSALKKSWASGSGSPLNENQVGYVYQEVGFKGFSAQLDDQAFKSLSRSAGVQEIIPDTLVSVHPIDEVISDALAVTGLGVEPSHNATVNAQGVPDHYSQKGTAPWGLQRIDQRKVITTHGANSAAVNYDYYYSQPAGEGVIVYILDTGARETHNDFGGRVEMAAQFGGYDLSDGNGHGTHCAGIVAGNRWGVAKKAQVKAIKVLADDGSGSTSDVIAGVQYTLQQYKASGYQPSVVSLSLGGDKNSALDRAVQAAINQGLHFVVAAGNSNTDACLSSPASVSGANVVTASDIKDNRASYGSWGTCVDFFAPGTDISSAWFSADDAVKRMSGTSMATPHVAGLIAAHLSRQNYTTQQMSDKLKRDATQNVIYLGPADSDPSSTPNLLLYNSLQ

>pbla_23527

RDHYHAANSLGEGPVGELQTYFLVSKRITDPGFPQQWHLVNQQYPGKDINVVDVWKQGVTGNGSTVVILDDGLDYESQDLAANFFAEGSYDFNDHTPFPKPRLWDDSHGTRCAGQIAAVNDVCGIGIAYNSRVAGVRILSGDITDADEAAALNYKYQDNDIYSCSWGPSDDGEKMEAPVGMLADAFQNGVKNGRGGKGSVFVFATGNGAASGDNCNFDGYTNSIYTITVGAIDHTDNHPPYSESCSAQLVVTYSSGGGIYTTNVCTNSHSGTSAAAPNAAGIFALVLGVRPDLTWRDMQHLCVQTAEPKDWKRLPSGYNHKFGYGRLNTLALIEAARFESVNIQTHLSAITVTEEMIKAAGLRLEHVTATVDIEHQQRGNIVINLQSPHMESELATERPRDLSPDGIRDWKFMSVKHWDENPVGDWTLLVYDKWTLTLFGE

>pbla_36687

IRDPGFDKQWHIINRDHRGHDINVAGVWSQNITGQNVVVAILDDGLDMDNEDLKDNFFAPGSYDFNDHTNLPKPKLFDDTHGTRCAGEIAAVNDVCGVGMAYGAKVAGIRILSADITEADEAAALNYKFQQNDIYSCSWGPPDQGEVAEAPKGIVLDAIKNGINNGRDGSGTIFVFASGNGGANDDNCNFDGYTNSLYTITVGAIDRLDRHPYYAESCSAQLIVTYSSGNGIYTTDVCSDRHGGTSAAAPLAAGVFALVLSVRPDLTWRDMQHLCVRTAVPDDWDVLPSGYNHKFGYGKLDAFAIVEAAKFKSVGPHTFLSVVAIDQERLDSVGGTLEHVTVTVDIEHGRRGDLEVFLESPNKVSKLGASRKFDNSKDGLVNWTFMSVKHWEENPVGDWTLRVMDQWSLTLWGE

>pbla_39049_gene_estExpbla_Genewise1Plus.C_120145_pbla_scaffold_12:complement(join(859892..860347860405..860926860975..861325))

MAIVKAFATAIIAALSLSVAAAPARDNAQNDKYIITLKSGISSRDVESHMNWARGIHDASLGRRALDLPGIEKRYDVADFHAYLGSFDKETLRKIMESPDVLGVERDAETIPMALTTQQNPPWALSAISSRTPGPQPYQYDDSAGKDTFAYVLDSGVNANHVEFGGRATIGYSGYSNNNPNKGVADRSGHGTMVAGLIASNTYGVAKKANIIAVQSQNSASALLDSMSWAVQDIQKQGRVGRAVINYSGGIQKFADSNPFVTESPGISMARIMETAFNQGILCVIASGNQGVVVEQSDAPYQGNSTSALVVGAVDEKWGHASFSNYGPSVDILAPGANVVTTTIGSDTATVTQSGTSLAAPHVAGLALYLITAENIKTAAELRARILALATKNKVTNVPANTVNLLASNRAQ

>pbla_41915_gene_estExpbla_Genewise1Plus.C_420045_pbla_scaffold_42:join(296454..296795296917..297438297558..297962)

MAILKAFTTAVIAALSLGVAAAPTQDSNGQKDKYIVTLKSGISSRDVESHMNWARGVHDASLGHRALDLPGIQKRYDFGDFHAYLGSFDKETLEKIMANPDVLGVEPDGIIMPLALTTQQNPPWALSAMSSRTPGPQPYRYDDSAGENTFAYVLDSGVHDKHVEFGGRAAPGWSAYEEDFPDRPHGDTSGHGTMVAGIIASNTYGVAKKANIIAVQTDHTVSGTLGGIAWAVRDIQSQGRVGQAVINYSGEVGLETIPDSAGYKYQPGVAMAQSMDIAFNEGILCVIAAGNDGQVVEQSSTPYQGNSTTALAVGAINQQWNFMRLSNHGPSVDILAPGENVMTISKDSDTATTVQSGTSLAAPHVAGLALYLIAAEKIKTPAELRARILALATKDKITNVPANTVNLLAFNGVQ

>pbla_58746

YVIMNAWPVYGTTVTRVRGFEGEGIKIGVIDSGDFLHPALGGCYGGCKVAYGYDFVPNPRDVCHGTHVAGIIGGIAPKVTLGAYKIFGCDVIMALEKAFTDGMDIINLSIGAIMADMIAVTVLASAGNGDQGFKVNVPALDGCVVIAGASPFSSWGLGLSIKPDISAPGGIFSTYPGTSMACPSGIVALGSPRQGGGLINIEKMTLIFNDKIKNDTKPVIYRFSHMPAEIPAKIYSGFIPYAGGIIKAGYYRLRIMALRVFGDY

>pbla_59777

YIVFRGYPVNGLSVNDVHNLTGKGITIGILDSGDYNHPALGGGFGGYKFKYGYNLVPEPFDPCHGTHVSGIIGGIAPDATFGMWRIFGCDTVIALEMAYEAGCDIINLSLGAVVADRLTVIVVGVAGNGNQGFMQNTPGSGKNSISVASVDNTGCLLVVGASAFSSVGPTLDLKPGVSGIGGVYSTLPGTSMAAPAGVTALARLDHPLRQGAGLVQPYTSIHISNDTVTNGKRPLCLTIENIPSTVSPGYPIYGGFIPYFGGKVSKGTYFLRIRALKLLGDW

>pbla_59778

YIVFHGYPVHEMTVNRVHGLTGKGVVVGIIDSGDYTHPSLGGGFGGFKVQFGADLSKGPLDTCHGTHVAGIIAGVAPDVTLGMWRVFGCDLVIALILAYEAVVAAAGNGSEGFMVASPSVADNVISVASVDNLACVLVKGASSFSSVGTSLDLKPNLAGVGGIFSLLPGTSMASPAGAIALALPDNPAKQGAGLIQVYDASYVSNDTITNEKEPMTYTIRHFSNTVQPGFPMYGGFIPYVGGSIKNGTYYMRWKALRLLASW

>pbla_61860_gene_fgeneshPB_pg.4__535_pbla_scaffold_4:complement(join(1712619..17130381713115..17136361713733..1714116))

MAILKAFTTAVIAALSLSVAAAPAQDSNGQKDKYIVTLKGGISSRDVESHMNWARGIHDASLGRRALDLPGIEKRYDFGDFHAYLGSFDKGTLGKIMDSPDVLGVEPDGITMPLALTTQQNPPWALSAMSSRTPGPPSLIDTGVHDKHVEFGGRVTPGWSAYEEDFPDRPHGDVTGHGTMVAGIIASNTYGVAKKANIIAVQTDQTVSGLLGGIAWAVRDIQSQGRVGQAVINYSGGLPTIPDSAGYKYQPGIAMAQSMDIAFNEGILCVIAAGNDGKVVEQSTTPYQGNSTTALVVGGINQQWDFMRLSNHGPSVDILAPGENVITISRDSDTATTVQTGTSLAAPHVAGLALYLIAAEKIKTPVELRARILALATKDKITNVPANTVNLLAFNGVQ

>pbla_62430

YVVLRGWPIAMLTANNAYGQNGSGIKIGVIDSGDYTHPALGGCFGGCKVAYGYDLVKEPIDSCHGTFVSGIIAGVAPGATLGMWRVFGCDILIAMEMAYNDGMDIINISLGSVVADRLVVHVVAASGNGTSGFLTAAPATGRNVISVASTSNDACVLIRGAASFSSLGPTLQLKPELTAVGGVFSTMPGTSMSAPAGSVALANPDSPIRQGAGLVNVVQAFHVSNDTLHNKSTPLKVKLSHSPSTIPPNHVIYGGFFPYIGGVYEPGVYRIRVRALNVFGDW

>pbla_64399

YIVFNGYPVHGMTVDRVHKKFGKGIKISVVDSGDYLHPALGGGFGGFKVQFGYDLVPDPLDSCHGTHVSGIIALLAFLMAYDAGMDVISVSIGTIVAQRIAVPFIVSTGNGSDGFTVGIPSIGKDVWSIASVENDGCALVQGASDFSSIDASLDIKPNIAGIGGIYSTLPGTSMAAPSGSIALAMKESPLLQGAGLVQVYDAVSVSNDSIHNGKARAVFKVINEPSTINSGHIMYGGYIPYFG

>pbla_64400

YVIFNGYPVHGMTVDRVHKIFGKGIKVGVIDSGDYLHPALGGGFGGFKVQYGYDLVPGPLDSCHGTHVSGIIAGVAPQATLGMWRIFGCDVIVALLMAYDAGMDIISASVGAIVAQRIAVPFIVSSGNGDRTFTVTAPSTSKDVWSIASAENDACALVQGASDFSSIGASLDLKPNIAGVGGVFSTLPGTSMAAPSGSIALAMKESPLLQGAGLVQVYDAVSVSNDSIHNGKARAVFKVINEPSTINSGHIIYGGYIPYFGATYPSGTYILSLRALKLLGDW

>pbla_65757

YVVFNGYPVHAMTVDRVHKKFGKGIKVGVIDTGDYLHPALGGGFGGFKVQYGYDLAQDPLDSCHGTHVSGIIAGVAPQATLGMWRVFGCDVLIAFLMAYDAGMDIISVSIGTIVAQRIAIPFIISAGNGSEGFTVGMPSTAKDVWSIASVDNDACALIQGGSDFSSVGASLDLKPNIAGIGGVFSTLPGTSMAAPSGSVALALKQSPLLQGAGLVQVYDAVRVSNDTIINGKARTTFKVVNEPSTLNAGHIMYGGYIPYFGATYPSGTYILNLRALKLLGDW

>pbla_66178_gene_fgeneshPB_pg.14__133_pbla_scaffold_14:join(446166..446283446429..447045447247..447359447551..447675447789..448248448370..448553)

MVGLKNVALFAASIILPASITWAAPIIEVETKPIPEKYIVLLKPHADLEGHLSWAKDVHARSLSRRDTAGVHKAWSVGSKFKAYAGEFDEETLKIIQRDERNVHSIEPDKSWRLYKSNKKDNDDSNSDNTTIITQKQAPWGLGYLSHKGKTSSDYVYNSTAGTGTYAYVVDTGCWKDHVEFEGRVQLGYNAYPDSPFIDMDGHGTHVTGTLISKTYGVAKNATVICVKVFHGGGSANTIVMDGFEWAVKDIIAKKRQRNSVINMSLGCDRSEAFNAIVDAAYDQGILTVVAAGNENQPAALVSPASSARAFSVGAIDNKNTRAYFSNYGAIVDIFAPGVNIVSTYIGKKDGDNNRTMTMSGTSMASPHVAGLALYLKSLDPEKYGNSSDAHSGLRALGVPDKVWDAGEMSPNLVAYNGVQG*

>pbla_77009_gene_estExpbla_fgeneshPB_pg.C_50262_pbla_scaffold_5:join(914502..914661914749..915133915226..915350915447..915566915668..916001916093..916267)

GEGATVYILDTGILANHNDFKGRVTIGATIVGDVDDPTDTNGHGTFVAGVCCGTTYGVAKKASIVSVKTLDDEGNGRLSDLLKGIEWVLQQHLLSNSTKSIVNLSLGAMRSQATNDAVEQAIAMGLHFTIAAGNYGEDACLYSPGSARGAITVGAIDEDDSVSYYSNFGKCVDIFAPGTNIRSTWNTGVQDTHVLTGTSMAAPCTILLYIYQACFL*

>pbla_77720

YIVFNGYPVHAMTVDRVHKKFGKGIKVGVVDTGDYLHPALGGGFGGFKVQYGYDLVPDPLDSCHGTHVSGIIAGVAPQATLGMWRVFGCDVLVSFLMAYDAGMDVISVSIGTIVAQRIAIPFIVAAGNGADGFTVGMPSTAKDIWSTASVDNDACALVQGASDFSSVGASLDLKPNVAGIGGIYSTLPGTSMATPAGSVALALKESPLLQGAGLVQVYDAVRVSNDSIYNGKVRATFKVINEPSTLNPGHIMYGGYIPYFGATYPSGTYILHLRALKLLGDW

>pbla_77747_gene_estExpbla_fgeneshPB_pg.C_90303_pbla_scaffold_9:complement(join(1057171..10574061057532..10576531057749..10580371058107..10582281058297..10585921058731..10590201059120..1059243))

MYFTLISCILYVHLSMAIAVNVKKYVAPTVTELKDTEHYVIIFKPNVSTTKINNQIKRIKLHQTNQTSTINSTISNHTLSSKKQAVQYSTIGNFKWYYAQFHTTSLENYLSTNADVDDTVHYWAKDAQFSLQEFIQTNPPSWGLDRIDQRIGTDGEYRFASSQGAGVTIYLMDTGIRQDHSDIDGRVTIGKTVVGDTSFDNNGHGTFVAGVCCGTKYGVAKKANIVSVKTLDDEGNGRLSDVLVGLQWIVEQHISAFYSQPTNDAITEAIGLGIHFSIAAGNYGEDACRYSPGSTPGAITVGAFDEDDSVSYYSNFGKCVDIFAPGTNIKSIWPTSKDATHKLTGTSMAAPHVAGAMAIFLSENDYTPLQLATYIKNTSTLRTEDFTINNTGIYYSENKTVVDNAIDMGYQVENLMNQKTRVNLLYSHPSDGKPFWVYGRTLNAATNHLLSFSSSAYTTVFILTMSVTLLVLL

>pbla_79238_gene_estExpbla_fgeneshPB_pg.C_240043_pbla_scaffold_24:join(141294..141432141567..141595141663..141717141801..141994142067..142337142477..142585142654..142738142815..145796)

MTSISKYIQEYPVNGLMPKQETQAASFIKKYPNYDGRNTVIAILDTGVDPGAAGMQITTDGKPKIIDIVDCTGGGDVNTSAIVKAQTEEDGDKLTYIQALSGRKLIIDPTWKCPSGEYHVGVKPAYELFPTELVNRLKKERSKAFDARHLHLVDEAKARVCSFTKQNSSKLTSDPVVAAELADLETRVEVLKGLKDKYEDPGVILDCVVFNDGQDWRAVIDLQESGDLRGQPCLTDYRKEFKYHTFGETDLLNFSVNIYEDGNLLSIVTLAGSHGTHVAGITAANFPEEPALNGVAPGAQIVSLRIGDARLGSMETGPGLTRAALHLAINKVDLANMSYGESSASPTDGHFIKLLAEEAIAKSGCIFVTSAGNDGPCYSSIGAPAGMHSSFITVGAYVKHSQMQAEYALLESMTEQPYTWSSRGPCTDGYSGVDIYAPGSAITSVPVYGLHKLDLKNGTSMSSPNACGCVSLLVSGLKAEKRDYSPYRVKAAVVQSAKSVKDPLNVGFLQVEKAWEYLDTYKSRNDQDIAFEITVSKRGPQRGIYLREREETNQVQYLPVTVKPTFMAENNVEDPKYNRAKFNYEARIALVATESWISAPDYLYLHSGGNSFQVKVDPLALKENEFHFGEVLGYDTTAPDRGPVFRVPVYVVKPVLASHGSLEYKKIAFGPGDIVRNFVHVPEGAAYCQLTIRSKSLVNTVPARFMLHLLQLIPKKSQKNKQTYSFVLGSGTYSNSDSEEEVIVKRFAVRGGLNLEVCLAQFWSALGKHTVDLSLEFHGVQLAGNLANGHGVVYFDPQVTRLDVAAPIRKENKVDISVSFNKLRKYIRPYESEIAPLYADRDMLPDKTLLYALVLSYKLKVDASNTMVVRFPTVMNQLYEHYLAGVFGIVYDANCKVIGYLDVFDQTIKLDNKGEYTIRLQLMTEEQDVLEKLKNTVCQVDLDVKAVTFNVHKSMADVYRSDKSTCPKVTLERKDTKALFVAAPLGELPKEAKHGDALVGKISFTSNRVDGGQYSAVYPVPPTISEPKKKEDKADKPDDEKLKQNLEDAIRDLQISYLKKLSSGSDAFKSVLSHLEENHGKSIGLYEQKIEAIWTVAGGRNENIIAVPSKLTQEQAKEISSLADSILEQINQNELFAFFGRQKSQEETDEAKEKRKENEKKKSQVIKALKNKAAAWAAVTESKDQDSEFETCLTELNQLASDDSNSDITSLLIKVKKERLAGRFGNALKAVQAYVSESSVAPDSIKNLQKAWELRRLLFEDLEWSIWAENDAKLKTIRAPPGGYAPF*

>pbla_79866

YVVFSGYPVHAMTVDRVHKKFGKGVKVGVVDTGDYLHPALGGGFGGFKVQYGYDLVPDPIDSCHGTHVSGIIAGVAPQATLGMWRVFGCDVLVAFLMAYDAGMDVISVSIGTIVAQRIAIPFIVSASNGADGFTVGMPSTAKDVWSVASVDNDACAIVQGASDFSSVGASLDLKPNVAGIGGVFSTLPGTSMAAPSGSVALALQENPLLQGAGLVQVYDAVRISNDTIYNGKVRTTFKIINEPSTLNAGHIMYGGFIPYFGATYPSGNYILNLRALKLFGDW

>pchr_130748

VHEPFGFMRIALQQDPEGLTDALSTPGSARYQKFLVSAFTAPSKEASDSVISFLDWLGFVEKANEMFGADFHVFVTMEYSVPTDLSPHIQLVHPSISYTPACVQDLYNKLGVAGFIQQFANQADLKSFLFTLQTLGGNTQEADLDTQYTIGIATGSFVSCGNNVGGMDWANFINGLASPPQVVTTSYGENENISAANSLCNLYQQLGAKGVSVLFSSGDGGVGQSFLATFPSGCPFMTSVGAETSSSGGFSNIFAQPSYQAVSAFLKFNPAGRGFPDVAAVGVEINFQQVAGTSCSSPIFASIISLINDRLAAQGKGPLGFLNPFLYFTDITTGGCGFPAEAGWDPVTGLGTPFAAL

>pchr_133020

VREPGGYLRIALQNNPDGLIDALSTPGSASYGEHLVEKFVAPTAQSSEAVNAWLDWLSVVSKANEIFDADFAVYRTMSYSIPASLEGHLDFVHPTISFTPACIESLYNTLGVSGFIDQFANQADLTTFLFTLQTLGGNPQEANLDIQYTVGIASGTFISVGLEGLDIINFLLNESNPPHVLTTSYGDNESISLANNLCNAYAQLGARGTSILFASGDGGVSQSFVPTFPSGCPFMTSVGAETASSGGFSNYFATPSYQVVSSYIKYNASGRAFPDVAAIGLEIVVDGVDGTSCSSPVFASAIALINDALVAQGKSPLGFLNPFLYFNDITSGGCGFKAAKGWDPVTGLGTPFAAL

>pchr_133398

VHEAAGFLRIALQSNPERLIDALSTPGSPSYGQHLVEALVAPSTETVSAVEEWLDWLSIVSKANELLDADFNVYRTMRYSVPPELEGHLDFVYPTVSFTPACVYTLYNKLAVSGFIEQFANQNDLATFLFALQTLGGNPQEANLDIQYTVGIASGTFISVGEQLGGLDIINALLNENHPPQVLSTSYGEDEPISLANKLCNAYAQLGARGTSILFSSGDGGVSSSFVPTFPSGCPFMTSVGAETASSGGFSDIFAAPSYQAVQTYLKFNPAGRAYPDVAAIGLGIVVDGVDGTSCSTPIFASIVSLINDELIGKGKSPLGFLNPFLYFFDVTTGGCGFPASKGWDPVTGLGTPFPAL

>pchr_133613

FVGHPVHVMTVDKAHGLTGAGIKIGIIDTGDYNHPFLGRGIGGHKIIGGYDFVPDPLDECHGTHVAGIIGGVAYNASLSMYRVFGCDIIIALLRAFNDGNDVLTLSLGGVVASRIAKVVTIAAGNGAYGWYTSSPGTGKDVISIASIDNDACVIVRGGSSFSSYGPTMYFKPAVAAPGGILSTYPGTSMATPAGSAALAAPQTLAQQGSGLIQVDKATVISNDTIKNGTKPLTYRLSHVAAVPPGLPVYSGFIAYLGGDGTYKVLVRALKVNGDY

>pchr_133799

FIVFVGRPVHVITVDKLHGITGKGIKIGIIDTGDFTHPDLGGGIGGFKIIGGFDFVPDPLDQCHGTHVAGIIGGVAFDASITSYRIFGCDVIVALLRGVSEGQDILTMSLGSVVSSRISKIVTIAAGNGADGFFTSGPGNAIDAISVASLDNDACVIVRGGSSFTSYGPTMFFKPAVSAPGGILSTFPGTSMATPAGVSALAQLQTVAQQGAGLVNAFQAIIITNDTIKNGKTAESFKISHVPAVHPGLPVFSGFITYLGPNGLYKILLRVLKVTGDF

>pchr_138363_gene_e_gww2.4.218.1_pchr_scaffold_4:join(1200933..12009711200972..12013221201344..12013761201431..12019411201997..12021311202184..12025991202600..1202602)

MKLLRLISICLLTRIGYSLVIPSIWAQTGKSPGVFNIDSDSPSNIIHHAASPIPNQYIVLFKSDTTDIQRETHLATLNSWLLNTDPQYTILHSFQLAHDLQGYTSRLPEQLIRSLATDPIIQSIEQDSYVHALGRQKQIDSPWGLARISQRERLKLLQDQFYHYDDSGASSNTKCYILDTGINENHNEFQGRAKWGAVFTPMEPNQEDENGHGTHCAGVIGSHEYGVAKNTTLIAVKVLDARGDGEMSAVIRGIEYVTQQHEQDMKKQDQTGFKGSVINLSLGAGKSPALLRVIEAAVKLGVHFAVAAGNEDDDACSTSPADSPHALTVGASSFSDDRVFFSNWGPCVDVFAPGINIMSTYIGPHNNETTSLSGTSMSAPFVTGLIAYFLSLQPDTKSQFYTGITSPTPYQLKYKIISFSTEGVLHEIPEGTPNRIVYNGGGHSLKDFWNN*

>pchr_26825

PTGWMRISLQDRLEDLITALSDPAHEKYGQHLAEALVAPHEDSVELVDAWLSWLTIVEQASRMMNATYNIYRTMSYSLPSVLHGHVGVVTPTTYFTPACLRALYNKLGVAGYLGEFANDADLQTFFFQHVQVGGNNQEANLDIQYTEGMSFPIYYSTGGSNEPLDWLNFILAQTTVPQTFTTSYGDDEQVPYATEVCNLFAQLGARGSSIMFSSGDDGVGCLTNDGFQPNFPASCPFVTTVGAEVASGGGFSNYFAQPSYQAVSAFLLFNTSGRAYPDVAAQGFQVIIGGVAGTSASSPTFAGVVALLNDFRLSQGKSALGFLNPIIYFNDITSGGCGFSAGAGWDPVTGLGTPFGKL

>Pchr_g03930.t1

LVEPDGWFRLAIQHNAREFEQKVSTPEHPSYGQHMVKQFLTPHPAISDRIIAWLHWVSFIFQAERMLKTQFFYFRTLGYSVPRALRSHIQLIQPTTLFTPDCLRDLYNRLGISGFLEQYARHDDFNEFLFSVVSIGGDGQEANLDVQYSIPLADETFYSTGGRNEPLEQLHYLLDLPDEELPAVLSNSYGENEQLPYLNATCSLFAQLGARGVSILFGSGDSGPGCVKNDGFLPGYPASCPFVTAVGGEKASGGGFSEVFSRPQYQAVKGYLLYNATGRGYPDISAQAFIVRDQGVGGTSASTPVLAGVISRLNAARIAQGKPRMGFLNPWLYFTDIVLGGCSWDATEGWDPATGLGTPFPAL

>Pchr_g14680.t1

VFEPQGWLQIALQGDTAGFEEAVSTPDHPSYGNHFMKRMLQPSAESADSIRDWLDWMTFVETANELLAANFQFYRTLQYSVPEALMPHINMIQPTTRFTPQCLKNMYNKVGFASYLEEYARYSDLELFEFSVIQYGGNDQEANLDLQYIVGVSSPTEFSVGGRNEPLEFLQNVLKMEQQDLPQVISTSYGENEQVPYARTVCNLFSQLGSRGVSVIFASGDSGVGCQTNDGFPAQFPAACPWVTSVGAEKASSGGFSDLWDRPKWQAVSDYLLFNPKGRAFPDVSAQGYAIYDKGVDGTSCSAPAFAGVIALLNDARLKANKPPMGFLNPWLYLNDIVHGGCSWNATKGWDPVSGLGSPFATM

>Pchr_Wisconsin_541255:Pc21g14160.t1_gene_Pc21g14160_Pchr_Wisconsin_541255_contig_Pc00c21:complement(join(3343256..33435003343575..33442203344291..3344596))

MKIIRLASVATVATLAHSLLIPSLDQLNIQLPFSLPHHTESPSLKLQGSNPFESSTVRPDPIQIYSTGYKVIENSYIVTVDSSITDSELQQLYDYIKGGYEFMLNNEDPFFVAMGIKRLPKHSLFEPFSIGDSMKGFVGFFPPRLVEKLASMDLPIVAIEADSVVHSTKEYAVDEHVTWGLARISQRQTLDTNRNYIYHVDGGKNVTAYVIDTGIFVDHEQFEKRARWGATIPFGDVDKDANGHGTHVAGTIGSAKYGVAKQTSLVAVKVLNSDGTGTLSDVIKGIEFVVKDHKSSRGKSKGSVANMSLGGGLSVALTYAVNAAVDNGIHFAVAAGNDNANACSYSPANSAKAITAAASTVEDERAFFSNWGTCVDVFAPGYLIESTYIGLPTSTRVLSGTSMASPHVAGLLSYYLSLQPGHESQYNSGDSLTPAQLKARILSFSTKDVLDDSDLNYGTPNLLIYNSRDNITEFWDY

>Pchr_Wisconsin_541255:Pc21g16970.t1_gene_Pc21g16970_Pchr_Wisconsin_541255_contig_Pc00c21:join(4002814..40031824003248..40041744004230..4004418)

MQFFTRIAALAVAAAPFLNAAPLKPDTDVASIAAHHNKVRSIYARNLARRGEDGTSGSPVEREFGFGDFKAYAGSFDEATIEELKAMPEVLDVEQDFIMRTHAIVTQENAPWGLGSISSRTPGASSYVYDDSAGDGTFTYIMDSGVRVSHADFEGRATWGINTSSGSQAADDDGHGTHVAGSAGGKTYGVAKKTNIIAVKVIQGSTGSASAVFAGFDWTVNDIVSKGRQNTAVINMSLGSTASTTWDAAITAAWNKGVLAVTSSGNAESPAAEYSPCRSPEIICVGNIEITNKRHGGPGGSNYGPEVDIFAAGTNVTSASYLSDTGSARQTGTSMASPHVAGLISYIRALEGPMSAAAVKARLFELATLDVVTDPKGSVNRLAYNGNNRR*

>Pchr_Wisconsin_541255_Pc22g029

RSQDFSPAQVLGEGQIGQLDGHHTFSKRITDPIFREQWHLLNTLQPGHDLNVTGLWLEGITGKGVVTAVVDDGLDMDSNDLKPNYLPEGSYDFNENVPEPRPLLLDDKHGTRCCGEIAAANDVCGVGVAYDSKIAGIRILSKPIDDVDEAAAINFAYQTNDIYSCSWGPIDDGATMDAPGILIKRAMANGVQKGRGGKGSVFVFAAGNGAAYGDNCNFDGYTNSIYSITVGAIDREGNHPSYSESCSAQLVVAYSSGSGIHTTDVCFSGHGGTSAAGPLAAGSAALALSARPDLTWRDLQHLMVETAVPDGSWQVLPSGFSHDWGFGKVDTYTMVQLAKWELVKPQAWLHSSRYTVTADQLKEANAKLEHVTVTMNVNHTRRGDLSVELRSPAGVSYLSVARRKDDMPVGYDDWTFMSVAHWGESPVGDWSIIVKDDWRLNLWGEAVDGTKQIKRRRYEFLYNAF

>PGTT_08683

RSYAYSGADRLDVERVGELENFWLIKKRIYDPLWPKQWHLVNDVIHKHMINATGVWEMGITGKNVTVAIVDDGIDMSSDDLKSNFFEAGSWDYNDHTPLPEPRLPDDLHGTRCAGEIAAVNDVCGVGVAFDGKVAGIRILSASISDADEASALNYGYQENHIYSCSWGPPDDGKSMEAPSRLIFKAMLNGIQKGRGGKGSVFVFASGNGGAVDDQCNFDGYTNSIYSVTISAIDRQGLHPYYSEVCSANMVVTYSSGSGIHTTDVCTDRHGGTSAAAPLGAGIFALVLQARPDLTWRDVQYLAVTTAIPPDWQKTASGYNHKFGFGNMDAYQIVQAAKWKLVKPQAWWTSATIVVSQADLDGANESLEHITVAVNIKHTRRGNVRVLLISPHGVSILAAHRRYDDASTGFPGWVFMTVKHWGENPVGAWTLSVQDDWAMGMWGECKDPTIQRMRRRF

>PGTT_10045_gene_PGTG_10045_pgra_supercont2.29:join(75290..7572475816..7617076241..7637376471..7668676765..7707077182..77197)

MLFFTRVTAFVAAAAPFFANAAPVAAEQRTNEVIPGKYIVQLRSDADVAAVAAHHNKVRDIYARNLARRGDDGSAGGAPVEREYGFGEFKAYAGSFDEATVEELRGLPEVISVEQNFIMRSTGFVTQASVPWGLGSISSRTPGSTSYVYDDSAGKGTFSYVIDTGVRITHNEFEGRAIWGFNAVSGSPDTDDDGHGTHVAGTLGGKTYGVAKKTTIIAVKALAGDTGSASDVFAAFDWTVNDIVAKNRQNTSVINMSLGSSASSTWDAAITAAWNKGVLVVTTAGNENSLASQRSPARSPEVICVGNVRIDNQRLGGPGGSNYGDAVDIFAAGTNVLSADMASDSATRVRNGTSMASPHVAGLVSYLRGLEGPMSARDVKARVYALATPGVVTDTMGSANLLAYNGNK*

>PGTT_10542

YLVFCGSRPHIQTISKLHGIAGQGVKVALIDTGDCTHPAFGNGFGGFKIGFGRSFVSEPCTRCHGSSTAGILAGVAPSINLGMYLVGCDVIIAMLQAQKDAADIISISLGRVANKLVQVIMVASAGNGHNGFIGESISSSKNVIAVGSVDSDACVLIKPGGVGGILATLPGTSMAAPAGIIALAAVVHQGGGLIDAFCATTISNDSISNGTSFVDYVLSHRPALSPGLAVYSGYIPYY

>PGTT_10975

HAVFSGRPVHVQVISRLHGYRCKGINIAVIDTGDCQHPALGKGFGGFKIAKGYDFVPDPCTSCHGTHIMGVLAGVCPDATMSSYRIFGCELVAALLRAYKDGADVFSLSVGAVIASRIARAVVVSAGNGDEGFFATSPSTGTGAISVGSVQSDACALVRGTPQSVMQGTSISAPAGIAALATPECLVNAWCATVVSNDTITNGKQSVSYTVEHVTALRPGLPVYSGFVPGKIPDGKYRILVRALRVTGDY

>PGTT_12076

FIVFYGKPIQIQTVSELHGVFGEGIKVAFLDSGDCDHPALGPGFGGHKIGFGYDLVPDPCTQCHGTHVAGIVGGVAPNATLGMYLMGCDIVMALLMAVRDGADVISASIGDLINNLVSVALVLAAGNGDEGFYAETPAAATNSIAIGSVESDACVVIRSNYSQYGPSSMNIPNFLGVGGILSTVPGTSMSTPAGITALAQQQTTIHAGGGLVDAFCATVLSHDLITNGDQSYSFRTGHIPALAAKLPVYSGYVPYYGPSGYYKILIRALRVNGDF

>PGTT_12109_gene_PGTG_12109_pgra_supercont2.37:complement(join(707485..707500707612..707917707992..708207708311..708443708514..708868708960..709394))

MLATKLLLLAASTQALLIPNLEALSSFPQILRTELETSKEQAPFFAGDDTVAPLLMAKPSLSGIPGRYIVVLKDHASTEDISEHLNWLEALPANFKVDPLESEYLKSYGLTRDIVSFNVEGLKGYSGFLDEGRVQEVRNHPLVKFVEQETVVKLTEFDVQKDATWGLARVSHRELEPSTSYYYDNEGGKGVTAYVIDTGIKVDHEEFENRASWGSVLPFPYFKFDDNGHGTHCAGTIGSKTYGIAKNVDLVAVKVMNALGSGVTSDIIKGIEFVVNAHKELVAAKKKGYKGATINLSLGGGELTALDLAVNAATAAGVHVAVAAGNDNQDACSLSPARATGPVCVGASNNADDRASFSNWGSCVDIFAPGEDILSTFTWSETTLMSGTSMATPHVVGLLSYFASLYPDINSEFSESLISPALLKLKLIKYGTKGVLNNLDKASPNILAYNGAGGNLTDFWSL

>PGTT_12163

LLVFYGRRAQVQSITELHGIYGKGVKVALIDSGDCSHPALGKGFGGFKIAFGKNYANHPCTQCHGTHVAGIVAGVAPNATLGMYLFGCDSLVAMLQAHKDGADIISASIGTTVNKLVQAIIIVAAGNGSEGFFGDNPASAKNAISVGSVEADACVLVKGGSNFSQYGPSFESPPAVAAVGGTYPGTSMATPAGVAALDSAVHQGGGLIDAFCATTISNDSIFNGTGVFNYILTHRPALPPGLTVYSGFIPYYGPNGNYKVLLRALRVTGDY

>PGTT_14692_gene_PGTG_14692_pgra_supercont2.55:complement(join(246542..246625246711..247260247326..247494247564..247745247811..248125248210..248562))

MQLQLLLPLLVAQQAAALVLPNLNLFDFPDVLPISQKEESLDGKQKPLLPPQSTGSKPTAKLIPNKYIVVLKEGLSQQEIAAHHSWATETLASLVKLGEDQLVSFLIDAFNGYLAYMPPAFVEMVKKLPVVDFVEQDLVMHVNEFDVQKDAPWGLARVSQRELTTPSVDYLYDTEGGKGVTAYIIDTGIKTEHPDFEGRAVWGDAIAFPKLKVDAHGHGSHVAGTIGSKTYGIAKNVDLVAIGVMNLLGSGTTSDIIKGVEFAAKDHQSKLSAKQKGYKGATVNMLIGGGASDALDLAVNAGINAGLHIAVAAGNEDQDACEVSPARALGPITVGATDNADGKASFSNWGSCVDIQAPGVDILSVGIWLDTMVMSGTSMAAPHITGLLSYYLSLQPDLSSEFSTGLVSPQELKRRLIRYGTKNVVSGLDAASPNVLAFNGAGGNITDFWK

>PGTT_15038

LFEPRSFLKIALSGGMDLIKKRLSDPESSSYGRHLIRDLSAPSSSSLEAIVKWLDWVSLLQKAEEMLDTTYFYYRTERYSLPEEIHSHVELIQFTNACLRELYNRIGVTAYIGERANYEDLKDFLFTVVSVGGNPQEGNLDIQTTMGFTAPIFYTTAGSNEPLDWLLYIASQPDSAVPQVISTSYGDDEQVPYAKRVCDQLAALTARGVSLIFSSGDDGVGCLSNDGFMPIFPATCPYVTSVGAEEASGGGFSEYFDRPQYQQVQTYLLYNPKGRGVPDVSAQGYLMTWQKVGGSSAAAPTFASVIALLNDNRIARGMPALGFLNPWLYLNDIVIGGCGFAAAKGWDPVTGLGTPFPAM

>PGTT_16096

HAVFSGRPVHVQVISRLHGYRCKGINIAVIDTGDCQHPALGKGFGGFKIAKGYDFVPDPCTSCHGTHIMGVLAGVCPDATMSSYRIFGCELVAALLRAYKDGADVFSLSVGAVIASRIARAVVVSAGNGDEGFFATSPSTGTGAISVGSVQSDACALVRGTSPFSNYPQSVMQGTSISAPAGIAALATPECLVNAWCATVVSNDTITNGKRSVSYTVEHVTALRPGLPVYSGFVPGKIPDGKYRILVRALRVTGDY

>PGTT_17142

YLVFYGRRAQVQSITELHGIYGKGVKVALIDSGDCSHPALGKGFGGFKIAFGKNYANHPCTQCHGTHVAGIVAGVAPNATLGMYLFGCDSLVAMLQAHKDGADIISASIGNTVNKLVQAIIIVAAGNGSEGFFGDNPASAKNAISVGSMEADACVLVKGGSNFSQYGPSFESPPAVAAVGGGTSMATPAGVAALDSAVHQGGGLIDAFCATTISNDSIFNGTGVFNYILTHRPALPPGLAVYSGFIPYYGPNGNYKVLLRALRVSGDY

>PGTT_17496

VHETLWLKFGLQSNLDTLHDELSHPDSSSYGHHWVKEHFSPSAQAIDEVTAWLTWINILSEAENLLDTKYHLYACEDYKLPERLFRHIDMVMPTVHFTIECLKALYHTLAIVEYTPQSVIYKDMDLFFPNLIPIGGELNESNLDLQYSMPLVHPSLYQVGDFNNLDALDSKRDCGIVNPANVISTSYGMNEAATYLIRQCNEYGKLGLMGTTFLFSSGDNGVACLSRDGFNPSFPGTCPYVTSVGAEVASGGGFSNVFKMPNYQAVQNFFRYNSSMRGFPDLSANGYVVAVEGVYGTSASAPVVASMLTMINDARISVGKKPVGFINPAIYFNDIKDGGCGFSAVTGWDPVTGLGTMFTKF

>PGTT_18576

FYGFPLHVQTITDLHGILGQGIKVAIIDSGDCAHPALGGGFGGKRISFGIDLVHGTHVAGIVAGAAPKAEIGMYLFGCDIILALLMAYKDGADVINLSLGDVINTLVQAVIIASAGNGAEGFMASNPASSKNAISVGSVDADACVIIRGASNFSQSGPSFLSPPALSGVGGATYPGTSMAAPAGVAALARVESTVHQGGGLVNAFCATSVSNDSLVNGTQPVTYRVEHLPAVMPGLHVYSGYIPYYGPGGKYKVLLRALKVTGDF

>PGTT_19401

LLVFYGRRAQVQSITELHGIYGKGVKVALIDSGDCSHPALGKGFGGFKIAFGKNYANHPCTQCHGTHVAGIVAGVAPNATLGMYLFGCDSLVAMLQAHKDGADIISASIGTTVNKLVQAIIIVAAGNGSEGFFGDNPASAKNAISVGSVEADACVLVKGGSNFSQYGPSFESPPAVAAVGGTYPGTSMATPAGVAALDSAVHQGGGLIDAFCATTISNDSIFNGTGVFNYVLTHRPALPPGLTVYSGFIPYYGPNGNYKVLLRALRVTGDY

>PGUT_00804_gene_PGUG_00804_cgui_supercontig_1.1:complement(1411207..1412517)

MQPTSIVASFTFIVTLACGFLFPGYGAVLETMNKLLPGKYHQQIAPADPTFEEYKANLINLNKVVPHHYIVIFNEDLSQSQVEQYTQWLQVEYNNMLTIVDAAPKEVQVLKKLEFFNVEGFFRGFTGFFTADVISKIKNDPHVKLVEQDAIFKTNEFDIQKDAPWGISRVSHRENEEERKYFYDNDGGKGVTAYVIDTGIKVEHEEFEGRAQWGDAIAFPKIKIDGHGHGTHCAGIIGAKTYGIAKQVDLVAVGVMNLLGSGTTSDIIKGVEYVVNRHQEDVKSRKAGFKGSVVNMSIGGGVSEALDLAVNAAVKAGLHVAVAAGNDDADACQYSPARAVGPITVGASNIGDAKASFSNWGQCVDVFAPGEAIESTFIYSDSAEMSGTSMASPHIAGLLSYFLSLQPEQSSEYFTGLIEPAALKTKLIKYGIKGVLTGLDATTPNILAYNGAGGNLTDFWNI*

>PGUT_03723

KDHRYSSADFITEHPVRGLDDHFVFSKRINDPTFKEQWHLINTFNPGHDVNVTGLWYEGITGKGIVSALIDDGLDYESEDLKDNFNMKGSWDFNDNRNLPMPTLYDDWHGTRCAGEIAAVNDVCGLGVAYESNVSGIRILSGPITAEDEAAAMIFGLDVNDIYSCSWGPTDNGKVVSAPNKIVKKAMIKGVQDGRDKKGAIYVFASGNGGRFGDSCNFDGYTNSIYSITVGAIDYKGMHPIYSEACSAVMVVTYSSGSGIHTTDICSAIHGGTSAAAPLAAGVFALVLQSNPDLTWRDLQYIAALSSIPDGNYQDSALGYSQRYGFGKLDAYGMAHFAKWKNVKPQAWYYSSVIDVTEHDMKVSNERVEHVTVTVNIQATFRGKIGVRLISPRGISDLATERRGDRSMSGLKNWTFMSVANWGEKGTGNWTLEVDWSLRIFGESIDPAKARRYEFLFDEL

>PGUT_05640_gene_PGUG_05640_cgui_supercontig_1.7:824579..826402

MLPTSAVYTTFLLFATASHALLLPGFDSLFSLINYLPFQSNNKQTYLKQDEVNYEEYKAKLISNKFKKIVPDHYIVVLKDGLSKSTLMQHTNWVSNEYATMMTAATESAGVFSNSKPQLMKALEFFQVDNFISGYMGYFTKDMIQRVMQNPDVDFVEEDSIFKVNEFDVQKNAPWGISRISRRENLTGGGADSDLKYLFDNEGGKGVVAYVVDTGIMVEHEDFEGRAVWGEAVAFPKIKKDGNGHGTHCAGIIGSKTYGIAKSVDLVAVGVMNLLGAGSTSDILKGIEYVVNQHQNDVRTKKKGFKGSVINMSLGGGASDALDLAVNAAVRAGVHVAVAAGNDNADACDYSPAKADGPITVGASNIVDARASFSNWGTCVDIIAPGENIDSTYIWSDHTAMSGTSMAAPHVAGLLSYYLSLQPEQTSEYYTLVEPADLKKRLLKYGTKGVLTDVQGSPNILAYNGAGGNLTEFWSN*

>PGUT_05905_gene_PGUG_05905_cgui_supercontig_1.8:complement(410048..411433)

MKITTTLSVAAVASTVSSLVIPEISSFFSNINQILIPQQKQVPLVNQDYQESTQKQQQTDDEYIPGLLSNPLNTIIPDHYVIVLKDGLSESQISQHNNWIQTEHVAMMAVNTNQKGMTKPLEFFQIDNFVRGYTGFFTENLIARILENPDVNFVEKDSIFHTVEFDIQKDATWGISRISHRENTNDGQYLFDDDGGEGVTAYVIDTGIKVDHPDFEQRAEWGASIAFPNIKQDYNGHGTHCAGTIGSKTYGIAKKVNLVAVGVMNMLGAGSTSDIIKGIEFVVNQHQNDVKSKKKGFKGSTVNMSIGGGISEALDLAVNAGTKAGLHISVAAGNDNADACQYSPARASGPITIGASNVRDEKASFSNWGTCVDLFAPGEDITSTFIWSDTTVMSGTSMASPHIAGLLSYYLSLQPDVSSEYYTQAIDPQTLKNKLIKYGTKGVLTGLDSTSPNILAFNGAGGNLTDFWNL

>Pmar_007090.t1

LHEPARWMRIGLQSNLEEMYQHLSHPDSANYGKHWVIKAFQPSDKTVESVKTWLGWLAFPDEAERLLKTEFHEFACDEYHVPHHLKEHVDFVTPGIKLSPACVAALYNSLGIFESELQFYTQQDLDLFFPIAANVGGQSTEVSLDLQLAYPIVYPTDYEVDDFNDKLDQTYPELMCGVYKPTNVISVSYGGQEVLPYQRRQCLEYAKLGLQGVSFLFASGDSGVSCIGFNPTWPNTCPYVTNVGAESASGGGFSNVFPIPDYQAVATFFIYNRIGRGVPDVAANGIMVYNGGSGGTSASTPIFAAVINRINDERLWAGKSPLGFLNPSLYLNDITNGGCGFSAVPGWDPVTGLGTPFPKM

>Pmar_010230.t1

VHEPASWVRIGLQSNSDVGHDLLSNPNSPNYRKYMVNDFFAPSEKAVSAVRTWLQWLQFADEVERLLGTEYYIYGCEEYHVPKHLSEHIDYITPGVKTTPDCIRTMYNELGIFEDLGDYYAQEDLDLFFPTLKGIGAAPAESDLDFQIAYPIIWPVLFQTDDADPPPDPAANSLQCGVYKPTNVVSISYGGDEILSYQKRQCDEFKKLGLQGVSVVVASGDSGVACQGKSGFNPDFPAGCPYITTVGAEVAASGGFSNIYPRPAYQAVNTYLLYNAAGRGYPDVAAVGVLIYNAGIGGTSASAPVFAAILTRINEELLAKKGTTVGFVNPTLYFHDITSGGCGFKTAAGWDPVTGLGTPYPAL

>Pmar_012620.t1

VVEPDGWFRLAMQPRAGEFEQHVATPGHKMYGKHMVKAFMQPSAEVSDAVVSWLDWAKFLHQAEKMLNTTFYIFRTLQYSVPREIHQHVRLIQPTTHFTPDCLRDLYNKLGISGYLEQYARYDDLKAFLFTVQEIGGNDQEASLDMQYGISLSYQIFFSTGGRNEPLEQLHYLLNLSDEDLPAVLSTSYGENEQLPYTNTTCSLFAQLGARGVSIIFSSGDEGVGCLTNDGFNPIYPASCPFVTSVGGERASSGGFSERFPRPSYQAVASYLLYNPGGRGFPDVSAQAYLVRDHGVDGTSASAPTFAAIIADLNSVRLDNNQSILGFLNPWLYFTDIINGGCSWNATPGWDPVTGLGTPFDSL

>Pmar_ATCC_18224:PMAA_005820.t1_gene_PMAA_005820_NW_002196667:complement(join(1468162..14687881468850..14691681469232..1471123))

MAVASDSSGSLTDSSLLLNTAKTLHEVAKLLRSDGVSRESKKFYTNFMAHCVLIRHHVNRVKSSIPEDYRLLVGILNRFEAIVTSSILDTRPLSPANIYPRLRALNQAWNKVRYDSDDNKRLFIQKSVDFVCDTDQPDKLNEFLEGWEDEITEQYPDDPSLWADDHVPQRKSRGQPSYAVWNASQSLFRALLESKECECDPMHEVGVRLFLSTFRKLPSNERDPADSDYDFEIFLSVQKEWHEANVHVAKTGTAIRFTKEDPSKKKLPAMKVKNLCEPIKKRKQFDRIKLRVDDDGILWKLRSEKCCFAIDESKPPVSLQQFIQDQYRSLTDKTKRILAVVLGYTVLYLHETPWLPSTWGPSSILFFHTTSSSIPLKPFIQTKLAHDHTKTSEAEDIDPDDFDPDDLDPDDIMMHQCPHLVTLGIVLMELYLATTFEELAEKYNIPLNERTRSIDAELVFSKCKSEIPENSQFHCAVEKCLDPKVWEDENGARLDDQTLKITIYQEVIRPLEDELNHAFSYIPIEDLDRIAQSLDLGSWGQIAQQVNARPVVAAPKSPNESIYSVKQQQQLQQLFLPGQWHVGTPLVVPIFQQSVPNQITLYHEEKSGQLDYKRMRFFDDETVSEEHSAEARTNYSNWRHRFKEVYEQYTIEPPEIPVKIAILDTGIDQTHPDIDACTEQIKGQYNWTNEKFPKHVDDHNGHGTFTAGLLLEYLPDAELYIAKISDGNPCSPENIAKAIDYAIDEWNVDIISMSFGFPTREIKDYDKLEGAIHKAYSNHTLLFAAASNSGANLDRAYPARDENVICIHSTDANGNRSKFSPTALADALNLATVGEAVESSWPLRLCEGNNSLGTKYKSGTSFATPIAVSIAAFLLQYVRTYVPDYAHMLKRQSRMKAVLNKISGKTQESRSRDDYHYVAISRYSDNLFGKEREFINYTLRDLLSQ*

>Pmar_ATCC_18224:PMAA_064980.t1_gene_PMAA_064980_NW_002196662:join(859276..859593859657..860367)

MEHFSNFLQRIKPTNAAPIKIAIIDDGIDATIPDIQRRIAVGKTFSPYPNSTEFMNAYFVPRGLHGTLMARLICKICPKPKLFVARLEERLADDGSSRFTPESAVQAIEWAEACGVDIISMSWTIETRQETGEIKQLKKIINEVGGENGARTGKPPIFLFASASDQGGHIGDASYPGDLRPPCFRIGGSSERGEKLPWVNRDMVDFLLPGQRIPFHDREKDSFTYETGSSLATACAAGLAGLLLYCERVLGESNRIINRGKMEEAFQNLVSPIAPKFPDVSTKLEQGLLEEVKRQTRDKSIQSSSMGLEGLDWDEKQVKDAFAALMNRILPPNQPWARNYST*

>Pmar_ATCC_18224:PMAA_079390.t1_gene_PMAA_079390_NW_002196663:complement(join(703390..703566703623..704561704624..704983))

MRVNNIISFTAVASTAVALVIPDITSFFSSFNQILIPHHPQAPLINQDQHLPSHDKEQNTKPSILSNPLNKIIPNHYIVVLKDDLSQAQFLQHQDWVQKEHVSISSCKSEVNKKPLEFFQIDNFVKGYTGFFTEDLINKILELPEVAFVERDSIFHTVEFDIQKDATWGISRISHRENIDDGKYLFDNDGGRGVTAYVIDTGIKVGHPDFEDRAQWGASIAFPNIKQDYNGHGTHCAGTIGSKTYGIAKNVNLVAVGVMNMLGAGSTSDIIKGIEFVVKSHQEDVRAKKKGFKGSTVNMSIGGGISDALDLAVNAGTKAGLHISVAAGNDNADACQYSPARASGPITIGATNIRDEKASFSNWGSCVDLFAPGEDIESTFIWSDTTVMSGTSMASPHIAGLLSYYLSLQPDITSEFYTQAIEPADLKNRLIKYGTKGVLTGLDSVSPNILAFNGAGGNLTEFWSL

>Pmar_ATCC_18224:PMAA_092590.t1_gene_PMAA_092590_NW_002196664:615923..618058

MSNSIVINGRSVALSHDLDSSSTNYIILRTKGEPLRKSQKAKLKELGVTVYEFVGDESDQVYLCGFKEDSLEQINNLDFVDYVGIYADDFVVPEELQADAHAATVNVDVLLQRDVDEVSEELIEKIAEAAGVNPEDIILEDGGIQIEVEADKLEKIAALDEVRVLHTINEPVLFNNVARRILNFGDDDRDVDDAATAQNTVYTGKGQIVCVADTGLDKGSNTDVHEAFSGRIKHLFAWGRAESDLADDLDGHGTHVCGSVLGKGMHNSQGLVQGTAPAADLIVQSLFSGFNALNQARLGGIPKTNLVPLFDQAYQAGARVHTNSWGSPLPATKIQRPYDGRAESIDLFVWEHQDMTILFAAGNDGQDADLEGKLDGTVNPQSLGAEASAKNCITVGATENYRPDLTSGDENRPYTYGGFWSKRFAMNPLRDDHMANNSEGLAAFSSRGPTTEGRLKPDIVAPGTAILSARSQRQKFSARVDQTGKSGDNKYMYLSGTSMATPLVAGCCAVLREALLANGYRDERPDGVKNPTGSLIKALLINGAVPIKGQYMPEGANEAHNSHSGFGRVDLAGSLPMLNGAYSGYGVGVADEDDKAPFMLEIPVPELKDKKTTAQIGKPQWTFKITLVYADLPGGKLQNDLNLTVILDGYEYHGNQPNCKFSVGSMEAFDRHNNIEQVVLPIVSNETIRIIVKPFRFMSDSVPFAYAWRFI*

>Pmar_ATCC_18224_PMAA_039890.t1

RTYDFTPAEVLGEGQIGNLPGHHKFSKRIKDPEFEKQWHLFNTVQVGHDMNVTGLWLEGITGAGVVTAIVDDGLDMYSNDLKANYFAEGSWDFNEDSPEPRPLLRDDKHGTRCAGEVAAVNNVCGVGLAYDSKVAGIRILSKPIDDADEAAAINYGFQKNDIYSCSWGPRDDGETMEAPGILVRRAMVNGIVQGRGGKGSIYVFAAGNGGFYGDNCNFDGYTNSIYSVTVGAIDRQGQHPSYSEACSAQLVVAYSSGGSIHTTDVCTSIHGGTSAAGPLVSGAIALALSVRPELTWRDVQYLLIETSVPGDEVQMTPIGFSHQFGYGKVDTYSFVQMAKWDLVKPQAWFTTSYFEVTSDMLKAANERIEHVTVTMNINHTRRGDLSTELRGPQGVSHLSVPRNKDGAIAGYEDWTFMSVAHWGETGEGVWSVVVKDDWRMTLWGVSIDPAVQRNRLRYEFLYNAF

>Pnod_SN15:SNOG_02928.t1_gene_SNOG_02928_Pnod_SN15_scaffold_4:complement(join(776019..776519776579..776888776947..777073777180..778201778250..779031))

MALHHLPLLKALGENAHKIRRLPVSVELLDKTTQAPFDAKLDYAERFESQLLELVRKKLSLEHVQSLPSREALALECGLDSLFGHLTNIFNRRLQVDRSQSRSGFSDTVIRLIDVLDDAQYPVDLCGEGGSCLFKFDTIQDARIAHSFARNVNHILQKVHMTTSTHRNLSRPVKNPQAEDIVDTWIQTFGRASSERLGQVLDTIKAEFDACELAQGDTTHELQLLVSSYENQHWEAQGGPNMCLLCPGANAWQLVRCDIGSGLSGNDMHITLCHEMCESIDFDQGLALMLYEDSNSFEPGSTVVDFTHRPLSTSLKGLIENESLFSLPIVPYRFGRKQRRSLAAKLALHLSIFCPWWRHASAPWDENAVHFLKLDSGKIDRESPFIVWKLGTEECYGEEIDARVLAGSFASFAKLLLEIEYGSISNMGIPEYELDNGNLAKSLRKIHKDLLQDADPITQGPYMEAVRACLDFHQRYKFERSRQDVSLRSVVFENPQDTYQRLVRSEITSRVLKGLPDFDILSKRPENPGDNVAEHGDYPCETDCEIIDDDVVPEYRDVPGDLVAVEVSTIPDHSVQPVLKRKISQRWDNLQNGSPSDELEEIIIPRVIEPSLHDSIESVIRGLSDAENGHLFDHEPAPDSQQAQRTDKWFNNSIKFLKALRLGETSAEPLKVAILDSGFNLKNPEFNTDERNRVKCFKSFVDDEPNVDKVDHGTFIATIILRLSVNVDLYIAEITNTNNPDVKTVVEALKYARTTWKVNMISLSFGFDRRNPSDGLFEEIKACLHKDIIVFASASNDGPEGSRTYPAKFPDVICFHSADQRGRKSEFNPTPRGDGDLSFIGEHVRPTWGRTDLTNTSQMVYRDGTSYATPVAVAFAAFMIGFIHTKGWAEWQWTYAPSSPIADDGAKYLLLVL*

>Pnod_SN15:SNOG_06492.t1_gene_SNOG_06492_Pnod_SN15_scaffold_9:complement(join(938186..939118939168..939425))

MKVTNVISFATVASTAVSLVIPDITSFFSSFNQILIPHHQQALLINQDAHLPSDDNQPDTKTGILSNPLNKIIPNHYIVVLKDDLSQAQFLQHQNWVQNEHVSISSCKLNVNKKPLEFFQIDNFVRGYTGFFTDDLINKILELPEVAFVERDSIFHTVEFDIQKDATWGISRVSHRENIDDGKYLFDNDGGKGVTAYVIDTGIKVGHPDFEDRAQWGASIAFPNIKQDYNGHGTHCAGTIGSKTYGIAKNVNLVAVGVMNMLGAGSTSDIIKGLEFVVKLHQDDVRAKKKGFKGSTVNMSIGGGISDALDLAVNAGTKAGLHISVAAGNDNADACQYSPARATGPITIGASNIRDEKASFSNWGSCVDLFAPGEDIESTFIWSDTTVMSGTSMASPHIAGLLSYYLSLQPDVTSEFYTQAIEPADLKNKLIKYGTKGVLTGLDSVSPNILAFNGAGGNLTDFWSL*

>Pnod_SN15:SNOG_11360.t1_gene_SNOG_11360_Pnod_SN15_scaffold_19:190549..191715

MKLQIAILATAIQVSITSALVIPDLQSILGFDQLSDSVKIGKQADTSQGEYNGFAPMLNKGLKGEFIPHKYVIVFKDGIDTEQMKLHKEWVETEYKLMITNEDSAMSSIWKKMVKYGDDAMVDFFNIHDLVSGYSGYFNDALLEKIQHNPMVKFVERDTVFHTREFEVEKEATWGLSRISHRDLTPTVDYLFDNEGGRGVTAYVVDTGIKVEHEEFEGRAQWGEVVPLPKLKLDGHGHGTHCAGIIGSKTYGIAKQVDLVAVGVMNLLGSGTTSDIIKGLEYVVNDHKANSNGKKKNFKGSVVNMSIGGGILEALDLAVNAGTKAGLHIAVAAGNDNQDACEYSPARATGPITVGATNNKDSKASFSNFGRCVDVFAPGEDILSTFTWSKTVSMSGTSMASPHIAGLLSYYLSLQPEVSSQYATPVDPLSLKAKLLKYSTKGVINDLTDGLSPNLLAYNGGGGNLTDFWSI*

>Pnod_SN15:SNOG_13210.t1_gene_SNOG_13210_Pnod_SN15_scaffold_25:join(398549..398614398667..398867398922..399081399132..399234399286..399928)

ANALVIPDVNSFFNFNQFVPLTVAVNAAQQQPQQLWTANKNEAPVLAAGSKSVAIPNRYIVIYKEDVTEAQRNHHKKWLIAEHTEMVATAGIRPSVGVLDFFDVDTLLLGYFGYFTPEMLRKIQKDPRIKFIEQDTVMKVNEFDVEKDAEWGLSRISHRESSPQLEYLYDNEGGKGVTAYVIDTGIKVEHEEFEGRALWGEAVAFPKLKIDGHGHGTHCAGIIGSKTYGVAKNVELVAVGVMNLLGSGTTSDIIKGVEFVVGDHKSNFLAKKKGFKGSTVNMSIGGGESEALDLAVNAATKAGLHVAVAAGNDNADTCTFSPARASGPITVGASDINDNKAEFSNWGSCVDIFAPGVDIVSTYIWSNTASMSGTSMASPHIAGLLSYYLSLYPEPESEYSVAVLDPATLKDKVIKYATKGVIKGLKNDGSPNLLAFNGAGANITDFWSL

>Pnod_SN15:SNOG_15937.t1_gene_SNOG_15937_Pnod_SN15_scaffold_41:245199..246404

MKFLTASLLIGSLLPLISAAPTPQSPSTGSSSDKKFIVVLKDTVNTNQLKKHTQWAARIHARNLQKRQLNGLPVERHISGVEKTFDINRFKAYSGSFDDDTLREIEENDDVAYVEPEQDAYTSEIITQSEATWGISELSNQASNTSTYYYDSSAGEGMYAYIIDSGINFSHEEFEGRAELGYNALKHLNNTDTSGHGTHVSGTVAGKTYGVAKKANLVNVKVFQGRKTSTINIFDGFNWAVQDILEKNRTNIAVINMSLTTKTSKAFNAMVDSAYEKGILSVVAAGNKNVSASLYSPASANTSIAVGAVSRGHVRGSYSNWGPSVNLFAPGTGITSAGIANDTAVRVLTGTSMASPHVAGLICYLRAAGGLTDAKDVVDRMLGLAQQGVLNETTLNESPNLLASNGVELNSYYEEPVGGDYYYY

>Pnod_SN15:SNOG_16555.t1_gene_SNOG_16555_Pnod_SN15_scaffold_50:79046..80473

MVSFRRLATLLVAALLPLGNASPLPETRAASVVEGKYIITLKDDISAAALGGHMNWVRDVHENSLGRRQLSFAGVEKTFGVGNFNAYAGHFDADTLERIRNSPEVADVEQQQLYYLSRLATQKNSTYGLATISHRRPGATEYVYDESAGEGSTVYVLDSGIDIDHEEFQGRAGLGYNAVKHAPARDVDGHGTHVAGTVGSRTYGVAKRAKLVDVKLFHDGASTTEVILDGTEWAIKDIIAKKIQNRAILNMSFGGGKSAVQNKLVKTAYDAGILCVIAAGNEATDAEDRSPASSPDGITVAAIDDEWKLWQYSNYGSGVHILAPGVNVLSTYIGSKTATQEDSGTSMAAPHVAGLAAYLAAAENINTVKELKARILALGTNGTATSVKQGTVNLVAYNGNK

>Pnod_SN15_SNOG_04420.t1

RNYDYSPAAHLGEGPLSSLAEHHVFKRSIHDPIFEEQWHIFNVKDTRQRHQRITGKGVTACVVDDGLDYDSNDLKDNFFAEGSHDYNDHEDLPTPKLSDDRHGTRCAGEIAAGNDACGVGLAYDAKISGVRILSGDITDMDEALAINYEMQKNFIYSCSWGPPDDGKTMQAPGILIEKAMVTAVQQGRGGKGSIYVFAAGNGAASDDNCNFDGYTNSIYSITVGAIDKNNQHPYYSEACSAQLVVTYSSGGGIHTTDVCTSQHGGTSAAGPIGVGVYALLLEARPDLTWRDVQWLTVMTAVPPSDWTKTALDYSHQFGYGKLDAWAIVEKSKWKLVKPQAWFWSSVFEVTADMLKEANERVEHITLTMNVKHQRRGDLLVQLHSPTGISHLSTARRDDEDIRGYQDWTFMSVAHWGESGVGNWTVIIKDDWKLRLWGESIDAAKARKRLRYEFLYDAF

>Pnod_SN15_SNOG_15948.t1

YIVFKGWPSHVMTVDKLHGVTGKGFRISIIDSGDYTHPALGGCFGGCLVEIGYDFTPDPMDNCHGTHVAGTAAGSAPGAKLGAYRMWGCEIELAFARAVEDGASIISYSNGAVIISRIVIPVVVSEGNGGQGFYASTPAAGGSITGTGAVSNNACVLLEGGGQLSSWGPSLDMTPQVVSPGEILSTYPGTSMASPAGIYAMSKAPVAQQGAGIVQAWEAIELDNDTIKNGSEEAVLQIGHRKATVPAGLPVYSGFIPYLGGLLEEGTYRLRVQALRIFGDW

>Pnod09464.t1

PFEPEGWLQIALQGDVAGFEQHVSTPSHPSYGAHYMKRMIQPSSETVASVSAWLDWVTFVGVANKMLDTKFAWYRTLEYSVPDDVAEHINLIQPTTRFTPQCLKTLYSKVAFASYLEQYARYNDLALFEFSVVQFGGNDQEANLDLQYIVGVSAPTEFSTGGRNEPLEFLQGVLKLPQSELPQVISTSYGENEQVPYALSVCNLFAQLGSRGVSVIFSSGDSGPGCQSNDGFQPQYPAACPFVTSVGSETASSGGFSDYWKRPSYQAVKAYFYFNRHGRGFPDVATQGFRVYDQGLQGTSASAPAFAGVIGLLNDARLKAKKPTLGFLNPLLYLNDIVLGGCGWNATAGWDPVTGLGTPFPKL

>Pnod10163.t1

VHEPMAWVRIGLQRNLEHSDDFLADPDSPNFGKFWVANTFAPHPETSEAVLAWLNWIEFVGEVEELLQTEYHYYACDEYGLPRHVREHVDFIMPTIQLTIDCLRAIYNQLGVAEWADYLYLPDLKIYFPEFISIGGNLTESALDIQTAYSIIWPRLYQNGDSNAPLDPAYPPLQCGGAPLSNVISVSYGQIEGLPYQERQCREWMKLGLQGVSVVFASGDSGVACLNSESFSPSFPANCPYVTSVGADGASGGGFSNIFPRPSWQAVSNYLVFNSSGRGIPDVSAIGVATVYLNFGGTSASAPIFASIITLLNEERLEQGKGPIGFLNPTIYFNDVTVGGCGFPASPGWDPVTGHGTPYEKM

>Pnod12886.t1

AVKPRKWLQIGVQGDFDELERHLSDPYHVRYGQHLVNALTQPKDEALDAVHEWLDWINIIEDAEKLLATKYSVYRATSWSLPSHLHKHIDTIQPTTSFTPTCFKTLYNQIGFNNFLKEVPIRPDAKLFLYKFVSIGGQQDEANLDVQTILGMTYPTAFTTGGENEPLEWVTYVLKQKSLPQVISTSYGDDEQVPYAKRVCNSFAQLGARGVSLLFSSGDGGAGCVSNDGFLPSFPAGCPYVTTVGAEVAASGGFSEYFSRPSYQAVKGYLLYNAAGRGYPDISAQGFEFVWNSISGTSASSPLAASVIALVNDALISAGKPTLGFLNPWLYFTDVLSGGCGFAVTKGWDAVTGFGTPFPEL

>Pnod12941.t1

VVEPVGWLKVHLNKDMDKFHDLAATPGHAEYGNHLILAMIAPKQESTDLVMQWLDYVTIVKTIEKLLDAEYSVFRTLKYSLPASLKSHVDMVQPTTFFTPTCLANLYGLLGIAGFLEEYAIKADFTTFLFTCAAVGGCPSEANLDVQYAGSISTSTYYSTAGRNEPLEFLNYLLALPAAQLPNTLSISYGDDEAVPYATNACNLFSQLGARGVSILVSSGDSGVGCSVNGKFTTAFPAACPWVTTVGGEAAGGGGFSEIFGRPSYQAVSKWLYFNASGRAYPDISAQAFVIIAGGVSGTSCSAPATAGIIQLLNSGRIAAGKKGLGFLNPWLYFTDIKTGGCGFSAVSGWDPATGWGTPYGSL

>Pnod14758.t1

TVEPEGWFRVAVSADSSLLERTLSSPSSPNYGQHLLKDLIKPRAESSDAVLSWLEWISFVKRAEEMMGAKFSTYRSLSYSVPKEIRRHIDMIHPITRFTPSCLADLYVSIGVNGFLEQYARYSDFVKFTFTYTLVGGNDQEANLDIQYTAGLVGPSFFSTAGRNEPMEFFTYLLSLENEKLPSVLSTSYGESEQVGYANKVCDMIGQLGTRGVSVIFSSGDTGPGCQTNDGFQAIFPASCPYVTSVGGERASSGGFSDFWKRPAYQAVTNYLLYNAAGRGFPDVAAQGFRVVDKGVGGTSASAPVFASVVALLNNARKAAGMSQMGFLNPWIYLNDIVNGGCSWNATVEWDPVTGHGTPFEKL

>ppla_107691_gene_estExppla_fgenesh3_pm.C_70017_ppla_scaffold_7:complement(join(462588..463012463061..463195463247..463757464080..464532))

MFSFKNLASLLVAALPLSNATPLAGSAADLVPDKYIITLKDGTSANDFNAHMNWVRDVQVARARHRRGLNFRGVEKTYGVGNFNAYAGHFDEHTLEAIRRNADVESVEQQQLYHLHELTTQKNSTHGLATVSHREPGSTEYVYDSRAGEGSTVYVLDSGIQLDHPEFEGRAIHGYNAVKGETDDDVQGHGTHVAGIVGSKTYGVAKKTKLVDVKLFHDAGSTSEIILDGIEWTIKDITAKQIQNRTVVNMSFGGGNSTALNKIIKAAYDAGILCVIASGNMGVDASDWSPASSPDGITVSAIDANWRLWDHSNYGPVVHILAPGVDVLSLAPGNQTQSGSGTSQAAPHVAGLAAYLAVAKNINTVKELKASILSLGTSNKAISVQNNTVNLVAYNGIK

>ppla_111539_gene_estExppla_Genewise1Plus.C_510082_ppla_scaffold_51:join(340995..341447341770..342280342332..342466342515..342939)

MFSFKTLASLLVAALPLGNATPQAGSAANLVPDKYIVTLKDGISANDFNFHMNWVRDVQVARAGHRRGLNFRGVEKTYGVGNFNAYAGHFDEHTLEAIRRNADVESVEQQQVYHLHELTTQKESTHGLATISHREPGSTEYVYDSSAGEGSTVYVLDSGIQVDHPEFEGRAIRGYNAVKDATDEDVQGHGTHVAGIVGSKTYGVAKKTKLVDVKMFHDAGSTNEIILDGIEWTIKDITAKQIQNRTVVNMSFGGGNSTALNKIIKTAYDAGILCVISSGNVGVDASDWSPASSPDGITVGAIDANWRLWDHSNHGPVVHILAPGVDVLSLAPGNETKTGSGTSQAAPHVAGLAAYLAVAKNINTAKELKATILSLGTRDKATAVKDGTVNLVAYNGII

>ppla_112809

LHEPSGFLRIALQNNVDGLVDALSDPTSESYGQHLAASFVAPASESTSAVQAWLDWLGVSSTANDLLSANFSVFRTLAYSIPAELTAHIDFVHPTTTFTPWCLQYIYNGIAVTAYGGAIPQESDLQRFLFTFQSVGGSTSEANLDVQYAIGLATGTFLSVGGEDDALDTANYLLSLESPPPVVATSYGDNENISLAYNLCNAYAQLGARGVSVIFASGDGGVSKNFVPTFPSGCPYVTSVGAEQASSGGFSNYWPQPSYQAVSAFLLYNASGRAFPDVAAYGYDIYNSRVSGTSAAVPTVASIVALLNDRLLTAGRATLGWLNPWLYFASVTAGACGFYATSGWDPITGLGTPFDKL

>ppla_113327

VHEPSGFLRLGLNSNTDGLIATLSTPWSANYGKHLVEAFTAPTKGTVDAVNTWVDWLSIVSKANEMLDANFSVYRTLSYSIPTDLKGHLDLVHPTTTFDPQCLEELYDYLVVTGYNNEYPSTTDLVDFLYTVVEIGGYDPEADIDTQYTVGLAIGTFTSVGESVFGLDTANYWLGQSTTPSVISTSYGSNEEISVYNSLCNAYASLGLRGTSVLFSSGDGAVTQSFVPTFPSGCPYVTSVGAETASSGGFSNVFAIPPFQSVAGYLLYNASGRGYPDVSAQGFIFNYQGVEGTSCSSPTFASVIALVNDRLVAAGRSKLGWLNPFLYLTDITSGGCGFSATTGWDPVTGLGTPFDEL

>ppla_117103

RHYDYASASSLGVEQAGALQDHWLVRKRITDPEFGRQWHIVNDASPHNMMNVTGVWDMGITGKGVISALVDDGLDYTSDDLAANFYAYGSYDFNDHLDLPTPTLFDDHHGTRCAGQIAAVNDVCGVGIAYDSKVAGLRILSGPISDVDESAALNYDYQNTSIFSCSWGPPDDGRSMEGPGYLIKKAMVNGVQNGRQGKGSIFVFASGNGGRSADQCNFDGYTNSIFSVTVAAVDYRGLHPDYSEACAANMVVAYSSGSGITTTDRCAHSHGGTSAAAPNAAGIFALALQTRPDLSWRDVQHLCVKTAQMDPDWETTAAGFSYKYGYGVINGYEFIKAAQWQLVKPQAFIDLNSIEVTQSMLDENNEKLEHITVKVWITHTRRGDVEVELVSPNGRSILAATRYGDSAKTGFPGWTFMTVKHWDENPVGKWSLRVNDGWTMTVWGSVKDATKPRARLYDAF

>ppla_117588

KVKPRGWLRIALQSNFAELEQHLSDPFHARYGAYLVEALVAPHDDSVQLVEEWLDWITVVSLAETMLKTEYHIWRTTSYSLPERLVDHVDLVQPTTMFTPTCLKQLYNKVAATGYLDEYANYEDLQMFYFSVVSVGGNNQEADLDVEYAFSLSYPTFYTTGGSNEPSEWLSYMLSIEDPPQTISTSYGDDEQVPYAKRVCEDFAQLGARGVSIIFASGDGGVGCYSNDGFIPGFPASCPYVTAVGAEIASGGGFSNYFPRPSYQAVEAYLLYNPNGRAIPDVAAQGFQIFWDGVAGTSASTPSFSGIVSLLNDARIANSLSPLGFLNPLVYFNDITVGGCFNASVGWDPVTGWGSPFGVL

>ppla_120448

SSDTFTVETLGGDPQEANLDTQYTVGLATDVFISVGLDGLDIINYLLAQDAPPQVLTTSYGSSESVPMAENLCNAYAQLGARGVSILFASGDGGVSQDSFVPTFPSGCPYLTSVGAETASSGGFSNYWGVPSYQAVSGYLRYNASGRGYPDVSAQGFNIVLDQVSGTSCASPTFASVIALLNDELIAAGKSPLGFLNPWLYLNDVTSGGCGFSATTGW

>ppla_121576

VLEPRGFLRLALQNNMDGLIEALSTPSSAKYGQHLVEEYVSPTSMTIAAVNAWLDWLAIVSQANSMLDADFSVFRTLSYSIPSDLVSHLDLIHPTVTFDADCIETLYNYIAVTGYNNYYANEADLDYFLFTVETLGGNDQEANLDTQYTTGLATYTFLSVGQNVFGLDVANYFLDMETPPSVITTSYGSNEELSVAYALCNAYAQLGARGVSVLYSSGDGGVSQNFVPSFPSGCPYVTSVGAEVASSGGFSNYWATPSFQAVSNYLLYNASGRGFPDVSAQGIMIAQYQVDGTSCSSPIFASVVGMLNNQLISAGKSTLGWLNPFLYLTDITSGGCSFSATTGWDPVTGLGTPYTLL

>ppla_128724

VLGPAGFLRIALQSNPAGLEDALSTPSSPNYGQFLAASFVVPSPETQATVNAWLDWLSFVSKANEIFEAEFNVYRTMSYSIPQELQGHLTVVYPTTTFTPSCLQSLYNKIGVAGFDEQYADDQDLKTFLFTVQTLGGNSQEASLDIQYTVGVATNIFISAGEQLEGLDMANFLLNEDSLPQVFTTSYGPNENIPLSINVCNAYAQLGARGVSVLFASGDGGVSQYDFIVPFPCGCPYHTSVGSETASSGGFSNYFARPSYQAVSAYLLYNASGRGFPDISTQGFDVVIDTVSGTSASSPTFASVIALINDELIAAGKSPLGFLNPWLYLNDITEGGCGFSATTGWDPVTGWGTPYAKL

>ppla_39235

LRVALQNNIAGLIDALSSPSSPNYGKWLVEAHVAPKQHSVAAVNSWLDWLRIVAKANDMLAANFSVFRALSYSVPSDLAEHIDLIHPTTAYSSACLQHLYNGIAVTEFEKQYAQAADLHSFLYTVISIGGNPQEADLDLQYTAGLATGTVTIAGGDLAGLDTGLSLLGLESPPQVVSTSWDGDEDFPHAVYLCNVYAQLGARGVSMIFASGDEGASRSEGFSPTFPATCPHVTTVGAEVVSGGGFSNYFLRPDYQAVSAYLLYNASGRAYPDVAAYGCTYIMGGGTGTSCSAPIFASVIALLNDRLLAAGKPTSGFLNPW

>ppla_48962

VLGPAGFLRIALQSNPTGLEDALSTPSSSNYGQFLAASFVTPSSETQAAVNAWLDWLSFVSKANGIFEAEFNVYRTMSYSIPQELQGHLTVVYPTTTFTPACLQSLYNKLGVTGYGDNWANKADLKTFLFSLETLGGDPQEADLDTQYTVGIATGTFISVGLDGLDTINYLLNQDAPPQVLTTSYGDYEPIPMAENLCNAYAQLGARGVSLLFASGDGGVSQSFVPEFPSGCPYMTSVGGETASGGGFSNYWSRPSYQVVSDYLLYNASGRGFPDVATQAFIIAYEGVSGTSCASPTFASIIALLNDELIAAGDSPLGFLNPWLYFTDITSGGCGFNATVGWDPVTGLGTPYAKL

>ppla_48986

VLAPAGYLRIALQSNPAGLEEALSTPTSPNYGQFLAASYVAPSPETQTVINAWLDWLGFVGKANELFDAAYSVYRTLSYSIPQELEGHLSVVYPTTTFTLSCLQALYSRIAVAGFDDQWANEADLMTFLFTLQDLGGNNQEANLDIQYTVGIATDVFVSAGLDGLDMADLLLNEDSPPQAFTTSYGPNEDVPLSYNLCNAYAQLGARGVSVLFASGDGGVSQSFVVPFPDGCPFMTNVGSEIGSSGGFSNYYARPSYQAVTAYLLYNASGRAFPDVATQGFTVVIDQVSGTSCSSPTFASVVALLNNELISAGKSPLGFLNPWLYFNDITEGGCGFYAIAGWDPVTGWGTPYAKL

>ppla_50115

VHEPSGYLRVGLENNVDGLISALSDPSSANYGQHLANSYLTPTAESASAVNAWLDWLAVVSKANELLGADYSVFRTLSYSIPANLTGHLDLVHPTISFTPDCLLYLYNGYAVTEYIEQWATYADLKTYLWIYQSIGGNPQEAELDVQYAIGLTTDTFISVGLDTALTLLNETAPPQVMSTSYGDDEDVSFAYKLCNAYAALGARGVSVVYASGDGGVSHFDFLPVFPAACPYVTSVGAQTASGGGFSNYWTRPLYQAVAGYLLYNPSGRGYPDVAAYGFDVVYAGVSGTSCSSPTFGSVIALLNARLLAAGRPTLGFLNPFLYLTDITTGACGFYASEGWDPITGLGTPFANL

>ppla_50482

LHEPSGFLRIALQNNVDGLVDALSDPTSESYGQHLVASFVAPASESTSAVQAWLDWLGVSSTANDLLSANFSVFRTLAYSIPAELTAHIDFVHPTTTFTPWCLQYIYNGIAVTAYGGAIPQESDLQRFLFTFQSVGGSTSEANLDVQYAIGLATGTFLSVGGEDDALDTANYLLSLESPPPVVATSYGDNENISLAYNLCNAYAQLGARGVSVIFASGDGGVSKNFVPTFPSGCPYVTSVGAEQASSGGFSNYWPQPSYQAVSAFLLYNASGRAFPDVAAYGYDIYNSRVSGTSAAVPTVASIVALLNDRLLTAGRATLGWLNPWLYFASVTAGACGFYATSGWDPITGLGTPFDKL

>ppla_50496

VHEPSGYFKLALQNDPNGLVDALSAPDGPKYGQYLVVSFVSPRPESTAAVNAWLDWLSIVNKINDLIDAEYSVFRTLSYSIPADLIDHLDLIHPTISFDPACIQYLYNRLAVTGYDGEWASKADLQQFLFTLQTLGGNPQEAAIDVQWTVGLATDTFISVGGDLDTANFLLGEDSPPQVVSTSYGDDESVSLAYALCNAYAQLGARGVTVINSSGDGGVSHYFVPTFPSGCPFVTSVGGEWGSGGGFSNYWSRPAYQAVSHYLLYNASGRGYPDVSAYSFDVIVDGEVGTSCSAPTWASVVALLNDRLVSAGKPALGFLNPFLYLTDIVNGWCGFEATVGWDPVTGLGTPFTNL

>ppla_53113

VLAPAGFLRVALQSNPAGLEDALSTPSSANYGNHLAAAFVAPTQEATAAVTSWLDWLSLVSQANELFGAQFNVYRTMSYAVPQTLAAHLTVVYPTTTYTPACLQSLYNQLGVSGFIDQFANQADLKTFLFSVQTLGGNSQEANLDTQYTVGLATGTFISVGEKDLGLDIMNFLLNENDPPAVLTTSYGDNEDIPMADNLCNAVAQLGARGVSVLFASGDGGVSQAFVPTFPSGCPYLTSVGAETASAGGFSNYFGTPSYQAVSTYLLFNASGRGYPDVSTQGFEIVVDGVDGTSCASPTFASVIALLNDQLVAAGKSTLGFLNPWLYLTDITSGGCGFPAVTGWDAVTGLGTPFAKL

>ppla_53544

LHEPVGFIQLALQNNVSGLVDELSDPSSANYGQYLVVSFVSPRPESTAAVNAWLDWLSIVNKINDLIDAEYSVFRTLSYSIPADLIDHLDLIHPTISFDPACIQYLYNRLAVTGYDGEWASKADLQQFLFTLQTLGGNPQEAAIDVQWTVGLATHTFISVGGDLDTANFLLGEDSPPQVVSTSYGDDENVSLAYALCNAYAQLGARGVTVINSSGDGGVSHYFVPTFPSGCPFVTSVGGEWGSGGGFSNYWSRPAYQAVSHYLLYNASGRGYPDVSAYSFDVIVDGEVGTSCSAPTWASVVALLNDRLVSAGKPALGFLNPFLYLTDIVNGWCGFEATVGWDPVTGLGTPFTNL

>ppla_53719

VHEPSGFLRLGLNSNTDGLITTLSTPSSANYGQHLVGAFTAPTKETVDAVNAWIDWLAIVSKANEMLDADFSVFRTLSYSIPTDLKGHLDLVHPTTTFDPQCLEQLYNYLVVTGYNNEYPSTTDLVDFLYTVVEIGGYDPEADIDIQYTVGLAIGTFTSVGESVFGLDTANYWLGQSTTPSVISTSYGSNEEISVYNSLCNAYASLGLRGTSVLFSSGDGAVTQSFVPTFPSGCPYVTSVGAETASSGGFSNVFAIPPFQSVAGYLLYNASGRGYPDVSAQGFIFNYQGVEGTSCSSPTFASVIALVNDRLVAAGRSKLGWLNPFLYLTDITSGGCGFSATTGWDPVTGLGTP

>ppla_56190

VLGPSGFLRIALQSDPAALEEALSTPSSSNYKQYLVSAFVAPSPEAVSAVNAWLDWVEVVSKANEIFNADYSVFRTLSYSIPEELTDHVAIVHPTTTFTPACLQSLYNTLGVSGFSDQYANQADLATFLFTVETLGGDPQEANLDTQYTVGLATDVFISVGLDGLDIINYLLAQDAPPQVLTTSYGSSESVPMAENLCNAYAQLGARGVSILFASGDGGVSQDSFVPTFPSGCPYLTSVGAETASSGGFSNYWGVPSYQAVSGYLRYNASGRGYPDVSAQGFNIVLDQVSGTSCASPTFASVIALLNDELIAAGKSPLGFLNPWLYLNDVTSGGCGFSATTGWDPVTGLGTPYTSL

>ppla_58105

ISFASTGPKLPITVKQLDPACLEALYNYIVVTGYDDQYPSTSDLENFLYTVVELGGYDPEADLDIQYTVGLALGTFFSVGEDVFGLDTANYWLGQSTAPSVITTSYGSDESISVFNSLCNAYASLGARGTSVLFASGDGGVSQSGFVPTFPSGCPYVTSVGAETASSGGFSNVFGTPSFQDVSSYLLYNASGRGFPDVSAQGFIIGYEGVSGTSCASPTFASVIGLVNDRLVAAGKSPLGWLNPFLYLTDITSGGCGFSATTGWDPVTGLGTPFSAL

>ppla_88376

VHEPTGYLRVALQNNIAGLIDALSSPSSPNYGKWLVEAYVAPKQDSVAAVNSWLDWLGIVSKANNMLAANFSVFRTLSYSVPSDLVDHIDLIHPTIIFTPACLQHLYNGIAVTEYEEQYAQGADLHSFLYTVISIGGNPQEADLDLQYTAGLATGTVTIDGLTGLNTGFSLLGLESPPQVVSTSWGGDENFSYATNLCNVYAQLGARGVSMIFSSGDGGVSQFEFNPTFPSTCPHITTVGAEVASGGGFSNYFPRPDYQVVSAYLLYNASGRAYPDVSAYGCSVVIGGVSGTSCSAPIFASTIAILNDRLLAAGKPTLGFLNPWLYFTDIVSGSCGFFATKGWDPVTGFGTPFASL

>ppla_89125

VHEPSGYLRVALQNNIDGLIEALSTPSSSNYGKWLVEAYTAPESETVDAVNSWLDWLGIVSKANEILATDFSVFRTLSYSLPSNLIGHVALVHPTITFTPACLQELYNGLAVAEYEYEYAEESDLHTFLFTVLSINGNPQEASLDLQYTVGIATNTFITVGYNLDTANTLIALDSPPQVVSTSYGEDEQVSFATTLCNAYAQLGARGVSLIFSSGDGGVSHFFNPTFPSVCPHITTVGAEVASGGGFSNIFPRPSYQAVSAYLLYNASGRGYPDVSAQGFEVVNAGVSGTSCSAPTFASVVALLNDRLLASGKPTLGFLNPFLYFNDITSGACGFFTAPGWDPVSGLGTPFAKL

>ppla_89318

VLAPARFLRVALQSNPAGLEDALSTPSSANYGNHLAAAFVAPTKEATAAVTSWLDWLSLVSQANELFGAQFNVYRTMSYAVPQTLAAHLTVVYPTTTKTPACLQSLYNQLGVSGFIDQFANQADLKTFLFSLQTLGGNSQEANLDTQYTVGLATGTFISVGEKDLGLDIMNFLLNENDPPAVLTTSYGDNEDIPMADNLCNAVAQLGARGVSVLFASGDGGVSQAFVPTFPSGCPYLTSVGAETASAGGFSNYFGTPSYQAVSTYLLFNASGRGYPDVSTQGFEIVVDGVDGTSCASPTFASVIALLNDQLVAAGKSTLGFLNPWLYLTDITSGGCGFPAVTGWDAVTGLGTPFAKL

>ppla_89706

MHGPTGYLRVALQNNIAGLIDALSSPSSPNYGKWLVEAYVAPKQDSVAAVSSWLDWLEIVAKANDMLAANFSVFRTLSYSVPSDLAEHIELIHLTIAFTPVCLQHLYNGIAVTEFEKQYAQAADLHSFLYTVISIGGNPQEADLDLQYTAGLATGTVTIAGLAGLDTGLSLLGLESPPQVVSTSWSGDEDIPYAVHLCNVYAQLGARGVSMIFASGDGGASRTEFSPTFPATCPHVTTVGAEVASGGGFSNYFPRPDYQAVSAYLLYNMSGRAYPDVTAYGCTFIMGGGSGTSCSAPIFASVIALLNDRLLAAGEPTLGFLNPWLYFTDIVSGSCGFVATEGWDPVTGFGTPFANL

>ppla_98402

VHEPSGFLRLALNSDTDGLIAALSTPSSANYGQHLVDAFNAPTKESVDAVNAWLDWLAIVSKANVMFDADFSIFRTLSYSIPTDLVDHLSLVHPTTTFDPACLEALYNYIVVTGYDDQYPSTSDLENFLYTVVELGGYDPEADLDIQYTVGLALGTFFSVGEDVFGLDTANYWLGQSTAPSVITTSYGSDESISVFNSLCNAYASLGARGTSVLFASGDGGVSQSGFVPTFPSGCPYVTSVGAETASSGGFSNVFGTPSFQDVSSYLLYNASGRGFPDVSAQGFIIGYEGVSGTSCASPTFASVIGLVNDRLVAAGKSPLGWLNPFLYLTDITSGGCGFTATTGWDPVTGLGTPFSAL

>ppla_99394

FLYTVVELGGYDPEADLDIQYTVGLALGTFFSVGEDVFGLDTANYWLGQSTAPSVITTSYGSDESISVFNSLCNAYASLGARGTSVLFASGDGGVSQSGFVPTFPSGCPYVTSVGAETASSGGFSNVFGTPSFQDVSSYLLYNASGRGFPDVSAQGFIIGYEGVSGTSCASPTFASVIGLVNDRLVAAGKSPLGWLNPFLYLTDITSGGCGFSATTGWDPVTGLGTPFSAL

>psti_14973

RDYNYSQADFTSEHQLQGLDNHYVFSKRIRDPGFIEQWHLINTAYPGHDVNVTGLWYEGITGTGIVSAIVDDGLDAESEDLRANFNAKGSWDFNDNTNIPLPRLYDDHHGTRCAGEIAAVNDVCGVGVAYDSTVAGIRILSGPITAAEEAAALIYGLDVNDIYSCSWGPTDDGRTLAEPETVVKKAMIKGVQEGRKDKGSIYVFASGNGGRSYDSCNYDGYTNSIFSITVGAIDYKGIHPDYAEACSAVMVVTYSSGSGIHTTDICTASHGGTSAAAPLAAGIYALVLQANPNLTWRDVQYVSVLSSVPDGNYQTTALNYSHKYGYGKIDAYQMVHFAKWKNVKPQAFFYSKKITVTEEDLKIMNERVEHVTVKLNIMATFRGRVGVRLISPTGTSDLATFRPRDNSGVGFKDWTFMSVAHWGESGLGDWTIEVNWQLRFFGESIDADKARRFEFVYDEF

>psti_28842_gene_fgenesh1_pg.C_chr_1.2000015_psti_chr_1.2:46154..47350

MFSFKTLASLLVAALPLGNATPQAGSAANLVPDRYIVTLKDGISANDFNFHMNWVRDVQVARAGHRRGLNFRGVEKTYGVGNFNAYAGHFDEHTLEAIRRNADVESVEQQQVYHLHELTTQKESTHGLATISHREPGSTEYVYDSSAGEGSTVYVLDSGIQVDHPEFEGRAIRGYNAVKDATDEDVQGHGTHVAGIVGSKTYGVAKKTKLVDVKMFHDAGSTNEIILDGIEWTIKDITAKQIQNRTVVNMSFGGGNSTALNKIIKTAYDAGILCVISSGNMGVDASDWSPASSPDGITVGAIDANWRLWDHSNHGPVVHILAPGVDVLSLAPGNETKTGSGTSQAAPHVAGLAAYLAVAKNINTAKELKASILSLGTRDKATAVKDGTVNLVAYNGII

>psti_4493_gene_gwh1.4.1.442.1_psti_chr_4.1:complement(934969..936315)

MVKITYLLLCVGIALALPVAKRDNAPVPIPGKYIITLRPGVAPSLETHLSWVRDVHTRSLSRRDESGIEKVYSALDFHGYAGSFDEETIAQIRANPDVSSVEQDQTFHLTYHLPSQPRPRQTGLTTQKDAPWGLGSISHRAPNSTDYIYDSRGDAGDGYTAYVVDTGIRTTHNEFEGGRAIFGYNAYPDADSDEDNIGHGTHVSGTIAGKTYGVAKKARVVAVKVFDWGSVSYFFLSLQGCPSTTSIVLDGYLWAVNNITTPAKSVINLSLGGPQSDAVDSAIAAAYSAGILTVVAAGNDGRSSDNGWGSPASAPEALAVGAVDVENVRPSFSNWGPGVDIWAPGVMVRSAWNWDDGDYLEVEGTSMASPHVAGLVLYLRSLEGGGQGGLVGAVVDKVRELGTKGVVKEAGRGSVNLLAYNGNGA*

>psti_78783_gene_estExpsti_genewise1_worm.C_chr_6.10406_psti_chr_6.1:complement(1293989..1295623)

MASYLTILTALAAVFAPVFAAPAAIPHPKIKTPTAAKEIVADSYIVVYNTDITTEVTASHVDFVNSIVAKRDNAVSVGATYKIKDFAGYHISADEATIVEIANKPEVAYIEKDQKVYASTLTTQSGATWGLGRISHRAKSTTSYIYDSTAGSGTTVYVVDTGVYAAHSQFGGRASMGANFVSGSANTDENGHGTHCSGTIAGSTYGVAKAAKIVGVKVLDASGSGTTAGVISGIQWVATNHVSKSVLSMSLGGGFSTSLNSAVTSTVASGVTVVVAAGNDNANAANTSPASTPNAITVGAIDTNDARASFSNYGSVLDVFAPGVNVLSSWIGSTSATNTISGTSMATPHVAGLAAYLIALEGLSTPAAVEARIKALATSGSITNAGSGSPNLIAYNGDGA*

>Ptri_00288.t1

VVEPEGWFRIAVSKNRDLFERTLSSPSSPNYGKHLLKDLIKPRAESVATVINWLEWINFVKRAEAMMGTTFKTYRSLGYSVPGHVRPSIDMIQPTTRFTPTCLADLYTKLGVTGYLEQYARFGDLEKFLFDVEAIIGLISPTFYTSPGRNEPLDLFTYLMELDDGELPQVLSTSYGENEQVPYAKKVCDMIGQLGARGVSVIFSSGDSGTGCQTNDGFSPIFPAACPYVTSVGGERASSGGFSDLWPRPAYQAVGDYLLYNPNGRGFPDVAAQGFQVVDSGVAGTSASAPVFAAVVAMLNNARMGAGMPTLGFLNPWIYMNDVVDGGCSWNATKGWDPVTGYGTPFEQL

>Ptri_00647.t1

FIVFKGWPSHVMTVDKLHGITGKGFRISVVDSGDWTHEALGGCFGGCLIEAGYDFIPDPMDNCHGTHVAGIIVGSAPGAKLGMYRMWGCEIEFAFARAVEEGADIISYSNGAILVSRIVIPVVVSEGNGGKGFYASTPATAVSATGAGAVTNNACVLVEGGGLLSTWGPSLNMTPQLVAPGEILGTWPGTSMSSPAGVYALAKAPVPQQGAGIVQAWNAIELDNDTISNGSTDEVLKMGHRKATIPAGLAVYSGFIPYMGGLLDAGVYKMRVRALRIFGDW

>Ptri_01300.t1

IVEPSGWLKIHLNKDMDKFHEHAATPGHNLYGQHMILAMVAPAEESAALVMKWLDYVTVVKEIEQLLDAEYNVFRTLSYSLPKFLKSHVDMVQPTTFFTPTCLATLYGLLGIAGFLEEYAIKSDYTSFLFTCTTIGGCPSEANLDVQYAGSISTSTYYSIAGRNEPIEFLQYVLALPDPSLPNTLSISYGDEEAVPYATNACNLFSQLGARGVSILVSAGDSGVGCTVGGKFTTAFPAACPWVTTVGGEVAGGGGFSEIFGRPSYQTVSKWFYFNASGRAYPDISAQAFVIVVGGVSGTSCSAPTTAGIIQLLNSGRIAAGKKGLGFLNPWLYFTDIKNGGPPHNDWYPVSQ

>Ptri_03887.t1

PFEPEGWLQIALQGDTEAFEQHVSTPSNAKYGQHYMKRMLMPSEQTVTSVSSWLDWVTFVGVANELLGTKFSWFRTLEYTVPDDVAQHINLVQPTTRFTPQCLKKLYSKVAFASYLEQYARYNDLELFEFTVVQFGGNDQEANLDMQYMFGLAQPTEYSTGGRNEPLEFLQGVLKLPQEELPQVISTSYGENEQVPYALTVCNMFAQLGSRGVTVLFSSGDSGTGCLSNDGFQPQYPATCPFVTSVGSETASSGGFSDYWKRPAYQAIKAYFYFNRHGRGFPDVAAQGYAVYDKGYQGTSCSSPAFGGIVALLNDARLKSKKPSLGFLNPLLYLNDVVLGGCSWNATVGWDPVTGLGTPFPKL

>Ptri_07068.t1

AVKPREWLQIGLQGRFEELDRHLSDPDHVRYGQHLVDELVAPTSETYNLVHEWLDWVIVIEMVESLLDTEYHTYRTTKWSLPRHLHSHIDTVQPTTSFTPECFQTLYNSVAFNNFLGEIPIRPDTKKFLFKTISINGLQDEANLDVQAIAGISWKTSYSTGGSNEPLVWVNWLLTQRSIPNIISTSYGDSEQVPYAERVCRQFAQVGARGTTLFFSSGDSGIGCYTNDGFNPNFPASCPYVTTVGAEEAASGGFSNYFSRPSYQVVPKYILYNKTGRGYPDLAAQGFAYFWNGISGTSASSPLTAGIFALVNDALISKGKPTLGFLNPWLYLTDITKGGCGFPVTKGWDPVTGFGTPFPEL

>Ptri_07828.t1

YLVFKGWPVHVIGVDKLRNLTGKGIFIGVIDGGDFMHPALGGGFGGFKISAGEDLVPGPMDCHGTHVSGIIGGVAPEATLGVWKIFGCDMIMGFNIAYEAGVDIISASFGSVVVQRISVVVVVAAGNGTIGFDAQAPANSAGALAVASIDNTACTLVQGASDFSSWGPTLSIKPEVAAPGGILSTWPGTSMAAPAGTIALADPAPVMQQGGGGLNAYKATTVDNDTIKNFKQAQTYTFDNLTVEAGLPLYNGYVPYMGGTLPAGTYKLILRALKIFGDY

>Ptri_08228.t1

HIVLQGWSNHVMTIDKLHNITGKDVKIAIIDSGDYTLDALGGCLGNCLVAGGYDFVPDPMDNCHGTHIAGIISGVAPVRYLPIRCDVASGNVASIASFVNMGCVLMRGASDFSSWGPTLDFKPQFGAPGEILSTYPGTSMSCPAGIYALSKPAPVAQQGAGLLQAYDATALSNDTISNGHKAITYQLDVVNATVPAGLPVYGGYIPYQGGQLPAGTYKMRVRAMHLFTY

>Ptri_Pt1CBFP:PTRG_01819.t1_gene_PTRG_01819_Ptri_Pt1CBFP_supercont1.1:join(5267917..52682015268412..5269644)

MLSVKILLALLPSALAQFTIGEVVEDNYIITLKPKVNETEIEQHIEWVDDIHTASLLRRGEDGVDKVWNSTFKGYCGEFDKSTIRRINQSEDVLAVEPVKKVTLATVQRNAEWGLASISHRTTGSTDYLYDASAGNGMYAYLVDTGINYGHRDFQGRASPGYNAYPGVPFVDVNGHGTHCAGTIAGKVYGVAKRANLIAVKVFHSGSSTTAIVLDGYNWAVNNITNTPGRNQQSVISMSLGGGKSDAFNLAVEMAYRQNIHTVVAAGNSNVNANDTSPASAQNATTVGAIDKNNNRASFSNFGPFVDIFAPGVSIKSTWIGSDSATETLSGTSMACPHVAGLSLYLRAKEGLKTVKSVQDRIKQLATKNVIANAGAGSPNLLAYNGGVPTRTTFRKGDWV

>Ptri_Pt1CBFP:PTRG_02358.t1_gene_PTRG_02358_Ptri_Pt1CBFP_supercont1.2:join(201116..202117202168..202362)

MHYLKLLLALLPAIIAAPTVTDDDDIIEGAYIVTLKQKLDEKKVEQHIDWVDGIHNGSVFRRTEDGVKLVWNETTYKGYSGDFDKQTIKEIKKSKDVFAVEPVRKINLYETITQQRSTWGLGSISHRTPHFNNYIYDSSAGAGTYAYVVDTGINIGHDEFQGRAALGYNAYPGAEFVDANGHGTHCAGTIAGKEYGVAKRANLIAVKVFHTGSSRTDIVLDGYNWAVTNITNTPGRKEQAVISMSLGGSRSDAFNAAVQAAYSAGVHTVVAAGNDNADAAKYSPASAPNATTIGAIDVDNKRASFSNYGELVDLFAPGVNVKSAWYTSNSATNTISGTSMACPHVAGLSLYLRAKEGLTTPESVARRLKELATSGVVQDAGSGSPNLLAYNGAPSS

>Ptri_Pt1CBFP:PTRG_02359.t1_gene_PTRG_02359_Ptri_Pt1CBFP_supercont1.2:join(205222..205371205420..205568205621..205670205717..206006206056..206487)

MHYLKLLLALLPAIIAAPTVTNDDDIIEGAYIVTLKQKLDEKKVEQHIDWVDGIHNGSVFRRADDGVKLVWNETTYKGYSGDFDKQTIKEIKKSKDVLAVEPVRKINLYETITQQKSTWGLGSISHRTPHFNDYIYDSTAGAGTYAYVVDTGINIAHDEFQGRAALGYNAYPGADFVDANGHGTHCAGTIAGKEYGVAKRANLIAVKVFHLGSSRTDIVLDGYNWAVTNITNTPGRKEQAVISMSLGGGRSDAFNAAVQAAYNAGVHTVVAAGNDGKDAYNYSPASAPNATTVGAIDINNNRASFSNYGELVDLFAPGVNIKSAWNTTNSATKTISGTSMACPHVAGLSLYLRAKEGLTTPESVARRLKELATTGVVQNAGSGSPNLLAYNGAPPS

>Ptri_Pt1CBFP:PTRG_08105.t1_gene_PTRG_08105_Ptri_Pt1CBFP_supercont1.8:complement(1731938..1733359)

MVNFAVIATTLVGTLLLPGAVLGAPVDPVDHSITDRYIVTLKPGVTMERRDAHLDRVASLHKRSLGRRDLPGTESTVDIEEFHAYYGKFDDATVEEIKRDPDVAAVEPDQIWTTSAEVTQNGSTWGLAAVSHHNAGFDSYIYDDTVTGADMYAYVIDSGININHVDFGGRAVRGYNAWGGVHTDVSGHGTHVAGTIGSKTYGVFKDVNLIDVKVLSGSSTTTAVVLEAYTWSVNDILAKSRTAKSVINMSLSARNSDAYSGAIAAAYNAGVLSVVAAGNDNLPSSTRSPGSAPEAITVGAIASDWTEAEYSNYGPSVDVLAPGSHVLSTYIGTNTATFSMSGTSSATPHVAGLALYLMARDGVSSPAAVADRIKELATPNAATLRNADTPNLVAFNGSSLV*

>Ptri_Pt1CBFP:PTRG_08685.t1_gene_PTRG_08685_Ptri_Pt1CBFP_supercont1.9:complement(join(1747859..17484951748549..17486511748711..17488701748932..1749258))

MLFKSIIAGASALVSLSAAAPVDSRASSNVIEGSYIVKLKDNVAADEHISWVAYVEADQIWTLEESAVKRDLTTQESAPWGLAAISHREANAAGYIHDTAAGAGTFGYVVDSGVRTTHQEFEGRASTGWTGYPGDESDTLGHGTHVAGTVGGKTFGVAKKATIISVKVFQGKEGSTSIVLAGFDWAVNDIISKGRRDKSAINLSLAGPYSRAWNEAVRTAFTKGVLSIVAAGNAQADAGNYSPASAPNAVTVGAVNQNWGIVTDWPVGQGSNYGTVLDIFAPGDNILSAGFNSDSESRFDSGTSMAAPHVTGLALYAISVDGVRGAKAVTDHLIKNSGKGVVTGPLRGSPNRFANNGNPSQ

>Ptri_Pt1CBFP_PTRG_07955.t1

RNYDYSPAAHLGEGPFGSLEDHHVFKRSIKDPIFEEQWHLFNVKTPGNDINVTGVWTQGITGKNVTACVVDDGLDYTSNDLKDNFFAQGSHDYNDHEDLPTPKLSDDRHGTRCAGEIAAGNDACGVGLAYDAKISGVRILSGDITDLDEALAINHEMQANDIYSCSWGPPDDGKTMQAPGILIEKAMVTAVQQGRGGKGSIYVFAAGNGAASDDNCNFDGYTNSIYSITVGAIDMNNAHPYYSEACSAQLVVTYSSGGGIHTTDVCTAQHGGTSAAGPIGVGVFALALSARPELTWRDVQWITVMTAIPPSDWTKTSLGFSHQFGYGKLDAWAVVEKAKWKLVKPQAWFYSSSFEVTEEMLKKVNERVEHITLTMNIEHERRGDLSVELRSPSGVSHLSTPRRSDEAPYGYVDWTFMSVAHWGENAVGNWTVIVKDDWKLRLWGECIDASKARKRMKYEFLYDAF

>RO3T_00044_gene_RO3G_00045_rory_supercontig_3.1:join(104076..104455104508..104632104748..105385)

MLFKSIIAAVSAFAGLSAAAPANIRASNVIEGSYIVKLKDNVATDKHLSWVSGIHARRNVNNDVAGVEREYNSPAFHGYAGQFDQQTIVEIESSPEVAYVEADQIWTLEESMEKRDLTTQESAPWGLAAISHRQPNATGYIYDTAAGQGTFGYVMDTGIRATHREFEGRASTGWTGWAGDDRDISGHGTHVAGTVGGVTFGVAKKATIIAVKVFHGTQTSTSIIMGGFDWAVNDIVSKGRQDKSVINQSLGGPYSRAWNDAIEAAFTRGVLSVIAAGNAQRDASEVSPASAPNAVTVGAVDKNWRIVTNWPNGQGSNYGPVLDIFAPGDGIESAEPNSDSQTGLRSGTSMASPHVAGLALYAMSVDGVRGAQAVTDHLIKNSGKGVVTGPLRGSPNRFANNGNPSQ

>RO3T_00078

AQHLGEGQVGELSTYYMVSIQDPLFNKQWHLINQMNTGNDINVTGVWKQGISGKGVTVVIVDDGLDYNSTDLAANFYAEGSYDFNDHESLPTPKLWDDTHGTRCAGQIAAVNNACGIGIAYESKIAGVRILSGDLTDADEALALNYKYQENDIFSCSWGPTDNGETMEAPKGILADAFLNGIKNGRGGKGSIYVFATGNGGTSGDNCNFDGYTNSIYTITVGAIDFTNSHPPYSEACSAQLVQTTDVCSDRHGGTSAAAPNAAGIFALVLSVRPDLSWRDLQHLCVQTAVPSDWKRLPSGYNHKFGYGKLDAYALVEAAKHKGVNQQTWLSIVKVTEEMIKAAGLRLEHITATVNIEHQRRGDLTIDLLSPHQKSELATRRNLDTSTEGFPNWKFMSVKHWEENPVGDWTLTIYDNWTLTLYGEQDPEFVHPLDKYEFLGN

>RO3T_02460

YILFNGWPIYELTANYAYNLDGSSIKVGIIDSGDYTHPALGGCFGGCKVAYGYDLVQSPIDNCHGTFLSGLIAGVAPGVTLGMWKVYGCDILMAMEMAYQAGMDVINLSLGAIMANRIVVHVAVANGNGPNGFLSASPASGKDVIAVGSIMNDLCLLVHGASGFSSQGPTLQLKPEIVGVGGVFSTLPGTSMAAPSGQIALATQDSPIRQGAGIINVAQAFHASNDTFYNKHSSLTLHLTHQPSVIPPGHAIYGGYIPYLGGDYKSGTYQLKIKALRVFGDW

>RO3T_02879_gene_RO3G_02880_rory_supercontig_3.2:complement(join(1686525..16873051687358..16878771687931..16880531688230..1688236))

MLFKSIIAAVSAFAGLSAAAPANIRASNVIEGSYIVKLKDNVATDKHLSWVSGIHARRNVNNDVAGVEREYNSPAFHGYAGQFDQQTIVEIESSPEVAYVEADQIWTLEESMEKRDLTTQESAPWGLAAVSHRQPNATGYIYDTAAGQGTFGYVMDTGIRATHREFQGRASTGWTGWAGDDRDISGHGTHVAGTVGGVTFGVAKKATIIAVKVFHGTQTSTSIIMGGFDWAVNDIVSKGRQDKSVINQSLGGPFSRAWNDAIEAAFTRGVLSVIAAGNAQRDASEVSPASAPNAVTVGAVDRNWRIVTNWPLGQGSNYGPVLDIFAPGDGIESAEPNSDSQTGLRSGTSMASPHVAGLALYAMSVDGVRGAQAVTDHLIKNSGKGVVTGPLRGSPNRFANNGNPSQ

>RO3T_03816_gene_RO3G_03817_rory_supercontig_3.2:join(4056931..40573564057408..40574704057525..40577984057890..40580914058141..40582294058290..4058654)

MLNVKNLVLTAAAALASQAIAAPTGPDAGNAKIQAAQGGQVIPGKFIVTLKPGSKPAVLESHMRWVNGVHARASGDEAIKGVETMLDGIYGFMGYVGSFSEAVLAQIKAHPDVEAVEQDKIWTLDWITDDQQLEARDDDKEPPSSGGGSNFIQQKNATWGLGSVSHRAPYATEYGYQESAGKDTYAYVIDTGIRTTHEEFEGRASHAWSAYLTRTDNVGHGTHVAGTIGGKTYGVAKNAKLLAVKIFNSRSSSTSVILAGYNWAVNDIVRKGRTKRAAINMSLGGPKSTAFNTAVERASASGVLSIIAAGNEAQDASNVSPASAPSAITVAAINRDWTLASYSNFGSVVDICAPGSNITSAWNTGDSSEKTISGTSMATPHVVGLALYAISVDGATGVDGVTKHLLSTATKDKVAGDTRGSPNLIGNNNNPYQK

>RO3T_04301_gene_RO3G_04302_rory_supercontig_3.3:complement(join(231740..234218234274..234779234835..234919234977..235085235143..235395235456..235640235697..235780235893..235998))

MTHSSKPIQEYPVNGLMPKQDTQAASFIKKYPEYDGVDPGAAGMQVTTDGKPKLLDIVDCTGGGDVDTSKKVKPTTEDGLNVIEGQSGRKLILDSSWNNPSGEYRVGVKSAYELFPTELKNRIKAERRQNFIKKQAQLLSEAQRRLADYIKTTDKLDESEKSELEARVESLKNLDKNYEDPGVLLDCVLFFDGKDWRAVIDVDESGDLRGQPCLTDYRKELQYHRFGKADLLNFSVNIYNDGDILSIVTLSGSHGTHVAGITAANFPDEPALNGVAPGAQLISLRIGDARLGSMETGPGLTRAAAHLANHKVDLANMSYGESSGLPTDGHFIKLLANEAIGKSGCIFVTSAGNDGPCYSSIGAPAGMDASFITVGAYVKHSQMQAEYALLESVTERPFTWSSRGPTSDGYHGVDIYAPGSAITSIPVYVLNKLDLKNGTSMSSPNACGCIALLVSALKAQKEEYTPYRLKNAVVQTAKSVEDPLGVGFIQVDKAYEYLENYKDRKDLDLLFKVTVQKRGVQRGIYLREAEETNGIQYITTKVQPKFMGEFDPENPKYNEAKFNYEARVALIASESWITVPDYLYIHSGGNAFQVKVDPTSLSQNKFHYGEVLGYDTSSPERGPLFRVPVSVVKPQLPSNGSIEFKNIEFDPGFISRNFIQVPEGATSCELVIRSRAPAETSPARFMLHLLQLVPKQNQKGKHAYSFLLGDGSFGNPSSEEQIIKKHFSVRANLTLELCLAQFWSALGKHVVNISLNFHGIQITGNLANGQSTVHLEPQLTRLDISAPLRREDGLDVKVSFSKLRKYIRPAESTITPMHPCRDMLPSSRVLYQLVLRYNFTIDSATTITARFPTVMNQLYEHFLAGVFGIVYDTNRKVVGYLDVFDHNIKLSQKGEYTIMLQLSTEEENVLEKLKDVICELDLDLKAVNFNTFQNIADAYKNGNSTLTKFNLERKNIKVLYIAPPAGKDALPKDVKAGDALFGKLTFMSNVEGGQYKVIYTVPPLIVESKPSDEKDDKKPTEEEIERQLKNATRDLEISYLKKFEADSAAYKNLLSKLETEYANDINFLEYKLNSLWTASGSTDVDSLLTPGKLSEAQANEIIKISDTIISQLNERELSEFYGLKQAENETDEQKQKRKENDQKKKQLINALKQKAIAYAAISDAAENLDAFNASVKALQQWTSDDSSKDLASLLIKVKQERKAGHPGNALKAIEKYLSEASFTSTAVKDVAKVWKVRNEIYKELGWTLWSEYDDKWNIIRQPPYGFALF

>RO3T_05734

YIIFRGYPVHQMSVDQVHKNKGKGILVAVIDSGDYMHPALGQGFGDFKVVKGYDFVPDPMDSCHGTHVSGIIAGVAPEAKLAMYRVFGCDVVLAILKAYDEGADIINLSLSPVIEQMAVQVVIAAGNGKRGYSVGIPGTSLGAYTVASIENSACALIYGASTFTSVGSTLDLKPNIAAVGGVFSTLPGTSMATPSGSLALAIPDNPIRIGAGLVQVFDATHITNDTITNGQQTAQYDVVHQPSKLPPGHLFYGGSIPYFGGTYPDGTYKIRLSVLKLFGDW

>RO3T_05983_gene_RO3G_05984_rory_supercontig_3.4:join(1296817..12969701297030..12975491297604..1298390)

MLHLKNLAVFLAALAPFAAGAPIDKEPQVKVAGKYIVTLKPGIAARDIESHMGWVNSVHRRNVADGKEVAGVENKFDILDFHAYAGEFDEATLEEIRNSPDSSAPWGLASVSSRSSGATTYRYDSSAGSGVYAYIVDSGINTAHVDFEGRAVKGFNAAGGANEDTLGHGSHVAGTIGGKTYGVAKSVNLVDVKVFTGRSASTSTIISGFNWAVSDIQSKNRVGKAVINMSLGGPASTAFNSAVNNAFSAGILSIVASGNDGVRVTNESPASATNAFVVGAIDNTWREASFSNFGAEVDILAPGVNILSSWYNSSTATNTISGTSMATPHVVGVAAYLLGLESISTPAALRSRIIALGTTGKALGLKSQTPNRILFNGISA*

>RO3T_06131

REDYVAQQLGEGQVGELDTYYMVSIKDPLFDKQWHLINQKNPGNDINVTGVWKQGIAGKGVTVVILDDGLDFNSTDLADNFYAEGSYDFNDHEPLPKPKLWDDTHGTRCAGQIAAVNNACGVGIAYESKVAGIRILSGDLTDADEALALNYDYQHNHIFSCSWGPPDNGENMEAPKAILTDAIANGVRNGRDGKGSIYVFATGNGATLGDNCNFDAYTNSIYTITVGAIDHTNKHPAYSESCSAQLVVTYSSGSGIQTTDVCFDRHGGTSAAAPNAAAVPSDWKELPSGYNHKFGYGKLDAYALVEAAKFKSVNQQTWLSIVKVTEGMIKAAGLKLEHVTATVNIEHERRGDLVINLESPHLKSELATRRILDKSKDGILNWKFMSVKHWEEDPIGDWVLSVYDNWTLTLYGEQDPEFKYVVPTYEF

>RO3T_10127_gene_RO3G_10128_rory_supercontig_3.7:complement(join(2388757..23897582389803..2390147))

MVNNFQRLSVLVAALLPVGLAAPVQSEERDAESFGVVGASAKDSYIITLKDDLEARDVESHLDWLNEVQARALNKRDFPGVKKHYDIGAYHGYSGKFDEETLEAIKASPEVAAVEKDQIWTLFAVTSQSGAPYGLGSLSSRTGASTTYRYDDSAGQGTYAYVVDSGVQVGHSQFGGRATLGSNPAGGAHTDTSGHGTHVAGTIGGSTYGVAKRTNIISVKVFVGNTASTSVILSGFNWAANDIRTKARTTTSVVNLSLGGGFSSAFNNAVNAASAQGVVSVIAAGNENQNVANVSPASAASAIAVGAVDSAWAIASYSNWGAGVTIFAPGSNVLSAWIGSNTATRSISGTSMASPHVAGLVVYLQRLEGLASPAAAKARLIALATTGRVTGNLRGSPNRLAYNGVA*

>RO3T_10487_gene_RO3G_10488_rory_supercontig_3.8:join(699739..699892699947..700463700514..701291)

MVNNFQRLSVLVAALLPVGLAAPVQSEERDAESFGVVGASAKDSYIITLKDDLEARDVESHLDWLNEVQARALNKRDFPGVKKHYDIGAYHGYSGKFDEETLEAIKASPEVAAVEKDQIWTLFAVTSQSGAPYGLGSLSSRTGASTTYRYDDSAGQGTYAYVVDSGVQVGHSQFGGRATLGSNPAGGAHTDTSGHGTHVAGTIGGSTYGVAKRTNIISVKVFVGNSASTSVILSGFNWAANDIRTKARTTTSVVNLSLGGGFSSAFNNAINAASAQGVVSVIAAGNENQNVANVSPASAASAIAVGAVDSAWAIASYSNWGAGVTIFAPGSNVLSAWIGSNTATRSISGTSMASPHVAGLVVYLQRLEGLASPAAAKARLIALATTGRVTGNLRGSPNRLAYNGVA*

>RO3T_11851_gene_RO3G_11852_rory_supercontig_3.9:complement(join(2026289..20272182027274..2027633))

MAFLKRILPLLALILPAVFSATEQVPHPTIQTIPGKYIVTFKSGIDNAKIESHAAWVTELHRRSLEGRSTTEDDLPAGIERTYRIANFAGYAGSFDEKTIEEIRKHDHVAYVEQDQVWYLDTLVTERRAPWGLGSISHRGGSSTDYIYDDSAGEGTYAYVVDTGILATHNEFGGRASLAYNAAGGEHVDDVGHGTHVAGTIGGKTYGVSKNAHLLSVKVFVGESSSTSVILDGFNWAANDIVSKNRTSKAAINMSLGGGYSYAFNNAVENAFDEGVLSCVAAGNENRDAARTSPASAPDAITVAAINRSNARASFSNYGSVVDIFAPGEQVLSAWTGSNSATNTISGTSMATPHVTGLILYLMGLRDLATPAAATTELKRLATRNAVTNVAGSPNLLAYNGNSGVSKGGSDDGDED*

>RO3T_12236

FLIIHAWPVYNQTINKLRGLSGQGIKVGVIDTGDYTHPALGRCFGGCRVAYGYDFVPDPRDICHGTHVAGIIGGVASEGAYRIFGCDIIMAMERAYLDGMDVINLSLGSILADELSMIVCAAAGNGDRGFEVGSPSLGKHAISVASIDNDACVLVGGASSFSSWGLGLSLKPDISAPGGIYSTYPGTSMASPAGVVALGHPRQGAGLIDVYQATMITNDEIKNGRLDTEYTITHQASEANIYSGYIPYAGGDVFSGKYRLKLTALRPLGDF

>RO3T_12258

YIIFRGYPVHKTTVDRTRNLQGKGVVVGIIDSGDYRHPAFGNGFGGYPVSLGYDLVERPLDTCHGTHVAGIIAGIAPQVTLGAWRIFGCDLVIALIDAHEAGCDVINLSLGAIVANRVSSIVVAAAGNGIDGFYISAPGTGQGTVSVASVDNDACVLVQGASSFSSVGPLVSLKPDIAGPGGIFSTLPGTSMASPAGALALALPSHPVRQGTGLIQ

>RO3T_12286

YILFNGWPIYELTANYAYKLNGSGVKVGIIDSGDYTHPALGGCFGGCKVAYGYDLVKPPIDNCHGTFLAGIIAGVAPGVTLGMWKVYGCDIIVALEMAYKAGMDIINLSLGAEIVSRIVVHVVAAIGNGSNGFLPSSPASGKDVIAVGATMNDACMLIHGASGFSSQGPTLQLKPEIMGVGGVFSTYPGTSMAAPSGQIALATQDSPIRQGAGKIDVVQAFHAFNDTFYNKHNSLTLHLHHQPSVIPPGHAIYGGYIPYLGRNQ

>RO3T_15325_gene_RO3G_15326_rory_supercontig_3.16:join(135944..136240136294..137286)

MQSIKRTLLLLGAVLPAVLAGPIFPHRRAPTTIPGKYIVTFKSDVDQAAIDKHTAWATDIHKRNLQRRDSSEEDLPIGIERNFKINKFAAYSGSFDEDTIAQIRQSDEVAAVEEDQVWHLFDLTTQSDAPWGLGSISHKGQPSTDYIYDTNGGEGTYAYVVDTGINVDHEEFEGRASLAYNAAGGQHVDGVGHGTHVSGTIGGKTYGVAKKANLLSVKVFVGESSSTSIILDGFNWAANDIVSKKRTGKAAINMSLGGGYSKAFNDAVENAFNEGVLSIVAAGNENTDASSTSPASAPDAFTVAAINVNNTRAYFSNYGSVVDIFAPGQNILSAWIGSNTATNTISGTSMATPHIVGLSIYLMSLEDLSSPKAVSDRIKELATRGVVSNVAGSPNLLAYNGNA*

>RO3T_15504

YIVFKGAPVHHMTVDIVHKNKGKGVLVGILDSGDYKHPALGGGFGGYKVVTGYDLVPDPLDECHGTHVSGIIAGVAPEANLAMYRVFGCDIIVGLLMAYDAGVDVINLSLGSIVNQIVVHVVISAGNGAQGYTIGSPSTASSAFSVASVQNDACALVKGASSFSSLGPSLILKPNIAGVGGIYSTLPGTSMASPAGSVALASPDSPARAGAGLVQVYDATHITNDTVTNGSKTIQYELVNQVSKLAPGHIYYGGYIPYVGGTYPAGTYRFRVSALKLFGDW

>RO3T_16043_gene_RO3G_16044_rory_supercontig_3.18:complement(join(424499..425285425341..426157426209..426362))

MHSFKRSLLLLGALLPAVFGAPVEPRRAAEKVPGKYIVTFKSGLNVDQIDAHTSWASNVHKRNLERRGLAERDQYSGIEKNYKINKFAAYSGSFDDATIEEIRNSADVAHVEEDQIWYIDALTSQSGAPWGLGAISHKGEASTTYVYDTSAGEGTYAYVVDTGINADHEEFGGRASLAYNAVGGQHVDSVGHGTHVAGTIGGETYGVSKKANLLSVKVFQGESSSTSIILDGFNWAANDIVSKGRTGKSAINMSLGGGYSYAFNQAVEDAYDEGVLSVVAAGNDNIDASDSSPASAPNALTVAASTKSNTRASFSNYGSVVDIFAPGQDILSAWIGSTTATNTISGTSMATPHVVGLSLYLIALEGLSSASAVVSRIKELATQGVLSNVQGSPNLLAYNGADE*

>RO3T_16115_gene_RO3G_16116_rory_supercontig_3.18:join(622658..622676622743..622968623020..623144623221..623316623506..623701623763..623934)

MQSIKRTLLLLGALLPAALAAPAREPHPSSNIIPGKYIITFKSGIDTAAIESHTAWASNIHKRNLERRGLVGGEFPAGIERKFKIKDFAAYAGSFDPATIEEIRNSEDVAHVEEDQIWYLDALTTQSGAPWGLGSISHKGQASTNYVYDTSAGAGTYAYVVDSGINVDHIEFQGRATKAYNAVGGDHVDTLGHGTHVAGTIGGKTYGVAKQTNLLSVKVFEGRTGSTSVILDGFNWAANDIVSKGRKGKAAINMSLGGGYSYAFNNAVESAYEQGVLSVVAAGNEGVDASNSSPASAPNALTVGATNKSNARASFSNYGKVLDIFAPGQDILSAWIGSTTATNTISGTSMATPHVVGLAVYLMGLEGVSGPAAVTQRILQLATSGVISDVKGSPNKLAYNGAA*

>RO3T_16311

YIIFRGYPVHAITVDKVHKLTGKNIVVGIIDSGDYRHPAFGSGFGGFPVRYGYDLVEKPLDACHGTHVAGVIAGIAPQVTLGAWRIFGCDLVIALISAHEAGCDIINLSLGSIVANRVSSIVIAAAGNGNDGFYISAPGSGTSTVSVASTDNDACVLVQGASLFSSVGPLVSLKPDIAGPGGIFSTLPGTSMAAPTGAFALAQPDNPARQGAGLIQVFDAIHISNDTISNSKETVSFEISHEAGTLAPGATVSNGSYYLRWKALKLLSSW

>RO3T_16658

YMIFHGSPVHHMTVDLVHKNKGKGILVGVLDTGDYMLPALGGGFGGYKVVTGYDLVPDPLDACHGTHVSGIIAGVAPEANLAMYRVFGCDVIVALLMAYDAGADVINLSLGKVVNQIVVHVVISAGNGEDGYTLSSPSSARLAFSVASVENDACALVKGASSFSSMGPSLIFKPNIAGVGGVFSTLPGTSMASPAGSIALASPDSPIRAGAGLVQVYDTIHVSNDTVTNGNVTVQYRLTQLSSTLAPGHIYYGGYIPYIGGTYPAGVYQFRLSALKLFGDW

>SCY_0619.t1_gene_SCY_0619_Scer_YJM789_chr3_chrIII.Contig85:complement(188817..190292)

MQSIKRTLLLLGAILPAVLGAPVQETRRAAEKLPGKYIVTFKPGIDEAKIQEHTTWATNIHQRSLERRGATGGDLPVGIERNYKINKFAAYAGSFDDATIEEIRKNEDVAYVEEDQIYYLDGLTTQKSAPWGLGSISHKGQQSTDYIYDTSAGEGTYAYVVDSGVNVDHEEFEGRASKAYNAAGGQHVDSIGHGTHVSGTIAGKTYGIAKKASILSVKVFQGESSSTSVILDGFNWAANDIVSKKRTSKAAINMSLGGGYSKAFNDAVENAFEQGVLSVVAAGNENSDAGQTSPASAPDAITVAAIQKSNNRASFSNFGKVVDVFAPGQDILSAWIGSSSATNTISGTSMATPHIVGLSLYLAALENLDGPAAVTKRIKELATKDVVKDVKGSPNLLAYNGNA*

>SCY_1438.t1_gene_SCY_1438_Scer_YJM789_chr5_chrV.Contig317:complement(33742..35658)

MQSIKRTLLLLGAILPAVLGAPVQETRRAAEKLPGKYIVTFKPGIDEAKIQEHTTWATNIHQRSLERRGATGGDLPVGIERNYKINKFAAYAGSFDDATIEEIRKNEDVAYVEEDQIYYLDGLTTQKSAPWGLGSISHKGQQSTDYIYDTSAGEGTYAYVVDSGVNVDHEEFEGRASKAYNAAGGQHVDSIGHGTHVSGTIAGKTYGIAKKASILSVKVFQGESSSTSVILDGFNWAANDIVSKKRTSKAAINMSLGGGYSKAFNDAVENAFEQGVLSVVAAGNENSDAGQTSPASAPDAITVAAIQKSNNRASFSNFGKVVDVFAPGQDILSAWIGSSSATNTISGTSMATPHIVGLSLYLAALENLDGPAAVTKRIKELATKDVVKDVKGSPNLLAYNGNA*

>SCY_4566.t1

KDHQYSNSRLEEEHDVRGLPNHYVFSKRINDPLFERQWHLVNPSFPGSDINVLDLWYNNITGAGVVAAIVDDGLDYENEDLKDNFCAEGSWDFNDNTNLPKPRLSDDYHGTRCAGEIAAKNNFCGVGVGYNAKISGIRILSGDITTEDEAASLIYGLDVNDIYSCSWGPADDGRHLQGPNDLVKKALVKGVTEGRDSKGAIYVFASGNGGTRGDNCNYDGYTNSIYSITIGAIDHKDLHPPYSEGCSAVMAVTYSSGSGIHSSDICSNSHGGTSAAAPLAAGVYTLLLEANPNLTWRDVQYLSILSAVGDGDWRDSAMGYSHRYGFGKIDAHKLIEMSKWENVNAQTWFYLSVITISEKSLQDANKRIEHVTVTVDIDTEIRGTTTVDLISPAGISNLGVVRPRDVSSEGFKDWTFMSVAHWGENGVGDWKIKVSWRLKLFGESIDSSKTRRRYEFITEP

>SCY_5076.t1_gene_SCY_5076_Scer_YJM789_chr15_chrXV.Contig353:298543..299979

MLSIKRTLLLLGAVLPAVFGAPVQETRRAAQKIPGKYIVTFKPGTDTATIESHTLWATDLHKRNLERRDTTSGEPPVGIEKSYKIKDFAAYAGSFDDATIEEIRKSADVAHVEEDQIWYLDALTTQKGAPWGLGSISHKGQASTDYIYDTSAGAGTYAYVVDSGINVNHVEFESRASLAYNAAGGSHVDSIGHGTHVAGTIGGKTYGVAKKTNLLSVKVFQGESSSTSIILDGFNWAVNDIVSKGRTKKAAINMSLGGGYSYAFNNAVENAFDEGVLSVVAAGNENSDASNTSPASAPNALTVAAINKSNARASFSNYGSVVDIFAPGQDILSAWIGSTTATNTISGTSMATPHIVGLSVYLMGLENLSGPAAVTARIKELATNGVVTNVKGSPNKLAYNGNA*

>SJAG_02432T0_|_SJAG_02432_|_Schizosaccharomyces_japonicus_yFS275_vacuolar_serine_protease_Isp6_(459_aa)

MLSIKRTLLLLGAVLPAVFGAPVQETRRAAQKIPGKYIVTFKPGTDAATIESHTLWATDLHKRNLERRDATSGEPPIGIEKNYKIKDFAAYAGSFDDTTIEEIRKSADVAHVEEDQIWYIDALTTQKGAPWGLGSISHKGQASTDYIYDTSAGAGTYAYVVDTGINVNHVEFEGRASLAYNAAGGSHVDSVGHGTHVAGTIGGKTYGVAKKTNLLSVKVFQGESSSTSIILDGFNWAANDIVSKGRTRKAAINMSLGGGYSYAFNNAVENAFDEGVLSVVAAGNENTDASNTSPASAPNALTVAAINRSNARASFSNYGSVVDIFAPGQDILSAWIGSNTATNTISGTSMATPHIVGLSVYLMGLESLSGPAAVTSRIKQLATNGVVTNAQGSPNKLAYNGNA*

>SJAG_04397T0

SPAEFLGVRRMRNLPNYFVYSKRINDPLLSQQWHIINTNAIGHDLNVTGVWEEGYLGENVTVAFVDDGLDFRHADLQDAFSAVGSWDFNDDVPEPLPKLADDTHGTRCAGEVAAANDVCGVGIAPKAKVAGLRMLSGPVTDLMESEALNYGFDTNDIYSCSWGPADDGRAMEAPEPATRKALLNGVVNGRNGLGSVFVFASGNGGYYDDNCNFDGYTNSIFSVTVGAVDTEDSWPAYGEYCAAQLVSAYSSGHNIVTTNVCTHRHGGTSAAAPLGSAVYALALSARPELTWRDIQHITVYSALPSHKFGFGKLDAGRFIETAKWELVKPQTWYITLKFNMTRAMVHQSNQDLEHVTVRTTIPFSRRGKLQVVLRSPSDESVLATERPFDENAQGIQDWTFMTVQHWGEKPEGVWTLIVRDNWQLGLWGQASNASQTKSP

>SJAG_04785T0_|_SJAG_04785_|_Schizosaccharomyces_japonicus_yFS275_serine_protease_Psp3_(432_aa)

MVNFRNVLLGLLPFAAAAPVAPGTPDVANKYIVTLKPGISTDNVASHLNWVSDVHKRSLGRRDLAGVEKTYDIKNFHAYAGTFDESVVAELKNNPDVAAVEPDQIYTLSAYTTQSSAPWGLASISSRTSGATSYRYDNSAGAGTFAYVVDSGILTTHTNFGGRAIRGYNAAGGEWVDSIGHGTHVAGTIGSTTYGVAKAATLIDVKVFVGRTSSTSIILDGFNWAVNDIVSKNRASRSVINLSLGGPTSTAWTSAINAAYSSGILSVAAAGNENVAASTRSPANAPNALTVGAINSAWAEDTSYSNFGPSVDILAPGTNVLSLGYTSNTSTRTLTGTSMAAPHVAGLALYLAAFENINTPAALRNRIVALGTSGRATGIRGGSPNLIAYNGNA*

>SJAG_04928T0_|_SJAG_04928_|_Schizosaccharomyces_japonicus_yFS275_tripeptidylpeptidase_II_Tpp2_(1108_aa)

MRPSEFQINGFPVDGVVPKHETQAAEFLKKYPDFDGRGVVVGILDTGVDPGAAGLSITSDGKPKFKNIVDCTGAGDVETSKVVDAKSNGEYLEIEGLSGRTLRLSKEWKNPTGKWHIGSPMNDFDKKQEWSTFGPVDLLSYGVHVYEDGNITSIVTVCGTHGTHVAGIIGAHHPEHPELDGAAPGCQLVSLMIGDARLDSLETSHAFSRACAEIVKNKVDIINISFGEDAGLPNQGRVIELLRDELSGKRDVVIVSSAGNEGPAYTTVGAPGGTTFDIISVGAYVTGNMMQAQYNLLKPVNDTPYTWCSRGPTLDGDVGVSIYAPGGAITCVPPYSLQNSQLMNGTSMSSPSACGGISLILSALKAREIPYSASSIKKAVTFSAKSVRSEFEIGMLQVIEAYNYLVETKDTIDRDVSFKVSGPQGNRGIYLRESADFQEASRHTFTVSPVFYDGQESLKAHFEMQLTLSATEPWIQATEYIMMAGTGRSFAIRVDPTSLTPGFHFGKIRAYDAKSQQRRVVFEIPVTIMKPFEVTDNTLSLKSLTFEPSKIERRFITPPKGTTYAEIRIRPLCKLEASSMLWICTNQLLPQTKHKDSSSELILGIVENEVTSKTFKVNDGYTLELCLAQWWSSLEPMLLDIDVTFHGIKSTAGQSLCLNASAGYKRIDCFSLKKEPFKPKVVFDRFSDSYRPVSAVIKPLKFRDVLPDGQQLFETVITYKFDIKEKTEVTLKFGVPEHMYDNGFNGIFFMLFDAQKQLIHYGDMYPRPHELSKGEYTVRLQLISVYTQVLETFKDVPLTLGRKLKKEITLPLFANHIDFCDNKKADYGNATIALDRPKSFIIGTDLSADIYPSDVNPHSILMGSMKFNDKSSIPASMFLPPKVVEEKATTEDENEENLVRLEVDILSKLKGAEKDKHLKNLLSMHRKSLPVQLAKLDNAKDDQERLSTVDMILSLIDVSALSQYFSNEAKTSELAVKNVALENKMKEEKAAYIKALQVKSEVFSRQSNVDLEAFGKTVQTLMNWVEDSDTKLVNAKRALYTKSGQPALALQSLLKALEENGNSREGDVKTLLQEAITLCEELKWPFWKQVFEKLVIKKAPPYGYALF*

>SOCG_00573T0

DPADAIGVRPMRNLKNHYLFAKRLIDPLLSDQWHILNYEVPGHDLNLQEVWDAGILGENVTVAFVDDGIDFRHPDLQAAYSSLGSWDFNDNMADPLPKLSDDLHGTRCAGEVAGANDVCGVGIAPKAKVAGLRILSAPINDVVESEALNYGFQTNHIYSCSWGPADDGRAMEAPRLATRRALINGVINGRNGLGSVFVFASGNGGHYRDNCNFDGYTNSIFSITVGAVDMDHQVPFYSEICAAQLISAYSSGSRIATTNPCTKSHGGTSAAAPLASAVYALALSIRPDLTWRDIQHITVHSAVPDLEWTKTPAGFSHRFGFGKLDAKRFIDITRWQLVNPQTWVISSELAVTEEMVEKSNKSLEQITVKVSIPFTCRGAMTIELESPAGRSMLATLRPYDQNNEGFPEWTFMTVQHWSESIIGSWKLIVRDYWQLAFWGESQDPSLTNP

>SOCG_00777T0_|_SOCG_00777_|_Schizosaccharomyces_octosporus_vacuolar_serine_protease_Isp6_(475_aa)

MVNFRNVLLGLLPFAAAAPVAPGSPDVANKYIVTLKSGISTDNVASHLNWVSDVHKRSLGRRDLAGVEKTYDIKNFHAYAGTFDESVVAELKNNPDVAAVEPDQIYTLSAYTTQSSAPWGLASISSRTSGATSYRYDNSAGAGTFAYVVDSGILTTHTNFGGRAIRGYNAAGGEWVDSIGHGTHVAGTIGSTTYGVAKAATLIDVKVFVGRTSSTSIILDGFNWAVNDIVSKNRASRSVINLSLGGPTSTAWTSAINAAYSSGILSVAAAGNENVSASTRSPANAPNALTVGAINSAWAEDTSYSNFGPAIDILAPGTNVLSLGYTSNTSTRTLTGTSMAAPHVAGLALYLAAFENINTPAALRNRIVALGTSGRATGIRGGSPNLIAYNGNA*

>SOCG_01007T0_|_SOCG_01007_|_Schizosaccharomyces_octosporus_serine_protease_Psp3_(449_aa)

MPSFHQLSLLLGTILPAALAAPFASNHKLQEGVIHGKYIVTLKTDSDDTTVQSHLHWVEGVHRRSLSKRRIVGIQSTYNATNWHGYFGEFDEDTIKEIEASPEVAFIEPDHKIHLQGDYEDGKVYFWEQPGQISKRALTTQQGATWGLGTISHREPGFTTYTYDTSVGANSYAYVVDSGVQVNHSEFEGRAIAGYSVSPAAHVDTVGHGTHVAATIAGKTYGVAKKAQIISVKVSQGRESSNSASLQGFDWAVNDIVSKKRAGRSVINLSLGGPASQAWTAAIASAYKSGILAVVAAGSGDEDRNPLPTSRRSPANAPNALTVGAIDSDWNPTSFTNYGPEVDIMAPGDHIESAGIGSSNAVATMSGTSMACPHVAGLALYLQVKENLSAPAAVTKRIKALGTPGRISGNLNGSANLVAFNGVRQ*

>SOCG_04541T0_|_SOCG_04541_|_Schizosaccharomyces_octosporus_tripeptidylpeptidase_II_Tpp2_(1276_aa)

MQFSKIANPSFVTSRSFWEIRLHIKNAPHTIFSSTKSFFQPCKPRSFYFSRQYNTSMIPSDFSGNYFPFDGVVPKHETQAFEFLKKYPEYDGRGVTVGILDTGVDPGAPGLSVTTTGEAKFKNIVDCTGAGDVDTSLEVESLDNGTSNEYISVQGRSGRMLKLSKEWKNPSKKWRVGCKFGYDFFPTDLRKRLQGLETEDMNKNNRKLLQDVTDEYAKFKAKNPDTPTDKDKLLELKELEARIECLKEFGEKFEKNGPLYDIVTFHDGEHWNVVVDTNQSGDLIEQKPLRPFSVAQEWSTFGSKDLLSYGVHVYNNGDVTSLVAVSGTHGTHVGGIIGAHHPEKPDLNGAAPGCRLVSLMIGDGRLDSLETSHAFSRACTEIVKNKVDIINISFGEDAAVCDKGRVIELLRDELSGKRNVIIVSSAGNNGPAYTTVGAPGGTTTDVISVGAYVTGSMMQAQYSLLSNVRDTPYTWCSRGPTLDGDTGVSIYAPGGAITSVPPYSLQNSQLMNGTSMSSPSACGGIALILSALKAQNIPYTASGIKKAVMYTSKDVRDDFEVGMLQVDTAYNYLTESDAEAASSRCFTVKGNLGNSKRGIYLREVADLANPSRHTFSVAPKFEEGEETAKSQFEVQLSLATTQPWIQAPEYVMMAGTGRAISVRVDPTVLAPGFHFGKVRAFEATKASRRCLFEIPVTVMKPSTVVNANFSLRDVSFEPTLIRRNFLVPPKGATYAEIRVKAVSPLESSNMLWISTNQTLPQTKLKDASTELIMSVSDNEVTTKLIPLHDAYTLELCMAQWWSSLEPMTLDIDINFHGIQLLNGSEMTLHGDKGLERLDCVSIRRGKFKPEISLDKYTECYKPSESVIQPLGERDILPDNQQLFELINTYPLTVDEKLDLTADFSVPHNMYDNGFNGIFFMIFDSQKQRVHYGDMYSSSHSLEKGEYKYKIQLLSVDPIKLERFKNLTLKLTKKLKKSITLPLYSDHIDFCDNQKESFDRAVIEANIVKSFVVGTELEEYPSEINDKSVLNGTLKFNDCDKATVPITLIPSAKPNGKSSDAKETPNLVELQVELLSKLEGADKEKHLKYLQSTFKNSLEVQLARLDAAKEDNERLSVAETILSLVDQEALSRYYSSQKKVDDTIPKNTVLEKSTALQKDAFIKALEVKCSSYAKQPSKDATGYCNSYQLLLNWLEDSDSRVAVIKKDYYKSLSQYGLALKALRKQISDNGNSGKLDVSKLLDEEKDLVQKLGWSFWLDVSKVNSVKRVPPYGYELF*

>SPOG_01018T0_|_SPOG_01018_|_Schizosaccharomyces_cryophilus_serine_protease_Psp3_(449_aa)

MASLRRLALYLGALLPAVLAAPAVNYKLPEAVPNKFIVTLKDGASVDTDSHLTWVKDLHRRSLGKRSTAGVEKTYNIDSWNAYAGEFDEETVKQIKANPDVASVEPDYIMWLSDIVEDKRALTTQTGAPWGLGTVSHRTPGSTSYIYDTSAGSGTFAYVVDSGINIAHQQFGGRASLGYNAAGGDHVDTLGHGTHVSGTIGGSTYGVAKQASLISVKVFQGNSASTSVILDGYNWAVNDIVSRNRASKSAINMSLGGPASSTWATAINAAFNKGVLTIVAAGNGDALGNPQPVSSTSPANVPNAITVAALDINWRTASFTNYGAGVDVFAPGVNILSSWIGSNTATNTISGTSMATPHVVGLALYLQALEGLSTPTAVTNRIKALATTGRVTGSLNGSPNTLIFNGNSA*

>SPOG_01720T0_|_SPOG_01720_|_Schizosaccharomyces_cryophilus_vacuolar_serine_protease_Isp6_(475_aa)

MASLVRLALYLGAFLPAALAAPTAPSKGSDVIPGKYIVTLKPSASSAKVESHLQWVGDVHRRSLSKRDTAGIEHTFNIKNWNAYAGQFDEDTIKEIEASPEVAFVEPDKVVKLSFEQSSELSDRALTSQSGAPWGLGTISHRTSGSTSYIYDTSAGEGSYAYVVDSGVLISHSQFGGRAVAGYSIFSGANTDTLGHGTHVAGTIAGSTYGVAKKANIVSVKVFQGAEGTDSGVLAGFNWAVNDITSKGRAGKAVINLSLGGDASQAWVTAIDAAYNSGVLSVVAAGNGDENGNPLPVSSQSPANAANAITVAALTSAWKPTSFTNYGAGVDIFAPGQSILSAWIGSNSATNSISGTSMASPHVAGLALYLKVLEGLTTPASVASRIKALGTSGKITGTLSGSPNLIAYNGNGA

>SPOG_01932T0

DPADAIGVRPMRNLENHHLFTKRLNDPLLSEQWHILNYEKPGHDLNLREVWDAGILGENVTVAFVDDGIDFRHPDLQAAYTSLGSWDFNDNMADPLPKLSDDLHGTRCAGEVAASNDVCGVGIAPKAKVAGVRILSAPINDVVESEALNYGFQTNHIYSCSWGPADDGRAMEAPRLGTRRALVNGVINGRNGLGSVFVFASGNGGHYRDNCNFDGYTNSIFSVTVGAVDIEHQVPFYSELCAAQLVSAYSSGSHIATTNPCTKSHGGTSAAAPLASAVYALALSIRPDLTWRDIQHITVHSAIPNVEWTKTPAGFSHKFGFGKLDAKRFIETASWQLVNPQTWLVSSEYVVTEEMIEKSNKGVEQITIKVSIPFTCRGAMTIELESPAGRSMLASLRPYDQNNEGFPEWTFMTVQHWSEPILGSWKLIVRDYWQLAFWGESRDPSLTSP

>SPOG_03716T0_|_SPOG_03716_|_Schizosaccharomyces_cryophilus_tripeptidylpeptidase_II_Tpp2_(1276_aa)

MQFSKIAKPSFIASRRFCEIRLHIKKAPQTILSSTKPSIRPCKSQSLCFFRQYNTSMIPSDFSSNYFPFDGVVPKHETQAYEFLKKYPEYDGRGVTVGILDTGVDPGAPGLSVTSTGQAKFKNVVDCTGAGDVDTSLEVESLDNATSNEYITVQGRSGRTLKLSKEWKNPTKKWRVGCKLGYDFFPSDLRERLQGLETEDMNKNNRKLLQDATEEYAKFKAENPETPTDKDKLLKLKELEARIECLKQFSDDFKKNGPLFDIITFHDGEHWQVVVDTSQSGDLVQQKPLRPFSVAQEWGTFGSKDLLSYGVHVYDNGNVTSIVAVSGTHGTHVAGIIGAHHPEKPDLNGAAPGCRLVSLMIGDGRLDSLETSHAFSRACTEIIKNEVDIINISFGEDAGVCDKGRVIELLRDELSGKRNVVIVSSAGNNGPAYTTVGAPGGTTTDVISVGAYVTGSMMQAQYSLLSNVRDTPYTWCSRGPTLDGDTGVSIYAPGGAITSVPPYSLQNSQLMNGTSMSSPSACGGISLILSALKAQNISYTASAVKKAVIYTSKDVRDDFDVGMLQVDAAYNYLTESDAEAASSRSFTIKGNIGNNKRGVYLREVTDLASPSRHTFSVAPKFEEGEEAAKSQFEVQISLAATQPWIQAPEYVMMAGTGRAIPVRVDPTALVPGFHFGKVKAFEATKDSRRCLFEIPVTVMKPSAVVNANFPLRNVSFEPTLIRRYFLVPPKGATYAEIRVKAISPLESSNMLWISTNQALPQTKLKDASTELIMSVSDNEVTTKLVPLHDTHTLELCMAQWWSSLEHMTLDIDINFHGIKVLNGSEITLHGNKGLERLDCISVRREKLKPEISFDKFTESYKSTESVIQPLGERDVLPDNQQLFELVNSYSLTVEEKSDLTADFSVPHNMYDNGFNGFFVMIFDSQKQRVHYGDMYTSSHSLEKGEYVYKVQLLSVDPIKLERFKNMTLKLSKKLKKSITLPIYSDHIDFCDNRKESFDRSVIEANIIKSFIVGTDFEEYPSEISDKSVLTGTLKFNDCDKASVPVYLIPSVKPVEKPSTSKESPNLVQLQVDLLSKLEGADKDKHLKYLQSTYKNSLEVQLAKLDAAKEDNERLSAADSILSLIDQEGLSRYYSSQKKVEDTIPKDTALEKNTSMQKDAFIKALEVKCSLYAKQPGKDTDSYSKSYQLLLNWLEDSDSRVSVIKKDYYKSLSQYGLALKALMKLINENGNSGKSDITKLLDEEKDLLQKLGWTYWYDVSYVDSVKRIPPYGYELF*

>Spom_972h:SPAC1006.01.t1_gene_SPAC1006.01_Spom_972h_chrI:5061690..5063045

MASIRRLALFLGALLPAALAAPVDGPVRRQETAIPNKYIITLKPEASDSKISAHLDWVGDVHRRSLSKRDTAGVEETYNISNWNAYAGEFDEATIEEIKSSPEVAFVEPDFEAYLWVEESSELSDRALTTQTGATWGLGTISHRTSGSTSYIYDTSAGSGSYAYVVDSGVQVGHSQFGTRASLGFNAFSGAHTDTLGHGTHVAGTIAGSTYGVAKQANIISVKVFQGNQGSTSTILSGFNWAVNDITSKGRQGRSVINLSLGGESSQTWISAINAAYNSGVLSVVAAGNGDINGNPLPVSSQSPANAPNALTVGAIDSSWRPASFTNYGAGVDIHAPGVSVLSSYIGSNTATRSLSGTSMACPHVAGLALYLQVLEGLSTPAAVTNRIKALGTSGRITGTLNGSPNLVAYNGNGA*

>Spom_972h:SPAC4A8.04.t1_gene_SPAC4A8.04_Spom_972h_chrI:2545350..2546753

MTSFRRLALALGALLPAVLAAPTEKRQELTAAPDKYIITLKPEATENKIEAHLNWVSDVHRRSLNKRDTSGVEKKFNISSWNAYSGEFDKATIDEIKKSPEVAFVEPDYTVYLDFETELTDRALTTQSGAPWGLASISRRTSGGSTYTYDTTAGSGSYGYVVDSGINVNHRDFGGRASLGYNAAGGAHVDTLGHGTHVAGTIASSTYGVAKAANVISVKVFTGNSASTSTILAGFNWAVNDITSKGRAGRSVINMSLGGPSAQTWTTAINAAYNSGVLSVVAAGNGDDLGRPLPVSGQSPANVPNALTVAAIDSSWRTASFTNYGAGVDVFAPGVGILSTWYTSNTATNSISGTSMACPHVAGLALYLQVLEGLSTPAAVTNRIKALATTGRVTGTLNGSPNLIAFNGAST

>Spom_972h:SPAP8A3.12c.t1_gene_SPAP8A3.12c_Spom_972h_chrI:complement(5330665..5334489)

MKFRLNANFNFSFRRYCFVQCRNKYHSHVRYLSSAKKSGILRNSYNQRTERYFTNIMIPSDYSNKFYPVDGVVPKHETQAYEFLKKFPEYDGRGVTVGILDTGVDPGAPGLSVTTTGLPKFKNIVDCTGAGDVDTSVEVAAADSNDYLTITGRSGRTLKLSKEWKNPSKKWKVGCKLAYEFFPKDLRKRLQKLETEDMNKSNRKLLQDATDEYAKFKDKFPEAPLDKDNLQTQKELEARIECLKQLAEKFDNPGPLYDVVVFHDGEHWRVVIDSDQTGDIYLHKPLADFNVAQEWSTFGSLDLLSYGVHVYDNGNITSIVAVSGTHGTHVAGIIGANHPETPELNGAAPGCQLVSLMIGDGRLDSLETSHAFSRACSEIIKNEVDIINISFGEDAGIPNKGRVIELLRDELAGKRNVVIVSSAGNNGPAYTTVGAPGGTTFDVISVGAYVTSGMMQAQYNLLSTVHDTPYTWCSRGPTLDGDTGVSIYAPGGAITSVPPYSLQNSQLMNGTSMSSPSACGGISLILSALKAQKKPYTAAAIKKAVMYTSKDLRDDFNTGMLQVDNAYEYLAQSDFQYTGARSFTINGNIGNSKRGVYLRNPTEVCSPSRHMFNVAPKFEDGEEYEKSHFEVQLSLATTQPWIQAPEYVMMAGTGRGIPVRVDPTALAPGHHFGKVLAYDASNESRRCVFEIPVTVMKPSSISNTCFSLRDVSFEPTLIKRHFLVPPKGATYVEIRVKATSELESTNMLWISVNQTIPQTKLNEASTELIMPVTQNEVTTKLVSIDDSYTLELCMAQWWSSLEPMVLDIDVNFHGIKVVNGKEINLISSQGLKRVDCASIRRENFKPDITLKDYVDSFKPTNTVIKPLGDRDIMPDGQQLFELMATYSVEISEKTELKADFAVPHNMYDNGFNGLFFMVFDSQKQRVHYGDMYTSSHTLEKGEYLYKFQLLSVDPSTLERFRNVTLRLTKKLKKPITLPLYADHIDFCDNKTYERENIDAGVVESFVVGTNIEGEQYASELKENSLLTGELKFGDCEKGTVPVTLVLPPKISTKEDTKLGEKCANIVQLQVDLLSKLADQEKEKHLKYLQSSYKNSLEVQLAKLDIVKETNERLSTADSILSLIDTEALSRYYSCQQKVEDTIPRDVVLEKKMALQRDAFIRALVVKCETFSTQGHKDKDNYFQNYQLLLNWLENSDPRVWQIKKDYYKSQNQYGLALKALLELLKENGNSGKMDVAKLLSEEKELLVNLGWNYWHDIVFVETVKRVPPYSYALF*

>Spom_972h_SPAC22E12.09c.t1

DPAEAIGVRPLLNLKYHHLIKKRISDPLFYGQWHIFNSNNPGHDLNLREVWDAGYFGENVTVAFVDDGIDFKHPDLQAAYTSLGSWDFNDNIADPLPKLSDDQHGTRCAGEVAAANDVCGVGIAPRAKVAGLRILSAPITDAVESEALNYGFQTNHIYSCSWGPADDGRAMDAPNTATRRALMNGVLNGRNGLGSIFVFASGNGGHYHDNCNFDGYTNSIFSATIGAVDAEHKIPFYSEVCAAQLVSAYSSGSHILTTNPCTRSHGGTSAAAPLASAVYALALSIRPDLSWRDIQHITVYSASPNAEWQKTPAGFSHHFGFGKLDASKFVEVAKWQVVNPQTWLIASEFTVTKDMIEKSNKRLEHVTVRVCIPFNRRGALEILLESPSGRSILASERPYDENSKGFLDWTFMTVQHWAEPPEGVWKLLVNDNWQLALWGESENPSNTKAP

>sros_2788_gene_gw1.11.84.1_sros_scaffold_11:join(419628..419999420082..420188420272..420656420737..420939421033..421084)

MTSIRRLALALGALLPAVLAAPADILSKRQAVPDKYIITLKPDASDSSVAAHLNWVGDVHRRSLNKRDTSGVEKTFNISSWSAYSGEFDKSTIAEIKKSPEVAFVEPDYTMYLSYEESEPELADRALTTQSGAPWGLGTISHRTSGSTSYIYDTTAGQGSYAYVVDSGVQVSHTNFGGRASLGYNAVGGAHEDTLGHGTHVAGTIAGTTYGVAKRANIISVKVFAGREGSTSTILAGFNWAVNDITSKSRAGRSVINLSLGGPASQTWTSAINAAYNSGVLSVVAAGNGDDAGRPLPVSGQSPANAPNALTVAAIDSSWRPASFTNYGAGVDVFGPGVNILSTWIGSNSATNTISGTSMACPHVAGLALYLQVLEGLSTPASVTNRIKSLATTGRITGTLSGSPNSVAYNGNGA

>sros_4401_gene_gw1.2.620.1_sros_scaffold_2:complement(join(1205355..12054601205538..12059781206050..12061441206219..12063031206374..1206483))

MTSFRRLALALGALLPAVLAAPADILSKREAVPDKYIITLKPDASDSSVAAHLNWVGDVHRRSLNKRDTSGVEKTFNISSWSAYSGEFDKSTIAEIKKSPEVAFVEPDYTMYLSYEESEPELTDRALTTQSGAPWGLGTISHRTSGSTSYIYDTTAGQGSYAYVVDSGVQVSHTNFGGRASLGYNAVGGAHEDTLGHGTHVAGTIASSTYGVAKQANIISVKVFAGSSGSTSTILAGFNWAVNDITSKSRAGRSVINLSLGGPASQTWTSAINAAYNSGVLSVVAAGNGDQFGRPLPVSGQSPANAPNALTVAAIDSSWRPASFTNYGAGVDVFGPGVNILSTWIGSNSATNTISGTSMACPHVAGLALYLQVLEGLSTPASVTNRIKSLATTGRITGTLSGSPNSVAYNGNGA

>sros_784

RTYAYLEAEALGVEQVGELRDHYLIRQRILDPLWPKQWHLVNGVIEENSINVTGVWDQGVFGKGVNVAIVDDGLDMHSDDLAANFHAEGSWDYNDNTPLPEPRLSDDQHGTRCAGEIAAVNDVCGVGVAHQAGIAGIRILSASISDADEASSLNYGYQTNDIYSCSWGPPDDGRSMEAPGRLITKAMLNGVTNGRGGKGSVFVFASGNGGAVDDQCNFDGYTNSLMSITVGAIDRKGLHPFYSEACAANMVVTYSSGSGIHTTDVCTDRHGGTSAAAPIAAGIFALVLEARPDLTWRDMQHLCVRTAVQPDWQMTASGYNHKYGFGKLDAWAIVNAARWQIVKPQTWWNSLTTGGSRITSDGIASSRLRNRISKMPTSKSLSTSRSLSLSSMNKSNXXARKAXRVFSRGREGLMRRRRVCWVGSSXLSRPEHSTAGRCNSGARRGSSSPF

>SS1G_03282_gene_SS1G_03282_sscl_supercontig_1.4:complement(join(484489..485682485937..486311))

MVNFKNLAVAATSLLGLANAAPTAKVNSDEVIPGKYIVTLKSDIAASKIESHLNWVGDVHKRGLNERAEKGVERTYNGKYGFHGYAGSFDKDTIKEIKENPDVALVEEDRVWTINWVDEPEKESLSKRAETTQSSATWGLGTVSHRSKGSTSYIYDTNAGSGTYAYIVDTGIITSHNEFEGRAQAVYTAFSGQNADTNGHGTHVAGTIAGKTYGVAKKATIQAVKVFQGSSSSTSIILAGFNWAANDIISKGRTKTSVVNMSLGGGYSASFNNAVESASSSGIISAIAAGNDGANAANTSPASAPSAITVGAIDSNWAIASYSNYGTVLDIFAPGTSVLSAWYTSNSATNTISGTSMATPHIAGLVLYGISVKGVSGVSGVTNWLTSTATSGKITGNLRSSPNLIGNNGNSLQ

>SS1G_04958
[truncated: 98,570 more chars]
