# Supplementary material for: Phylogenomic evolutionary surveys of subtilase superfamily genes in fungi
Source: Sci Rep. 2017 Mar 30;7:45456. doi: 10.1038/srep45456 (PMC5371821; doi:10.1038/srep45456)
Supplement: Supplementary Table S3 [file srep45456-s3.docx]

**Phylogenomic evolutionary surveys of subtilase superfamily genes in fungi**

Juan Li*, Fei Gu, Runian Wu, JinKui Yang and Ke-Qin Zhang*

*State Key Laboratory for Conservation and Utilization of Bio-Resources in Yunnan*, *Yunnan University*, *Kunming*, *650091*, *P.R. China.*

* Corresponding author: Juan Li and Ke-Qin Zhang

Tel: 86-871-65033805; Fax: +86-871-65034838.

E-mail address: [juanli@ynu.edu.cn](mailto:juanli@ynu.edu.cn) (Juan Li); kqzhang@ynu.edu.cn(Ke-Qin Zhang)

**Supplementary Table S3:** **Subtilase superfamily genes in 83 fungal organisms.**

| Species | strain information | lifestyles | Taxonomy | proteinase K-like | Pyrolisin | Kexin | S53 | OSP | new 3 | new 1 | new 4 | new 2 | Total |
| --- | --- | --- | --- | --- | --- | --- | --- | --- | --- | --- | --- | --- | --- |
| *Phaeosphaeria nodorum* | *SN15* | phytopathogenic | Ascomycota:Pezizomycotina:Dothideomycetes | 5(2)* | 1(1) | 1 | 5 | / | / | 1 | / | / | 13(3) |
| *Pyrenophora tritici-repentis* | */* | phytopathogenic | Ascomycota: Pezizomycotina:Dothideomycetes | 5 | 3 | 1 | 4(1) | / | / | / | / | / | 13(1) |
| *Aspergillus clavatus* | *NRRL_1* | human/animal pathogenic | Ascomycota: Pezizomycotina:Eurotiomycetes | 2 | / | 1 | 1 | 1 | / | / | / | / | 5 |
| *Aspergillus flavus* | *NRRL3357.20* | human/animal pathogenic | Ascomycota:Pezizomycotina: Eurotiomycetes | 2 | / | 1(1) | 2 | 1 | 1 | / | / | / | 7(1) |
| *Aspergillus fumigatus* | *Af293* | human/animal pathogenic | Ascomycota: Pezizomycotina:Eurotiomycetes | 3 | / | 1 | 2 | / | / | / | 1 | / | 7 |
| *Aspergillus fischeri* | *NRRL_181.14* | human/animal pathogenic | Ascomycota:Pezizomycotina: Eurotiomycetes | 3 | / | 1 | 2 | / | / | / | 2 | / | 8 |
| *Aspergillus nidulans* | *FGSC_A4.04* | saprophytic | Ascomycota: Pezizomycotina:Eurotiomycetes | 2 | / | 1 | 1 | / | / | / | / | / | 4 |
| *Aspergillus niger* | *CBS_51388.04* | saprophytic | Ascomycota: Pezizomycotina:Eurotiomycetes | 2 | / | 1 | 3 | / | 3 | 1 | / | / | 10 |
| *Aspergillus oryzae* | *RIB40.23* | saprophytic | Ascomycota:Pezizomycotina: Eurotiomycetes | 2 | / | 1 | 2 | / | / | / | / | / | 5 |
| *Aspergillus terreus* | *NIH2624.14* | saprophytic | Ascomycota:Pezizomycotina: Eurotiomycetes | 2 | 1 | 1 | 1 | / | 1 | / | / | / | 6 |
| *Penicillium chrysogenum* | *Wisconsin_54-1255.01* | saprophytic | Ascomycota:Pezizomycotina:Eurotiomycetes | 2 | / | 1 | 2 | / | / | / | / | / | 5 |
| *Penicillium marneffei* | *ATCC_18224.17* | saprophytic | Ascomycota:Pezizomycotina: Eurotiomycetes | 1 | / | 1 | 3(1) | 1 | 1 | 1 | / | / | 8(1) |
| *Coccidioides immitis* | *rs_3* | human/animal pathogenic | Ascomycota:Pezizomycotina: Eurotiomycetes | 14 | / | 1 | 3 | / | / | / | / | / | 18 |
| *Ccoccidioides posadasii* | *rmscc_3488* | human/animal pathogenic | Ascomycota:Pezizomycotina: Eurotiomycetes | 14 | 0(1) | 1 | 3 | 1 | / | / | / | / | 19(1) |
| *Microsporum canis* | *CBS_113480* | human/animal pathogenic | Ascomycota:Pezizomycotina: Eurotiomycetes | 12 | / | 1 | 4 | 1 | / | 2(1) | / | / | 20(1) |
| *Paracoccidioides brasiliensis* | *pb18* | human/animal pathogenic | Ascomycota:Pezizomycotina:Eurotiomycetes | 2 | / | 1 | 2 | 1 | / | / | 2 | / | 8 |
| *Blastomyces dermatitidis* | *er-3* | human/animal pathogenic | Ascomycota:Pezizomycotina: Eurotiomycetes | 2 | / | 1 | 2 | 1 | / | / | 1 | / | 7 |
| *Histoplasma capsulatum* | *nam1_* | human/animal pathogenic | Ascomycota:Pezizomycotina: Eurotiomycetes | 2 | / | 1 | 2 | 1 | / | / | 1 | / | 7 |
| *Microsporum gypseum* | *CBS_118893* | human/animal pathogenic | Ascomycota:Pezizomycotina: Eurotiomycetes | 11(1) | / | 1 | 2 | 1 | / | / | / | / | 15(1) |
| *Uncinocarpus reesii* | */* | saprophytic | Ascomycota:Pezizomycotina: Eurotiomycetes | 15 | 0(1) | 1 | 3 | 2 | / | / | / | / | 21(1) |
| *Trichophyton rubrum* | *CBS_118892* | human/animal pathogenic | Ascomycota:Pezizomycotina: Eurotiomycetes | 12 | / | 2 | 3 | 1 | / | 0(1) | / | / | 18(1) |
| *Trichophyton tonsurans* | *CBS_112818* | human/animal pathogenic | Ascomycota:Pezizomycotina: Eurotiomycetes | 12(1) | / | 3 | 3 | 1 | / | 0(1) | / | / | 19(2) |
| *Trichophyton equinum* | *cbs127.97* | human/animal pathogenic | Ascomycota:Pezizomycotina: Eurotiomycetes | 12 | / | 2 | 3 | 1 | / | 0(1) | / | / | 18(1) |
| *Chaetomium globosum* | *CBS_14851* | human/animal pathogenic | Ascomycota:Pezizomycotina: Sordariomycetes | 4(1) | 1 | 1 | 1 | / | 2 | 1(2) | / | / | 10(3) |
| *Epichloë festucae* | *Fl1* | endophytic | Ascomycota:Pezizomycotina: Sordariomycetes | 8 | 4 | 2 | / | 1 | / | / | / | / | 15 |
| *Fusarium graminearum* | */* | phytopathogenic | Ascomycota:Pezizomycotina: Sordariomycetes | 11 | 3 | 1 | 1 | 1 | 3(1) | 1(1) | / | / | 21(2) |
| *Fusarium oxysporum* | *CBS_4286* | phytopathogenic | Ascomycota:Pezizomycotina: Sordariomycetes | 12(2) | 2(2) | 1 | 1 | 1 | 1(2) | 1(2) | / | / | 19(8) |
| *Fusarium solani* | */* | phytopathogenic | Ascomycota:Pezizomycotina:Sordariomycetes | 6 | 8 | 1 | 1(1) | / | 4 | 8(1) | / | / | 28(2) |
| *Fusarium verticillioides* | */* | phytopathogenic | Ascomycota:Pezizomycotina: Sordariomycetes | 11(2) | 1(2) | 1 | 2(1) | 2 | 1(1) | 1 | / | / | 19(6) |
| *Magnaporthe grisea* | *70-15* | phytopathogenic | Ascomycota:Pezizomycotina: Sordariomycetes | 6(1) | 16 | 1 | 1 | / | / | 0(1) | / | / | 24(2) |
| *Magnaporthe poae* | *ATCC_64411* | phytopathogenic | Ascomycota:Pezizomycotina: Sordariomycetes | 3(1) | 9(1) | 1 | / | / | 1(1) | 16(3) | / | / | 30(6) |
| *Metarhizium acridum* | *CQMa_102* | entomopathogenic | Ascomycota:Pezizomycotina: Sordariomycetes | 15(2) | 7(1) | 1 | 2 | 1 | 0(1) | 2(1) | / | / | 28(5) |
| *Metarhizium anisopliae* | *ARSEF 23* | entomopathogenic | Ascomycota:Pezizomycotina: Sordariomycetes | 19 | 9(3) | 1 | 5 | 2 | 2(1) | 5 | / | / | 43(4) |
| *Metarhizium robertsii* | */* | entomopathogenic | Ascomycota:Pezizomycotina: Sordariomycetes | 19(1) | 11(1) | 1 | 4 | 3 | 3(1) | 7 | / | / | 48(3) |
| *Verticillium dahliae* | *vdls.17* | phytopathogenic | Ascomycota:Pezizomycotina: Sordariomycetes | 6 | 6(4) | 1 | / | / | 1 | / | / | / | 14(4) |
| *Verticillium albo-atrum* | *vams.102* | phytopathogenic | Ascomycota:Pezizomycotina: Sordariomycetes | 4(2) | 10(2) | 1 | / | / | 1 | / | / | / | 16(4) |
| *Neurospora crassa* | *OR74A* | saprophytic | Ascomycota:Pezizomycotina: Sordariomycetes | 3 | 1 | 1 | 3 | / | 1 | / | / | / | 9 |
| *Podospora anserina* | *DSM_980.24* | saprophytic | Ascomycota:Pezizomycotina: Sordariomycetes | 6(2) | 1 | 1 | 2 | / | 2 | 1 | 1 | / | 14(2) |
| *Trichoderma reesei* | *fgenesh5?* | saprophytic | Ascomycota:Pezizomycotina: Sordariomycetes | 5 | 5 | 1 | 5 | / | 3 | 3 | / | / | 22 |
| *Botryotinia fuckeliana* | *B0510.01* | phytopathogenic | Ascomycota:Pezizomycotina: Leotiomycetes | 1(1) | 1 | 1 | 2 | / | / | / | / | / | 5(1) |
| *Sclerotinia sclerotiorum* | *1980* | phytopathogenic | Ascomycota:Pezizomycotina: Leotiomycetes | 2 | 1 | 1 | 4 | / | / | / | / | / | 8 |
| *Arthobotrys oligospora* | *ATCC_24927* | nematophagous | Ascomycota:Pezizomycotina: Orbiliomycetes | 19(1) | 2 | 1 | 1 | 1 | 2 | 2 | / | / | 28(1) |
| *Pichia pastoris* | */* | saprophytic | Ascomycota: Saccharomycotina | 3 | 1 | 1 | / | / | / | / | / | / | 5 |
| *Zygosaccharomyces rouxii* | *CBS_732* | saprophytic | Ascomycota: Saccharomycotina | 3 | / | 1 | / | / | / | / | / | / | 4 |
| *Lachancea thermotolerans* | *CBS_6340* | saprophytic | Ascomycota: Saccharomycotina | 3 | / | 1 | / | / | / | / | / | / | 4 |
| *Ashbya gossypii(Eremothecium gossypii)* | *ATCC_10895* | phytopathogenic | Ascomycota: Saccharomycotina | 2 | / | 1 | / | / | / | / | / | / | 3 |
| *Candida albicans* | *SC5314* | human/animal pathogenic | Ascomycota: Saccharomycotina | 4 | / | 1 | / | / | / | / | / | / | 5 |
| *Candida dubliniensis* | *CD36* | human/animal pathogenic | Ascomycota: Saccharomycotina | 4 | / | 1 | / | / | / | / | / | / | 5 |
| *Candida glabrata* | *CBS_138* | human/animal pathogenic | Ascomycota: Saccharomycotina | 4 | / | 1 | / | / | / | / | / | / | 5 |
| *Candida lusitaniae* | *ATCC 42720* | human/animal pathogenic | Ascomycota: Saccharomycotina | 3 | / | 1 | / | / | / | / | / | / | 4 |
| *Candida parapsilosis* | *CDC 317* | human/animal pathogenic | Ascomycota: Saccharomycotina | 4 | / | 1 | / | / | / | / | / | / | 5 |
| *Candida tropicalis* | *MYA-3404* | human/animal pathogenic | Ascomycota: Saccharomycotina | 4 | / | 1 | / | / | / | / | / | / | 5 |
| *Candida guilliermondii* | *ATCC_6260* | saprophytic | Ascomycota: Saccharomycotina | 3 | / | 1 | / | / | / | / | / | / | 4 |
| *Debaryomyces hansenii* | *CBS_767* | saprophytic | Ascomycota: Saccharomycotina | 3 | / | 1 | / | / | / | / | / | / | 4 |
| *Kluyveromyces lactis* | *NRRL_Y-1140* | saprophytic | Ascomycota: Saccharomycotina | 2 | / | 1 | / | / | / | / | / | / | 3 |
| *Lodderomyces elongisporus* | *NRRL_YB-4239* | saprophytic | Ascomycota: Saccharomycotina | 3 | / | 1 | / | / | / | / | / | / | 4 |
| *Pichia stipitis* | *CBS_6054* | saprophytic | Ascomycota: Saccharomycotina | 3 | / | 1 | / | / | / | / | / | / | 4 |
| *Saccharomyces cerevisiae* | *YJM789* | saprophytic | Ascomycota: Saccharomycotina | 3 | / | 1 | / | / | / | / | / | / | 4 |
| *Saccharomyces kluyveri* | *CBS_3082* | saprophytic | Ascomycota: Saccharomycotina | 1 | / | / | / | / | / | / | / | / | 1 |
| *Saccharomyces kudriavzevii* | */* | saprophytic | Ascomycota: Saccharomycotina | 2 | / | / | / | / | / | / | / | / | 2 |
| *Saccharomyces mikatae* | */* | saprophytic | Ascomycota: Saccharomycotina | 3(1) | / | / | / | / | / | / | / | / | 3(1) |
| *Saccharomyces castellii* | */* | saprophytic | Ascomycota: Saccharomycotina | 3(3) | / | 1 | / | / | / | / | / | / | 4(3) |
| *Vanderwaltozyma polyspora* | *DSM_70294* | saprophytic | Ascomycota: Saccharomycotina | 5 | / | 1 | / | / | / | / | / | / | 6 |
| *Yarrowia lipolytica* | *CLIB122 W29* | saprophytic | Ascomycota: Saccharomycotina | 16 | / | 1 | / | / | / | / | / | / | 17 |
| *Schizosaccharomyces japonicus* | *yfs275* | saprophytic | Ascomycota: Taphrinomycotina | 2 | / | 1 | / | / | / | / | / | 1 | 4 |
| *Schizosaccharomyces pombe* | *972h* | saprophytic | Ascomycota: Taphrinomycotina | 2 | / | 1 | / | / | / | / | / | 1 | 4 |
| *Schizosaccharomyces cryophilus* | *oy26* | saprophytic | Ascomycota: Taphrinomycotina | 2 | / | 1 | / | / | / | / | / | 1 | 4 |
| *Schizosaccharomyces octosporus* | *yfs286* | saprophytic | Ascomycota: Taphrinomycotina | 2 | / | 1 | / | / | / | / | / | 1 | 4 |
| *Cryptococcus neoformans* | *grubii_h99* | human/animal pathogenic | Basidiomycota: Agaricomycotina | 1 | 1 | 1 | / | / | / | / | / | / | 3 |
| *Laccaria bicolor* | */* | saprophytic | Basidiomycota: Agaricomycotina | 2 | 1 | 2 | 3 | / | / | / | / | / | 8 |
| *Phanerochaete chrysosporium* | *RP78* | saprophytic | Basidiomycota: Agaricomycotina | 1 | 2 | / | 4(2) | / | / | / | / | / | 7(2) |
| *Postia placenta* | */* | saprophytic | Basidiomycota: Agaricomycotina | 2 | / | 1 | 23 | / | / | / | / | / | 26 |
| *Puccinia graminis* | */* | phytopathogenic | Basidiomycota: Pucciniomycotina | 3 | 8(2) | 1 | 2 | / | / | / | / | / | 14(2) |
| *Malassezia globosa* | *CBS_7966* | human/animal pathogenic | Basidiomycota: Ustilaginomycotina | 1 | / | 1 | / | / | / | / | / | 1 | 3 |
| *Ustilago maydis* | *521* | phytopathogenic | Basidiomycota:Ustilaginomycotina | 1 | 1 | 1 | / | / | / | / | / | 1 | 4 |
| *Coprinus cinereus* | *okayama7#130* | saprophytic | Basidiomycota: Agaricomycotina | 6 | 2 | 2(1) | 1 | / | / | / | / | / | 11(1) |
| *Sporobolomyces roseus* | */* | saprophytic | Basidiomycota: Pucciniomycotina | 2 | / | 1 | / | / | / | / | / | / | 3 |
| *Rhizopus oryzae* | *RA_99-880.23* | human/animal pathogenic | Mucormycotina:Mucorales | 10(1) | 8(1) | 2(1) | / | / | / | / | / | 1 | 21(3) |
| *Phycomyces blakesleeanus* | */* | saprophytic | Mucormycotina:Mucorales | 10(1) | 9(3) | 2 | / | / | / | / | / | 1 | 22(4) |
| *Batrachochytrium dendrobatidis* | *JAM81* |  | Chytridiomycota: Euchytrids | 1(1) | / | 0(2) | / | / | / | / | / | 1(1) | 2(4) |
| *Encephalitozoon cuniculi* | *GB-M1* |  | Microsporidia; Apansporoblastina | 1 | / | / | / | / | / | / | / | / | 1 |
| *Enterocytozoon bieneusi* | *H348.13* |  | Microsporidia; Apansporoblastina | / | / | 0(1) | / | / | / | / | / | / | 0(1) |
| *Encephalitozoon intestinalis* | *ATCC_50506* |  | Microsporidia; Apansporoblastina | / | / | 0(1) | / | / | / | / | / | / | 0(1) |
|  |  |  |  | 429 | 136 | 84 | 126 | 26 | 33 | 53 | 8 | 9 | 904(89) |

*numbers in brackets represent the deleted gene numbers which show ambiguously aligned regions around the three active catalytic residues.
