# Supplementary material for: Phylogenomic evolutionary surveys of subtilase superfamily genes in fungi
Source: Sci Rep. 2017 Mar 30;7:45456. doi: 10.1038/srep45456 (PMC5371821; doi:10.1038/srep45456)
Supplement: Supplementary Table S4 [file srep45456-s4.docx]

**Phylogenomic evolutionary surveys of subtilase superfamily genes in fungi**

Juan Li*, Fei Gu, Runian Wu, JinKui Yang and Ke-Qin Zhang*

*State Key Laboratory for Conservation and Utilization of Bio-Resources in Yunnan*, *Yunnan University*, *Kunming*, *650091*, *P.R. China.*

* Corresponding author: Juan Li and Ke-Qin Zhang

Tel: 86-871-65033805; Fax: +86-871-65034838.

E-mail address: [juanli@ynu.edu.cn](mailto:juanli@ynu.edu.cn) (Juan Li); kqzhang@ynu.edu.cn(Ke-Qin Zhang)

**Table S4: Best-fit model of protein evolution for each subtilase family sequence.**

| Name | Best-fit Model ^a^ | Paramater |
| --- | --- | --- |
| Proteinase K-like family | GTR+I+G | I ^b^ =0.0043,G ^c^ =1.1777 |
| Pyrolysin family | GTR+I+G | I=0.0109,G=1.4869 |
| Kexin family | TrN+I+G | I=0.0766,G=1.1100 |
| S53 family | TrN+I+G | I=0.0715,G=1.2675 |

^a^, The best-fit model for each family is selected by Modeltest 3.7 [^35^](#_ENREF_35).

^b^, Proportion of invariable sites.

^c^, Gamma distribution shape parameter.
