# Supplementary material for: Phylogenomic evolutionary surveys of subtilase superfamily genes in fungi
Source: Sci Rep. 2017 Mar 30;7:45456. doi: 10.1038/srep45456 (PMC5371821; doi:10.1038/srep45456)
Supplement: Supplementary Data S5 [file srep45456-s5.docx]

**Phylogenomic evolutionary surveys of subtilase superfamily genes in fungi**

Juan Li*, Fei Gu, Runian Wu, JinKui Yang and Ke-Qin Zhang*

*State Key Laboratory for Conservation and Utilization of Bio-Resources in Yunnan*, *Yunnan University*, *Kunming*, *650091*, *P.R. China.*

* Corresponding author: Juan Li and Ke-Qin Zhang

Tel: 86-871-65033805; Fax: +86-871-65034838.

E-mail address: [juanli@ynu.edu.cn](mailto:juanli@ynu.edu.cn) (Juan Li); kqzhang@ynu.edu.cn(Ke-Qin Zhang)

**Supplementary data S5: Amino acid sequences alignment of 146 proteinase K-like sequences.**

　　　For proteinase K-like genes, MUSCLE v3.5 was used to generate protein alignment with default settings [^20^](#_ENREF_20). The ambiguous areas of alignment were located and removed by using the program Gblocks 0.91b [^21^](#_ENREF_21)^,^[^22^](#_ENREF_22) with default parameters. The gap selection criterion “with half” was used here. Finally, an alignment consisting of 184 amino acids from 146 proteinase K-like genes were obtained from Gblocks 0.91b.

>CHGG_10086.t1

YIIRYK--------KDQYNAPWGLARISHYVYSAGTGTVYVVDTGIRTSHDFNGRAIWGH

GTHVAAIIAGSTYGVAKNATVIAVKVLDKTGSGSMSGLLQGVINLSVASVNDAIKIAAAA

GNSPGSAPAITVGAGEFSNWGPGVDIFAPGVSINSAYWLSGTSMATPHVAGLAAYFMARS

TTNR

>FOXT_04749

YIIKYK---VDYIEQDQTSAPWGLARISQYSYDAGAGSVYVLDSGIRTTHEFGGRALWGH

GTHVAGTVGGKTYGVAKGCRMYAVKVIDKNGGGTMSNILQGIINASLGIGNAAVKAAVAA

GNSPASAPAITVAATLWSNYGTVVDIFAPGSDILSAGYKSGTSMAAPHVAGLAAYFMAKD

SPQR

>Pans1184.t1

YIVKYK---IEYIEKDQLHAAWGLARISRYYYDAGQGIVYVLDSGIRTTHEFEGRAVWGH

GTHVAGTIASKTYGVAKKATVVAVKVLDKNGSGTMSGLISGVINISLGSVNAAVKGAVSA

GNSPASAPAITVGAAWFSNWGNLVDIFAPGVSVLSAYYMDGTSMAAPHVAGLAAYFIAKG

SWNR

>FOXT_02695

--------------SEQLDAPWNLARISQYRYDAGSGAVYVVDSGINFSHEFSGRAFRGH

GTHIAGIIGGKTYGVAKSCTMISVKVVSKSGRSNMLWIRQGVINVSVGSVNFAVKNAVAA

GNSPASADAITVAAAPFSNYGRCVDMFAPGAHIASAWYESGTSAAAAHVTGLAAYFISSY

SSNR

>FVET_01532

--------------SDQPNNPWNLERISPCHDDEKPGAVYVIDSGINFLHEFSGRALHGH

GTHMAGIIGGTTYGLAQNCTMISVKVVDKRGKGKQEWLRQAVINISLGTTNHAVKKAVSA

GNSPASASAITVAAAPFSNYSLCVNIFAPGTRVPTVPYSSGTSPAAAQVSGLAAHFISSW

SRNR

>EXU96636.1

---------VASIEVDQQNAPGNLVRLSHYEYDAGQGIVYVLDGGIRLTHEFEGRATFGH

GTHVAAIIGGAKYGVAKQVQIVSVKLQPR-----EPQLEKAIISMSMSDIDKMFRRLVSA

GNSPSRDPVITVAAWEESSYGPAVDLYAPGADITSASTLSGTSQAVPHVAGLAAYIMSLG

-PEY

>XP_007811723.1

---------IATIEANQSDAPGNLVRLSKYDYDGGEGIVYVLDGGIRLTHEFKGRATFGH

GTHVSGIIGGVKFGVAKKVKLVAVKLDPE-----ASQMIQAVISMSMHIVDKKFKHLM--

--SPGRDPVITVAAWSKSNYGPSVTIYAPGVGIESSYYLNGTSQATPHVAGLAAYIMALG

IPDY

>KFG77701.1

YLVAFD---LASIECDQSNAPDNLVRLSNYTYDGGEGIVYVLDGGIHLTHEFGGRATFGH

GTHIAGIIGGAKFGVAKKVKLVAVKLDNE-----ASQMIQAVISMSMHILDQKFKHAVSA

GNSPGRHPIITVAAWPNSNWGSAVTIYAPGVGIESAAYHDGTSQAAPHVAGLAAYIMALG

RFYD

>FGST_12163

FVVALKFKGVLRVE--QQSPPWGLSAISNYTYDAGEGVAYVLDSGIYLEHEFQGRAVFGH

GTLVAAIVNGATYGVAKKATVVDVQVLGDS-TGTTSGVIDGVINMSLSIMNDAVQKAAAA

GNSPGNNPVITVAAWQHSNWGPACDIFAPGEEILSAWTADGTSEAAPHVAGVIAYLLALG

VPNL

>FGST_12292

FIVALKFQGVLLVE--QQSPPWGLSAISNYTYDAGAGTVYVLDSGIYLEHEFQGRAVFGH

GTLVAAIVNGATYGVAKKATVVDVQVLGDD-GGSSSGVLAGVINMSLSVFNEAVQKAAAA

GNSPGNQPVITVAAWAQSNWGSACDIFAPGEQIISAWIASGTSEASPHVAGVVAYLLALG

VPNL

>FOXT_17011

FIVALKFQGVLHVE--QQSPPWGLSAISNYTYDAGAGTVYVLDSGIYLEHEFQGRAVFGH

GTLVAAIVNGATYGVAKKATVVDVQVLGDD-GGSSSSVLAGVINMSLSIFNEAVQKAAAA

GNSPGNQPVITVAAWAWSNWGPACDIFAPGEQITSAWIASGTSEASPHVAGVIAYLFALG

VPNL

>FVET_13831

FIIALKFRGVLHVE--QQSPPWSLSAISNYTYDAGAGTVYVLDSGIFLEHEFQGRAVFGH

GTLVAAIVNGATYGVAKKATVVDVQVLGDD-SGSSSGILAGVINMSLSIFNEAVQKAAAA

GNSPGNQPVITVAAWAWSNWGSACDIFAPGEEITSAWIASGTSEASPHVAGIVAYLLALG

VPNL

>fsol_95099

WIVALKFSAVTAVEKNQENPPWGLSAISHYRYDAGAGTSYVLDSGLLASHEFEGRAALAH

GTHVAGIIGGVTYGVAKKTSIIGVQVTGSS-SGSGAWILDGVINMSLLVVNNAVQAAAAA

GNSPANLPAITVAAWRASNWGSVVDLFAPGESITSAWTTSGTSQAAPHVAGVVAYLLALE

VPNL

>VDAG_05709T0

FVVTLKFAGVSSVE--QKNPPWGLSAISRYVFHAGQGTSYFLDSGIYTDHDFEGRASLGH

GTAIAAVIGGKVHGVAKRTSLIGVKVSGSI-EGSLSWLIDGVINISMVAINSAVQAAVSA

GNSPANLPAITVAAWAASNWGPTADIFAPGEDILTADRHSGTSEAAPYVAGVIAYLLALG

VPNF

>VDBG_06654T0

YVVTLKFAGVSSVE--QENPPWGLSAISRYVFYAGQGTSYFLDSGIYTDHDFEGRASLGH

GTAIAAVIGGNVHGVAKRTSLIGVKASGSH-GGSLSWLIDGVINISMLAINSAVQAVVSA

GNSPANLPAITVAAWAASNWGPTADIFAPGEDILTADRHSGTSEAAPYVAGVIAYLLALG

VPNF

>VDAG_07131T0

SLLSTRLRGVQAVEPLQSNAPWGLARISQYKYDAGAGTAYILDTGIRDTHEFEGRASKGH

GTHVAGTIGGKTYGVAKKAELVGVKVFNDD-PATNADIIRAVVNMSLGALDAAVASTVAA

GNSPAREPAITVGAAIFTSSGKVVDIFAPGVDIKSSWSLDGTSMASPHVAGAVCYLLSQI

DALL

>tree_58698

YVIGLKYRA-------LEKPPWGLATLSNYRYDAGEGTAYVLDTGINSKHDFEGRAYMGH

GTHVAGIIGGKTFGVAKKTQLIGVKVFLDD-EATTSTLMEGVINMSLGALNDAIDHIAAA

GNSPASADAMTVGATNFSNFGPQVNILAPGEDVLSAYVLSGTSMAAPHVAGLALYLMALD

SPNL

>FGST_04745

YIITLKWNAVAFVEPDQSGAPWGLASISRYTYDAGSGSGYVVDSGINVNHDFGGRASLGH

GTHVAGTIASSTYGVAKAANVISVKVFTGN-SASTSTILAGVINMSLGTWTTAINAAVAA

GNSPANVPALTVAAASFTNYGAGVDVFAPGVGILSTWSISGTSMACPHVAGLALYLQVLG

SPNL

>FOXT_09801

YIITLKWSAVAFVEPDQSGAPWGLGTISHYIYDAGQGSAYVVDSGVQVSHNFGGRASLGH

GTHVAGTIAGTTYGVAKRANIISVKVFAGR-EGSTSTILAGVINLSLGTWTSAINAAVAA

GNSPANAPALTVAAASFTNYGAGVDVFGPGVNILSTWTISGTSMACPHVAGLALYLQVLG

SPNS

>FVET_08694

YIITLKWSAVAFVEPDQSGAPWGLGTISHYIYDAGQGSAYVVDSGVQVSHNFGGRASLGH

GTHVAGTIASSTYGVAKQANIISVKVFAGS-SGSTSTILAGVINLSLGTWTSAINAAVAA

GNSPANAPALTVAAASFTNYGAGVDVFGPGVNILSTWTISGTSMACPHVAGLALYLQVLG

SPNS

>fsol_51284

YIITLKWNAVAFVEPDQTGATWGLGTISHYIYDAGSGSAYVVDSGVQVGHQFGTRASLGH

GTHVAGTIAGSTYGVAKQANIISVKVFQGN-QGSTSTILSGVINLSLGTWISAINAAVAA

GNSPANAPALTVGAASFTNYGAGVDIHAPGVSVLSSYSLSGTSMACPHVAGLALYLQVLG

SPNL

>FVET_00027

YIVTLKWNAVAFVEPDQSGAPWGLGTISHYIYDAGEGSAYVVDSGVLISHQFGGRAVAGH

GTHVAGTIAGSTYGVAKKANIVSVKVFQGA-EGTDSGVLAGVINLSLGAWVTAIDAAVAA

GNSPANAAAITVAATSFTNYGAGVDIFAPGQSILSAWSISGTSMASPHVAGLALYLKVLG

SPNL

>tree_121495

FIVTLKWNAVASVEPDQTGAPWGLGTVSHYIYDAGSGTAYVVDSGINIAHQFGGRASLGH

GTHVSGTIGGSTYGVAKQASLISVKVFQGN-SASTSVILDGAINMSLGTWATAINAAVAA

GNSPANVPAITVAAASFTNYGAGVDVFAPGVNILSSWTISGTSMATPHVVGLALYLQALG

SPNT

>fsol_52137

YIVTLKWHGVAFIEPDQQGATWGLGTISHYTYDVGANSAYVVDSGVQVNHEFEGRAIAGH

GTHVAATIAGKTYGVAKKAQIISVKVSQGR-ESSNSASLQGVINLSLGAWTAAIASAVAA

GSSPANAPALTVGATSFTNYGPEVDIMAPGDHIESAGTMSGTSMACPHVAGLALYLQVKG

SANL

>VDAG_05967T0

YIITLKYHGVAAVEKDQSGAPYGLGSLSSYRYDAGQGTAYVVDSGVQVGHQFGGRATLGH

GTHVAGTIGGSTYGVAKRTNIISVKVFVGN-TASTSVILSGVVNLSLGAFNNAVNAAIAA

GNSPASAAAIAVGAASYSNWGAGVTIFAPGSNVLSAWSISGTSMASPHVAGLVVYLQRLG

SPNR

>VDBG_07258T0

YIITLKYHGVAAVEKDQSGAPYGLGSLSSYRYDAGQGTAYVVDSGVQVGHQFGGRATLGH

GTHVAGTIGGSTYGVAKRTNIISVKVFVGN-SASTSVILSGVVNLSLGAFNNAINAAIAA

GNSPASAAAIAVGAASYSNWGAGVTIFAPGSNVLSAWSISGTSMASPHVAGLVVYLQRLG

SPNR

>MGG_07965.t1

YIVTLKFHA--------SSAPWGLASVSSYRYDAGSGVAYIVDSGINTAHDFEGRAVKGH

GSHVAGTIGGKTYGVAKSVNLVDVKVFTGR-SASTSTIISGVINMSLGAFNSAVNNAVAS

GNSPASATAFVVGAASFSNFGAEVDILAPGVNILSSWTISGTSMATPHVVGVAAYLLGLQ

TPNR

>VDAG_07176T0

YIVTLKFHAVAAVEPDQSSAPWGLASISSYRYDAGAGTAYVVDSGILTTHNFGGRAIRGH

GTHVAGTIGSTTYGVAKAATLIDVKVFVGR-TSSTSIILDGVINLSLGAWTSAINAAAAA

GNSPANAPALTVGATSYSNFGPSVDILAPGTNVLSLGTLTGTSMAAPHVAGLALYLAAFG

SPNL

>VDBG_05526T0

YIVTLKFHAVAAVEPDQSSAPWGLASISSYRYDAGAGTAYVVDSGILTTHNFGGRAIRGH

GTHVAGTIGSTTYGVAKAATLIDVKVFVGR-TSSTSIILDGVINLSLGAWTSAINAAAAA

GNSPANAPALTVGATSYSNFGPAIDILAPGTNVLSLGTLTGTSMAAPHVAGLALYLAAFG

SPNL

>MGG_02863.t1

YIVTLKFHAVAAVEPDQNGSTWGLAAVSHYIYDTGADMAYVIDSGININHDFGGRAVRGH

GTHVAGTIGSKTYGVFKDVNLIDVKVLSGS-STTTAVVLEAVINMSLSAYSGAIAAAVAA

GNSPGSAPAITVGAAEYSNYGPSVDVLAPGSHVLSTYSMSGTSSATPHVAGLALYLMARD

TPNL

>EXU94896.1

YIVTLKFHAVLGVEPDQQNPPWALSAMSSYRYDAGENTAYVLDSGVHDKHEFGGRAAPGH

GTMVAGIIASNTYGVAKKANIIAVQTDHTV-SG----TLGGVINYS--AMAQSMDIAIAA

GNSTPNSTALAVGAMRLSNHGPSVDILAPGENVMTISVQSGTSLAAPHVAGLALYLIAAN

TVNL

>KFG85392.1

YIVTLKFHAVLGVEPDQQNPPWALSAMSS-----------LIDTGVHDKHEFGGRVTPGH

GTMVAGIIASNTYGVAKKANIIAVQTDQTV-SG----LLGGVINYS--AMAQSMDIAIAA

GNTTPNSTALVVGGMRLSNHGPSVDILAPGENVITISVQTGTSLAAPHVAGLALYLIAAN

TVNL

>XP_007815016.1

YIITLKFHAVLGVERDQQNPPWALSAISSYQYDAGKDTAYVLDSGVNANHEFGGRATIGH

GTMVAGLIASNTYGVAKKANIIAVQSQNSA-SA----LLDSVINYS--SMARIMETAIAS

GNDAPNSTALVVGAASFSNYGPSVDILAPGANVVTTTTQSGTSLAAPHVAGLALYLITAN

TVNL

>EXV06069.1

YIVTLKFNAVESVEQQQKESTHGLATISHYVYDAGEGSVYVLDSGIQVDHEFEGRAIRGH

GTHVAGIVGSKTYGVAKKTKLVDVKMFHDA-GSTNEIILDGVVNMSFGALNKIIKTAISS

GNSPASSPGITVGAWDHSNHGPVVHILAPGVDVLSLATGSGTSQAAPHVAGLAAYLAVAG

TVNL

>KFG80219.1

YIVTLKFNAVESVEQQQKESTHGLATISHYVYDAGEGSVYVLDSGIQVDHEFEGRAIRGH

GTHVAGIVGSKTYGVAKKTKLVDVKMFHDA-GSTNEIILDGVVNMSFGALNKIIKTAISS

GNSPASSPGITVGAWDHSNHGPVVHILAPGVDVLSLATGSGTSQAAPHVAGLAAYLAVAG

TVNL

>XP_007807911.1

YIITLKFNAVESVEQQQKNSTHGLATVSHYVYDAGEGSVYVLDSGIQLDHEFEGRAIHGH

GTHVAGIVGSKTYGVAKKTKLVDVKLFHDA-GSTSEIILDGVVNMSFGALNKIIKAAIAS

GNSPASSPGITVSAWDHSNYGPVVHILAPGVDVLSLASGSGTSQAAPHVAGLAAYLAVAN

TVNL

>ACB30123.1

YIITLKFNAVADVEQQQKNSTYGLATISHYVYDAGEGSVYVLDSGIDIDHEFQGRAGLGH

GTHVAGTVGSRTYGVAKRAKLVDVKLFHDG-ASTTEVILDGILNMSFGVQNKLVKTAIAA

GNSPASSPGITVAAWQYSNYGSGVHILAPGVNVLSTYEDSGTSMAAPHVAGLAAYLAAAG

TVNL

>FGST_12083

YIITLKFKGVLAVEPVQRNAEWGLASISHYLYDAGNGMAYLVDTGINYGHDFQGRASPGH

GTHCAGTIAGKVYGVAKRANLIAVKVFHSG-SSTTAIVLDGVISMSLGAFNLAVEMAVAA

GNSPASAQATTVGAASFSNFGPFVDIFAPGVSIKSTWTLSGTSMACPHVAGLSLYLRAKG

SPNL

>FOXT_04896

YIVTLKYKGVFAVEPVQQRSTWGLGSISHYIYDAGAGTAYVVDTGINIGHEFQGRAALGH

GTHCAGTIAGKEYGVAKRANLIAVKVFHTG-SSRTDIVLDGVISMSLGAFNAAVQAAVAA

GNSPASAPATTIGAASFSNYGELVDLFAPGVNVKSAWTISGTSMACPHVAGLSLYLRAKG

SPNL

>FVET_03249

YIVTLKYKGVLAVEPVQQKSTWGLGSISHYIYDAGAGTAYVVDTGINIAHEFQGRAALGH

GTHCAGTIAGKEYGVAKRANLIAVKVFHLG-SSRTDIVLDGVISMSLGAFNAAVQAAVAA

GNSPASAPATTVGAASFSNYGELVDLFAPGVNIKSAWTISGTSMACPHVAGLSLYLRAKG

SPNL

>Pans_2598.t1

YIITLRFHGVSSVEQDQKDAPWGLGSISHYIYDAGDGYAYVVDTGIRTTHEFEGRAIFGH

GTHVSGTIAGKTYGVAKKARVVAVKVFDWG-SSTTSIVLDGVINLSLGAVDSAIAAAVAA

GNSPASAPALAVGAPSFSNWGPGVDIWAPGVMVRSAWEVEGTSMASPHVAGLVLYLRSLG

SVNL

>NCU06949.t1

YIVLLKFKAVHSIEPDQKQAPWGLGYLSHYVYNAGTGTAYVVDTGCWKDHEFEGRVQLGH

GTHVTGTLISKTYGVAKNATVICVKVFHGG-GSANTIVMDGVINMSLGAFNAIVDAAVAA

GNSPASSAAFSVGAAYFSNYGAIVDIFAPGVNIVSTYTMSGTSMASPHVAGLALYLKSLM

SPNL

>FGST_00947

YIVTLKFHGVALVEEDQSSATWGLGTVSHYIYDAGSGTAYIVDTGIITSHEFEGRAQAVH

GTHVAGTIAGKTYGVAKKATIQAVKVFQGS-SSSTSIILAGVVNMSLGSFNNAVESAIAA

GNSPASAPAITVGAASYSNYGTVLDIFAPGTSVLSAWTISGTSMATPHIAGLVLYGISVS

SPNL

>FOXT_01145

YIVTLKFHGVAIVEEDQSGATWGLGTVSHYIYDAGTNTAYVVDTGVRTTHEFEGRAQAVH

GTHVSGTIAGKTYGVSKKATIQAVKVFQGS-SSSTSIILAGVVNMSLGSFNNAVDSAIAA

GNSPASAAAITVGAASYSNYGTVLDIFAPGSAVLSAWSISGTSMATPHIAGLVLYGISVS

SPNL

>FVET_00372

YIITLKFQGVAIVEQDQSGAPWGLGTVSHYIYDAGTNTAYVVDTGVRTTHEFEGRAQAVH

GTHVSGTIAGKTYGVSKKATIQAVKVFQGS-SSSTSIILAGVVNMSLGSFNNAVNSAIAA

GNSPASATAITVGAASYSNYGTVLDIFAPGTGVLSAWSISGTSMATPHIAGLVLYGISVS

SPNL

>FGST_09702

YIITLKFRGVAHVEPDQTPSTWGLGTISHYYYHSGSDSAYLVDSGVRTTHEFQGRAKNGH

GTHVAGILAGKTYGVAKKAKIISVKVFQGD-SADLSVIMTGVINLSLGALNDIIKNAVAA

GNSPSSAPAITVGAPSWSNYGSSVDILAPGVDIVSASVEDGTSMACPHVAGLVLYAQSVG

SPNR

>FOXT_03262

YIITLKFHGVVRVEPDQKTSTWGLGTISHYLYHSGSDSAYLVDSGVRTTHEFQGRAKNGH

GTHVAGTIAGKTFGVAKKAKVISVKVFQGD-SADLSVILNGVINLSLGALNDIIKNAVAA

GNSPASAPAITVGAPSWSNYGSSVDMMAPGVNVVSASIESGTSMATPHVSGLVLYAQSVG

SPNR

>FVET_02136

YIITLKFHGVVRIEPDQKSSTWGLGTISHYIYHSGSDSAYLVDSGVRTTHEFQGRAKNGH

GTHVAGTIAGKTFGVAKKAKIISVKVFQGD-SADLSVILNGVINLSLGALNDIIKNAVAA

GNSPASAPAITVGAPSWSNYGSSVDMMAPGVDVVSASIESGTSMATPHVSGLVLYAQSVG

SPNL

>fsol_58928

YIITLKFHAVIAVEPDQQSATWGLGTISHYLYDAGIESAYVVDSGVRATHDFGGRVKQVH

GTHVAGTIAGKTYGVAKKAQVLAVKVFQGE-SAELSVILAGVINLSLGALNKVVENAVAS

GNSPGSVDAITVGAASFSNWGSFVDILAPGVGVTSCSVEDGTSMAAPHVAGLVLYAQSIG

TKNL

>FGST_13031

YIITLKFQGVLAIEPNQKKSTWNLGTISHYIYDAGTDMAYVIDGGVRVSHEFGGRAKAAH

GTHVAGVIAGKTYGVAKKANILALKVFKGE-ESDTSIVLDAVINMSLGAFNRAVDNAVAS

GNSPGSAATITVGAADFSNYGKTVDIMAPGVGILSSGTEDGTSMAAPHVAGLVL------

----

>FOXT_13463

YIITLKFQGVLAVEPNQKKSTWGLGTISHYIYDAGTDMAYVIDGGVRVTHEFGGRAKAAH

GTHVAGTIAGKTYGVAKKANILALKVFNGD-ESDTSIVLDAVINMSLGAFNKAVDTAVAS

GNSPGSASAITVGAADFSNWGKTVDILAPGVGITSAGTEDGTSMAAPHVAGLVLYAMSVG

APNL

>FVET_13954

YIITLKFQGVLAVEPNQKKSTWGLGTISHYIYDAGTDMAYVIDGGVRVTHEFGGRAKAAH

GTHVAGTIAGKTYGVAKKASILSLKVFNGD-ESDTSIVLDAVINMSLGAFNKAVDTAVAS

GNSPGSASAITVGAADFSNWGKTVDILAPGVGITSAGTEDGTSMAAPHVAGLVLYAMSVG

APNL

>EXU96871.1

YIVKLKFHGVAYVEADQESAPWGLAAISHYIYDAGQGTGYVMDTGIRATHEFEGRASTGH

GTHVAGTVGGVTFGVAKKATIIAVKVFHGT-QTSTSIIMGGVINQSLGAWNDAIEAAIAA

GNSPASAPAVTVGAGQGSNYGPVLDIFAPGDGIESAELRSGTSMASPHVAGLALYAMSVG

SPNR

>KFG83000.1

YIVKLKFHGVAYVEADQESAPWGLAAVSHYIYDAGQGTGYVMDTGIRATHEFQGRASTGH

GTHVAGTVGGVTFGVAKKATIIAVKVFHGT-QTSTSIIMGGVINQSLGAWNDAIEAAIAA

GNSPASAPAVTVGAGQGSNYGPVLDIFAPGDGIESAELRSGTSMASPHVAGLALYAMSVG

SPNR

>XP_007814940.1

YIVKLK----AYVEADQESAPWGLAAISHYIHDAGAGTGYVVDSGVRTTHEFEGRASTGH

GTHVAGTVGGKTFGVAKKATIISVKVFQGK-EGSTSIVLAGAINLSLAAWNEAVRTAVAA

GNSPASAPAVTVGAGQGSNYGTVLDIFAPGDNILSAGFDSGTSMAAPHVTGLALYAISVG

SPNR

>ACB30133.1

FIVTLKFMGVEAVEQDQKNATWGLGSVSHYGYQAGKDTAYVIDTGIRTTHEFEGRASHAH

GTHVAGTIGGKTYGVAKNAKLLAVKIFNSR-SSSTSVILAGAINMSLGAFNTAVERAIAA

GNSPASAPAITVAAASYSNFGSVVDICAPGSNITSAWTISGTSMATPHVVGLALYAISVG

SPNL

>EXU95680.1

----HG----------QTSAPWGLRSISHYYYDSGSGTAYILDDGIRETHEFEGRAKNIH

GTAVAGIIGSKTYGVAKKTTLLSVKTLGTTG-ADHSEVLKAVINLSFGALNKFIELLTAA

GNTPGSAKAINVGYAPRSNWGPAVTILAPGVDVETTGLQSGSSYAAPYISGLVLNAISVS

TPNL

>KFG81922.1

----HG----------QTSAPWGLRSISHYYYDSGSGTAYILDDGIRETHEFEGRAKNIH

GTAVAGIIGSKTYGVAKKTTLLSVKTLGTTG-ADHSEVLKAVINLSFGALNKFIELLTAA

GNTPGSAKAINVGYAPRSNWGPAVTILAPGVDVETTGLQSGSSYAAPYISGLVLNAISVG

TPNL

>EXV03193.1

----PE----------QTSAPWGLRAISHYYYDAGAGTAYILDSGIRTTHEFEGRAKAAH

GTGVAGIIGSKTYGVAKKATLISIHLLGPDG-CTGSEAINAVINLSFGALNTFVERLVAA

GNSPGSADVISVGHSETSNFGSAVSILAPGVGVETTGRETGSSFATPYISGLILNAISIR

TPNL

>KFG79137.1

----PE----------QTSAAWGLRAISHYYYDAGAGTAYILDDGIRTTHEFEGRAKFAH

GTGVAGIIGSKTYGVAKKATLISIHLLGPDG-CTGSEAINAVINLSFGALNTFVERLVAA

GNSPGSADVISVGHSETSNFGSAVSILAPGVGVETTGRQTGSSYAAPYISGLILNAISTR

TPNL

>EXU97775.1

----LD----------QKGAPWGLRAISHYYYDAGLDTAYILDSGIRTTHEFEGRAETVH

GTAVAGVLGSKTYGVAKRAKLLSVKTLDDKGSCAASAALHAVINLSFGALDTFIEALTAA

GNTPGSAKVINVGHSPNSNWGPAVTMLAPGVQVECPSLESGSSFAAPHVAGLVLNAISVN

TPNI

>KFG84469.1

----LD----------QKGAPWGLRAISHYYYDAGLDTAYILDSGIRTTHEFEGRAETVH

GTAVAGVLGSKTYGVAKRAKLLSVKTLDDNGSCAASAALHAVINLSFGALDTFIEALTAA

GNTPGSAKVINVGHSPNSNWGPAVTMLAPGVQVECPSLESGSSFAAPHVAGLVLNAISVN

TPNI

>XP_007810847.1

----LD----------QKSAPWGLRAVSHYYYDAGLGTAYILDGGIRTTHEFEGRAETVH

GTGVAGVLGSKTYGVAKRAKLLSVKTLDEEGGCTASAALRAVINLSFGSLNTFIEALTAA

GNTPGSAKVINVGHSPKSNWGPSVTMLAPGVDVECPSLQSGSSFAAPHVAGLVLNAISVN

TPNI

>EXU99955.1

----------------QSKAPWNLQAISHYLYTPKDKTAYVLDTGIRTTHEFEGRAENFH

GTHVAGIIAAKTYGVAKQARVLSVKVFGPNGQVLTSQAILGVINYSGGAWNTIVERATSA

GNSPACADAITVGSAPSSNFGCKVNILAPGGKILSLSTLSGTSMAAPHVAALALNAMAVG

SPNL

>KFG83510.1

----------------QSKAPWNLQAISHYLYTPKDKTAYVLDTGIRTTHEFEGRAENFH

GTHVAGIIAAKTYGVAKQARVLSVKVFGPNGQVLTSQAILGVINYSGGAWNTIVERATSA

GNSPACADAITVGSAPSSNFGCKVNILAPGGKIVSLSTLSGTSMAAPHVAALALNAMAVG

SPNL

>EXV03192.1

----------------QNKAPWNLRAISHYYYDTNGKTAYVVDTGIRTTHEFEGRAENLH

GTHVAGIIASKTYGAAKQARVLSVKVFDDKNDATTSQILAGVINCSLGALKLVYERATSA

GNSGGSASSITVGSASHSNYGSHVTIFAPGADILSLSIMSGTSMAAPHVAAVVLNAMAAG

SPNV

>KFG79136.1

----------------QNKAPWNLRAISHYYYDTNGKTAYVVDTGIRTTHEFEGRAENLH

GTHVAGIIASKTYGAAKQARVLSVRVFDDKNDATTSQILAGVINCSMGALKVAYERATSA

GNSASSASSITVGSAAHSNYGSHVNIFAPGADILSLSIKSGTSMAAPHVAAVVLNAMAVG

SPNV

>XP_007813188.1

----------------QHKAPWNLRTISHYYYQPSDKTAYVVDSGVRISHEFEGRAENLH

GTHVAGTIAAKTYGVAKTARVVSVRVLDKEDRAPTSTIIKGVINMSVGAMNTIIQRAAAS

GNSPASSNALTVGAVKWSNYGRKVDILAPGDGVTSLSTMSGTSMAAPHVAALALNAMAVG

APNL

>XP_007815269.1

----------------QDKAPWNLRAISHYYY-NDETPAYVIDSGIRVSHEFENRAENLH

GTHVAGIIASKTYGVAKKARVVSVKVLNNEGRGDLSQAIAGVINISAGALATAIDRAVA-

GNTPTDSTSITVGAAPFTNLRYKVDIFAPGVDILSLATKSGTSMAAPHVAALALNAMSVG

SPNL

>CHGG_10867.t1

YIVVYN---VSYIEQDQGRTTTGLARISHYVFDAGEGIAFVVDTGIRVTHEFEGRATFAH

GSHVAGTIGGKTFGVAKKVNLVAVKVLGADGSGSNSGVLAGVMNMSLGAINAAINNIVAA

GNSPGSAEAITVGAAEFSNFGRLVDVFAPGVQVLSVGALSGTSMASPHVAGLAAYLMALG

TTTL

>NCU06055.t1

YIVVYN---VEYIEADQNSTTTGLARLSHYIFDAGEGIAFVVDTGIRVTHEYEGRATFAH

GSHVAGTIAGATFGVAKKAKLVAVKVLDGSGSGSNSGVLQGVLNMSLGAINSAINQIVAA

GNSPGSAPAITVGAASFSNFGAGVDIFAPGVNVLSVGTLSGTSMASPHVAGLAAYLMALG

TTSL

>Pans_2378.t1

FIVVYN---VSYIEQDQSRAPTGLARLSHYFFDAGEGIAYVVDTGIRTTHDLQGRARFGH

GTHVAGTIGGWKFGVAKKTQLVAVKVLGADGSGSNSGVIAGVMNMSLGAVNTAINRIVAA

GNSPGSAEAITVGAASFSNFGPLVDIFAPGVRVESCGTLSGTSMASPHVAGLAAYIMALG

TTDL

>FGST_10778

YIVVYN---VAYIEADQVNAPPGLNRLSHYIFDAGEGIAYVVDTGIKVDHEFEGRATFGH

GSHVAGTIGGATFGVAKKVDLVAVKVLDASGGGSNSGVLQGVMNMSLGAINRAIEALVAA

GNSPGSAPAITVGAADFSNFGPEVDIYAPGVDVLSVGTLSGTSMASPHVAGLAAYLMGFG

TTDG

>FOXT_06062

YIVVYN---VAYIEADQVNAPPGLDRLSHYVFDAGEGIAYVVDTGIKIDHEFEGRATFGH

GSHVAGTIGGATFGVAKKVDLVAVKVLDASGGGSNSGVLQGVMNMSLGAINRAIEALVAA

GNSPGSAPAITVGAADFSNFGPEVDVYAPGVNVLSVGTLSGTSMASPHVAGLAAYLMGFG

TTDS

>FVET_03935

YIVVYN---VAYIEADQVNAPPGLDRLSHYVFDAGEGIAYVVDTGIKVDHEFEGRATFGH

GSHVAGTIGGATFGVAKKVDLVAVKVLDASGGGSNSGVLQGVMNMSLGAINRAIEALVAA

GNSPGSAPAITVGAADFSNFGPEVDVYAPGVNVLSVGTLSGTSMASPHVAGLAAYLMGFG

TTSN

>tree_64719

YIVVYN---VAYIEADQTNAAPGLIRLSNYIFDAGSNIAYVVDTGIRITHEFEGRATFGH

GSHVAGTIGGATFGVAKNVELVAVKVLDADGSGSNSGVLNGVMNMSLGAVNNAITALVAA

GNSPGSAPAITVGAAGFSNFGTGVDIYAPGVDVLSVGVLSGTSMASPHVAGLAAYLMALG

TTSL

>ACN30270.1

YIVVYN---VAYVEADQINAPLGLQRLSEYLFDAGEGIVYVVDTGVRITHEFQGRARFGH

GSHVAGTIAGGTFGVAKKARITAVKVLDAKGSGANSGILGGIMNMSLGAMNHAIENVVAA

GNSPASAPAITVGAAAFSNFGPDVDIFAPGVDVLSVGTLSGTSMASPHIAGLTAYLMHIE

TTRV

>MAPG_11267T0

FIVVYN---VAYVEGDQDGAPNGLARLSSYAFDAGQNIVYVVDTGIRETHEFEGRATFGH

GSHCAGTIGGKTFGVAKKANIIGVKVLDANGGGSNAGVIRGVMNMSLGAVNDAINNIAAA

GNSPASAPAITVGAASFSNFGTVVDIFAPGVNVLSVDTLSGTSMAAPHIAGLAAYLMALG

TTTL

>MGG_09246.t1

YIVVYN---VRYVEADETGAPNGLARLSQYRFEAGEGIVYIVDTGIRATHEFEDRATFGH

GSHCAGTIGGKTFGVAKKATLVGVKVLGASGGGSNRGVIQGVMNMSLGAVNEAINSMVAA

GNSPASAPAITVGAASFSNFGTDVDIFAPGVNVLSVDTLSGTSMASPHVAGLAAYLMALG

TTTL

>VDAG_06012T0

YIVVYN---VAWVEANQMNAPPGLNRLSNYVFDGGEGVVYVVDTGIRTDHEFQGRATFGH

GSHVAGTICGQTFGVAKSANVVAVKVLDGTGAGSNAGVLDGVMNMSLGAVNRAVQALVAA

GNSPASAPAITVGAASFSNFGADVDIFAPGVDVLSVGTLSGTSMASPHVAGLAAYLIALG

TTNL

>EXU95412.1

YIVVYK---VDYVEKNQENAPAGLQRLSEYVYDAGNGTAYVVDSGCRTTHDFEGRATTIH

GTHVACTIAGAKFGVAKLATVKCVKVMNAEGQGTNADIIAGTMNMSLGALDTAINNVVAA

GNSPAAAPAITVGAAGFSNFGPSVDINAPGVDVQSCGTKSGTSMASPHVAGLANYLMRLD

TTPL

>KFG84169.1

YIVVYK---VDYVENNQRNAPVGLQRLSE------------VDSGCRTTHDFEGRATTIH

GTHVACTIAGRKFGVAKKATVKCVKVMNAKGQGTNADIIAGTMNMSLGALDTAINNV---

--SPAAAPAVTVGAAGFSNFGPSVDINAPGVDVQSCGTKSGTSMASPHVCGLANYLMRLD

TTPL

>007815324.1

YIAVYK---VDYVEPDQQNAPPGLQRLSEYVFDAGNTTAYVVDSGCLTTHDFEGRATTIH

GTHVACTIGGKNFGVAKKATVKCVKVMNANGQGQNADIIAGVVNLSLGALDAAMNNVVAA

GNSPASARAITVGAASFSNFGRDVDINAPGVKVQSCGVKSGTSMASPHVAGLAAYLMTLG

TTPL

>CHGG_06380.t1

YIVKFKFLGVEYIERDEKSAPWGLARISHYLYAGGEGVAYVIDTGTNIEHDFEGRANWGH

GTHCSGTIAGKKYGVAKKAKVYAVKVLRSNGSGSMSDVVAGVANMSLGTLDDTVNAAVAA

GNSPAAAAAITVGAAYFSNYGKCTDIFAPGLSILSTWTISGTSMASPHIAGLLAYYLSLD

TPNL

>Pans_2023.t1

YIVKFKFLGVEYIERDEKAAPWGLARISHYLYAGGEGVAYVIDTGTNVDHDFDGRAKWGH

GTHCSGTIAGKKYGVAKKANVYAVKVLRSNGSGTMSDVVAGVANMSLGTLDDTVNAAVAA

GNSPAAAAAVTVGAAYFSNYGKCTDIFAPGLSILSTWTISGTSMASPHIAGLLAYYLSLG

TPNL

>MAPG_11020T0

YIIKFKFLGVEAIERDEKSAPWGLARISHYLYSGGEGVAYVIDTGTNTEHDFEGRAHWGH

GTHCSGTVAGKKYGVAKKANVYAVKVLKSNGSGTMSDVIAGVANMSLGLLDQAVNAAVAA

GNSPAAAAAVTVGAAYFSNWGKCTDIFAPGLNIQSTWTISGTSMASPHIAGLLAYYLSLD

TPNL

>MGG_03670.t1

YIIKFKFLGVEAIERDEKGAPWGLSRVSHYLYSGGEGVAYVIDTGTNIDHDFEGRAHWGH

GTHCSGTVAGKKYGVAKKAQVYAVKVLKSNGSGTMSDVIAGVANMSLGLLDAAVNAAVAA

GNSPAAAAAVTVGAAYFSNWGKCTDIFAPGLNIQSTWTISGTSMASPHIAGLLAYYLSLD

TPNV

>VDAG_02670T0

YIVKFKFLGVEFVEKDEKSAPWGLARVSHYLFAAGEGVAYIIDTGTNVDHDFEGRAKWGH

GTHCSGTVAGSKYGVAKKAHVYAVKVLRSNGSGTMSDVVKGVANMSLGALDRVVDAAVAA

GNSPAAAAAVTVGASYFSNYGKCTDIFAPGTNILSTWTISGTSMASPHICGLLAYYLSLD

TPNI

>VDBG_02202T0

YIVKFKFLGVEFVEKDEKSAPWGLARVSHYLFAAGEGVAYIIDTGTNVDHDFEGRAKWGH

GTHCSGTVAGSKYGVAKKAHVYAVKVLRSNGSGTMSDVVKGVANMSLGALDRVVDAAVAA

GNSPAAAAAVTVGASYFSNYGKCTDIFAPGTNILSTWTISGTSMASPHICGLLAYYLSLD

TPNI

>FGST_00238

YIIKFKFKGVEFIERDERSAPWGLARISHYLYSGGEGVAYIVDTGTNIKHDFEGRAHWGH

GTHCSGTVAGKKYGVAKKASVYAVKVLRSNGSGSMSDVVKGVANMSLGALDAAVNAAVAA

GNSPAAASPVTVGAAYFSNYGKCTDIFAPGLNIVSTWTISGTSMASPHIAGLLAYYLSLD

TPNL

>FOXT_01284

YIIKFKFKGVEFIERDERQAPWGLARISHYLYSGGEGVAYIVDTGTNVDHDFEGRAHWGH

GTHCSGTVAGKKYGVAKKANVYAVKVLRSNGSGSMSDVVKGVANMSLGALDAAVNAAVAA

GNSPAAASPVTVGAAYFSNYGKCTDIFAPGLNIQSTWTISGTSMASPHIAGLLAYYLSLD

TPNL

>FVET_00212

YIIKFKFKGVEFIERDERQAPWGLARISHYLYSGGEGVAYIVDTGTNVDHDFEGRAHWGH

GTHCSGTVAGKKYGVAKKANVYAVKVLRSNGSGSMSDVVKGVANMSLGALDAAVNAAVAA

GNSPAAASPVTVGAAYFSNYGKCTDIFAPGLNIMSTWTISGTSMASPHIAGLLAYYLSLD

TPNL

>fsol_61473

YIIKFKFKGIEFIERDETSAPWGLARISHYLYSGGEGVAYIVDTGTNIDHDFEGRAHWGH

GTHCSGTVAGKRYGVAKKANVYAVKVLRSNGSGSMSDVVKGVANMSLGALDAAVNAAVAA

GNSPAAAAPVTVGAAYFSNYGKCTDIFAPGLNIQSTWTISGTSMASPHIAGLLAYYLSLD

TPNL

>tree_123244

YIIKFKFKGVEYIERDEKQAPWGLARISHYLYTGGEGVAYVIDTGTNIEHDFEGRAKWGH

GTHCSGTVAGKKYGVAKKAHVYAVKVLRSNGSGTMSDVVKGVANMSLGALDAAVNAAVAA

GNSPAAATPLTVGAAYFSNYGKCTDIFAPGLSIQSTWTISGTSMASPHICGLLAYYLSLD

TPNL

>EXU97046.1

YIIKFKFKGVEFIEKDEKQAPWGLARVSHYLYAGGEGVAYVIDTGTNTGHDFEGRAKWGH

GTHCSGTIAGKKYGVAKKANVYAVKVLRSNGSGTMADVVKGVANMSLGALDAAVNAAVAA

GNSPAAAEPVTVGAAYFSNYGKCTDIFAPGLNILSTWTISGTSMASPHICGLLAYYLSLD

TPNK

>KFG81188.1

YIIKFKFKGVEFIEKDEKQAPWGLARVSHYLYAGGEGVAYVIDTGTNTDHDFEGRAKWGH

GTHCSGTIAGKKYGVAKKANVYAVKVLRSNGSGTMADVVKGVANMSLGALDAAVNAAVAA

GNSPAAAEPVTVGAAYFSNYGKCTDIFAPGLNILSTWTISGTSMASPHICGLLAYYLSLD

TPNK

>XP_007810725.1

YIIKFKFKGVEFVEKDERQAPWGLARVSHYLYAGGEGVAYVIDTGTNTEHDFEGRAKWGH

GTHCSGTIAGKKYGVAKKANVYAVKVLRSNGSGTMADVVKGVANMSLGALDAAVNAAVAA

GNSPAAAEPVTVGAAYFSNYGKCTDIFAPGLNILSTWTISGTSMASPHICGLLAYYLSLD

TPNK

>ACB30128.1

YIIKFKFKGVEFIEKDEKQAPWGLARLSHYLYAGGEGVAYVIDTGTNVDHDFEGRAKWGH

GTHCSGTIAGKKYGVAKKAHVYAVKVLRSNGSGSMSDVVKGVANMSLGALDAAVNAAVAA

GNSPAAAEPVTVGAAYFSNYGKCTDIFAPGLNILSTWTISGTSMASPHICGLLAYYLSLD

TPNK

>SJAG_02432T0

YIVLLQFQGVAIVERDQSNAPWGLARISHYEYDAGEGVVYIIDTGINTNHEFEGRAVWGH

GTHVAGTVASRAYGVAKKAKVIAVKVLRSNGSGTMSDVISGIANMSLGILDFAVDVAVAA

GNSPAASNAITVGAAYFSNYGDCVDVFAPGLNILSTWIISGTSMATPHIAGLAAYLVGLD

TPNL

>SOCG_00777T0

YIIILDFKGVIAVERDQSGAPWGLSRVSHYVYDAGEGVAYVVDTGVNVHHEFEGRAKWGH

GTHVAGTIASRAYGVAKKAKIIAVKVLRSNGSGSMSDVISGVANMSLGVLDFAVDSAVAA

GNSPAASKAITVAAAYFSNYGKCVDIIAPGVNILSTWTISGTSMATPHVAGLAAYYLGLD

TPNL

>SPOG_01720T0

YIIILDFKGVIAVERDQSGAPWGLSRVSHYVYDAGEGVAYVVDTGVNIHHEFEGRAKWGH

GTHVAGTIASRAYGVAKKAKIVAVKVLRSNGSGSMSDVISGVANMSLGVLDFAVDSAVAA

GNSPAASKAITVAAAYFSNYGKCVDIIAPGVNILSTWTISGTSMATPHVAGLAAYYLGLD

TPNL

>SpomC4A8.04.t1

YIIVLQFKGVIAVERDQSGAPWGLARISHYVYDAGDNIAYVVDTGVSIHHEFEGRASWGH

GTHVAGTIASRAYGVAKKAEIVAVKVLRSSGSGTMADVIAGVGNMSLGVLDMAVDSAVAA

GNSPAASKAITVGAAYFSNYGSCVDIFAPGLNILSTWTISGTSMATPHVAGLSAYYLGLS

TINL

>SJAG_04785T0

YIIVFKFRGVLLVEPDRTLAPWGLARISHYQYDAGEGVAYVVDTGINTEHDFGGRAVWGH

GTHVAGTIAGNTFGVSNKAKVVAVKVLNAEGSGLVSDIIKGVVNMSLGALDTAVSAALAA

GNSPARVSAMTVGAASFSNIGSCVDVFAPGHYVLSDWLLSGTSMASPHIAGLAAYFTALD

TPNL

>SOCG_01007T0

YIVMFKFMGIAIVEPDQRFAPWGLARVSHYNYNAGEGVAYVIDTGVNVNHDFEGRASWGH

GTHVAGTIAGKTFGVAKNSKIVAVKVMRADGTGTTSDIIKGVANMSLGALDLAVSAAVAA

GNSPSRVSAMTVGASSFSNHGSCVDIFAPGSLILSDWLLSGTSMASPHVAGLAAYYISLG

TPNV

>SPOG_01018T0

YIVMFKFMGIALVEPDQRFAPWGLARISHYNYNAGEGVAYVIDTGINVDHDFGGRATWGH

GTHVAGTIAGKNFGISKNAKLVAVKVMRADGTGSTSDIIKGVANMSLGALDSAVSAAVAA

GNSPSRVSAMTVGSSSFSNHGSCVDIFAPGSLILSDWLLSGTSMASPHVAGLAAYFISLG

TPNV

>SpomC1006.01.t1

YIVMFKFLGIALVEPDQTFAPWGLARVSHYQYNAGEGVAYVIDTGINIEHDFQGRATWGH

GTHVAGTIAGKTFGVSKNAKLVAVKVMRADGTGTVSDIIKGVVNMSIGALDLAVNAAVAA

GNSPARVSAMTVGAASFSNIGSCVDIFAPGSLILSDWILSGTSMASPHVAGLAAYFISLD

TPNV

>CHGG_06857.t1

YLIKLRFRGVEYVEEDQSGAPWGISRLSHYVYDAGEGTSYVIDTGIYTAHDFGGRATFAH

GTHVAGTIGSTTYGVAKKTKLYAVKVLGSDGSGSTSGVVAGVANMSLGSINQAAAALVAA

GNSPASEAVCTVGAASYSNYGSVVDIQAPGTNILSTWTISGTSMASPHIAGLAAYLLALG

TTNR

>NCU07159.t1

YIVKLRFRGVEYVEQEQSSVPWGLARLSSYVYDAGAGTAYIIDTGINTAHDFGGRATWLH

GTHVAGTVGGTTYGVAKKTQLYAVKVLDSNGSGSNSGVIAGVANMSLGSTNSAAAAMVAA

GNSPASEPVCTVGAAYYSNYGTIVDIFAPGTSITSAWTISGTSMATPHITGLGAYLLTLG

TVNK

>Pans_199.t1

YIVKLKFKGVEYVEEEQTGAPWGLARISSYRYDAGAGTSYIIDTGIYTAHDFQGRAIWGH

GTHVAGTVGGRLYGVAKKTTLIAVKVLNASGSGSTSGVVAGVANMSLGSINTAARNLVAA

GNSPASEAVCTVGASTFSNYGAVVDIFAPGTNILSTWSISGTSMASPHIAGLGAYLLTLG

TINK

>MGG_08966.t1

YIVVLKFNGVEFIEEDQTGAPWGLARISSYRYDAGVGTSYIIDTGLYAAHDFGGRASQVH

GTHVAGTIGGTKYGVAKRTTLLGVKVLNASGSGTNSGVISGVVNMSLGAVNNAARAIVAA

GNSPASEPACTVGAASYSNYGALVDVFAPGSGILSAWTIDGTSMASPHIAGLGAYLLALG

TVNA

>MAPG_06159T0

YIVMLKLNGVDYIEQNQSGATWGLARLSSYTYDAGKGVAYILDTGIRVTHEFGGRAIWAH

GTHVAGTVAGTTYGVAKAATLYAVKVLNNSGGGTTASVIAGVVNMSLGSLNAAARAVVAA

GNSPANEPVCTVGSSSFSNYGPVVDVFAPGTDVTSAWTISGTSMATPHITGLGAYLLGLG

TVNL

>MGG_10449.t1

YIVVLKFRGVDYIEEDQTGADWGLARLSSYRYDAGTGTAYVIDTGIMIDHEFEGRATWAH

GTHVAGTIGGVTYGVAKQTRLFAVKVLDSSGSGTNSQIIAGVVNMSLGAVNSAARAVVAA

GNSPSSEEVCAVGAASYSNFGAGVALFAPGSDIKSAWSISGTSMATPHVAGLGAYLMGIG

TANR

>Pans_667.t1

YIVKLRSRGVEFVEQDQEDVPWGLARISHYVYDAGEGTSYIIDTGIYVNHQFTNRAHWLH

GTHVAGTIGGITYGVSKKTSLYAVKVLRASGSGTLAAVIAGTANLSLGAVNAAAAAAVAA

GNSPASEEVCTVGAAWFSNYGEGVDVFAPGVGVESAWTISGTSMAAPHVAGLASYLLALG

TFNG

>FGST_05139

YIVKMKFSGVEYIEQDQDNAPWGIARLSSYTYDAGEGTSYVIDTGIDVEHDFDGRAKFLH

GTHVAGTIGSTTYGVAKKTTLYAVKVLGDDGSGTNSAVIAGVVNMSLGAVNSAAKSIVAA

GNSPASEAACTVGASYFSNFGDLVDVLAPGTDILSTWTISGTSMASPHVAGLGAYFLGLD

TVNK

>FOXT_12263

YIVKMKFGGVEYIEQDQSNAPWGIARVSSYTYDAGEGTAYVIDTGIDVDHDFDGRAKFLH

GTHVAGTIGSTTYGVAKKTSLFAVKVLGDDGSGTNSAVIAGVVNMSLGAVNQAAKAIVAA

GNSPASEEACTVGAADYSNFGSVVDVLAPGTDILSTWTISGTSMASPHVAGLAAYFLGLG

TVNK

>FVET_10880

YIVKMKFGGVEYIEQDQSNAPWGIARISTYTYDAGEGTAYVIDTGIDVDHDFDGRAKFLH

GTHVAGTIGSTTYGVAKKTSLFAVKVLGDDGSGTNSAVIAGVVNMSLGAVNQAAKAIVAA

GNSPASEEACTVGAADYSNFGSVVDVLAPGTDILSTWTISGTSMASPHVAGLAAYFLGLG

TVNK

>fsol_86938

YIVKMKFNGVEYIEQDQDNAPWGLARISSYTYDAGEGTAYVVDTGIDVDHDFDGRAKFLH

GTHVAGTIGSTTYGVAKKTSLFAVKVLDDNGEGTNSGVIAGVVNMSLGAVNNAAKSIVAA

GNSPASEPACTVGAAYYSNTGKIVDVLAPGTDILSTWTISGTSMASPHVAGLGAYLLGLG

TPNK

>EXV01910.1

YIIKMKWNGVDFIEQDQQNADWGLARLSSYTYDAGEGTAFIIDTGIEAGHDFEGRAEFLH

GTHVAGTIGSKTYGVAKKTKLFGVKVLDAQGSGSNSFVIAGVVNMSLGAVNQAAQAIVAA

GNSPASESACTVGAATYSNTGSGVDVLAPGSDIESTWTISGTSMASPHVAGIGAYFLGKG

TANV

>KFG84128.1

YIIKMKWNGVDYIEQDQQNADWGLARLSSYTYDAGEGTAFIIDTGIEAGHDFEGRAEFLH

GTHVAGTIGSKTYGVAKKTKLYGVKVLDAQGSGSNSAVIAGVVNMSLGAVNDAARKIVAA

GNSPASEAACTVGAATYSNTGAGVDVLAPGSDIASTWTISGTSMASPHVAGIGAYFLGKG

TVNV

>XP_007811482.1

YIVKMKFNGVDYIEQDQQNADWGLARLSSYTYDAGEGTAFIIDTGVEASHEFEGRATFLH

GTHVAGTIGSKTYGVAKKTKLFGVKVLDAQGSGSNSAVIAGVVNMSLGAVNQAAQAIVAA

GNSPASEQACTVGAAQYSNHGSIVDVLAPGSDIASTWTISGTSMASPHVAGIGAYFLGKT

TPNV

>ACB30132.1

YIIKMKFNGVDFIEQDQSGADWGLARLSSYIYDAGEGTAFIIDTGVEADHEFEGRAKLLH

GTHVSGTIGSKTYGVAKKTQIYGVKVLDAQGSGSNSAVIAGVANMSLGAVNEAAAGIVAA

GNSPASAEACTVGAATYSNIGKLVDVLAPGSNISSTWTISGTSMASPHVAGIGAYFLGKE

TVNV

>FGST_10218

YIVMMKLGGVAYIEQDQKQPPWGLARVSSYTYDAGKGTAYVLDTGIDTTHDFEGRAEFIH

GTHCAGTIGSKTYGVAKKTKLYGIKLFNSTGEGVASSIIAGVVSMSFGGINDAAKALAAA

GNSPASEPICTIGAATFSNYGKVVDLYAPGVAVLSTWSISGTSMATPHVAGVAAYFLGLG

TKNL

>FOXT_05860

----MH----------QKNAPWGLARISKYTYDAGEGTTYVLDTGIEVDHEFEGRARFVH

GTHIAGTIGSKTYGVAKKTQLFAVKVLNEYTAGQTSGILAGVVNMSVSAINAAARYIVAA

GNSPSNEPACTVGAASFSNYGVSVDVFAPGVDIKSTWLESGTSMATPHVTGLAAYLLGLN

TVNL

>EXU96964.1

YIVKFKFKGVEFIEQDQKNAPWNLARISHYIYDAGEGTSYIIDTGIDATHQFGGRAQNIH

GTHLAGVIGSVIYGVAKKTKLYGVKCLDDQGSGTTSNVIAAMANMSLGAVNKAAASLVAA

GGSPASEPVCTVGAASYSNYGPVVDIFAPGVSILSTWTLSGSSMSAAHITGLGAYIAALG

TLNL

>KFG82971.1

YIVKFKFKGVEFIEQDQKNAPWNLARISHYVYDAGEGTSYIIDTGIDATHQFGGRAQNIH

GTHLAGVIGSAIYGVAKKTKLYGVKCLDDQGSGTTSNVIAAMANMSLGAVNKAAASLVAA

GGSPASEPVCTVGAASYSNYGPVVDIFAPGVSILSTWTLSGSSMSAAHITGLGAYIAALG

TLNL

>XP_007815345.1

YIIKLKFKGVEFIEQDQKTAPWNLARISRYVYDAGEGTSYIIDTGIDASHQFGGRAQNIH

GTHLAGIVGSVIYGVAKKTKLYGVKCLDDQGSGTISNVIAAMANMSLGAVNKAAASLVAG

GGSPASEPVCTVGAATYSNYGAVVDIFAPGGSILSTWTISGSSMSAAHITGLGAYIAALG

TPNL

>EXU97185.1

YIVKYKFNGVDFIENDQPGAPWGLSRISHYAYDAGEGTAYVIDTGVEASHEFEGRAEFIH

GTHVAGTIGSKKYGVAKKTKILGIKVLSDQGSGDYSGILAGVANMSLGAINQAAAKMVAA

GNSPASEPVCTVGASSFSNYGAAVDILAPGSDILSTWSISGTSMATPHIVGLGAYLSSLG

TVNL

>KFG81480.1

YIVKYKFNGVDFIENDQPGAPWGLSRISHYAYDAGAGTAYVIDTGVEASHQFEGRAEFVH

GTHVAGTIGSKEFGVAKKTKILGIKVLSDQGSGDYSGILAGVANMSLGAINQAAAKMVAA

GNSPASEPVCTVGASSFSNYGAVVDILAPGSNILSTWSISGTSMATPHIVGLGAYLASLS

TVNL

>XP_007812076.1

YIVKYRFNGVEFIENDEPGAPWGLSRISHYAYDAGEGTAYVIDTGVDGSHDFEGRAQLIH

GTHVSGTIGSRSYGVAKKTTIYGIKVLSNQGSGDYSGILAGVANMSLGAINQAAAQMVAA

GNSPASEPVCTVGASSFSNYGAPLDILAPGSDILSTWSISGTSMATPHVVGLAAYLASLG

TVNL

>ABK27194.1

YIVKYKFHGVDYIEKDQRDAPRGLRRVSHYVYHAGEGTSYIIDTGVDDSHEFEGRAQLVH

GTHVAGTIGSRSYGIAKKTQLLGIKVLSDQGSGNNSAIIAGLANMSLGSLNDAAAQMVAA

GNSPASEPVCTVGSSSFSNYGSVVDILAPGSDILSTWILSGTSMATPHIVGLAAYLAGLG

TLNL

>EXV04324.1

YIVKFKFHGVDFIEKDQSGAPWGLGRISHYRYDAGQGTVYIIDTGIEASHEFEGRATFLH

GTHCAGTIGSKTYGVAKKAKLYGVKVLDNQGSGSYSGIISGIASMSLGSVNQGAAALVAA

GNSPASEPACTVGASSFSNYGRVVDIFAPGSNVLSTWTISGTSMATPHIAGLAAYLSALG

TVNY

>KFG86683.1

YIVKFKFHGVDFIEKDQSGAPWGLGRISHYRYDAGEGTVYIIDTGIEASHEFEGRATFLH

GTHCAGTIGSKSYGVAKKAKLYGVKVLDNQGSGSYSGIISGIASMSLGSVNQGAAALVAA

GNSPASEPACTVGASSFSNFGRVVDIFAPGTGVLSTWTISGTSMATPHIAGLAAYLSALG

TVNY

>XP_007807399.1

YIVKFK---VDFIEKDQNGAPWGLGRISHYRYDAGEGTVYIIDTGVEVSHEFGGRATWLH

GTHCAGTIGSRSYGVAKNAKLFAVKVLDDQGSGSYSGIISGIASMSLGSVNQGAAALVAA

GNSPASEPACTVGASTFSNYGRVVDIFAPGTGILSTWTISGTSMATPHIAGLAAYFSALG

TVNF

>ACB30118.1

YIVKFKFRGVDYIEKDQPGSPWGLGRISHYRYDAGAGTAYIIDTGIEASHEFEGRATFLH

GTHCSGTIGSKSYGVAKKTKLYGVKVLNNSGSGAYSAIIAGIASMSLGSVNRAAAALVAA

GNSPASEAACTVGASSFSNYGRVVDIFAPGTNILSTWSISGTSMATPHVAGLAAYLSALG

TVNY

>tree_123234

YIVKFKFRGVDYIEQDQTGAPWGLGRISHYVYDAGAGTSYVIDTGVDATHDFEGRATLLH

GTHVSGTIGSRTYGVAKKTQIYGVKVLDNSGSGSFSTVIAGVANMSLGSVNQAAARLVAA

GNSPASEPVCTVGAASFSNYGSVVDIFAPGQDILSTWTISGTSMATPHIVGLGAYLAGLG

TINA

>EXU97160.1

YTVKFKFVGVEYVEQDQPKASWNLGRIANYVYDAGEGTAYVIDTGVDDTHDFGGRAKQIH

GTHVAGILGSTTYGVAKQTRIFGVKVLDNNNQGYESRIIQGVVNLSTGIFNAAAAALAAA

GNSPGSDPVCVVGGPFFTNFGARVDIFAPGQDIVSTRTMSGTSQACPHVVGIAAYLASLN

TPNR

>KFG78683.1

YTVKFKFVGVEYVEQDQPKASWNLGRIAHYVYDAGEGTAYVIDTGVDDTHDFGGRAKQIH

GTHVAGILGSTTYGVAKQTRIFGVKVLDNNNQGYESRIIQGVVNLSTGIFNAAAAALAAA

GNSPGSDPVCVVGGPFFTNFGARVDIFAPGQDIVSTRTMSGTSQACPHVVGIAAYLASLN

TPNR

>EXU98483.1

YTVILKFKGVDFVEMDGP-VPSHPDQVFRYLNNGGEGVAYVVDSGVDVTHEFGGRAHMVH

GTHVAGILGSNSYGVAKRVTIYGIKALSERPDSGISNMIAGVVNLSAGALNMAARGLVAA

GNSPASEPICTVG-YRDSNFGPAVDIQAPAVNVLSTVRLTGTSMASPYIAGLAASIASA-

----

>KFG79696.1

------FKGVDFVEMDGP-VPSHPDQGFRYLNNGGEGVAYVVDSGVDVTHEFGGRAHMVH

GTHVAGILGSNSYGVAKRATIYGIKALSEQPDSGIWTLIAGVVNLSAGALNRAARGLVAA

GNSPASEPICTVG-YRDSTFGPAVDIQAPAVNVLSTVRLTGTSMASPYIAGLAASIASA-

----

>ACN30265.1

YIVKMKFRGVDYIEPDQT-APWGLTRISHYVYDGGKGVAYVIDTGVDARHEFEGRAHQLH

GTHVAGTIGSRTYGVAKRVTIFGVKVLAANNKSN-SVIIKGVVNMSIGAENQAAARLVAA

GNSPASEPVCTVGGYTMSNWGPALDINGPGVDVLSTLRKTGTSMATPHIAGLGAYLAALG

TVNL

>EXU98627.1

YIVKLKFKGVDYVEQNQQ-APWGLARLSHYIYDAGEGTAYVVDSG----NEFEGRAHFLH

GTHVAGTIGGRQVGVAKKTTIYGIKVLDMNREADTSVIIAGVVNLSLGAMNEAAAALVAA

GNSPASEPVCTVGSARDSNYGDVVDVQAPGVEVVSARTMSGTSMAAPHVAGLGAYLLGLG

TRNL

>KFG79277.1

YIVKLKFKGVDYVEQNQQ-APWGLARLSHYIYDAGEGTAYVVDSGLYAAHEFEGRAHFLH

GTHVAGTIGGRQVGVAKKTTIYGIKVLDMNREADTSVIIAGVVNLSLGAMNEAAAALVAA

GNSPASEPVCTVGSARDSNYGDVVDVQAPGVEVVSARTMSGTSMAAPHVAGLGAYLLGLG

TRNL

>XP_007807117.1

YIVKLKFKG------NQQ-APWNPARLSHYIFDAGEGTAYVVDSGLYAAH-FEGRAQFLH

GTHVAGTIGGRQVGVAKKTAIYGIKVLDLNREADTSVIVAG-------ASSSTSA-----

--SPSTRPTTTPWTARDSNYGGVVDIQAPGVDVVSARSMSGTSMATPHVAGLAAYLLGL-

----

>EXV02470.1

YVVYLRRRGVDFIEQVQKSATWNLGRISNYVYDAGEGTTYVIDTGVDDTHEFEGRALQIH

GTHVAGIIGSASYGVAKKTKIFGIKVLDSNGNAEGDRLIAGVVNYSINSINVAAAELVAA

GNSPASEAVCTVGSAVDTGYGPGVDLMAPGVDIMSLQLLSGTSMATPHVTGLAAYFASIY

TANL

>KFG81372.1

YVIYLRRRGVDFIEQVQKSATWNLGRISNYVYDAGEGTTYVIDTGVDDTHEFEGRALQIH

GTHVAGIIGSASYGVAKKTKIFGIKVLDSNGDAEGDRLIAGVVNYSINSINVAAAELVAA

GNSPASEAVCTVGSAVDTGYGPGVDLMAPGVDIMSLQLLSGTSMATPHVTGLAAYFASIY

TANL

>XP_007814373.1

YVVYLRVRGVDFIEQVQKGVTWNLDRISHYVYDAGEGTAYVIDTGVDDTREFKGRALQIH

GTHVAGIIGS---GVAKKTRIFGIKVLNSEGSRETDALIAGVVSFSINAISVAAGALASA

GNSPASEAVCTVGSASDYGYGPGLDLLAPGVDILSLQLLSGTSQATPHVTGLAAYFASIY

TANL
