# Supplementary material for: Phylogenomic evolutionary surveys of subtilase superfamily genes in fungi
Source: Sci Rep. 2017 Mar 30;7:45456. doi: 10.1038/srep45456 (PMC5371821; doi:10.1038/srep45456)

**Phylogenomic evolutionary surveys of subtilase superfamily genes in fungi**

Juan Li*, Fei Gu, Runian Wu, JinKui Yang and Ke-Qin Zhang*

*State Key Laboratory for Conservation and Utilization of Bio-Resources in Yunnan*, *Yunnan University*, *Kunming*, *650091*, *P.R. China.*

* Corresponding author: Juan Li and Ke-Qin Zhang

Tel: 86-871-65033805; Fax: +86-871-65034838.

E-mail address: [juanli@ynu.edu.cn](mailto:juanli@ynu.edu.cn) (Juan Li); kqzhang@ynu.edu.cn(Ke-Qin Zhang)

**Supplementary Fig. S6:NJ tree of proteinase K-like family**

The program MEGA 6 [^24^](#_ENREF_24) was used to construct a neighbor joining (NJ) tree with 1,000 replicates.


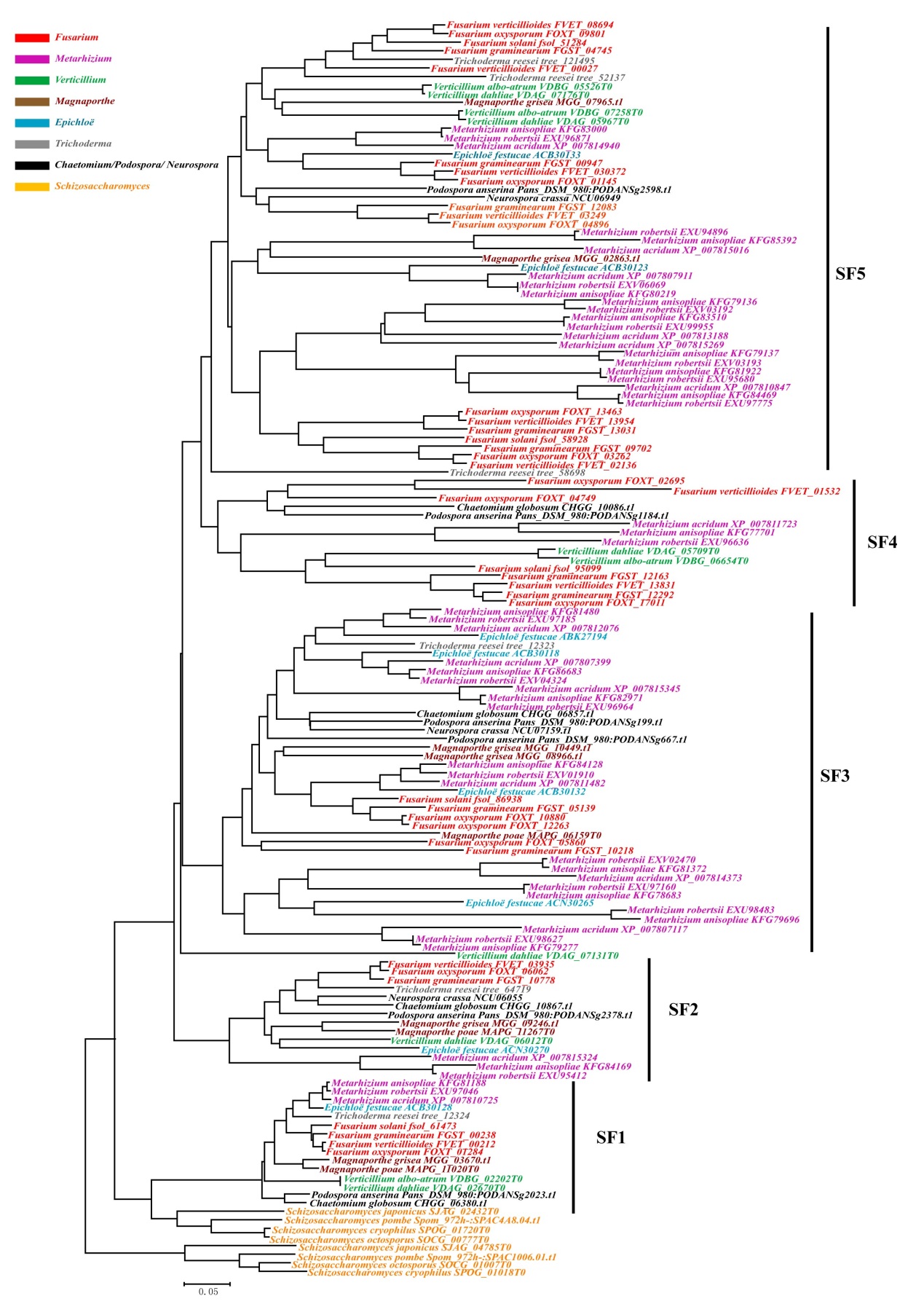

Supplement: Supplementary Fig. S6 [file srep45456-s6.docx]
