# Supplementary material for: Phylogenomic evolutionary surveys of subtilase superfamily genes in fungi
Source: Sci Rep. 2017 Mar 30;7:45456. doi: 10.1038/srep45456 (PMC5371821; doi:10.1038/srep45456)
Supplement: Supplementary Data S7 [file srep45456-s7.docx]

**Phylogenomic evolutionary surveys of subtilase superfamily genes in fungi**

Juan Li*, Fei Gu, Runian Wu, JinKui Yang and Ke-Qin Zhang*

*State Key Laboratory for Conservation and Utilization of Bio-Resources in Yunnan*, *Yunnan University*, *Kunming*, *650091*, *P.R. China.*

* Corresponding author: Juan Li and Ke-Qin Zhang

Tel: 86-871-65033805; Fax: +86-871-65034838.

E-mail address: [juanli@ynu.edu.cn](mailto:juanli@ynu.edu.cn) (Juan Li); kqzhang@ynu.edu.cn(Ke-Qin Zhang)

**Supplementary data S7: 136 pyrolisin amino acid sequences in fungi.**

　　　For pyrolisin genes, MUSCLE v3.5 was used to generate protein alignment with default settings[^20^](#_ENREF_20). The ambiguous areas of alignment were located and removed by using the program Gblocks 0.91b [^21^](#_ENREF_21)^,^[^22^](#_ENREF_22) with default parameters. The gap selection criterion “with half” was used here. Finally, an alignment consisting of 282-bp amino acids from 136 pyrolisin genes were obtained from Gblocks 0.91b.

>XP_007816042

---YYS---------------------AVVDTGDYTNPALGGCFGGCRVAFGDNF--DPMD-CHGTAVAGIVAGVAPNATLAGYRVLNCDDLIGWVKAYEDGAQIIVSSAGAAVVSRIVVPCIVGLGNNGSGFSTLNPSSGRGVTSVNAFAR---------ARFSTFVPNLEIKPTVGAPGDVPGI--GTSFAGPAGILALAVP--VAQQGGGLARAWDATLVENDTITNAKVNVTYRLDTLAAVLGPGLP------PYLGGRLPVGEYKLAVRAVRLFGDR

>XP_007813810

YIFFKGYPVHVMTVDKLRGVTGKGVKIAVVDTGDYKHPALGGCFGGCLVAFGTDLVPDPMD-CHGSHVAGIVAGAAPGATLGAYRVFGCDVLIAFNQAYQDGANIITASIGAEAVSRIVVPCTVSAGNGAEGFYASTAANGRRVSAIASYDNDGCVLIRGASTFTSWGPTMDTKPQFGAVGGVLSTYPGTSMSCPAGIIALANPAPVPQQGGGLIQAYDATLLSNDTLQNESKKITYKITHTPATLNGGLALWSGYIPYQGGRLPPGKYRFVVRALRIFGDW

>XP_007812468

YFIYKAHPVHVMMVDKLRGITGKGIKIAMIDTGDYEHPALGGCFGGCLFSFGADLV--PKD-CHGTNAAGIIGGAAPGAQLGMYRI-TCDVMVAIYRALEEGVDIISSSAGSSAATRVVVVFVQGAGNGELGFSQLDPAVGSGVISVGSVHSHACVLLHGASSFSSWGPSLGLKPSLTAVGEIITTSPGTSFSGPAAIVALSHPAPVAQQGGGLARAYDATLVQNDTIKNGAGVVTYRLSHVPATVAPGMPLWSGWIPYQGGQLPEGYYKLVVRALRIFGDW

>XP_007812048

LIIFNGWPAHLMTVDKLHGYLGSGIKIATVDTGDYKHPALGGCFGGCKVATGENF--KPID-CHGTIVAGILAGAAPNATIMAYRVLNCDDMIGWLKAKEDGAQIIISSIGAMVAARIVVPCVAGLGNRDNGFYAMNPSTGRGVTSVNSFSR---ALIRGAAKLSANGPTLDIKPTIGAPGQVPVT--GSSFAGPAGVLALAEP--VAQQGGGLLRAWEATLVENDTINNAKTEVTYQLANLAATIGAGLPLWSGWVPYLGGAFPPGRYKLVARALSIMGHW

>XP_007811751

FIFFRGWPIHFMTIDKLHGYTGKGVHVAVIDTGDYKHPSLGGCFGGCLVTKGFDFVPDPMD-CHGSHVAGIIAGGAPGVTLGAYRIFGCDVIIAINRAYLDGADIITMSIGAVTASRIVVIVTVSAGNGAQGFYASSGSSGEGVAAIASYDNDLCVLIRGASTFTTWGPTMDVKPQFGAPGGILSTYPGTSMACPAAIYALAKPAPVAQQGAGLIQAHDATLLENETLTNGDGKVTYEISYVPTTLAKGLPVWSGYVPYQGGRLQEGTYRIVVEMLRLYGAW

>XP_007811004

---FTGWPVHHWTVDKLHGMRGKGVKVAVVDTGDYSHPALGGCFGGCKVAGGYDLVPDPM-DYHGTHVAGIIAGVAPGAELLIFKVF-SDVLIAFCDAYTAGADVITASVNALVANRIVVFVSIAAGNGTRGFYSGVGSNGRHVVSVAA------------AYFTSWGPTLIMKPDIGAPGYILSTYLGSSMAAPAGIAALGRNAPPFQVGTGLVDAWKVTQVSLDTITNANQTVIYTFAHESLTLHSGLPLFSGKIPYGGGKLSPGNYT------------

>XP_007809312

YFIYKAYPIHVMTVDKLRGITGKGVKIALLDSGDYEHPALGGCFGGCLVSFGADLL--PKD-CHGTQVAGIIAGAAPGVQLGMYRV-ACDILVAIHLALKEGVDIISSSVGSKTAMRAVVVFVQGAGNGEGGFSNLDPAVSPGVISVGMMQNHACVLMRGASALSSWGPSLGLKPSLAAIGEVVTTSWGTSSSAPAGIVALSHPAPVAQQGGGLVRAYDATLVANDTLKNGQVGVTYSLSHVPATVAPGLPIWSGWIPYQGGKLPEGSYKLVVRALRIFGDW

>VDBG_10161T0

YIVFYVWPNHNSTVWALHGIKGKGVKIAVVDTGDYTHPDLGGGFGGFRVAGGYDFAPDPM-GLHGTHVAGIVAGVAPEATIYAYKTY-AAINIAFLAAYEAGVDIITASIGEEVATRLAVVITVSVGNGDGGFRALSPSGAKGVLAVSNVD-------------TSWGPLLQLKPDVGAPGTIYSTVFGTSMATPAGVAALVRKATIAQVGSGLINATSLTDLVNDTVINSDKPVEYQFTKEDSTLKPGLPLYSGNI----------------TAIV-----

>VDBG_09997T0

---FKGWRSHHMTVDRLHGIRGQGVTVAIIDTGQYTHPAM--------------FVPDPF-DIHGTHVAGIIAGVAPDATLKSYKVFGG--------------DIITASIGSTVASRIVTMVTISAGNGELGFFGSSGSSGKFVLAVASVEAVGCPLSVGASYFTSQAALLEIKPDVAAPGGIFSSYLGTSMACPAGVAALGRAAPVTQVGTGIVDAYKVTSLGNDTIKNGTSRVRYRFEVQDSWVNPGLPVYSGKIPFMGGRLEPGNYT------------

>VDBG_09528T0

FILFKGWHSHYMTVDRLHGLFGKGVKVGVVDTGEYTHPALGGCLGGCKVAGGYDFVPDPM-DYHGTHVAGIIAGVAPEASIYSYKVFGPEVLIAFLKAYEDGMDIITASVGALVASRIVVVVTIAAGNGDVGFFASSGGSAEKVLTVASVEADACPLVRGASDFTSWGSTLELKPDIAGPGGIFAPFVGTSMATPAGVAALGRSAPPIQVGTGLVDAFAVTQLSNDTITNGEKEIEYQFKVQDARVAPG------------GRLAPGKYKMRFAALAPFGNW

>VDBG_09218T0

YIIFSGWLNHIATVDKLHGILGKGVKIGVVDTGAYNHNALGKGFGGFKVAGGYDLVPDPN-DFHGTHVAGIIAGVAPEATLYAYKVFSQ-------------VDIITASIGAVVASRLVVVVTISAGNGAAGFFGTSGGSGRNVVAVASIRNDACALVRGASIFTSWSATLQFKPDVAGPGGIFSAWFGTSMACPAGVAALGVSAPPAQVGSGLINAWKVTLLRNDTVKNGKQSVEYALSYEHGTLKPGLPLYGGK-----GRLAEGKYHIRFATLKPFGNW

>VDBG_09212T0

YIVFRGWQSHDMTVDKLHNIKGKGAIVGVIDTGNYRHKALGGGFGGFKVVGGHDFVPDPD-DLHGTHVAGIIAGVAPEASLYAYKVF-TETLISMLRAYDDGVDIISMSIGALVASRIVVLVVIAAGNGGPGFAADSGSAGVNVVSVASIEPEACVLVRNAATFTS----------------------GTSMATPAGVAALGEPAPVAQVGTGLVNASKVTSLSNDTITNGTRPVTYTFSQQDYTVNPGLPVYSGRIPYLGGELAPGRYRMRVASLRPFGNW

>VDBG_09016T0

YIVFKGWPVHIMTVDRLHGITGKGLRIGIVDTGDYTHPALGGCFGGCLVEFGADVVPDPFEDCHGTHVAGIIAGAAPGVKIGMFRAFSCDALMATSKAYEGGSDIITGSVGALLVSRIVVPCTFSAGNGTEGFGVSAPSTGDGVISVSSFQNDA-------STFTSWGPTLKNKPDVGAPGGILSTLPGTSMSCPASHCC-----------------------------------------------------SGC--------------------------

>VDBG_08435T0

FIIFAGWPVHQATVDKLHGLFGKGVKIGVIDSGDYNHTALGGGIGGFKVIGGYDLVPDPM-DTHGTHVAGIIAGVAPEATLLAFKVFGA-------------VDIITASVAASICSRIVVVVTVAAGNGENGFYASSGSSGNNVLAIASVETDACVLVRGASYTTSWGATLQIKPDVAGPGGIWSTAPGTSMATPAGVAALGTSAPVAQVGSGFINATKVTTLSNDTITNGEKDVTYTFRLEPARVRAGLPIYSGKVPYLGGKLAPGNYT------------

>VDBG_07757T0

YIIFDGWPVHTFTAKEVHDFTGAGQRVCIIDSGDVNHPALKDRIA-----GGKNL-----EDCHGTFVSSVIVGVAPGAEVYMYKIFGCDLVLGLLAADADNCDIISMSVGARVASE-VRLVIIAAGNGQMGYFASSPAVGQGVVSVGSVEADSCVLSKGYSAFSSWGPSNDLAPLISAPGGVYGAFPGTSFSTPAGVAALFDKGPVAQQGAGLIN---VVLLSNDTVQNGNEEVVFNVTHVPSTVAAGGALWSGKIPYLG---------------------

>VDBG_07489T0

YIIFRGWPAHSWTVDKIHGNFGQGVKVAVVDTGDYRHEALGGGFGGFKVSGGYDVV-----DGHGTHVAGIIK----------------------------GADVITCSIGATIASRIAVIITIAAANGAQGFYASSGSSGKEVLAVASAEAQACTLVRNVSEFTSWGGLMDLKPEVSAPGGILSTWLGTSMACPAGIAALGNSAPTIQAGSGLVDAWKVSTLSNDTIANANVDVEYEFVLQPAKLKPGQPLYSGKVSYFGGKLKPGIYK------------

>VDBG_02994T0

YIVFNGWPVHLMTVDKLRGFTGKGLHIGVVDTGDYRHPALGGCFGGCLVTKGWDYAPDPIDTCHGTHVSGIVAGAAPDVTLGVYKTSGCEVLVGIYRAYDEGADIISLSAGADAVSRLAVPVIVATGNGGLGWNAASPASGREVG-------PTA-PMRGGSGFTSWGPSMVLKPQFTAPGGILSTWTGTSMSAPAAIYGLAK-APVAQQGAGLVQAYDATLVDNDTLENAAEAVTYTFGHAKATVPAGLPVYSGYIPYVG-PSPEGAFQFVSNALRIHGDW

>VDAG_10474T0

YIVFYGWPNHNSTVWALHGIKGKGVKIAVVDTGDYTHPDLGGGFGRFKVAGGYDFAPDPM-GLHGTHVAGIVAGVAPEATIYAYKTY-AAINIAFLAAYEAGVDIITASIGEEVATRLAVVITVSVGNGDGGFRALSPSGAKGVLAVSNID-------------TSWGPLLQLKPDVGAPGTIYSTVFGTSMATPAGVAALVRKATVAQVGSGLINATSLTDLVNDTVTNSDMPVEYTFTKEDSSLKPGLPLYSGNIPFLGGKLANGEYVLRFAALVPFGNW

>VDAG_09626T0

YIIFDGWPVHTFTAKEVHDFTGAGQRVCVIDSGDVNHPALKDNIA-----GGKNL-----EDCHGTFVSSVIVGVAPGAEIYMYKIFGCDLVLGLLAADADNCDIISMSVGARVASE-VRLVITAAGNGQKGCFASSPAVGQGVVSVGSVEADSCVLSKGYSEFSSWGPSNDLAPLISAPGGVYGAFPGTSFSTPAGVAALSDKGPVAQQGAGLIN---VVLLSNDTVQNGNEEVVFNITHVPSTVPAGGALWSGKIPYLG---------------------

>VDAG_07367T0

YIVFRGWQSHDMTVDKLHNIKGKGAIVGVIDSGNYRHKALGGGFGGFKVVGGHDFAPDPD-DAHGTHVAGIIAGVAPEASLYAYKVL-SETLIAMLRAYDDGVDIISISIGALVADRIVVLVIIAAGNGGPGFAADSGSAGVNVVSVASTEPEACVLVRNASTFTSVGPGLALKSDISAPGSILSTYLGTSMATPAGVAALGEPAPVAQVGTGLVNASKVTSLS---------PITYTFSQQDYTVNPGLPVYSGRIPYLGGELAPGRYRMRVASLRPFGNW

>VDAG_04865T0

FIIFQGWQAHHMTVDKLHNIKGKGAKIIIIDTGNYRHKALGGGIGGFKVAGGYDFVPSPD-DQHGTHVAGIIAGV--DASLYAYKVF-TDTIIAMLRAYDDGVDIITMSLGSLVASRMVVLVINSAGNGLFGFATSTGAAGANVVSVASVEPEACVLVRNASLYTSFGPDLAVKSDISAPGSILSTYLGTSMAAPAGVAALGEPASVAQ---------------------------------DYTVKPGLPVYSGRIPYLGGALAPGWYQMRVAALRPFGNW

>VDAG_03685T0

YLFFHGRTLHLMTVDMLRGYTGKGFKIAVIDTGDFKHPALGGCFGGCLVSYGHDYVP-TMD-CHGTH------------------IFGCDAIAAFAQAFEDGSDIISASLGSVIVQRIVVPCVVATGNGASGFFASGPVDGIGSTAVASFDNDACVLIRGASTFTSWGPTAELKPQFAAPRGILSLWPGTSMATPSGVLALAKPAPVPKQGAGLIQAYEATILSNDTIKNGDADVTYTLDHRPATVKAGLPVYSGYIPYMGGQLPAGRYKFDVKVLRVFGDY

>VDAG_02277T0

YIVFRGWPAHSWTVDKIHGNFGQGVKVAVVDTGDYRHEALGGGFGGFKVSGGYDVVPDPL-DGHGTHVAGIIAGVAPGVDLLAYKVFSS-----------IGADVITCSIGATIASRIAVIITIAAANGAQGFYASSGSSGKEVLAVASAEAQACTLVRNVSEFTSWGGLMDLKPEVSAPGGILSTWLGTSMACPAGIAALGNSAPTIQTGSGLVDAWKVSTLSNDTIANANVDVEYGFLLQPAKLKPG----HGKVSYFGGKLQPGIYK------------

>um03024

YIVFVGYRTHVMSVDKVHGFLGRGQVVGVIDTGDYRHPALGGKFGGCLVIGGYGFVASPYD-CHGSHVTGTIAGVAPHAKIRAYRVFGCDIIIALQRAFFDGCDVLSLSLGSAVAGRIATPLAIANGNGAFGAYASSPGTGSNVMAVGSVQNDACVLIGGASSFSTYGPSNKLNPVVSAVGGVLSTYPGTSMATPSGSIAVATPESVAHQGTGLVDVNKAIVLSNDTVKNGSKPLAYTLSHIPV-VPPKLPFYSGYIAYGG---TPGTYKVLLRVQRVLTDY

>tree_60791

---FNGWPMHQWTVDQLHGIRGKGATIAIIDTGDYTHRALGGCFGGCKIAGGYDLVPDPM-DFHGTHVAGIIAGVAPDARLLSFKVF-ADVLIAFCDAYGAGADVITASIGALVASRIAVVVTISAGNGNTGFYSSSGANGHNVLSIAA------------AIFTSWGPTLLLKPDVGAPGFIISTVLGTSMAAPAGIAALGVSAPPFQVGTGLVNALKVTQLYSDSITNGNRTAVYSFEIEPQALAPGLPLFGGKVPYGGGKLAPGNYTLRFAALRPYGHW

>tree_57433

---FRGWPVHVLTVDKLHGFTGKGIRVGLVDSGDYNHPVLGGCFGGCIISYGKDLIPDPQD-CHGTHVSGIIAGAAYGANLGMYKALNCDVLIAFNEAYEDGSDIISLSVGAIAVSRIIVPCVIANGNGPF-FSSATPADGKGVMAVASIENNACVL--NADEFSSWGPTADLNPRISTPGGILSTYPGTSMAAPASIYALAN-APVAQQGAGLAQAYDATTLSNDTITNAKQAVTYSVGHVPTTVPAGLPVYGGYIPYLGGVTPEGNYAVLLRALKIFGDY

>tree_51365

YIFFKGWPVHQMTIDKLRGYTGKGVRVAVIDTGDYTHPALGGCFGGCLVSFGTDLVPDPVD-CHGSHVAGIIAGGAPDVTLGAYRVFGCDVLIAYNQAFEDGAQIITASIGAVAVTRIVVPCTVSAGNGDSGFFASTAANGKKVIAVASVDNDGCVLVRGASGYTTWGPTLDVKPQISSPGGILSTYPGTSMACPAAAVALANPAPVPQQGGGLIQAYDATLLSNDTLKNSKQRVTYKLNHVPTTLPAGLALWSGYIPYQGGELPPGTYKAVFHALRIFGDW

>tree_35726

YIVFKGWPVHAQVVDKLHGITGKGIRISIIDTGDFNHPALGGCFGGCVVSYGRDMVPGPLSTCHGTHVTGIIGGAAPDATIGMYRVFGCDVAIAVNQAFEDGTDIITLSLGSVAVSRIVVVCTVANGNGSAGFLAGTPANGKGVTAVSSVESGACIL--NADTFTSWGPTADVNPSISTPGGILSTWPGTSMATPAAIYALAL-ASVAQQGAGLAQAFKATLLNNDTIHNGKQSVTYSIGQTPATVAAGLPVYSGYVPYLGGMTPEGSYKFLIRAAKIYGDY

>tree_109276

FIVFYGWSVLDMGVQKLHGIKGKGVQVALIDSGDYTHPALGGGFGGHKVALGYDFVPDPLATCHGTHTAGIVAGVAPEATLAAYRVFGCDILIAMIQAWRDGADIVSMSLGEDVVDKLTVAVVASIGNGTAGYSSSTPAISLSALSVGSIESDAWLQVSGVDYFSSFGPTMTLEPKISAPGGILSTFPGTSMSAPSGSLALARP----RQGAGMIDAYAATVISRDSISNSRSRKRYSLQHVPGTLGPGNPVYSGFIPYLGG-LAAGDYRVLFRTLRWRGDY

>SS1G_09060

FVVFTGWPVLEMGIDKLHGIKGKGIKIGIVDTGDYRHPALGGGFGGFKIAGGYSFVGNPLSTCHGTHVSGILGGVAPEASLYMYRTFDCDTIMGMLKAQGDGVDIISMSLAASVVQSITIAVIVAVGNGSASFAADYPAVDPSAIAVGAIANGTWFEAFSSDYYSNFGPNYDLKPQISAPGGILSTYPGTSMATPAGCFALATP----QQGAGLINAHDASVISGDDIENSGSSKTYTLSHVGATLASG-PVFGGFIPYIGN-AGDGDYRFFASVLRWGGDY

>RO3T_16658

YMIFHGSPVHHMTVDLVHKNKGKGILVGVLDTGDYMLPALGGGFGGYKVVTGYDLVPDPLDACHGTHVSGIIAGVAPEANLAMYRVFGCDVIVALLMAYDAGADVINLSLGKVVNQ-IVVHVVISAGNGEDGYTLSSPSSARLAFSVASVENDACALVKGASSFSSMGPSLIFKPNIAGVGGVFSTLPGTSMASPAGSIALASPDSPIRAGAGLVQVYDTIHVSNDTVTNGNVTVQYRLTQLSSTLAPGHIYYGGYIPYIGGTYPAGVYQFRLSALKLFGDW

>RO3T_16311

YIIFRGYPVHAITVDKVHKLTGKNIVVGIIDSGDYRHPAFGSGFGGFPVRYGYDLVEKPLDACHGTHVAGVIAGIAPQVTLGAWRIFGCDLVIALISAHEAGCDIINLSLGSIVANRVSSIVIAAAGNGNDGFYISAPGSGTSTVSVASTDNDACVLVQGASLFSSVGPLVSLKPDIAGPGGIFSTLPGTSMAAPTGAFALAQPDNPARQGAGLIQVFDAIHISNDTISNSKETVSFEISHEAGTLAP------------GATVSNGSYYLRWKALKLLSSW

>RO3T_15504

YIVFKGAPVHHMTVDIVHKNKGKGVLVGILDSGDYKHPALGGGFGGYKVVTGYDLVPDPLDECHGTHVSGIIAGVAPEANLAMYRVFGCDIIVGLLMAYDAGVDVINLSLGSIVNQ-IVVHVVISAGNGAQGYTIGSPSTASSAFSVASVQNDACALVKGASSFSSLGPSLILKPNIAGVGGIYSTLPGTSMASPAGSVALASPDSPARAGAGLVQVYDATHITNDTVTNGSKTIQYELVNQVSKLAPGHIYYGGYIPYVGGTYPAGTYRFRVSALKLFGDW

>RO3T_12286

YILFNGWPIYELTANYAYKLNGSGVKVGIIDSGDYTHPALGGCFGGCKVAYGYDLVKPPIDNCHGTFLAGIIAGVAPGVTLGMWKVYGCDIIVALEMAYKAGMDIINLSLGAEIVSRIVVHVVAAIGNGSNGFLPSSPASGKDVIAVGATMNDACMLIHGASGFSSQGPTLQLKPEIMGVGGVFSTYPGTSMAAPSGQIALATQDSPIRQGAGKIDVVQAFHAFNDTFYNKHNSLTLHLHHQPSVIPPGHAIYGGYIPYLGRNQ------------------

>RO3T_12258

YIIFRGYPVHKTTVDRTRNLQGKGVVVGIIDSGDYRHPAFGNGFGGYPVSLGYDLVERPLDTCHGTHVAGIIAGIAPQVTLGAWRIFGCDLVIALIDAHEAGCDVINLSLGAIVANRVSSIVVAAAGNGIDGFYISAPGTGQGTVSVASVDNDACVLVQGASSFSSVGPLVSLKPDIAGPGGIFSTLPGTSMASPAGALALALPSHPVRQGTGLIQ------------------------------------------------------------------

>RO3T_12236

FLIIHAWPVYNQTINKLRGLSGQGIKVGVIDTGDYTHPALGRCFGGCRVAYGYDFVPDPRDICHGTHVAGIIGGVASE---GAYRIFGCDIIMAMERAYLDGMDVINLSLGSILADELSMIVCAAAGNGDRGFEVGSPSLGKHAISVASIDNDACVLVGGASSFSSWGLGLSLKPDISAPGGIYSTYPGTSMASPAGVVALGHP----RQGAGLIDVYQATMITNDEIKNGRLDTEYTITHQASE--AN--IYSGYIPYAGGDVFSGKYRLKLTALRPLGDF

>RO3T_05734

YIIFRGYPVHQMSVDQVHKNKGKGILVAVIDSGDYMHPALGQGFGDFKVVKGYDFVPDPMDSCHGTHVSGIIAGVAPEAKLAMYRVFGCDVVLAILKAYDEGADIINLSLSPVIEQ-MAVQVVIAAGNGKRGYSVGIPGTSLGAYTVASIENSACALIYGASTFTSVGSTLDLKPNIAAVGGVFSTLPGTSMATPSGSLALAIPDNPIRIGAGLVQVFDATHITNDTITNGQQTAQYDVVHQPSKLPPGHLFYGGSIPYFGGTYPDGTYKIRLSVLKLFGDW

>RO3T_02460

YILFNGWPIYELTANYAYNLDGSSIKVGIIDSGDYTHPALGGCFGGCKVAYGYDLVQSPIDNCHGTFLSGLIAGVAPGVTLGMWKVYGCDILMAMEMAYQAGMDVINLSLGAIMANRIVVHVAVANGNGPNGFLSASPASGKDVIAVGSIMNDLCLLVHGASGFSSQGPTLQLKPEIVGVGGVFSTLPGTSMAAPSGQIALATQDSPIRQGAGIINVAQAFHASNDTFYNKHSSLTLHLTHQPSVIPPGHAIYGGYIPYLGGDYKSGTYQLKIKALRVFGDW

>Ptri_08228.t1

HIVLQGWSNHVMTIDKLHNITGKDVKIAIIDSGDYTLDALGGCLGNCLVAGGYDFVPDPMDNCHGTHIAGIISGVAP-----------------------------------VRYLPIR--CDVASGN---------------VASIASFVNMGCVLMRGASDFSSWGPTLDFKPQFGAPGEILSTYPGTSMSCPAGIYALSKPAPVAQQGAGLLQAYDATALSNDTISNGHKAITYQLDVVNATVPAGLPVYGGYIPYQGGQLPAGTYKMRVRAMHLFT-Y

>Ptri_07828.t1

YLVFKGWPVHVIGVDKLRNLTGKGIFIGVIDGGDFMHPALGGGFGGFKISAGEDLVPGPM-DCHGTHVSGIIGGVAPEATLGVWKIFGCDMIMGFNIAYEAGVDIISASFGSVVVQRISVVVVVAAGNGTIGFDAQAPANSAGALAVASIDNTACTLVQGASDFSSWGPTLSIKPEVAAPGGILSTWPGTSMAAPAGTIALADPAPVMQQGGGGLNAYKATTVDNDTIKNFKQAQTYTFDNL--TVEAGLPLYNGYVPYMGGTLPAGTYKLILRALKIFGDY

>Ptri_00647.t1

FIVFKGWPSHVMTVDKLHGITGKGFRISVVDSGDWTHEALGGCFGGCLIEAGYDFIPDPMDNCHGTHVAGIIVGSAPGAKLGMYRMWGCEIEFAFARAVEEGADIISYSNGAILVSRIVIPVVVSEGNGGKGFYASTPATAVSATGAGAVTNNACVLVEGGGLLSTWGPSLNMTPQLVAPGEILGTWPGTSMSSPAGVYALAK-APVPQQGAGIVQAWNAIELDNDTISNGSTDEVLKMGHRKATIPAGLAVYSGFIPYMGGLLDAGVYKMRVRALRIFGDW

>Pnod_SN15_SNOG_15948.t1

YIVFKGWPSHVMTVDKLHGVTGKGFRISIIDSGDYTHPALGGCFGGCLVEIGYDFTPDPMDNCHGTHVAGTAAGSAPGAKLGAYRMWGCEIELAFARAVEDGASIISYSNGAVIISRIVIPVVVSEGNGGQGFYASTPAAGGSITGTGAVSNNACVLLEGGGQLSSWGPSLDMTPQVVSPGEILSTYPGTSMASPAGIYAMSK-APVAQQGAGIVQAWEAIELDNDTIKNGSEEAVLQIGHRKATVPAGLPVYSGFIPYLGGLLEEGTYRLRVQALRIFGDW

>PGTT_19401

LLVFYGRRAQVQSITELHGIYGKGVKVALIDSGDCSHPALGKGFGGFKIAFGKNYANHPCTQCHGTHVAGIVAGVAPNATLGMY-LFGCDSLVAMLQAHKDGADIISASIGTTVNKLVQAIIIVAAGNGSEGFFGDNPASAKNAISVGSVEADACVLVKGGSNFSQYGPSFESPPAVAAVGG---TYPGTSMATPAGVAAL---DSAVHQGGGLIDAFCATTISNDSIFNGTGVFNYVLTHRPA-LPPGLTVYSGFIPYYG---PNGNYKVLLRALRVTGDY

>PGTT_18576

---FYGFPLHVQTITDLHGILGQGIKVAIIDSGDCAHPALGGGFGGKRISFGIDLV-------HGTHVAGIVAGAAPKAEIGMY-LFGCDIILALLMAYKDGADVINLSLGDVINTLVQAVIIASAGNGAEGFMASNPASSKNAISVGSVDADACVIIRGASNFSQSGPSFLSPPALSGVGG--ATYPGTSMAAPAGVAALARVESTVHQGGGLVNAFCATSVSNDSLVNGTQPVTYRVEHLPA-VMPGLHVYSGYIPYYG---PGGKYKVLLRALKVTGDF

>PGTT_17142

YLVFYGRRAQVQSITELHGIYGKGVKVALIDSGDCSHPALGKGFGGFKIAFGKNYANHPCTQCHGTHVAGIVAGVAPNATLGMY-LFGCDSLVAMLQAHKDGADIISASIGNTVNKLVQAIIIVAAGNGSEGFFGDNPASAKNAISVGSMEADACVLVKGGSNFSQYGPSFESPPAVAAVGG------GTSMATPAGVAAL---DSAVHQGGGLIDAFCATTISNDSIFNGTGVFNYILTHRPA-LPPGLAVYSGFIPYYG---PNGNYKVLLRALRVSGDY

>PGTT_16096

HAVFSGRPVHVQVISRLHGYRCKGINIAVIDTGDCQHPALGKGFGGFKIAKGYDFVPDPCTSCHGTHIMGVLAGVCPDATMSSYRIFGCELVAALLRAYKDGADVFSLSVGAVIASRIARAVVVSAGNGDEGFFATSPSTGTGAISVGSVQSDACALVRGTSPFSNYPQSVMQ---------------GTSISAPAGIAALATPEC-------LVNAWCATVVSNDTITNGKRSVSYTVEHVTA-LRPGLPVYSGFVP---GKIPDGKYRILVRALRVTGDY

>PGTT_12163

LLVFYGRRAQVQSITELHGIYGKGVKVALIDSGDCSHPALGKGFGGFKIAFGKNYANHPCTQCHGTHVAGIVAGVAPNATLGMY-LFGCDSLVAMLQAHKDGADIISASIGTTVNKLVQAIIIVAAGNGSEGFFGDNPASAKNAISVGSVEADACVLVKGGSNFSQYGPSFESPPAVAAVGG---TYPGTSMATPAGVAAL---DSAVHQGGGLIDAFCATTISNDSIFNGTGVFNYILTHRPA-LPPGLTVYSGFIPYYG---PNGNYKVLLRALRVTGDY

>PGTT_12076

FIVFYGKPIQIQTVSELHGVFGEGIKVAFLDSGDCDHPALGPGFGGHKIGFGYDLVPDPCTQCHGTHVAGIVGGVAPNATLGMY-LMGCDIVMALLMAVRDGADVISASIGDLINNLVSVALVLAAGNGDEGFYAETPAAATNSIAIGSVESDACVVIR--SNYSQYGPSSMNIPNFLGVGGILSTVPGTSMSTPAGITALAQQQTTIHAGGGLVDAFCATVLSHDLITNGDQSYSFRTGHIPA-LAAKLPVYSGYVPYYG---PSGYYKILIRALRVNGDF

>PGTT_10975

HAVFSGRPVHVQVISRLHGYRCKGINIAVIDTGDCQHPALGKGFGGFKIAKGYDFVPDPCTSCHGTHIMGVLAGVCPDATMSSYRIFGCELVAALLRAYKDGADVFSLSVGAVIASRIARAVVVSAGNGDEGFFATSPSTGTGAISVGSVQSDACALVRGT------PQSVMQ---------------GTSISAPAGIAALATPEC-------LVNAWCATVVSNDTITNGKQSVSYTVEHVTA-LRPGLPVYSGFVP---GKIPDGKYRILVRALRVTGDY

>PGTT_10542

YLVFCGSRPHIQTISKLHGIAGQGVKVALIDTGDCTHPAFGNGFGGFKIGFGRSFVSEPCTRCHGSSTAGILAGVAPSINLGMY-LVGCDVIIAMLQAQKDAADIISISLGRVANKLVQVIMVASAGNGHNGFIGESISSSKNVIAVGSVDSDACVLIKPG-----------------GVGGILATLPGTSMAAPAGIIAL---AAVVHQGGGLIDAFCATTISNDSISNGTSFVDYVLSHRPA-LSPGLAVYSGYIPYY----------------------

>pchr_133799

FIVFVGRPVHVITVDKLHGITGKGIKIGIIDTGDFTHPDLGGGIGGFKIIGGFDFVPDPLDQCHGTHVAGIIGGVAFDASITSYRIFGCDVIVALLRGVSEGQDILTMSLGSVVSSRISKIVTIAAGNGADGFFTSGPGNAIDAISVASLDNDACVIVRGGSSFTSYGPTMFFKPAVSAPGGILSTFPGTSMATPAGVSALAQLQTVAQQGAGLVNAFQAIIITNDTIKNGKTAESFKISHVPA-VHPGLPVFSGFITYLG---PNGLYKILLRVLKVTGDF

>pchr_133613

---FVGHPVHVMTVDKAHGLTGAGIKIGIIDTGDYNHPFLGRGIGGHKIIGGYDFVPDPLDECHGTHVAGIIGGVAYNASLSMYRVFGCDIIIALLRAFNDGNDVLTLSLGGVVASRIAKVVTIAAGNGAYGWYTSSPGTGKDVISIASIDNDACVIVRGGSSFSSYGPTMYFKPAVAAPGGILSTYPGTSMATPAGSAALAAPQTLAQQGSGLIQVDKATVISNDTIKNGTKPLTYRLSHVAA-VPPGLPVYSGFIAYLG---GDGTYKVLVRALKVNGDY

>pbla_79866

YVVFSGYPVHAMTVDRVHKKFGKGVKVGVVDTGDYLHPALGGGFGGFKVQYGYDLVPDPIDSCHGTHVSGIIAGVAPQATLGMWRVFGCDVLVAFLMAYDAGMDVISVSIGTIVAQRIAIPFIVSASNGADGFTVGMPSTAKDVWSVASVDNDACAIVQGASDFSSVGASLDLKPNVAGIGGVFSTLPGTSMAAPSGSVALALQENPLLQGAGLVQVYDAVRISNDTIYNGKVRTTFKIINEPSTLNAGHIMYGGFIPYFGATYPSGNYILNLRALKLFGDW

>pbla_77720

YIVFNGYPVHAMTVDRVHKKFGKGIKVGVVDTGDYLHPALGGGFGGFKVQYGYDLVPDPLDSCHGTHVSGIIAGVAPQATLGMWRVFGCDVLVSFLMAYDAGMDVISVSIGTIVAQRIAIPFIVAAGNGADGFTVGMPSTAKDIWSTASVDNDACALVQGASDFSSVGASLDLKPNVAGIGGIYSTLPGTSMATPAGSVALALKESPLLQGAGLVQVYDAVRVSNDSIYNGKVRATFKVINEPSTLNPGHIMYGGYIPYFGATYPSGTYILHLRALKLLGDW

>pbla_65757

YVVFNGYPVHAMTVDRVHKKFGKGIKVGVIDTGDYLHPALGGGFGGFKVQYGYDLAQDPLDSCHGTHVSGIIAGVAPQATLGMWRVFGCDVLIAFLMAYDAGMDIISVSIGTIVAQRIAIPFIISAGNGSEGFTVGMPSTAKDVWSIASVDNDACALIQGGSDFSSVGASLDLKPNIAGIGGVFSTLPGTSMAAPSGSVALALKQSPLLQGAGLVQVYDAVRVSNDTIINGKARTTFKVVNEPSTLNAGHIMYGGYIPYFGATYPSGTYILNLRALKLLGDW

>pbla_64400

YVIFNGYPVHGMTVDRVHKIFGKGIKVGVIDSGDYLHPALGGGFGGFKVQYGYDLVPGPLDSCHGTHVSGIIAGVAPQATLGMWRIFGCDVIVALLMAYDAGMDIISASVGAIVAQRIAVPFIVSSGNGDRTFTVTAPSTSKDVWSIASAENDACALVQGASDFSSIGASLDLKPNIAGVGGVFSTLPGTSMAAPSGSIALAMKESPLLQGAGLVQVYDAVSVSNDSIHNGKARAVFKVINEPSTINSGHIIYGGYIPYFGATYPSGTYILSLRALKLLGDW

>pbla_64399

YIVFNGYPVHGMTVDRVHKKFGKGIKISVVDSGDYLHPALGGGFGGFKVQFGYDLVPDPLDSCHGTHVSGIIA------------------LLAFLMAYDAGMDVISVSIGTIVAQRIAVPFIVSTGNGSDGFTVGIPSIGKDVWSIASVENDGCALVQGASDFSSIDASLDIKPNIAGIGGIYSTLPGTSMAAPSGSIALAMKESPLLQGAGLVQVYDAVSVSNDSIHNGKARAVFKVINEPSTINSGHIMYGGYIPYFG---------------------

>pbla_62430

YVVLRGWPIAMLTANNAYGQNGSGIKIGVIDSGDYTHPALGGCFGGCKVAYGYDLVKEPIDSCHGTFVSGIIAGVAPGATLGMWRVFGCDILIAMEMAYNDGMDIINISLGSVVADRLVVHVVAASGNGTSGFLTAAPATGRNVISVASTSNDACVLIRGAASFSSLGPTLQLKPELTAVGGVFSTMPGTSMSAPAGSVALANPDSPIRQGAGLVNVVQAFHVSNDTLHNKSTPLKVKLSHSPSTIPPNHVIYGGFFPYIGGVYEPGVYRIRVRALNVFGDW

>pbla_59778

YIVFHGYPVHEMTVNRVHGLTGKGVVVGIIDSGDYTHPSLGGGFGGFKVQFGADLSKGPLDTCHGTHVAGIIAGVAPDVTLGMWRVFGCDLVIALILAYEA--------------------VVAAAGNGSEGFMVASPSVADNVISVASVDNLACVLVKGASSFSSVGTSLDLKPNLAGVGGIFSLLPGTSMASPAGAIALALPDNPAKQGAGLIQVYDASYVSNDTITNEKEPMTYTIRHFSNTVQPGFPMYGGFIPYVGGSIKNGTYYMRWKALRLLASW

>pbla_59777

YIVFRGYPVNGLSVNDVHNLTGKGITIGILDSGDYNHPALGGGFGGYKFKYGYNLVPEPFDPCHGTHVSGIIGGIAPDATFGMWRIFGCDTVIALEMAYEAGCDIINLSLGAVVADRLTVIVVGVAGNGNQGFMQNTPGSGKNSISVASVDNTGCLLVVGASAFSSVGPTLDLKPGVSGIGGVYSTLPGTSMAAPAGVTALARLDHPLRQGAGLVQPYTSIHISNDTVTNGKRPLCLTIENIPSTVSPGYPIYGGFIPYFGGKVSKGTYFLRIRALKLLGDW

>pbla_58746

YVIMNAWPVYGTTVTRVRGFEGEGIKIGVIDSGDFLHPALGGCYGGCKVAYGYDFVPNPRDVCHGTHVAGIIGGIAPKVTLGAYKIFGCDVIMALEKAFTDGMDIINLSIGAIMADMIAVTVLASAGNGDQGFKVNVPAL------------DGCVVIAGASPFSSWGLGLSIKPDISAPGGIFSTYPGTSMACPSGIVALGSP----RQGGGLINIEKMTLIFNDKIKNDTKPVIYRFSHMPAEIPAK--IYSGFIPYAGGIIKAGYYRLRIMALRVFGDY

>Pans_DSM_980_PODANSg4588.t1

YIVFKGFPVHLMTVNKFKGITGKGIKIAVIDT-------------GCLVSYGADLVPDPVDNCHGTHVAGIIAGAAEGVQLGAYRVFGCDLLIAYNMAYEAGSDIITASIGAAVVSRIVVPCVVSAGNGAAGFYASTAANGKQVTAIASVDNHGCVLIRGASSFTSWGPTVESKPQFSTPGGILSTYPGTSMACPAAIYALAKPAPVPQQGAGLVQAWDATLLSNDTVSNGSSSVTYSLSNVGATLGAGLPVYSGYIPYLGGRLPAGRYKLTVRALRIFGEY

>NCU00263.t1

YIVFKGYPVHLMTVNKFRGITGKGIKIAVIDTGDYLHPALGGCFGGCLVSYGTDLVPDPMDTCHGSHVLGLLSGAAPDVTLGAYRVFGCDILIAYLKAYDDGSDIITASIGAAVVSRIVVPCLVSAGNGATGFYASTAANGKRVTAVASVDNNGCVLIRGASTYTSWGPTVDVKPQISSPGGILSTYPGTSMACPAATWALAHPAPVAQQGAGLIQAWDAALLSNDTVTNGKKAVTYQLGHTSATLNPGLPVYSGYIPYQGGALPADTYKITLKALKIYGDW

>MGG_13977.t1

FIVFRGWHSHHMTVDELHGILGKGVKVAVVDSGDYTHPALGGAFGGNKVVGGYDLIPDPM-DHHGTHVAGIIAGVAPEATLLAYKVFGEDTLIAFLRAYQDGADVITASIGALVASRLVIVVTISAGNGQAGFGASSGATGINVVSVASVQGEACVLARGASAFTSIGSTLAIKPDVAAPGGIFSTFLGTSMSCPAGIAALGQPAPINQVGTGLINATKVSALSNDTVTNGSTPVTYTFQATPFTLQPGLPLYGGAIPYMG---------------------

>MGG_10445.t1

YIAFNGWPVHVQTVDKLHGLTGKGVRISIIDSGDYLHPALGGCFGGCIVSYGYDLVPDPLDQCHGTHVAGIIGGAAPGATLGMYRVFGCDVLIAVTKAFEDGSDIITGSLGSVTVSRMVVHVTFAAGNGTEGFGISAPSTGDGVLAISSYEADA-------SRFTSWGPNLQLKPQFGAPGGILSLWPGTSMATPAGVIALAKPAPVVQQGAGLIQALDAITFDNDTVTNGQEVATYNVSHSPATLAPGLPMYSGWVPYFGGSLPAGRYRLGISALKIMASW

>MGG_09990.t1

FIVFRGWYNHRATVEALHGILGQGALVGIIDTGDYTHDALGGGIGGFKVAGGYDFVPSPR-DRHGTHVAGIVAGVAPEATIYAYKVL-AATLVAFMRAYEDGMDVISASIGAVIASRLVVVVTIAQGNGFAGFHGGTGSEGKNVIAVGSVNADACPLVRGASDFTSWGGSLGFKPDISAPGGIFSTWPGTSMATPAGVAALGEGAPTAQLGAGLVNAVKVTTLDNDTVRNADGPVTYNFSLVAHTLQPGLPLYGGKIPYMGGVLERGSYKMRFAALKPFGDW

>MGG_09817.t1

YIVFTGWPNHAATVDRLHGILGQGVKVAVVDSGWYKHPALGGGFGGFKVAGGWDFVPDPL-DTHGTHVAGIVAGVAPNATILAYKIMATATIIAWLRAYSDGADVITMSISAVLAARLVVVMTVSAGNGADGYYAGDANTSPHLLSVASVGADACALVRGASVFSSWASTLQLKPDVAAPGGIFSTWPGTSMATPAGIAALGSSAPPLQMGSGLVDATRVTALDNDTVTNGAAAVSYSFALEPASLGPGIPAYSGKVPYMGGKMAPGNYTMRVAASRPFAGW

>MGG_09352.t1

FIVFRGWHAHDMTVDKVQGILGKGVKVGVVDTGDYTHPNLGRCFGGCKVEGGYDFVPDPQ-DAHGTHVAGIIAGVAPEATLYSYKIFGFETIIAFIKAYEDGMDIISASVGAEVSRRIVIVVVIAAGNGELGWAMSSGASSPDALAVASVDADACPLIRGASTFTSWGPTLSLKPDIAAPGGVLSTYLGTSQATPSGVAALGKTAPPIQVGTGMVDAVAVTDMSNDTITNGQESVEYTFTVQDAVLAPGLPIYSGKVPYFGGKLAPGRYQMRVAALAPFGNW

>MGG_09073.t1

YIVFKGWPQHLMTVNQLRGVVGKGIKVAVIDSGDWKHPALGGCFGECLVSFGYDLVPDPMD-CHGTHVSGIIAGAATGVTMGHYRVFGCDVLIAYNMAYQDGADLITASIAAVAVTRIVVPCVISAGNGATGFFTSSAADARGATAIASFDNNACVLIRGASTYTSWGPTGQVKPQVAAPGGILSTYPGTSMACPAGVYALAKPAPVPQQGAGLIQAYDATLLSNDTISNGSSSVTYTLSQRGAEVGPGLPVYSGYVPYMGGILPSGHYKIVFRALRVFGEY

>MGG_08436.t1

FIVFTGWPMHKYTVEHLHGLSGKGVTVAIVDTGDYSHPALGGCFGGCKVAGGYDLAPDPR-DRHGTHVAGILAGVAPGATLLAYKVFGSDILIAFLMAYKDGADVITASIGAEVASRLVVVVTISASNGQTGFFASTGASGKNVISIASAEGDACALIRGA------------KPDVAAPGGIFSTSLGTSMATPAGVAALGAAAPPAQVGSGMVNATKVTTIDNDTLTNGDAAVTYRFSLQPAVVAPGMPLYGGKVPYVGGAMNSGQYTMRFAALRPLMSW

>MGG_08429.t1

VMVFVGWPVHYSTVDALHGVFGKGVRIAVIDSGEYTHPALGGGLGGFKVSGGYDLVPDPM-DHHGTHVAGIIAGVAPEAEILAFKVFGADTLVATIMAYEAGVDIITASIGATIASRIVVVVTIAAGNGEDGVYASSGSSGKDVLAVASVDTTGCVLVRNASYFSSWGGTLEIKPDVAAPGDIYSTYMGTSMATPAGVVALAETAPVAQVGGGLVNATKVTAVSNDTLTNGAAPLTYTFALQPATVQPGLPIYSGKVPYYGGKLVPGTYKMRFAALTPFGGW

>MGG_08415.t1

YMVFKGWPVHVMAVDRLHGITGAGVRLAVVDTGDYTNAILGGCLGGCVVTHGWDAVPDPMD-CHGTHVAGIVAGAAPGVSLGAYRVMDCETIAGMLRAFDDGNHILTLSVSSMTAARIVVPVFVAIANGETGFDPVAPADARHVGSVSSFDPGGCVL--GASSFSSWGPTLRPAPVFGGPGRILSTY-GTSMAAPAASAALAI-PHPLQAGAGLVQLWDAGVLSNDSLRNDAAPAVYRLSHTAASVPAGWPIYSGRVPYVGGLMQEGRYSILVSALRLFGDW

>MGG_07358.t1

YIVFPGWQ-LHGSVKELHDITGSEITVAVVDTGDYLHPALGGGVGGFKVRFGIDLVPRPYAECHGTHVSGIVAGVAPAANLEHYRVVGCDMIIAVLMAQAREVDVLSLSLTSEVLTRISILVVVASGNGWRGFSARAPASAREVLTVGSV-NDACILVGGANGQGSWGPTYDLTS-ILAPGQIWSTIPGTSMAAPAGCAALARPAPVAMQGNGVVDAMGATFISNDTIQNGGEPAEYNLSHKPATITP-CPLYSGFISYSGG-LAEGLYKFKVCALR---AW

>MGG_04733.t1

YIIFTGWYNHWATVDQLHGVFGDGVKVGVVDTGQYTHAALGGGIGGFKVAGGWDFVPDPM-DYHGTHVAGILLGVAPRATMHSYKVFGRETIMAFLRAYEDGMDVITASIGAVVANRIAVVVTIGAGNGEGGYYASSGSSGEYVLAVAS------------SYFTSWGGLLSVKPDITAPGTIYSTYIGTSMATPAGVAALGSPAPVHQVGGGLVNATAAMQLENDTLTNGATAASYTFDHEDWELQPGLPVYSGRVVYQGGKLVPGNYT----------SW

>MGG_03870.t1

YIVFNGWPVHMQTVAKLHGFTGKGMKIALIDSGDYTHPLLGGCFGGCKVSFGYDLVPDPMD-CHGTHVAGIVGGVAPDAELGAYKALNCEMLIAFNMAYEAGADIISSSTGSSAVSRIVVPVVIAAGNGEQGWRPSSPSAGRGVTSVASVDNDACYL--GASYWSSWGPDLFATPNVAAPGGIVSTYPGTSMACPAGALALSK-HSVLQGGPGLLQVFDAGLLSNDTLKNGKREATYELGHRPARVPAGQPFYSGFIQYQGGLMPEGKYKIAVAALRLFGDW

>MGG_03316.t1

FVVFNGWPVHIDTIDMLHGIKGNGKRVAIIDSGDWKHPALGGCFGGCVIEGGWDFVEDPYDNCHGTHVTGIIVGAAPGAKIRMYRAWNCEIYIAFLRAFDEGADIISLSAGAMVASRIAVPVVVAVGNGGAGFYTLNPAAGRSVLGVGSVRNDACVLLRGGSTFTTWGPTLSLKPSVAAPGGIFGTFPGTSMACPAGAFALAK-APVAQQGAGIIRAFDATTLSNDTIENGSDEATYALGHTRATVPAGLPIYGGYVPYLGGKLPAGSYKIVLTALRVFGDW

>MGG_02649.t1

YIVFQGWRNYSATVDRLHGILGQGVKVGFIDTGAYRHPALGGGFGGFKVAGGWDFVPDPD-DQHGTHVAGIVAGVAPNATIFAYKVMGTETLVALLRAYDDGMDVITISISAEVASRIVTVVTMSADNGDRGFWIGDGASGRNVIAVASVDADGCPLIRGASIFTSWGPLLTPKPEIAAPGGIFSTYLGTSMATPAATAALGVAASVAQVGNGLVNAYKVTVVDKDTVRNGSSTVTYTVSMSAAVLQAGLPTYNGKVPYLGGKLAPGNYTMRFAALRPFASW

>MGG_02531.t1

FIIFTGWLNHIVTVSKLHGLFGKGVKVGVVDTGWYDHPALGGGFGGFKVAGGWDFVPDPI-DSHGTHVAGIVAGVAPEAELYAYKVFSQATLISFLRAYEDGMDIITASIGAEVASRLVVVVTISAGNGAIGFYGSSGSSGRNVIAVASVSTDGCPLVRGANTFTSWGALLQLKPDIAAPGGIFSTWVGTSMACPAGVAALGVAAPPAQVSTGLIDAFKVTQLENDTVTNGSSPVSYKFSSQPATLRPGLPLYGGKVPYMGGKLAEGRYMMRVAVLKPFGNW

>MGG_00282.t1

FIVFTGWPVHKMGVDRLHGITGAGIRIAVVDSGDTSVHGLSE----TKIT--YS-VPDNCSI-HGTHVLGIVG---------MY-----DDLIGFTEAAKRGVDIITCSFGAAT--RIAIYVSLPAGNAGPGFTGVNPATAPVVAATGSVDN---LITSG-TYYSSWGPSRTM-PTYMAPGYILSTFPGTSMATPAGVAALAQPAPILQQGGGLLDAYAATYVNNDTISNGTKELTYNLQHVGATLGPGVSYFGGYVPYTG---PAGDYFWRIKMLRLNGAW

>MAPG_11419T0

FIVFKGWHAHDMTVDRLHGILGKGAKIAVVDTGDYSHRNLGGCFGGCKVAGGYDFVPDPM-DQHGTHVAGIIAGVAPDATLYAYKIFAQETIIAFLRAYEDGVDIISASVGAEVSRRLVLVVVIAASNGQLGWQMSSGASAPDVLAVASVDADTCPLIRGASIFTSWGPNLSLKPDIAAPGGILSTYPGTSMATPAGVAALGRTAPPIQVGTGLVDAVAVTSMSNDTITNGRVDVEYRFSVQDAVVGPGLPVYSGKVPYFGGRLKPGRYAMRVAVLLPFGNW

>MAPG_10305T0

FVVFAGWPVHHSTVDKLHGILGKGVKVAVIDSGNYRHEALGGGFGGFKVSGGFDLVPDPM-DVHGTHVAGIIAGVAPEAEILSFKVFGADTLVATLMAYEAGADIITASVGATVASRIVVVVTIAAGNGEEGMYASSGSSGRDVLAVAAVDTDGCVLARGASSYSSWGGTLGIKPDVAAPGSIYSTFLGTSMATPAGVAALAESSPVPQSGSGLINATRVTSLANDTITNGTEPVSYTFGLLPAVVQPGLPVYSGKVPYYGGRLAPGRYRMRFAALAPFASW

>MAPG_09314T0

YIVFTGWPVHVMTVNQLRGIRGKGIKIAIVDSGDWKHEALGGCFGGCLVSFGYDLVPDPMD-CHGTHVAGIIAGAATDVTLGMFRVFGCDVLIAFGMAYESGADIISASLGAAVVSRIVVPCTFSAGNGPKGFFSSNPADGHGVTSIASFDNNGCVLIRGASDYTSWGPTGQMKPQFGAPGGILSTYPGTSMACPAAVYALAKPAPVAQQGGGLLQARDATLLSNDTISNADKAITYTLSNLGATIPAGLAVYSGYVPYQGGLLPPGVYKFVVRSLRIFGEY

>MAPG_09140T0

IIVFTGWPMHNYTVDKVHGVYGKGVVVAVVDTGDYTHPALGAGFGGHKVVGGYDLVPDPK-DQHGTHVAGIIAGVAPEATLRAYKVFGGDVLIAFLMAYKDGVDIITASIGAVVASRIVIIVTISASNGVFGFVASSGSSGKNVIAVASSLADACALVRGASDFTSWGPLLQIKPDVTAPGGILSTYPGTSMACPAGVAALGGAAPVAQVGTGMINAAKVTRLTNDTITNGKEPVEYSFQLQPTTVAPGMPLYSGKVPYLGGALPSGKYRMRFAALRPFVSW

>MAPG_06934T0

YILFTGWPVHSHTVDKLHGILGKGARVAVVDTGDYDHPALGGGFGGFKIAGGYDLVPDPF-DTHGTHVAGIIAGVAPEASLYIYKVFAHDTLIAFLMAFQEGADIISCSIGAVVASRLVVVVTIAAGNGYDGVFSSSGAAGKNVIAVASTDPDPCALVRGASEYTSWGTLLESKPDIAAPGAIYAPYVGTSMACPAGVAALGGAAPVPQVGGGQIDALKVTQLTNDTITNGSGPVTYKFALQPATVQPGLPIYSGKIPYTGGKLAPGNYTFRFAALLPLGNW

>MAPG_06002T0

YIVFTGWPNHKATVDRLHGILGKGVKVAIVDSGWYKHPALGGGFGGFKVAGGWDFAPDPL-DTHGTHV------------------MSAATIIAWLRAFDDGADVITTSIGAVVASRIVVVMTMSAGNGAEGFFGGEGGSSEDALAVASVQADACALVRGCSSFSSWAATLLLKPDVAAPGGIFSTWPGTSMAAPAGIAALGRSAPALQMGSGLVNASRVATLDNDTVTNGASTVAYNFTLEPASLAPGIPTYSGKVPYLGGRLAPGNYTMRVAAARPFGGW

>MAPG_04807T0

YMVFHGWPVHVMAVDRLHGFTGRGMRIAVVDTGDYTNAILGGCLGDCIVTHGWDSVPDPMD-CHGTHVAGIISGVAPDARLGAYRVVDCETIAGMLRAFDDGNDILTLSISSLTASRIVVPVFVAIGNGELGFDPVAPADARLVSSVSSYDATGCVL--GGSSFSSWGPTLRSASTFGGPGGIMSTY-GTSMAAPAASAALAK-HPPSQAGAGLIQLWDAGVLSNDSLRNGGSDAIYKLAHTATSVAAGWPIYSGYIPYIGGFLQEGRYAVLVSALRLFADW

>MAPG_03852T0

YILYLGTPVLRMAIDKVHGIKGKGIKVGIIDTGDHRHPALGAGFGGKKIAGGYAWVVQPLTTCHGTHVSGIVGGVAPEAEIFMYRIFDCDYIIAMLRGLQDKVDILSLSLAADIIKKVTVAVIVAIANAGPSYTQEFPSTEPTAIAVGSVANASWFEVSS-DYYSSFGPVYDLKPQISAPGGILSTFPGTSMATPAGCYALAKP----QQGAGLVDVHAATRLSADNLGNGKSPKTYKLSHRGASVAPGLPVFGGYVPYSGG-LEPGDYRWLLSVLRWGGDW

>MAPG_03201T0

YIAFKGWSVHVQTVDRLLGFTGKGLRIGIVDTGDYTHTALGGCFGGCLVEYGADLVPDPMDSCHGTHVAGIIAGAAPGAKLGIYRVFGCDVILATLRAFEDGSDIITGSVGSLVVSRIVVPCTFAMGNGAEGFGVSAPASGPGVMGITSFENDS-------STFVLWGSTLTMKPQFGAPGGIISTLPGTSMATPASIVALAKPSPVPKQGAGIMQAYDAAVLSMDKVTNGSSAVTYNLSHVAATVEPNLPFYSGWVPYFGGRLPAGRYSLVTLALRINGDY

>lbic_232257

FIVFVGRPVHILTVDKLHGITGKGIKIGIIDTGDYTHPLLGGNFGGNKVIGGYDFVPDPLDQCHGTHVAGIIGGVAYDSSLSAYRVFGCDIIVALLRGVKDGQDILTLSLGSVVASRIAKVVTIAAGNGASGWYSSSPGNGVDVISVSSVDNDACVIVRGASSFTSYGPSFYFKPALAAPGGILSTLPGTSMATPAGSAALAKRQTVSQQGAGLINVFDATVVENDTVKNGNASKKYKLTHFPA-LRPGYPLYSGFITYIG---PSGSYRVLIRALRVTGDY

>KFG87255

YIVFHGWPVHLMTVDKLHGFSGKGIKIAVVDTGDYTHPALGGCFGGCRVAFGDNF--KPMD-CHGTQVAGVLAGAAPNATLMAYRVLDCDDMMGWLKAYEDRAQIIVSSAGAMVASRIAVTCIGGLGNQEQGFYAMAPATGDGVISVNSVAS---------PYLSAYGPTLRIKPNVMAPGQIWVT--GTSYATPGGIAALAKP--VAQQGGGLIDAWEATLVENDTITNAKSEVSYELSNLAATLGPGLPLWSGWVPYLGGAYPPGRYKIIARALAVFGDW

>KFG82765

FIFFRGWPVHFMTIDKLHRYTGKGVHVAVIDTGDYKHPSLGGCFGGCLVTKGFDLVPDPMD-CHGSHVAGIIAGGAPGVTLGAYRVFGCDVIIAINRAYLDDADIITMSIGAVTASRIVVIVTISAGNGSQGFFASSGSSGEGVAAIASYDNDLCVLIRGASAFSSWGPTMDVKPQFGAPGGILSTYPGTSMACPAAIYALSKPAPVAQQGAGLIQAHDATLLENETLTNGDSIVTYEISYVPTTLAKGLPVWSGYVPYQGGRLREGTYKIIVRMLRLYGAW

>KFG82316

YIVFTGWPVHHWTVDKLHGMRGKGVKVAVVDTGDYSHLALGGCFGGCKVAGGYDLVPDPM-DYHGTHVAGIIAGVAPGAELLIFKVF-SDVLIAFCDAYTAGADVITASVNALVASRIVVFVSIAAGNGTRGFYSGVGSNGRHVVSVAA------------AYFTSWGPTLIMKPDIGAPGYILSTYLGSSMAAPAGIAALGRNAPPFQVGTGLVDAWKVTQVSLDTITNANQTVKYTFEHESLTLHSGLPLYSGKIPYGGGKLSPGNYT------------

>KFG81532

LIIFRGWPVLAMTIDKLHGFTGNGIRAAIVDTGNYTHEAFGACVGNCRIVTGDNF--KPMD-CHGTALAGILGGVAPNATLMAYRILDCDSAMGWEKAALDGAQIIVSAFGAMMVSRIAIICVGPAGNPENGFNSVSPSAGRGVISVGSFSR---------QDQSSYGPTLDIKPSLGAPGAVPTP--GTSIASPAGVAAVAKP--VAQQGGGLISAWDATLVQNDTITNAQSEVTYRLSPLHATLQPGLPIWSGWIPFLGGEIPPSRYRVALSTLAPFGDW

>KFG81454

FLLFYGWPVHIMTIDKLHGFKGSGIQIAVVDTGDYTNPALGGCFGGCRVALGDNF--DPMD-CHGTAVAGIVAGVAPNATLAGYRVLNCDDLVGWVKAYEDGAQIILSSAGAVVVSRIVVPCIVGLGNRDSGFSTLNPSSGRGVTSVNSFAR---------ARFSTFGPNLEIKPTVGAPGDVPGI--GTSYAGPAGMLALAVP--VAQQGGGLARAWDATLVENDTITNAKVNVTYHLDTLAAVLGPNLPVWSGWIPYLGGRLPVGEYKLAVRALRLFGDW

>KFG80029

YIFFKGYPVHVMTVDKLRGITGKGVKIAVVDTGDYKHPALGGCFGGCLVAFGTDLVPDPMD-CHGSHVAGIVAGAAPGATLGAYRVFGCDVLIAFNQAYQDGANIITASIGAEAVSRIVVPCTVSAGNGAEGFYASTAANGRRVSAIASYDNDGCVLIRGASTFTSWGPTMDTKPQFGAVGGVLSTYPGTSMSCPAGIIALANPAPVPQQGGGLVQAYDATLLSNDTLQNDRKEITYKITHTPATLNGGLALWSGYIPYQGGRLPPGKYKFVVRALRIFGDW

>KFG79636

YIVFKGWPIHVMTVDKLRGVTGKGIKVAIIDTGDYTHPALGNCFGGCLVSFGTDLVPDPMD-CHGSHVAGIVAGAAPNVTLGAYRVFGCDVLIAFNKAFEDGAQIISASIVAVAVSRIVVPCAISAGNGDHGFYISTAANGKGVTAVASYDNDACVLVRGASLFSSWGPTMDFKPQIGAPGGILSTYPGTSMSCPAAIIALANPAPAAQQGGGMVQAYDASLLSNDTITNGTNPATYRLGQVSSTVAPGLALWSGYIPYQGGKLPAGKYQFVTRALRIYGDW

>KFG78468

YFVYKAHQVHVMMVDKLRGITGKGIKIGMIDTGDYNHPALGGCFGGCLFSFGADLV--PMD-CHGTNAAGIIGGAAPGAQLGMYRI-TCDVMVAIYRALADGVDIISSSAGSSAATRAVVVFVQGAGNGTLGFSHLDPAVGNGVISVGSVNSHACVLMNGASDFSSWGPSLGLKPSLTAVGGIISTDWGTSFSGPAALVALSNPAPVAQQGGGLARAYDATLVQNDTIKNGQGAITYRLSHVPATVDPGMPLWSGWIPYQGGQLPEGYYKLVVRALRIFGDW

>KFG77777

LILFHGWPVHLMTVDKLHGYLGSGIKIAVIDTGDYNHPALGGCFGGCRVVTGENF--RPID-CHGTIVAGILAGAAPNATIMAYRVVNCDDMIGWLKAKQDGAQIMVSSVGAVVAARIVVPCIVALGNKDHGFYALNPSTGRGVTSVNSFGR---ALIRGAAPMSAYGPTMDIKPTIGAPGHVPVT--GTSFAGPAGVFALGAP--VAQQGGGLLRAWEATLVENDTITNAKTEVTYRLSHLAATLGPGLPLWSGWVPYLGGAFPPGRYKIVARALSIMGHW

>Jan02995

FIIFVGRPVRGLDVDKLHGITGKGVKVAVIDSGDYNHPNLGEGFGGFKVAGGHDFVPDPMDECHGSHVAGIIAGVAPDATLYAYRVFGCTLIIAMLRAVKDGVDVINISIGSVVASRIAKVVVISAGNGMSGFYTSGPANGIDVISVGSVENDACVLVRNASNFSSYGPTFYFKPALAAPGGILSTVPGTSMSAPAGSAALASYQTAIQQGAGLVNVHKATLVSNDTLNNGDTERTYSVKHVPA-LAPGFPIYSGFVSYIG---PPGDYRLLLRALKVTGDY

>Jan02994

FIIFVGHPAHLLTVDKLHGILGQGIKIAVLDTGDYTHPFLGGGIGGKKIIGGFDFVPDPLDQCHGTHVAGIIGGVAPEAEQFAYRIFGCDVIIALFRGVDDGADILSLSLGAVVASRIAKIITIAAGNGSRGWYAASPSSAINAISVGSLENDACTIVRGGSTFSTYGPTFYFKPAISAPGGILSAYPGTSMATPAGAAALAMTETVTQTGSGLINVYNATTVTNDTIANGDAPKTYKLSHTAA-VAPGYPVYSGFITYIG---PNGSYRLLVRALRVTGDY

>FVET_05568

YIFFKGWPVHVMTIDKLRGITGKGLKVALVDSGDYKHPALGGCFGDCLVSFGTDLVPDPMD-CHGTHVAGILAGAAPGVQIGAYRAFGCDVLIAFNQAFEDGADIISASIGAVAVSRIVVPCVLAAGNGAMGFYASTAANGKKVTAVGSYDNDGCVLLRGASTFSSWGPTMDVKPQFGAPGGILSTYPGTSMASPAGIFALANPAPVPQQGGGIVQAHDAVLLSNDTVKNGKKQIDLQISHVPATIDAGLPVWSGYVPYQGGELPAGQYVVRYRALRIFGDW

>fsol_869

---FYGWPNHNATVWALHGIKGKGVKIAVVDTGDYTHPDLGGGFGGFKVAGGHDFAPDPM-GLHGTHVAGIVAGVAPEATIYAYKTY-AAINIAFLAAYEAGVDIITASIGEEVATRLAVVITVSVGNGEGGYRALSPSGAKGVLAVSNID-------------TSWGPLLQMKPDVGAPGAIYSTVFGTSMATPAGVAALVRKASVAQVGSGLINATSLTDLMNDTVINSKKPVEYTFTKEDSTLQPGMPLYSGNIPFVGGKLARGEYVLRFAVLVPFGDW

>fsol_84201

FIVFQAWPVHEVTVDELHGIKGKGQRVCVVDSGDASHPVLSGRIA-----GGKNM-----QDCHGTFVSSVIVGVAPEAEVYMYKVFGCDIVLGMLAADADDCDIVSLSLGSRVASE-IRLVIIAAGNGEQGFYASSPASGRGVVSVASVNSDSCVLIKGFSAFSSWGLTNDFSPSIAAPGGVYGAFPGTSFSTPSGLAALAMGASLAQQGAGLVD---AVLVSNDTLRNGSEAVTYQVSHVAATIAAGGALWSGKIPYMG---------------------

>fsol_78688

---FSGWAAHYSTVDKLHGIRGKGATVAIIDTGDYTHKALGGCFGGCKIRGGYDLVPDPM-DYHGTHVAGIIAGVAPDAELLIYK----ETIMALCDAYSAGADIITSSIGAVLASRLVIVVVASAGNGEIGFYASSGATGHGVLAVAA------------VYFTTWGPTLLIKPDITAPGFIVSTVLGTSMAAPAGLAALGSSASPFQVGTGLVDAWKVTQLDRDTITNDKKGHRYTFKLEPQFVQPGLPLYSGKVPYGGGKLAPGNYTMRFAALRPYGHW

>fsol_764

---FQGWPAHNDTVAAMHGHFGEDVVIAIVDSGDYTHPAFGGGFGGYRVEAGYDLVPDPM-DCHGTHVAGIAAGVAPKARLRAYKVFGCDTIVAFIKAYEDGADVINASLGALIASTIAVLVVFAAGNGATGFYTSSGGNGVGSLAVGSVQA---MVIPGKNDFSSWGPTARMKPEISAPGGILSTWPGTSMASPAGVGALGRLASIVRQGAGIVDAASVTSVSNDTLTNNDETVTYKVTHE--TLGAGLPVYGGRITYMG--FPDGEYRLLGRAVRTFGDW

>fsol_52284

FIIFKGWQVHHMTVDKLHGVLGKGAVVAVVDTGAYNHPAVSRRFGGFKIEGGYDFVPDPM-DNHGTHVSGIIAGVAPDATLRVYKVFAKDVLIAFLKAYDDGADIITASIGAVVASRIVVVVTISAGNGQAGFAASTGSSGAHVLAISSIDGEACVLVRGASYFTSLGATLFIKPDVAAPGGILSSYLGTSMACPAGIAALGEAASVAQVGTGIVNATKVTSLSNDTITNGDKATTYDLSVENYTIQPGLPLYSGKIPYMGGRLAEGRYKMRFAALKPFAGW

>fsol_46676

FIVFNGWPVHVMTVDKLRGVTGKGIRVAIIDSGDYTHPALGGCFGGCLVEAGWDFTPDPMDDCHGTHVAGTVAGAAPGVKLAAYRAWGCEILLAFIRAFEEGADIISCSDGSVLATRIVVPVVISAGNGGLGFYASAPATGRGVTGVGAVTNNACVLLEGGGDLSSWGPTLAMNPQIAAPGAILSTFPGTSMSAPAGIYALAK-APAPQQGAGIAQAFDAVELSNDTVKNGASDVALQLSHRKANVPAGLPVYSGHIPYLGGLLDEGVYSLRVSALRVFGDW

>fsol_39895

FIVFHGWPVHMITVDKLRGYTGNGIRIAVVDSGDYTHPALGGCFGGCLIAYGRDLVPDPFDDCHGTHVAGTIAGAAPDVTLGMYRAWGCDIMLAFNAAFEDGSDIISYSAGAIAASRIAVSIMVAPGNGSSGFLTASPATGVNVNAIGSVQNDACVLVRGGSISSSWGPTGESKPQFTAPGGILSTYPGTSMATPAAIFALSK-APVAQQGSGLIQAYDATVLSNDTIKNGDKDVTYKLGHKKATVPAGLPVYSGLIPYMGGQLPEGRYKVVFSALRVFGDW

>fsol_39203

YIFFKGWPVHIMTVDKLRGATGKGIKVALVDSGDYTHPALGGCFGGCLVSFGTDLVPDPQD-CHGTHTAGTLAGAAPGVTIGAYRAFGCDILIAFNRAFEDGADIISASIGAVAVSRIVVPCILSAGNGEAGFYANTAGNGKHVTAVASFDNDACVLIRGASAFTSWGPTMDVKPQFGAPGGILSTYPGTSMATPAGIVALSKPAPVPQQGSGIVQAHDAIILSNDTLKNGSEEVEFQISHVPASISPGLPVWSGYVPYQGGELPAGMYVVRYRALRIFGDW

>FOXT_14564

YIFFKGWPVHVQVVDKLRGITGHGIKVAVVDTG---HPALGGCFGNCLVSFGTDLVPDPMD-CHGSHVAGIVAGAAPGVTLGAYRVFGCDVLIAFNQAYQDGADIITASIGAVAVSRIVVPCTVSAGNGDVGFYASTAANGNKVMAIASYDNGGCVLVRGASTYSSWGPTLDVKPQFGSPGGILSTYPGTSMACPAGVIALSNPAPVPQQGGGLIQAYDALLLSNDTLHNGKTDLDLSISHIPTSVGAGLALWSGYIPYQGGKLPAGKYKIAYRALRIFGDW

>FOXT_02380

YIFFKGWPVHVMTIDKLRGVTGKGLKVALVDSGDYKHPALGGCFGNCLVSFGTDLVPDPMD-CHGTHVAGILAGAAPGVQIGAYRAFGCDVLIAFNQAFEDGADIISASIGAVAVSRIVVPCVLAAGNGATGFYASTAANGKKVTAVGSFDNDGCVLLRGASTFSSWGPTMDVKPQFGAPGGILSTYPGTSMASPAGIVALANPAPVPQQGGGIVQAHDAVLLSNDTVKNGKKQIDLQISHVPATIDAGLPVWSGYVPYQGGELPAGQYVVRYRALRIFGDW

>FGST_12951

YIFFKGWPVHVMTVDKLRGITGHGIKVAVIDTGDYKHPALGGCFGDCLVSFGTDLVPDPMD-CHGTHVAGIVAGTAPGVTLGAYRVFGCDVLIAYNQAYQDGADIITASIGAVAVSRIVVPCPVSAGNGDVGFYASTAANGNKVMAIASYDNGGCVLVRGASTYSSWGPTMDVKPQFGSPGGILSTYPGTSMACPAATIALSNPAPVPQQGGGMIQAYDAVLMDNDTIHNGKKEIDLSVSHIPTSVGAGLALWSGYIPYQGGKLPAGKYKVKYQALRIFGDW

>FGST_07502

YIFFKGWPVHVMTIDKLRGVTGKGLKVALVDSGDYKHPALGGCFGKCLVSFGTDLVPDPMD-CHGTHVAGILAGAAPGVKLGSYRAFGCDILIAFNQAYEDGADIISASIGAVAVSRIVVPCVLAAGNGSAGFYASTAANGKKVSAVGSFDNDGCVLLRGASAFSSWGPTMDVKPQFGAPGGILSTYPGTSMASPAGIVALANPAPVPQQGGGLVQAYDAVLLSNDTLKNGKKQIDLQISHVPATIDAGLPVWSGYVPYQGGELPAGQYVVRYKALRIFGDW

>FGST_07235

------WPVHHWTVDKLHGIRGKGATVAIVDTGDYTHKALGGCFGGCKVKGGYDLVPDPM-DYHGTHVAGIIAGVAPDAELLIYKVF-SVTIMALCDAYNAGADVITSSIGAVLASRLVVVMIASAGNGEFGFYSSSGAIGHGVLAVAA------------VYFTTWGPTLLLKPDIAAPGFITSTVLGTSMSAPAGIAALGKSSPPFQVGTGLVDAIKVTQLERDTITNGGRVHQYKFKLEPQFIEPGLPLYGGKVPYGGGKLVPGNYT------------

>EXV06538

YIVFHGWPVHLMTVDKLHGFSGKGIKIAVVDTGDYTHPALGGCFGGCRVAFGDNF--KPMD-CHGTQVAGVLAGAAPNATLMAYRVLDCDDMMGWLKAYEDRAQIIVSSTGAMVASRIAVTCIGGLGNQEQGFYAMAPATGDGVISVNSVAS---------PYLSAYGPTLRIKPNVIAPGQIWVT--GTSYATPGGIVALAKP--VAQQGGGLIDAWEATLVENDTITNAKSEVSYELSNLAATLGPGLPLWSGWVPYLGGAYPPGRYKIIARALAVFGDW

>EXV06306

YIVFTGWPVHHWTVDKLHGMRGKGVKVAVVDTGDYSHLALGGCFGGCKVAGGYDLVPDPM-DYHGTHVAGIIAGVAPGAELLIFK----DVLIAFCDAYTAGADVITASVNALVASRIVVFVSIAAGNGTRGFYSGVGSNGRHVVSVAA------------AYFTSWGPTLIMKPDIGAPGYILSTYLGSSMAAPAGIAALGRNAPPFQVGTGLVDAWKITQVSLDTITNANQTVKYTFEHESLTLHSGLPLYSGKIPYGGGKLSPGNYT------------

>EXV00396

YFVYKAHQVHVMMVDKLRGITGKGIKIGMIDTGDYNHPALGGCFGGCLFSFGADLV--PMD-CHGTNAAGIIGGAAPGAQLGMYRI-TCDVMVAIYRALADGVDIISSSAGSSAATRAVVVFVQGAGNGTLGFSHLDPAVGNGVISVGSVNSHACVLLNGASDFSSWGPSLGLEPSLTAVGGIISTDFGTSFSGPSAIVALSNPAPVAQQGGGLARAYDATLVQNDTIKNGQGAITYRLSHVPATVDPGMPLWSGWIPYQGGQLPEGYYKLVVRALRIFGDW

>EXU99232

FLLFYGWQVHVMTIDKLHGFTGSGIRIAVVDSGDYTHPALGGCFGGCRVALGGNF--DPMD-CHGTAVAGIVAGVAPNATLAGYRVLDCDDLIGWVKAYQDGAQIIVSSAGAAVVSRIVVPCIVGLGNNNSGFNTLNPSSGRGVTSVNAFAR---------AQSSTFGPNLEIKPTVGAPGDVPGI--GTSFAGPAGILALAVP--VAQQGGGLARAWDATLIENDTITNARVKVTYHLDTLAAVLGPGLPVWSGWIPYLGGRLPAGKYNLVVRALRLFGDW

>EXU99167

HVVFEGWLTHLMTVDKLHGYTGKGVTIAVVDTGDYTHPALGGCFGGCRVAKGANLI-RPMD-THGTQVAGIIAGALPNATIHAYASESSDTMIAWLEAYKDGAQVIVSSLGALVVSRITIPCIVALGNR-LGFDAAAPGTGRGAVAVNSFSR---ALIRGAADLSSYGPTLDIKPNVLAPGEVRST--GTSHAAPAGIYALAEP--VAQQGAGLVKAWEATLVSNDTIKNANIAVQYQLTHISATLSSGLPLWSGWIPYLGGSLPPGRYKLMARALSILGDW

>EXU97954

YIVFKGWPIHVMTVDKLRGVTGKGIKVAIIDTGDYTHPALGNCFGGCLVSFGTDLVPDPMD-CHGSHVAGIVAGAAPNVTLGAYRVFGCDILIAFNKAFEDGAQIISASIVAVAVSRIVVPCAISAGNGDHGFYISTAANGKGVTAVASYDNDACVLVRGASLFSSWGPTMDFKPQIGAPGGILSTYPGTSMSCPAAIITLANPAPAAQQGGGMVQAYDASLLSNDTITNGTNPATYRLGQVSSTVAPGLALWSGYIPYQGGKLPAGKYQFVTRALRIYGDW

>EXU97623

YIFFKGYPVHVMTVDKLRGITGKGVKIAVVDTGDYKHPALGGCFGGCLVAFGTDLVPDPMD-CHGSHVAGIVAGAAPGATLGAYRVFGCDVLIAFNQAYQDGANIITASIGAEAVSRIVVPCTVSAGNGAEGFYASTAANGRRVSAIASYDNDGCVLIRGASTFTSWGPTMDTKPQFGAVGGVLSTYPGTSMSCPAGIIALANPAPVPQQGGGLVQAYDATLLSNDTLQNDSKGITYKITHTPATLNGGLALWSGYIPYQGGRLPPGKYKFVVRALRIFGDW

>EXU97232

LILFHGWPVHLMTVDKLHGYLGSGIKIAVIDTGDYNHPALGGCFGGCRVVTGENF--RPID-CHGTIVAGILAGAAPNATIMAYRVLNCDDMIGWLQAEKDGAQIIVSSTGAVVAARIVVPCVVGLGNKEQGFYAMNPSTGRGVTSVNSFGR---ALIRGAAPMSAYGPTMDIKPTIGAPGHVPVT--GTSFAGPAGVFALGAP--VAQQGGGLLRAWEATLVENDTITNAKTEVTYRLSHLAATLGPGLPLWSGWVPYLGGAFPPGRYKIVARALSIMGHW

>EXU96563

YIIFYGWPVHAMTVDMLHGFYGTNITIAVVDTGNYTHPALGGCFGGCRVARGANFV-HPMD-HHGTAVAGVLAGVAPGATLAAYRVVDSDDLIGWLKAVEDGAQIIASSAGAAVVARIAIPCIVGNGNSKKGFFSLDPSTGRNVMAANSFAH---------SGLSASGPTLDLKPNVGVSGDIPCP--GTSFAGPAGMVALAAV--VVQQGGGLAQAWQATLIENDTVTNALFEVTYQLSILPAKLAANLPVWSGWVPYMGGQIPPGDYELEVRALRIFGDW

>EXU96513

LILFRGWRVHIMTVDLLHGFIGSNITIGLVDSG------------GCKVAFGDNL--GPMD-CHGTAVAGVLAGVAPNATLGAYRVLDCDDFMGLIRAFDDGVQIIVSSAVAIIASRIVVPCIIGVGNQDLGFSTSIPSTGRGVIAVTSFGQ---------GSNSASGPTLDIKPNVGAPGRVRLV--GTSFAAPGGIVALAAP--VARQGGGLVQAWEATLVHNDTITNAESEVTYELSNLPARLRPGLPVWSGWIPYQGGSLPPGSYKLVARALAIFGHW

>EXU96296

FIFFRGWPVHFMTIDKLHGYTGKGVHVAVIDTGDYKHPSLGGCFGGCLVTKGFDLVPDPMD-CHGSHVAGIIAGGAPGVTLGAYRVFGCDVIIAINRAYLDNADIITMSIGAVTASRIVVIVTISAGNGSQGFYASSGSSGEGVAAIASYDNDLCVLIRGASAFSSWGPTMDVKPQFGAPGGILSTYPGTSMACPAAIYALSKPAPVAQQGAGLIQAHDATLLENETLTNGDSKVTYEISYVPTTLAKGLPVWSGYVPYQGGRLREGTYRIIVRMLRLYGAW

>cneo_00150T0

YIVFFGRAVHSPSADAVQGNKGKGIKIGIIDGGDYTREPLGGCFGGCKIAGGYDFVPDPYDNCHGTFISGIIGGVAPEASLYVYRVFGCDIVLAMQKAYDDDMDVINLSLGSVFASRVVTVVTVSAGNGQVGFYSSSPAAGKGVINVGSSDSDIYVIVRGTSYFSEIGPTLYFAPSVLAPGTVVGVMPGTSYSSAAGAAALAQQVSVAVQGAGLSN---AAVISNDTIKNGNKWVTYKLSHEPA-LWPGFPIYSGFIPYMG---PNGDYQ------------

>CHGG_03658.t1

YYVFKGYPVHLMTVNRFKAVTGKGIKIGIIDTGDYLHPALGGCFGGCLVSYGTDLVPDPLDECHGTHVAGIIAGAAEGVTLGSYRVFGCDILIAYNMAYEDGSDIITASIGAAVVSRIVVPCVVSAGNGAAGFYASTAANGKRVTAIASVDNNGCVLVRGASDFTSWGPTVEVKPQFSTPGGILSTYPGTSMACPAAIYALARPAPVAQQGAGLLQAWDATLLSNDTISNGATAVTYSLSNIGATIPAGLPVYSGYIPYLGGKLPAGRYKFAVKALRIFGDY

>CAY68779

YIIFNGYPAHGITVDSLHGYTGSGVVIAVIDTGDYTHPALGGGIGNFPIKAGYDL-----MDCHGTFVSSIIVGVAPDAQIVMYKVFPCDIVMGMQKAYDDGHKIISLSLGSLMASR-IRVVLVAAGNGELGFYASSPASGKQVISVGSVQNCVYILLRGYDYYSSQGPALEFFPTISAPGGSWGAWPGTSFACPAGLTALAVKAPLIQQGAGLVN---AVIVSNDTIKNNSETITYQVVHVPG-LGPGAPIIQGKIPYMG---LSGESS------------

>Bfuc_12343.t1

FIIFVGWPVLEMGVDKLHGIKGKGIKIGIVDTGDYRHPALGGGFGGFKITGGYSFVGDPLSTCHGTHVSGILGGVAPEANLYMYRTFDCDTIMGMLKAHEDGVDVISMSLAASVVQSITTAVIVAVGNGSLGFTADYPSSEPGAIAVGAIANSTWFEAFSGDYYSNFGPTYDLKPQISAPGGILSTYPGTSMATPAGCFALAAP----QQGAGLINAYDATTISGDDIDNSGSTKTYTLSHIGATLAGG-PVFGGYIPYIGS-AGDGDYRFFASVLRWGGDY

>ATEG_09889.t1

YIVFNGTPVHQMTVDKLRGYYGSGLRVAVVDSGDYKHPALGGCYGGCLVAFGYDLV--PYDNCHGTHVSGLIAGVAPNVTLGHYKVSNCERLMAFKLAFEAKADIITTSVGGVLLQRIVVPCLVAVGNGFYGFLASNGADGKGATGVGAVNNDACVLIRGAADFSQWGPTLVALPTVVSPGGMLSTYPGTSMATPAGCIALANPDSVAHQGAGLINVYEATILNNDTVNNGSESVEYSIGQVDSTIPPGLPIYSGYVPYLGGELPPGTYSLLVRALKIFGDY

>AOL_s00215g551p

---FNGFPLHVQGVHQLHGLDGQGIRVAVVDTGDYRHPSLGGGFGGFKVEFGTDLVPDPI-DCHGTHVAGILAGVAPKVTLGAYKVFGCEVLIAFCQAVKDKADIISASISSRAVARIVIPCTIAAGNGGEGFYAGAAGDAAGAISVCSVNN-ACVVIRLPSPGNSWGPTLNQRPHICAPGGILSTTRGTSMATPAGVIALGQPSPVAQQGGGMIDAYQFTAVDNDTITNGDAEAFYDLTDI--RIKKGIPVYGGWIPYMGGQVPAGTYQFIYRALRIGGDY

>AOL_s00054g992p

YIVFNGSPVHVQGVNRLHGLDGKGIKIAVIDTGDYTHPSLGGKFGGNKVAFGLDLVPKPI-DCHGTHVAGIIAGVAPEVTLGAYKVFGCDVLIAFLQAQADGADLITASIGAKVVTRIVTPCSIAAGNGAEGFYASGAADAIGAIGVGSVNN-ACVVIRGASVFSSWGPTMYQRPHISAPGGILSTYPGTSMATPAGVIGLGRPAPVAQQGAGIVDAYKFTSVDNDTITNGDKQATYKLTEI--NIKPGIPVYGGWIPYMGGRIPAGTYKLVFRALKMTGDY

>ACN30271

---FNGWTVHVMTVDKLRGLTGDGYKIALVDTGDYTHPDLGGCFGHCIVSFGRDFVPGPKD-CHGTHLAGIVAGAAPGVKLGAYRIFGCDLVVALNKAFEDGAHIIATTAEAVVASRIVVPVIASAGNGDSGFYAASPASAKGVIAVASYDSDACVLVRGASSYTSWGPTMDSKPQLGAPGGIISTWAGTSAASPAAIIALAKPAPAAQQGGGIVQAYDASLLYNDTISNGKKSVTYKICHVPATLAPGLPLWSGYIPYQGGKLPAGKYKFVTRALKISGDW

>ACN30268

YIIFTGWPVHQWTVHELHGFKGKGTKVAIVDTGDYSHQALGGCFGGCKIAGGYDLVPDPM-DYHGTHVAGIIA---------------QDVLIAFCDAYSAGADVITASINALVASRIAVFVSIAAGNGEIGFYSGVGSNGRHVVSVAA------------AYFTSWGPTLILKPDIGAPGYIISTYLGSSMAAPAGIAALGRNAPPFQVGAGLVDARKVTQLSLDTLTNANRTVLYTFQHEPLRIEAGLPLYSGKIPYGGGKLAPGNYSMRIAALRPYGHW

>ACB30121

FIFFSGWPVHVMTIDKLRGYTGKGVHVAVIDTGDYGHPGLGACYGGCLVTNGYDLVPDPMD-CHGTHVAGIVAGGSPGVTLGAYRVFGCDVLIAMNRAYIDGANIITMSIGAVAASRIVVIVTISAGNGKQGFYASSGSSGENVAAIAAYQNDLCVLVRGASNFTSWGPTLDVKPQFGAPGGILSTFPGTSMACPASIYALSNPAPVPQQGGGMVQVYDATLLENETLTNGKGDVTYEISHVPATLKKGLPVWSGYVPYQGGKLPAGTYKIVVKALRLYGDW

>ACB30119

YIFFKAYPVHVMTVDKLRGITGKRVKIALVDTGDFGHPALGGCFGGCLVSFGTDLVPDPKD-CHGTHVAGIIAGTAPDATLGIYRVFGCEILIAFNMAYQGGANIITASVGAEAVSRIVVPCVLSAGNGDQGFYASAAADGHRVAAIAAFDNDGCVLIRGASSTTSWGPTMDLKPQFGAVGGILSTFPGTSMACPAGIMALSNPAPVPQQGAGLIQAYDATVLSNDTLKNDKKRATYRVTHVPTTLRGGLALWSGYIPYQGGRLPPGKYQFVVRALRIFGDW
