# Supplementary material for: Phylogenomic evolutionary surveys of subtilase superfamily genes in fungi
Source: Sci Rep. 2017 Mar 30;7:45456. doi: 10.1038/srep45456 (PMC5371821; doi:10.1038/srep45456)
Supplement: Supplementary Data S9 [file srep45456-s9.docx]

**Phylogenomic evolutionary surveys of subtilase superfamily genes in fungi**

Juan Li*, Fei Gu, Runian Wu, JinKui Yang and Ke-Qin Zhang*

*State Key Laboratory for Conservation and Utilization of Bio-Resources in Yunnan*, *Yunnan University*, *Kunming*, *650091*, *P.R. China.*

* Corresponding author: Juan Li and Ke-Qin Zhang

Tel: 86-871-65033805; Fax: +86-871-65034838.

E-mail address: [juanli@ynu.edu.cn](mailto:juanli@ynu.edu.cn) (Juan Li); kqzhang@ynu.edu.cn(Ke-Qin Zhang)

**Supplementary data S9: 84 kexin amino acid sequences in fungi.**

　　For kexin genes, MUSCLE v3.5 was used to generate protein alignment with default settings [^20^](#_ENREF_20). The ambiguous areas of alignment were located and removed by using the program Gblocks 0.91b [^21^](#_ENREF_21)^,^[^22^](#_ENREF_22) with default parameters. The gap selection criterion “with half” was used here. Finally, an alignment consisting of 465-bp amino acids from 84 kexin genes were obtained from Gblocks 0.91b.

>ACB30122.1|_kexinlike_protease

RDYDYTPGSRVGEGPLGSLSDHHVFRKRIKDPLYKRQWHLHNTVQLGHDVNVTGVWLEGITGKKPTVAIIDDGLDMNSLDLKDNYFAEGSYDFNDGDAIPAPELSDDRHGTRCAGEVAAVNDVCGLGVAYESKIAGIRILSKPISDADEAEAMMYKYDKNQIYSCSWGPRDDGRTMEAPGVLIRRAMLKSIQEGRDGLGSIYVFASGNGAASDDNCNFDGYTNSIFSITVGAVDRAGQHPYYSEHCSAQLVVTYSSGSGIHTTDVCASGHGGTSAAAPLAAGIFALVLEVRPDLGWRDMQYLAMDTAKLHAGWQQTAIGFSHVFGYGKIDSYDLVQKAKWKKVKPQAWFFSVSFDVTEDMLSKANERLEHVTVTMNVNHTRRGDISVDLVSPANVSNIATARKDDNKNVGYVDWTFMTVAHWGEKGVGKWTLVVRDDWHLKLWGEARDASKARRRLRYEFLYDAF

>ACB30127.1|_kexinlike_protease

RSFDIAPARELASGPVGELPDHHVFSRRISDPRFESQWHLMNTIQPGNDLNVSGVWLEGVFGEGVTTAIVDDGLDFHNLDLSPNYYAGGSYDFNDDVPEPLPRLQDDHHGTLCAAEIAAANEICGVGVAYRSRVSGIRMLSGTVDDVDQAAAMNFDYQNNDIYSCSWGPKDDGRHMKAPGVLVQRAIVNGVQRGRGGKGSIYVFSAGNGASQDDNCNFDGYTNSIYSITVGAIDRTGRHALYSESCSAQLVVAWSSGSGIYTTDNCTALHSGTSAAAPLAAGVIALALSVRPDLTWRDVQHLLVEAAVPDGSWQTTKTGYSHDWGYGKIDAYALVQAARWKLVKPQAWLHAGSYTVTSEALGGANARLEHVTVTINVRHARRGDLSVELVSPSGVSYLSTPRLPDDAETGYVDWEFMSVAHWGETGEGTWRIIVKDNWRLNLWGEAID------------M----

>ACLA_051950.t1

RSYEFSPAQLLGEGQIGELANHHTFSKRIADPIFGDQWHLFNAVQLGHDLNVTGVWMEGITGKGVTTAVVDDGLDMYSNDLKPNYFAEGSYDFNDHTPEPRPRLTDDKHGTRCAGEIAAANDVCGVGVAYDSRIAGVRILSKAIDDADEAKAINFAYQENDIFSCSWGPPDDGATMEAPGVLIKRALVNGVQNGRGGKGSIFVFAAGNGASFDDNCNFDGYTNSIYSITVGAIDREGKHPSYSESCSAQLVVAYSSGSSIHTTDVCYSFHGGTSAAGPLAAGTVALALSARPELTWRDAQYLLVETSVPDGSWQVTKSGFSHDWGYGKVDAYSLVQKAKWELVKPQAWYHSSSHEVTEEMMKTANARLEHVTVTMNVNHTRRGDLSVELRSPDGVSHLSTTRRSDNQKAGYVDWTFMTVAHWGESGIGKWTVIVKDDWRLNLWGEAIDGTNQRKRIRYDFLYNAF

>AFL2T_10381

RSYDFSPAQRLGEGQVGELTQHHTFSKRITDPIFGGQWHLYNTVQVGHDLNVSDVWLEGITGKGVITAVVDDGLDMYSNDLKPNYFAEGSYDFNDHVPEPRPRLGDDRHGTRCAGEIGAANDVCGVGVAYDSQVAGIRILSAPIDDADEAAAINYGFQRNDIYSCSWGPPDDGATMEAPGILIKRAMVNGIQNGRGGKGSIFVFAAGNGAGYDDNCNFDGYTNSIYSITVGAIDREGKHPSYSESCSAQLVVAYSSGSSIHTTDVCYSLHGGTSAAGPLAAGTIALALSARPELTWRDAQYLMIETAVPDGSWQTTKMGFSHDWGFGKVDAYSLVQLAKWELVKPQAWFHSSSYEITKDMMYQANEKLEHVTVTMNVNHTRRGDISVELRSPEGVSHLSTARRSDNAKAGYEDWTFMTVAHWGESGVGKWTVIVKDDWRLNLWGLSIDGFSQRKRLRYDFLYNAF

>AFUA_4G12970.t1

RSYEFSPAQLLGEGQIGELANHHTFSKRITDPIFNGQWHLFNTVQLGHDLNVTGVWMEGITGKGVTTAVVDDGLDMYSNDLKPNYFPEGSYDFNDHTPEPRPRLSDDKHGTRCAGEIAAANDVCGVGVAYDSRVAGVRILSKAIDDADEATAINFAYQENDIFSCSWGPPDDGATMEGPGILIKRAFVNGVQNGRGGKGSIFVFAAGNGASFEDNCNFDGYTNSIYSITVGAIDREGNHPSYSESCSAQLVVAYSSGSGIHTTDVCYSFHGGTSAAGPLAAGTVALALSARPELTWRDAQYLMVETAVPDGSWQVTKAGFSHDWGYGKVDAYALVQKAKWELVKPQAWFHSSSYEVTEQMMKNANARLEHVTVTMNVNHTRRGDLSVELRSPEGVSHLSTTRKSDNEKAGYVDWTFMTVAHWGESGVGRWTVIVKDDWRLNLWGEAIDGANQRKRILYDFLYNAF

>AGOS_ABL203W.t1

KDHEYADEELLEEHAVRGLERHYVLSKRIKDPLFDEQWHLLNTRYPKNDMNVTGLWQKNITGHGIVVAVVDDGLDYESEDLKDNFCAEGSWDFNSNTALPKPMLSDDTHGTRCAGEIAAANQFCGLGVAFNSKVSGIRILSEDITPEDEAASLVYGLDINDIYSCSWGPTDNGEELQAPSDLVKKAIIRGVTEGRDRKGALYVFASGNGGALGDNCNYDGYTNSIYSITVSALDHRGLHPTYAESCSAVLVVAHSSGSGIRTTDVCFDHHGGTSAAAPLAAGVYALLLQVNPNLTWRDVQYLTILTSIEQ-RLQEGSLGYSHKYGYGKLDAYNIVELAKWKNVNPQAWYYHSTTSVSRDALDKANKRVEHVTVTVDIEASIRGFTTVDLIAPNNISHLGVVRKKDKSHAGFRNWTFMSVAHWGYAGEGDWKLQV--GWRLKLFGESIDASKAR--R-FEFMLSD-

>An01g08530.t1

RSYDYSPAQRLGEGPVGELPSHHTFSKRIADPIFGEQWHLYNTVQLGHDLNVTGIWLEGVTGQGVTTAIVDDGLDMYSNDLRPNYFAAGSYDYNDKVPEPRPRLSDDRHGTRCAGEIGAANDVCGVGVAYDSRIAGIRILSAPIDDTDEAAAINYAYQENDIYSCSWGPYDDGATMEAPGTLIKRAMVNGIQNGRGGKGSVFVFAAGNGAIHDDNCNFDGYTNSIYSITVGAIDREGNHPPYSESCSAQLVVAYSSGASIHTTDVCSTTHGGTSAAGPLAAGTVALALSVRPELTWRDVQYLMIEAAVPDGSWQDTKNGFSHDWGYGKVDTYTLVKRAEWDLVKPQAWLHSSSYEVTEDMLKGANERLEHVTVTMNVNHTRRGDLSVELRSPDGVSHLSTPRRPDNQEVGYVDWTFMSVAHWGESGIGKWTVIVKDDWRLNLWGEAIDGAEQRKRIRYDFLYNAF

>AO090009000291.t1

RSYDFSPAQRLGEGQVGELTQHHTFSKRITDPIFGGQWHLYNTVQVGHDLNVSDVWLEGITGKGVITAVVDDGLDMYSNDLKPNYFAEGSYDFNDHVPEPRPRLGDDRHGTRCAGEIGAANDVCGVGVAYDSQVAGIRILSAPIDDADEAAAINYGFQRNDIYSCSWGPPDDGATMEAPGILIKRAMVNGIQNGRGGKGSIFVFAAGNGAGYDDNCNFDGYTNSIYSITVGAIDREGKHPSYSESCSAQLVVAYSSGSSIHTTDVCYSLHGGTSAAGPLAAGTIALALSARPELTWRDAQYLMIETAVPDGSWQTTKMGFSHDWGFGKVDAYSLVQLAKWELVKPQAWFHSSSYEITKDMMYQANEKLEHVTVTMNVNHTRRGDISVELRSPEGVSHLSTARRSDNAKAGYEDWTFMTVAHWGESGVGKWTVIVKDDWRLNLWGLSIDGSSQRKRLRYDFLYNAF

>AOL_s00078g136p

RDYDYSPAKRLGVEPIGRLDDHHLFRKLINDPIFKDQWHLINTREIGHDVNVGRLWLDGIFGENATVAIVDDGLDFKSHDLAENYFKEGSWDFNDPGPDPLPRLSDDRHGTRCAGEVAAANDVCGVGVAYKAKVAGIRILSKSITDADEAVALNYAYEKNNIYSCSWGPPDDGVAMDAPGILIKKAIQQGVQKGRDGKGSIFVFASGNGAANGDNCNFDGYTNSIYSITVGAIDRAGAHPYYSEECSANLVVTYSSGSGIHTTDVCYTMHGGTSAAAPLAAGIFALVVSVRPDLTWRDMQYLCVEAAVPDPDWETTTIGFNHKYGYGKIDAVKLVEAAKWKLVKPQAWFHSTTIEITKEHLENANQRLEHVTVTMDLNHTRRGDLDVDLISPNGVSKIAAQRPKDSSTEGYKEWTFMTVKHWGESGIGKWTIVVKDWWRLNLWGEAINGQKQKKHQAYEFLYDAF

>ATEG_03179.t1

RSYDFSPAHQLGEGPIGELPHHHTFSKRITDPIFTGQWHLYNTVEVGHDLNVTGVWLDGITGNGVTTAVVDDGLDMYSNDLKPNYFPEGSYDFNEGVPEPRPRLRDDKHGTRCAGEIAAANDVCGLGVAYDSRIAGIRILSEPIDDTDEAAAINFGYQQNDIYSCSWGPMDDGKTMEAPGILIKRAMVNGVQKGRGGRGSIFVFAAGNGASFDDNCNFDGYTNSIYSITVGAIDREGNHPSYSESCSAQLVVAYSSGAKIHTTDVCYSYHGGTSAAGPLAAGTVALALSARPELTWRDAQYLMVETAVPDGSWQILKSGFSHDWGFGKVDAYSLVQKAKWELVKPQAWFHSSSYEVTETMMKDANERLEHVTVTMNVNHTRRGDLSVELRSPDGVSHLSTARRPDEENTGYVDWTFMSVAHWGESGVGKWTVIVKDDWRLNLWGEAIDGAKQRRRKRYDFLYNAF

>BDCG_03334T0

RNHDYSPAQLLGEGQVGELADHHTFSKRIEDPIFADQWHLFNTVEVGHDLNVTGLWLEGITGEGVISAIVDDGLDMNSNDLMDNYFAEGSYDYNDKSPVPKPRLFDDKHGTRCAGEIAAVNNVCGVGVAYDSKVSGIRILSKPVTDEDEAASINYKYQHNQIYSCSWGPIDDGATMDAPGILIRRALVNGIQKGRAGRGSIYVFAAGNGAGNEDNCNFDGYTNSIYSVTVGAVDRDDNHPYYSEWCSAQLVVTYSSGANIHTTDVCATRHGGTSAAGPLVAGVVALALSVRPELTWRDVQYILLETAIPDSDWQETSIGFSHEFGYGKVDAYSAVHLAKWKLVKPQAWMHSSSFEVTKKMLDMNNERIEHVTLTMNVNHTRRGDLSVELHSPSGVSLLSTTRKNDDHAVGYVDWTFM-----GESGIGEWTVIVKDDWQLNLWGEAINPKIQRKRLRYEFLYDAF

>Bfuc_B0510_BC1G_01499.t1

RNYDYDPAGRLGDGQLGELEDHHVFSKRIQDPIFKDQWHLYNPVQVGHDVNVTDVWMQNITGTGSIVAIVDDGLDMYSNDLKANYYAEGSYDFNENTLEPKPRLSDDKHGTRCAGEVSAVNDVCGVGVAYDSKIAGIRILSKMITDADEAVAMNYAYQHNQIYSCSWGPPDDGRSMDAPGILIKRAMVNAVQKGRGGLGSIYVFASGNGAANEDNCNFDGYTNSIYSITVGAIDRKGLHPYYSEKCSAQLVVTYSSGSGIHTTDVCSDAHGGTSAAAPLAAGIFALVLQIRPDLSWRDMQYLVMSTALPTGEWQTTTIGFSHTFGYGKIDTWATIEAAKFKNVKPQAWFYSVSFEVTKEMLQEANERLEHVQVTMNIAHTKRGDLSVDLVSPDKVSHLSASRRYDSEPEGYDDWTFMSVVHWGESGIGTWTITVRDDWHLKLWGESIDAEKARKRLRWEFLYDAF

>CAGL0J07546g.t1

KNFDYSHAELSKEHLVRGLDNHYVFSKRINDPLFPKQWHLINPAFPGNDINVKDVWLQNITGKGVVAAIIDDGVDYTSPDLKDNFCKEGSWDFNENQQLPMPLLSDDNHGTRCAGEIAAINNYCGVGVAYDAKVSGIRILSGPLTAEDEAASLVHALDVNDIYSCSWGPTDDGKHLQGPSPLVKKAMKKGVTEGRGNKGAIYVFASGNGGMHGDNCNYDGYTNSIYSITVGAIDHKGLHPPYSESCSAVLVVTYSSGSGIHSTDICYDRHGGTSAAAPIAAGIYALVLEANPNITWRDMQYLSILSSETDGDWQTTKLEYSHKYGYGKLNAHNIVALAKWENVNPQVEFATSTIEITASDLEKAKRSVEHVTINVDISTENRGTTTIDLISPFGVSHLGVVRRKDDSNEGFRDWTFMSVAHWGELGSGEWKLIV--SWSITLFGLSEYSEQKR--RRYDYLSD--

>CAR28945.1|_ZYRO0F15598p_Zygos

KDHRYSEAELQEEHAVRGLDSHIVFSKRIHDPLFEKQWHLINTNYPGNDVNATGLWYENITGHGVVAAIVDDGLDYESEDLKDNFCKEGSWDFNDNTKLPKPRLDDDYHGTRCAGEIAAVNDFCAVGVAFNAKLSGIRILSGEITAEQEAASLIYGLDINDIYSCSWGPADDGRHLQGPTDLVRKALVKGVQEGRDKKGALYVFASGNGGAFGDNCNYDGYTNSIYSITVGALDHKGLHPSYSESCSALMVTTYSSGSGIHTTDICSETHGGTSAAAPLAAGIYALILEANPNLTWRDVQYLSVLSAREDGEWQQGALGYSHKYGYGKIDAYAMAKMATWKNVNPQAWYYTSHFTIKEKDLKNANKRVEHIIVTVDVDTDLRGATTIDLISPSGVSNLGVVRKYDNSNEGFKEWSFMSVAHWGENAVGDWQLRV--NWRLKLFGESIDASKAR--RRYEFMLDT-

>CAR30036.1|_KLTH0H00418p_Lacha

KDHSYSVPRLLKEHEARGLDKHYVFSKRIEDPLFGIQWHLVNGNYPGHDVNVSGLWYENVTGHNVVVAVVDDGLDYESEDLKDNFSAEGSWDFNDNGPMPKPRLSDDYHGTRCAGEIAAVNKACGIGVAYNAKVAGIRILSAEVTAEDEAASLIHALDVNDIYSCSWGPLDDGRVLQGPDDLVRKALVTGVTKGRNEKGALYVFASGNGGMYDDNCNYDGYTNSIYSITVGAIDHKGLHPPYSESCSAVMVVTYSSGSGIHSTDICSDTHGGTSAAAPLAAGVYSLVLEANPNLSWRDVQYLSILSSEEDGEWQEGALGYSHKYGYGKLDAYRIVTMGRWENVGPQSWYYSSSISVSSEQLKNANKRVEHITVTVSIETTIRGRTTINLVSPKGVSKLGVVRRSDTSPEGFQNWTFMSVAHWGEIGEGDWSLHV--DWRLKLFGESLDASKAR--RLYEFMMGE-

>CAX45145.1|_kexin_precursor_pu

RDYNYSQIDFINEHQLSSLDNHYVFSKRIHDPEFTTQWHLINLKYPGHDVNVTGLWLEDILGQGIVTALVDDGVDAESEDIKQNFNSKGSWDFNNNGKSPLPRLFDDYHGTRCAGEIAAVNDVCGIGVAWKSQVSGIRILSGPITSSDEADAMVYGLDTNDIYSCSWGPTDNGKVLSEPELIVKKAMIKGIQQGRDKKGAIYVFASGNGGRFGDSCNFDGYTNSIYSITVGAIDYKGLHPQYSEACSAVMVVTYSSGSGIHTTDICSATHGGTSAAAPLASGIYSLILSANPNLTWRDVQYISVLSATPDGNYQTTALNYSHKYGYGKTDAYKMVHFAKWKNVKPQAWYYSSSVKVTEKDLKIMNERVEHITVKVNIDSTYRGRVGMRIISPTGISDLATFRINDASSRGFQNWTFMSVAHWGETGIGEWKVEV--DWQFRIFGESIDGDKAR--R-YEFLFDDF

>CAY68919.1|_Kex2_proprotein_co

---EI--PTFLEERQINGLDDYHVFSKRIDDPLFAKQWHLFNPRYPGHDVNVSQVWYDGITGKGVVTAIVDDGLDMDSKDLKESFCEEGSWDFNANTRLPKPRLRDDHHGTRCAAEIAAKNKYCGVGVAYDSKVSGIRILSDKITPEDEALSLIYGLDVNDIYSCSWGPADNGITMQGPSSLVKEAMLKGVQDGRKGKGALYVFASGNGASSGDNCNFDGYTNSIYSITVGAIDIKGLHPPYAEACSAVMTVTYSSGSGIHTTDICSDTHGGTSAAAPLAAGLYSLVYQANPDLTWRDIQWLTVLTAVPEPGWQKTAIGYSHKYGYGKIDAYALVNLARFPYLKPQSWIYGSKYELTQEAKDLMNEKIEHVTVTVDIKAAERGKVLVELISPSGVSELAPYRRMDKDKEGFPNWTFMSVAHWGEDGLGEWILKI--SWQIKFFGESQDPEKAKLERRYEFF----

>CHGG_01943.t1

RNWDYSPARSLGEGPLGELRDHHIFAKRINDPIFHKQWHLFNTIQVGHDVNVADVWLQGVTGSNTTVAIVDDGLDMYSDDLKDNYYALGSYDFNDKTDEPKPRLSDDRHGTRCAGEVSAGNNACGLGVAYDSKIAGLRILSKLISDADEAVAMNYDFQHNQIYSCSWGPPDDGKSMDAPGILIRRAMLNAVQNGRQGLGSIYVFASGNGAQNEDNCNFDGYTNSIYSITVGAIDRKGMHPPYSEKCSAGLVVTYSSGGGIHTTDVCSNSHGGTSAAAPLAAGIFALALQVRPDLSWRDMQYLAMNTAVPTGEYQDTTIGFSHTFGYGKLDSSAIVEAARWKKVKPQAWFYTVEFKVTEAMLKEANQRVEHITVTMNVEHGRRGDISVDLISPNKVSHLSVTRKNDESTEGYDDWTFMSVAHWGESGVGTWTIIVRDDWHMKLWGETRDASKARRRLRYEFLYDAF

>CIMG_00625.t1

RDYDYSPAQLLGEGRIGELQDHHTFSKRISDPIFVDQWHLFNTEQPGHDLNVTGLWLEGITGNGTVTAIVDDGLDMYSHDLKDNYFAEGSYDFNDKGKEPRPRLVDDKHGTRCAGEVAAVNDICGVGVAYNGKVAGIRILSKPVTDEDEAAAINYGFQKNQIYSCSWGPVDNGATMDAPGLLIRRAMVHGIQQGRGGKGSIFVFAAGNGAASGDNCNFDGYTNSIYSITVGAIDREDKHPYYSESCSAQLVVTYSSGGTISTTDVCSNRHGGTSAAGPLVVGVVALALDVRPDLTWRDIQYLIVETAIPEPGWQTTAIGFSHDFGYGKVDAYSLVQLAKWELVKPQAWLHSSSFEITEELLKKNNERVEHVTVTMNVNHTRRGDLSVELKSPSGISYLSTTRSGDFEKKGYVDWTFMSVAHWGETGKGKWTVIVKDDWQLSLWGEAIDGKIQQKRRRYEFLYDAF

>CLUT_01869

RDYQYTPDNFIREHPARGLDDHYVFSRRINDPIFTEQWHLINTLSPGNDVNVKDVWYRGVRGRNVTVAVIDDGVDCDSEDLAANFNARGLWDFNDNTELPKPRLFDDYHGTRCAGEIAAVNEVCGIGVAWEAKVAGIRILSGTITAEDEAAAMVYGLDANDIYSCLWGPTDNGQTVAAPDVLVRKALIKGVQQGRDKKGAVYVFASGNGGRVGDQCNFDGYTNSIYSITVGAIDYQGQHPPYSEACSAVMVVTYSSGGRIHTTDICASTHGGTSAAAPLAAGLFALVLSANPALTWRDVQYVCAKAAVPDGEYQVTGLGYLHKYGYGKLDADKLVTVAQWKNVKPQAWYYSSTITVSEEELRGMNERVEHVTVKVNIASNVRGRIGARLVSPKGVSTLAQFRAVDTLHNGLSDWVFMSVAHLGEDGVGDWRLEV--DWQLRIFGESVDASKAR--R-FEFLFEDL

>cneo_grubii_CNAG_05446T0

RSYTYVASKSLGVERIGELDGHWLVRKRLADPMLDQQWHLINTQMKDIELNVTGLWGRGVTGEGVHVVIIDDGLDVESKDLKDNFFAEGSYDFNDHTALPIPRLRDDQHGTRCAGEIAAVNDVCGVGVAYGSKIAGVRILSAPISDADEAAALNYAYQLNDIYSCSWGPPDDGRSMEAPDGLILKAMVNGVQKGRDGKGSVFVFAAGNGGGSDDQCNFDGYTNSIFSVTVGAVDRKGLHPYYSEMCAAMMVVAPSSGSGIHTTDVCAHNHGGTSAAAPLAVGVFALALSVRPDLTWRDIQHLAVRHAVFDPAWELTAAGFSYKYGYGKLDAGLFVEAAEWELVKPQTWYDSSTYEVTQSMLYDANERLEHVTVRVWIDHQRRGDVEVELISPNGVSVLCRQRRFDDANSGFPGWKFMSLKHWDENPVGTWVIKVKDAWSLQLWGESVDPALAA-R-RYE-LYDAF

>CPAG_02963.1

RDYNYSQLDFISEHQLPSLENYYVFSKRIHDPEFASQWHLFNLQYPGHDVNATGLWLEDILGQGIVTAIVDDGLDAESADLKANFNAKGSWDFNDNGPLPLPRLSDDYHGTRCAGEIAAVNDVCGVGVAYKSQVSGVRILSGPITSAEEASALVYGLETNDIYSCSWGPTDNGRTLSEPEIIVKKAMLRGIQEGREGKGAIYVFASGNGGRFSDSCNFDGYTNSIYTITVGAIDHKGMHPLYSEACSAVMVVTYSSGSGIHTTDICSARHGGTSAAAPLASGIFSLILGANPDLTWRDLQYINVLSATPDGNYQTTALNYSHMYGYGKIDAYKMVEFAKWKNVKPQSWHYCSKVTVTEEDLKVMNEKIEHVTVKANIDSSFRGRTGVRLVSPSGVSDLAKFRPLDFSSRGFQDWTFTSIAHWGEDGLGEWTLEV--NWQLRFFGTTIDASKAR--R----LYEDF

>CPAT_02260

RDYDYSPAQLLGEGRIGELQDHHTFSKRISDPIFVDQWHLFNTEQPGHDLNVTGLWLEGITGNGTVTAIVDDGLDMYSHDLKDNYFAEGSYDFNDKGKEPRPRLVDDKHGTRCAGEVAAVNDVCGVGVAYNGKVAGIRILSKPVTDEDEAAAINYGFQKNQIYSCSWGPVDNGATMDAPGLLIRRAMVHGIQQGRGGKGSIFVFAAGNGAASGDNCNFDGYTNSIYSITVGAIDREDKHPYYSESCSAQLVVTYSSGGTISTTDVCSNRHGGTSAAGPLVVGVVALALDVRPDLTWRDIQYLIVETAIPEPGWQTTAIGFSHDFGYGKVDAYSLVQLAKWELVKPQAWLHSSSFEITEELLKKNNERVEHVTVTMNVNHTRRGDLSVELKSPSGISYLSTTRSGDFEKKGYVDWTFMSVAHWGETGKGKWTVIVKDDWQLSLWGEAIDGKIQQKRRRYEFLYDAF

>CTRT_03249

RDYNYSQIDFINEHQLPSLDNYYVFSKRIHDPEFAAQWHLINLKYPGHDVNATGLWLEDILGQGIVTALVDDGVDAESEDIKDNFNADGSWDFNNNGKSPLPRLFDDYHGTRCAGEIAAVNDVCGIGVAWKSQVSGIRILSGPITSADEASAMIYGLDHNDIYSCSWGPTDNGRVLSEPEVIVKKAMIKGIQEGRDKKGALYVFASGNGGRFGDSCNFDGYTNSIYSITVGAIDHKGLHPEYSEACSAVMVVTYSSGSNIHTTDICSATHGGTSAAAPLASGIYSLVLSANPDLTWRDVQYISVLSATPDGNYQVTALNYSHKYGYGKTDAYQMVHFAKWKNVKPQAWYYSSSITVTEKDLKVMNERVEHITVKVNIEANYRGRVGMRIISPTGISDLAAFRRSDASGKGFQNWTFMSVAHWGESGLGEWKVEV--DWQFRIFGESIDADKAR--R-YEFLFDEF

>DEHA0C11308g.t1

RDYNYSSYKFIDEHQVRGLDDHFVFSKRISDPIFQKQWHLVNTFYPGHDVNVTGLWYEGNTGKGIVTAVVDDGLDYESEDLHDNFNSLGSWDFNDNTNLPKPRLFDDYHGTRCAGEIGAVNDVCGVGVAYDSQISGIRILSGTISAEEEASAMMYGLDVNDIYSCSWGPTDDGKTLSQPDAIVKKAMIKGIQTGRKDKGAVYVFASGNGGRYADSCNFDGYTNSIYSITVGAIDYKGLHPMYAEACSAVMVVTYSSGSGIHTTDICSALHGGTSAAAPLAAGIYSLVLHANPNLTWRDVQYVSALSSVPDGNYQITALGYSHKYGYGKIDAYAMAHFAEWKNVKPQAWYYSKKIKITKEDLKIVNERVEHITVTVNIQATERGKVGVRLISPHKTSDLATFRPQDRSGAGFKDWTFMSVAHWGESGIGEWAIEV--DWQLRLFGESIDPEKAR--R-YEFLFDEF

>Enid_FGSC_A4_AN3583.2.t1

RSYDYSPAARLGEGQVGELDGHHTFSKRIEDPIFTKQWHLFNTVQVGHDLNVTGVWLEGITGKGATAAIVDDGLDMYSNDLSPNYFPEGSWDFNDHTAEPRPRLRDDRHGTRCAGEVAAANDVCGVGVAYDSRIAGIRILSGPIDDTDEASAINYAYQENDIYSCSWGPPDDGATMDAPGILVSRAIVNGVQKGRDGKGSIFVFAAGNGAASGDNCNFDGYTNSIYSITVGAIDREGQHPQYSESCSAQLVVAYSSGISISTTDVCYSVHGGTSAAGPLVVGAISLALSVRPELTWRDAQYIVLETAVPDGSWQVTKSGFSHDWGYGKIDVYSLVQKAKWELVKPQAWYHSASWEVTEQMMKDANEKLEHVTVTMNVNHTRRGDLSVELRSPEGVSHLSTPRKNDNAEVGYIDWTFMTVAHWGESGVGTWTVIVKDDWRLNLWGMAVDGAKQRRRLRYDFLYNAF

>EXV04772.1|_peptidase_S8_famil

RDYDYPPASRLHEGTVGALSDHHVFRKRIKDPIFTAQWHLFNSVEVGNDVNVTGVWMEGITGKNATVAIVDDGLDMHSEDLRENYFAEGSYDFNDHDPEPAPVLSDDHHGTRCAGEVAAVNDVCGIGVAYESKVAGIRILSAVISDEDEAEALMYKNDKNQIYSCSWGPSDDGRTMEAPSVLIRRAMLKSIQEGRNKLGSIFVFASGNGAKSGDNCNFDGYTNSIFSITVGAVSRDNQQTYYSEPCSAQLAVTYSSGGSIHTTDVCTDRHGGTSAAAPLAAGIFALVLEVDPELSWRDMQYLVMDTAKPGVVWNQTGIGFSHAFGYGKIDTYDLVQKAKWNKVKPQAWFFSANFTVTKDMLKEANERLEHVTVFMNVNHTRRGDISVDLISPSSVSQIATTRSGDEHYAGYVNWTFMSVAHWGESGVGTWTLVVRDDWRLKLWGESIDAKKAKR--RYEFLYDAF

>FGST_10511

RDYDYTPASRLGEGQLGALDDHHVFRKRIQDPIFKEQWHLLNPLQPGHDVNVTGLWLEGITGKNVTVAVVDDGLDMNSDDLKPNYFAEGSWDFNDNDPEPAPVLDDDRHGTRCAGEVAAANDVCGVGVAYDSKVAGIRILSKLISDADEAEALMYKYHDNHIYSCSWGPSDDGQTMEAPDVVIRRAMLKAIQEGRSGLGSVYVFASGNGAGQGDNCNFDGYTNSIYSITVGAVDRTGLHPYYSEECSAQLVVTYSSGSGI-----CYKAHGGTSAAAPLAAGIFALVLQVRPDLTWRDLQYLAMDTALPEANQQNTTIGFSHTFGYGKIDSWALVEKAKWKLVKPQSWYFSVTLDVTEDMLKDSNARVEHVTVTMNVEHTRRGDLSVDLISPDNVSHLAVSRRSDAKDAGYVDWTFMSVAHWGESGVGKWTIIVRDDWRMKLWGEAIDADKARRRLRYEFLYDAF

>FOXT_05775

RDYDYTPASRLGEGQLGSLDDHHVFRKRIQDPIFKEQWHLLNPTQVGHDVNVTGLWLDGITGKNVTVAVIDDGLDMHSDDLKPNYFAAGSWDFNDNDPEPAPVLDEDRHGTRCAGEVAAANDVCGIGVAYDSKVAGLRILSKLISDADEAEAMMYKYDDNHIYSCSWGPSDDGQTMEAPDVVIRRAMLKAIQKGRRGLGSIYVFASGNGAGQGDNCNFDGYTNSIYSITIGAVDRTGLHPYYAEECSAQLVVTYSSGSGIHTTDVCYKAHGGTSAAAPLAAGIFALALQVRPELTWRDLQYIAMDTAIPESNQQNTTIGFSHVFGYGKIDSWALVERAKWPLVKPQSWYFSVTLEVTEDMLKGSNARVEHITVTMNVEHTRRGDLSVDLISPDNVSHLAVARRGDAKEEGYIDWTFMSVAHWGESGVGKWTIIVRDDWRLKLWGEAIDADKARRRLRYEFLYDAF

>fsol_96635

RDYDYAPANRLGEGQLGALDDHHVFRKRIADPIFTEQWHLYNPIQLGHDVNVTGLWLDGITGQNVTVAVVDDGLDMNSDDLKPNYFAAGSWDFNDNDPVPAPELDDDRHGTRCAGEVAAANDVCGVGVAYDSKVAGLRILSKLISDADEAEALMYKYDDNHIYSCSWGPSDDGQTMEAPDVVIRRAMLKAIQEGRGGLGSIYVFASGNGAGAGDNCNFDGYTNSIYSITVGAVDRTGQHPYYSEECSAQLVVTYSSGSGIHTTDVCYKAHGGTSAAAPLAAGIFALVLQVRPDLTWRDLQYLAMDTALPEANQQDTAIGFSHTFGYGKIDSWALVEKAKWELVKPQTWYFSVTFEVTQDMLKDANARLEHVTVTMNVEHQRRGDLSVDLISPKNVSHLAVSRERDAKAEGYVDWTFMSVVHWGESGAGKWTIIVRDDWRLKLWGEAIDADKARRRLRYEFLYDAF

>FVET_03649

RDYDYTPASRLGEGQLGALDDHHVFRKRIQDPIFKEQWHLLNPTQVGHDVNVTGLWLDGITGKNVTVAVIDDGLDMHSDDLKPNYFAAGSWDFNDNDPEPAPVLDEDRHGTRCAGEVAAANDVCGIGVAYDSKVAGLRILSKLISDADEAEAMMYKYDDNHIYSCSWGPSDDGQTMEAPDVVIRRAMLKAIQKGRRGLGSIYVFASGNGAGQGDNCNFDGYTNSIYSITIGAVDRTGQHPYYAEECSAQLVVTYSSGSGIHTTDVCYKAHGGTSAAAPLAAGIFALALQVRPELTWRDLQYIAMDTAIPESNQQNTTIGFSHVFGYGKIDSWALVERAKWSLVKPQSWYFSVTLEVTEDMLKDSNARVEHITVTMNVEHTRRGDLSVDLISPDNVSHLAVARRGDAKEEGYIDWTFMSVAHWGESGVGKWTIVVRDDWRLKLWGEAIEADKARRRLRYEFLYDAF

>HCAG_07015.t1

RNHDYSPAQLLGEGQIGELADHHTFSKRIADPMFHDQWHLFNTVQLGNDLNVTGLWLEGITGKESISAIVDDGIDMHSKDLKDNYFADGSYDYNDKSPIPKPRLFDDKHGTRCAGEIAAVNDICGVGVAYDSRVSGIRILSKPVSDEDEAAAINYKYQDNQIYSCSWGPVDDGMTMEGPGTLIQRAFVNGIQKGRAGRGSIYVFAAGNGALHEDNCNFDGYTNSIYSVTVGAVDRDDNHPYYSESCSAMLVVTYSSGVNIHTTDVCSTRHGGTSAAGPLVAGVVALALSVRPELTWRDIQYIFLETAIPDSDWQDTSIGFSHEFGYGKVDAYSAVHLAKWKLVKPQAWLHSSSFEVTKKMLKLHNQRLEHVTVTMNVNHTRRGDLSVELRSPAGVSHLSTTRKRDNNPVGYTDWTFMSVAHWGESGVGKWTVIVKDDWQLDLWGEAIDPNIQRKRIRYEFLYDAF

>Jan_02953

RNHDYVPTRHLGVDRVGELDDIWLVRHRVEDPLFPRQWHLVNERYPENMMNVTPVWDMGYTGKGILTALIDDGLDYTAADLAEKFDAENSYDFNDHVPLPYPKLEWQHHGTRCAGQIAASNNVCGVGIAYDSRVSGLRILGGRITTVDQATALNYGFQNVHIYSCSWGPRDDGTKMQAPRYIVRKAFLNGVNKGRGGKGSIYVFASGNGGRSGDQCNFDGYTNSIYSVTVGSVDYKGLHPTYSETCTANMIVAYSSGSGIVTTDRCAFSHGGTSAAAPNAAGVIALALQARPELTWRDVQHLCVETARRDRDWDRTAAGYSNKYGFGVIDGSLYVQRALWKLVDPQAWLQSSTISITKEMLEEANKGLEHVTIKVWIDHTRRGDVEVELVSPNGKSVLAQKRGRDEATTGYPGWTFMSVKHWGEKPIGDWTIRVSDGWNMILWGSTIDPSKAR-R-G--------

>Jan_12317

RNHDYAPSNQLGVERVGELDDVWLVRKRIEDPFFIMQWHLANGDYPEHMMNVTPVWDMGYTGKGVITSILDDGLDYTSEDLKDNFDPDNSYDFNDHEALPYPKRVRDHHGTRCAGQIAAGNKACGVGIAYESKVAG-------------------------------------------------AVLNGINKGRGGKGSIFVFASGNGAHKGDQCNFDGYTNSIYSVTVGAVDFRGQHPKYSEACAANMVVAYSSGSGIVTTDRCALVHGGTSAAAPNVAGVFALALQARPDLTWRDIQYLCVETARQDRDWERTATGYSYKYGYGVLDASLYVQRALWKLVKPQAWLETSKMTITKKMMQDANESLEHVTIKVWIDHTRRGDVEVAIVSPNGRSVLAGARERDDSMSGFPGWTFMSVKHWGEDPVGDWTIHVTDGWNMILWGTTVDPSKAG-C-G----YDQD

>KFG80585.1|_kexinlike_protease

RDYDYPPASRLHEGTVGALSDHHVFRKRIKDPIFTAQWHLFNSVEVGNDVNVTGVWMEGITGKNATVAIVDDGLDMHSEDLRENYFAEGSYDFNDHDPEPAPVLSDDHHGTRCAGEVAAVNDVCGIGVAYESKVAGIRILSAVISDEDEAEALMYKNDKNQIYSCSWGPSDDGRTMEAPSVLIRRAMLKSIQEGRNKLGSIFVFASGNGAKSGDNCNFDGYTNSIFSITVGAVSRDNQQTYYSEPCSAQLAVTYSSGGSIHTTDVCTDRHGGTSAAAPLAAGIFALVLEVDPELSWRDMQYLVMDTAKPGVVWNQTGIGFSHAFGYGKIDTYDLVQKAKWNKVKPQAWFFSANFTVTKDMLKEANERLEHVTVFMNVNHTRRGDISVDLISPSNVSQIATTRSGDEHYAGYVNWTFMSVAHWGESGVGTWTLVVRDDWRLKLWGESIDAKKAKR--RYEFLYDAF

>KLLA0D19811g.t1

KDHQYSYGNLLAEHDVRGLANHYVFSKRISDPLFDQQWHLINPNYPGNDVNVTGLWKENITGYGVVAALVDDGLDYENEDLKDNFCVEGSWDFNDNNPLPKPRLKDDYHGTRCAGEIAAFNDICGVGVAYNSKVSGIRILSGQITAEDEAASLIYGLDVNDIYSCSWGPSDDGKTMQAPDTLVKKAIIKGVTEGRDAKGALYVFASGNGGMFGDSCNFDGYTNSIFSITVGAIDWKGLHPPYSESCSAVMVVTYSSGSGIKTTDLCSNTHGGTSAAAPLAAGIYTLVLEANPNLTWRDVQYLSILSSEEDGKWQDTAMGYSHTYGFGKLDAYNIVHMAKWINVNPQGWLYLSTVSVSAEEFKQNNKRLEHVTVTVDIDAPYRGHVLVDLISPDGTSTLATARRLDKNRYGFQNWTFMSVAHWGSSGVGSWKLKV--SWRLKMFGETIDAKKAR--RIYEFEVND-

>lbic_248825

RTYDYASARALGVEQAGELRDHWLVRTRIRDPLFSQQWHIVNEDDPEHMMNVTGVWEMGLTGKGVLSSLIDDGLDYTHDDLAANFDAANSYDFNDHEALPTPKTDRDHHGTRCAGQIAAINDVCGVGIAYDSKVAGLRILSAPISDVDEAAALNYGYQDVSIYSCSWGPRDNGEKMQGPGYLVKKAVVNGINNGRQGKGSIFVFASGNGGGYGDQCNFDGYTNSIYSVTVSSVDHKGLHPYYSEACAANMIVAYSSGDGIVTTDRCATNHGGTSAAAPNAVGVFALALEARPDLTWRDIQYLCVETAQMDPDWERMASGYSYKYGFGVLDAYRYVTVAKWKLVKPQAWLATSAIKITKDMMVEHNETLEHITVKVWIDHTRRGDVEVEIVSPRGRSILAGSRERDDDKTGFPGWKFMSVKHWGENPVGEWTIKVSDGWNMVLWGTTIDPSKAK-R-A---LYDED

>lbic_322240

RTYNYVSAQALGVEQAGELQHHWIVRKRIQDPMFSRQWHLVNDDFPEHMMNVTPVWDMGFTGKGVIASLVDDGLDYESEDLAANFDADDSYDFNDHEALPTPKNFDDHHGTRCAGQVAAGNNVCGIGIAYESKVAGVRILSGPITDIDEAAALNYGFQNVSIYSCSWGPPDNGRSMEGPGYLINKAVVNGINNGRGGKGSIFVFASGNGAAHGDQCNFDGYTNSIYSVTVSAVDYKGLHPYYSEPCAANMIVAYSSGGGIVTTDKCATTHGGTSAAAPNAVGVFALALQARPDLTWRDVQHLCVETARMDPDWERTAAGYSYKYGFGVLDASRYVRAAQWKLVKPQSWFLSSTLTVSTQMLIDNNESLEHINIRVWISHSKRGDVEVEVVSPHGKSVLASTRQGDQADTGYPGWTFMSVKHWGEDSVGDWIIKVSDGWNMIFWGSTIDPSKARMV-K---LYDAF

>LELG_00316.t1

RDYNYSQIDFISEHQVNSMDNFYVFSKRIHDPEFSKQWHLLNLQYPGHDINVTGLWLDGVFGEGITTAIIDDGLDAESEDLKDNFNAKGSWDFNDNGNIPLPRLYDDYHGTRCAGEIAAVNDVCGVGVAYKSKVAGIRILSGGITAAEEAAAMVFGLDTNDIYSCSWGPTDNGKTLSEPENIVKQAMIRGIQEGRDNKGAIYVFASGNGGRYSDSCNFDGYTNSIYSITIGAIDYKGEHPIYSEACSAVMVVTYSSGSGIHTTDICSALHGGTSAAAPIASGIYSLILGANPNLTWRDLQYINVLSATPDGNYQKTALGYSHRYGYGKTDAYKMVEFAKWKNVKPQSWYYSSTVTVTKDDLEVMNEKVEHITVKVNINSSFRGKVGVRLVSPLGVSDLATFRPGDMSSRGFQDWTFMSVAHWGEDGLGEWQIEV--NWQFRIFGVSIDADKAR--R-FEFLFEQF

>MAPG_07097T0

RNYDYSPARELGEGPLGELADHHIYSKRIFDPIFLEQWHLINPIQVGHDVNVSDVWLAGITGKNSTVAIVDDGLDMDSRDLKDNYYAKGSYDFNDKHPDPKPRLSDDRHGTRCAGEVAAVNDVCGVGVAYDGKVSGIRILSKLISDADEAVALNFDYHNNHIYSCSWGPPDDGVSMDRPGILIRRAMLNAIQNGRNGKGSIYVFASGNGAMSGDNCNFDGYTNSIYSITVGAIDRTGQHPYYSEACSANLVVTYSSGSGIHTTDVCYKQHGGTSAAAPLAAGIFALVLQVRNDLTWRDMQYLALMSAIPDGEWQDTPIGFSHTYGYGKVDTWGVVELAKWKLVKPQAWYFSVKFEVTADMLKEANERLEHVTVTMNVEHTRRGDLNVDLISPSKVSHLSVTRERDNTRAGYKDWTFMSVAHWGESGVGTWTIVIKDDWHLKLWGEAKDASKVRRRLRYEFLYDAF

>MCYG_01100T0

RDYDYSPEQLLGEGQIGELEGHHTFSKRIWDPIFKQQWHLFNTLYPGNDLNVTGLWLEGITGNGSISAIVDDGLDMYSNDLKDNYFAAGSYDFNEMHSEPRPLLDDDKHGTRCAGEVAAVNDICGVGVAYDSKVSGIRILSKAINDADEAVAVNYGFQENQIYSCSWGPIDDGRTMDTPGILVRRAIANGIQKGRGGKGSVFVFAAGNGAGHGDNCNFDGYTNSIFSITVGSVDWNNEHPYYSESCSAQLVVTYSSGGGIYTTDVCSSQHGGTSAAGPLVVGVMALVLQVRPELTWRDLQYLLVETAVPAPGWQTTSIGFSHDFGYGKVDAYTTVHLAKWKLVKPQAWFHSTSFDITTEMLKKNNERVEHVTVTMNINHTRRGDLSVELHSPSGISYLSTARPQDDERAGYVDWTFMSVAHWGEKGVGNWTVIVKDDWRITLWGESIDPSIQRKRIRYEFLYDAF

>MGG_13469.t1

RDFDYEPARELGVGQLGELDSHHVFSKRIEDPIFHEQWHLFNSVQLGHDVNVTDVWLSGVTGKNATVSIVDDGLDMYSDDLKGNYYAKGSYDFNDKTEEPKPRLSDDRHGTRCAGEVAAVNDVCGVGVAFDAKVSGLRILSKLISDADEAVALNYDFHNNHIYSCSWGPPDDGKSMDAPGLLIRRAMLNAVQKGRDGKGSIYVFASGNGAANEDNCNFDGYTNSIYSITVGAIDRKGLHPYYSEACSANLVVTYSSGSGIHTTDVCYNGHGGTSAAAPLAAGIFALVLEVRPDLTWRDMQYLAFMTAVPSGDWQDTTIGYSHTYGYGKVDTFGIVEAAKWKLVKPQAYFFSTKFEVTEDM---------------------------------------------------------------------------------------------------------

>MGL_3817.t1

RTYHYTPARALDVERVGELEGHWLLRKRIRDPLFYKQWHILNEQMPGHDLNIEGAWKLAS-GKGVTVSLIDDGVEYTHPDIAHAFEPAASYDFNDHTELPWPRLFDDTHGTRCAGEIAAANDVCGVGVAPDAHIAAVRILSAPISDADEAAALNYGYQISDIYSCSWGPSDSGRSMDGPHGLVAKAMLNGIYNGRKGRGSLFVFAGGNGGSLDDQCNFDGYTNSIYTITIAAVDSSGHRPYYSEMCSAIIASAWSSGKNITTSNVCTSVHGGTSAAAPLVAGVLALALEVRPELTWRDAQHLIIQSSVPDPDWQRTTAGYSHKSGFGVVDATRLVENARHKLVPPQSWLEMNTMNVTQAMMNEANASVEHVTVKVWIEHPRRGDVQVSLYGPHGKSVLASPRRYDNDVHGFPGWTFMTLKHWNESPIGTWTIEVSDAWSLTFWGAAKDPKLALLP-W---LYNAF

>MGYG_01647T0

RDYDYSPEQLLGEGQIGELDGHHTFSKRIRDPLFKEQWHLFNPFTPGNDLNVTGLWLEGITGKGSISAIVDDGLDMYSNDLKDNYFAKGSYDFNEMQEEPRPLLDDDKHGTRCAGEVAAVNNVCGVGVAYDSKVAGIRILSKYINDADEAEAVNYGFQENHIYSCSWGPIDDGMTMDAPGLLVRRAIANGVQKGRGGKGSVFVFAAGNGAGHEDNCNFDGYTNSIFSITVGSVDWNNEHPYYSESCSAQLVVTYSSGSSIHTTDVCSSSHGGTSAAGPLVVGVMALALQVRPELTWRDLQYILVETAVPSEGWQTTSIGFSHDFGYGKVDAYSTVHLAKWKLVKPQAWFHSTSFDISPEMLKEHNERVEHVTVTMNVNHTRRGDLSVELRSPSGVSHLSTTRSKDSERVGYVDWTFMSVAHWGEKGTGVWTVIVKDDWRLTLWGESIDPSIQRKRLRYEFLYDAF

>NCU03219.t1

RDFDYSPARSLGEGPLGELQDHHLFVKRIRDPIFKEQWHLFNTVQTGHDVNVTGLWLEGVTGKNATVAIVDDGLDMETDDLKDNYYAQGSWDFNDKGPDPKPRLSDDKHGTRCAGEVSAGNKACGVGVAYDSRIAGLRILSKLISDADEAVAMNYDFQHNQIYSCSWGPPDDGQSMDAPGILIKRAMLNAVQKGRGGLGSIYVFASGNGAGNGDNCNFDGYTNSIYSITVGAVDRNGDHPYYSESCSANLVVTYSSGGGIHTTDVCSDTHGGTSAAAPLAAGIFALVLQVRPDLSWRDMQYLTVNTAVPSGEWQTTAIGFSHMYGYGKLDSYAIVQAAKWKKVKPQAWFYSVSYEVTQAMLDEANERLEHITVTMNIMHTRRGDLSVDLISPNNVSHLSVSRKNDEARAGYDDWTFMSVVHWGETGVGNWTIIVKDDWHLKLWGESKDASKARKRLRYEFLYDAF

>NFIA_103380.t1

RSYEFSPAQLLGEGQIGELANHHTFSKRIADPIFNGQWHLFNTVQLGHDLNVTGVWMEGITGKGVTTAVVDDGLDMYSNDLKPNYFPEGSYDFNDNTPEPRPRLSDDKHGTRCAGEIAAANDVCGVGVAYDSRVAGVRILSKAINDADEATAINFAYQENDIFSCSWGPPDDGATMEGPGILIKRAFVNGVQNGRGGKGSIFVFAAGNGASFEDNCNFDGYTNSIYSITVGAIDREGKHPSYSESCSAQLVVAYSSGSGIHTTDVCYSFHGGTSAAGPLAAGTVALALSARPELTWRDAQYLMVETAVPDGSWQVTKAGFSHDWGYGKVDAYALVQKAKWELVKPQAWFHSSSYEVTEQMMKNANARLEHVTVTMNVNHTRRGDLSVELRSPEGVSHLSTTRKSDNENAGYVDWTFMTVAHWGESGVGRWTVIVKDDWRLNLWGEAIDGANQRKRILYDFLYNAF

>orf19.4755

RDYNYSQIDFISEHQLSSLDNHYVFSKRIHDPEFTTQWHLINLKYPGHDVNVTGLWLENILGQGIVTALVDDGVDAESDDIKQNFNSEGSWDFNNKGKSPLPRLFDDYHGTRCAGEIAAVNDVCGIGVAWKSQVSGIRILSGPITSSDEAEAMVYGLDTNDIYSCSWGPTDNGKVLSEPDVIVKKAMIKGIQEGRDKKGAIYVFASGNGGRFGDSCNFDGYTNSIYSITVGAIDYKGLHPQYSEACSAVMVVTYSSGSGIHTTDICSATHGGTSAAAPLASGIYSLILSANPNLTWRDVQYISVLSATPDGNYQTTALNYSHKYGYGKTDAYKMVHFAKWVNVKPQAWYYSSSVNVSEKDLKIMNERVEHITVKVNIDSTYRGRVGMRIISPTGISDLATFRVNDASTRGFQNWTFMSVAHWGETGIGEWKVEV--DWQFRIFGESIDGDKAR--R-YEFLFDDF

>ORFP_Scas_Contig706.10_YNL238W

KDYRYSTAELLEEHPVRALPDHYVFSKRISDPSFPKQWHLINAAFPGNDVNVKQLWYENVTGTGIVAAIVDDGVDYDNDNIKDNFSREGSWDFNDNGPLPKPKLKDDYHGTRCAGEIAASNGICGVGVAYDAKVAGIRILSGELTAEDEAASLVHALDVNDIYSCSWGPRDDGTHLQGPTDLVKKAMIRGVTEGRDQKGALYVFASGNGGAYGDNCNYDGYTNSIYSITVGAIDHKGLHPPYSESCSAVMVVTCSSGSGIHTTDICSNTHGGTSAAAPLAAGVYTLVLQANPELTWRDIQYVSILSSKQDGDWQMGALGYSHKYGYGKMDAYDMVTMARWENVKPQSWFYSSTIKIDEDQLKKANQRVEHVTVTVNIDTQIRGPTIIDLISPEGISNLGVVRKRDVSSDGFKDWTFMSVAHWGETGIGEWKLQV--NWKLKFFGESIDPEKTR--RRYEFTNDT-

>PADG_01553T0

RNHDYSPAQMLGAGQVGELADHHTFAKRIDDPIFTQQWHLFNTEQPGHDINVTGLWLEGITGKGAISAIVDDGLDMYSNDLKDNYFAAGSYDYNDKVDEPRPRLYDDKHGTRCAGEVAGVNDVCGVGVAYDSSVAGIRILSKPVSDEDEAASINYRFQDNMIYSCSWGPVDDGTTMDAPGILVQRAIVNGIQKGRGGRGSVYVFAAGNGALHEDNCNFDGYTNSIYSVTVGAIDHNDDHPYYSEPCSAQLVVTYSSGGRIHTTDVCTTKHGGTSAAGPLVVGVVALALSVRPELTWRDVQYILLETAIPESDWQDTATGFSHEYGYGKVDAYSAVHLAMWKLVKPQAWLHSSSFEVTKELLMRNNERLEHVTLTMNINHTRRGDLSVELRSPTGVSYLSTTRKLDDLRAGYVDWTFMSLVHWGESGIGKWTVIVKDDWQLNLWGEAINADIQRKRIRYEFLYDAF

>Pans_DSM_980_PODANSg09433.t1

RNYDYSPARSLGEGPLGNLKDHHIFVKRIQDPIFNEQWHLFNTVEVGHDVNVTGLWLEGITGKNATVAIVDDGLDMYSDDLKDNYYAAGSYDFNDKTEEPKPRLSDDRHGTRCAGEVAAGNTVCGVGVAYDARISGQRILSKLISDADEAVAMNYDFDHNQIYSCSWGPPDDGKSMDAPGILIKRAMLNAVQNGRQGLGSIYVFASGNGAMAEDNCNFDGYTNSIYSITVGAIDRKGLHPYYSEKCSAGLVVTYSSGSGIHTTDVCSNTHGGTSAAAPLAAGIFALVLSVRPDLTWRDMQYLAMDTAIPDGDWQPTTIGFSHTYGYGKLDSYAIVHAAKWKNVKPQAWFYSVPFEVTEDMLKEANERLEHVTVTMNLKHARRGDVSVDLISPNKVSHLSTTRKFDDSTEGYDDWTFMSVAHWGESGVGTWTIIVKDDWHLKLWGESRDASKARKRLRYEFLYDAF

>pbla_23527

RD-HYHAANSLGEGPVGELQTYFLVSKRITDPGFPQQWHLVNQQYPGKDINVVDVWKQGVTGNGSTVVILDDGLDYESQDLAANFFAEGSYDFNDHTPFPKPRLWDDSHGTRCAGQIAAVNDVCGIGIAYNSRVAGVRILSGDITDADEAAALNYKYQDNDIYSCSWGPSDDGEKMEAPVGMLADAFQNGVKNGRGGKGSVFVFATGNGAASGDNCNFDGYTNSIYTITVGAIDHTDNHPPYSESCSAQLVVTYSSGGGIYTTNVCTNSHSGTSAAAPNAAGIFALVLGVRPDLTWRDMQHLCVQTAEPK-DWKRLPSGYNHKFGYGRLNTLALIEAARFESVNIQT--HLSAITVTEEMIKAAGLRLEHVTATVDIEHQQRGNIVINLQSPHMESELATERPRDLSPDGIRDWKFMSVKHWDENPVGDWTLLVYDKWTLTLFGE--------------------

>pbla_36687

----------------------------IRDPGFDKQWHIINRDHRGHDINVAGVWSQNITGQNVVVAILDDGLDMDNEDLKDNFFAPGSYDFNDHTNLPKPKLFDDTHGTRCAGEIAAVNDVCGVGMAYGAKVAGIRILSADITEADEAAALNYKFQQNDIYSCSWGPPDQGEVAEAPKGIVLDAIKNGINNGRDGSGTIFVFASGNGGANDDNCNFDGYTNSLYTITVGAIDRLDRHPYYAESCSAQLIVTYSSGNGIYTTDVCSDRHGGTSAAAPLAAGVFALVLSVRPDLTWRDMQHLCVRTAVPD-DWDVLPSGYNHKFGYGKLDAFAIVEAAKFKSVGPHT--FLSVVAIDQERLDSVGGTLEHVTVTVDIEHGRRGDLEVFLESPNKVSKLGASRKFDNSKDGLVNWTFMSVKHWEENPVGDWTLRVMDQWSLTLWGE--------------------

>Pchr_Wisconsin_541255_Pc22g029

RSQDFSPAQVLGEGQIGQLDGHHTFSKRITDPIFREQWHLLNTLQPGHDLNVTGLWLEGITGKGVVTAVVDDGLDMDSNDLKPNYLPEGSYDFNENVPEPRPLLLDDKHGTRCCGEIAAANDVCGVGVAYDSKIAGIRILSKPIDDVDEAAAINFAYQTNDIYSCSWGPIDDGATMDAPGILIKRAMANGVQKGRGGKGSVFVFAAGNGAAYGDNCNFDGYTNSIYSITVGAIDREGNHPSYSESCSAQLVVAYSSGSGIHTTDVCFSGHGGTSAAGPLAAGSAALALSARPDLTWRDLQHLMVETAVPDGSWQVLPSGFSHDWGFGKVDTYTMVQLAKWELVKPQAWLHSSRYTVTADQLKEANAKLEHVTVTMNVNHTRRGDLSVELRSPAGVSYLSVARRKDDMPVGYDDWTFMSVAHWGESPVGDWSIIVKDDWRLNLWGEAVDGTKQIKRRRYEFLYNAF

>PGTT_08683

RSYAYSGADRLDVERVGELENFWLIKKRIYDPLWPKQWHLVNDVIHKHMINATGVWEMGITGKNVTVAIVDDGIDMSSDDLKSNFFEAGSWDYNDHTPLPEPRLPDDLHGTRCAGEIAAVNDVCGVGVAFDGKVAGIRILSASISDADEASALNYGYQENHIYSCSWGPPDDGKSMEAPSRLIFKAMLNGIQKGRGGKGSVFVFASGNGGAVDDQCNFDGYTNSIYSVTISAIDRQGLHPYYSEVCSANMVVTYSSGSGIHTTDVCTDRHGGTSAAAPLGAGIFALVLQARPDLTWRDVQYLAVTTAIPP-DWQKTASGYNHKFGFGNMDAYQIVQAAKWKLVKPQAWWTSATIVVSQADLDGANESLEHITVAVNIKHTRRGNVRVLLISPHGVSILAAHRRYDDASTGFPGWVFMTVKHWGENPVGAWTLSVQDDWAMGMWGECKDPTIQRMRRR-------F

>PGUT_03723

KDHRYSSADFITEHPVRGLDDHFVFSKRINDPTFKEQWHLINTFNPGHDVNVTGLWYEGITGKGIVSALIDDGLDYESEDLKDNFNMKGSWDFNDNRNLPMPTLYDDWHGTRCAGEIAAVNDVCGLGVAYESNVSGIRILSGPITAEDEAAAMIFGLDVNDIYSCSWGPTDNGKVVSAPNKIVKKAMIKGVQDGRDKKGAIYVFASGNGGRFGDSCNFDGYTNSIYSITVGAIDYKGMHPIYSEACSAVMVVTYSSGSGIHTTDICSAIHGGTSAAAPLAAGVFALVLQSNPDLTWRDLQYIAALSSIPDGNYQDSALGYSQRYGFGKLDAYGMAHFAKWKNVKPQAWYYSSVIDVTEHDMKVSNERVEHVTVTVNIQATFRGKIGVRLISPRGISDLATERRGDRSMSGLKNWTFMSVANWGEKGTGNWTLEV--DWSLRIFGESIDPAKAR--R-YEFLFDEL

>Pmar_ATCC_18224_PMAA_039890.t1

RTYDFTPAEVLGEGQIGNLPGHHKFSKRIKDPEFEKQWHLFNTVQVGHDMNVTGLWLEGITGAGVVTAIVDDGLDMYSNDLKANYFAEGSWDFNEDSPEPRPLLRDDKHGTRCAGEVAAVNNVCGVGLAYDSKVAGIRILSKPIDDADEAAAINYGFQKNDIYSCSWGPRDDGETMEAPGILVRRAMVNGIVQGRGGKGSIYVFAAGNGGFYGDNCNFDGYTNSIYSVTVGAIDRQGQHPSYSEACSAQLVVAYSSGGSIHTTDVCTSIHGGTSAAGPLVSGAIALALSVRPELTWRDVQYLLIETSVPGDEVQMTPIGFSHQFGYGKVDTYSFVQMAKWDLVKPQAWFTTSYFEVTSDMLKAANERIEHVTVTMNINHTRRGDLSTELRGPQGVSHLSVPRNKDGAIAGYEDWTFMSVAHWGETGEGVWSVVVKDDWRMTLWGVSIDPAVQRNRLRYEFLYNAF

>Pnod_SN15_SNOG_04420.t1

RNYDYSPAAHLGEGPLSSLAEHHVFKRSIHDPIFEEQWHIFNVKD-------TRQRHQRITGKGVTACVVDDGLDYDSNDLKDNFFAEGSHDYNDHEDLPTPKLSDDRHGTRCAGEIAAGNDACGVGLAYDAKISGVRILSGDITDMDEALAINYEMQKNFIYSCSWGPPDDGKTMQAPGILIEKAMVTAVQQGRGGKGSIYVFAAGNGAASDDNCNFDGYTNSIYSITVGAIDKNNQHPYYSEACSAQLVVTYSSGGGIHTTDVCTSQHGGTSAAGPIGVGVYALLLEARPDLTWRDVQWLTVMTAVPPSDWTKTALDYSHQFGYGKLDAWAIVEKSKWKLVKPQAWFWSSVFEVTADMLKEANERVEHITLTMNVKHQRRGDLLVQLHSPTGISHLSTARRDDEDIRGYQDWTFMSVAHWGESGVGNWTVIIKDDWKLRLWGESIDAAKARKRLRYEFLYDAF

>ppla_117103

RHYDYASASSLGVEQAGALQDHWLVRKRITDPEFGRQWHIVNDASPHNMMNVTGVWDMGITGKGVISALVDDGLDYTSDDLAANFYAYGSYDFNDHLDLPTPTLFDDHHGTRCAGQIAAVNDVCGVGIAYDSKVAGLRILSGPISDVDESAALNYDYQNTSIFSCSWGPPDDGRSMEGPGYLIKKAMVNGVQNGRQGKGSIFVFASGNGGRSADQCNFDGYTNSIFSVTVAAVDYRGLHPDYSEACAANMVVAYSSGSGITTTDRCAHSHGGTSAAAPNAAGIFALALQTRPDLSWRDVQHLCVKTAQMDPDWETTAAGFSYKYGYGVINGYEFIKAAQWQLVKPQAFIDLNSIEVTQSMLDENNEKLEHITVKVWITHTRRGDVEVELVSPNGRSILAATRYGDSAKTGFPGWTFMTVKHWDENPVGKWSLRVNDGWTMTVWGSVKDATKPR-A-R---LYDAF

>psti_14973

RDYNYSQADFTSEHQLQGLDNHYVFSKRIRDPGFIEQWHLINTAYPGHDVNVTGLWYEGITGTGIVSAIVDDGLDAESEDLRANFNAKGSWDFNDNTNIPLPRLYDDHHGTRCAGEIAAVNDVCGVGVAYDSTVAGIRILSGPITAAEEAAALIYGLDVNDIYSCSWGPTDDGRTLAEPETVVKKAMIKGVQEGRKDKGSIYVFASGNGGRSYDSCNYDGYTNSIFSITVGAIDYKGIHPDYAEACSAVMVVTYSSGSGIHTTDICTASHGGTSAAAPLAAGIYALVLQANPNLTWRDVQYVSVLSSVPDGNYQTTALNYSHKYGYGKIDAYQMVHFAKWKNVKPQAFFYSKKITVTEEDLKIMNERVEHVTVKLNIMATFRGRVGVRLISPTGTSDLATFRPRDNSGVGFKDWTFMSVAHWGESGLGDWTIEV--NWQLRFFGESIDADKAR--R-FEFVYDEF

>Ptri_Pt1CBFP_PTRG_07955.t1

RNYDYSPAAHLGEGPFGSLEDHHVFKRSIKDPIFEEQWHLFNVKTPGNDINVTGVWTQGITGKNVTACVVDDGLDYTSNDLKDNFFAQGSHDYNDHEDLPTPKLSDDRHGTRCAGEIAAGNDACGVGLAYDAKISGVRILSGDITDLDEALAINHEMQANDIYSCSWGPPDDGKTMQAPGILIEKAMVTAVQQGRGGKGSIYVFAAGNGAASDDNCNFDGYTNSIYSITVGAIDMNNAHPYYSEACSAQLVVTYSSGGGIHTTDVCTAQHGGTSAAGPIGVGVFALALSARPELTWRDVQWITVMTAIPPSDWTKTSLGFSHQFGYGKLDAWAVVEKAKWKLVKPQAWFYSSSFEVTEEMLKKVNERVEHITLTMNIEHERRGDLSVELRSPSGVSHLSTPRRSDEAPYGYVDWTFMSVAHWGENAVGNWTVIVKDDWKLRLWGECIDASKARKRMKYEFLYDAF

>RO3T_00078

-------AQHLGEGQVGELSTYYMVS--IQDPLFNKQWHLINQMNTGNDINVTGVWKQGISGKGVTVVIVDDGLDYNSTDLAANFYAEGSYDFNDHESLPTPKLWDDTHGTRCAGQIAAVNNACGIGIAYESKIAGVRILSGDLTDADEALALNYKYQENDIFSCSWGPTDNGETMEAPKGILADAFLNGIKNGRGGKGSIYVFATGNGGTSGDNCNFDGYTNSIYTITVGAIDFTNSHPPYSEACSAQLV---------QTTDVCSDRHGGTSAAAPNAAGIFALVLSVRPDLSWRDLQHLCVQTAVPS-DWKRLPSGYNHKFGYGKLDAYALVEAAKHKGVNQQT--WLSIVKVTEEMIKAAGLRLEHITATVNIEHQRRGDLTIDLLSPHQKSELATRRNLDTSTEGFPNWKFMSVKHWEENPVGDWTLTIYDNWTLTLYGE-QDPEFVHPLDKYEFLGN--

>RO3T_06131

RE-DY-VAQQLGEGQVGELDTYYMVS--IKDPLFDKQWHLINQKNPGNDINVTGVWKQGIAGKGVTVVILDDGLDFNSTDLADNFYAEGSYDFNDHEPLPKPKLWDDTHGTRCAGQIAAVNNACGVGIAYESKVAGIRILSGDLTDADEALALNYDYQHNHIFSCSWGPPDNGENMEAPKAILTDAIANGVRNGRDGKGSIYVFATGNGATLGDNCNFDAYTNSIYTITVGAIDHTNKHPAYSESCSAQLVVTYSSGSGIQTTDVCFDRHGGTSAAAPNAA-------------------------AVPS-DWKELPSGYNHKFGYGKLDAYALVEAAKFKSVNQQT--WLSIVKVTEGMIKAAGLKLEHVTATVNIEHERRGDLVINLESPHLKSELATRRILDKSKDGILNWKFMSVKHWEEDPIGDWVLSVYDNWTLTLYGE-QDPEFKYVVPTYEF-----

>SCY_4566.t1

KDHQYSNSRLEEEHDVRGLPNHYVFSKRINDPLFERQWHLVNPSFPGSDINVLDLWYNNITGAGVVAAIVDDGLDYENEDLKDNFCAEGSWDFNDNTNLPKPRLSDDYHGTRCAGEIAAKNNFCGVGVGYNAKISGIRILSGDITTEDEAASLIYGLDVNDIYSCSWGPADDGRHLQGPNDLVKKALVKGVTEGRDSKGAIYVFASGNGGTRGDNCNYDGYTNSIYSITIGAIDHKDLHPPYSEGCSAVMAVTYSSGSGIHSSDICSNSHGGTSAAAPLAAGVYTLLLEANPNLTWRDVQYLSILSAVGDGDWRDSAMGYSHRYGFGKIDAHKLIEMSKWENVNAQTWFYLSVITISEKSLQDANKRIEHVTVTVDIDTEIRGTTTVDLISPAGISNLGVVRPRDVSSEGFKDWTFMSVAHWGENGVGDWKIKV--SWRLKLFGESIDSSKTR--RRYEFITEP-

>SJAG_04397T0

-----SPAEFLGVRRMRNLPNYFVYSKRINDPLLSQQWHIINTNAIGHDLNVTGVWEEGYLGENVTVAFVDDGLDFRHADLQDAFSAVGSWDFNDDVPEPLPKLADDTHGTRCAGEVAAANDVCGVGIAPKAKVAGLRMLSGPVTDLMESEALNYGFDTNDIYSCSWGPADDGRAMEAPEPATRKALLNGVVNGRNGLGSVFVFASGNGGYYDDNCNFDGYTNSIFSVTVGAVDTEDSWPAYGEYCAAQLVSAYSSGHNIVTTNVCTHRHGGTSAAAPLGSAVYALALSARPELTWRDIQHITVYSALP-----------SHKFGFGKLDAGRFIETAKWELVKPQTWYITLKFNMTRAMVHQSNQDLEHVTVRTTIPFSRRGKLQVVLRSPSDESVLATERPFDENAQGIQDWTFMTVQHWGEKPEGVWTLIVRDNWQLGLWGQASNASQTKSP----------

>SOCG_00573T0

-----DPADAIGVRPMRNLKNHYLFAKRLIDPLLSDQWHILNYEVPGHDLNLQEVWDAGILGENVTVAFVDDGIDFRHPDLQAAYSSLGSWDFNDNMADPLPKLSDDLHGTRCAGEVAGANDVCGVGIAPKAKVAGLRILSAPINDVVESEALNYGFQTNHIYSCSWGPADDGRAMEAPRLATRRALINGVINGRNGLGSVFVFASGNGGHYRDNCNFDGYTNSIFSITVGAVDMDHQVPFYSEICAAQLISAYSSGSRIATTNPCTKSHGGTSAAAPLASAVYALALSIRPDLTWRDIQHITVHSAVPDLEWTKTPAGFSHRFGFGKLDAKRFIDITRWQLVNPQTWVISSELAVTEEMVEKSNKSLEQITVKVSIPFTCRGAMTIELESPAGRSMLATLRPYDQNNEGFPEWTFMTVQHWSESIIGSWKLIVRDYWQLAFWGESQDPSLTN-P----------

>SPOG_01932T0

-----DPADAIGVRPMRNLENHHLFTKRLNDPLLSEQWHILNYEKPGHDLNLREVWDAGILGENVTVAFVDDGIDFRHPDLQAAYTSLGSWDFNDNMADPLPKLSDDLHGTRCAGEVAASNDVCGVGIAPKAKVAGVRILSAPINDVVESEALNYGFQTNHIYSCSWGPADDGRAMEAPRLGTRRALVNGVINGRNGLGSVFVFASGNGGHYRDNCNFDGYTNSIFSVTVGAVDIEHQVPFYSELCAAQLVSAYSSGSHIATTNPCTKSHGGTSAAAPLASAVYALALSIRPDLTWRDIQHITVHSAIPNVEWTKTPAGFSHKFGFGKLDAKRFIETASWQLVNPQTWLVSSEYVVTEEMIEKSNKGVEQITIKVSIPFTCRGAMTIELESPAGRSMLASLRPYDQNNEGFPEWTFMTVQHWSEPILGSWKLIVRDYWQLAFWGESRDPSLTS-P----------

>Spom_972h_SPAC22E12.09c.t1

-----DPAEAIGVRPLLNLKYHHLIKKRISDPLFYGQWHIFNSNNPGHDLNLREVWDAGYFGENVTVAFVDDGIDFKHPDLQAAYTSLGSWDFNDNIADPLPKLSDDQHGTRCAGEVAAANDVCGVGIAPRAKVAGLRILSAPITDAVESEALNYGFQTNHIYSCSWGPADDGRAMDAPNTATRRALMNGVLNGRNGLGSIFVFASGNGGHYHDNCNFDGYTNSIFSATIGAVDAEHKIPFYSEVCAAQLVSAYSSGSHILTTNPCTRSHGGTSAAAPLASAVYALALSIRPDLSWRDIQHITVYSASPNAEWQKTPAGFSHHFGFGKLDASKFVEVAKWQVVNPQTWLIASEFTVTKDMIEKSNKRLEHVTVRVCIPFNRRGALEILLESPSGRSILASERPYDENSKGFLDWTFMTVQHWAEPPEGVWKLLVNDNWQLALWGESENPSNTKAP----------

>sros_784

RTYAYLEAEALGVEQVGELRDHYLIRQRILDPLWPKQWHLVNGVIEENSINVTGVWDQGVFGKGVNVAIVDDGLDMHSDDLAANFHAEGSWDYNDNTPLPEPRLSDDQHGTRCAGEIAAVNDVCGVGVAHQAGIAGIRILSASISDADEASSLNYGYQTNDIYSCSWGPPDDGRSMEAPGRLITKAMLNGVTNGRGGKGSVFVFASGNGGAVDDQCNFDGYTNSLMSITVGAIDRKGLHPFYSEACAANMVVTYSSGSGIHTTDVCTDRHGGTSAAAPIAAGIFALVLEARPDLTWRDMQHLCVRTAVQP-DWQMTASGYNHKYGFGKLDAWAIVNAARWQIVKPQTWWNSLTTGGSRITSDGIASSRLRNRISKMPTSKSLSTSRSLSLSSMNKSNXXARKAXRVFSRGREGLMRRRRVCW----VGSSXLS---RPEHSTAGRCNSGARRGSSSP-------F

>SS1G_06027

RNYDYDPAERLGDGQLGELEDHHVFSKRIQDPIFKDQWHLYNPVQVGHDVNVTDVWMQNITGIGSIVAIVDDGLDMYSNDLKANYYAEGSYDFNENTLEPKPRLSDDNHGTRCAGEVSAVNDVCGVGVAYDSKIAGIRILSKMITDADEAVAMNYAFQHNQIYSCSWGPPDDGRSMDAPGILIKRAMVNAVQKGRGGLGSIYVFASGNGAANEDNCNFDGYTNSIYSITVGAIDRKGLHPYYSEKCSAQLVVTYSSGSGIHTTDVCSSLHGGTSAAAPLAAGIFSLVLQIRPDLSWRDMQYLVMSTALPTGEWQTTTIGFSHTFGYGKIDTWATIEAAKFKNVKPQAWFYSVSFEVTKEMLQAANERLEHVQVTMNVAHTKRGELSVDLVSPDKVSHLSTTRRYDSDTDGYDDWTFMSVVHWGESGIGTWTITVRDDWHLKLWGEAIDAEKARKRLRWEFLYDAF

>TEQG_02035T0

RDYDYSPEELLGEGQIGELEGHHTFSKRIRDPLFKEQWHLFNPYTPGNDLNVTGLWLEGITGKGSISAIVDDGLDMYSNDLKDNYFAKGSYDFNEMKAEPRPTLDDDKHGTRCAGEVAAVNNVCGVGVAYDSKVAGIRILSKYINDADEAEAVNYGFQDNQIYSCSWGPIDDGMTMDAPGL-CRRAIANGVQKGR---------AAR--APHDDNCNFDGYTNSIFSITVGSVDWNNEHPYYSESCSAQLVVTYSSGSGIHTTDVCSGSHGGTSAAGPLVVGVMALALQVRPELTWRDLQYILVETAVPSDGWQTTSIGFSHDFGYGKVDAYSTVHLAKWKLVKPQAWFHSTAFDISPEMLKSHNERVEHVTVTMNVNHTRRGDLSVELRSPSGISYLSTARAQDSERAGYVDWTFMSVAHWGEKGTGVWTVIVKDDWRLTLWGESIDPKIQRKRLRYEFLYDAF

>TEQG_08695T0

KTHDFSPANHLKEGQIGELTDYHKFSKRITDPLFNSQWHLFNTVQLKQDMNVTGAWLEGVTGKGTVTAVIDDGLDFHSNDLNNNYFPAGSYNFVENSKEPDPKYVNQTHGT--------------------------------------------------------------RVIGGPGTMVKRALENGVANGRRGKGSIFVVSAGNGGYLDDDCNFDGYANSIYTIAVGAIDREGNHPEYSEPCSALSVVAYASAGAIYTTDVCASGHGGTSAAAPLVAGAIALALSVRSELTWRDIQYLLYATAIPEDDWQMTKLGFSHNYGYGKVDSYGLVQMARWKLVKPQTSYHSSIFEVRPPAI--SVERLEHVTVTTNVNHTRRGDLSIELHSPEGVSRLSTTRRNDNATSGYAGWAFMSVAHWGESGIGNWTIVVKDSWQLSLWGEGTD-----------------

>TERG_02808T0

RTHDFSPATHLGEGQIGELTDHHNFSKRITDPLFNSQWHLFNTVQLTQDMNVTGAWLEGVTGKGTVTAVIDDGLDFHSNDLKKNYFPAGSYNFVENSKEPDPKHVNQTHGTRCVGEIAPGNGICGLGMAYDGKISGIRLLSGQIDESDEAAAINYQYQSNDIYSCSWGSPDNGRVIGGSGTLVKRALENGVTNGRQ---------------------------------------------------------------IYTTDVCVAGHGGTSAAAPLVAGAIALALSVRLELTWRDIQYLLYAMAVPEDDWQMTKLRFSHNYGHGKVDSYSLVX---------------------------------------------------------------------------------------------------------------------------------

>TERG_07513T0

RDYDYSPEELLGEGQIGELDGHHTFSKRIRDPLFKEQWHLFNAFTPGNDLNVTGLWLEGITGKGSISAIVDDGLDMYSNDLKDNYFAKGSYDFNEMKAEPRPMLDDDKHGTRCAGEVAAVNNACGVGVAYDSKVAGIRILSKYINDADEAEAVNYGFQDNQIYSCSWGPIDDGMTMDAPGLLVRRAIANGVQKGRGGKGSVFVFAAGNGAGHDDNCNFDGYTNSIFSITVGSVDWNNQHPYYSESCSAQLVVTYSSGSGIHTTDVCSGSHGGTSAAGPLVVGVMALALQVRPELTWRDLQYILVETAVPSDGWQTTSIGFSHDFGYGKVDAYSTVHLAKWKLVKPQAWFHSTAFDISPEMLKAHNERVEHVTVTMNVNHTRRGDLSVELRSPSGISYLSTARAQDSERAGYVDWTFMSVAHWGEKGTGVWTVIVKDDWRLTLWGESIDPKIQRKRLRYEFLYDAF

>TESG_07565T0

----------------------------IRDPLFKEQWHLFNPYTPGNDLNVTGLWLEGITGKGSISAIVDDGLDMYSNDLKDNYFAKGSYDFNEMKAEPRPTLDDDKHGTRCAGEVAAVNNVCGVGVAYDSKVAGIRILSKYINDADEAEAVNYGFQDNQIYSCSWGPIDDGMTMDAPGLLVRRAIANGVQKGRGGKGSVFVFAAGNGAGHDDNCNFDGYTNSIFSITVGSVDWNNEHPYYSESCSAQLVVTYSSGSGIHTTDVCSGSHGGTSAAGPLVVGVMALALQVRPELTWRDLQYILVE----------------------------------------------------------------------------------------------------------------------------------------------------------------

>TESG_08213T0

RDYDYSPEELLGEGQIGELEGHHTFSKRIRDPLFKEQWHLFNPYTPGNDLNVTGLWLEGITGKGSISAIVDDGLDMYSNDLKDNYFAKGSYDFNEMKAEPRPTLDDDKHGTRCAGEVAAVNNVCGVGVAYDSKVAGIRILSKYINDADEAEAVNYGFQDNQIYSCSWGPIDDGMTMDAPGLLVRRAIANGVQKGRGGKGSVFVFAAGNGAGHDDNCNFDGYTNSIFSITVGSVDWNNEHPYYSESCSAQLVVTYSSGSGIHTTDVCSGSHGGTSAAGPLVVGVMALALQVRPELTWRDLQYILVETAVPSDG---------------------------------------------------------------------------------------------------------------------------------------------------------

>TESG_08312T0

KTHDFSPANHLKEGQIGELTDYHKFSKRITDPLFNSQWHLFDTVQLKQDMNVTGAWLEGVTGKGTVTAVIDDGLDFHSNDLNNNYFPAGSYNFVENSKEPDPKYVNQTHGTRCAGEIAAGNGICGLGMAYDGKIAGIRLLSGQIDESDEAAAINYRYQSNDIYSCSWGPPDNGRVIGGPGTMVKRALENGVANGRRGKGSIFVVSAGNGGYLDDDCNFDGYANSIYTIAVGAIDREGNHPEYSEPCSALSVVAYASAGAIYTTDVCASGHGGTSAAAPLVAGAIALALSVRSELTWRDIQYLLYATAIPEDDWQMTKLGFSHNYGYGKVDSYGLVQMARWKLVKPQTSYHSSIFEVRPPAI--SVERLEHVTVTTNVNHTRRGDLSIELHSPEGVSRLSTTRRNDNATSGYAGWAFMSVAHWGESGIGNWTIVVKDSWQLSLWGEGTD-----------------

>tree_123561

RDYDYQPASRLGEGQLGELTDHHVFRKRIADPIFKEQWHLLNTVQVGHDVNVTGLWLEGITGKNATVAMVDDGLDMYSRDLKDNYFAEGSWDFNDNDPEPKPELSDDRHGTRCAGEISAVNDACGLGVAYDSKIAGIRILSSAISDADEAEAMIYKFQDNQIYSCSWGPPDDGRSMEAPDVLIRRAMLKGVQEGRGGLGSIYVFASGNGAASGDNCNFDGYTNSIYSITVGAVDRAGQHPYYSEHCSALLVVTYSSGGGIHTTDVCYGMHGGTSAAAPLAAGIFALVMQVRPDLTWRDLQYLAMDTAVKDAEWQTTAAGFSHTFGYGKIDSYSLVEKAKWQKVKPQAWFFSVSFDVTADMLKDANARLEHVTVTMNVEHTRRGDLSVDLISPDNISHIAVTRKSDDHKGGYDDWTFMSVAHWGETGIGRWTLVVRDDWHLKLWGESIDASKARRRLRYEFLYDAF

>um02843

RSYHYDPAEALGVERAGELQNHWLVRKRIKDPIFTDQWHLANDRKTGNDLNVTAIWEQGILGKGIKVCLIDDGLDMHSPDLRDNFYAPGSYDFNSHTELPEPRESDDQHGTRCAGEIAAVNDVCGVGVAYEAKVSGVRILSGPISDVDEAASLNYAYQENDIYSCSWGPPDDGRSMDAPKGLIAKAMLNGVQNGRDGKGSVFVFAGGNGGASDDQCNFDGYTNSIYSMTIAAVDREGQHPWYSEMCSAIIATSWSSGSGIHTTDVCTGSHGGTSAAAPLAAGVIALGLSVRPELTWRDVQHIAVRSAVKDPDWQQTQAGFNHKYGYGLLDAYQFVQEAKHKLVNPQAWYESSTYTVTEDHLKGANASVEHVTVRVWITHQRRGDVNVELISPHGKSALARSRRYDDATTGFPGWSFMTLKHWGESPTGEWKLRVFDAWSMSLWGESIDPSKAKMR-R---LYDAF

>URET_00628

RDHDYSPAQILGEGQIGELEDHHTFSKRIKDPIFVNQWHLFNTVQPGHDLNVTGLWLEGITGNGTITAIVDDGLDMYSNDLKDNYFAEGSYDFNDKGKEPKPRLSDDKHGTRCAGEVAAVNNVCGVGVAYNGKVAGIRILSKPVTDEDEAAAINYGFQQNQIYSCSWGPVDSGATMDAPGLLIRRAMVNGIQKGRDGKGSLFVFAAGNGAGNGDNCNFDGYTNSIYSITVGAIDREDQHPYYSESCSAQLVVTYSSGGNISTTDICSNKHGGTSAAGPLVVGVVALALGVRPELTWRDMQYLIVETAIPQEGWQTTAIGFSHDFGYGKVDAYSMVQLAKWKLVKPQAWLHSSSFEITEEMLKKNNERIEHVTVTMNVNHTRRGDLSVELRSPTGVSHLSVARDGDTVNAGYVDWTFMSVAHWGETGKGKWTVIVKDDWQLSLWGESIDGKIQHKRLRYEFLYDAF

>VDAG_01043T0

RNYDYAPAERLGEGELSGLDGHHIFAKRINDPIFDVQWHLFNPVQKGHDINVTGVWMGGITGQNATVAIVDDGLDMYSGDLKPNYYAAGSYDFNDHRDEPKPTLADDRHGTRCAGEVSAANDVCGVGVAYDSKIAGLRILSKLISDADEAVAMTYDYQHNDIYSCSWGPPDDGRSMDAPGILIKRAMLKGIQDGRNGLGSIYVFASGNGAAKEDNCNFDGYTNSIYSITVGAVDRTGQHPYYSEKCSAQMVVTYSSGAGIHTTDVCTDAHGGTSAAAPIGAGVYALVLSARSDLSWRDMQWLAMDTAVPTGEWQDTTIGFSHTFGYGKIDAYAMVEAAKWKKVKAQAWYYSVSHEVTSDALKQANARVEHVTVTMNVNHTQRGDLSVDLISPDGVSHIATTRKNDKDANGYIDWTFMSVAHWGEKGIGKWTVIVKDDFHIKLWGESIDEKKARRKLRYEFLYDAF

>VDBG_03817T0

RNYDYAPAERLGEGELSGLDGHHVFAKRINDPIFDVQWHLFNPVQKGHDINVTGVWMGGITGQNATVAIVDDGLDMYSGDLKPNYYAAGSYDFNDHRDEPKPTLADDRHGTRCAGEVSAANDVCGVGVAYDSKIAGLRILSKLISDADEAVAMTYDYQHNDIYSCSWGPPDDGRSMDAPGILIKRAMLKGIQDGRNGLGSIYVFASGNGAAKEDNCNFDGYTNSIYSITVGAVDRTGQHPYYSEKCSAQMVVTYSSGAGIHTTDVCTDAHGGTSAAAPIGAGVYALVLSARPDLSWRDMQWLAMDTAVPTGEWQDTIIGFSHTFGYGKIDAYAMVEAAKWKKVKAQAWYYSVSHEVTSDALKQANARVEHVTVTMNVNHTQRGDLSVDLISPDGVSHIATTRKNDKDANGYVDWTFMSVAHWGEKGIGKWTVIVKDDFHIKLWGESIDEKKA-----YEFLYDGF

>Vpol_DSM_70294_Kpol_1015p8.t1

KDHQYSNEELVSEHDVRGLDNHYVFSKRIKDPLFERQWHLINPSFPGHDVNVTGLWYEGITGKGVVVAIVDDGLDYESEDLKDNFCKEGSWDFNENQNLPKPLLYDDYHGTRCAGEIAAANEYCGLGVAYGSKVSGIRILSGQITAEDEAASLVYGLDVNDIYSCSWGPPDDGKHLQGPSELVKKSLLKGIQEGRGEKGSIYVFASGNGGMFGDNCNYDGYTNSIYSITVSAIDHKGMHPPYAESCSAVMVVTYSSGSGIHSTDICSDRHSGTSAAAPLAAGIYALVLEANPNLTWRDIQYLSILAAAEDGEWQDGALNYSHRYGYGKIDSYEIATMAKWKNVNPQTWYYH----------------IEEVN---------------------------------------------------------------------------------------------

>XP_007809137.1|_kexinlike_prot

RDYDYSPESRLHEGTVGALSDHHVFRKRIKDPIFTAQWHLFNSVEVGNDVNVTGVWMEGVTGKNATVAIVDDGLDMHSEDLRENYFAEGSYDFNDHDPEPAPVLSDDHHGTRCAGEVAAVNDVCGIGVAYESKVAGIRILSAVISDEDEAEALMYRNDKNQIYSCSWGPSDDGRTMEAPSVLIRRAMLKSIQEGRNKLGSIFVFASGNGAKSGDNCNFDGYTNSIFSITVGAVSRDNQQTYYSEPCSAQLAVTYSSGGSIHTTDVCTDRHGGTSAAAPLAAGIFALVLEVNPELSWRDMQYLVMDTAKPGVVWNETGIGFSHAFGYGKIDTYDLVQKAKWNKVKPQAWFFSANFTVTKDMLKEANGRLEHVTIFMNVNHTRRGDISVDLISPSNVSQIATTRSGDEHYAGYVNWTFMSVAHWGESGVGAWTLVVRDDWRLKLWGESIDAKKAKR--RYEFLYDAF

>YALI0F13189g.t1

RDYVY-SLDHGQEHPVGTIPNTYVFSKRIKDPSLWKQWYLHNVHKAGHDLNVTGLWLRNVTGWGVVTAVVDDGLDMNAEDIKANYFAEGSWDFNFNKSDPKPSSHDDYHGTRCAGEIAAVNNVCGVGVAYDSKVAGIRILSKEIAEDIEALAINYEMDKNDIYSCSWGPPDNGQTMARPGKVVKDAMVNAITNGRQGKGNVFVFASGNGGSRGDNCNFDGYTNSIYSITVGALDFNDGHPYYSEACSANMVVTYSSGSEIVGTDICQNQHGGTSAAAPLAAGVFALALSVRPDLTWRDMQYLALYSAVED-GWQDTASGFHHQFGYGKLDASKIVELAEWNLVNNQTSFHSSVITVTRDDLDKVNKRAEHITAVLNLEASYRGHVRVLLKGPRGVSELAALRRDDRSKDGYDNWAFMSVAHWADEGEGDWELTV--NWQLNVFGE-QKDKRERVRRNFEFLYDPF
