# Supplementary material for: Phylogenomic evolutionary surveys of subtilase superfamily genes in fungi
Source: Sci Rep. 2017 Mar 30;7:45456. doi: 10.1038/srep45456 (PMC5371821; doi:10.1038/srep45456)
Supplement: Supplementary Data S11 [file srep45456-s11.docx]

**Phylogenomic evolutionary surveys of subtilase superfamily genes in fungi**

Juan Li*, Fei Gu, Runian Wu, JinKui Yang and Ke-Qin Zhang*

*State Key Laboratory for Conservation and Utilization of Bio-Resources in Yunnan*, *Yunnan University*, *Kunming*, *650091*, *P.R. China.*

* Corresponding author: Juan Li and Ke-Qin Zhang

Tel: 86-871-65033805; Fax: +86-871-65034838.

E-mail address: [juanli@ynu.edu.cn](mailto:juanli@ynu.edu.cn) (Juan Li); kqzhang@ynu.edu.cn(Ke-Qin Zhang)

**Supplementary data S11: 126 S53 family sequences amino acid sequences in fungi.**

For S53 family genes, MUSCLE v3.5 was used to generate protein alignment with default settings[^20^](#_ENREF_20). The ambiguous areas of alignment were located and removed by using the program Gblocks 0.91b [^21^](#_ENREF_21)^,^[^22^](#_ENREF_22) with default parameters. The gap selection criterion “with half” was used here. Finally, an alignment consisting of 363-bp amino acids from 126 S53 family genes were obtained from Gblocks 0.91b.

>ACLA_055020.t1

VFEPQGWLQIALQQDVDGFEKALSDPSHASYGKHFMKRMLLPSQESVQSVRSWLDWINFVGVANTLLDADFKWYRTLSYSIPESLASHVHMIQPTTRFTPQCLKSLYSKVAFASFLEEYARYDDLAKFEFSVIQYGGNDQEANLDLQYMLGISAPTEFSTGGRNEPLEFLQNVLKMPQHKLPQVISTSYGEDEQIPYALSVCNLYAQLGSRGVSVIFSSGDSGVGCQTNDGFPPQFPAACPWVTSVGGEEASSGGFSDLWPRPGYQAVRGYLLYNPRGRAFPDVAAQAYAVFDKGYAGTSCSAPAFGGIIGLLNDARLRAHKPPMGFLNPWLYFNDIVVGGCSWNATEGWDPATGLGTPFGKL

>AFL2T_01290

VVEPDGWLQLAVPEKITEFEQRVSTPGNQNYGRHMVRNFLYSSDVAPRKVLSWLGWITFVSQAEQLLRTRFYTFRTLKYSVPKELRSFVQMIQPTTRFTPSCLRKLYNRLGVSGFLDQYARYSDFHQFLYTVELIGGNLQEASLDIQYAAALADTTFYSTAGRHEPLEQLRYLLDLPDDELPAVLTTSYGELEQVPYARTTCNMFAQLGARGVSVIFSSGDSGVGCITNDGFQPLYPASCPFVTSVGGEMASTGGFSEYFPRPSYQSVNQYLLYNPNGRAIPDVAAQAFIIMDHGTGGTSAAAPVFAGIVSRLNAARLESNKPRLGFLNPWLYFTDIVDGGCSWNATPGWDPVTGLGTPYKAL

>AFL2T_05009

AFEPKGWLKIALQKDAAGFEKTVSDPDHPSYGQHFMKRMLLPRDDTVDAVRQWLDWINFVDTANKLLNAQFKWYRTLQYDVPESVTPHINTIQPTTRFTPTCLKELYSKIAFASYLEEYARYADLENFEFSVTTFGGNDQEANLDLQYILGVSAPTEFSTGGRNEPLEFFQNVLKLDQKDLPQVISTSYGENEQIPYARTVCNLIAQLGSRGVSVLFSSGDSGVGCMTNDGFPPQFPAACPWVTSVGAERGSSGGFSDYWPRPEWQAVSSYLLYNSSGRAFPDVAAQGFAVYDKGFDGTSASAPAFSAVIALLNDARLRAGKPTLGFLNPWLYLQDITLGGCSWNATQGWDPVTGLGTPFAEL

>AFUA_3G08930.t1

VVEPDGWFRLAMQERAAEFERRVSTPGHSSYGQHMVREFLRPPEEVSDKVLSWLNWVTFVSQAERMLRTRFYAFRTLAYSVPHDVHRYIQMIQPTTRFTPNCLRELYNRLGVSGFLDQYARYDDFENFMFTVVSIDGNLQEASLDVQYAYSLAYKTYYTTGGRNEPLDQLHYLLDLPDEELPAVLSTSYGEDEQVPYSNATCNLFAQLGARGVSIIFSSGDSGVGCITNDGFLPVFPASCPFVTAVGGEKASSGGFSDHFPRPSYQSVQGYLLYNPSGRGFPDVAAQAFVVIDHGVGGTSASAPVFAAIVSRLNAARLEDGLLKLGFLNPWLYFTDIIDGGCSWNATPGWDPVTGLGTPYNTL

>AFUA_4G03490.t1

VFEPQGWLQIALQHDVEGFETALSDPYHPNYGKHFMKRMLLPTQEAVESVRGWLDWIKFVGVANDLLDADFKWYRTLAYSLPQSVASHVNMVQPTTRFTPQCLKDLYSKVAFASFLEEYARYDDLAKFEFSVIQYGGNDQEANLDLQYIVGVSSPTEFSTGGRNEPLEFLQNVLKMDQDKLPQVISTSYGEDEQIPYARSVCNLYAQLGSRGVSVIFSSGDSGVGCLTNDGFPPQFPAACPWVTSVGGEEASSGGFSDLWERPSWQAVKRYLLYNPKGRAFPDVAAQAYAVFDKGFDGTSCSAPAFSAIVALLNDARLRAHKPVMGFLNPWLYFNDIVKGGCSWNATDGWDPATGLGTPFGKL

>An11g01110.t1

VHEPSVWMRIGLQSNLDRGHDLLSHPQSSRYGKHLVHDLFAPSNEAVETVRTWIQWLQFASEVEQLLQTEYYIYTCHEYHVPETIQSHIDYITPGVKMTPDCIRAMYNELGIFEDLGDIYSQDDLNLFFPTLDSIGAAPTESDLDFQIAYPIIWPILYQTDNYLDPPDPSPGPKQCGVYTPTNVISISYGSPEALPYQRRQCHEFMKLGLQGISVVVASGDSGVACFGDADFVPDFPATCPYLTAVGGEIAPSGGFSNIYARPSYQSVETYFVYNRIGRGYPDVSAIAIIIYNQGVGGTSAAAPAFAAMLTRINEERLAKGKSTVGFVNPVLYFRDVTVGGCGFPVAGGWDPVTGLGTPFEDL

>An14g02470.t1

IFEPNGWLQIALQHDVAGFEQAVSTPGHADYGKHFMKRMLLPSETAVDSVRDWLDWVKFVNKANALLDADFKWYRTLQYSIPDALVSHINMIQPTTRFTPHCLKQLYSKIGFASYLEEYARYADLERFEFSVVQFGGNDQEANLDLQYILGVSAPTEYSTGGRNEPLDFLQGILKLNNSDLPQVISTSYGEDEQIPYARTVCNLYAQLGSRGVSVIFSSGDSGVGCLTNDGFPPQFPASCPWVTSVGAEQASSGGFSDLWPRPSYQAVQTYLLFNASGRAFPDVSAQGYAVYDKGFDGTSCSAPTFSGVIALLNDARLRAGLPVMGFLNPFLYLNDIVNGGCSWNATTGWDPVSGLGTPFAKL

>An16g02250.t1

VVDPDGWFWLSMHEYKADFEQKVSTPGHRDYGRHMVMAFMRPSDQVSKIIFSWLDWVAFLAQAQSMMKTDFYNFRTLKYSVPEQVDAHLQMIQPTTRFTPICLRQLYNVLGISGYLDQYARYSDLDEFLFSVVSIGGNPQEASLDIQYALSMAFDTFYTTAGR---LEQLQYLVGLPDEDLPAVLSTSYGEDEQLPYTEATCNLFAQLGARGVSVIFSSGDSGVGCVSNDGFQPIFPASCPFVTSVGGEKASSGGFSERFARPSYQSVEAYLLYNPDGRGIPDVSAQAYVIRDHGTAGTSAAAPVFAAVISRLNAARLEQGKPTLGFLNPWLYFTDIVDGGCSWNATKGWDPVTGLGTPYQTL

>AO090005001380.t1

VVEPDGWLQLAVPEKITEFEQRVSTPGNQNYGRHMVRNFLYSSDVAPRKVLSWLGWITFVSQAEQLLRTRFYTFRTLKYSVPKELRSFVQMIQPTTRFTPSCLRKLYNRLGVSGFLDQYARYSDFHQFLYTVELIGGNLQEASLDIQYAAALADTTFYSTAGRHEPLEQLRYLLDLPDDELPAVLTTSYGELEQVPYARTTCNMFAQLGARGVSVIFSSGDSGVGCITNDGFQPLYPASCPFVTSVGGEMASTGGFSEYFPRPSYQSVNQYLLYNPNGRAIPDVAAQAFIIMDHGTGGTSAAAPVFAGIVSRLNAARLESNKPRLGFLNPWLYFTDIVDGGCSWNATPGWDPVTGLGTPYKAL

>AO090011000235.t1

AFEPKGWLKIALQKDAAGFEKTVSDPDHPSYGQHFMKRMLLPRDDTVDAVRQWLDWINFVDTANKLLNAQFKWYRTLQYDVPESVTPHINTIQPTTRFTPTCLKELYSKIAFASYLEEYARYADLENFEFSVTTFGGNDQEANLDLQYILGVSAPTEFSTGGRNEPLEFFQNVLKLDQKDLPQVISTSYGENEQIPYARTVCNLIAQLGSRGVSVLFSSGDSGVGCMTNDGFPPQFPAACPWVTSVGAERGSSGGFSDYWPRPEWQAVSSYLLYNSSGRAFPDVAAQGFAVYDKGFDGTSASAPAFSAVIALLNDARLRAGKPTLGFLNPWLYLQDITLGGCSWNATQGWDPVTGLGTPFAEL

>AOL_s00054g268p

TFEPNGWLRIHLQQNVPQFEKALSTPGHPSYGNHMIDDILRPHEDTAAAVRSWLDWIVVIGKAESLLDAKYKVFRTLSYSVPRNLHSSVTLIQPTTLFTPDCLANLYNKLGVNGFLEQYAQNDDLAKFLFTCTPIDGCTQEANLDIQYTVGASNPIYYSTAGRNEPLEWLEYMLNLSDEQLPQTITTSYGDNEHVPYAIKVCNMIGQLGARGVSVLFSSGDSGPGCTGKDGFVPTFPATCPFVTSVGGEKASSGGFSNYFSRPDYQAVSNYLYYNASGRAFPDISAQGFHVFVRGVSGTSASSPAFAAVISLVNNDRISGGKKPLGFLNPWLYIVDITSGGCGWKATTGWDPVTGLGTPFSVL

>ATEG_02150.t1

VFEPKGWLKIALQHDVEGFEQALSTPGHENYGKHFMKRMLLPSDSAVDAVQTWLDWINLVEHANALLDTQFGWYRTLQYSIPETVAAHINMVQPTTRFTPKCLKDLYSQVAFASYLEEYARYADMVKFQFSVVLYGGNDQEANLDLQTIMGLSAPTEYITGGRNEPLEFLQNILKLDQDELPQVISTSYGEDEQIPYAESVCNMLAQLGSRGVSVVFSSGDSGVGCQTNDGFNPQFPASCPWVTSVGAEQASSGGFSDFWKRPKYQAVAAYLLFNKGGRAFPDVAAQGYAIYDKGLDGTSCSAPAFSAIISLLNDARLREGKPTMGFLNPWLYLNDVVVGGCSWNATQGWDPVTGLGTPFAKM

>BDCG_03663T0

VLKPTGWVSIGLQHGWETLEQHLSDPSHPRYGKYLVNRLVRVAPTSSNLVTAWLDLISFIGVVERLFRANYAEYRSLTWSIPAGLVDHIDVVEPTNSFSIVCLSVLYNSIALVNFLGEVNNRSDIELFLFTTEIIDGDQQEGALDAQTILGLSWPTTYNVGSKNEPLAWLQY--MQTKETLPHVISISYADTERVPYARRVCREFAKLGARGVSILVASGDWGVGCVMDNGFAPSFPSSCPYVTSVGAEIVSGGGFSELFERPRYQAVENYLLFNRRGRGYPDIAALGFSVLWNGQDGTSASAPTVSAIIALVNDALLDNGHPPLGFLNPWLYFTDITWGGCGFPALAGWDPATGLGTPFPKL

>BDCG_03667T0

SLKPSKWLQIGLQGRLAELENRLSNPRHKRYGQFLVARLLRPHRDAVDRTQAWLDWLAVIAEAERLLDTKYHIYRAMEWSVPQYLHDAIDTIQPTTSLTSLCLRTLYTKMALVNYLGEFNNRSDVSHFLFEDISIGGNQQEGNLDAEVMLGIAHPTIYSVGKSNEPLVWLNWILDQPDSELPSVVSTSYGDIEHVPYARRVCNGFAQLGARGVSVIMGSGDYGVGCYSNDGFLVSFPDSCPWVTSVGAEVVSSGGFSNYFPRPDYQSIARYLMFNPYGRAIPDVSAQGFVTIWNGIDGTSASAPTFAAIVALVNDALAAEKKPPLGFLNPWLYFQDVTEGGCGFPALQGWDAASGWGTPFPKF

>Bfuc_05765.t1

VHEHPRWMRIGLQNNLDKGHEHLSDPASSHFGQHWVIEAFRPSQQTEDEVRKWLGWFAFAEEAENLLYTEYHEYSCEAYHVPKDIRKHIDYISPGIKLTPACVAALYNSMGIFESELQFYTQNDLDLFFPIPANIGGQSTEANLDIQLAYPILYPTLYQVD--FNTLDALDGSLMCGTFTPTNVISLSYGGQESLPYQKRQCLEYMKLGLQGVSFLFASGDSGVSCL---GFNPTWPGTCPYVTSVGAESASSGGFSNIYPIPDYQFVAKFFIYNRIGRGIPDVAANGIAVYNGNSGGTSASTPIFSAVINRINEERINVGKGPIGFLNPSLYLNDIVNGGCGFSAVPGWDPVTGLGTPYPKM

>Bfuc_12776.t1

AVKPAQWLQIGLQGQFDELERHLSDPDHHRYGQHLVNDLVKPSDETHNLVHEWLDWIKVIEDIESLLDTEYSTYRTPEWSLPLHLHDHIDTIQPTNSFTPLCLRTLYNKMALTNYLGESNNRSDTKLFLFDVQVIGGNDQEGNLDSETMLGIGYPIAYTTGGSNEPLTWLQYVLSQKNL--PGVVSNSYQDTEQVPYAVSVCKGFAQLGARGVSVLFGSGDNGVGCISNVDFLAMFPSTCPYVTSVGGEVVSGGGFSRYFPRPSWQALKPYLFFNATGRGFPDIAAQGYITVWNGLDGTSAATPAASAILALINDALIAAGKPTLGWLNPWLYFTDVTIGGCGFPAATGWDAATGFGTPFPKI

>CHGG_07661.t1

VFEPAGWLRIALQPDEALFERTLSDPAHARYGQHLVASLLAPRAESTAAVLAWLEWVNVVRQAAALLEADFGVWRALRYSVPDEVAPHIRMVAPVVRFTPECLRALYSLFGVAGYLEEWAKYDQLELFSFTAVGVGGNEQEANLDIQYAVALSYKTYYSTAGRNEPLDFFSYLLKLPDQKLPQTLTTSYGEDEQVPYAEKVCQMIGQLGARGVSILFSSGDTGVGCQTNDGFLPIFPAACPYVTSVGGEQASSGGFSDIWPRPSYQAVSKYLLYNPEGRGFPDVAAQGYHVFSQNVSGTSASAPMFAALISLLNNARLAEGRPPLGFLNPWLYLTDIVHGGCSWNATPGWDPVTGLGTPFDKL

>CIMG_00167.t1

LLEPDGWMKINLASQTEDLHQKVGTPGHARYGLHLINSLMTPNEVVLNDVLKWINWIDIIGAASKMLNARFYEYRTTEYSAPKSVAQHIFYIYPLILFTPNCLRGLYNKIAVSGYLDQYAQYKDLAAFLFSVSLVGGNIQEANLDTQYAVALTYNDFVSVKGRNEPMDQLEYLMGLPDKDLPTVLTTSYGETEQVPYARATCNEFAKLTARGVSIIFSSGDTGVGCTSNDGFNPIFPASCPFVTAVGGERASAGGFSNYFKRPGWQAVTKYLYYNPLGRGFPDVAAQAYPIYEKGAAGTSASAPTIAAIIAHLNEVRLSQGKPVLGF------FTDITHEGCSWNATKGWDPVTGFASKLNVL

>CIMG_02188.t1

LAEPDGWLKVSVAKDPNLLHRSLSTPNHPRYGQHMVRDIVAPHPEASDGIMAWLDWIDFVDTAERLLNTRFYHFRALEYSLPSSLSKHVRTVQPTTFFTPSSLRELYNLLGVSGYLEQYARYSDLNAFIFSVELYGGNDQEASLDIQYTVGLTYNTYYSGGGRNEPMEQLKFFANLSDSRLPTVLSTSYGENEQVPYAKAVCDEFAKLGARGVSVIFSSGDSGVGCLTNDGFNPIFPAACPYVTSVGGESASSGGFSEIFPRPSYQSVDAFLYFNRNGRGFPDVAAQGYAVYDHGVGGTSASAPLIASVISNLNEVRLSQGKPVLGFLNPWLYFTDIVDGGCAWDAVEGWDPVTGFGTPFKKL

>CIMG_03887.t1

VVEPETWLHLAMNDVLDQFEQRVSTPGNEHYGEHMVQAFLQPPSYTSDAVLAWLDWLHFVKKAEELFNTQFYYYRTLEYSVPKIIAPYVHMIQPTTKFTPDCLRDLYNKLGISGYLEQFARYDDFARFLFDVVSIGGNDQEASLDVDYAIGLSG-VYYTTAGRNEPLDQLHYLLSLPDDQLPSVLSTSYGENEQVPYTDMTCNLFARLGARGVSVIFSSGDTGVGCQTNDGFLPVFPAACPFVTSVGAERASSGGFSDRYRRPWYQAVGHYLLYNPAGRGFPDVAAQGFSVVDHDVSGTSASAPVFAAIVANLNSIRQAKGKPVLGFLNPFLYFTDIVHGGCSWNATKGWDPVTGLGTPFEVL

>CPAT_00674

LAEPDGWLKVSVAKDPNLLHRSLSTPNHPRYGQHMVRDIVAPHPEASDGIMAWLDWIDFVDTAERLLNTRFYHFRALEYSLPSSLSKHVRTVQPTTFFTPSSLRELYNLLGVSGYLEQYARYSDLNAFIFSVELYGGNDQEASLDIQYTVGLTYNTYYSGGGRNEPMEQLKFFANLSDSRLPTVLSTSYGENEQVPYAKAVCDEFAKLGARGVSVIFSSGDSGVGCLTNDGFNPIFPAACPYVTSVGGESASSGGFSEIFPRPSYQSVDAFLYFNRNGRGFPDVAAQGYAVYDHGVGGTSASAPLIASVISNLNEARLSQGKPVLGFLNPWLYFTDIVDGGCAWDAVEGWDPVTGFGTPFKKL

>CPAT_02756

LLEPDGWMKINLASQTEDLHQKVGTPGHARYGLHLINSLMTPNEVVLNDVLKWINWIDIVGAASKMLNARFYEYRTTEYFAPKSVAQHIFYIYPLILFTPNCLRGLYNKIAVSGYLDQYAQYKDLAAFLFSVSLVGGNIQEANLDTQYAVALTYNDFVSVKGRNEPMDQLEYLMGLPDKDLPTVLTTSYGETEQVPYARATCNEFAKLTARGVSIIFSSGDTGVGCTSNDGFNPIFPASCPFVTAVGGERASAGGFSNYFKRPGWQAVTKYLYYNPLGRGFPDVAAQAYPIYEKGAAGTSASAPTIAAIIAHLNEVRLSQGKPVLGFLNPWIYFTDITHEGCSWNATKGWDPVTGFGTPFKKL

>CPAT_09576

VVEPESWLHLAMNDVLDQFEQRVSTPGNEHYGEHMVQAFLQPPSYTSDAVLAWLDWLHFVKKAEELFNTQFYYYRTLEYSVPKIIAPYVHMIQPTTKFTPDCLRDLYNKLGISGYLEQFARYDDFARFLFDVVSIGGNDQEASLDVDYAIGLSG-VYYTTAGRNEPLDQLHYLLSLPDDQLPSVLSTSYGENEQVPYTDMTCNLFARLGARGVSVIFSSGDTGVGCQTNDGFLPVFPAACPFVTSVGAERASSGGFSDRYRRPWYQAVGHYLLYNPAGRGFPDVAAQGFSVVDHDVSGTSASAPVFAAIVANLNSIRQEKGKPVLGFLNPFLYFTDIVHGGCSWNATKGWDPVTGLGTPFEVL

>Enid_FGSC_A4_AN7159.2.t1

AFEPDGWLRIALQHNVAGFEQALSTPGHSSYGQHFMKQLLLPTEEASSSVRDWLDWINFVDQANALLDADFLWYRTLSYSVPSELAGYVNMIQPTTRFTPSCLADLYSKVAFASFLEEYARYDDLAEFEFSVISIGGNDQEANLDLQYIIGVSSPTEFTTGGRNEPLDFLEAVLKLDQKDLPQVISTSYGEDEQIPYARSVCNLYAQLGSRGVSVLFSSGDSGVGCQTNDGFPPQFPASCPWVTAVGGESGSSGGFSDYWARPAYQAVESYLYFNRSGRAFPDVAAQAFAVVDKGFDGTSCSSPVFAGIVALLNDVRLKAGLPVLGFLNPWLYLNDIVDGGCGWNATEGWDPVTGLGTPFAKL

>EXU96178

VIHTSRVVRIALQRNLDKGMEYLSDPSSKNYGSHYVVDLFSPAPESIETVKRWLGWLDFVGQLEDILKTNYHLYGADSYSLPSEVSQHVDFITPGVVPTPACIKALYNQLGMFESDNEMHKQSDLDQFYPKIDLIWGTKPEAALDFDVSIPVIYPELYQTKSNDDPVDGVTANEACGTFTPANVISFSYGLTENWP-TQRQCDEFMKLGLQGSSIVFASGDGGVACLGSNGFNPASPSSCPYVTSVGAESAGSGGFSNIWPSPDYQAVASFFIYNRAGRGFPDIAAIGGVIVLNGTGGTSMSAPIVAAIFTRVNEVRLKAGKKPIGFANPALYFKDVTLGACGFSAVKGWDPVTGLGTPFPAV

>EXU99937

QHEPQGWLSIALQPEIHRLASKF-------GSGHLVRTLRAPDPKDAAAVVDWLDWIHVVSTAESLLNTQLQRYRAREYSVPSHLSDAISFINPISNFTPKCLRQLYVRLGVSGYLEEHSNHADVRDFLFKVELVGGDPQEAQLDLEYVMGLGFPTYYATGGRNEPLEFIQALLDKPDNEVPHVLSVSYGDDELVPYAERVCGMLGLLTKRGTSIIHSTGDGGSACRTKDGTMSTFPASCPWVTAVGAPSGSSGGFSQYFERPAWQAVDKYVYYNASMRAVPDISAVGFRVIVGALEGTSASAPVFAAMISLVNDARLRKGKPSLGWLNEILYLQDITKGSCGWPAKQGWDAITGLGVPFAKF

>EXV03254

IHESGRWVRIALQKNLDKGMDYLSDPSSAKYGQHYVVELFAPDESSINAVRSWLGWVDFVGQLESILKTKYHMYGTDEYSLPNEISDLVDFITPAVVMTPACIKSQYNRMGIFEISEDVYSQEDLDSFYPKIDLIGSAPVESDLDFEIAIPIIYPELYEAANDDDPVDGNTPNEMCGTFKAANVISFSYGTAEAYPYLQRQCDEFMKLGLQGTSIVLSSGDDGVACLGPKGFTPGQQASCPYVTSVGSEIASSGGFSNIWSTPDYQAVSSYFIYNRVGRGYPDVAALGAVVVVNGSGGTSMSAPLVGAILTRINEERIKAGKKSVGFANPALYFTDVVRGACGFSAVEGWDPVTGLGTPYPAM

>EXV04474

-LEPDGWFSIALQPEMHGLASKI---------DGLTRSLRTPAQDDVDHVMEWLDWIRVVGEANNLLDMQLRRYRAPEYNIPDSLDTAIDFIHPIANFTPDCINKLYIRFGIAGFLEQWANYDDTRRSFFTVELIGGNQQEANLDIQFGMAVGYPIYYSTGGRNEPLDFFHHLSSKKNEELPHVLSISYADDELVPYAIRVCNEIGMLASRGVSVLSGSGDGGAKCRSNDGTISTFPASCPWVTSVGANGSSSGGFSAYFKRPDWQAVSEYIYYNSSMRAVPDISAIGFQTVINGLDGTSASTPVLAGMIALVNDARVRQGKPVLGWLNKRLYLQDIKAGSCGWPATEGYDAITGLGVPFNRF

>FGST_02821

KVEPNGWLSIAMQPDIENLKTGLDATSGQYIQRHLALALRTPDKKDVDKVLAWLDWIHVVKDAQDLLDAKIGFYRTRDYSLPESLVNSISFIHPIANFKPDCLREQFIRFGIAGFLEEYANYEDAQDFLFSVQLIGANSQEAALDVQYAMALGYPTYYLADGRNEPLEFLDYLLDLSDDEVPHVLSVSYGDNEVVPYAERVCSMFGLLTARGTTILAASGDGGAKCRTNDGTMAVFPATCPWVTSVGGFEGSGGGFSQYFPREKWQSIKSYVNYNASNRGVPDISMPAYITRLKGLRGTSASTPVMAAMIALINDARVRKGKNVLGWINEVLYLSDVTAGPCGWPAAKGWDAITGLGVPFQKL

>FOXT_10424

KVEPVGWLSIALQPDIDNLKTNLDCTSGKYTQNHLALALRDPDQKDVGQVLGWLDWIHVVKAAEDLLDTKIGFYRTKQYSVPESVADAVNFIHPIANFTPDCLREQYIRFGIAGFLEENANYQDSNDFLFSVQLIGANSQEAALDVQYGMALGYPTYYLADGRNEPLEFLDYLLDLSDDEIPHVLSISYGDNEVVPYAERVCSLFGLLTARGTTIVAASGDGGAKCRTNDGTMSVFPATCPWVTSVGGFEGSGGGFSQYFPREKWQSIKSYVHYNASNRGVPDISISAFITRLKGLRGTSASAPVVAAMLALVNDARVRKGKEVLGWLNEVLYLQDVTNGSCGWPAAKGWDAITGLGVPFQKL

>fsol_81544

VVEPDGWLSIAMQPDIDNLKTRLRDTSGKFTQNHLALALRDPDQADIDEVLTWLDWIHVIEAAEDLLEMKIGFYRTREYSVPESVADAISFIYPIANFVPECLHKLYIRLGIAGFLEEYANFQDSDEFLYSVELIGGNSQEAALDVQYAMALGYPIYYLAGGRNEPLEFLDYLLDLSDDEIPHVLSISYADNEVVPYAERVCSLFGLLTARGTSVLAASGDGGAKCHTNDGAMSVFPATCPWVTSIGGPVGSGGGFSQYFLREKWQDIESYVFYNASYRAVPDISAVSFITKVAGVRGTSASTPVVAAMIALINDARVRKGKDVLGWLNEVLYLQDITGGSCGWPAAKGYDAITGLGVPFEKL

>FVET_03498

IHEPGDYLRIYL-ADLEKPYLKVSDPNSKQYGQYLLRSMLPDKSLASTKVAGWLERLDVIADWNRVLNTTFHRFRTTKYSIPSTQQDTISYIFPTIHFCPSYLRTKYSTFAIAGFLNNFPNITDVRAFLINTVSVKGPPVEAELDLDYSMAFTGPTFYSVGGHNEPAEFFDYVLGLKSP--PKVISISYNDDEKVPYAQHVCDLFAKAAARGISIIGSSGDGGASCLGVSGFVPTFPSSCPWMTSVGA--ASSGGMSNIFKRPSWQAVSGYILYNVSGRAQPDVSLLGYLTLTGGHDGTSASAPVFAAMVALTNDIRLREKKPALGFLNPLLYFRDIKDGGCGWEALAGWDAATGLGEPFTKL

>FVET_09088

KVEPVGWLSIALQPDIDNLKTSLDCTSGKYTQNHLAFALRDPDQKDVGQVLGWLDWIHVVKAAEDLLNTKIGFYRTNQYSVPESVANAVNFIHPIANFTPDCLREQYIRLGIAGFLEENANYEDSNDFLFSVQLIGANSQEAALDVQYGMALGYPTYYLADGRNEPLEFLDYLLDLSDDEIPHVLSVSYGDNEVVPYAERVCSLFGLLTARGTTIVAASGDGGAKCRTNDGTMSVFPATCPWVTSVGGFEGSGGGFSQYFPREKWQSIKSYVHYNASNRGVPDISISAFITRLKGLRGTSASAPVVAAMLALVNDARVRKGKEVLGWLNEVLYLQDVTKGSCGWPAAKGWDAITGLGVPFQKL

>HCAG_04281.t1

LKEPSKWLQIGLQVRLKELEKRLSNPLHKRYGKFLAAKLLHPGQDAINRTQAWLDWLRVIAEAERLLDTKYFIYRAMEWSLPQSLHDVIDTIQPTTSFTPLCLRTLYTRMALVNYLGEFNNRSDISQFLFEDISIGGNQQEGNLDAEVMIGIAHPTTYTVGESNEPLTWLNWILDQPDSELPSVVSTSYGDIEHVPYARRVCNGFAQLGARGVSVIMGSGDHGVGCYSNDGFLVSFPDSCPWVTSVGAEVVSSGGFSNYFPRPAYQSVARYLMFNPYGRAIPDVSAQGYVTIWNGVDGTSASTPTFAAVVALVNDALAAEDKPPLGFLNPWLYFRDINEGGCGFPALHGWDAASGWGTPFPKF

>HCAG_04284.t1

LIASSSWVCIGLQHGWETFEQHLSDPSHPRYGKHLVSLLVKAAPESLNAVTTWLDLVSFIEVVERLFMAKYAEYRSLSWSVPADLVNHIDVVEPTNSFSIVCLSVIYNSISLVNFLGEVNNRSDIDLFLFTTEIIGGTQSEGALDAQTILGLSWPTTYNVGSKNEPLAWLQY--MQSKETLPHVISISYADTEQVPYARRVCKEFAKLGARGVSIIVASGDWGVGCVLDNGFAPSFPASCPYVTSVGAEIVSGGGFSELFSRPRYQAVENYLLFNRKGRGYPDVAALGFSVLWNGQDGTSASAPTVAAIIALVNDALLDKGHPPLGFLNPWLYFTDITWGGCGFPALTGWDPATGLGTPFPKL

>Jan02137

VKEPPGWLRIALQPHFNILEKHLSDPDHPRYGAYLVEKIIAPHPASLEAVNTWLDWVNVVALAERILQTEYHVWRTTSYSLPEDLHDHIELIQPTTIFTISCLKQLYTSIGVTGYLEEFANEQDLQSFFFKFVSVGGNNQEANLDVQFAFGLAYPTFWSTAGRNEPLDWVNFVLSQKDV--PHAISTSYGEPEQ------TGRQLAQLSARGVSLMFSSGDGGVGCFTNDGFLPNFPATCPYVTAVGGETASGGGFSDYFRRPNYQVVPAFLLYNREGRGLPDVAAQGFRVWYRGIGGTSASAPAFTAIIALLNDARIAKGMPPLGFLNPLLYFNDITVGGCGFNATKGWDP--RLGTPFIRL

>KFG78015

VIHTSRVVRIALQRNLDKGMEYLSDPSSKNYGSHYVVDLFSPAPESIETVKRWLGWLDFVGQLEDILKTNYHLYGADSYSLPSEVSQHVDFITPGVVPTPACIKALYNQLGMFESDNEMHKQTDLDQFYPKIDLIWGTKPEAALDFDVSIPVIYPELYQTKSNDDPVDGVTANEACGTFTPANVISFSYGLTENWP-TQRQCDEFMKLGLQGSSIVFASGDGGVACLGSNGFNPASPSSCPYVTSVGAESAGSGGFSNIWPSPDYQAVASFFIYNRAGRGFPDIAAIGGVIVLNGTGGTSMSAPIVAAIFTRVNEVRLKAGKKPIGFANPALYFKDVTLGACGFSAVEGWDPVTGLGTPFPAV

>KFG80701

QHEPQGWLSIALQPEIHRLASKF-------GSGHLVRTLRAPDPKDAAAVVDWLDWIHVVSTAESLLNTQLQRYRAREYSVPSHLSDAISFINPISNFTPKCLRQLYVRLGVSGYLEEHSNHADVRDFLFKVELVGGDPQEAQLDLEYVMGLGFPTYYATGGRNEPLEFIQALLDKPDNEVPHVLSVSYGDDELVPYAERVCGMLGLLTKRGTSIIHSTGDGGSACLTKDGTMSTFPASCPWVTAVGAPSGSSGGFSQYFERPAWQAVDKYVYYNASMRAVPDISAVGFRVIVGALEGTSASAPVFAAMISLVNDARLRKGKPSLGWLNEILYLQDITQGSCGWPAKQGWDAITGLGVPFAKF

>KFG81969

IHESGRWVRIALQKNLDKGMDYLSDPSSAKYGQHYVVELFAPDESSINAVRSWLGWVDFVGQLESILKTKYHMYGTDEYSLPNEISYLVDFITPAVVMTPACIKSQYNRMGIFEIFEDAFSQEDLDSFYPKVDLIGAAPVESDLDFEIAIPIIYPELYEAANDDDPVDGNTPNEMCGTFKAANVISFSYGTAEAYPYLQRQCDEFMKLGLQGTSIVLSSGDDGVACLGPKGFTPGQQASCPYVTSVGSEIASSGGFSNIWSTPDYQAVSSYFIYNRVGRGYPDVAALGAVVVVNGSGGTSMSAPLVGAILTRINEERIKAGKKSVGFANPALYFTDVVRGACGFSAVEGWDPVTGLGTPYPAM

>KFG84385

-LEPDGWFSIALQPEMHGLASKI---------DGLTRSLRTPAQDDVDHVMEWLDWIRVVGEANKLLDMQLRRYRAPEYNIPDSLDTAIDFIHPIANFTPDCINKLYIRFGIAGFLEQWANYDDTRRSFFTVELIGGNQQEANLDIQFGMAVGYPVYYSTGGRNEPLDFFHHLSSKKNEELPHVLSISYADDELVPYAIRVCNEIGMLASRGVSVLSGSGDGGAKCRSNDGTISTFPASCPWVTSVGANGSSSGGFSAYFKRPDWQAVSEYVYYNSSMRAVPDISAIGFQTVINGLDGTSASTPVLAGMIALVNDARARQGKPVLGWLNKRLYLQDIKAGSCGWPATEGYDAITGLGVPFNKF

>KFG84515

VHEHQQWVRIALQSNLEKAEDYLSDPASPKYGQHFIVDLFAPSEQTINQVKTWLGWINFASELESILKTKFYLYGTESYSLPHDVSSLVDFVMPGISFTPRCISALYNTLGIFETEGDVYSQEDLNQFYPIIHLIGANPPESDLDFEIAIPIIYPSLYQIQGNEGS------GKECNDLTPPNVLSVSWGDSEDQPFAQRQCTEWMKYGLQGTSVFVASGDYGVACLGPKQFVPDGLCSCPYITAVGSEVASSGGFSNIFATPEWQAVSEYLIYNRGGRGYPDISAIGGVVVVGGIGGTSMSAPLVAAIFNRINEERLNIGKSPVGFINPALYFNDITKGGCGFSAASGWDPVTGLGTPYTQL

>lbic_185466

---------------MDDFIAHSSDPDHVRYAQHLVADFMTPHSSSVEAVESWLEWVTLVAEAERMLGAKYHIFRTLAYSLPRELHRHIDVVAPTTYFTPACLRALYNKIGIAGYLNEFASYSDLGTFFFVTVQVNGDDQEANLDIQYTTAMTFPTYYSTGGSNEPLDWLDFILTQDTI--PQVISTSYGDNEQVPYAETVCKRLAILGARGTTVLFSSGDYGVGCKTNDGFQPSFPASCPFVTTVGGEVASGGGFSRYFPQPSYQAVDGYLLFNRSGRAYPDVAAQAFQVVVGGIGGTSASCPTVASVFTLLNDYRLSLGKRSLGFINPLLYFNDIVAGGCGFSAGKGWDPVTGLGTPFLKL

>lbic_191088

DLAPKGWLRIGVQAGMEDLIANLSDPGHGRYAQHLVEAFAKPHPDSTEAVSSWLDWVTVVAQAERMLGTKYNVYRTMGYSLPRELHSHIDVVAPTTYFTPTCLRDLYNKLGVAGYLGEFASTSDLQTFFFSIEQVGGNDQEANLDIQYTIGMSFPIYYSTGGSNEPLDWLNFILAQKTI--PQVITTSYGDDEQVPYAVKVCNMFAQLGSLGTTVFFSSGDFGVGCKTNDGFQPAFPASCPFVTAVGGEVASGGGFSQYFTQPSYQAVSAFLMFNQTGRAYPDLAAQGFQVVVSGVGGTSASSPTVAGVFSLLNDFRLSKGKTSLGFINPLIYFNDITSGGCGFTAVKGWDPVTGLGTPFGKL

>lbic_248220

LHEPSGWLRFGLQSSINKLEDMLSHPDSSNYGNHWVTQTFAPSQETVEIVRGWLNWLEVVEEAEHLLKTEYHVYACSEYHLPIYVSPHVDLVNPSIHFTPICLRALYNTFGIVEYTPQAYLQSDLDLFFPEFVSIGGTMQESNLDLQYAMNLVNPVLYQVGPQDDPQDEIFPAANCGTTKPANVISTSYGYNEALSYTLRQCAEYAKLGLMGVTVLFSSGDNGVACLNVDGFNPGFPSTCPFVTSVGAEVASGGGFSNYFGIPDYQAVGKYLIWNSTGRGYPDISANGYVVAVDGVFGTSASTPVVGSILAMVNDARLTIGKKPIGFINPAIYFHDITSGGCGFSAVPGWDPVTGLGTPFAKL

>MCYG_00184T0

VVEPEGWFWLAIRENPEQLYDTISTPGRARYGKHLLDDLLRPRVETSEGIISWLDWIRFVKTAEQLMKTQFHVFRTLEYSVPASISSHVQMIQPTTLFTTACLRELYNRIGVSGFLEEYAQYRDLDLFLFSEGLIGGNTQEANLDMQYVVGLSHKTYYSTAGRNEPLEQLRYLVKLPKNQLPSVLSTSYGDTEQLPYTKATCDLFAQLGTMGVSVIFSSGDTGPGCQTNDGFNPIYPASCPFVTSIGGERASSGGFSDRFPRPQYQAVKGYLLFNPNGRAFPDIAAQGYAVYDKGVSGTSASAPAMAAIIAQLNDFRLAKGSPVLGFLNPWIYFTDIVDGGCSWNATKGWDPVTGFGTPFQAL

>MCYG_01559T0

VHEPVWWLTIALQQNLEHAEDYLSDPSSPQYAQYWVAAKFAPSEPTARKVMSWLGELYMAQEAEKLLHTTFYIYICEKYSVATFVEKYVDFITTTEQFTPDCLRALYNSLGIIEFTWVGYLESDLDKFFPKFESIGGIQTEADLDIEYAMALTHPTNYQVGDIYDPPDPTPASSDCGTHKPTKVISISYAYEEGFSYERRQCLEYLKLGLQGVTVVFASGDHGTACGNTVIYVPTFPSTCPYVTSVGGDAESAGGFSNVFAVPGYQATRDYLLNTGFGRGFPDVAANAYATAVNGVYGTSASAPVFASVIAWINDARLNMGKQPVGFVNPVLYLNDVAKGDCAYHASSGWDPVTGLGTPFDRM

>MCYG_05446T0

VLEPQGWFKLALQDKAAAFEQHVSNPKHENYGKHMVDAFLQPPAHMTDSVFNWLDWLTFVQKAEKLLNTRFYNFRTLQYSVAETVAPYVHMIQPTTKFTPDCIRDLYNRLGISGYLEQYARLDDFSTFIFDFKSIGGNEQEASLDVDYAIGLS-GTYYGTAGRNEPLEQLHYLLGLPDSELPAVLSTSYGENEQVPYTDSACHLFARLGARGVSVIFSSGDTGVGCQSNDGFNPIFPAACPFVTSVGGEVASSGGFSERFSRPWYQDVNHYLMYNPSGRGFPDVSAQSFATRDHGVSGTSASAPLFAGVVSILNSIRLAHHKPRLGFLNPWLYFTDIVHGGCSWNATRGWDPVTGLGTPFETL

>MCYG_06077T0

TVEPEGWMNLALQEKAHAFEQMVSTPGHSNYGKHLLKDFLRPRKEVSDSILSWLDWIHFVSQAERMLKTRFHYYRTLQYSVPSHLAPDIHMIQPTTKFTPQCLRDIYNVIGVSGYLDQYARYNDFYKFIFSVKYIKGNLQEASLDIDYALGLSN-VFYTTSGRNEPLDQLHYLLSLPSDELPAILSTSYGENEQVPFSNATCSLFAQLAARGVSVIFSSGDTGVGCLTNGRFNPTFPASCPFVTSVGAEMASSGGFSDRHIRPRFQAVLTYLLYDPRGRGIPDVAAQGFAVYDHGVSGTSASAPAFAAIIANLNSIRLNANKPVLGYLNPFIYFTDIVHGGCSWNATEGWDPVTGVGTPFEIL

>MGG_07404.t1

VFDPRGWLRIALQQNAAALEQVVSNPRHANYGQHLLRSYTAPTPRAVRSVTSWLDWVTLVGAADRLLGADFAWYRTLSYGVDDSVAPHVDLVQPTTRFTPLCLRTLYNLVAFASFLEQYARYSDQQAFTFSVETVGGNDQEANLDLQ----------------NEPLEFLTYLLAQPDSAIPQTLSVSYGEEEQVPYAIKVCNMFMQLGARGVSVMFSSGDSGPGCVRASDFGSTFPAGCPYVTSVGSERASSGGFSIYHARPDYQVVPKYIFFDGNGRGIPDVAAQGFVVIDKGISGTSASSPAFAGMVALVNAARKSKDMPALGFLNPMLYMTDIVNGGCRFNATAGWDPVTGLGTPFDKL

>MGYG_00757T0

VVEPEGWFWLAIRENPEKLYDTISTPGRARYGKHLLDDLVRPRAETSESIVSWLDWIKFVKVAEQLMKTQFHVFRTLEYSVPAAISAHVQMIQPTTLFTTACLRELYNRIGVSGFLEEYAQYRDLDLFLFSVGLIGGNTQEANLDMQYVVGLSHKTYYSTAGRNEPLEQLRYLVKLPKDQLPSVLTTSYGDTEQLPYTKATCDLFAQLGTMGVSVIFSSGDTGPGCQTNDGFNPIYPASCPFVTSIGGERASSGGFSDRFPRPQYQAVKGYLLFNPNGRAFPDIAAQGFAVYDKGVSGTSASAPAMAAIIAQLNDFRLAKGSPVLGFLNPWIYFTDIVDGGCSWNATKGWDPVTGFGTPFQAL

>MGYG_06500T0

VVEPQGWFKLALQSKTAEFEQRVSNPRHADYGKHMVDAFLQPSSLAKESVLNWLDWLTFIENAEKLFDTHFYTFRTLKYSVPASAAPYIQMIQPTTKFTPDCIRDLYNRLGISGYLEQYARLEDFSTFIFDFKSIGGNEQEASLDVDYAIGLS-GTYYGTAGRNEPIEQLFYLLDLPDSELPAVLSTSYGENEQIPYTSVVCSLFGRLGARGVSVIFSSGDTGVGCQSNDGFNPIFPAACPFVTSVGGEVASSGGFSERFARPWYQDVRHYLLYNPSGRGFPDVAAQSFATRDHGVSGTSASAPLFAAVVSILNSIRLAHNKPKMGFLNPWLYFTDIVHGGCSWNATKGWDPVTGLGTPFEKL

>NCU04903.t1

VLEPAGWLSIALEPGIEELKRRLSTSDDHPNSRQFVEKHRQPDQRSVTAVGRWLSWITFAATVQMLFEADLAYYRSRSYTIPRWLSDDIDFVHPLTNFFPGCIRKLYVRFGIASFLEQYITHRDVTSFLITITLLNADPHEANLDVQYALSLGHPIYYATGGRNEPLEFLQALLALPDNQIPHVLSISYADDEQVPYAHRVCDLFAAVAARGTSVLVATGDGGAACIKNDGFVPTFPASCPWVTSVGALTGSSGGFSEYFDRPLWQAVDPYVVYSHNGRGMPDMAAIGFQIIHRGVRGTSASTPVVAAMVALVNDQRLRQGKRSLGWLNGHLYLTDVKWGGCGWDARKGWDPVTGLGVPFQEM

>NCU08418.t1

VKSPSGWLKIALAHQPDALETAISDPNHHEYGMHLVRSLVAPADETTDAVTSWLDWVSFVAKANNLLNTTFDWYRTLQYSVPDELDAHVDMIQPTTRFYPDEIRSLYNTIAFASYLEQYSNYDDFTSFAYTVKLVGGHDQEANLDLQYILAISNPREYSIGGRNEPLDFFQYLLSLKNSELPATLSTSYGEEEQVPYALKVCSMIGQLGARGVSVIFSSGDSGPGCIRNDGFEPTFPGACPWVTSVGGEKASSGGFSMYHKRPVYQVVKKYLFFDEQGRGFPDVSAQAYAVYVDGVSGTSASAPMFAGLVALLNAARKSHGLPSLGFINPLLYFTDIVNGGCKWNATEGWDPVTGLGTPFDKL

>NCU10306.t1

VKSPSGWLKIALAHQPDALETAISDPNHHEYGMHLVRSLVAPADETTDAVTSWLDWVSFVAKANNLLNTTFDWYRTLQYSVPDELDAHVDMIQPTTRFYPDEIRSLYNTIAFASYLEQYSNYDDFTSFAYTVKLVGGHDQEANLDLQYILAISNPREYSIGGRNEPLDFFQYLLSLKNSELPATLSTSYGEEEQVPYALKVCSMIGQLGARGVSVIFSSGDSGPGCIRNDGFEPTFPGACPWVTSVGGEKASSGGFSMYHKRPVYQVVKKYLFFDEQGRGFPDVSAQAYAVYVDGVSGTSASAPMFAGLVALLNAARKSHGLPSLGFINPLLYFTDIVNGGCKWNATEGWDPVTGLGTPFDKL

>NFIA_029950.t1

VFEPQGWLQIALQHDVESFETALSDPYHPNYGKHFMKRMLLPTQEAVESVRGWLDWVKFVGVANDLLDADFKWYRTLAYSLPQSVASHVNMVQPTTRFTPQCLKDLYSKVAFASFLEEYARYDDLAKFEFSVIQYGGNDQEANLDLQYIVGVSSPTEFSTGGRNEPLEFLQNVLKMDQDELPQVISTSYGEDEQIPYARSVCNLYAQLGSRGVSVIFSSGDSGVGCLTNDGFPPQFPAACPWVTSVGGEEASSGGFSDLWERPSWQAVKRYLLYNPKGRAFPDVAAQAYAVFDKGFDGTSCSAPTFSAIVALLNDARLRAHKPVMGFLNPWLYFNDIVNGGCSWNATDGWDPVTGLGTPFGKL

>NFIA_068200.t1

VVEPDGWFRLAMQERAAEFERRVSTPGHSSYGQHMIREFLRPSEEVSDRVLSWLNWVTFVSQAE----------RTLAYSVPDDVHRYIQMIQPTTRFTPNCLRELYNRLGVSGFLDQYARYDDFENFMFTVVSIDGNLQEASLDVQYAYSLAYKTYYTTGGRNEPLDQLHYLLDLSDEKLPAVLSTSYGEDEQVPYSNATCNLFAQLGARGVSIIFSSGDSGVGCITNDGFLPVFPASCPFVTAVGGEKASSGGFSDRFPRPSYQSVQGYLLYNPSGRGFPDVAAQAFVVIDHGVGGTSASAPVFAAIVSRLNAARLEDGLPKLGFLNPWLYFTDIVDGGCSWNATPGWDPVTGLGTPYNTL

>PADG_04152T0

---RPYW----------------SNPLHRRYGQFLAARLLRPQQHVIDRIQAWLDWLTIIAEAELLLGTKYSIYRTMEWSLPQDLHDAIDTIQPTTSFTPLCLRTLYTRMGLVNYLGEFNNRSDISQFLFEDISIGGNQQEGNLDAEVMIGIAHPTTYSVGGPNEPLAWLNWILDQPDSNLPSVVSTSYGDIEHVPYARRVCNGFAQLGARGVSVIMGSGDHGVGCYSNDGFLVSFPDSCPWVTSVGAEVVSSGGFSNYFPRPDYQSVSQYLMFNPQGRAIPDVSAQGYVTIWNGVDGTSASTPTFAAIVALVNDVLAAENKPSMGFLNPWLYFSDVTEGGCGFPALPGWDAASGWGTPFPKF

>PADG_04156T0

VLRPAGWVSIGIQQGWDMFEQHLSDPSHSRYGQHLVDQLVQASPASFDAVTAWLDWISFIWILERLFDAKYAQYRSLSWSLPANLADHIDIVEPTNSFSVVCLSVLYNSIALVNFLGEVNNRSDVDLFLFTTEIVNGDRQEGALDAQTILGLSWPTAYNVGSKNEPLAWLQH--MQSKETLPHVISISYADTEQVPYARRVCNEFAKLGARGVSILVASGDWGVGCIIDSIFAPSFPASCPYVTSVGAEIVSGGGFSDYFERPRYQAVEEYLLYNQNGRGYPDIAAMGFSVLWNGQDGTSASAPTVAAIIALVNDALLENSRPPLGFLNPWIYFTDVTWGGCGFPASRGWDPATGLGTPFPKL

>Pans_587.t1

VMDPRGWLRIALQQRAEALEQAVSTPGHPKYGRHLLRSYTTPSQSATLAVTRWLDWVTFVENASHLLKTDFAWYRTLAYSVPDEVAPHIDLVQPTTRFTPDCLKWLYNTIAFASFLEQYARYDDFQTFQFTVELVGGDDQEANLDVQYIHAVSHPLQYSTGGRNEPLEWLTYLLNQTDDKIPKVISVSYGEEEQIPYAVKVCNMFMQLGGRGVSVIFASGDSGPGCIRSTDFEPTFPAGCPYVTSVGAEKASSGGFSMYHPRPQWQAVEPYLYFDPRGRAIPDISAQGFNVIDKGLSGTSASAPVVAGIVGLLNAARYTLGLPSLGFLNPWLYFTDVVAGGCSWNATVGWDPVTGLGTPFGRL

>Pans_6015.t1

VILPPGWLTIALEPGFAEVKARL--RQFGGNNHHLLHTYLQPKNKNIETVKSWLSLLSFARQVKDLFSADLKYYRALSYSIPSWLRAYIDFVHPITNFSPRCIKQLYINFGVAGFLEQWILHADVAYFLFTVELIGGNPQEASLDVEYAMALGYPIYYVTGGRGNPLPRRNR-----QRALPRI----------VPYALRVCDLFAALAARGVSTFVASGDGGAACVMNDGFIPTFPASCPY---------SAGGFSNYFDRPSWQAVKPYVLFNSTGRAVPDISAIGFQIIMGGVLGTSASAPVVAAMVALINDARMRAGKQSLGWLNPLLYLRDVMVGGCGWPAVQGYDCVTGLGAVFDEL

>pchr_130748

VHEPFGFMRIALQQDPEGLTDALSTPGSARYQKFLVSAFTAPSKEASDSVISFLDWLGFVEKANEMFGADFHVFVTMEYSVPTDLSPHIQLVHPSISYTPACVQDLYNKLGVAGFIQQFANQADLKSFLFTLQTLGGNTQEADLDTQYTIGIATGSFVSCGNNVGGMDWANFINGLASP--PQVVTTSYGENENISAANSLCNLYQQLGAKGVSVLFSSGDGGVG----QSFLATFPSGCPFMTSVGAETSSSGGFSNIFAQPSYQAVSAFLKFNPAGRGFPDVAAVGVEINFQQVAGTSCSSPIFASIISLINDRLAAQGKGPLGFLNPFLYFTDITTGGCGFPAEAGWDPVTGLGTPFAAL

>pchr_133020

VREPGGYLRIALQNNPDGLIDALSTPGSASYGEHLVEKFVAPTAQSSEAVNAWLDWLSVVSKANEIFDADFAVYRTMSYSIPASLEGHLDFVHPTISFTPACIESLYNTLGVSGFIDQFANQADLTTFLFTLQTLGGNPQEANLDIQYTVGIASGTFISVG--LEGLDIINFLLNESNP--PHVLTTSYGDNESISLANNLCNAYAQLGARGTSILFASGDGGVS----QSFVPTFPSGCPFMTSVGAETASSGGFSNYFATPSYQVVSSYIKYNASGRAFPDVAAIGLEIVVDGVDGTSCSSPVFASAIALINDALVAQGKSPLGFLNPFLYFNDITSGGCGFKAAKGWDPVTGLGTPFAAL

>pchr_133398

VHEAAGFLRIALQSNPERLIDALSTPGSPSYGQHLVEALVAPSTETVSAVEEWLDWLSIVSKANELLDADFNVYRTMRYSVPPELEGHLDFVYPTVSFTPACVYTLYNKLAVSGFIEQFANQNDLATFLFALQTLGGNPQEANLDIQYTVGIASGTFISVGEQLGGLDIINALLNENHP--PQVLSTSYGEDEPISLANKLCNAYAQLGARGTSILFSSGDGGVS----SSFVPTFPSGCPFMTSVGAETASSGGFSDIFAAPSYQAVQTYLKFNPAGRAYPDVAAIGLGIVVDGVDGTSCSTPIFASIVSLINDELIGKGKSPLGFLNPFLYFFDVTTGGCGFPASKGWDPVTGLGTPFPAL

>pchr_26825

---PTGWMRISLQDRLEDLITALSDPAHEKYGQHLAEALVAPHEDSVELVDAWLSWLTIVEQASRMMNATYNIYRTMSYSLPSVLHGHVGVVTPTTYFTPACLRALYNKLGVAGYLGEFANDADLQTFFFQHVQVGGNNQEANLDIQYTEGMSFPIYYSTGGSNEPLDWLNFILAQTTV--PQTFTTSYGDDEQVPYATEVCNLFAQLGARGSSIMFSSGDDGVGCLTNDGFQPNFPASCPFVTTVGAEVASGGGFSNYFAQPSYQAVSAFLLFNTSGRAYPDVAAQGFQVIIGGVAGTSASSPTFAGVVALLNDFRLSQGKSALGFLNPIIYFNDITSGGCGFSAGAGWDPVTGLGTPFGKL

>Pchr_g03930.t1

LVEPDGWFRLAIQHNAREFEQKVSTPEHPSYGQHMVKQFLTPHPAISDRIIAWLHWVSFIFQAERMLKTQFFYFRTLGYSVPRALRSHIQLIQPTTLFTPDCLRDLYNRLGISGFLEQYARHDDFNEFLFSVVSIGGDGQEANLDVQYSIPLADETFYSTGGRNEPLEQLHYLLDLPDEELPAVLSNSYGENEQLPYLNATCSLFAQLGARGVSILFGSGDSGPGCVKNDGFLPGYPASCPFVTAVGGEKASGGGFSEVFSRPQYQAVKGYLLYNATGRGYPDISAQAFIVRDQGVGGTSASTPVLAGVISRLNAARIAQGKPRMGFLNPWLYFTDIVLGGCSWDATEGWDPATGLGTPFPAL

>Pchr_g14680.t1

VFEPQGWLQIALQGDTAGFEEAVSTPDHPSYGNHFMKRMLQPSAESADSIRDWLDWMTFVETANELLAANFQFYRTLQYSVPEALMPHINMIQPTTRFTPQCLKNMYNKVGFASYLEEYARYSDLELFEFSVIQYGGNDQEANLDLQYIVGVSSPTEFSVGGRNEPLEFLQNVLKMEQQDLPQVISTSYGENEQVPYARTVCNLFSQLGSRGVSVIFASGDSGVGCQTNDGFPAQFPAACPWVTSVGAEKASSGGFSDLWDRPKWQAVSDYLLFNPKGRAFPDVSAQGYAIYDKGVDGTSCSAPAFAGVIALLNDARLKANKPPMGFLNPWLYLNDIVHGGCSWNATKGWDPVSGLGSPFATM

>PGTT_15038

LFEPRSFLKIALSGGMDLIKKRLSDPESSSYGRHLIRDLSAPSSSSLEAIVKWLDWVSLLQKAEEMLDTTYFYYRTERYSLPEEIHSHVELIQ----FTNACLRELYNRIGVTAYIGERANYEDLKDFLFTVVSVGGNPQEGNLDIQTTMGFTAPIFYTTAGSNEPLDWLLYIASQPDSAVPQVISTSYGDDEQVPYAKRVCDQLAALTARGVSLIFSSGDDGVGCLSNDGFMPIFPATCPYVTSVGAEEASGGGFSEYFDRPQYQQVQTYLLYNPKGRGVPDVSAQGYLMTWQKVGGSSAAAPTFASVIALLNDNRIARGMPALGFLNPWLYLNDIVIGGCGFAAAKGWDPVTGLGTPFPAM

>PGTT_17496

VHETL-WLKFGLQSNLDTLHDELSHPDSSSYGHHWVKEHFSPSAQAIDEVTAWLTWINILSEAENLLDTKYHLYACEDYKLPERLFRHIDMVMPTVHFTIECLKALYHTLAIVEYTPQSVIYKDMDLFFPNLIPIGGELNESNLDLQYSMPLVHPSLYQVGD-FNNLDALDSKRDCGIVNPANVISTSYGMNEAATYLIRQCNEYGKLGLMGTTFLFSSGDNGVACLSRDGFNPSFPGTCPYVTSVGAEVASGGGFSNVFKMPNYQAVQNFFRYNSSMRGFPDLSANGYVVAVEGVYGTSASAPVVASMLTMINDARISVGKKPVGFINPAIYFNDIKDGGCGFSAVTGWDPVTGLGTMFTKF

>Pmar_007090.t1

LHEPARWMRIGLQSNLEEMYQHLSHPDSANYGKHWVIKAFQPSDKTVESVKTWLGWLAFPDEAERLLKTEFHEFACDEYHVPHHLKEHVDFVTPGIKLSPACVAALYNSLGIFESELQFYTQQDLDLFFPIAANVGGQSTEVSLDLQLAYPIVYPTDYEVDDFNDKLDQTYPELMCGVYKPTNVISVSYGGQEVLPYQRRQCLEYAKLGLQGVSFLFASGDSGVSCI---GFNPTWPNTCPYVTNVGAESASGGGFSNVFPIPDYQAVATFFIYNRIGRGVPDVAANGIMVYNGGSGGTSASTPIFAAVINRINDERLWAGKSPLGFLNPSLYLNDITNGGCGFSAVPGWDPVTGLGTPFPKM

>Pmar_010230.t1

VHEPASWVRIGLQSNSDVGHDLLSNPNSPNYRKYMVNDFFAPSEKAVSAVRTWLQWLQFADEVERLLGTEYYIYGCEEYHVPKHLSEHIDYITPGVKTTPDCIRTMYNELGIFEDLGDYYAQEDLDLFFPTLKGIGAAPAESDLDFQIAYPIIWPVLFQTDDADPPPDPAANSLQCGVYKPTNVVSISYGGDEILSYQKRQCDEFKKLGLQGVSVVVASGDSGVACQGKSGFNPDFPAGCPYITTVGAEVAASGGFSNIYPRPAYQAVNTYLLYNAAGRGYPDVAAVGVLIYNAGIGGTSASAPVFAAILTRINEELLAKKGTTVGFVNPTLYFHDITSGGCGFKTAAGWDPVTGLGTPYPAL

>Pmar_012620.t1

VVEPDGWFRLAMQPRAGEFEQHVATPGHKMYGKHMVKAFMQPSAEVSDAVVSWLDWAKFLHQAEKMLNTTFYIFRTLQYSVPREIHQHVRLIQPTTHFTPDCLRDLYNKLGISGYLEQYARYDDLKAFLFTVQEIGGNDQEASLDMQYGISLSYQIFFSTGGRNEPLEQLHYLLNLSDEDLPAVLSTSYGENEQLPYTNTTCSLFAQLGARGVSIIFSSGDEGVGCLTNDGFNPIYPASCPFVTSVGGERASSGGFSERFPRPSYQAVASYLLYNPGGRGFPDVSAQAYLVRDHGVDGTSASAPTFAAIIADLNSVRLDNNQSILGFLNPWLYFTDIINGGCSWNATPGWDPVTGLGTPFDSL

>Pnod09464.t1

PFEPEGWLQIALQGDVAGFEQHVSTPSHPSYGAHYMKRMIQPSSETVASVSAWLDWVTFVGVANKMLDTKFAWYRTLEYSVPDDVAEHINLIQPTTRFTPQCLKTLYSKVAFASYLEQYARYNDLALFEFSVVQFGGNDQEANLDLQYIVGVSAPTEFSTGGRNEPLEFLQGVLKLPQSELPQVISTSYGENEQVPYALSVCNLFAQLGSRGVSVIFSSGDSGPGCQSNDGFQPQYPAACPFVTSVGSETASSGGFSDYWKRPSYQAVKAYFYFNRHGRGFPDVATQGFRVYDQGLQGTSASAPAFAGVIGLLNDARLKAKKPTLGFLNPLLYLNDIVLGGCGWNATAGWDPVTGLGTPFPKL

>Pnod10163.t1

VHEPMAWVRIGLQRNLEHSDDFLADPDSPNFGKFWVANTFAPHPETSEAVLAWLNWIEFVGEVEELLQTEYHYYACDEYGLPRHVREHVDFIMPTIQLTIDCLRAIYNQLGVAEW-ADYLYLPDLKIYFPEFISIGGNLTESALDIQTAYSIIWPRLYQNGDSNAPLDPAYPPLQCGGAPLSNVISVSYGQIEGLPYQERQCREWMKLGLQGVSVVFASGDSGVACLNSESFSPSFPANCPYVTSVGADGASGGGFSNIFPRPSWQAVSNYLVFNSSGRGIPDVSAIGVATVYLNFGGTSASAPIFASIITLLNEERLEQGKGPIGFLNPTIYFNDVTVGGCGFPASPGWDPVTGHGTPYEKM

>Pnod12886.t1

AVKPRKWLQIGVQGDFDELERHLSDPYHVRYGQHLVNALTQPKDEALDAVHEWLDWINIIEDAEKLLATKYSVYRATSWSLPSHLHKHIDTIQPTTSFTPTCFKTLYNQIGFNNFLKEVPIRPDAKLFLYKFVSIGGQQDEANLDVQTILGMTYPTAFTTGGENEPLEWVTYVLKQKSL--PQVISTSYGDDEQVPYAKRVCNSFAQLGARGVSLLFSSGDGGAGCVSNDGFLPSFPAGCPYVTTVGAEVAASGGFSEYFSRPSYQAVKGYLLYNAAGRGYPDISAQGFEFVWNSISGTSASSPLAASVIALVNDALISAGKPTLGFLNPWLYFTDVLSGGCGFAVTKGWDAVTGFGTPFPEL

>Pnod12941.t1

VVEPVGWLKVHLNKDMDKFHDLAATPGHAEYGNHLILAMIAPKQESTDLVMQWLDYVTIVKTIEKLLDAEYSVFRTLKYSLPASLKSHVDMVQPTTFFTPTCLANLYGLLGIAGFLEEYAIKADFTTFLFTCAAVGGCPSEANLDVQYAGSISTSTYYSTAGRNEPLEFLNYLLALPAAQLPNTLSISYGDDEAVPYATNACNLFSQLGARGVSILVSSGDSGVGCSVNGKFTTAFPAACPWVTTVGGEAAGGGGFSEIFGRPSYQAVSKWLYFNASGRAYPDISAQAFVIIAGGVSGTSCSAPATAGIIQLLNSGRIAAGKKGLGFLNPWLYFTDIKTGGCGFSAVSGWDPATGWGTPYGSL

>Pnod14758.t1

TVEPEGWFRVAVSADSSLLERTLSSPSSPNYGQHLLKDLIKPRAESSDAVLSWLEWISFVKRAEEMMGAKFSTYRSLSYSVPKEIRRHIDMIHPITRFTPSCLADLYVSIGVNGFLEQYARYSDFVKFTFTYTLVGGNDQEANLDIQYTAGLVGPSFFSTAGRNEPMEFFTYLLSLENEKLPSVLSTSYGESEQVGYANKVCDMIGQLGTRGVSVIFSSGDTGPGCQTNDGFQAIFPASCPYVTSVGGERASSGGFSDFWKRPAYQAVTNYLLYNAAGRGFPDVAAQGFRVVDKGVGGTSASAPVFASVVALLNNARKAAGMSQMGFLNPWIYLNDIVNGGCSWNATVEWDPVTGHGTPFEKL

>ppla_112809

LHEPSGFLRIALQNNVDGLVDALSDPTSESYGQHLAASFVAPASESTSAVQAWLDWLGVSSTANDLLSANFSVFRTLAYSIPAELTAHIDFVHPTTTFTPWCLQYIYNGIAVTAYGGAIPQESDLQRFLFTFQSVGGSTSEANLDVQYAIGLATGTFLSVGGEDDALDTANYLLSLESP--PPVVATSYGDNENISLAYNLCNAYAQLGARGVSVIFASGDGGVS----KNFVPTFPSGCPYVTSVGAEQASSGGFSNYWPQPSYQAVSAFLLYNASGRAFPDVAAYGYDIYNSRVSGTSAAVPTVASIVALLNDRLLTAGRATLGWLNPWLYFASVTAGACGFYATSGWDPITGLGTPFDKL

>ppla_113327

VHEPSGFLRLGLNSNTDGLIATLSTPWSANYGKHLVEAFTAPTKGTVDAVNTWVDWLSIVSKANEMLDANFSVYRTLSYSIPTDLKGHLDLVHPTTTFDPQCLEELYDYLVVTGYNNEYPSTTDLVDFLYTVVEIGGYDPEADIDTQYTVGLAIGTFTSVGESVFGLDTANYWLGQSTT--PSVISTSYGSNEEISVYNSLCNAYASLGLRGTSVLFSSGDGAVT----QSFVPTFPSGCPYVTSVGAETASSGGFSNVFAIPPFQSVAGYLLYNASGRGYPDVSAQGFIFNYQGVEGTSCSSPTFASVIALVNDRLVAAGRSKLGWLNPFLYLTDITSGGCGFSATTGWDPVTGLGTPFDEL

>ppla_117588

KVKPRGWLRIALQSNFAELEQHLSDPFHARYGAYLVEALVAPHDDSVQLVEEWLDWITVVSLAETMLKTEYHIWRTTSYSLPERLVDHVDLVQPTTMFTPTCLKQLYNKVAATGYLDEYANYEDLQMFYFSVVSVGGNNQEADLDVEYAFSLSYPTFYTTGGSNEPSEWLSYMLSIEDP--PQTISTSYGDDEQVPYAKRVCEDFAQLGARGVSIIFASGDGGVGCYSNDGFIPGFPASCPYVTAVGAEIASGGGFSNYFPRPSYQAVEAYLLYNPNGRAIPDVAAQGFQIFWDGVAGTSASTPSFSGIVSLLNDARIANSLSPLGFLNPLVYFNDITVGGC-FNASVGWDPVTGWGSPFGVL

>ppla_120448

--------------------------------------------------------------------------------------------------SSDT---------------------------FTVETLGGDPQEANLDTQYTVGLATDVFISVG--LDGLDIINYLLAQDAP--PQVLTTSYGSSESVPMAENLCNAYAQLGARGVSILFASGDGGVS---QDSFVPTFPSGCPYLTSVGAETASSGGFSNYWGVPSYQAVSGYLRYNASGRGYPDVSAQGFNIVLDQVSGTSCASPTFASVIALLNDELIAAGKSPLGFLNPWLYLNDVTSGGCGFSATTGW-------------

>ppla_121576

VLEPRGFLRLALQNNMDGLIEALSTPSSAKYGQHLVEEYVSPTSMTIAAVNAWLDWLAIVSQANSMLDADFSVFRTLSYSIPSDLVSHLDLIHPTVTFDADCIETLYNYIAVTGYNNYYANEADLDYFLFTVETLGGNDQEANLDTQYTTGLATYTFLSVGQNVFGLDVANYFLDMETP--PSVITTSYGSNEELSVAYALCNAYAQLGARGVSVLYSSGDGGVS----QNFVPSFPSGCPYVTSVGAEVASSGGFSNYWATPSFQAVSNYLLYNASGRGFPDVSAQGIMIAQYQVDGTSCSSPIFASVVGMLNNQLISAGKSTLGWLNPFLYLTDITSGGCSFSATTGWDPVTGLGTPYTLL

>ppla_128724

VLGPAGFLRIALQSNPAGLEDALSTPSSPNYGQFLAASFVVPSPETQATVNAWLDWLSFVSKANEIFEAEFNVYRTMSYSIPQELQGHLTVVYPTTTFTPSCLQSLYNKIGVAGFDEQYADDQDLKTFLFTVQTLGGNSQEASLDIQYTVGVATNIFISAGEQLEGLDMANFLLNEDSL--PQVFTTSYGPNENIPLSINVCNAYAQLGARGVSVLFASGDGGVS---QYDFIVPFPCGCPYHTSVGSETASSGGFSNYFARPSYQAVSAYLLYNASGRGFPDISTQGFDVVIDTVSGTSASSPTFASVIALINDELIAAGKSPLGFLNPWLYLNDITEGGCGFSATTGWDPVTGWGTPYAKL

>ppla_39235

-------LRVALQNNIAGLIDALSSPSSPNYGKWLVEAHVAPKQHSVAAVNSWLDWLRIVAKANDMLAANFSVFRALSYSVPSDLAEHIDLIHPTTAYSSACLQHLYNGIAVTEFEKQYAQAADLHSFLYTVISIGGNPQEADLDLQYTAGLATGTVTIAGGDLAGLDTGLSLLGLESP--PQVVSTSWDGDEDFPHAVYLCNVYAQLGARGVSMIFASGDEGAS--RSEGFSPTFPATCPHVTTVGAEVVSGGGFSNYFLRPDYQAVSAYLLYNASGRAYPDVAAYGCTYIMGGGTGTSCSAPIFASVIALLNDRLLAAGKPTSGFLNPW--------------------------------

>ppla_48962

VLGPAGFLRIALQSNPTGLEDALSTPSSSNYGQFLAASFVTPSSETQAAVNAWLDWLSFVSKANGIFEAEFNVYRTMSYSIPQELQGHLTVVYPTTTFTPACLQSLYNKLGVTGYGDNWANKADLKTFLFSLETLGGDPQEADLDTQYTVGIATGTFISVG--LDGLDTINYLLNQDAP--PQVLTTSYGDYEPIPMAENLCNAYAQLGARGVSLLFASGDGGVS----QSFVPEFPSGCPYMTSVGGETASGGGFSNYWSRPSYQVVSDYLLYNASGRGFPDVATQAFIIAYEGVSGTSCASPTFASIIALLNDELIAAGDSPLGFLNPWLYFTDITSGGCGFNATVGWDPVTGLGTPYAKL

>ppla_48986

VLAPAGYLRIALQSNPAGLEEALSTPTSPNYGQFLAASYVAPSPETQTVINAWLDWLGFVGKANELFDAAYSVYRTLSYSIPQELEGHLSVVYPTTTFTLSCLQALYSRIAVAGFDDQWANEADLMTFLFTLQDLGGNNQEANLDIQYTVGIATDVFVSAG--LDGLDMADLLLNEDSP--PQAFTTSYGPNEDVPLSYNLCNAYAQLGARGVSVLFASGDGGVS----QSFVVPFPDGCPFMTNVGSEIGSSGGFSNYYARPSYQAVTAYLLYNASGRAFPDVATQGFTVVIDQVSGTSCSSPTFASVVALLNNELISAGKSPLGFLNPWLYFNDITEGGCGFYAIAGWDPVTGWGTPYAKL

>ppla_50115

VHEPSGYLRVGLENNVDGLISALSDPSSANYGQHLANSYLTPTAESASAVNAWLDWLAVVSKANELLGADYSVFRTLSYSIPANLTGHLDLVHPTISFTPDCLLYLYNGYAVTEYIEQWATYADLKTYLWIYQSIGGNPQEAELDVQYAIGLTTDTFISVG-----LDTALTLLNETAP--PQVMSTSYGDDEDVSFAYKLCNAYAALGARGVSVVYASGDGGVS---HFDFLPVFPAACPYVTSVGAQTASGGGFSNYWTRPLYQAVAGYLLYNPSGRGYPDVAAYGFDVVYAGVSGTSCSSPTFGSVIALLNARLLAAGRPTLGFLNPFLYLTDITTGACGFYASEGWDPITGLGTPFANL

>ppla_50482

LHEPSGFLRIALQNNVDGLVDALSDPTSESYGQHLVASFVAPASESTSAVQAWLDWLGVSSTANDLLSANFSVFRTLAYSIPAELTAHIDFVHPTTTFTPWCLQYIYNGIAVTAYGGAIPQESDLQRFLFTFQSVGGSTSEANLDVQYAIGLATGTFLSVGGEDDALDTANYLLSLESP--PPVVATSYGDNENISLAYNLCNAYAQLGARGVSVIFASGDGGVS----KNFVPTFPSGCPYVTSVGAEQASSGGFSNYWPQPSYQAVSAFLLYNASGRAFPDVAAYGYDIYNSRVSGTSAAVPTVASIVALLNDRLLTAGRATLGWLNPWLYFASVTAGACGFYATSGWDPITGLGTPFDKL

>ppla_50496

VHEPSGYFKLALQNDPNGLVDALSAPDGPKYGQYLVVSFVSPRPESTAAVNAWLDWLSIVNKINDLIDAEYSVFRTLSYSIPADLIDHLDLIHPTISFDPACIQYLYNRLAVTGYDGEWASKADLQQFLFTLQTLGGNPQEAAIDVQWTVGLATDTFISVGGD---LDTANFLLGEDSP--PQVVSTSYGDDESVSLAYALCNAYAQLGARGVTVINSSGDGGVS----HYFVPTFPSGCPFVTSVGGEWGSGGGFSNYWSRPAYQAVSHYLLYNASGRGYPDVSAYSFDVIVDGEVGTSCSAPTWASVVALLNDRLVSAGKPALGFLNPFLYLTDIVNGWCGFEATVGWDPVTGLGTPFTNL

>ppla_53113

VLAPAGFLRVALQSNPAGLEDALSTPSSANYGNHLAAAFVAPTQEATAAVTSWLDWLSLVSQANELFGAQFNVYRTMSYAVPQTLAAHLTVVYPTTTYTPACLQSLYNQLGVSGFIDQFANQADLKTFLFSVQTLGGNSQEANLDTQYTVGLATGTFISVGEKDLGLDIMNFLLNENDP--PAVLTTSYGDNEDIPMADNLCNAVAQLGARGVSVLFASGDGGVS----QAFVPTFPSGCPYLTSVGAETASAGGFSNYFGTPSYQAVSTYLLFNASGRGYPDVSTQGFEIVVDGVDGTSCASPTFASVIALLNDQLVAAGKSTLGFLNPWLYLTDITSGGCGFPAVTGWDAVTGLGTPFAKL

>ppla_53544

LHEPVGFIQLALQNNVSGLVDELSDPSSANYGQYLVVSFVSPRPESTAAVNAWLDWLSIVNKINDLIDAEYSVFRTLSYSIPADLIDHLDLIHPTISFDPACIQYLYNRLAVTGYDGEWASKADLQQFLFTLQTLGGNPQEAAIDVQWTVGLATHTFISVGGD---LDTANFLLGEDSP--PQVVSTSYGDDENVSLAYALCNAYAQLGARGVTVINSSGDGGVS----HYFVPTFPSGCPFVTSVGGEWGSGGGFSNYWSRPAYQAVSHYLLYNASGRGYPDVSAYSFDVIVDGEVGTSCSAPTWASVVALLNDRLVSAGKPALGFLNPFLYLTDIVNGWCGFEATVGWDPVTGLGTPFTNL

>ppla_53719

VHEPSGFLRLGLNSNTDGLITTLSTPSSANYGQHLVGAFTAPTKETVDAVNAWIDWLAIVSKANEMLDADFSVFRTLSYSIPTDLKGHLDLVHPTTTFDPQCLEQLYNYLVVTGYNNEYPSTTDLVDFLYTVVEIGGYDPEADIDIQYTVGLAIGTFTSVGESVFGLDTANYWLGQSTT--PSVISTSYGSNEEISVYNSLCNAYASLGLRGTSVLFSSGDGAVT----QSFVPTFPSGCPYVTSVGAETASSGGFSNVFAIPPFQSVAGYLLYNASGRGYPDVSAQGFIFNYQGVEGTSCSSPTFASVIALVNDRLVAAGRSKLGWLNPFLYLTDITSGGCGFSATTGWDPVTGLGTP----

>ppla_56190

VLGPSGFLRIALQSDPAALEEALSTPSSSNYKQYLVSAFVAPSPEAVSAVNAWLDWVEVVSKANEIFNADYSVFRTLSYSIPEELTDHVAIVHPTTTFTPACLQSLYNTLGVSGFSDQYANQADLATFLFTVETLGGDPQEANLDTQYTVGLATDVFISVG--LDGLDIINYLLAQDAP--PQVLTTSYGSSESVPMAENLCNAYAQLGARGVSILFASGDGGVS---QDSFVPTFPSGCPYLTSVGAETASSGGFSNYWGVPSYQAVSGYLRYNASGRGYPDVSAQGFNIVLDQVSGTSCASPTFASVIALLNDELIAAGKSPLGFLNPWLYLNDVTSGGCGFSATTGWDPVTGLGTPYTSL

>ppla_58105

-------ISFAS-----------TGPKLPITVKQL---------------------------------------------------------------DPACLEALYNYIVVTGYDDQYPSTSDLENFLYTVVELGGYDPEADLDIQYTVGLALGTFFSVGEDVFGLDTANYWLGQSTA--PSVITTSYGSDESISVFNSLCNAYASLGARGTSVLFASGDGGVS---QSGFVPTFPSGCPYVTSVGAETASSGGFSNVFGTPSFQDVSSYLLYNASGRGFPDVSAQGFIIGYEGVSGTSCASPTFASVIGLVNDRLVAAGKSPLGWLNPFLYLTDITSGGCGFSATTGWDPVTGLGTPFSAL

>ppla_88376

VHEPTGYLRVALQNNIAGLIDALSSPSSPNYGKWLVEAYVAPKQDSVAAVNSWLDWLGIVSKANNMLAANFSVFRTLSYSVPSDLVDHIDLIHPTIIFTPACLQHLYNGIAVTEYEEQYAQGADLHSFLYTVISIGGNPQEADLDLQYTAGLATGTVTIDG--LTGLNTGFSLLGLESP--PQVVSTSWGGDENFSYATNLCNVYAQLGARGVSMIFSSGDGGVS---QFEFNPTFPSTCPHITTVGAEVASGGGFSNYFPRPDYQVVSAYLLYNASGRAYPDVSAYGCSVVIGGVSGTSCSAPIFASTIAILNDRLLAAGKPTLGFLNPWLYFTDIVSGSCGFFATKGWDPVTGFGTPFASL

>ppla_89125

VHEPSGYLRVALQNNIDGLIEALSTPSSSNYGKWLVEAYTAPESETVDAVNSWLDWLGIVSKANEILATDFSVFRTLSYSLPSNLIGHVALVHPTITFTPACLQELYNGLAVAEYEYEYAEESDLHTFLFTVLSINGNPQEASLDLQYTVGIATNTFITVGYN---LDTANTLIALDSP--PQVVSTSYGEDEQVSFATTLCNAYAQLGARGVSLIFSSGDGGVS----HFFNPTFPSVCPHITTVGAEVASGGGFSNIFPRPSYQAVSAYLLYNASGRGYPDVSAQGFEVVNAGVSGTSCSAPTFASVVALLNDRLLASGKPTLGFLNPFLYFNDITSGACGFFTAPGWDPVSGLGTPFAKL

>ppla_89318

VLAPARFLRVALQSNPAGLEDALSTPSSANYGNHLAAAFVAPTKEATAAVTSWLDWLSLVSQANELFGAQFNVYRTMSYAVPQTLAAHLTVVYPTTTKTPACLQSLYNQLGVSGFIDQFANQADLKTFLFSLQTLGGNSQEANLDTQYTVGLATGTFISVGEKDLGLDIMNFLLNENDP--PAVLTTSYGDNEDIPMADNLCNAVAQLGARGVSVLFASGDGGVS----QAFVPTFPSGCPYLTSVGAETASAGGFSNYFGTPSYQAVSTYLLFNASGRGYPDVSTQGFEIVVDGVDGTSCASPTFASVIALLNDQLVAAGKSTLGFLNPWLYLTDITSGGCGFPAVTGWDAVTGLGTPFAKL

>ppla_89706

MHGPTGYLRVALQNNIAGLIDALSSPSSPNYGKWLVEAYVAPKQDSVAAVSSWLDWLEIVAKANDMLAANFSVFRTLSYSVPSDLAEHIELIHLTIAFTPVCLQHLYNGIAVTEFEKQYAQAADLHSFLYTVISIGGNPQEADLDLQYTAGLATGTVTIAG--LAGLDTGLSLLGLESP--PQVVSTSWSGDEDIPYAVHLCNVYAQLGARGVSMIFASGDGGAS---RTEFSPTFPATCPHVTTVGAEVASGGGFSNYFPRPDYQAVSAYLLYNMSGRAYPDVTAYGCTFIMGGGSGTSCSAPIFASVIALLNDRLLAAGEPTLGFLNPWLYFTDIVSGSCGFVATEGWDPVTGFGTPFANL

>ppla_98402

VHEPSGFLRLALNSDTDGLIAALSTPSSANYGQHLVDAFNAPTKESVDAVNAWLDWLAIVSKANVMFDADFSIFRTLSYSIPTDLVDHLSLVHPTTTFDPACLEALYNYIVVTGYDDQYPSTSDLENFLYTVVELGGYDPEADLDIQYTVGLALGTFFSVGEDVFGLDTANYWLGQSTA--PSVITTSYGSDESISVFNSLCNAYASLGARGTSVLFASGDGGVS---QSGFVPTFPSGCPYVTSVGAETASSGGFSNVFGTPSFQDVSSYLLYNASGRGFPDVSAQGFIIGYEGVSGTSCASPTFASVIGLVNDRLVAAGKSPLGWLNPFLYLTDITSGGCGFTATTGWDPVTGLGTPFSAL

>ppla_99394

-------------------------------------------------------------------------------------------------------------------------------FLYTVVELGGYDPEADLDIQYTVGLALGTFFSVGEDVFGLDTANYWLGQSTA--PSVITTSYGSDESISVFNSLCNAYASLGARGTSVLFASGDGGVS---QSGFVPTFPSGCPYVTSVGAETASSGGFSNVFGTPSFQDVSSYLLYNASGRGFPDVSAQGFIIGYEGVSGTSCASPTFASVIGLVNDRLVAAGKSPLGWLNPFLYLTDITSGGCGFSATTGWDPVTGLGTPFSAL

>Ptri_00288.t1

VVEPEGWFRIAVSKNRDLFERTLSSPSSPNYGKHLLKDLIKPRAESVATVINWLEWINFVKRAEAMMGTTFKTYRSLGYSVPGHVRPSIDMIQPTTRFTPTCLADLYTKLGVTGYLEQYARFGDLEKFLFDVEAI--------------IGLISPTFYTSPGRNEPLDLFTYLMELDDGELPQVLSTSYGENEQVPYAKKVCDMIGQLGARGVSVIFSSGDSGTGCQTNDGFSPIFPAACPYVTSVGGERASSGGFSDLWPRPAYQAVGDYLLYNPNGRGFPDVAAQGFQVVDSGVAGTSASAPVFAAVVAMLNNARMGAGMPTLGFLNPWIYMNDVVDGGCSWNATKGWDPVTGYGTPFEQL

>Ptri_01300.t1

IVEPSGWLKIHLNKDMDKFHEHAATPGHNLYGQHMILAMVAPAEESAALVMKWLDYVTVVKEIEQLLDAEYNVFRTLSYSLPKFLKSHVDMVQPTTFFTPTCLATLYGLLGIAGFLEEYAIKSDYTSFLFTCTTIGGCPSEANLDVQYAGSISTSTYYSIAGRNEPIEFLQYVLALPDPSLPNTLSISYGDEEAVPYATNACNLFSQLGARGVSILVSAGDSGVGCTVGGKFTTAFPAACPWVTTVGGEVAGGGGFSEIFGRPSYQTVSKWFYFNASGRAYPDISAQAFVIVVGGVSGTSCSAPTTAGIIQLLNSGRIAAGKKGLGFLNPWLYFTDIKNGGP---PHNDWYPVSQ--------

>Ptri_03887.t1

PFEPEGWLQIALQGDTEAFEQHVSTPSNAKYGQHYMKRMLMPSEQTVTSVSSWLDWVTFVGVANELLGTKFSWFRTLEYTVPDDVAQHINLVQPTTRFTPQCLKKLYSKVAFASYLEQYARYNDLELFEFTVVQFGGNDQEANLDMQYMFGLAQPTEYSTGGRNEPLEFLQGVLKLPQEELPQVISTSYGENEQVPYALTVCNMFAQLGSRGVTVLFSSGDSGTGCLSNDGFQPQYPATCPFVTSVGSETASSGGFSDYWKRPAYQAIKAYFYFNRHGRGFPDVAAQGYAVYDKGYQGTSCSSPAFGGIVALLNDARLKSKKPSLGFLNPLLYLNDVVLGGCSWNATVGWDPVTGLGTPFPKL

>Ptri_07068.t1

AVKPREWLQIGLQGRFEELDRHLSDPDHVRYGQHLVDELVAPTSETYNLVHEWLDWVIVIEMVESLLDTEYHTYRTTKWSLPRHLHSHIDTVQPTTSFTPECFQTLYNSVAFNNFLGEIPIRPDTKKFLFKTISINGLQDEANLDVQAIAGISWKTSYSTGGSNEPLVWVNWLLTQRSI--PNIISTSYGDSEQVPYAERVCRQFAQVGARGTTLFFSSGDSGIGCYTNDGFNPNFPASCPYVTTVGAEEAASGGFSNYFSRPSYQVVPKYILYNKTGRGYPDLAAQGFAYFWNGISGTSASSPLTAGIFALVNDALISKGKPTLGFLNPWLYLTDITKGGCGFPVTKGWDPVTGFGTPFPEL

>SS1G_04958

LLEPQGWLRIALQPDHELFEQKLSSPDHVEYGQHLLKRFIKPADETTHAVLSWLEWINFISQAEEMLNTTFHYYRTLKYSVPQEISSHITMIQPTTRFTPQCLRDLYSRLGVAGYLSQWAKYDGLEAFLFSYALIGGDTQEANLDMH------------TGGRNEPLDYLNYMLGLPNNQLPQTITTSYGEDEQVPYSKTVCKMFGQLGLRGVSILFSSGDTGVGCQTNDGFLPIFPAACPYVTSVGGEAASSGGFSDRWPRPSYQAIKGYLLYNRHGRGFPDVAAQGFHVVDVNLSGTSASSPAFAAVISLLNNARLNAGRKPLGFLNPWLYLNDIVNGGCSWNATKGWDPVTGLGTPFAKL

>SS1G_09268

VKEPKQWLNIALQSQFDELERQLSDPSHQKYGNHLVNELIRPSSQTLDSTHEWLDWIHLVEVAERLLNTEYHVYRTPNWSLPMHLHKHIDAIQPTTSFSPQCFQALYNKIGFNNFLGEVPIRPDDALFLFKYVSIGGNTTEANLDLTAISGISYPTAYSTAGENEPADWLNYILAQPDSDIPQVISTSYGDDEQVPYAKRVCEQLAQLGARGVSILFSSGDSGVGCFSNDGFIPEFPTSCPYVTSVGAEVATGGGFSNYFARPSYQTVSAYVLYNKSGRAYPDISAQGFAYVWNQISGTSASTPLMSGIISLVNDALITKGKPVLGFLNPWLYFTDILGGGCGFPVTKGWDPATGFGTPFPEL

>SS1G_12210

VHEPRNWMRFAIQNNLHNAEAYLSNPSSANYGKHWVAETFAPSAESVKAVFQWLNWIHAVAEAESLLNTKYYAYACDDYSVPENLRKHIDFITPTLHFTPDCLRALY------------SAKGNLDLFFPIFDSIGGLQTESDLDLEYGMALAYPTLYQAGGEDDPSDYYKGPPNCGGFAATKVISTSYGFDESLTYERRQCNEYLKLGLQGVTILYSSGDYGVAC-----FSPSFPGTCPYITSVGAEEASGGGFSNVFSMPKYQAVATYFQYNNSQRGYPDISANGYVVAVDGVYGTSASSPVIGAIFTLINAARINAGKAAIGFVNPVLYLNDITSGGCGFTAVKGWDPVTGLGTPYPNL

>SS1G_13922

AVKPSQWLQIGLQGQFDELERHLSDPSHHRYGQHLVNDLIKPTDETYNLVHEWLDWIKIIEDIESLLDTEYSTYRTPEWSLPLHLHDHIETIQPTNSFTPLCLRTLYNKMALTDYLGESNNRSDTKLFLFEVQIIGGDEQEGNLDSETMLGIGYPIAYTTGGSNEPLTWLQYMLDQKDKDLPGVVSNSYQDTEQVPYAVSVCKGFAQLGARGVSVLFGSGDNGVGCISNVDFLAMFPSTCPYVTSVGGEVVSGGGFSRYFPRPSWQAVKPYLYFNATGRAFPDIAAQGYITVWNGLDGTSAATPTASAILALINDALIAAGKPTLGWLNPWLYFTDVTSGGCGFPATKGWDAASGFGTPFPKL

>TEQG_02578T0

VIEPEGWFWLAIRENPEKLYDTISTPGRAQYGKHLLDDLLRPKAETSESIVNWLDWVRFVKTAENLMNTRFNVFRTLEYSVPVAISAHVQMIQPTTLFTTACLRELYNRIGVSGFLEEYAQYRDLDLFLFSEGLIGGNTQEANLDMQYVVGLSHKTYYSTAGRNEPLEQLRYLVKLPKNQLPSVLSTSYGETEQLPYTKATCDLFAQLGTMGVSVIFSSGDTGPGCQTNDGFNPIYPASCPFVTSIGGERASSGGFSDRFPRPQYQAVKGYLLFNPNGRAFPDIAAQGYAVYDKGVSGTSASAPTIAAIIAQLNDFRLAKGSPVLGFLNPWIYFTDIVNGGCSWNATKGWDPVTGFGTPFQAL

>TEQG_07021T0

VVEPEGWMNLAVQQNTRTFEQIVSTPGHRNYGKHLLKGLLRPRRETSNLILSWLDWIHFISKAERMLQTQFYYFRTLKYSIPSHLARHVYMIQPTTKF-------LY----------------------FSVVHISGNLQEASLDIDYALGLSN-VFYTTSGRNEPLDQLHYLLSLPQEALPAVLSTSYGENEQVPFSHATCNLFAQLGARGVSVIFSSGDSGVGCLTNGKFNPTFPASCPFVTSVGAERASSGGFSDRHSRPRYQAVQHYLLYNPKGRGIPDVSAQGFAIYDHGVSGTSASAPAFAAIIANLNAIRLRANKPVLGYLNPFIYFTDIVHGGSSWNATEGWDPVTGVGTPFRRL

>TEQG_07074T0

VVEPQGWFKLALQGKTAEFEQRVSNPKHADYGKFMLDAFLQPSPQVKDSVFNWLDWLTFIAKAEKLFNTRFYTFRTLKYSVAASAAPYVQMVQPTTKFTPDCIRDLYNRLGISGYLEQYARLDDFSTFIFDFKSIGANEQEASLDVDYAIGLS-GTYYGTAGRNEPIEQLFYLLDLPDSELPAVLSTSYGENEQVPYSSVVCSLFGRLGARGVSVIFSSGDTGVGCQSNDGFNPIFPAACPFVTSVGGEVASSGGFSERFARPWYQDVRHYLMYNPQGRGFPDVSAQSFATRDHGVSGTSASAPLFAGVVSILNSIRLANNKPRMGFLNPWLYFTDIVHGGCSWNATKGWDPVTGLGTPFEKL

>TERG_00619T0

VIEPEGWFWLAIRENPEKLYDTISTPGRAQYGNHLLDDLLRPRAETSESIISWLDWVRFVKTAEKLMNTRFNVFRTLEYSVPVAISAHVQMIQPTTLFTTACLRELYNRIGVSGFLEEYAQYRDLDLFLFSEGLIGGNTQEANLDMQYVVGLSHKTYYSTAGRNEPLEQLRYLVKLPKNQLPSVLTTSYGDTEQLPYTKATCDLFAQLGTMGVSVIFSSGDTGPGCQTNDGFNPIYPASCPFVTSIGGERASSGGFSDRFPRPQYQAVKDYLLFDPNGRAFPDIAAQGYAVYDKGVSGTSASAPAMAAIIAQLNDFRLAKGSPVLGFLNPWIYFTDIVDGGCSWNATKGWDPVTGFGTPFQAL

>TERG_06023T0

VVEPEGWMNLAVQQNTRTFEQIVSTPGHRNYGKHLLKGLLRPRRETSALILSWLDWIHFISQAERMLQTRFYHFRTLKYSVPSRLARHVYMIQPTTKFTPRCLRELYNVIGVSGYLDQYARYSDFHKFLFSVVHISGNLQEASLDIDYALSLSN-VFYTTSGRNEPLDQLHYLLSLPQEALPAVLSTSYGESEQVPFSHATCNLFAQLGARGVSVIFSSGDSGVGCLTNGKFNPTFPASCPFVTSVGAERASSGGFSDRHSRPRYQAVQHYLLYNPKGRGIPDVSAQGFAIYDHGVSGTSASAPAFAAIIANLNAIRLRANKPVLGYLNPFIYFTDIVHGGCSWNATEGWDPVTGVGTPFRML

>TERG_06625T0

VVEPQGWFKLALQGKTAEFEQRVSNPKHADYGKFMLDAFLQPSPQVKDSVFNWLDWLTFIAQAEKLFNTRFYTFRTLKYSVAASAAPYVQMVQPTTKFTPDCIRELYNRLGISGYLEQYARLDDFSTFIFDFKSIGANEQEASLDVDYAIGLS-GTYYGTAGRNEPIEQLFYLLDLPDSELPAVLSTSYGENEQVPYSSVVCSLFGRLGARGVSVIFSSGDTGVGCQSNDGFNPIFPAACPFVTSVGGEVASSGGFSERFARPWYQDVRHYLMYNPQGRGFPDVSAQSFATRDHGVSGTSASAPLFAGVVSILNSIRLAHNKPRMGFLNPWLYFTDIVHGGCSWNATKGWDPVTGLGTPFEKL

>TESG_02139T0

VIEPEGWFWLAIRENPEKLYDTISTPGRAQYGKHLLDDLLRPRAETSESIVNWLDWVRFVKTAENLMNTRFNVFRTLEYSVPVAISAHVQMIQPTTLFTTACLRELYNRIGVSGFLEEYAQYRDLDLFLFSEGLIGGNTQEANLDMQYVVGLSHKTYYSTAGRNEPLEQLRYLVKLPKNQLPSVLSTSYGETEQLPYTKATCDLFAQLGTMGVSVIFSSGDTGPGCQTNDGFNPIYPASCPFVTSIGGERASSGGFSDRFPRPQYQAVKGYLLFNPNGRAFPDIAAQGYAVYDKGVSGTSASAPTIAAIIAQLNDFRLAKGSPVLGFLNPWIYFTDIVNGGCSWNATKGWDPVTGFGTPFQAL

>TESG_07804T0

VVEPQGWFKLALQGKTAEFEQRVSNPKHADYGKFMLDAFLQPSPQVKDSVFNWLDWLTFIAKAEKLFNTRFYTFRTLKYSVAASAAPYVQMVQPTTKFTPDCIRDLYNRLGISGYLEQYARLDDFSTFIFDFKSIGANEQEASLDVDYAIGLSG-TYYGTAGRNEPIEQLFYLLDLPDSELPAVLSTSYGENEQVPYSSVVCSLFGRLGARGVSVIFSSGDTGVGCQSNDGFNPIFPAACPFVTSVGGEVASSGGFSERFARPWYQDVRHYLMYNPQGRGFPDVSAQSFATRDHGVSGTSASAPLFAGVVSILNSIRLANNKPRMGFLNPWLYFTDIVHGGCSWNATKGWDPVTGLGTPFEKL

>TESG_07897T0

-------MNLAVQQNTRTFEQIVSTPGHRNYGKHLLKGLLRPRRETSNLILSWLDWIHFISKAERMLQTQFYYFRTLKYSIPSHLARHVYMIQPTTKFTPRCLRDLYNVIGVSGYLDQYARYSDFYKFLFSVVHISGNLQEASLDIDYALGLSN-VFYTTSGRNEPLDQLHYLLSLPQEALPAVLSTSYGENEQVPFSHATCNLFAQLGARGVSVIFSSGDSGVGCLTNGKFNPTFPASCPFVTSVGAERASSGGFSDRHSRPRYQAVQHYLLYNPKGRGIPDVSAQGFAIYDHGVSGTSASAPAFAAIIANLNAIRLRANKPVLGYLNPFIYFTDIVHGGCSWNATEGWDPVTGVGTPF---

>tree_111838

---HREWLRIGLQSNIHLGYEKLSDPSSETFGKHLVHDLFAPAHETFDAVHSWLGWLAIVTHVEGLFQTQYHEHGCDQYYVPRHLSEHIDYIVPGIKLTAVCYRALYLEPAVYESGDTFAQ-GDLDSYYPRILSVGGAPVESDIDVNIIQTLVWPVLYQVDDRIDPPDNRPGTALCGAYKPNKVISISYGEGEIVPYFQRQCNEWLKLGLQGTTVLVSSGDFGVACISGSGYNPGNPVSCPYLTSVGAEGASGGGFSNYFPAPDYQAVSKYFIYNRAGRGIPDISANGFRAFNNGWFGTSLAAPLWASIITLINQERTKIGKGSVGFINPVLYLTDIKQGNCGFTAVEGWDPVTGLGTPYPSL

>tree_123865

-LKPD--VSIALPECRELLEQALSDPSSPRYGRYLAKALLRPRREATAAVKRWLQFIHVAEKAQALLGFEYNSTAI--STLPGKIRKHVMTVQYVPLWTPSCLKRLYSLFGIVGFSGQAAQHDELDKFLFSIESVGGSPQEANGDVQYAVAMGYHRYYAVGGELEPLEFASHLLDLDDDELPRVVSISYGANEQFPYAHQVCDMFGQLGARGVSIVVAAGDLGPGCQSNDGFIPSFPATCPYVTSVGSEVASSGGFSDYFARPAWQAVGAYLFYNPAGRGFPDVAAQGFRFRAHGSSGTSLSSPVFAALIALLNDHRSKSGMPPMGFLNPWIYFTDIIEAGCGWSAVPGWDPVTGWGTPFDRM

>tree_70962

VHENARWVRIALQNNLDKGMDYLSHPKSANYGNHYVVDLFAPSPPSVAAVTAWLGWLDFSGQLESLLQTSYNTYGTDEYRLPEEISQHIDFITPGVVFTPQCIRSMYNALGIFETGDTYAQ-QDLTLFWPKVDEIGAAPTESDLDFEIAIPIIYPVLYQAANDDDPIDGTTRNEQCGSFKPTNVISFSYGTAEAYPYLQRQCDEFMKLGLQGKSIVFASGDDGVACL---GFTPGEPASCPYVTSVGAETASSGGFSNIWTTPSYQAIASYFIYNRAGRGYPDVSAVGGVVVNKEEGGTSMSAPLFAGILTRINEERIAAGKSPIGFANPALYFHDITVGGPGFSAVSGWDPVTGLGTPYPAL

>tree_81517

-FDPEGWLSIAVQPYIDSLEARM-------KGNRLVRELQTPAKKDIDNVLHWLDFIRVVAKAEPLLKMKLSRFRTTKYTIPDSVADSISFINPINNFTPSCLSKLYVIFGVAGFLEENANLQDLRQFLINVELVGGNSQEAALDVDYAVSLGFPTFYSTGGRNEPLEFFQYLLAKPDGQVPHVLSLSYSDDELVPYAKRVCSLFGLLTARGTSIIFSSGDGGARCLTNDGTMATFPPTCPWVTSIGAPNGSTGGFSQYFAQPRWQAVEGYVYYNESMRAIPDVSAVGFSIISGGVQGTSASAPVFAAMIALINDARLRAGKKSLGFLNQHLYLQDITAGSCGWPAAEGWDAITGLGVPFDKL

>tree_82623

LLEPHGWLQVALQQNIDQLESRLSTPTSSTYGKYLINSIFAPSDASSSAVESWLSSIWFISTANAMLSTNFHTYRTLKYSIPESLIGHVDLISPTTYFQPDCLRTEYSRIGFGSFLNESASFADQALFEFSVVLIGGDLPEANLDAQTILTIAHPTEFITAGSNEPLQYYEFLLSKSNAEIPQVITNSYGDEEQVPYAVRVCNLIGLLGLRGISVLHSSGDEGVGCVATNSFNPIFPATCPYVTSVGGEVASSGGFSYYFSRPWYQAVGTYLPYDFSGRGFPDVAAHSYPVFQGGSGGTSAASPVVAAIVALLNDARLREGKPTLGFLNPLIYFTDITSGGCHWNATKGWDPTTGFGVPLKKL

>URET_00260

LVEPAGWMKINLASKVEHLHQKVSTPGHASYGQHLIDTLMKPNVTVTESILQWLDWIRVVGVASKLLNARFYEFRTTEYWVPKT---------PSVTFPPNCLRELYNKITVSGYLDQYAQYRDLSLFLFSVSLVGGNTQEANLDIQYVIALSYNEYLSVKGRNEPMNQLEYLHSLPDEDLPTVLTTSYGESEQVPYARATCNEFAKLAARGVSIIFSSGDSGVGCMTNDGFNPIFPATCPWVTSIGSEAASSGGFSNYFARPEWQAVTEYLYYNPHGRGFPDIAAQGYPIYDKGAAGTSASAPTIAAIIAHLNEIRLSQGKPVLGFLNPWLYFTDITNGGCSWNATKGWDPVTGFGTPFEKL

>URET_01816

LAEPDGWLKVSVAEDPNWLYRTLSTPGHPRYGQHMVQAMVAPDPDASDSILAWLDWIDFVDKAERLLNTSFYCFRALEYSLPSNISRHVRTVQPITYFTPASLRQLYNLVGVSGYLNQYARYSDLNLFIFSVELIGGNDQEASLDIQYAISLTYNTYYSTGGLNEPSEQLKYFAGLADDKIPTVLSTSYGENEQVPFAKSVCDEFAKLGARGVSVIFSSGDSGVGCQTNDGFNPIFPASCPFVTSVGGERASSGGFSDIFDRPSYQAISTFLYFNKNGRGFPDVAAQAYSVYDHGVAGTSASAPTIAAVISNLNELRISQGKTVLGFLNPWLYFTDIVDGGCAWDAVKGWDPVTGFGTPFGKL

>URET_02074

VVEPETWLRLAMHDKLEEFEQRVSTPGHETYGQHMVKAFLQPPAHASDAVLAWLDWVHFVEQAEKLLNTKFHYYRTLEYSVPKSVAPYVHMIQPTTKFTPDCLRDLYNKLGISGYLEQFARKGDFAKFLFDVVSIGGNDQEASLDVDYAIGLS-NVYYTTAGRNEPLDQLHYLLSLPDRELPSVLSTSYGENEQVPYTDSACNLFARLGARGVSVIFSSGDTGVACQTNDGFLPIFPAACPFVTSVGAERASSGGFSDRYRRPFYQAVSHYLLYNPRGRGFPDVAAQGFAVVDHDVSGTSASAPAFAAIVANLNAVRLAHGKRVLGFLNPFLYFTDIVHGGCSWNATRGWDPVTGLGTPFQVL

>XP_007807535

-LEPDGWFSIALQPEMHGLASKI---------DGLTRSLRTPAQEDVDHVMRWLDWIRVVGEANKLLDMQLRRYRAPEYNIPDSLSTAIDFIHPIANFTLDCINKLYIRLGIAGFLEQWASYDDTRRSFFSVELIGGNKQEANLDIQFGMAVGFPVYYSTGGRNEPLDLFNYLSSKTNEELPDVLSISYADDELVPYAIRVCNEIGMLASRGVSVLSGSGDGGAKCRSNDGTISTFPASCPWVTSVGANGSSSGGFSAYFERPDWQAVEEYVYYDPNMRAVPDISAVGFRTVINGLQGTSASTPVLAGMIALVNDARVRQGKPVLGWLNKRLYLQDITAGSCGWPATKGYDAITGVGVPFNKF

>XP_007809290

QHEPGGWLSIALQPEIRQLASKF-------GSRHLVRTLRAPDQRDAAAVLDWLDWIHVIRSAESLLGTRLQRYRARQYSVPSHLSGAISFIDPVSNFTPKCLGRLYVRLGVSGYLEEHSNHADVRDFLFGVELVGGDPQEAQLDLEYAMGLGFPTYYATGGRNEPLEFLQALLDKPDGQVPHVLSVSYGDDELVPYAERVCSMLGLLTGRGTSVIHSSGDGGSACRTKDGTMSTFPASCPWVTAVGAPPGSSGGFSH--------------------RAVPDISAVGFLVIVGGLEGTSASAPVFAAMISLVNDARLRKGKPSLGWLNEILYLQDITAGSCGWPAKQGWDAITGLGVPFAKL
